# Integrating machine learning to construct aberrant alternative splicing event related classifiers to predict prognosis and immunotherapy response in patients with hepatocellular carcinoma

**DOI:** 10.3389/fphar.2022.1019988

**Published:** 2022-10-03

**Authors:** Wangrui Liu, Shuai Zhao, Wenhao Xu, Jianfeng Xiang, Chuanyu Li, Jun Li, Han Ding, Hailiang Zhang, Yichi Zhang, Haineng Huang, Jian Wang, Tao Wang, Bo Zhai, Lei Pan

**Affiliations:** ^1^ Department of Interventional Oncology, Renji Hospital, Shanghai Jiao Tong University School of Medicine, Shanghai, China; ^2^ Department of Transplantation, Xinhua Hospital Affiliated to Shanghai Jiao Tong University School of Medicine, Shanghai, China; ^3^ Department of Urology, Fudan University Shanghai Cancer Center, Shanghai, China; ^4^ Affiliated Hospital of Youjiang Medical University for Nationalities, Baise, China; ^5^ Department of Hepatobiliary Surgery, Tenth People’s Hospital of Tongji University, Shanghai, China; ^6^ Department of Hepatic Surgery, Eastern Hepatobiliary Surgery Hospital, Naval Medical University, Shanghai, China; ^7^ State Key Laboratory of Oncogenes and Related Genes, Shanghai Cancer Institute, Reni Hospital, School of Medicine, Shanghai Jiao Tong University, Shanghai, China

**Keywords:** hepatocellular carcinoma, tumor microenvironment, immune checkpoint molecules, alternative splicing event, machine learning

## Abstract

**Introduction:** In hepatocellular carcinoma (HCC), alternative splicing (AS) is related to tumor invasion and progression.

**Methods:** We used HCC data from a public database to identify AS subtypes by unsupervised clustering. Through feature analysis of different splicing subtypes and acquisition of the differential alternative splicing events (DASEs) combined with enrichment analysis, the differences in several subtypes were explored, cell function studies have also demonstrated that it plays an important role in HCC.

**Results:** Finally, in keeping with the differences between these subtypes, DASEs identified survival-related AS times, and were used to construct risk proportional regression models. AS was found to be useful for the classification of HCC subtypes, which changed the activity of tumor-related pathways through differential splicing effects, affected the tumor microenvironment, and participated in immune reprogramming.

**Conclusion:** In this study, we described the clinical and molecular characteristics providing a new approach for the personalized treatment of HCC patients.

## Introduction

Hepatocellular carcinoma (HCC) has become the second leading cause of cancer-related deaths worldwide, with more than 800,000 deaths each year (Sung et al., 2021). Surgical resection, liver transplantation, tumor ablation, and interventional techniques are all potential treatment methods (Bishay et al., 2016; Guro et al., 2016; Sun et al., 2019; Yoon and Lee, 2019). However, improvements in the prognosis of liver cancer remain challenging. The therapeutic effects of first-line HCC drugs such as sorafenib are poor (Galun et al., 2017; Saffo and Taddei, 2019), and no prognostic classification and markers have been identified to provide guidance for personalized treatment (Liu et al., 2020a; Zhao et al., 2021a; Zhao et al., 2021b). Therefore, a new treatment strategy is required to predict the prognosis of liver cancer.

Aberrant alternative splicing (AS) is the result of splicing regulatory sequence mutations or ectopic RNA binding protein regulation. It plays an indispensable role in cancer and many other diseases (Gamundi et al., 2008; Fu and Ares, 2014; Shiraishi et al., 2018). Although integrated multiomics analyses have been reported in HCC subtypes, splicing characteristics and splicing regulatory networks are rarely systematically discussed. We previously studied the regulatory mechanism of AS-related genes and their effect on the prognosis of some malignant tumors (Liu et al., 2020b). On this basis in the current study, we conducted a comprehensive analysis of HCC subtype classification and splicing characteristics and their relationship with clinical characteristics, gene mutations, pathway changes, and immune heterogeneity.

## Materials and methods

### Patients and tissue samples from online databases and real-world cohorts

All splicing data for liver cancer were downloaded from the cancer genome atlas (TCGA) SpliceSeq database including AS data, expression data, phenotype data, and survival data (Supplementary Table S1).

We also downloaded the human genome sequence from the TCGA database (Barrett et al., 2013), the human gtf file from the Ensembl database (Yates et al., 2020), and the Gene Set Variation Analysis (GSVA) gene set (Hänzelmann et al., 2013) and immune cell-related gene set.The variable splicing score was calculated by the network tool Maximum Entropy (Kim et al., 2018).

### Sample clustering and survival differences

We used the R package Rtsne (v0.15), which based on the t-distributed stochastic neighbor embedding (t-SNE) method, to cluster the samples according to their PSI values (Chen et al., 2021). Because the clinical feature grouping is displayed in t-SNE, the sample division was not obvious. Therefore, the R package ConsensusClusterPlus (v1.50.0) was used to perform unsupervised clustering of the samples (Zheng et al., 2020). The Kaplan–Meier algorithm was used to obtain the PSI-based AS subtype, and t-SNE was undertaken for verification and presentation of the results, followed by analysis by the R packages survival (v3. 2–7) and survminer (v0.4.8) to determine the survival of the samples and construct Kaplan–Meier curves (Rizvi et al., 2019; Wang et al., 2020).

To further detect the differences in the distribution of age, sex, grade, pathologic T stage, alcohol, hepatitis B, and hepatitis C groups in the AS subtypes, Fisher test was applied (Di Francesco et al., 2019).

### Identification and presentation of subtype differences in AS events, and analysis of the differential alternative splicing events

DASEs of cancer samples and normal samples were called according to the PSI value of AS. DASEs met two conditions: 1) Wilcoxon rank-sum test between groups reached a significant level (after Bonferroni correction adjustment *p* < 0.05); and 2) Chi-squared test based on the median PSI reaching a significant level (*p* < 0.05). After DASEs were obtained, those whose average PSI of cancer samples were greater than the average PSI of normal samples were regarded as upregulated, and those whose PSI were less were regarded as downregulated. Next, DASEs between samples of different subtypes and normal samples were collected in the same way, and the number of different AS types in the relevant subtypes was counted, with the results included in a histogram. After obtaining the DASEs between subtypes, the overlap similarity of the upregulated and downregulated DASEs between subtypes was calculated as follows: Overlapping similarity = intersection of two sets/minimum value of the two sets. According to the obtained DASEs, analysis of variance was used to screen differential AS events between the two subtypes, with a threshold of *p* < 0.05, and then the intersection was taken for subsequent analyses.

### Analysis of splicing characteristics of DASEs in alternative splicing subtypes, corresponding gene expression display and GSVA difference analysis

In a further analysis of the AS score, GC content, and AS fragment length of DASEs, Python 3.8.8 (Spyder (Anaconda3)) was first used to obtain the reference sequence of each chromosome from the reference genome (Paillusseau et al., 2020), and the splice site positions provided by TCGA SpliceSeq database were combined to obtain all DASEs with alternate acceptor site (AA), alternate donor site (AD), exon skip (ES), retained intron (RI) sequence, and 5′ or 3′ splice site sequence splicing types. To calculate the AS score, the first 3′ position sequence of AA, the second 3′ position sequence of AA, the first 5′ position sequence of AD, and the second 5′ position sequence of AD were extracted according to the requirements of MaxEntScan. The ' site sequence, the 5′ and 3′ site sequence of ES, and the 5′ and 3′ site sequence of RI were analyzed online to obtain the score of the corresponding site, which was shown in a box plot. The GC content was the percentage of G and C bases in the entire AS sequence. Alternative splicing length = log10 (exon/intron length). Finally, a box plot was drawn to show the GC content and AS length. We identified genes corresponding to DASEs from the AS information, and then drew a heat map to show the expression of the corresponding gene [log2 (fpkm-uq+1)].

All gene sets were downloaded from the MSigDB database (Guo and Wan, 2014), and the R package GSVA (v1.34.0) was used to calculate the enrichment scores of each sample for different gene sets according to the expression data, and the cumulative distribution curve of GSVA scores was drawn according to the different subtypes. Then the R package limma (v3.42.2) was used to obtain the enriched differential gene sets (DESs) in different subtype samples and normal samples (Ritchie et al., 2015), and the threshold to |logFC|> 0.5 and adj.P.Val<0.05 was called. A bar graph was drawn to plot the upregulated and downregulated adjustments in different subtypes of DESs.

### Analysis of the correlation between differential gene sets, AS events, and AS factors

The correlation between the differential gene sets within the subtypes and the AS events was further calculated. First, the AS event was selected according to a PSI interquartile range of greater than 0.05 as the threshold in the samples, and then the screening results were used to calculate the Spearman’s correlation coefficient (coef) for the differential gene set. Alternative splicing-related pathways (SPs) in the MSigDB database were searched using “splice”, “splicing”, and “spliceosome” as keywords, and protein-coding genes in related pathways were used as splicing factors (SFs). After that, Spearman’s correlation coefficients of AS events and SPs and SFs were further calculated, and then the largest |coef. of SP| and |coef. of SF| corresponding to each AS event was selected to construct a scatter plot. Because coef. of SP and coef. of SF had the greatest number of AS events greater than 0.5 at the same time, the relevant AS events were selected for further PSI display, as well as the strongly correlated SP enrichment score of each subtype and the strongly correlated SF differential expression [edgeR (v3.28.1)] (Robinson et al., 2010). The R package estimate (v1.0.13) was used to calculate the StromalScore, ImmuneScore, ESTIMATEScore, and TumorPurity of all samples in TCGA-LIHC data, and the immune cell-related gene set was used to calculate the enrichment scores of 28 immune infiltrating cells, combined with immune checkpoints.

### Combining mRNA expression profiles to predict differences in AS typing immunotherapy and drug sensitivity

To predict whether an immunosuppressive agent has a therapeutic effect on different AS subtypes, SubMap was used to map different AS subtype samples to samples with inhibitor processing information (Ay et al., 2011), as well as to calculate the similarity between the samples and then predict the possible effects of variant splicing subtypes on treatment with two inhibitors.

The R package pRRophetic (v 0.5) was then to predict the sample’s response to 138 drugs (Geeleher et al., 2014), generating predicted IC_50_ values, and then the differences in the IC_50_ value of the samples of different AS subtypes was further counted using Kruskal’s algorithm to detect the significant differences. Next adj.*p* < 0.05 was used to screen for significantly different drugs, and the IC_50_ values of bosutinib, dasatinib, midostaurin, elesclomol, pazopanib, bortezomib, sorafenib, docetaxel, and gefitinib were plotted in box plots.

### Construction of a prognostic model of AS

Cancer samples were collected according to the PSI value of differential AS in the previous step combined with OS data, and batch Cox one-way regression analysis was performed on differential AS. After regression analysis, *p* < 0.05 was used as a threshold to screen significantly related AS events for subsequent analyses.

Lasso regression was further performed on the single-factor Cox regression results and a risk scoring model was built. This process mainly relied on the R package glmnet (v4.0–2). In the glmnet function (Engebretsen and Bohlin, 2019), Y is Surv (time, event), and family is Cox. To build a more accurate regression model, we first used cross-validation for lambda screening, then selected the model corresponding to lamdba.min, and further extracted the expression matrix of related genes in the model, and then calculated the risk score of each sample according to the following formula:

$$\mathrm{RScor}e_{i}=\overset{n}{\underset{j=1}{\sum }}\mathrm{PS}I_{\mathrm{ji}}\times \beta_{j}$$

Where PSI represents the PSI value of the corresponding AS, β represents the regression coef. of the corresponding gene in the lasso regression result, and RScore represents the PSI value of the significantly related AS event in each sample multiplied by the corresponding AS event. The coef was then calculated, where i is the sample and j is the AS event. On the basis of the risk score of the sample, the high and low risk groups were divided by the median as the node and combined with the overall survival (OS) and disease-free interval (DFI) data to generate a Kaplan–Meier curve, with a *p*-value of <0.05 indicating that the difference between the high and low risk groups was significant.

### Validation in human HCC tissues

Paired tumoral and adjacent normal samples were from patients diagnosed with HCC and accepted surgery at the Department of Transplantation, Xinhua Hospital affiliated to Shanghai Jiao Tong University School of Medicine (Shanghai, China) after the written informed consent. All these samples were kept and processed as previously described. RT-PCR was implemented using transcripts specific primers by 2 × Green PCR Mix (Vazyme, Nanjing, China). Splicing specific transcripts were distinguished using agarose gel electrophoresis and grayscale-measured using software ImageJ (Rawak Software Inc., Stuttgart, Germany). PSI of each lane was calculated by the greyscale of the longer transcript divided by the sum greyscale of the longer and the shorter transcripts.

## Results

### Splicing clustering and clinical features of HCC subtypes

HCC AS data (percentage of samples with PSI value = 100%) was downloaded from TCGA SpliceSeq database, and 11,179 AS events were obtained, corresponding to 4423 genes, which included 568 AAs, 469 ADs, 968 alternate promoters (APs), 6346 alternate terminators (ATs), 1992 ESs, 29 mutually exclusive exons (MEs), and 807 retained introns (RIs). At the same time, relevant HCC expression data and clinical data were downloaded from the UCSC Xena database, and 370 cancer samples and 50 normal samples were obtained after integration.

**FIGURE 1:**
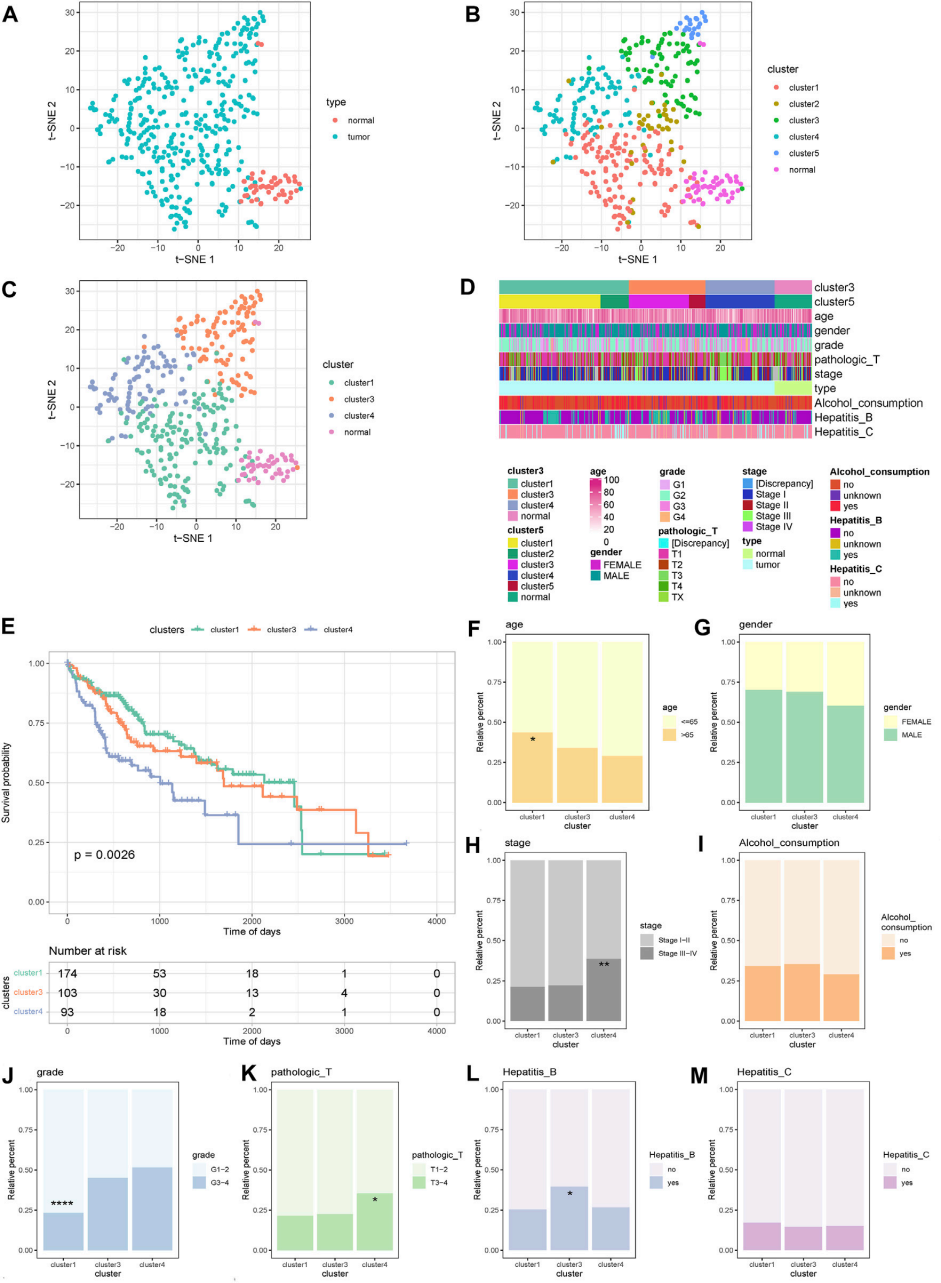
Sample cluster display and survival differences and clinical analysis. **(A)** Cluster scatter plot display of cancer samples and normal samples. **(B)** Unsupervised clustering scatter plot display of cancer samples in 5 categories. **(C)** Scatter plot display of 5 categories of samples merged into 3 categories.**(D)** Clustering Heat map display of samples and clinical traits. **(E)** KM curves of 3 alternative splicing subtypes. **(F**–**M)** Display of clinical features and distribution of typical types. ****p < 0.0001; ***p < 0.001; **p < 0.01; *p < 0.05.

According to the t-SNE method, the PSI values of all samples were displayed in clusters, and a scatter plot revealed that the cancer samples could be clearly distinguished from the normal samples (Figure 1A). Next, unsupervised clustering of cancer samples was performed to obtain five subtype samples (Figure 1B). The t-SNE method was used to demonstrate that cluster 1 and cluster 2 samples were relatively similar in the five subtype samples, and cluster 3 and cluster 5 samples were similar. Therefore, cluster 1 and cluster 2 were merged into cluster 1, and cluster 3 and cluster 5 were merged into cluster 3, and finally three subtypes (cluster 1, cluster 3, cluster 4) were obtained. The scatter plot shows that the three subtypes were more distinct from each other (Figure 1C).Then, we analyzed the correlation between cluster samples and clinical traits such as age, sex, grade, pathological stage, type, alcohol consumption, and hepatitis B/C infection, and found that AS also affects various clinical characteristics of HCC (Figure 1D).

### Analysis of differences in survival between subtypes, clinical characteristics, and distribution of typical types

The survival analysis of the three subtypes was further based on OS, and a Kaplan–Meier curve was generated. The results showed that the survival difference of the three subtypes was significant, and the survival curve of cluster 4 samples dropped faster (Figure 1E).

The distribution differences of age, sex, grade, pathological T stage, alcohol consumption, hepatitis B, and hepatitis C groupings in AS subtypes were further examined (Figures 1F–M). The results showed that there were significant differences in the distribution of age, grade, pathological T stage, and hepatitis B groupings among the subtypes. For age, cluster 1 was more than 65 years old and had significantly more samples than cluster 4 (Figure 1F). For grade, the number of G1–2 samples in cluster 1 was significantly higher than that in cluster 3 and cluster 4 (Figure 1J). For pathological T stage, the number of T3–4 samples in cluster 4 was significantly higher than in cluster 1 (Figure 1K). For stage, the number of stage III–IV samples in cluster 4 was significantly higher than that in cluster 1 and cluster 3 (Figure 1H). For hepatitis B, there were significantly more hepatitis B patients in cluster 3 than cluster 1 (Figure 1L). These data indicate that AS exhibits different patterns according to the histological type of HCC and is closely related to clinical characteristics and patient survival, and thus is suitable as a subtype classification.

### Overall differences in AS events and identification of subtype differences in AS events

**FIGURE 2:**
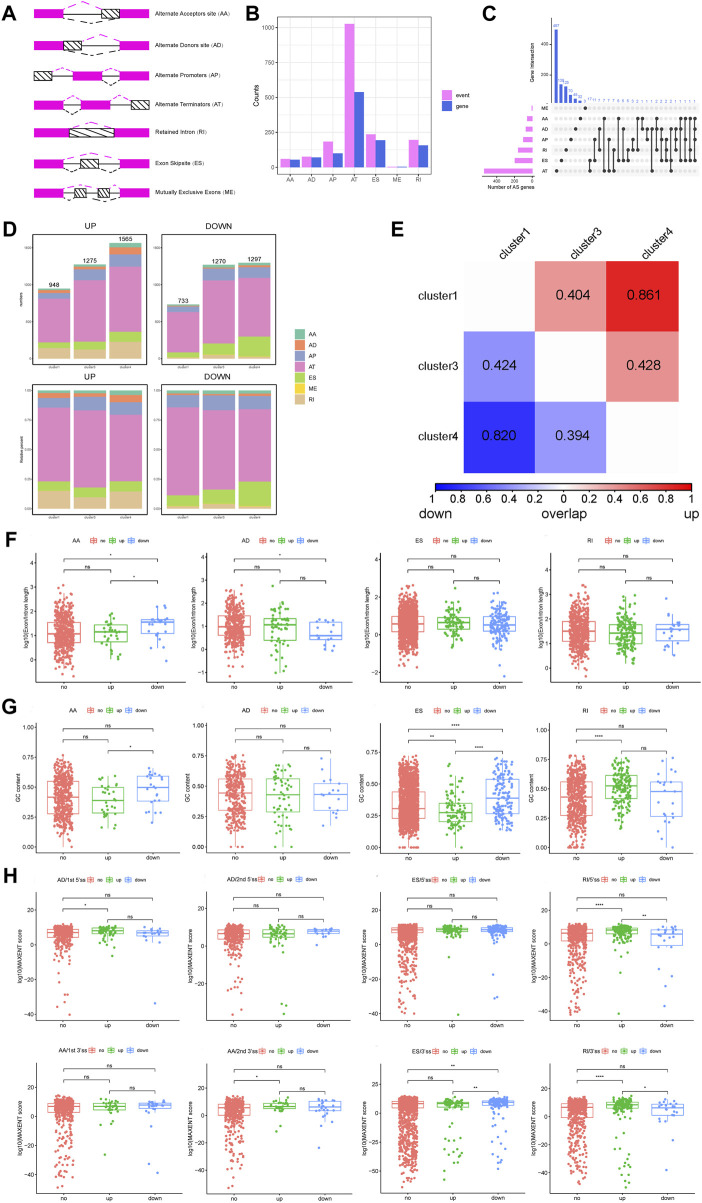
Analysis of DASEs and DASEs between subtypes and alternative splicing fragment length and score of the overall DASEs. **(A)** Schematic diagram of alternative splicing types. **(B)** Statistics of the number of splicing types of DASEs and corresponding gene splicing types. **(C)** UpSet diagram of the different alternative splicing types of DASEs corresponding genes. **(D)**Statistics of alternative splicing types of DASEs of each subtype. **(E)**Overlap similarity of DASEs up and down between subtypes. **(F)**Alternative splicing fragment length of overall DASEs. **(G)** GC content of overall DASEs. **(H)** Alternative splicing score for overall DASEs.

On the basis of the PSI value of AS, the DASEs of cancer samples and normal samples were retrieved. After threshold screening, 1,777 DASEs were obtained, of which 977 were upregulated and 800 were downregulated, corresponding to 1,005 genes (Supplementary Table S2). According to the type of AS, DASEs had the most AT events, followed by ES, RI, and AP, and the corresponding genes also had the most AT events. The UpSet chart showed that 17 genes had AT and ES at the same time, and 11 genes had AP and ES at the same time (Figures 2A–C, Supplementary Table S3).

**FIGURE 3:**
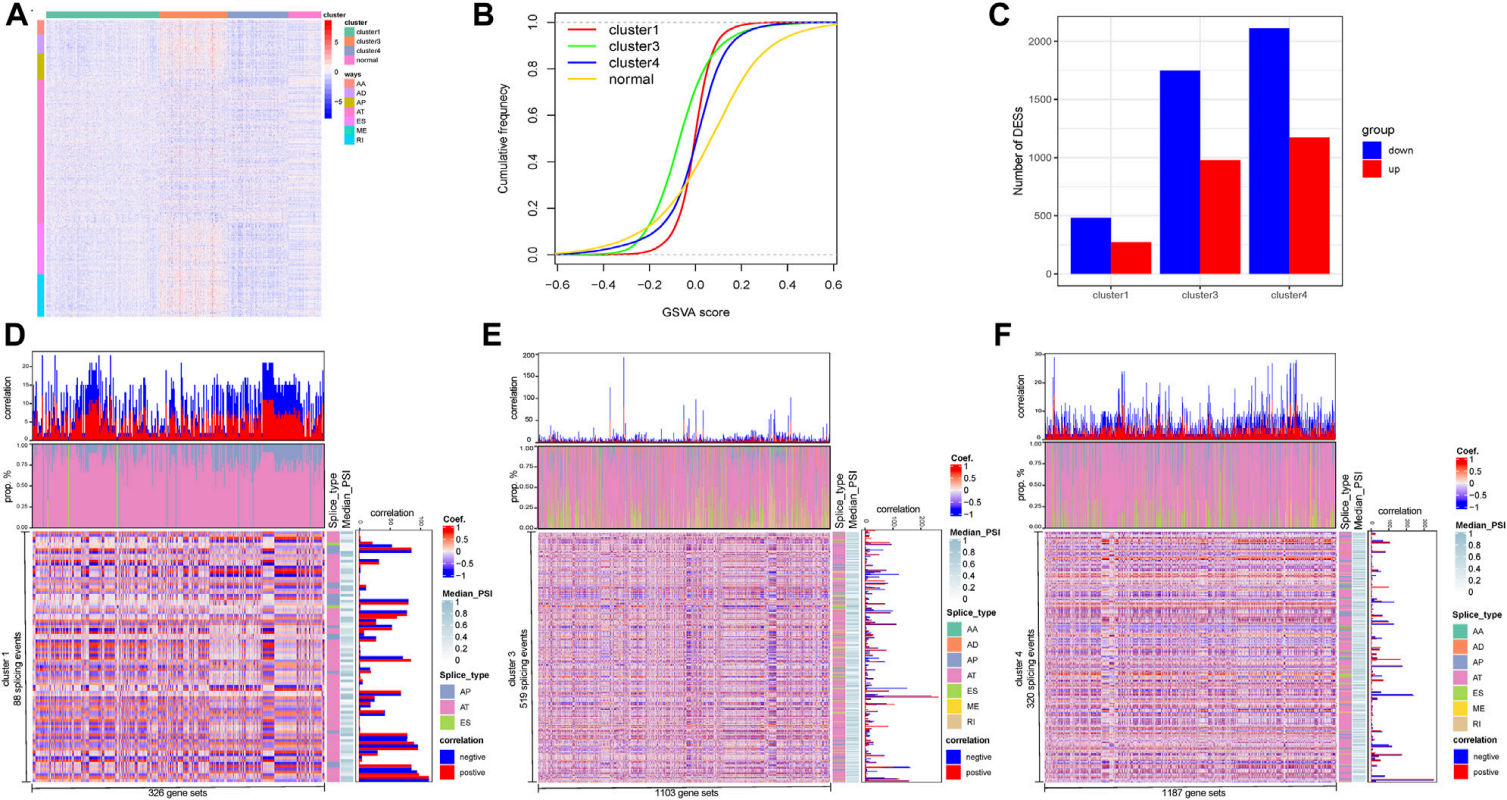
Analysis of DASEs Corresponding Genes and Alternative Splicing Events and GSVA Analysis. **(A)** Heat map of DASEs corresponding gene expression. **(B)** Statistics of the number of differential enrichment pathways of each subtype compared with normal samples. **(C)** We exploited a Nomogram to evaluate the prognosis of HCC with prediction model of POLD1 expression and pTNM stage. **(D**–**F)** Display of strong correlation between differential gene sets and alternative splicing events.

Next, the DASEs between the three subtypes and the normal samples were obtained. Cluster 1-normal had 1,681 DASEs, including 948 that were upregulated and 733 that were downregulated; cluster 3-normal had 2,545 DASEs, including 1,275 that were upregulated and 1,270 that were downregulated; and cluster 4-normal had 2,862 DASEs, including 1,565 that were upregulated and 1,297 that were downgraded. (Figure 2D, Supplementary Table S4). After obtaining the DASEs between the subtypes, the overlap similarity of the upregulated and downregulated DASEs was calculated between the subtypes. The heat map in Figure 3E shows that in cluster 1 and cluster 4, regardless of the upregulation or downregulation of DASEs, the overlap similarity was relatively high, at greater than 0.8 in both (Figure 2E).

### Analysis of splicing characteristics of DASEs in AS subtypes

The AS score, GC content, and AS fragment length of the overall DASEs were further analyzed. For the length of AS fragments, the AS length of downregulated AAs was significantly higher than that of upregulated AAs and unchanged AAs, while the AS length of downregulated ADs was significantly lower than that of unchanged ADs, ESs, and RIs. There were no significant differences among the three groups (Figure 2F).

For GC content, the GC content of downregulated AAs was significantly higher than that of upregulated AAs, and the GC content of downregulated ESs was significantly higher than that of upregulated ESs and unchanged ESs. The GC content of upregulated RIs was significantly higher than that of upregulated RIs (Figure 2G).

For the AS score, the score of the first 5′ site of upregulated ADs was significantly higher than the score of the first 5′ site of unchanged ADs (Figure 2I), and the score of the second 3′ site of upregulated AAs was significantly higher than the score of the second 3′ locus of unchanged AAs (Figure 2H). Conversely, the score of the 3′ locus of downregulated ESs was significantly higher than that of the other two groups (Figure 2H). Furthermore, the score of the 5′ locus of upregulated RIs was significantly higher than the score of 5′ sites with downregulated RIs and 5′ sites with no change in RIs, and the score of 3’ sites with upregulated RIs was also significantly higher than the other two groups (Figure 2H).

### Correlation analysis of DASE-corresponding genes in AS subtypes

Next, we analyzed the total DASEs corresponding to 1,005 genes, and drew heat maps based on the expression of related genes. The heat map showed that the overall gene expression of cluster 3 samples was high, while the overall gene expression of cluster 4 samples was low (Figure 3A).

A total of 32,284 gene sets was downloaded from the MSigDB database. The enrichment score of each sample for all gene sets was obtained and the cumulative distribution curve of GSVA scores according to different subtypes was generated. The score curve showed that compared with normal samples, the GSVA score distribution of samples of other subtypes was relatively. The score distribution of cluster 1 samples among subtypes was the most concentrated, while cluster 3 samples had more scores of less than 0, and cluster 4 had more scores of greater than 0 (Figure 3B). After obtaining the DESs of different subtype samples and normal samples, for all subtypes, there were more downregulated enrichment pathways than upregulated enrichment pathways, and cluster 4 samples and normal samples had the most different pathways (Figure 3C, Supplementary Tables S5, S6).

Correlations between differential gene sets within subtypes and AS events were further calculated. According to the interquartile range of the sample PSI, 3,662 AS events were screened, and strong correlations were screened from each subtype according to a Spearman’s correlation coefficient greater than 0.6. Eighty-eight splicing events and 326 gene sets in cluster 1 samples had a strong correlation, 519 splicing events in cluster 3 samples and 1,103 gene sets had a strong correlation, and 320 splicing events and 1,187 gene sets in cluster 4 samples were strongly correlated (Figures 3D–F). The above research showed that cluster 4, which had the most splicing events, gene sets, and differential pathways, was closely related to worst overall survival. These results partly describe the differences between the internal and external environments of HCC subtypes and normal tissue cells and the corresponding different splicing regulatory mechanisms.

### Correlation analysis of AS pathways, AS events, and AS factors

**FIGURE 4:**
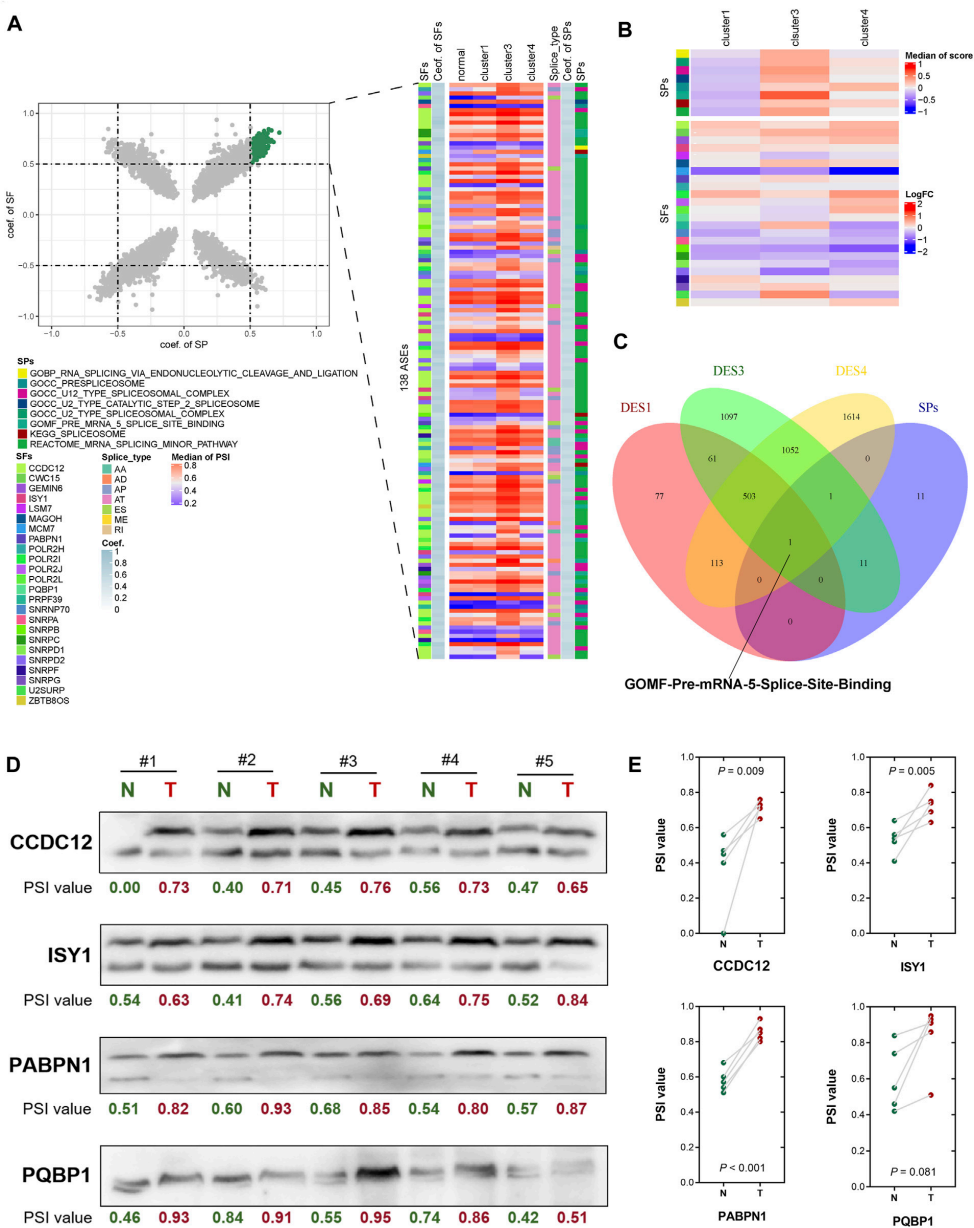
Correlation analysis of alternative splicing pathways, alternative splicing events and alternative splicing factors. **(A)** Scatter plot showing the association between alternative splicing events and SPs and SFs. **(B)** Heat map showing the association between alternative splicing events and SPs and SFs. **(C)** Venn diagram shows the common pathways of SPs and differential pathways. **(D)** Differentially expressed splicing transcripts of CCDC12, ISY1, PABPN1 and PQBP1 were validated in human HCC tissues by RT-PCR and consequent agarose gel electrophoresis. PSI of each lane was calculated by the greyscale of the longer transcript divide the sum greyscale of the longer and the shorter transcripts. **(E)** Significance of difference between HCC tumor and adjacent normal tissues for splicing of CCDC12, ISY1, PABPN1 and PQBP1 were evaluated separately by two-tailed paired *t*-test.

Twenty-four SPs and 370 SFs were identified from the MSigDB database. In further calculations of the Spearman’s correlation coefficients of AS events and SPs and SFs, a scatter plot showed that the coef. of SP and coef. of SF at the same time were greater than 0.5 AS events, including 138 AS events in each subtype. A heat map of the median of related events showed that the PSI value of cluster 3 samples was higher than that of the other subtypes, and the enrichment score of SPs of cluster 3 samples was higher. For SFs, in the cluster 4 samples, some SFs were significantly downregulated (Figures 4A,B; Supplementary Table S7). A Venn diagram of SPs and differential pathways showed that there was one pathway in common, namely GOMF_PRE_MRNA_5_SPLICE_SITE_BINDING (Figure 4C). Therefore, these subtype-specific changes in HCC, including pathway activation and SF expression, may be related to its severe abnormal splicing (Supplementary Table S8). To characterize the splicing-based mechanisms that may contribute to the relative malignancy of HCC, we performed analyses according to the up- and down-regulation of DASE-related gene formation in subtypes. We selected PABPN1, CCDC12, ISY1 and PQBP1 for analysis based on the effect of SFs on survival in HCC. The ΔPSI values were determined for 25 each tumor-normal pair, and eight out of nine AS events showed a significant positive correlation (*p* < 0.01).

The longer spliced isoforms of these SFs were significantly overexpressed in all HCC subtypes and validated by RT-PCR in HCC tissues (Figures 4D,E). These may suggest that longer transcripts of PABPN1, CCDC12 and ISY1 are important for maintaining cancer cell survival (*p* < 0.01). The difference in PSI of PQBP1 was not significant (*p* = 0.081), but the trend was consistent with the other three SFs. Therefore, the upregulation of PSI in HCC may be responsible for the upregulation of SFs such as PABPN1, CCDC12, ISY1 and PQBP1. Overall, the heterogeneity and homogeneity of splicing changes in HCC-related pathways may suggest a distinct role for alternative splicing in tumorigenesis and maintenance of cancer cell survival. Irregular splicing may regulate isoform switching of genes in cancer biological pathways and mRNA expression to promote HCC infiltration and invasion.

### Immune-related and clinically relevant analysis of AS subtypes

**FIGURE 5:**
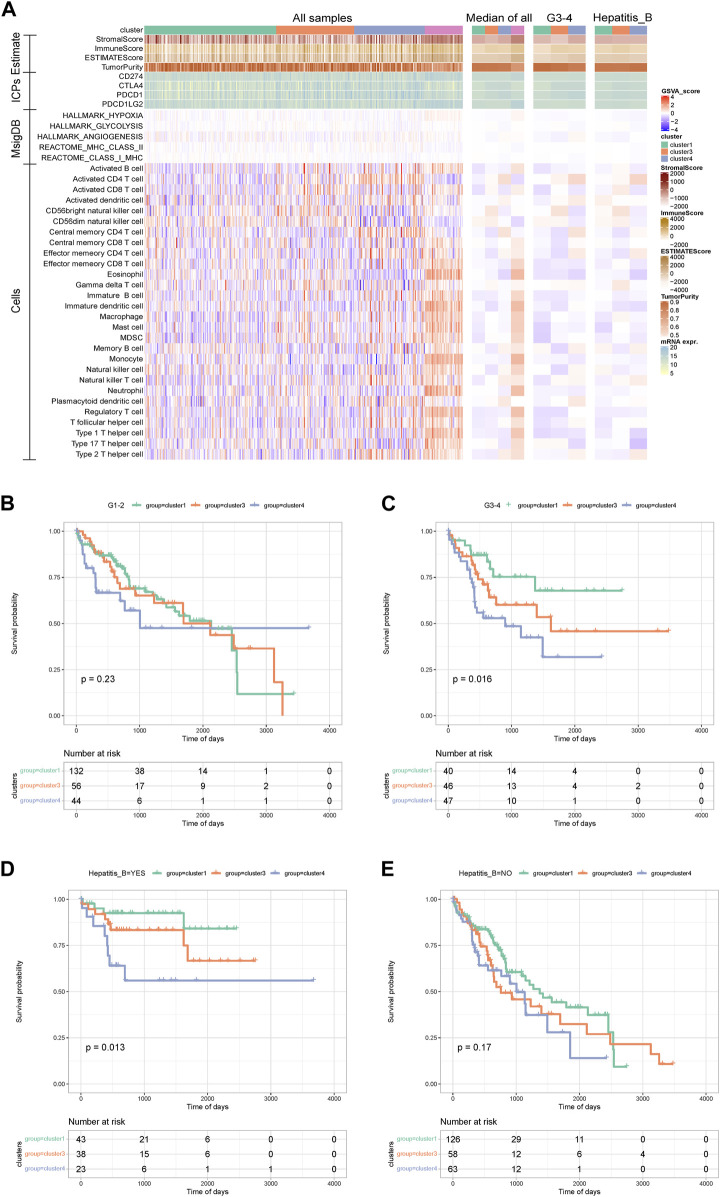
Analysis between alternative splicing subtypes and immunity and clinical survival. **(A)**The heat map shows the immune status of alternative splicing subtypes. **(B**–**E)** KM curve between grade group and Hepatitis_B group.

We further explored the immune status of AS subtypes. A heat map showed that the StromalScore, ImmuneScore, and ESTIMATEScore of the cluster 3 samples were slightly lower than the other two subtypes. From median data, the tumor purity of cancer samples was significantly higher than that of normal samples, and the immune-related scores were significantly lower than normal samples. There was no significant difference in the expression of immune checkpoints and the enrichment scores of related pathways in each subtype. For most immune cells, the immune cell score of normal samples was significantly higher than that of cancer samples. In addition, cluster 4 samples had a higher activated CD4 T cell enrichment score and a lower activated CD8 T cell enrichment score. For G3–4 and hepatitis B patients, the related differences were also obvious (Figure 5A).

Kaplan-Meier analysis was further performed within the group for grade grouping and hepatitis B grouping. This showed that for the G3–4 samples, the Kaplan–Meier curves between the internal AS subtypes were significantly different, and for the samples with hepatitis B, the Kaplan–Meier curves between the internal AS subtypes were significantly different (Figures 5B–E). The above results suggest that the anti-tumor immune response produced by SF can be offset by the tumor micro environment (TME), and aggressive cancer cells with a large number of intracellular mutations and tumor-associated antigens survive immune reprogramming. Therefore, blocking these immunosuppressive molecular pathways should be combined with immunotherapy against neoantigens to regulate the immune response of HCC patients.

### Combining mRNA expression profiles to predict differences in AS for immunotherapy and drug sensitivity

Using SubMap to predict the possible therapeutic effects of related immunosuppressive agents on different AS subtypes, the results showed that the similarity between cluster 4 samples and CTLA4 response samples reached a significant level, suggesting that CTLA4 inhibitors may have a better effect on cluster 4 samples. (Supplementary Figure S1).

**FIGURE 6:**
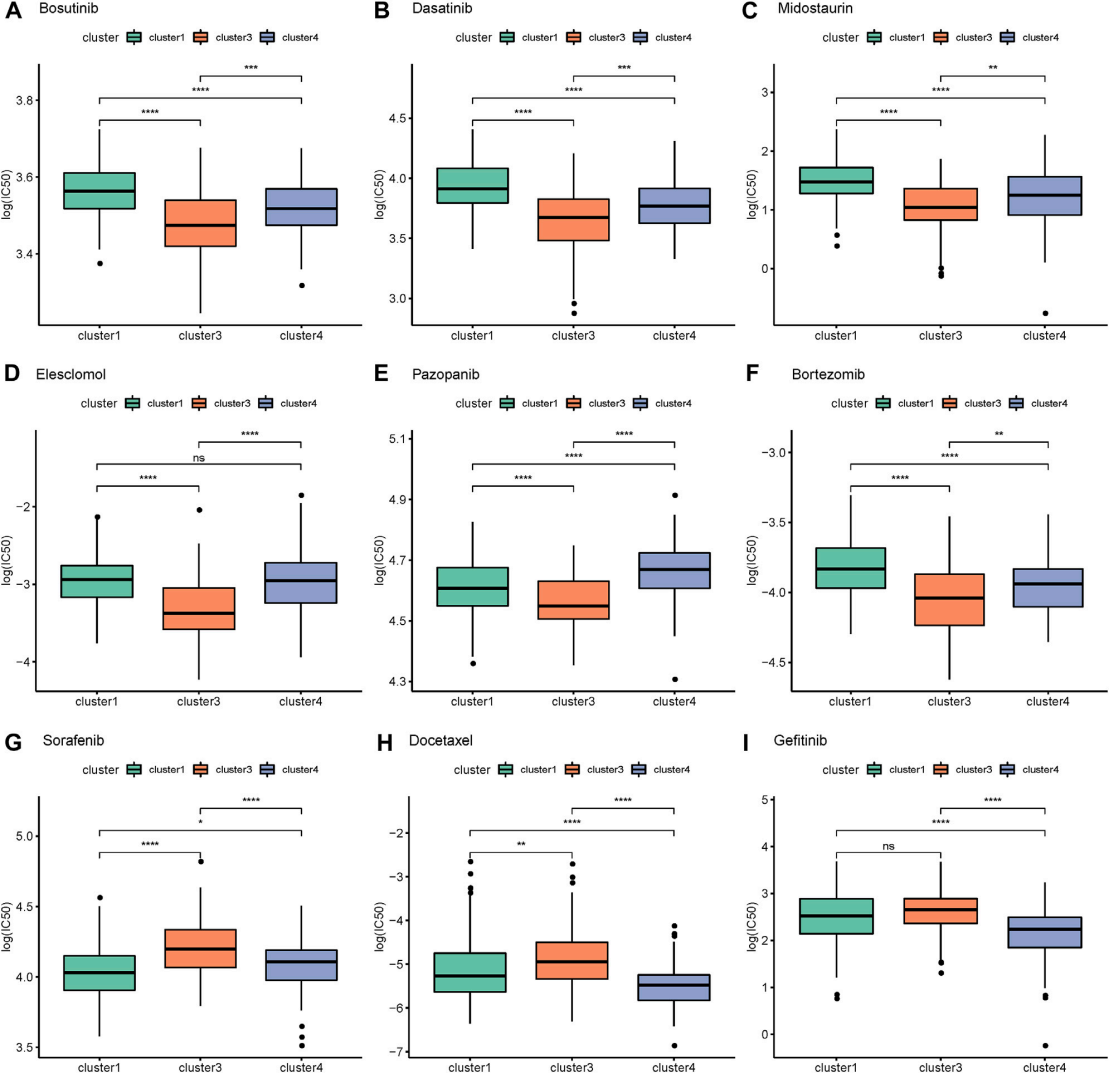
Response of alternative splicing subtypes to drugs. **(A)** Bosutinib **(B)** Dasatinib **(C)** Midostaurin **(D)** Elesclomol **(E)** Pazopanib **(F)** Bortezomib **(G)** Sorafenib **(H)** Docetaxel **(I)** Gefitinib.

We further predicted the samples’ responses to 138 drugs to obtain predicted IC50 values. The results showed that there were significant differences in the degree of response of 111 drugs among the different subtypes (Supplementary Table S9). The IC50 values of bosutinib, dasatinib, midostaurin, elesclomol, pazopanib, bortezomib, sorafenib, docetaxel, and gefitinib were plotted in box plots. For bosutinib, dasatinib, midostaurin, elesclomol, pazopanib, and bortezomib, the efficacy of cluster 1 and cluster 4 was relatively good and the efficacy of cluster 3 was relatively poor, while cluster 3 mainly responded well to sorafenib, docetaxel, and gefitinib (Figures 6A–I) 0Screening of AS events and construction of a prognostic model of AS.

**FIGURE 7:**
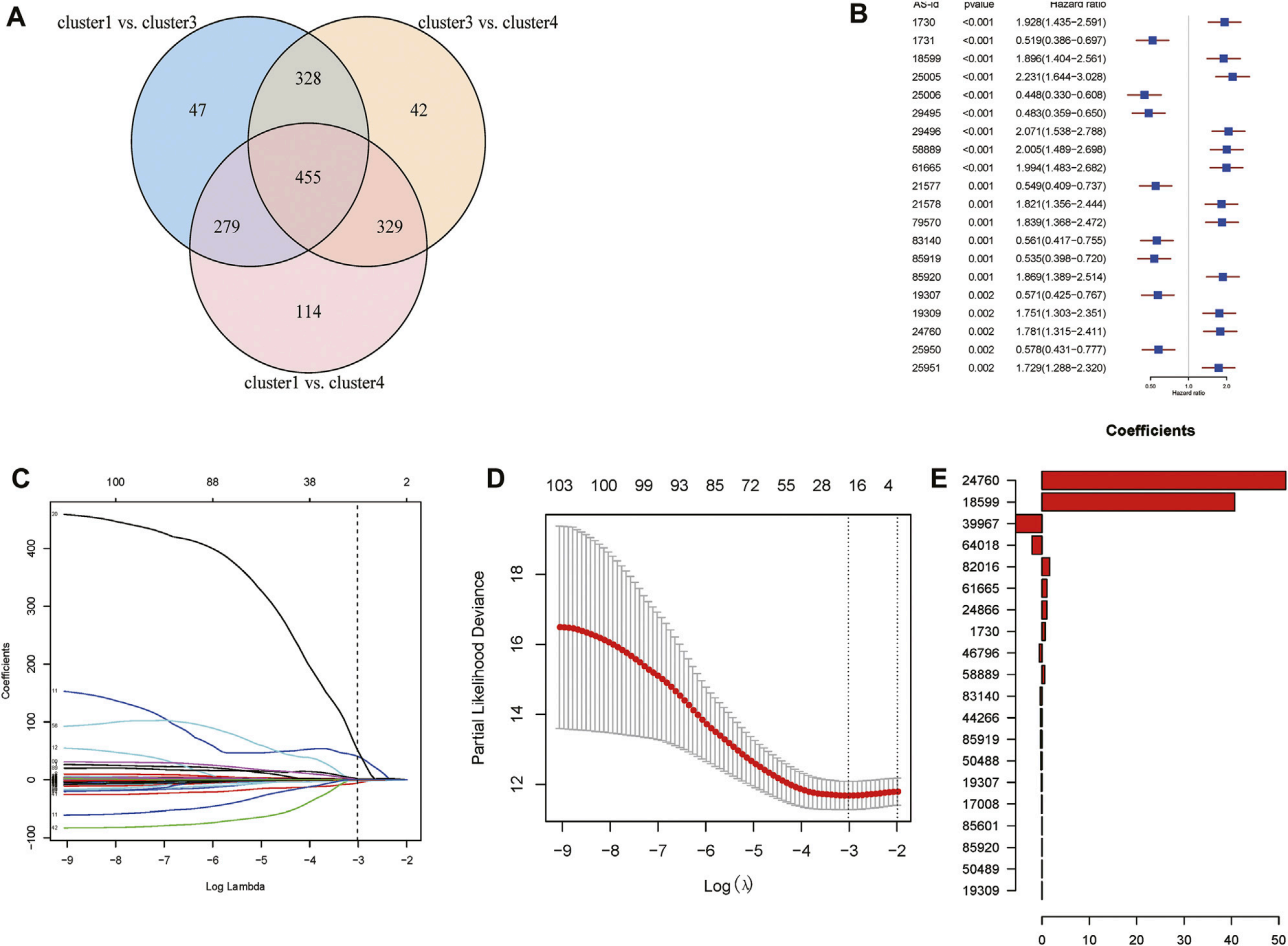
Analysis of Differential Alternative Splicing Events between Subtypes. **(A)**Venn diagram of alternative splicing events for differences between subtypes. **(B)** Top 20 single factor cox regression results. **(C)** Display the corresponding changes of lambda and variable coefficients. **(D)** Obtain lambda.min through cross-validation. **(E)** Display the regression coefficients corresponding to the variables after screening.

According to the obtained PSI values of DASEs, the differential AS events between subtypes were screened. There were 1,109 DASEs between cluster 1 and cluster 3, 1,154 DASEs between cluster 3 and cluster 4, and 1,177 DASEs between cluster 1 and cluster 4. Intersection of the three clusters resulted in a total of 455 DASEs for subsequent analysis (Figure 7A). Using cancer samples, on the basis of PSI values of the shared DASEs obtained in the previous step combined with the OS data, batch Cox one-way regression analysis was performed on differential AS, and 111 survival-related DASEs were obtained (Figure 7B).

Lasso regression was then performed on 111 survival-related DASEs, and 20 AS events were obtained to construct a risk model. Regression coefficients and PSI values were analyzed to obtain the following risk scores: PSI39967 * (−5.5748) + PSI64018 * (−2.0928) + PSI46796 * (−0.5633) + PSI83140 * (−0.3544) + PSI44266 * (0.3162) + PSI85919 * (−0.3101) + PSI50488 * (−0.2602) + PSI19307 * (−0.1502) + PSI17008 * (−0.0950) + PSI19309 * (1.0474) E−13) + PSI50489 * 0.0014 + PSI85920 * 0.0023 + PSI85601 * 0.0223 + PSI58889 * 0.5534 + PSI1730 * 0.6888 + PSI24866 * 1.0308 + PSI61665 * 1.0423 + PSI 82016 * 1.6102 + PSI18599 * 40.6609 + PSI24760 * 51.4456 (Figures 7C–E).

### Further validation of the prognostic model of AS

**FIGURE 8:**
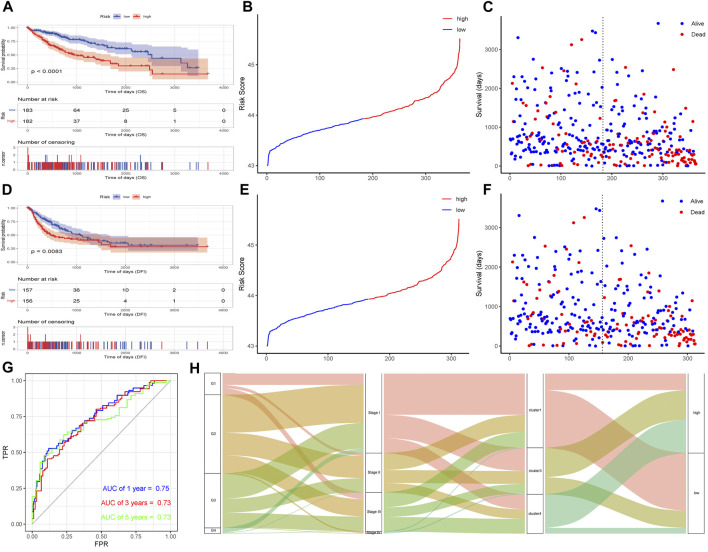
Effectiveness verification of a risk model based on PSI events. **(A)**Build model KM curve verification based on OS-based lasso regression. **(B)** Curve graph of risk scores of all samples based on OS. **(C)** Scatter plot of survival time of all samples based on OS. **(D)** Build model KM based on lasso regression of DFI Curve verification. **(E)** Curve graph of risk scores of all samples based on DFI. **(F)** Scatter plot of survival time of all samples based on DFI. **(G)** Time-based ROC curve. **(H)** Sankey diagram of clinical traits, risk grouping and alternative splicing subtype.

According to the risk score of the samples, the high and low risk groups were divided by the median as the node, and a Kaplan–Meier curve was drawn on the basis of the OS data and DFI data. The results showed that the difference between the high and low risk groups was significant (OS, p < 0.0001; DFI, p = 0.0083; Figures 8A–F). The sample risk score was used as the model prediction result, combined with the survival data to calculate the AUC value of the model, and then an ROC curve was drawn. The AUC values of 1-, 3-, and 5-year OS were all greater than 0.7, indicating that the model has good performance (Figure 8G). A Sankey diagram was constructed to show the relationship between risk score grouping, AS subtypes, and stage and grade groupings. As shown in Figure 8H, most of the cluster 1 samples belonged to the low-risk group, most of the cluster 1 samples were G1 and stage I samples, and most of the cluster 4 samples belonged to the high-risk group, consistent with the results of the Kaplan-Meier analysis.

## Discussion/conclusion

HCC is the most common primary liver cancer. Liver cancer is the sixth most common cancer and the second leading cause of cancer-related deaths worldwide. In the past few decades, the incidence of liver cancer and liver cancer-related deaths has increased in many parts of the world, including China (Siegel et al., 2021). Sorafenib remains the only targeted drug for the treatment of advanced liver cancer. As a chemotherapy-resistant tumor, HCC has an unsatisfactory response to radiotherapy and chemotherapy. In addition, patients with advanced liver cancer usually have obvious underlying liver disease, and thus the prognosis of patients is often poor and the mechanism is not understood.

Alternative splicing can be regulated by many different mechanisms, such as histone modification and DNA methylation, which are usually associated with specific SFs or transcriptional elongation (Li et al., 2018; Sessa et al., 2019). The latest research identified a significant correlation between intragenic DNA methylation and exon usage in solid tumors (Sun et al., 2020). There are also reports that mitochondria are involved in the regulation of transcriptional activity, which has a great influence on splicing regulation (Guantes et al., 2015). Alternative splicing is the main mechanism to increase the transcriptional diversity of eukaryotes (Pan et al., 2008; Wang et al., 2008). Dozens of abnormal splice variants are associated with human diseases (Schrock et al., 2016; Urbanski et al., 2018; Di et al., 2019). Studies have shown that these AS events play wide-ranging roles in the process of carcinogenesis, participating in cell proliferation, apoptosis, epithelial–mesenchymal transition, hypoxia, angiogenesis, and immune escape (Chen et al., 2018; Du et al., 2022). These previous studies have shown that in addition to classic cis-/trans-acting regulation, there are many other mechanisms of AS regulation (Yae et al., 2012; Picard, 2022; Zhang et al., 2022). Here, we use GSVA to conduct a comprehensive pathway analysis of 32,284 gene sets in MSigDB. We demonstrated that splicing regulation is also affected by many pathways, including negative/positive regulation of mRNA splicing by spliceosomes, pre-mRNA 5′-splice site binding, and the mRNA splicing minor pathway, among others. These pathways may constitute the basic environment for irregular splicing in HCC subtypes and affect the TME. We also observed changes in the mRNA expression of several SFs in different HCC subtypes. To determine how these changes are related to pathway activation and AS regulation, more research is required.

Future research aims to determine the molecular drivers of the transition from cluster 1 to cluster 4 subtypes. These changes may be triggered by changes in the genome or by epigenetic or transcriptional regulators that have been shown to drive splicing factor changes in other tumor types. Understanding these mechanisms will allow us to determine the development of AS-based HCC treatments. AS research is moving in the direction of making full use of the potential of AS in precision medicine.

In this analysis, we identified AS subtypes through unsupervised clustering, analyzed the characteristics of different spliced subtypes, obtained DASEs, and combined the findings with GSVA enrichment analysis to explore the differences in the subtypes. Finally, on the basis of the DASEs of different subtypes, the survival-related AS time was identified, and the PSI value was used to construct a risk proportional regression model to guide prognosis. We systematically described clinical, splicing, transcriptomic, genomic, and immunological characteristics, and identified the underlying regulatory mechanisms of AS in HCC subtypes (Supplementary Figure S2).

Our research shows that the splicing regulation of SFs may play a role in the transformation and survival of HCC cancer cells. We studied AS comprehensively and systematically and used TCGA data to explore possible non-classical regulatory mechanisms in HCC. The data sample size of our research was sufficient, and the results of the verification data are good and have strong statistical significance, covering a wide range of fields. Our findings may provide the foundation for more in-depth research in the future, such as studies of the splicing regulation mechanism, cancer biomarker design, targeted drug screening, and other clinical applications.

## Data Availability

The original contributions presented in the study are included in the article/Supplementary Material, further inquiries can be directed to the corresponding author.

## Supplementary material

Supplementary Figure S1 Significant degree of similarity between alternative splicing subtypes and various responses.

Supplementary Figure S2 Flowchart of this study.
