# Supplementary figures and images for: Integrating machine learning to construct aberrant alternative splicing event related classifiers to predict prognosis and immunotherapy response in patients with hepatocellular carcinoma
Source: Front Pharmacol. 2022 Oct 3;13:1019988. doi: 10.3389/fphar.2022.1019988 (PMC9573973; doi:10.3389/fphar.2022.1019988)

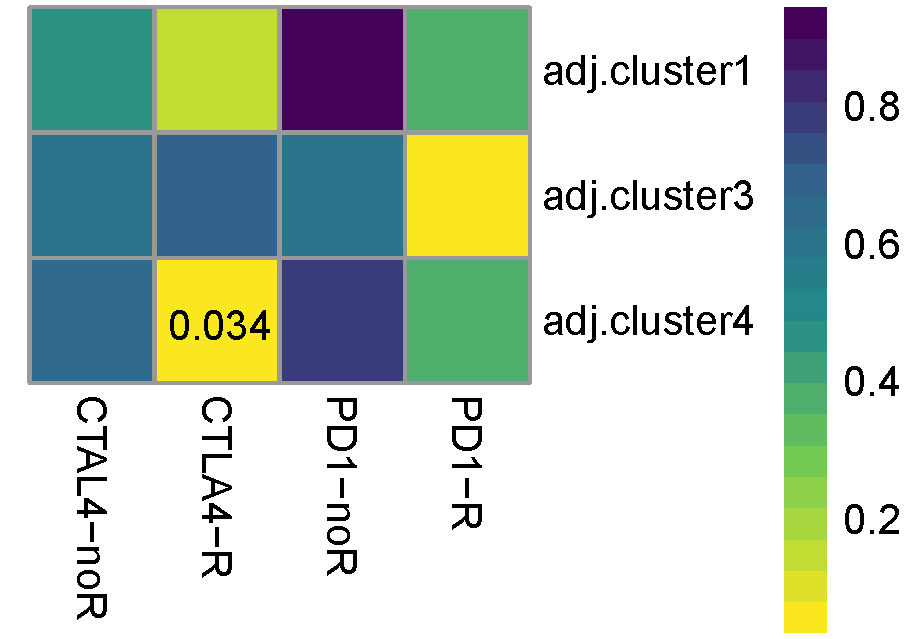

Supplement: Supplementary file 1 [file Image1.JPEG]

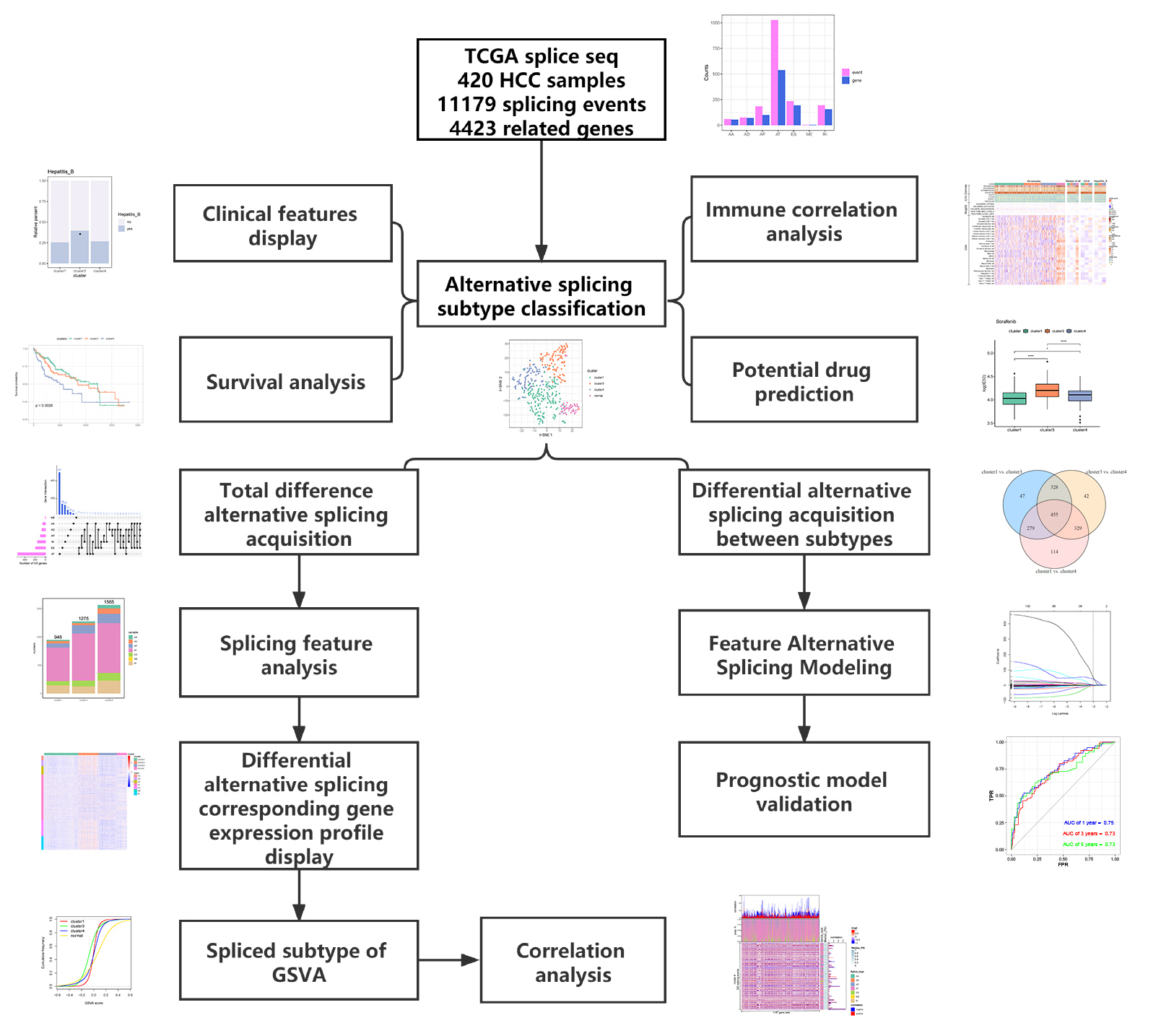

Supplement: Supplementary file 2 [file Image2.JPEG]
