# Supplementary material for: Integrating machine learning to construct aberrant alternative splicing event related classifiers to predict prognosis and immunotherapy response in patients with hepatocellular carcinoma
Source: Front Pharmacol. 2022 Oct 3;13:1019988. doi: 10.3389/fphar.2022.1019988 (PMC9573973; doi:10.3389/fphar.2022.1019988)
Supplement: Supplementary file 3 [file DataSheet1.PDF]

Table S1 Phenotypic information of TCGA-LIHC sample

		TCGA-Tumor (370)	TCGA-Normal (50)
age			
	>65	138	29
	<=65	232	21
gender			
	MALE	249	28
	FEMALE	121	22
grade			
	G1	55	5
	G2	177	27
	G3	121	15
	G4	12	0
	NA	5	3
pathologic_T			
	T1	181	20
	T2	93	14
	T3	80	13
	T4	13	3
	TX	1	0
	[Discrepancy]	1	0
	NA	1	0
stage			
	Stage I	171	18
	Stage II	85	11
	Stage III	85	12
	Stage IV	5	1
	[Discrepancy]	2	0
	NA	22	8
Alcohol_consumption			
	yes	117	9
	no	234	38
	unknown	19	3
Hepatitis_B			
	yes	104	7
	no	247	40
	unknown	19	3
Hepatitis_C			
	yes	56	5
	no	295	42
	unknown	19	3
OS			
	Alive	240	16
	Dead	130	34

as_id	wt_p	chi	chi_p	wt_adj.p	up_or_dow	symbol	splice_type	exons
41	1.42E-10	24.72324	6.62E-07	1.54E-06	up	SDF4	AA	6.1
165	2.56E-06	19.09297	1.25E-05	0.027883	down	MRPL20	AT	4
166	2.56E-06	19.09297	1.25E-05	0.027883	up	MRPL20	AT	3.2
179	9.25E-09	31.482	2.01E-08	0.000101	down	SSU72	AT	2.2
180	9.25E-09	30.68198	3.04E-08	0.000101	up	SSU72	AT	5
246	7.95E-07	16.55027	4.74E-05	0.00866	up	C1orf86	AT	7.2
247	7.95E-07	16.55027	4.74E-05	0.00866	down	C1orf86	AT	10
260	4.64E-08	24.72324	6.62E-07	0.000505	up	RER1	AA	6.1
289	3.23E-07	21.8173	3.00E-06	0.003514	up	TNFRSF14	AA	7.1
388	8.76E-13	34.53081	4.20E-09	9.53E-09	down	ACOT7	AP	3
391	1.79E-15	41.9773	9.23E-11	1.95E-11	up	ACOT7	AP	2
400	8.53E-08	16.26003	5.52E-05	0.000929	up	HES2	AT	4.3
401	8.53E-08	16.84383	4.06E-05	0.000929	down	HES2	AT	5
502	1.19E-09	34.53081	4.20E-09	1.29E-05	down	CAMTA1	AT	5
503	1.21E-07	27.81081	1.34E-07	0.001321	up	CAMTA1	AT	6
509	2.34E-06	12.00973	0.000529	0.025456	down	VAMP3	AP	1
510	2.34E-06	12.00973	0.000529	0.025456	up	VAMP3	AP	2
523	6.67E-09	23.66939	1.14E-06	7.27E-05	up	PARK7	AP	1.1
524	6.70E-09	25.81036	3.77E-07	7.29E-05	down	PARK7	AP	2.1
593	4.27E-06	21.8173	3.00E-06	0.04652	down	UBE4B	AT	3
594	4.27E-06	21.8173	3.00E-06	0.04652	up	UBE4B	AT	29
601	1.04E-06	12.00973	0.000529	0.011369	down	KIF1B	AT	25
602	1.04E-06	12.00973	0.000529	0.011369	up	KIF1B	AT	52.2
621	2.47E-08	27.81081	1.34E-07	0.000269	up	CASZ1	AT	16.2
622	2.47E-08	27.81081	1.34E-07	0.000269	down	CASZ1	AT	21
686	7.49E-10	24.72324	6.62E-07	8.15E-06	up	CLCN6	AT	24
687	7.68E-07	8.402559	0.003747	0.008357	down	CLCN6	AT	23.2
764	1.31E-09	27.81081	1.34E-07	1.43E-05	up	DNAJC16	AT	16
772	2.98E-09	31.08	2.48E-08	3.25E-05	up	FBLIM1	AT	11
773	2.98E-09	31.08	2.48E-08	3.25E-05	down	FBLIM1	AT	9.2
996	1.29E-08	24.36833	7.96E-07	0.000141	up	USP48	AT	27
997	1.29E-08	25.08186	5.49E-07	0.000141	down	USP48	AT	11.2
1072	3.96E-14	41.51466	1.17E-10	4.31E-10	up	LYPLA2	RI	8.5
1192	4.50E-12	31.08	2.48E-08	4.90E-08	up	MAN1C1	AP	4
1193	4.50E-12	31.08	2.48E-08	4.90E-08	down	MAN1C1	AP	1
1196	2.72E-07	12.00973	0.000529	0.002961	up	MTFR1L	AP	4.1
1272	2.17E-06	19.09297	1.25E-05	0.023583	up	DHDDS	AT	6.2
1273	1.36E-14	45.97297	1.20E-11	1.48E-10	down	DHDDS	AT	10.2
1274	1.10E-10	24.72324	6.62E-07	1.20E-06	up	DHDDS	AT	7
1591	5.30E-18	45.97297	1.20E-11	5.77E-14	up	BSDC1	RI	11.2
1612	7.17E-09	24.72324	6.62E-07	7.81E-05	up	ZBTB8OS	ES	5
1656	5.66E-07	19.09297	1.25E-05	0.006167	down	AK2	ES	2
1687	1.09E-16	41.9773	9.23E-11	1.19E-12	down	CSMD2	AT	50
1689	2.91E-16	41.9773	9.23E-11	3.17E-12	up	CSMD2	AT	78
1710	5.08E-07	21.8173	3.00E-06	0.005527	up	ZMYM6	AT	18
1712	6.34E-07	21.8173	3.00E-06	0.006902	down	ZMYM6	AT	20
1730	5.16E-12	23.66939	1.14E-06	5.62E-08	up	CLSPN	AT	25
1731	5.16E-12	25.08186	5.49E-07	5.62E-08	down	CLSPN	AT	26
1752	1.24E-06	13.92052	0.000191	0.013539	up	TRAPPC3	ES	4
1848	3.88E-07	21.48394	3.57E-06	0.004221	up	INPP5B	AT	7.3
1849	3.86E-07	19.40821	1.06E-05	0.004207	down	INPP5B	AT	24
1877	1.36E-13	34.7501	3.75E-09	1.48E-09	up	MACF1	AT	109
1879	4.05E-15	43.31349	4.66E-11	4.41E-11	down	MACF1	AT	4
1975	1.19E-09	38.16324	6.51E-10	1.30E-05	down	MYCL	AT	2
1976	1.19E-09	38.16324	6.51E-10	1.30E-05	up	MYCL	AT	1.4
2008	3.42E-15	45.97297	1.20E-11	3.72E-11	up	ZNF684	AT	5.2
2009	3.42E-15	45.97297	1.20E-11	3.72E-11	down	ZNF684	AT	6

2181	4.31E-11	31.08	2.48E-08	4.69E-07	up	HYI	AT	8.3
2182	4.29E-11	31.08	2.48E-08	4.67E-07	down	HYI	AT	9
2509	1.11E-09	30.68198	3.04E-08	1.21E-05	up	ATP6V0B	RI	7.2
2559	3.55E-10	27.81081	1.34E-07	3.86E-06	up	RNF220	AD	9.2
2561	3.70E-07	24.72324	6.62E-07	0.004032	up	TMEM53	AT	5
2562	3.70E-07	24.72324	6.62E-07	0.004032	down	TMEM53	AT	3.3
2577	3.36E-06	9.707363	0.001835	0.036577	down	RPS8	AD	3.2
2694	2.30E-06	16.26003	5.52E-05	0.025027	up	PRDX1	AD	1.2
2698	1.36E-11	41.9773	9.23E-11	1.48E-07	up	AKR1A1	ES	2
2706	2.51E-15	50.15027	1.42E-12	2.73E-11	up	NASP	ES	9
3019	1.81E-17	50.15027	1.42E-12	1.97E-13	up	ECHDC2	AT	8.2
3020	6.70E-08	38.16324	6.51E-10	0.00073	up	ECHDC2	AT	5.2
3021	1.62E-15	50.15027	1.42E-12	1.76E-11	up	ECHDC2	AT	9.2
3022	1.36E-15	50.15027	1.42E-12	1.48E-11	down	ECHDC2	AT	14
3024	8.65E-17	50.15027	1.42E-12	9.41E-13	down	ECHDC2	ES	9.1
3043	7.69E-13	45.97297	1.20E-11	8.37E-09	down	SCP2	ES	15
3045	7.08E-20	50.15027	1.42E-12	7.71E-16	up	SCP2	ES	12
3074	2.97E-08	28.19112	1.10E-07	0.000323	down	YIPF1	ES	12
3098	3.10E-06	19.09297	1.25E-05	0.033805	up	HSPB11	AT	5.2
3121	7.96E-11	34.53081	4.20E-09	8.67E-07	down	TMEM59	AD	1.2
3137	3.02E-08	19.09297	1.25E-05	0.000328	up	MRPL37	AT	8
3138	3.02E-08	19.09297	1.25E-05	0.000328	down	MRPL37	AT	7
3239	6.78E-07	21.8173	3.00E-06	0.007383	down	INADL	AT	47.3
3295	1.05E-11	41.9773	9.23E-11	1.14E-07	down	LEPR	AT	22
3297	3.99E-12	41.9773	9.23E-11	4.35E-08	up	LEPR	AT	21
3298	7.13E-10	36.25104	1.73E-09	7.77E-06	down	LEPR	AT	23
3346	5.27E-24	50.15027	1.42E-12	5.74E-20	up	SLC35D1	AT	12
3347	5.28E-24	50.15027	1.42E-12	5.74E-20	down	SLC35D1	AT	13
3354	7.11E-08	21.8173	3.00E-06	0.000774	down	SERBP1	AA	5.1
3514	2.38E-11	34.53081	4.20E-09	2.59E-07	up	RABGGTB	ES	5:06
3810	2.22E-20	49.64457	1.84E-12	2.42E-16	up	ABCD3	AT	10
3811	2.21E-20	51.17628	8.44E-13	2.40E-16	down	ABCD3	AT	25
4052	4.03E-11	34.53081	4.20E-09	4.39E-07	up	GSTM4	RI	5.2
4057	4.31E-10	30.68198	3.04E-08	4.69E-06	up	GSTM2	AT	13
4061	2.25E-10	34.53081	4.20E-09	2.45E-06	down	GSTM2	AT	9
4174	8.85E-20	50.15027	1.42E-12	9.64E-16	up	ADORA3	AT	3
4175	8.85E-20	50.15027	1.42E-12	9.64E-16	down	ADORA3	AT	8
4232	3.03E-08	22.49476	2.11E-06	0.00033	down	RHOC	ES	4
4377	6.57E-09	24.72324	6.62E-07	7.15E-05	down	GDAP2	AT	14
4378	6.57E-09	24.72324	6.62E-07	7.15E-05	up	GDAP2	AT	13.2
4410	1.26E-15	45.97297	1.20E-11	1.38E-11	down	PDE4DIP	AT	11.2
4412	6.04E-08	19.09297	1.25E-05	0.000658	up	PDE4DIP	AT	30.3
7278	7.87E-07	21.8173	3.00E-06	0.008568	down	POLR3GL	ES	4
7367	7.80E-21	45.4888	1.54E-11	8.49E-17	up	FMO5	AT	11
7486	8.34E-12	31.08	2.48E-08	9.08E-08	up	ADAMTSL4	AT	16.2
7487	8.34E-12	31.08	2.48E-08	9.08E-08	down	ADAMTSL4	AT	20
7490	1.53E-21	46.4618	9.34E-12	1.66E-17	down	ENSA	AP	1
7491	1.53E-21	49.14366	2.38E-12	1.66E-17	up	ENSA	AP	2
7527	9.94E-12	33.69577	6.44E-09	1.08E-07	up	CERS2	AT	12
7528	9.99E-12	35.38241	2.71E-09	1.09E-07	down	CERS2	AT	11
7563	1.65E-12	34.53081	4.20E-09	1.80E-08	down	SCNM1	AP	1
7564	1.65E-12	34.53081	4.20E-09	1.80E-08	up	SCNM1	AP	2.1
7662	3.14E-07	12.00973	0.000529	0.003423	up	TDRKH	AT	15.3
7776	6.59E-15	34.11125	5.20E-09	7.18E-11	up	JTB	RI	2.2
7792	1.26E-06	14.18919	0.000165	0.013675	down	TPM3	ES	12
7798	1.42E-11	41.51466	1.17E-10	1.55E-07	up	C1orf43	AT	6.2
7799	1.42E-11	43.87327	3.50E-11	1.55E-07	down	C1orf43	AT	7
7800	1.83E-10	41.9773	9.23E-11	1.99E-06	down	C1orf43	ES	4

7813	2.34E-09	14.18919	0.000165	2.55E-05	up	UBAP2L	AT	26
7814	2.20E-10	21.8173	3.00E-06	2.40E-06	down	UBAP2L	AT	29
7863	2.79E-14	34.53081	4.20E-09	3.04E-10	up	FLAD1	AT	2.3
7864	2.54E-06	16.55027	4.74E-05	0.027638	down	FLAD1	AT	4.4
7866	6.85E-11	18.47277	1.72E-05	7.46E-07	up	FLAD1	RI	6.2
7867	9.84E-09	12.00973	0.000529	0.000107	up	FLAD1	RI	4.2
7903	6.67E-11	27.81081	1.34E-07	7.27E-07	down	ADAM15	ES	21.1:21.2:2
7904	4.00E-08	31.08	2.48E-08	0.000436	down	ADAM15	ES	21.2:22.1
7944	1.57E-10	24.72324	6.62E-07	1.71E-06	up	SLC50A1	ES	4
7945	7.78E-20	41.51466	1.17E-10	8.47E-16	up	DPM3	AP	2.1
7946	7.78E-20	42.44441	7.27E-11	8.47E-16	down	DPM3	AP	1
8038	8.38E-09	25.81036	3.77E-07	9.13E-05	down	MTX1	ES	4
8168	3.54E-07	15.97308	6.42E-05	0.003856	up	LAMTOR2	AA	4.1
8221	8.47E-07	14.18919	0.000165	0.009228	up	C1orf85	AT	6.3
8222	8.47E-07	14.18919	0.000165	0.009228	down	C1orf85	AT	7
8409	4.75E-11	34.53081	4.20E-09	5.17E-07	down	DUSP23	AP	2.1
8410	4.75E-11	34.53081	4.20E-09	5.17E-07	up	DUSP23	AP	1.1
8426	9.06E-13	38.16324	6.51E-10	9.86E-09	down	IGSF8	AA	7.1
8439	5.51E-09	31.08	2.48E-08	6.00E-05	up	DCAF8	AT	10.2
8440	5.51E-09	31.08	2.48E-08	6.00E-05	down	DCAF8	AT	21
8555	7.18E-09	45.97297	1.20E-11	7.81E-05	up	NIT1	AT	8
8556	7.18E-09	45.97297	1.20E-11	7.81E-05	down	NIT1	AT	7
8597	3.32E-11	27.43434	1.63E-07	3.62E-07	up	NDUFS2	RI	14.2
8599	2.06E-10	28.96344	7.38E-08	2.25E-06	down	FCER1G	AT	5
8600	2.06E-10	30.68198	3.04E-08	2.25E-06	up	FCER1G	AT	6
8750	1.77E-08	45.97297	1.20E-11	0.000193	down	UAP1	ES	9.1:9.2
8799	1.12E-06	22.15422	2.52E-06	0.012165	down	MGST3	AT	10
8800	1.12E-06	26.32744	2.88E-07	0.012165	up	MGST3	AT	9
8877	2.52E-09	27.06166	1.97E-07	2.74E-05	up	MPC2	AP	2.1
8878	2.53E-09	26.55426	2.56E-07	2.75E-05	down	MPC2	AP	1
8966	2.76E-07	13.65504	0.00022	0.003002	up	GORAB	AT	4.2
8967	2.76E-07	14.46107	0.000143	0.003002	down	GORAB	AT	5
9096	6.80E-10	25.44421	4.55E-07	7.41E-06	down	TOR3A	AT	7
9097	6.80E-10	24.36833	7.96E-07	7.41E-06	up	TOR3A	AT	6
9239	4.12E-09	24.72324	6.62E-07	4.49E-05	up	UCHL5	AT	14
9240	1.16E-09	21.8173	3.00E-06	1.26E-05	down	UCHL5	AT	15
9577	2.07E-23	50.15027	1.42E-12	2.25E-19	down	FAM72A	AT	2
9616	2.04E-13	38.16324	6.51E-10	2.22E-09	down	PFKFB2	AT	18
9617	2.04E-13	38.16324	6.51E-10	2.22E-09	up	PFKFB2	AT	17
9717	1.41E-21	41.9773	9.23E-11	1.53E-17	up	NEK2	AT	8
9718	1.41E-21	41.9773	9.23E-11	1.53E-17	down	NEK2	AT	7.2
10057	3.51E-06	21.8173	3.00E-06	0.038202	down	SNAP47	AT	7
10058	3.51E-06	21.8173	3.00E-06	0.038202	up	SNAP47	AT	6
10178	1.96E-07	14.18919	0.000165	0.002138	down	GUK1	AP	2
10181	8.22E-08	31.08	2.48E-08	0.000895	up	GUK1	AP	4
10185	1.08E-08	27.81081	1.34E-07	0.000118	down	GUK1	ES	11.1:11.2
10188	1.10E-06	10.01189	0.001555	0.01199	down	GUK1	AA	7.1
10189	2.75E-09	24.01706	9.55E-07	3.00E-05	up	GUK1	AD	5.4
10261	1.21E-10	27.43434	1.63E-07	1.32E-06	up	ARV1	AT	6
10262	1.21E-10	28.57531	9.01E-08	1.32E-06	down	ARV1	AT	7
10316	1.88E-13	41.9773	9.23E-11	2.05E-09	up	NTPCR	AT	5
10317	1.88E-13	41.9773	9.23E-11	2.05E-09	down	NTPCR	AT	6
10335	1.21E-18	45.97297	1.20E-11	1.31E-14	up	COA6	AP	1
10336	1.21E-18	45.97297	1.20E-11	1.31E-14	down	COA6	AP	2.1
10475	1.27E-13	45.97297	1.20E-11	1.38E-09	up	COX20	ES	3
10479	9.65E-13	31.08	2.48E-08	1.05E-08	up	EFCAB2	AT	8
10480	9.65E-13	31.08	2.48E-08	1.05E-08	down	EFCAB2	AT	9
10516	5.16E-13	38.16324	6.51E-10	5.62E-09	up	ZNF124	AT	6

10596	5.40E-12	31.08	2.48E-08	5.88E-08	up	DIP2C	AT	28.2
10597	5.40E-12	31.08	2.48E-08	5.88E-08	down	DIP2C	AT	41
10750	3.23E-06	19.09297	1.25E-05	0.035214	up	USP6NL	AT	4
10751	3.23E-06	19.09297	1.25E-05	0.035214	down	USP6NL	AT	18
10760	1.09E-13	31.08	2.48E-08	1.19E-09	down	SEC61A2	AT	14
10761	1.42E-15	38.16324	6.51E-10	1.54E-11	up	SEC61A2	AT	16
10766	1.45E-06	16.55027	4.74E-05	0.015788	up	NUDT5	AT	10
10767	2.10E-08	19.09297	1.25E-05	0.000228	up	NUDT5	AT	9.5
10768	7.72E-09	24.72324	6.62E-07	8.41E-05	down	NUDT5	AT	11
10837	2.83E-06	19.09297	1.25E-05	0.03083	down	DCLRE1C	AT	18
10838	2.83E-06	19.09297	1.25E-05	0.03083	up	DCLRE1C	AT	17
11368	1.88E-11	31.08	2.48E-08	2.04E-07	up	ZFAND4	AT	6
12029	6.69E-13	38.16324	6.51E-10	7.28E-09	down	AIFM2	AT	10
12030	6.69E-13	38.16324	6.51E-10	7.28E-09	up	AIFM2	AT	9.2
12058	3.24E-09	41.9773	9.23E-11	3.53E-05	up	CDH23	AT	15.2
12059	3.64E-09	24.72324	6.62E-07	3.96E-05	up	CDH23	AT	27.2
12060	1.30E-19	50.15027	1.42E-12	1.42E-15	down	CDH23	AT	72
12090	5.58E-09	24.72324	6.62E-07	6.07E-05	up	DNAJB12	RI	9.3
12153	6.99E-15	45.97297	1.20E-11	7.61E-11	down	PPP3CB	AT	17
12154	6.99E-15	45.97297	1.20E-11	7.61E-11	up	PPP3CB	AT	15.2
12230	1.22E-09	27.81081	1.34E-07	1.33E-05	up	CHCHD1	RI	2.2
12295	1.04E-10	45.97297	1.20E-11	1.13E-06	down	RPS24	ES	5.1:5.2
12509	5.55E-10	31.08	2.48E-08	6.05E-06	up	PCGF5	AT	4
12510	5.55E-10	31.08	2.48E-08	6.05E-06	down	PCGF5	AT	12
12516	6.50E-08	19.40821	1.06E-05	0.000708	down	HECTD2	AT	21
12517	6.50E-08	18.78117	1.47E-05	0.000708	up	HECTD2	AT	5.2
12567	7.88E-10	31.08	2.48E-08	8.58E-06	up	SLC35G1	AT	5.2
12570	3.60E-09	34.53081	4.20E-09	3.92E-05	down	SLC35G1	AT	3
12730	1.30E-09	27.81081	1.34E-07	1.42E-05	up	MORN4	AT	2.2
12731	1.30E-09	27.81081	1.34E-07	1.42E-05	down	MORN4	AT	5
12760	2.79E-14	34.53081	4.20E-09	3.04E-10	up	HPS1	AT	10.2
12761	2.79E-14	34.53081	4.20E-09	3.04E-10	down	HPS1	AT	20
12824	2.67E-11	34.95451	3.37E-09	2.90E-07	down	NDUFB8	AT	6
12825	4.73E-12	34.11125	5.20E-09	5.15E-08	up	NDUFB8	AT	5.2
12839	4.96E-08	19.09297	1.25E-05	0.00054	up	FAM178A	AT	1.2
12841	1.02E-08	24.72324	6.62E-07	0.000112	down	FAM178A	AT	6.2
12955	1.32E-06	19.09297	1.25E-05	0.014417	down	TMEM180	ES	4
13044	8.33E-15	50.15027	1.42E-12	9.07E-11	down	GSTO1	AP	2
13045	8.33E-15	50.15027	1.42E-12	9.07E-11	up	GSTO1	AP	1
13089	1.91E-06	23.66939	1.14E-06	0.020751	up	BBIP1	ES	6
13115	1.86E-12	24.72324	6.62E-07	2.02E-08	up	VTI1A	AT	9
13116	1.86E-12	24.72324	6.62E-07	2.02E-08	down	VTI1A	AT	8
13400	5.10E-08	24.72324	6.62E-07	0.000555	down	LHPP	AT	8
13401	5.08E-08	24.72324	6.62E-07	0.000553	up	LHPP	AT	6
13429	3.68E-06	21.8173	3.00E-06	0.040055	down	UROS	AT	11
13431	2.16E-12	45.97297	1.20E-11	2.35E-08	down	BCCIP	AT	7
13477	2.72E-10	40.43456	2.03E-10	2.96E-06	down	BNIP3	AT	6
13478	2.72E-10	37.72214	8.16E-10	2.96E-06	up	BNIP3	AT	5.2
13641	2.02E-06	14.18919	0.000165	0.022043	up	IFITM2	AP	1
13642	2.02E-06	14.18919	0.000165	0.022043	down	IFITM2	AP	2.1
13652	1.81E-09	27.81081	1.34E-07	1.97E-05	up	SIGIRR	RI	10.2
13690	3.78E-07	16.55027	4.74E-05	0.004112	up	RASSF7	RI	6.2:6.3:6.4
13694	5.94E-09	27.81081	1.34E-07	6.47E-05	up	RASSF7	RI	6.2
13745	1.59E-06	243.8095	5.81E-55	0.017281	down	PDDC1	ES	7:8.1:8.2
13918	4.09E-13	38.16324	6.51E-10	4.45E-09	down	CD81	AP	3
13994	3.22E-14	34.53081	4.20E-09	3.51E-10	up	NUP98	AT	35
13995	3.22E-14	34.53081	4.20E-09	3.51E-10	down	NUP98	AT	20.2
14252	3.08E-08	12.00973	0.000529	0.000335	up	RPL27A	AT	3.5

14253	3.09E-08	12.00973	0.000529	0.000337	down	RPL27A	AT	4
14255	1.88E-09	43.36118	4.55E-11	2.05E-05	down	RPL27A	AA	3.3
14355	3.70E-07	13.92052	0.000191	0.004024	up	RNF141	AT	6.2
14356	3.70E-07	15.29641	9.19E-05	0.004024	down	RNF141	AT	7
14480	4.27E-06	12.25994	0.000463	0.046457	down	CYP2R1	AA	6.1
14577	1.62E-06	12.00973	0.000529	0.017656	up	SAA2	AT	5
14580	6.18E-07	16.84383	4.06E-05	0.006728	down	SAA2	AT	4
14634	5.22E-07	13.92052	0.000191	0.005679	up	LDHA	ES	3:04
14636	9.33E-12	31.08	2.48E-08	1.02E-07	up	LDHA	ES	3
14793	1.56E-08	31.08	2.48E-08	0.00017	up	MPPED2	AT	9
14794	1.56E-08	31.08	2.48E-08	0.00017	down	MPPED2	AT	10
14906	1.34E-06	12.00973	0.000529	0.01454	up	CD59	AT	10
14907	1.34E-06	12.00973	0.000529	0.014585	down	CD59	AT	8.3
14929	1.13E-06	14.18919	0.000165	0.012336	up	FBXO3	RI	12.2:12.3
15679	6.74E-09	33.13096	8.62E-09	7.34E-05	down	ACP2	AT	12
15681	5.63E-09	30.2879	3.72E-08	6.13E-05	up	ACP2	AT	7
15699	4.13E-11	34.53081	4.20E-09	4.50E-07	up	NR1H3	RI	8.2
15738	3.33E-07	19.09297	1.25E-05	0.003628	down	SLC39A13	RI	6.12
15780	6.24E-07	18.85715	1.41E-05	0.006793	up	NDUFS3	AT	2.2
15785	3.14E-11	30.68198	3.04E-08	3.42E-07	up	NDUFS3	RI	3.2
15805	7.46E-07	19.09297	1.25E-05	0.008125	up	NUP160	AT	3.4
15806	3.09E-07	21.8173	3.00E-06	0.003367	down	NUP160	AT	34.2
15924	4.01E-13	28.19112	1.10E-07	4.36E-09	down	C11orf31	AT	4
15925	3.92E-13	27.43434	1.63E-07	4.26E-09	up	C11orf31	AT	3.2
15932	2.07E-06	22.15422	2.52E-06	0.022521	down	CTNND1	RI	22.3
16288	2.56E-08	20.82773	5.03E-06	0.000279	up	TMEM258	AD	1.2
16295	3.31E-07	21.8173	3.00E-06	0.003605	down	FADS2	AT	17
16332	2.88E-17	52.26344	4.85E-13	3.14E-13	down	FTH1	AA	4.1
16347	1.01E-09	34.53081	4.20E-09	1.10E-05	down	AHNAK	AT	7
16348	1.01E-09	34.53081	4.20E-09	1.10E-05	up	AHNAK	AT	5
16387	4.39E-07	13.92052	0.000191	0.004784	up	C11orf48	RI	5.2
16396	1.72E-11	24.72324	6.62E-07	1.88E-07	up	UBXN1	RI	8.2
16427	1.11E-06	8.402559	0.003747	0.012125	down	TMEM179I	ES	4
16428	2.12E-07	22.15422	2.52E-06	0.002307	down	TMEM223	AT	2
16429	2.12E-07	24.36833	7.96E-07	0.002307	up	TMEM223	AT	3
16462	5.98E-15	41.9773	9.23E-11	6.51E-11	up	SLC3A2	AP	1
16463	8.63E-16	38.16324	6.51E-10	9.39E-12	up	SLC3A2	AP	6.1
16516	3.37E-18	49.64457	1.84E-12	3.67E-14	up	PLA2G16	AP	1
16517	3.37E-18	50.66081	1.10E-12	3.67E-14	down	PLA2G16	AP	2.1
16556	9.81E-07	16.55027	4.74E-05	0.010678	down	OTUB1	AT	8.4
16558	9.81E-07	16.55027	4.74E-05	0.010678	up	OTUB1	AT	10
16602	7.65E-07	16.55027	4.74E-05	0.008335	up	FKBP2	AP	3
16616	9.42E-18	45.97297	1.20E-11	1.03E-13	down	BAD	RI	1.2
16622	5.27E-09	19.09297	1.25E-05	5.74E-05	down	GPR137	AA	10.1
16623	5.48E-07	19.40821	1.06E-05	0.005964	down	GPR137	ES	9
16624	6.35E-08	24.72324	6.62E-07	0.000691	down	GPR137	ES	7
16640	3.88E-11	31.08	2.48E-08	4.23E-07	down	PRDX5	ES	3
16762	3.42E-07	243.8095	5.81E-55	0.003726	down	TM7SF2	ES	4:05
16855	4.93E-11	31.08	2.48E-08	5.36E-07	up	SCYL1	RI	17.2
16857	7.94E-07	17.14076	3.47E-05	0.008643	down	SCYL1	ES	17.1
16916	8.74E-08	24.72324	6.62E-07	0.000952	up	RNASEH2(	RI	1.2
16938	5.40E-11	21.8173	3.00E-06	5.88E-07	up	FIBP	AD	5.2
16979	3.28E-07	16.26003	5.52E-05	0.003572	up	BANF1	AP	1.1
16980	3.28E-07	16.84383	4.06E-05	0.003572	down	BANF1	AP	2.1
17003	1.18E-08	19.09297	1.25E-05	0.000128	up	YIF1A	AP	8.1
17004	1.18E-08	20.04919	7.55E-06	0.000128	down	YIF1A	AP	1
17007	2.91E-14	26.69271	2.39E-07	3.17E-10	up	YIF1A	RI	8.3
17008	7.22E-13	42.44441	7.27E-11	7.86E-09	down	YIF1A	ES	7

17073	1.28E-15	40.153	2.35E-10	1.39E-11	up	CCS	AA	6.1
17095	1.47E-06	11.76263	0.000604	0.015967	up	RBM4	AT	6
17172	1.19E-10	34.53081	4.20E-09	1.29E-06	up	POLD4	AD	1.2
17184	4.24E-10	31.08	2.48E-08	4.62E-06	down	PPP1CA	ES	2.2
17200	3.53E-07	10.01189	0.001555	0.003848	up	RPS6KB2	AT	5.2
17259	2.43E-18	38.16324	6.51E-10	2.64E-14	up	NUDT8	RI	3.2
17279	1.16E-06	14.18919	0.000165	0.012684	up	NDUFS8	AD	4.2
17340	1.37E-07	30.68198	3.04E-08	0.001487	up	MRPL21	RI	6.2
17392	1.39E-09	14.18919	0.000165	1.52E-05	up	PPFIA1	AT	27.2
17393	1.39E-09	14.18919	0.000165	1.52E-05	down	PPFIA1	AT	29
17433	8.13E-08	19.09297	1.25E-05	0.000886	up	FAM86C1	AT	5.3
17434	8.13E-08	19.09297	1.25E-05	0.000886	down	FAM86C1	AT	6
17557	1.04E-08	16.55027	4.74E-05	0.000113	up	LAMTOR1	RI	4.2
17567	2.26E-07	16.55027	4.74E-05	0.002457	down	ANAPC15	AA	7.1
17737	3.13E-06	14.18919	0.000165	0.034062	up	COA4	ES	2.2
17760	6.85E-08	21.8173	3.00E-06	0.000745	up	C2CD3	AT	35
17761	9.46E-08	21.8173	3.00E-06	0.00103	down	C2CD3	AT	8
17902	4.88E-09	27.81081	1.34E-07	5.31E-05	up	TSKU	AP	2
17903	4.88E-09	27.81081	1.34E-07	5.31E-05	down	TSKU	AP	1
17959	1.96E-06	12.00973	0.000529	0.02136	up	CLNS1A	ES	5
17979	1.38E-06	12.25994	0.000463	0.015055	down	AAMDC	ES	6
17991	3.76E-06	8.195676	0.004199	0.040915	down	INTS4	AT	13.2
17992	1.95E-07	14.18919	0.000165	0.002124	up	INTS4	AT	26
18114	2.19E-09	21.48394	3.57E-06	2.39E-05	up	TMEM126I	RI	6.2
18185	5.29E-11	31.08	2.48E-08	5.76E-07	up	C11orf73	RI	5.2
18205	3.41E-06	14.18919	0.000165	0.037166	down	TMEM135	AT	16
18206	3.40E-06	14.18919	0.000165	0.037055	up	TMEM135	AT	6
18388	1.83E-06	19.09297	1.25E-05	0.019916	up	CEP57	AT	10.4
18390	1.83E-06	19.09297	1.25E-05	0.019977	down	CEP57	AT	13
18599	1.13E-16	41.77557	1.02E-10	1.23E-12	up	ACAT1	AT	10.2
18600	4.89E-13	42.91607	5.71E-11	5.32E-09	down	ACAT1	AT	13
18630	2.73E-12	31.482	2.01E-08	2.97E-08	down	RDX	AT	15.3
18632	7.21E-13	41.9773	9.23E-11	7.85E-09	up	RDX	AT	17
18709	5.32E-13	38.16324	6.51E-10	5.80E-09	up	DIXDC1	AT	7.2
18710	5.32E-13	38.16324	6.51E-10	5.80E-09	down	DIXDC1	AT	24
18732	7.75E-22	50.15027	1.42E-12	8.44E-18	up	SDHD	AT	5
18734	2.62E-09	27.43434	1.63E-07	2.85E-05	up	SDHD	AT	6
18764	2.00E-09	37.72214	8.16E-10	2.18E-05	up	PTS	ES	4
18823	8.65E-09	21.8173	3.00E-06	9.42E-05	up	RBM7	RI	4.3
18868	9.78E-14	50.15027	1.42E-12	1.07E-09	down	APOA1	ES	2
18926	1.12E-07	30.68198	3.04E-08	0.001221	up	FXYD6	AT	10.6
18928	1.12E-07	28.96344	7.38E-08	0.001216	down	FXYD6	AT	11
19307	3.15E-07	19.40821	1.06E-05	0.003431	down	CHEK1	AP	1
19309	3.15E-07	18.78117	1.47E-05	0.003431	up	CHEK1	AP	2.1
19311	8.03E-10	19.09297	1.25E-05	8.75E-06	up	CHEK1	AT	13.3
19312	8.03E-10	19.09297	1.25E-05	8.75E-06	down	CHEK1	AT	14
19546	6.50E-08	21.8173	3.00E-06	0.000708	down	THYN1	ES	6
19551	3.37E-07	16.26003	5.52E-05	0.003667	up	ACAD8	AT	11
19552	3.37E-07	16.84383	4.06E-05	0.003667	down	ACAD8	AT	12
19827	1.18E-12	34.53081	4.20E-09	1.29E-08	down	TNFRSF1A	AT	11
19829	1.19E-12	34.53081	4.20E-09	1.29E-08	up	TNFRSF1A	AT	6
19960	5.37E-10	26.94978	2.09E-07	5.85E-06	down	MLF2	AP	3
19961	5.20E-13	38.16324	6.51E-10	5.67E-09	up	MLF2	AP	1
19966	2.26E-06	19.09297	1.25E-05	0.024612	up	PTMS	AA	3.1
20018	3.11E-09	30.89721	2.72E-08	3.39E-05	up	C12orf57	AT	2.4
20019	3.46E-09	35.81457	2.17E-09	3.77E-05	down	C12orf57	AT	3
20020	1.41E-06	14.18919	0.000165	0.015306	down	C12orf57	AD	2.3
20046	3.16E-10	27.06166	1.97E-07	3.44E-06	up	PHB2	RI	5.2

20072	2.97E-26	50.66081	1.10E-12	3.23E-22	down	C1RL	AT	6
20645	7.25E-07	31.08	2.48E-08	0.007895	down	PLEKHA5	AT	4
20646	5.45E-07	16.55027	4.74E-05	0.005934	up	PLEKHA5	AT	36
20993	2.26E-11	27.81081	1.34E-07	2.46E-07	up	DENND5B	AT	12.2
20994	2.26E-11	27.81081	1.34E-07	2.46E-07	down	DENND5B	AT	25
21214	3.46E-12	38.60865	5.18E-10	3.76E-08	down	PPHLN1	AT	19
21297	2.29E-06	12.25994	0.000463	0.024912	down	ANO6	AT	24
21391	8.94E-07	19.09297	1.25E-05	0.009738	up	TMEM106(	AD	5.2
21495	2.40E-08	34.53081	4.20E-09	0.000262	up	FKBP11	AT	8
21496	1.24E-08	34.53081	4.20E-09	0.000135	down	FKBP11	AT	7.2
21577	9.92E-20	50.15027	1.42E-12	1.08E-15	down	SPATS2	AT	6
21578	9.92E-20	50.15027	1.42E-12	1.08E-15	up	SPATS2	AT	19
21652	9.00E-07	16.26003	5.52E-05	0.009795	up	COX14	AD	1.2
21700	2.88E-09	19.40821	1.06E-05	3.13E-05	down	LARP4	AT	17
21701	2.90E-09	18.47277	1.72E-05	3.16E-05	up	LARP4	AT	12.2
21729	4.61E-09	24.36833	7.96E-07	5.02E-05	up	SLC11A2	AT	23.3
21730	4.61E-09	25.08186	5.49E-07	5.02E-05	down	SLC11A2	AT	22.2
21809	3.09E-09	34.53081	4.20E-09	3.37E-05	up	DAZAP2	AT	6
21811	1.40E-08	38.60865	5.18E-10	0.000153	down	DAZAP2	AT	5
21884	2.31E-18	41.9773	9.23E-11	2.51E-14	down	NR4A1	AP	4.1
21887	3.12E-18	41.9773	9.23E-11	3.39E-14	up	NR4A1	AP	2
21907	5.75E-10	27.81081	1.34E-07	6.27E-06	down	KRT8	AP	3.1
21908	1.12E-09	27.81081	1.34E-07	1.22E-05	up	KRT8	AP	1.1
21911	1.97E-12	38.16324	6.51E-10	2.14E-08	up	KRT18	AP	1
21912	1.97E-12	38.16324	6.51E-10	2.14E-08	down	KRT18	AP	2.1
21917	4.77E-16	38.16324	6.51E-10	5.19E-12	up	EIF4B	AD	12.2
22028	1.79E-06	13.23145	0.000275	0.01945	down	PRR13	AT	5
22029	1.77E-06	14.31828	0.000154	0.019263	up	PRR13	AT	4.6
22046	2.29E-06	24.72324	6.62E-07	0.024979	up	PCBP2	AT	14.2
22047	2.29E-06	24.72324	6.62E-07	0.024979	down	PCBP2	AT	18
22052	1.35E-13	41.9773	9.23E-11	1.47E-09	up	PCBP2	ES	15
22055	2.08E-07	12.25994	0.000463	0.002262	down	PCBP2	ES	12:13
22056	1.69E-06	10.01189	0.001555	0.018437	down	PCBP2	ES	13
22072	5.82E-09	24.72324	6.62E-07	6.34E-05	down	TARBP2	ES	8
22093	7.73E-07	6.561081	0.010423	0.008415	up	ATF7	AT	14
22127	1.66E-07	21.8173	3.00E-06	0.001807	up	SMUG1	RI	4.2
22146	3.92E-09	30.68198	3.04E-08	4.27E-05	up	HNRNPA1	ES	8
22147	2.14E-07	27.81081	1.34E-07	0.002332	up	HNRNPA1	ES	6.2:7.1:7.2:8
22229	1.56E-13	34.53081	4.20E-09	1.70E-09	down	BLOC1S1	AP	1
22230	1.56E-13	34.53081	4.20E-09	1.70E-09	up	BLOC1S1	AP	2
22233	2.63E-14	25.60778	4.18E-07	2.86E-10	up	BLOC1S1	RI	4.2
22245	2.75E-06	12.00973	0.000529	0.029901	up	CD63	AP	3.1
22340	4.10E-13	38.16324	6.51E-10	4.46E-09	up	SUOX	ES	3
22365	2.76E-13	41.51466	1.17E-10	3.01E-09	up	RPL41	RI	1.2
22376	2.48E-06	14.18919	0.000165	0.026971	down	MYL6	AT	5
22689	8.22E-09	24.72324	6.62E-07	8.95E-05	down	OS9	AT	15
22692	8.35E-09	24.72324	6.62E-07	9.09E-05	up	OS9	AT	5.4
22723	2.43E-12	31.08	2.48E-08	2.65E-08	up	TSPAN31	AT	6.2
22724	2.43E-12	31.08	2.48E-08	2.65E-08	down	TSPAN31	AT	7
22934	2.13E-09	21.8173	3.00E-06	2.31E-05	down	RAP1B	AT	9
22935	9.28E-11	24.72324	6.62E-07	1.01E-06	up	RAP1B	AT	8.2
23408	7.00E-10	14.18919	0.000165	7.63E-06	up	THAP2	AT	2
23409	7.00E-10	14.18919	0.000165	7.63E-06	down	THAP2	AT	4
23727	1.97E-06	19.09297	1.25E-05	0.021442	down	CCDC41	AT	9.2
23728	1.97E-06	19.09297	1.25E-05	0.021442	up	CCDC41	AT	18
23741	9.35E-13	34.53081	4.20E-09	1.02E-08	up	NR2C1	AT	12.2
23742	9.35E-13	34.53081	4.20E-09	1.02E-08	down	NR2C1	AT	15
23824	2.10E-06	11.76263	0.000604	0.022853	up	LTA4H	AA	18.1

24017	2.48E-07	18.47277	1.72E-05	0.002698	up	CCDC53	ES	11
24050	2.74E-10	27.43434	1.63E-07	2.98E-06	up	IGF1	AT	5.2
24051	2.74E-10	28.19112	1.10E-07	2.98E-06	down	IGF1	AT	6
24140	1.39E-06	16.55027	4.74E-05	0.015164	down	TCP11L2	AT	12
24322	2.32E-06	16.55027	4.74E-05	0.025224	up	MMAB	ES	5
24365	2.48E-11	27.81081	1.34E-07	2.71E-07	down	TCHP	AT	16
24366	2.48E-11	27.81081	1.34E-07	2.71E-07	up	TCHP	AT	14.2
24405	9.14E-08	34.53081	4.20E-09	0.000995	down	C12orf76	AT	11
24406	1.26E-08	31.08	2.48E-08	0.000137	up	C12orf76	AT	6
24427	2.57E-07	16.55027	4.74E-05	0.002803	down	FAM216A	AT	7
24428	2.57E-07	16.55027	4.74E-05	0.002803	up	FAM216A	AT	5.2
24444	2.86E-15	38.16324	6.51E-10	3.12E-11	up	VPS29	ES	3.1:3.2
24499	3.13E-14	34.53081	4.20E-09	3.41E-10	down	PPP1CC	AT	5.4
24501	3.11E-14	34.53081	4.20E-09	3.39E-10	up	PPP1CC	AT	6.3
24532	6.01E-08	30.33831	3.63E-08	0.000655	up	ACAD10	AT	19
24534	3.67E-06	21.8173	3.00E-06	0.039946	down	ACAD10	AT	23
24567	4.02E-20	50.15027	1.42E-12	4.38E-16	down	ERP29	AP	1
24568	3.93E-20	50.15027	1.42E-12	4.28E-16	up	ERP29	AP	2
24569	5.23E-16	45.97297	1.20E-11	5.69E-12	down	ERP29	ES	3
24645	8.47E-15	45.97297	1.20E-11	9.22E-11	down	RBM19	AT	24.3
24646	8.47E-15	45.97297	1.20E-11	9.22E-11	up	RBM19	AT	25
24730	2.16E-08	35.81457	2.17E-09	0.000235	down	RPLP0	ES	5.2:5.3
24760	4.08E-08	21.71496	3.16E-06	0.000444	down	DYNLL1	AT	5.2
24761	4.02E-08	19.24652	1.15E-05	0.000438	up	DYNLL1	AT	4.5
24866	1.97E-09	31.08	2.48E-08	2.14E-05	up	RNF34	AT	7.2
24867	1.97E-09	31.08	2.48E-08	2.14E-05	down	RNF34	AT	8
24875	7.91E-08	19.09297	1.25E-05	0.000862	down	KDM2B	AT	12
24995	2.85E-07	16.55027	4.74E-05	0.003108	up	ABCB9	AP	2
25005	1.55E-15	38.16324	6.51E-10	1.69E-11	up	OGFOD2	AP	1
25006	1.55E-15	38.16324	6.51E-10	1.69E-11	down	OGFOD2	AP	2
25024	6.74E-21	45.97297	1.20E-11	7.33E-17	up	ARL6IP4	RI	5.2
25026	1.21E-10	31.08	2.48E-08	1.32E-06	down	ARL6IP4	AA	3.1:3.2
25027	4.61E-09	19.09297	1.25E-05	5.02E-05	down	ARL6IP4	AA	3.2
25032	4.23E-07	24.72324	6.62E-07	0.004609	up	ARL6IP4	AD	2.2
25050	6.30E-13	41.9773	9.23E-11	6.86E-09	up	MPHOSPH	AT	26
25051	6.30E-13	41.9773	9.23E-11	6.86E-09	down	MPHOSPH	AT	25
25074	6.98E-11	19.40821	1.06E-05	7.60E-07	down	SNRNP35	AP	2
25076	6.40E-09	16.55027	4.74E-05	6.97E-05	up	SNRNP35	AP	1
25282	1.22E-08	34.53081	4.20E-09	0.000133	up	PXMP2	AT	12
25283	1.22E-08	34.53081	4.20E-09	0.000133	down	PXMP2	AT	7.2
25285	2.97E-09	31.482	2.01E-08	3.23E-05	down	PXMP2	ES	4
25293	1.58E-07	14.18919	0.000165	0.001723	down	PGAM5	AT	8
25294	1.59E-07	14.18919	0.000165	0.001729	up	PGAM5	AT	7
25304	4.57E-06	14.18919	0.000165	0.049797	up	GOLGA3	AT	16.2
25544	3.10E-10	38.16324	6.51E-10	3.37E-06	up	POLR1D	AT	3
25545	3.10E-10	38.16324	6.51E-10	3.37E-06	down	POLR1D	AT	5
25571	1.01E-06	18.36294	1.83E-05	0.011043	down	HMGB1	ES	7.1
25801	4.29E-09	24.25325	8.45E-07	4.67E-05	down	TPT1	ES	1.4
25899	7.96E-09	25.08186	5.49E-07	8.66E-05	down	EBPL	AT	6
25900	7.96E-09	27.43434	1.63E-07	8.66E-05	up	EBPL	AT	3
25911	6.65E-07	14.18919	0.000165	0.007242	down	EBPL	ES	2:4.1:4.2
25950	4.15E-11	34.53081	4.20E-09	4.52E-07	down	DHRS12	AT	10.3
25951	4.15E-11	34.53081	4.20E-09	4.52E-07	up	DHRS12	AT	9
25952	2.17E-09	27.81081	1.34E-07	2.36E-05	up	DHRS12	RI	10.2
26000	1.21E-09	21.8173	3.00E-06	1.31E-05	up	CKAP2	AT	7.2
26001	1.21E-09	21.8173	3.00E-06	1.31E-05	down	CKAP2	AT	10
26129	1.26E-15	38.16324	6.51E-10	1.38E-11	up	UGGT2	AT	42
26130	1.09E-11	34.53081	4.20E-09	1.19E-07	down	UGGT2	AT	8

26131	5.14E-11	34.53081	4.20E-09	5.59E-07	down	UGGT2	AT	11
26164	1.72E-08	34.95451	3.37E-09	0.000187	down	FARP1	AT	4
26165	1.72E-08	34.11125	5.20E-09	0.000187	up	FARP1	AT	30
26185	2.30E-09	31.08	2.48E-08	2.51E-05	up	CLYBL	AT	8
26186	2.30E-09	31.08	2.48E-08	2.51E-05	down	CLYBL	AT	9.3
26216	6.13E-08	19.09297	1.25E-05	0.000667	down	TEX30	ES	5
26360	7.39E-07	24.72324	6.62E-07	0.008048	down	PCID2	RI	14.2:14.3
26372	3.83E-06	16.55027	4.74E-05	0.04166	down	GRTP1	AT	8
26373	3.83E-06	16.55027	4.74E-05	0.04166	up	GRTP1	AT	7.2
26426	2.41E-07	11.76263	0.000604	0.002628	up	PARP2	RI	15.2
26442	5.82E-11	27.81081	1.34E-07	6.34E-07	up	OSGEP	AD	4.5
26443	8.07E-11	24.72324	6.62E-07	8.79E-07	up	OSGEP	RI	4.3
26458	3.22E-07	31.08	2.48E-08	0.003502	down	RNASE4	AP	2
26459	3.22E-07	31.08	2.48E-08	0.003502	up	RNASE4	AP	1.1
26463	2.07E-06	27.81081	1.34E-07	0.022524	down	ANG	AP	2
26464	2.07E-06	27.81081	1.34E-07	0.022524	up	ANG	AP	1
26473	4.16E-10	24.72324	6.62E-07	4.53E-06	up	METTL17	RI	10.2:10.3
26475	9.54E-19	50.15027	1.42E-12	1.04E-14	up	METTL17	RI	10.2
26476	1.70E-12	30.68198	3.04E-08	1.85E-08	up	METTL17	RI	9.2
26477	4.40E-08	21.8173	3.00E-06	0.000479	up	METTL17	RI	8.2
26542	7.37E-14	45.97297	1.20E-11	8.02E-10	up	HNRNPC	AD	4.2
26571	8.96E-10	18.78117	1.47E-05	9.75E-06	up	SUPT16H	AT	2
26572	8.96E-10	19.72694	8.93E-06	9.75E-06	down	SUPT16H	AT	28
26595	1.69E-08	19.09297	1.25E-05	0.000184	up	METTL3	RI	8.4
26645	2.41E-07	16.55027	4.74E-05	0.002619	down	MRPL52	AA	1.4
26646	5.72E-09	24.72324	6.62E-07	6.23E-05	up	MRPL52	RI	1.2:1.3
26688	3.93E-15	45.97297	1.20E-11	4.28E-11	down	PSMB5	ES	3
26731	2.54E-12	37.72214	8.16E-10	2.76E-08	up	PABPN1	RI	5.2
26756	4.96E-07	12.00973	0.000529	0.005402	up	NGDN	RI	10.2
26778	9.21E-16	51.17628	8.44E-13	1.00E-11	down	DHRS4	AT	8
26779	9.21E-16	48.1559	3.94E-12	1.00E-11	up	DHRS4	AT	7.2
26781	7.56E-07	14.18919	0.000165	0.008232	down	DHRS4	ES	4
26782	3.17E-13	31.08	2.48E-08	3.45E-09	down	DHRS4	ES	4:05:06
26787	5.03E-07	21.8173	3.00E-06	0.005481	down	DHRS4	ES	3:4:5:6
26797	5.50E-18	49.14366	2.38E-12	5.99E-14	up	DHRS4L2	AT	8.2
26799	5.44E-18	52.75289	3.78E-13	5.92E-14	down	DHRS4L2	AT	9
26801	4.78E-12	41.9773	9.23E-11	5.20E-08	down	DHRS4L2	ES	5:06:07
26815	1.88E-15	51.17628	8.44E-13	2.04E-11	down	PCK2	AT	12.2
26817	1.88E-15	49.64457	1.84E-12	2.04E-11	up	PCK2	AT	9.2
26820	3.55E-10	34.53081	4.20E-09	3.86E-06	down	PCK2	ES	11
26852	9.94E-10	20.18524	7.03E-06	1.08E-05	up	PSME1	RI	10.2:10.3
26856	3.11E-07	14.18919	0.000165	0.003385	up	EMC9	AA	3.1:3.2
26857	2.13E-13	31.08	2.48E-08	2.32E-09	up	EMC9	AA	3.2
26932	6.12E-09	31.08	2.48E-08	6.66E-05	down	TINF2	RI	6.4
26954	7.61E-23	50.15027	1.42E-12	8.28E-19	up	DHRS1	RI	6.3
27347	1.16E-10	34.53081	4.20E-09	1.27E-06	down	SEC23A	AT	23
27348	1.17E-10	34.53081	4.20E-09	1.28E-06	up	SEC23A	AT	4.2
27421	1.13E-10	41.51466	1.17E-10	1.24E-06	up	RPS29	AT	3
27422	1.13E-10	43.87327	3.50E-11	1.23E-06	down	RPS29	AT	4
27633	7.49E-07	19.09297	1.25E-05	0.00815	down	KTN1	ES	46
27882	3.36E-07	24.72324	6.62E-07	0.003655	down	ZBTB25	AT	7.3
27883	3.36E-07	24.72324	6.62E-07	0.003655	up	ZBTB25	AT	8
27892	1.94E-10	31.08	2.48E-08	2.11E-06	down	ZBTB1	AT	4.2
27893	1.94E-10	31.08	2.48E-08	2.11E-06	up	ZBTB1	AT	5
27931	3.41E-07	19.09297	1.25E-05	0.003715	down	MAX	AT	4
27932	6.74E-07	21.8173	3.00E-06	0.007335	up	MAX	AT	5.8
28155	3.30E-10	31.482	2.01E-08	3.59E-06	down	SRSF5	AT	10
28157	3.30E-10	30.68198	3.04E-08	3.59E-06	up	SRSF5	AT	7.3

28158	4.84E-13	34.53081	4.20E-09	5.27E-09	up	SRSF5	AA		8.1
28231	1.54E-07	24.01706	9.55E-07	0.001673	up	DCAF4	AT		14
28232	1.54E-07	25.08186	5.49E-07	0.001673	down	DCAF4	AT		13.2
28301	1.85E-11	27.81081	1.34E-07	2.02E-07	down	ACOT2	AD		2.3
28366	4.07E-14	31.08	2.48E-08	4.43E-10	down	ALDH6A1	AP		1
28367	4.07E-14	31.08	2.48E-08	4.43E-10	up	ALDH6A1	AP		7.1
29243	2.77E-07	24.72324	6.62E-07	0.00302	down	EVL	AT		17
29244	2.77E-07	24.72324	6.62E-07	0.00302	up	EVL	AT		14.2
29304	6.86E-08	18.47277	1.72E-05	0.000747	up	WDR25	AT		4
29305	6.86E-08	19.40821	1.06E-05	0.000747	down	WDR25	AT		9
29315	2.85E-07	24.72324	6.62E-07	0.0031	down	PPP2R5C	AT		9
29467	5.87E-09	31.08	2.48E-08	6.40E-05	down	KLC1	AT		19
29495	1.97E-19	45.97297	1.20E-11	2.15E-15	down	XRCC3	AT		8
29496	1.97E-19	45.97297	1.20E-11	2.15E-15	up	XRCC3	AT		12
29536	2.26E-10	21.8173	3.00E-06	2.46E-06	up	C14orf2	ES		3
29547	8.84E-11	24.72324	6.62E-07	9.62E-07	down	INF2	ES		22
29686	1.05E-06	15.97308	6.42E-05	0.011383	up	NIPA1	AP		1
29789	1.46E-06	12.00973	0.000529	0.015893	up	MTMR10	RI		18.2
29892	7.36E-13	38.16324	6.51E-10	8.01E-09	up	DPH6	AT		10
29893	7.36E-13	38.16324	6.51E-10	8.01E-09	down	DPH6	AT		4
29932	3.14E-07	21.8173	3.00E-06	0.003423	up	EIF2AK4	AT		4
29990	2.40E-09	21.8173	3.00E-06	2.61E-05	up	C15orf57	AT		5.3
30030	8.23E-12	34.53081	4.20E-09	8.96E-08	down	GCHFR	AP		2
30031	5.57E-09	27.81081	1.34E-07	6.07E-05	up	GCHFR	AP		3.1
30032	1.67E-12	33.13096	8.62E-09	1.81E-08	down	GCHFR	AT		4
30033	1.59E-12	36.85262	1.27E-09	1.73E-08	up	GCHFR	AT		3.4
30087	1.08E-18	45.97297	1.20E-11	1.17E-14	up	NDUFAF1	ES		4
30134	9.00E-07	16.55027	4.74E-05	0.0098	up	TMEM87A	AT		22
30136	1.46E-09	19.40821	1.06E-05	1.59E-05	down	TMEM87A	AT		8
30345	4.23E-07	9.786374	0.001758	0.004607	up	SPG11	AT		32.2
30378	9.58E-13	46.4618	9.34E-12	1.04E-08	down	SORD	AT		11
30379	9.71E-13	45.4888	1.54E-11	1.06E-08	up	SORD	AT		9
30410	3.08E-11	49.64457	1.84E-12	3.35E-07	up	SHF	AT		9.2
30411	3.08E-11	50.66081	1.10E-12	3.35E-07	down	SHF	AT		10
30419	5.42E-08	40.153	2.35E-10	0.000591	up	GATM	AT		9.2
30420	5.42E-08	39.97131	2.58E-10	0.000591	down	GATM	AT		10
30572	2.77E-07	12.00973	0.000529	0.00302	down	GABPB1	AT		10
30573	2.77E-07	12.00973	0.000529	0.00302	up	GABPB1	AT		9.2
31015	1.15E-12	41.9773	9.23E-11	1.25E-08	down	RPS27L	AT		6
31016	1.16E-12	41.9773	9.23E-11	1.26E-08	up	RPS27L	AT		5.2
31082	1.48E-09	44.06361	3.18E-11	1.62E-05	up	FAM96A	AT		5.2
31083	1.48E-09	46.4618	9.34E-12	1.62E-05	down	FAM96A	AT		6
31091	1.11E-06	16.55027	4.74E-05	0.012092	down	SNX1	AA		16.1
31269	2.31E-10	27.81081	1.34E-07	2.52E-06	up	SNAPC5	AT		3.3
31270	2.31E-10	27.81081	1.34E-07	2.52E-06	down	SNAPC5	AT		4
31374	4.10E-06	14.18919	0.000165	0.044627	down	ANP32A	AA		3.1
31540	1.90E-13	42.44441	7.27E-11	2.07E-09	down	HEXA	AT		15
31542	4.20E-09	21.8173	3.00E-06	4.57E-05	up	HEXA	AT		14.2
31543	4.78E-12	27.81081	1.34E-07	5.20E-08	up	HEXA	AT		13.2
31544	1.44E-06	8.195676	0.004199	0.015714	down	HEXA	ES		11
31774	1.07E-14	45.4888	1.54E-11	1.17E-10	up	MPI	AT		5.2
31775	8.97E-18	50.15027	1.42E-12	9.77E-14	down	MPI	AT		8
31776	2.77E-15	41.51466	1.17E-10	3.02E-11	up	MPI	AT		6.2
31878	4.15E-18	50.15027	1.42E-12	4.52E-14	up	SIN3A	AT		22
31879	4.17E-18	50.15027	1.42E-12	4.54E-14	down	SIN3A	AT		4.2
31913	1.08E-08	19.09297	1.25E-05	0.000118	up	NRG4	AT		17.3
31921	2.26E-13	34.53081	4.20E-09	2.46E-09	up	C15orf27	AT		11.2
31922	2.26E-13	34.53081	4.20E-09	2.46E-09	down	C15orf27	AT		12

32162	5.03E-08	16.55027	4.74E-05	0.000547	down	ST20	AP	1
32164	9.56E-09	27.81081	1.34E-07	0.000104	up	ST20	AP	2
32289	2.19E-09	21.8173	3.00E-06	2.39E-05	up	ZSCAN2	AT	5.7
32290	2.64E-09	19.09297	1.25E-05	2.88E-05	down	ZSCAN2	AT	3
32376	2.72E-06	10.47204	0.001212	0.029641	down	MRPS11	ES	3
32756	4.89E-08	24.72324	6.62E-07	0.000532	up	VIMP	AT	8
32757	4.90E-08	24.72324	6.62E-07	0.000534	down	VIMP	AT	7.2
32758	6.64E-14	37.72214	8.16E-10	7.23E-10	up	SNRPA1	ES	6
32759	4.19E-07	34.95451	3.37E-09	0.004561	down	PCSK6	AT	15
32760	4.19E-07	33.28432	7.96E-09	0.004561	up	PCSK6	AT	24
32767	5.71E-10	38.16324	6.51E-10	6.21E-06	up	TM2D3	AT	6
32768	5.75E-10	38.16324	6.51E-10	6.26E-06	down	TM2D3	AT	5
32778	3.13E-08	16.26003	5.52E-05	0.00034	up	WASH4P	RI	5.2
32901	4.01E-14	50.15027	1.42E-12	4.37E-10	up	PIGQ	AT	6.4
32902	3.99E-14	50.15027	1.42E-12	4.35E-10	down	PIGQ	AT	14
32916	1.70E-07	34.53081	4.20E-09	0.001851	down	C16orf13	ES	3:04
32917	1.08E-11	45.97297	1.20E-11	1.17E-07	down	C16orf13	ES	3
32919	2.48E-11	31.08	2.48E-08	2.71E-07	down	C16orf13	ES	2:03:04
32921	1.82E-13	46.4618	9.34E-12	1.98E-09	down	C16orf13	ES	2:03
32924	1.06E-13	46.95535	7.26E-12	1.16E-09	down	C16orf13	ES	2
32963	6.87E-11	27.81081	1.34E-07	7.48E-07	up	FAM173A	RI	4.2
33076	2.82E-07	19.09297	1.25E-05	0.003069	up	GNPTG	ES	4
33137	1.82E-11	41.9773	9.23E-11	1.98E-07	up	NUBP2	RI	6.2
33150	1.90E-07	34.53081	4.20E-09	0.002067	down	FAHD1	AT	3
33151	1.90E-07	34.53081	4.20E-09	0.002067	up	FAHD1	AT	1.2
33162	3.80E-07	15.97308	6.42E-05	0.004142	up	MSRB1	RI	2.2:2.3:2.4
33166	5.38E-07	24.72324	6.62E-07	0.005858	up	NDUFB10	RI	3.2
33188	2.09E-15	42.44441	7.27E-11	2.27E-11	down	SLC9A3R2	AP	2.1
33302	2.92E-06	12.00973	0.000529	0.031798	up	TCEB2	RI	4.2
33330	1.89E-21	45.97297	1.20E-11	2.06E-17	down	PKMYT1	AA	9.1:9.2
33354	5.13E-08	21.8173	3.00E-06	0.000559	down	HCFC1R1	ES	2
33387	1.78E-09	19.40821	1.06E-05	1.94E-05	down	IL32	AA	1.8
33417	5.26E-08	16.55027	4.74E-05	0.000573	up	IL32	RI	1.2:1.3:1.4:
33426	1.74E-07	16.55027	4.74E-05	0.001895	up	IL32	RI	1.2:1.3:1.4:
33428	3.30E-08	27.81081	1.34E-07	0.000359	up	IL32	AA	1.4:1.5:1.6:
33438	4.14E-06	16.26003	5.52E-05	0.045129	up	IL32	ES	1.4:1.5:1.6
33442	1.96E-07	18.78117	1.47E-05	0.00213	up	IL32	AD	1.2:1.3:1.4
33444	2.02E-06	16.26003	5.52E-05	0.021978	up	IL32	ES	1.4
33654	5.76E-07	12.00973	0.000529	0.006268	up	PAM16	AA	8.1:8.2
33952	8.78E-12	27.81081	1.34E-07	9.56E-08	down	CARHSP1	AP	3
33955	1.31E-09	18.78117	1.47E-05	1.42E-05	up	CARHSP1	AT	5.2
33956	1.31E-09	20.04919	7.55E-06	1.42E-05	down	CARHSP1	AT	6
34070	3.04E-06	12.25994	0.000463	0.033139	down	MKL2	AT	19
34071	3.04E-06	11.76263	0.000604	0.033139	up	MKL2	AT	12.3
34149	3.24E-09	24.72324	6.62E-07	3.53E-05	up	NPIPA5	AD	8.2:8.3
34150	7.13E-15	38.16324	6.51E-10	7.77E-11	up	NPIPA5	ES	8.3
34219	2.95E-20	50.15027	1.42E-12	3.21E-16	up	ABCC6	AT	2.2
34220	2.95E-20	50.15027	1.42E-12	3.21E-16	down	ABCC6	AT	32
34253	3.45E-07	17.86599	2.37E-05	0.003756	up	RPS15A	RI	5.2
34274	2.35E-12	34.53081	4.20E-09	2.56E-08	up	SMG1	AT	14.2
34275	2.35E-12	34.53081	4.20E-09	2.56E-08	down	SMG1	AT	64
34279	2.89E-06	14.18919	0.000165	0.031489	down	TMC7	AT	16.2
34280	2.89E-06	14.18919	0.000165	0.031489	up	TMC7	AT	17
34290	1.30E-06	16.55027	4.74E-05	0.014153	down	SYT17	AP	1
34292	1.09E-06	16.55027	4.74E-05	0.011863	up	SYT17	AP	2.1
34405	3.62E-12	27.81081	1.34E-07	3.94E-08	down	LYRM1	AT	10
34406	3.60E-12	27.81081	1.34E-07	3.92E-08	up	LYRM1	AT	9
35904	3.80E-07	19.40821	1.06E-05	0.004136	down	SPNS1	ES	7

35952	2.84E-07	243.8095	5.81E-55	0.003089	down	MAZ	ES	3.1:3.2
35990	1.28E-06	12.00973	0.000529	0.013983	down	TMEM219	AP	2
35991	1.40E-06	12.00973	0.000529	0.015212	up	TMEM219	AP	3.1
36016	1.23E-08	21.8173	3.00E-06	0.000134	down	INO80E	ES	6.2:6.3
36018	4.28E-12	34.53081	4.20E-09	4.66E-08	up	INO80E	AA	6.1
36056	5.46E-08	14.18919	0.000165	0.000594	up	PPP4C	AD	5.2
36074	2.89E-09	19.09297	1.25E-05	3.15E-05	up	YPEL3	AD	3.2
36088	4.05E-10	28.57531	9.01E-08	4.40E-06	down	MAPK3	ES	6.1
36156	4.39E-07	24.72324	6.62E-07	0.004777	up	ZNF688	AT	3
36157	4.39E-07	24.72324	6.62E-07	0.004777	down	ZNF688	AT	4
36161	1.29E-08	19.09297	1.25E-05	0.00014	down	ZNF785	AT	3.2
36162	1.29E-08	19.09297	1.25E-05	0.00014	up	ZNF785	AT	4
36207	7.50E-09	24.72324	6.62E-07	8.17E-05	up	ORAI3	AT	3.3
36208	7.50E-09	24.72324	6.62E-07	8.17E-05	down	ORAI3	AT	4
36229	4.45E-06	14.18919	0.000165	0.048496	down	VKORC1	ES	5
36540	9.32E-10	31.08	2.48E-08	1.01E-05	down	CX3CL1	AP	1
36541	9.32E-10	31.08	2.48E-08	1.01E-05	up	CX3CL1	AP	2.1
36570	1.13E-06	16.26003	5.52E-05	0.012334	up	DOK4	RI	8.2
36902	4.34E-08	31.482	2.01E-08	0.000472	down	CES2	AD	10.2
36985	4.20E-07	19.09297	1.25E-05	0.004571	up	TMEM208	RI	5.2
36986	1.30E-09	21.8173	3.00E-06	1.41E-05	up	TMEM208	AD	4.2
37126	8.44E-08	24.72324	6.62E-07	0.000919	down	CENPT	AT	16.2
37127	8.44E-08	24.72324	6.62E-07	0.000919	up	CENPT	AT	10.2
37178	5.45E-13	34.53081	4.20E-09	5.93E-09	up	NFATC3	AT	9.2
37183	1.91E-12	31.08	2.48E-08	2.08E-08	down	NFATC3	AT	13
37288	8.14E-14	37.72214	8.16E-10	8.86E-10	up	NFAT5	AT	13.3
37289	2.24E-11	27.81081	1.34E-07	2.44E-07	down	NFAT5	AT	17
37343	4.55E-13	45.97297	1.20E-11	4.95E-09	up	DDX19B	AT	8
37347	4.55E-13	45.97297	1.20E-11	4.95E-09	down	DDX19B	AT	15
37385	1.77E-07	16.55027	4.74E-05	0.001927	up	FUK	AT	6.2
37386	1.78E-07	16.55027	4.74E-05	0.00194	down	FUK	AT	24
37526	1.16E-09	37.50132	9.14E-10	1.26E-05	up	HP	AT	7.2
37527	1.14E-09	42.34784	7.64E-11	1.24E-05	down	HP	AT	8.2
37559	8.81E-10	21.48394	3.57E-06	9.59E-06	up	PSMD7	AT	7.2
37560	8.81E-10	22.15422	2.52E-06	9.59E-06	down	PSMD7	AT	8.2
37576	8.97E-08	24.72324	6.62E-07	0.000976	down	ZNRF1	AT	6.2
37577	8.97E-08	24.72324	6.62E-07	0.000976	up	ZNRF1	AT	4.2
37702	1.24E-10	27.81081	1.34E-07	1.35E-06	down	CMC2	AP	2
37703	3.35E-10	24.72324	6.62E-07	3.65E-06	up	CMC2	AP	1
37728	2.57E-06	19.09297	1.25E-05	0.027966	down	CMC2	ES	9
37730	1.03E-09	19.09297	1.25E-05	1.12E-05	down	CMC2	ES	7
37739	5.80E-11	31.08	2.48E-08	6.31E-07	down	CENPN	AT	12
37740	2.10E-11	27.81081	1.34E-07	2.28E-07	up	CENPN	AT	8
37779	3.66E-10	31.08	2.48E-08	3.99E-06	up	CDH13	AT	12.2
37903	6.87E-08	24.72324	6.62E-07	0.000748	up	COX4I1	RI	5.2:5.3:5.4
37907	8.50E-12	37.72214	8.16E-10	9.26E-08	up	COX4I1	RI	5.2:5.3
38010	3.48E-08	16.55027	4.74E-05	0.000379	down	MVD	ES	3:04
38039	1.80E-06	15.97308	6.42E-05	0.019614	up	TRAPPC2L	RI	5.2:5.3
38091	9.84E-07	12.87731	0.000333	0.010716	up	RPL13	RI	1.2
38142	8.52E-12	31.08	2.48E-08	9.28E-08	up	FANCA	AT	10.4
38143	8.52E-12	31.08	2.48E-08	9.28E-08	down	FANCA	AT	43
38461	2.09E-06	12.00973	0.000529	0.022726	down	SHPK	AT	7.4
38462	1.85E-08	19.09297	1.25E-05	0.000202	up	SHPK	AT	22
38463	3.36E-07	12.00973	0.000529	0.003655	down	SHPK	AT	14.2
38471	4.95E-07	12.00973	0.000529	0.005385	down	CTNS	AT	13.3
38472	4.93E-07	12.00973	0.000529	0.005368	up	CTNS	AT	6
38577	8.50E-13	34.53081	4.20E-09	9.26E-09	down	MED11	AT	3.2
38578	8.46E-13	34.53081	4.20E-09	9.22E-09	up	MED11	AT	2.2

38607	3.05E-06	14.73621	0.000124	0.033165	down	RNF167	ES	7:08
38666	2.72E-14	38.16324	6.51E-10	2.97E-10	up	SCIMP	AT	5
38667	2.72E-14	38.16324	6.51E-10	2.97E-10	down	SCIMP	AT	6
38689	1.63E-09	27.81081	1.34E-07	1.77E-05	down	RPAIN	ES	4
38690	3.17E-09	31.08	2.48E-08	3.45E-05	down	RPAIN	ES	05:06.1
38720	1.11E-06	14.18919	0.000165	0.012107	up	NLRP1	AT	19
38825	7.68E-09	12.25994	0.000463	8.36E-05	down	C17orf49	ES	3
38871	3.11E-09	33.69577	6.44E-09	3.38E-05	up	GABARAP	RI	1.2:1.3:1.4
38908	2.79E-12	30.68198	3.04E-08	3.04E-08	up	EIF5A	AP	3
38995	1.94E-12	41.9773	9.23E-11	2.11E-08	up	MPDU1	AT	9
38996	6.18E-09	26.69271	2.39E-07	6.73E-05	up	MPDU1	AT	1.2
38997	1.11E-11	45.97297	1.20E-11	1.21E-07	up	MPDU1	AT	4
39005	9.70E-12	31.08	2.48E-08	1.06E-07	up	MPDU1	AA	5.1
39032	2.88E-08	24.72324	6.62E-07	0.000314	up	SAT2	AD	1.2
39058	4.76E-09	19.09297	1.25E-05	5.18E-05	up	LSMD1	AP	1
39059	1.83E-07	21.8173	3.00E-06	0.001993	down	LSMD1	AP	5.1
39093	4.26E-08	21.8173	3.00E-06	0.000464	down	PER1	AT	24
39094	4.22E-08	21.8173	3.00E-06	0.000459	up	PER1	AT	19.3
39105	8.70E-13	31.08	2.48E-08	9.47E-09	up	VAMP2	RI	5.2
39163	5.17E-16	45.97297	1.20E-11	5.63E-12	up	RANGRF	RI	3.4
39175	4.15E-06	14.75513	0.000122	0.045229	up	RPL26	AT	4.2
39334	6.80E-08	16.55027	4.74E-05	0.00074	up	COX10	AT	7
39335	6.80E-08	16.55027	4.74E-05	0.00074	down	COX10	AT	3.2
39416	6.04E-12	38.60865	5.18E-10	6.57E-08	down	NCOR1	AT	46
39418	6.04E-12	37.28529	1.02E-09	6.57E-08	up	NCOR1	AT	22.2
39432	8.10E-07	16.55027	4.74E-05	0.008819	up	UBB	AP	2.1
39433	8.07E-07	17.14076	3.47E-05	0.008791	down	UBB	AP	1
39434	1.32E-06	14.73621	0.000124	0.014333	down	UBB	RI	3.2
39440	1.17E-07	14.18919	0.000165	0.001277	down	CCDC144A	AT	28
39485	4.80E-08	28.28851	1.05E-07	0.000523	down	PEMT	AP	3
39758	2.19E-06	14.73621	0.000124	0.023797	down	ALDH3A2	ES	9
39790	2.23E-13	31.08	2.48E-08	2.43E-09	up	SPECC1	AT	18
39792	2.24E-13	31.08	2.48E-08	2.44E-09	down	SPECC1	AT	10
39892	2.34E-09	21.8173	3.00E-06	2.55E-05	up	TMEM199	ES	5
39967	1.44E-06	10.47204	0.001212	0.015725	down	RPL23A	AP	1
40063	2.93E-06	19.09297	1.25E-05	0.031872	up	SSH2	AT	11
40365	1.27E-06	16.55027	4.74E-05	0.013881	up	CCL16	ES	2:03
40493	2.02E-06	19.09297	1.25E-05	0.021975	up	GGNBP2	AT	14
40494	2.02E-06	19.09297	1.25E-05	0.022043	down	GGNBP2	AT	2.2
40508	7.44E-08	21.8173	3.00E-06	0.00081	up	ACACA	AT	60
40509	7.44E-08	21.8173	3.00E-06	0.00081	down	ACACA	AT	5
40592	1.18E-06	15.58155	7.90E-05	0.01282	down	PSMB3	ES	4.1:4.2
40839	4.34E-08	19.09297	1.25E-05	0.000472	down	THRA	AT	12.2
40840	4.34E-08	19.09297	1.25E-05	0.000472	up	THRA	AT	11.2
40932	1.93E-07	24.72324	6.62E-07	0.002102	up	JUP	RI	15.2
41129	2.60E-11	39.05842	4.11E-10	2.84E-07	down	COA3	AT	2
41130	2.60E-11	35.99961	1.97E-09	2.84E-07	up	COA3	AT	4
41171	1.74E-08	22.15422	2.52E-06	0.00019	down	RPL27	AP	2
41172	3.27E-07	18.78117	1.47E-05	0.003556	up	RPL27	AP	3.1
41174	5.91E-10	15.56697	7.96E-05	6.43E-06	up	RPL27	AP	1
41693	9.28E-11	20.50479	5.95E-06	1.01E-06	up	NBR1	AT	23
41694	9.28E-11	23.89395	1.02E-06	1.01E-06	down	NBR1	AT	24
41753	4.54E-06	6.934426	0.008455	0.049397	down	TMEM101	AT	5
41754	4.54E-06	6.022542	0.014124	0.049397	up	TMEM101	AT	4.2
41930	3.12E-06	12.00973	0.000529	0.033949	up	DCAKD	AT	5
41931	3.12E-06	12.00973	0.000529	0.033949	down	DCAKD	AT	7
41941	2.58E-19	50.15027	1.42E-12	2.80E-15	down	ACBD4	AT	12
41942	2.58E-19	50.15027	1.42E-12	2.80E-15	up	ACBD4	AT	11

42017	2.79E-08	24.72324	6.62E-07	0.000304	up	ARL17B	AT	9
42076	9.18E-09	19.09297	1.25E-05	1.00E-04	up	NPEPPS	AT	15.2
42078	9.18E-09	19.09297	1.25E-05	1.00E-04	down	NPEPPS	AT	26
42120	2.30E-16	45.97297	1.20E-11	2.50E-12	up	SCRN2	RI	7.2
42121	8.13E-08	16.55027	4.74E-05	0.000885	up	SCRN2	AA	6.1
42124	1.83E-07	22.15422	2.52E-06	0.001993	down	SCRN2	ES	3
42236	1.37E-07	18.16772	2.02E-05	0.001495	up	ATP5G1	AT	4.3
42237	1.37E-07	16.84383	4.06E-05	0.001489	down	ATP5G1	AT	5
42238	5.98E-10	26.18036	3.11E-07	6.51E-06	down	ATP5G1	AA	4.1
42247	1.57E-11	34.11125	5.20E-09	1.71E-07	up	SNF8	ES	8
42374	2.07E-06	16.26003	5.52E-05	0.022517	up	MRPL27	RI	5.2:5.3:5.4:!
42503	3.81E-10	27.06166	1.97E-07	4.15E-06	up	NME1	ES	3
42507	1.21E-06	14.18919	0.000165	0.013176	down	NME1-NM	AP	8.1
42511	3.36E-06	19.72694	8.93E-06	0.036616	down	NME1-NM	RI	8.2:8.3
42534	1.80E-13	41.9773	9.23E-11	1.96E-09	up	TOM1L1	AT	12.2
42538	1.51E-13	41.9773	9.23E-11	1.65E-09	down	TOM1L1	AT	19
42567	2.46E-09	19.09297	1.25E-05	2.68E-05	down	COX11	RI	3.2:3.3
42630	7.10E-16	41.9773	9.23E-11	7.73E-12	down	SRSF1	RI	3.6
42793	7.56E-08	27.81081	1.34E-07	0.000823	up	PTRH2	ES	2.2
43144	2.44E-13	37.72214	8.16E-10	2.65E-09	up	PRKAR1A	AT	13
43146	2.44E-13	32.29817	1.32E-08	2.65E-09	down	PRKAR1A	AT	12
43199	1.28E-06	14.46107	0.000143	0.01398	down	SLC39A11	AT	13
43228	1.48E-10	28.96344	7.38E-08	1.61E-06	down	RPL38	AT	4.2
43229	1.48E-10	26.69271	2.39E-07	1.61E-06	up	RPL38	AT	3.2
43343	1.09E-10	28.19112	1.10E-07	1.18E-06	down	ATP5H	ME	4|5
43357	5.12E-09	19.09297	1.25E-05	5.57E-05	up	NT5C	AP	2.1
43358	5.12E-09	19.09297	1.25E-05	5.57E-05	down	NT5C	AP	1
43361	5.38E-07	16.55027	4.74E-05	0.005857	up	NT5C	RI	4.2
43362	1.14E-11	27.81081	1.34E-07	1.24E-07	up	NT5C	AD	3.3
43384	1.72E-11	27.81081	1.34E-07	1.88E-07	up	NUP85	RI	18.2
43452	1.87E-06	14.46107	0.000143	0.020312	down	CASKIN2	AT	21
43453	1.87E-06	13.65504	0.00022	0.020376	up	CASKIN2	AT	11.2
43495	2.34E-11	24.36833	7.96E-07	2.55E-07	up	GALK1	RI	4.4
43496	2.12E-14	27.81081	1.34E-07	2.31E-10	up	GALK1	RI	4.2
43660	1.65E-15	34.53081	4.20E-09	1.80E-11	down	SRSF2	RI	2.6
43663	5.26E-08	21.8173	3.00E-06	0.000573	up	SRSF2	RI	2.2:2.3:2.4
43664	1.03E-09	27.81081	1.34E-07	1.12E-05	up	SRSF2	AA	2.3:2.4
43665	9.92E-10	24.72324	6.62E-07	1.08E-05	up	SRSF2	AD	2.2:2.3
43702	2.90E-10	33.74146	6.29E-09	3.16E-06	down	SEC14L1	AP	8
43703	4.86E-12	34.53081	4.20E-09	5.30E-08	up	SEC14L1	AP	1
43776	1.05E-13	42.91607	5.71E-11	1.14E-09	down	SYNGR2	ES	4.1
43788	2.76E-14	38.16324	6.51E-10	3.01E-10	down	AFMID	AT	13
43789	2.77E-14	38.16324	6.51E-10	3.02E-10	up	AFMID	AT	4.2
43804	1.40E-20	50.15027	1.42E-12	1.53E-16	down	AFMID	ES	7:8:9:10:11
43928	2.04E-09	38.16324	6.51E-10	2.22E-05	down	LGALS3BP	AT	6
43930	4.89E-09	33.28432	7.96E-09	5.32E-05	up	LGALS3BP	AT	5.5
44053	1.46E-06	27.81081	1.34E-07	0.015889	up	ENDOV	AT	12.3
44054	6.50E-10	38.16324	6.51E-10	7.08E-06	down	ENDOV	AT	13
44056	1.58E-07	16.26003	5.52E-05	0.001725	up	ENDOV	AT	4
44114	1.96E-09	31.08	2.48E-08	2.14E-05	down	SLC38A10	AT	16
44115	1.96E-09	31.08	2.48E-08	2.14E-05	up	SLC38A10	AT	14.2
44199	6.25E-07	16.88261	3.98E-05	0.0068	up	ANAPC11	AP	7.1
44205	4.07E-08	20.18524	7.03E-06	0.000443	up	ANAPC11	ES	8
44206	9.83E-09	21.8173	3.00E-06	0.000107	up	ANAPC11	AD	7.3
44209	6.36E-10	21.8173	3.00E-06	6.92E-06	up	ANAPC11	ES	5:06
44210	1.48E-10	21.8173	3.00E-06	1.61E-06	up	ANAPC11	ES	3.4:6
44211	9.59E-07	19.09297	1.25E-05	0.01044	up	ANAPC11	ES	6
44212	4.33E-07	19.09297	1.25E-05	0.004715	up	ANAPC11	AD	3.4

44213	1.21E-08	16.55027	4.74E-05	0.000132	up	ANAPC11	ES		5
44215	2.88E-07	12.00973	0.000529	0.003131	up	ANAPC11	ES	3.3:5:6	
44220	4.21E-14	31.08	2.48E-08	4.58E-10	down	ANAPC11	AD		3.3
44221	1.37E-06	10.01189	0.001555	0.014888	up	ANAPC11	ES	3.3:5	
44230	1.19E-12	45.97297	1.20E-11	1.30E-08	down	PCYT2	ES		7
44256	1.27E-11	27.43434	1.63E-07	1.38E-07	up	ASPSCR1	ES		16
44264	2.08E-08	27.81081	1.34E-07	0.000226	up	STRA13	AD		3.2
44266	1.52E-08	38.16324	6.51E-10	0.000165	down	STRA13	ES		3.1
44331	4.17E-12	27.81081	1.34E-07	4.54E-08	up	HEXDC	RI	11.3:11.4	
44420	1.30E-06	24.164	8.85E-07	0.014154	up	TBCD	AT		22
44421	1.30E-06	243.8095	5.81E-55	0.014154	down	TBCD	AT		41
44459	1.07E-07	14.18919	0.000165	0.001165	up	ENOSF1	AP		11
44460	1.07E-07	14.18919	0.000165	0.001165	down	ENOSF1	AP		1.1
44462	3.23E-11	24.72324	6.62E-07	3.51E-07	up	ENOSF1	AT		19
44464	3.23E-11	24.72324	6.62E-07	3.51E-07	down	ENOSF1	AT		18.2
44492	7.03E-15	37.72214	8.16E-10	7.65E-11	up	MYL12A	AD		1.2
44494	1.55E-09	23.18684	1.47E-06	1.69E-05	down	MYL12B	AP		2.1
44495	1.70E-08	18.47277	1.72E-05	0.000185	up	MYL12B	AP		1
44591	1.20E-06	27.81081	1.34E-07	0.013051	up	NDUFV2	ES		1.3
44599	3.95E-06	14.18919	0.000165	0.043051	up	TWSG1	AT		6.2
44600	3.95E-06	14.18919	0.000165	0.043051	down	TWSG1	AT		7
44647	2.78E-10	31.08	2.48E-08	3.03E-06	up	MPPE1	ES		12
44716	3.69E-11	31.08	2.48E-08	4.02E-07	up	PTPN2	AT		13
44717	3.69E-11	31.08	2.48E-08	4.02E-07	down	PTPN2	AT		12.2
44797	5.82E-08	21.8173	3.00E-06	0.000634	up	TMEM241	AT		16
44800	5.82E-08	21.8173	3.00E-06	0.000634	down	TMEM241	AT		18
45144	2.61E-11	41.9773	9.23E-11	2.85E-07	down	ZNF397	AT		6
45145	4.04E-07	27.81081	1.34E-07	0.004404	up	ZNF397	AT		4.2
45155	1.49E-08	24.72324	6.62E-07	0.000162	down	ZSCAN30	AT		5
45264	3.23E-06	14.18919	0.000165	0.035213	down	TPGS2	AT		10
45266	6.68E-08	18.78117	1.47E-05	0.000728	up	TPGS2	AT		9.2
45442	2.41E-08	14.18919	0.000165	0.000262	up	IER3IP1	AT		3.2
45443	2.41E-08	14.18919	0.000165	0.000262	down	IER3IP1	AT		6
45575	4.62E-08	28.96344	7.38E-08	0.000503	down	MBD2	AT		8
45576	4.62E-08	27.43434	1.63E-07	0.000503	up	MBD2	AT		3.2
45685	3.94E-07	16.55027	4.74E-05	0.004289	up	SEC11C	ES		5
46259	2.13E-06	19.09297	1.25E-05	0.023154	down	PQLC1	ES		6:09
46271	7.18E-07	12.00973	0.000529	0.007819	down	TXNL4A	AP		3
46276	1.29E-07	28.57531	9.01E-08	0.001409	down	TXNL4A	ES		8
46289	1.22E-07	24.72324	6.62E-07	0.001325	down	TXNL4A	ES		4
46333	5.37E-10	31.08	2.48E-08	5.84E-06	up	MED16	RI		17.2
46360	6.95E-07	14.46107	0.000143	0.007566	down	TMEM259	ES		10
46382	7.32E-13	45.97297	1.20E-11	7.97E-09	down	POLR2E	AT		9
46383	7.29E-13	45.97297	1.20E-11	7.94E-09	up	POLR2E	AT		8
46423	3.45E-10	27.81081	1.34E-07	3.76E-06	up	CIRBP	RI		9.7
46432	2.49E-09	24.72324	6.62E-07	2.71E-05	up	CIRBP	RI		9.4
46446	1.75E-16	38.16324	6.51E-10	1.90E-12	up	C19orf24	AP		2
46447	1.75E-16	38.16324	6.51E-10	1.90E-12	down	C19orf24	AP		1
46466	3.80E-13	45.4888	1.54E-11	4.13E-09	up	NDUFS7	RI	9.2:9.3	
46484	1.20E-09	24.25325	8.45E-07	1.31E-05	down	RPS15	AD		3.4
46529	1.70E-09	20.82773	5.03E-06	1.85E-05	up	UQCR11	AT		2.2
46543	1.39E-12	38.16324	6.51E-10	1.51E-08	up	ATP8B3	AT		14.2
46544	1.39E-12	38.16324	6.51E-10	1.51E-08	down	ATP8B3	AT		29
46558	7.10E-11	34.53081	4.20E-09	7.73E-07	up	ABHD17A	AD		4.3
46587	2.61E-09	19.09297	1.25E-05	2.84E-05	up	PLEKHJ1	RI		5.2
46594	3.02E-07	20.50479	5.95E-06	0.003285	up	OAZ1	AA		5.1
46608	1.50E-06	10.70674	0.001067	0.016285	down	TIMM13	AD		1.2
46609	2.82E-09	21.8173	3.00E-06	3.07E-05	down	GADD45B	AT		4

46610	2.82E-09	21.8173	3.00E-06	3.07E-05	up	GADD45B	AT	3.2
46625	9.93E-07	16.55027	4.74E-05	0.010811	up	THOP1	ES	13
46695	1.46E-11	41.9773	9.23E-11	1.59E-07	up	MFSD12	AT	15
46697	1.46E-11	41.9773	9.23E-11	1.59E-07	down	MFSD12	AT	13.3
46707	1.16E-06	243.8095	5.81E-55	0.012663	up	TBXA2R	RI	3.2
46796	1.83E-12	39.05842	4.11E-10	2.00E-08	down	MPND	ES	11.2
46801	8.71E-08	29.357	6.02E-08	0.000948	up	UBXN6	AP	2
46802	8.71E-08	243.8095	5.81E-55	0.000948	down	UBXN6	AP	1
46808	1.82E-06	19.09297	1.25E-05	0.019853	down	PLIN5	AT	8
46809	1.81E-06	19.09297	1.25E-05	0.01967	up	PLIN5	AT	2.3
46831	5.53E-08	13.92052	0.000191	0.000603	up	KDM4B	AT	26
46832	5.53E-08	12.76986	0.000352	0.000603	down	KDM4B	AT	12
46952	5.83E-08	38.50037	5.47E-10	0.000634	down	NDUFA11	AT	4.3
46953	5.83E-08	32.47329	1.21E-08	0.000634	up	NDUFA11	AT	5
46954	5.42E-13	31.08	2.48E-08	5.90E-09	up	NDUFA11	RI	4.2
47038	5.64E-08	19.09297	1.25E-05	0.000614	up	SLC25A23	AT	11
47117	1.67E-13	30.68198	3.04E-08	1.81E-09	up	PET100	AT	3.2
47118	1.67E-13	31.482	2.01E-08	1.81E-09	down	PET100	AT	4
47226	1.89E-13	38.60865	5.18E-10	2.06E-09	down	RAB11B	AT	5
47227	1.89E-13	37.72214	8.16E-10	2.06E-09	up	RAB11B	AT	4.2
47440	1.93E-11	31.08	2.48E-08	2.10E-07	up	PIN1	AT	3.2
47441	1.92E-11	31.08	2.48E-08	2.09E-07	down	PIN1	AT	4.3
47450	7.31E-10	24.72324	6.62E-07	7.96E-06	down	C19orf66	AA	8.1
47481	1.27E-07	24.72324	6.62E-07	0.001385	down	MRPL4	AT	7.2
47482	1.27E-07	24.72324	6.62E-07	0.001385	up	MRPL4	AT	6.4
47485	6.84E-11	27.81081	1.34E-07	7.45E-07	up	MRPL4	RI	6.2
47514	2.71E-07	16.55027	4.74E-05	0.002951	up	CDC37	RI	4.2
47569	1.19E-06	24.72324	6.62E-07	0.012972	up	ILF3	AT	17.4
47570	1.19E-06	24.72324	6.62E-07	0.012972	down	ILF3	AT	20
47582	3.46E-17	34.53081	4.20E-09	3.76E-13	up	QTRT1	RI	6.2
47653	8.31E-07	15.97308	6.42E-05	0.009046	up	TMEM205	AP	1
47655	8.34E-07	17.14076	3.47E-05	0.009075	down	TMEM205	AP	2.1
47663	2.18E-06	12.00973	0.000529	0.023703	down	TMEM205	ES	2.6
47664	1.07E-06	16.26003	5.52E-05	0.011659	up	TMEM205	RI	2.3:2.4
47666	3.12E-12	34.53081	4.20E-09	3.40E-08	up	TMEM205	RI	2.3:2.4:2.5
47673	8.74E-13	38.16324	6.51E-10	9.51E-09	up	TMEM205	RI	2.2:2.3:2.4:[illegible]
47720	7.97E-10	21.48394	3.57E-06	8.67E-06	up	ECSIT	RI	6.2
47789	1.54E-06	11.76263	0.000604	0.016803	up	ZNF44	AT	18
47832	1.75E-08	27.81081	1.34E-07	0.000191	down	DHPS	ES	8
47871	1.09E-08	17.74487	2.53E-05	0.000119	down	PRDX2	ES	5
47884	1.32E-18	42.44441	7.27E-11	1.44E-14	down	GCDH	ME	4|5
47925	3.16E-08	21.8173	3.00E-06	0.000344	up	STX10	RI	7.2
47960	5.24E-08	21.8173	3.00E-06	0.00057	down	ASF1B	AT	4
47961	5.24E-08	21.8173	3.00E-06	0.00057	up	ASF1B	AT	3.3
48017	4.65E-11	31.08	2.48E-08	5.06E-07	down	ZNF333	AT	17
48018	8.04E-11	18.78117	1.47E-05	8.75E-07	up	ZNF333	AT	11
48068	1.90E-06	19.09297	1.25E-05	0.020645	up	BRD4	AT	14
48198	1.81E-07	19.09297	1.25E-05	0.001973	down	SMIM7	ES	5
48244	6.19E-13	45.97297	1.20E-11	6.74E-09	down	OCEL1	ES	4
48271	3.12E-08	31.08	2.48E-08	0.000339	down	MRPL34	AP	4.1
48272	3.39E-08	27.81081	1.34E-07	0.000369	up	MRPL34	AP	1
48446	1.79E-07	19.09297	1.25E-05	0.001953	down	FKBP8	AA	6.1
48448	1.29E-06	14.46107	0.000143	0.014062	down	FKBP8	ES	4:5:6.1:6.2
48450	3.47E-06	15.01465	0.000107	0.037811	down	FKBP8	ES	05:06.2
48483	2.20E-06	10.01189	0.001555	0.023947	down	UBA52	AD	1.3
48602	5.65E-11	27.81081	1.34E-07	6.15E-07	down	MEF2BNB	AT	6.2
48603	5.65E-11	27.81081	1.34E-07	6.15E-07	up	MEF2BNB	AT	4.2
48604	1.35E-08	34.53081	4.20E-09	0.000147	up	MEF2BNB	ES	5.2:6.1

48647	5.36E-08	14.31828	0.000154	0.000584	up	NDUFA13	AT	5
48649	1.56E-06	19.40821	1.06E-05	0.016996	down	NDUFA13	AT	4.4
48680	5.66E-10	22.15422	2.52E-06	6.17E-06	down	ZNF506	AT	5
48683	5.10E-08	14.18919	0.000165	0.000555	up	ZNF506	AT	8
48723	1.62E-06	16.55027	4.74E-05	0.017656	down	ZNF626	AT	4
48724	1.62E-06	16.55027	4.74E-05	0.017656	up	ZNF626	AT	5
48763	4.42E-06	12.00973	0.000529	0.048073	down	ZNF738	AT	4
48815	1.69E-09	27.81081	1.34E-07	1.84E-05	down	ZNF91	AT	4
48816	1.70E-09	27.81081	1.34E-07	1.85E-05	up	ZNF91	AT	5
48819	2.24E-07	14.18919	0.000165	0.002443	up	ZNF675	AT	10
48833	2.71E-09	38.16324	6.51E-10	2.95E-05	up	ZNF726	AT	7.2
48879	2.61E-08	21.8173	3.00E-06	0.000284	up	PDCD5	RI	5.4
48880	8.89E-08	16.55027	4.74E-05	0.000968	up	PDCD5	RI	5.2
48951	2.03E-09	38.16324	6.51E-10	2.21E-05	down	LSM14A	ES	11
49015	1.52E-08	24.01706	9.55E-07	0.000166	up	SCN1B	AT	3.3
49016	1.52E-08	25.08186	5.49E-07	0.000166	down	SCN1B	AT	6
49096	1.71E-07	243.8095	5.81E-55	0.001857	down	USF2	ES	04:05.1
49281	3.11E-15	37.28529	1.02E-09	3.38E-11	up	PSENEN	RI	3.2
49283	1.57E-07	19.09297	1.25E-05	0.001713	up	LIN37	RI	6.2
49433	2.90E-07	21.8173	3.00E-06	0.003163	down	ZNF568	AT	7
49449	3.95E-08	14.18919	0.000165	0.00043	down	ZNF585A	AT	7.6
49677	7.57E-07	13.92052	0.000191	0.008242	up	EIF3K	AT	7.2
49678	7.57E-07	15.29641	9.19E-05	0.008242	down	EIF3K	AT	8
49679	2.65E-10	21.03756	4.50E-06	2.88E-06	down	EIF3K	ES	6
49685	9.54E-08	16.84383	4.06E-05	0.001039	down	EIF3K	AD	1.2:1.3
49768	3.46E-13	34.11125	5.20E-09	3.77E-09	up	GMFG	RI	7.2
49829	3.81E-06	7.691425	0.005548	0.041535	up	RPS16	RI	3.2
49871	7.09E-09	31.08	2.48E-08	7.72E-05	up	AKT2	AT	14.2
49873	7.09E-09	31.08	2.48E-08	7.72E-05	down	AKT2	AT	15
49905	3.40E-12	37.72214	8.16E-10	3.71E-08	up	BLVRB	AD	3.2
50001	8.00E-11	27.81081	1.34E-07	8.71E-07	down	SNRPA	ES	3
50062	4.18E-06	16.55027	4.74E-05	0.045565	up	BCKDHA	AP	1
50063	4.18E-06	16.55027	4.74E-05	0.045565	down	BCKDHA	AP	2.1
50151	5.21E-12	38.16324	6.51E-10	5.67E-08	up	TMEM145	AT	16
50152	5.21E-12	38.16324	6.51E-10	5.67E-08	down	TMEM145	AT	14
50230	1.06E-12	24.72324	6.62E-07	1.15E-08	down	PLAUR	AT	8
50231	1.06E-12	24.72324	6.62E-07	1.15E-08	up	PLAUR	AT	9
50245	1.13E-08	24.72324	6.62E-07	0.000123	down	ZNF283	AT	8
50303	2.98E-08	16.55027	4.74E-05	0.000324	down	ZNF235	AT	7
50304	7.53E-07	10.01189	0.001555	0.008201	up	ZNF235	AT	6
50356	1.03E-06	24.72324	6.62E-07	0.011191	up	APOC1	AP	1
50357	1.03E-06	24.72324	6.62E-07	0.011191	down	APOC1	AP	2.1
50360	2.60E-13	40.88954	1.61E-10	2.83E-09	up	APOC1	AD	5.2:5.3
50371	7.38E-15	47.4537	5.63E-12	8.03E-11	down	APOC1	ES	2.2
50375	3.75E-11	45.4888	1.54E-11	4.08E-07	up	APOC2	RI	4.4
50376	1.57E-14	45.97297	1.20E-11	1.71E-10	up	APOC2	RI	4.3:4.4
50411	3.43E-09	21.8173	3.00E-06	3.73E-05	down	TRAPPC6A	ES	2
50413	5.45E-13	41.9773	9.23E-11	5.93E-09	up	TRAPPC6A	AD	1.2
50443	6.18E-07	31.08	2.48E-08	0.006727	down	ERCC1	ES	9
50444	3.35E-07	22.15422	2.52E-06	0.003649	down	ERCC1	ES	09:10.1
50450	4.76E-18	38.16324	6.51E-10	5.18E-14	down	FOSB	AT	3.3
50451	4.76E-18	38.16324	6.51E-10	5.18E-14	up	FOSB	AT	2.6
50488	1.35E-11	24.72324	6.62E-07	1.47E-07	down	OPA3	AT	4
50489	1.35E-11	24.72324	6.62E-07	1.47E-07	up	OPA3	AT	3
50529	5.66E-08	31.08	2.48E-08	0.000616	up	SYMPK	AP	1
50530	5.66E-08	31.08	2.48E-08	0.000616	down	SYMPK	AP	2.1
50576	5.86E-08	24.36833	7.96E-07	0.000638	up	DACT3	AT	4.2
50577	5.86E-08	25.08186	5.49E-07	0.000638	down	DACT3	AT	5

50659	3.11E-07	19.09297	1.25E-05	0.003388	down	NAPA	ES	3:04
50765	1.34E-10	50.15027	1.42E-12	1.46E-06	down	KDELR1	AP	2.1
50766	1.34E-10	50.15027	1.42E-12	1.46E-06	up	KDELR1	AP	1
50775	8.36E-13	26.69271	2.39E-07	9.10E-09	up	RPL18	RI	6.3
50814	4.47E-10	31.08	2.48E-08	4.87E-06	down	BCAT2	ES	3:04:05
50816	5.39E-14	45.97297	1.20E-11	5.87E-10	down	BCAT2	ES	3
50837	1.96E-06	9.563853	0.001984	0.021362	up	BAX	ES	5
50953	1.61E-06	25.94595	3.51E-07	0.017568	down	RPS11	ES	2.3:3.1
50967	4.35E-06	22.15422	2.52E-06	0.047371	down	FCGRT	ES	3:04:07
50975	2.07E-07	19.09297	1.25E-05	0.002254	up	NOSIP	AA	6.1
50996	1.58E-09	22.15422	2.52E-06	1.72E-05	down	IRF3	ES	5.1
51096	1.07E-12	30.68198	3.04E-08	1.16E-08	up	PTOV1	RI	13.2
51097	1.46E-11	31.08	2.48E-08	1.59E-07	up	PTOV1	RI	13.2:13.3
51108	2.28E-10	24.72324	6.62E-07	2.49E-06	up	AKT1S1	AP	3.1
51110	6.22E-07	21.8173	3.00E-06	0.006771	down	AKT1S1	AP	1.1
51112	5.43E-08	16.84383	4.06E-05	0.000592	down	AKT1S1	AD	1.2:1.3
51142	5.33E-08	24.72324	6.62E-07	0.00058	up	VRK3	AT	16.2
51203	1.78E-12	31.08	2.48E-08	1.94E-08	up	JOSD2	AP	1
51204	1.78E-12	31.08	2.48E-08	1.94E-08	down	JOSD2	AP	2
51205	3.25E-16	45.97297	1.20E-11	3.53E-12	down	JOSD2	ES	4
51467	7.16E-08	16.55027	4.74E-05	0.00078	down	ZNF808	AT	5.2
51468	7.16E-08	16.55027	4.74E-05	0.00078	up	ZNF808	AT	6
51780	3.15E-10	34.53081	4.20E-09	3.43E-06	up	NDUFA3	RI	4.2
51819	2.26E-08	21.48394	3.57E-06	0.000247	up	RPS9	AA	4.3:4.4
51820	1.95E-10	24.72324	6.62E-07	2.12E-06	up	RPS9	AD	4.2:4.3
51821	5.49E-12	31.08	2.48E-08	5.98E-08	up	RPS9	ES	4.3
51822	1.50E-07	13.65504	0.00022	0.001629	up	RPS9	AA	4.1:4.2:4.3:[illegible]
51823	2.84E-10	24.72324	6.62E-07	3.09E-06	up	RPS9	ES	4.1:4.3:4.4
51824	3.32E-13	34.53081	4.20E-09	3.62E-09	up	RPS9	ES	4.1:4.2:4.3
51825	1.12E-12	45.97297	1.20E-11	1.22E-08	up	RPS9	ES	4.1:4.3
51898	6.66E-12	24.72324	6.62E-07	7.25E-08	up	LENG8	RI	15.2:15.3:1[illegible]
51953	1.43E-06	14.18919	0.000165	0.015612	up	FCAR	AT	4.6
51954	1.43E-06	14.18919	0.000165	0.015612	down	FCAR	AT	5
52278	4.05E-06	21.8173	3.00E-06	0.044094	up	ZNF773	AT	6.3
52279	4.05E-06	21.8173	3.00E-06	0.044094	down	ZNF773	AT	4
52283	8.83E-07	21.8173	3.00E-06	0.009616	up	ZNF549	AT	5
52284	8.83E-07	21.8173	3.00E-06	0.009616	down	ZNF549	AT	4
52338	1.34E-06	14.18919	0.000165	0.014598	up	ZNF586	AT	6
52354	1.39E-07	24.72324	6.62E-07	0.001512	down	ZNF814	AT	9
52355	5.04E-09	27.81081	1.34E-07	5.49E-05	up	ZNF814	AT	4.2
52356	1.14E-06	16.26003	5.52E-05	0.012452	up	ZNF814	AT	8.3
52380	3.74E-06	12.00973	0.000529	0.040674	down	ZNF606	AT	3.2
52381	1.11E-13	41.9773	9.23E-11	1.21E-09	up	ZNF606	AT	4.2
52482	1.10E-06	19.09297	1.25E-05	0.011951	up	CHMP2A	AP	3.1
52520	1.24E-06	16.55027	4.74E-05	0.013549	up	TMEM18	AP	2
52521	1.24E-06	16.55027	4.74E-05	0.013506	down	TMEM18	AP	1
52562	3.87E-21	50.15027	1.42E-12	4.21E-17	down	ADI1	AP	1
52563	3.87E-21	50.15027	1.42E-12	4.21E-17	up	ADI1	AP	2
52570	1.81E-07	15.58155	7.90E-05	0.001967	down	RPS7	AT	7
52571	1.79E-07	13.65504	0.00022	0.001954	up	RPS7	AT	6.2
52618	7.57E-08	12.25994	0.000463	0.000824	down	ITGB1BP1	ES	8.1
52701	7.16E-09	27.81081	1.34E-07	7.79E-05	up	GREB1	AT	9.2
52702	4.65E-09	27.81081	1.34E-07	5.06E-05	down	GREB1	AT	38
52793	2.77E-18	50.19804	1.39E-12	3.01E-14	down	APOB	AT	30
52928	5.47E-08	28.19112	1.10E-07	0.000596	down	OST4	RI	1.2
52963	6.06E-07	21.8173	3.00E-06	0.006599	down	MPV17	AP	1
53234	1.77E-07	14.18919	0.000165	0.001927	down	RMDN2	AT	15
53380	8.49E-12	31.08	2.48E-08	9.24E-08	down	THADA	AT	41

53382	6.12E-11	34.53081	4.20E-09	6.66E-07	up	THADA	AT	40
53419	2.49E-09	34.53081	4.20E-09	2.71E-05	up	SLC3A1	AP	7.1
53420	1.31E-09	34.53081	4.20E-09	1.42E-05	down	SLC3A1	AP	1
53421	6.25E-14	31.08	2.48E-08	6.80E-10	up	SLC3A1	AT	9.2
53425	6.30E-13	38.16324	6.51E-10	6.86E-09	down	SLC3A1	AT	11
53563	1.67E-11	37.72214	8.16E-10	1.82E-07	up	ACYP2	AP	1
53565	7.89E-12	41.9773	9.23E-11	8.59E-08	down	ACYP2	AP	7
53566	2.55E-09	34.53081	4.20E-09	2.78E-05	down	ACYP2	AT	13
53569	2.00E-13	50.15027	1.42E-12	2.17E-09	up	ACYP2	AT	11
53681	1.35E-16	50.15027	1.42E-12	1.47E-12	down	PEX13	AT	5
53682	1.35E-16	50.15027	1.42E-12	1.47E-12	up	PEX13	AT	2.3
53777	1.69E-07	14.46107	0.000143	0.001845	down	CEP68	AT	7
53779	1.69E-07	13.92052	0.000191	0.001845	up	CEP68	AT	4.2
53824	6.76E-10	31.08	2.48E-08	7.36E-06	down	WDR92	AT	8
53825	6.73E-10	31.08	2.48E-08	7.33E-06	up	WDR92	AT	7.2
53844	1.64E-14	37.72214	8.16E-10	1.79E-10	up	ANTXR1	AT	13
53845	2.38E-14	38.16324	6.51E-10	2.59E-10	down	ANTXR1	AT	20
53868	1.78E-10	24.72324	6.62E-07	1.93E-06	down	TIA1	AT	15
53869	1.95E-10	24.72324	6.62E-07	2.12E-06	up	TIA1	AT	9.2
53881	4.17E-07	18.78117	1.47E-05	0.004544	up	SNRPG	AP	2.1
53883	4.05E-07	20.04919	7.55E-06	0.004413	down	SNRPG	AP	1
53928	4.31E-06	21.8173	3.00E-06	0.046931	up	ZNF638	AT	29
53929	4.27E-06	21.8173	3.00E-06	0.046513	down	ZNF638	AT	11
53946	1.86E-11	30.68198	3.04E-08	2.03E-07	up	SFXN5	AT	10.2
53947	1.86E-11	31.88804	1.63E-08	2.03E-07	down	SFXN5	AT	15
54003	1.14E-15	50.15027	1.42E-12	1.24E-11	down	DGUOK	ES	5:06
54015	8.65E-10	24.72324	6.62E-07	9.42E-06	down	DGUOK	ES	3
54101	3.85E-13	38.16324	6.51E-10	4.19E-09	up	AUP1	AA	11.1
54196	2.86E-07	16.55027	4.74E-05	0.003111	down	TGOLN2	RI	2.4
54282	3.87E-06	15.68938	7.46E-05	0.042148	up	GGCX	AT	6.2
54283	3.87E-06	14.73621	0.000124	0.042148	down	GGCX	AT	15
54293	5.00E-12	29.12869	6.77E-08	5.45E-08	up	RNF181	RI	4.2
54461	1.63E-18	45.97297	1.20E-11	1.78E-14	down	PLGLB1	AT	4.2
54462	8.13E-19	50.15027	1.42E-12	8.85E-15	up	PLGLB1	AT	6
55067	1.35E-08	24.72324	6.62E-07	0.000147	down	SLC35F5	AT	16.3
55068	1.35E-08	24.72324	6.62E-07	0.000147	up	SLC35F5	AT	7.2
55135	1.68E-09	31.08	2.48E-08	1.83E-05	up	TMEM177	AT	3
55136	1.68E-09	31.08	2.48E-08	1.83E-05	down	TMEM177	AT	2.2
55733	3.51E-06	19.40821	1.06E-05	0.03819	down	TANK	AT	14
55736	3.51E-06	18.78117	1.47E-05	0.03819	up	TANK	AT	7.2
56493	3.46E-10	38.16324	6.51E-10	3.77E-06	up	TFPI	AT	8
56494	3.46E-10	38.16324	6.51E-10	3.77E-06	down	TFPI	AT	10
56543	1.07E-07	12.00973	0.000529	0.001165	down	PMS1	AT	5.3
56544	1.07E-07	12.00973	0.000529	0.001165	up	PMS1	AT	14
56589	3.23E-07	24.72324	6.62E-07	0.003514	up	GLS	AT	20
56590	3.23E-07	24.72324	6.62E-07	0.003514	down	GLS	AT	16
56694	4.31E-06	12.00973	0.000529	0.046915	up	HSPE1	RI	3.2
56781	1.28E-08	21.15409	4.24E-06	0.000139	up	NDUFB3	ES	2.1:2.2
56794	1.23E-08	19.09297	1.25E-05	0.000133	down	CFLAR	AT	17
56795	1.02E-10	34.11125	5.20E-09	1.12E-06	up	CFLAR	AT	16
56799	3.48E-10	21.8173	3.00E-06	3.79E-06	up	CFLAR	ES	10
56930	4.27E-10	38.16324	6.51E-10	4.65E-06	down	SUMO1	RI	8.2
56931	1.92E-06	21.15409	4.24E-06	0.020939	up	SUMO1	ES	7
56965	1.08E-09	24.72324	6.62E-07	1.17E-05	down	CARF	AT	10.2
56967	1.07E-09	24.72324	6.62E-07	1.17E-05	up	CARF	AT	19
57074	7.49E-07	24.72324	6.62E-07	0.00815	up	RAPH1	AT	18
57075	1.82E-12	34.53081	4.20E-09	1.98E-08	down	RAPH1	AT	17
57077	2.34E-06	21.8173	3.00E-06	0.025456	up	RAPH1	AT	16

57260	8.07E-07	19.09297	1.25E-05	0.008782	down	KANSL1L	AT	16
57261	8.07E-07	19.09297	1.25E-05	0.008782	up	KANSL1L	AT	9.2
57365	5.10E-12	45.97297	1.20E-11	5.56E-08	up	FN1	RI	40.3
57624	1.92E-11	31.08	2.48E-08	2.09E-07	up	ZFAND2B	RI	4.1
57625	3.75E-11	27.81081	1.34E-07	4.09E-07	up	ZFAND2B	RI	4.8
57626	9.03E-08	12.00973	0.000529	0.000983	up	ZFAND2B	RI	4.6
57627	2.14E-11	27.81081	1.34E-07	2.33E-07	up	ZFAND2B	RI	4.4
57711	6.79E-11	27.81081	1.34E-07	7.39E-07	up	GMPPA	RI	9.2:9.3
57806	3.21E-06	24.72324	6.62E-07	0.034896	down	MFF	ES	8:09:10
57807	3.57E-07	31.08	2.48E-08	0.00389	down	MFF	ES	9:10
57998	8.21E-07	34.11125	5.20E-09	0.008938	up	EIF4E2	AT	6.2
58198	1.92E-06	14.18919	0.000165	0.020858	down	SCLY	AT	13
58199	1.92E-06	14.18919	0.000165	0.020858	up	SCLY	AT	9.2
58398	5.23E-16	45.97297	1.20E-11	5.69E-12	up	ATG4B	AD	14.2:14.3
58414	1.62E-11	31.08	2.48E-08	1.77E-07	up	D2HGDH	ES	10:11.1
58437	1.04E-13	31.08	2.48E-08	1.13E-09	up	C20orf96	AT	9.2
58438	1.04E-13	31.08	2.48E-08	1.13E-09	down	C20orf96	AT	11
58450	1.04E-06	16.55027	4.74E-05	0.011333	down	RBCK1	AT	3
58451	1.04E-06	16.55027	4.74E-05	0.011333	up	RBCK1	AT	13
58542	4.64E-08	31.08	2.48E-08	0.000505	down	SNRPB	AA	8.1
58546	4.43E-09	24.72324	6.62E-07	4.82E-05	up	ZNF343	AT	9
58547	4.43E-09	24.72324	6.62E-07	4.82E-05	down	ZNF343	AT	8
58672	9.63E-08	24.72324	6.62E-07	0.001049	down	PLCB1	AT	33
58673	9.63E-08	24.72324	6.62E-07	0.001049	up	PLCB1	AT	32.2
58744	8.37E-14	38.16324	6.51E-10	9.11E-10	down	SNX5	AT	13
58745	8.37E-14	38.16324	6.51E-10	9.11E-10	up	SNX5	AT	1.7
58855	3.17E-06	16.55027	4.74E-05	0.034565	up	APMAP	ES	8
58889	1.42E-08	24.72324	6.62E-07	0.000155	up	HM13	AD	12.2
58890	5.15E-08	28.19112	1.10E-07	0.000561	down	HM13	ES	12.1:12.2
58957	1.17E-06	18.78117	1.47E-05	0.012769	up	COMMD7	RI	9.2
59083	9.02E-15	45.97297	1.20E-11	9.82E-11	up	UQCC1	AT	10.2
59085	9.87E-12	38.16324	6.51E-10	1.07E-07	down	UQCC1	AT	13
59178	5.13E-08	16.26003	5.52E-05	0.000559	up	ERGIC3	ES	5
59183	4.36E-12	45.97297	1.20E-11	4.74E-08	up	CPNE1	AD	18.3
59184	2.31E-10	27.81081	1.34E-07	2.52E-06	up	CPNE1	AA	18.1
59212	1.30E-12	38.16324	6.51E-10	1.42E-08	up	NFS1	AT	6
59214	1.30E-12	38.16324	6.51E-10	1.42E-08	down	NFS1	AT	16
59219	3.93E-08	16.55027	4.74E-05	0.000428	up	NFS1	ES	7
59223	2.29E-07	19.09297	1.25E-05	0.002491	up	ROMO1	RI	1.3:1.4
59225	1.44E-09	24.72324	6.62E-07	1.57E-05	up	ROMO1	RI	1.2:1.3:1.4
59227	1.07E-06	24.36833	7.96E-07	0.011658	up	ROMO1	AD	1.2
59258	8.69E-07	24.72324	6.62E-07	0.009465	down	SCAND1	RI	3.2
59296	1.24E-06	14.18919	0.000165	0.013468	down	C20orf24	ES	03:04.1
59299	3.10E-07	38.16324	6.51E-10	0.003378	down	C20orf24	ES	3
59332	6.77E-12	38.60865	5.18E-10	7.38E-08	down	RPN2	ES	17
59527	2.01E-10	31.08	2.48E-08	2.19E-06	down	SYS1	AT	5
59528	2.01E-10	31.08	2.48E-08	2.19E-06	up	SYS1	AT	6
59616	7.25E-11	27.81081	1.34E-07	7.89E-07	up	SNX21	AT	5
59617	7.28E-11	27.81081	1.34E-07	7.93E-07	down	SNX21	AT	4.2
59620	8.59E-08	21.15409	4.24E-06	0.000935	up	ACOT8	AT	7
59621	8.59E-08	22.15422	2.52E-06	0.000935	down	ACOT8	AT	8
59624	1.19E-08	19.09297	1.25E-05	0.00013	up	ACOT8	AA	6.1
59771	8.23E-12	31.08	2.48E-08	8.97E-08	up	TMEM189	AT	8
59772	8.27E-12	31.08	2.48E-08	9.01E-08	down	TMEM189	AT	11
60005	8.83E-07	24.72324	6.62E-07	0.009616	up	GNAS	ES	6
60013	4.44E-10	18.94012	1.35E-05	4.83E-06	up	ATP5E	RI	2.2
60039	4.54E-07	21.8173	3.00E-06	0.004945	down	LSM14B	AT	10
60040	4.54E-07	21.8173	3.00E-06	0.004945	up	LSM14B	AT	4.2

60063	4.22E-06	243.8095	5.81E-55	0.045924	down	LAMA5	ES	76
60093	1.48E-15	45.4888	1.54E-11	1.61E-11	up	DIDO1	AT	16.2
60161	4.66E-25	50.15027	1.42E-12	5.07E-21	down	ZGPAT	AA	6.1
60188	2.27E-10	34.53081	4.20E-09	2.47E-06	up	TCEA2	RI	8.4
60226	1.11E-07	41.9773	9.23E-11	0.001213	down	CXADR	AT	7.2
60227	1.11E-07	41.9773	9.23E-11	0.001213	up	CXADR	AT	8
60262	8.94E-11	24.36833	7.96E-07	9.74E-07	up	ATP5J	ES	2
60265	5.50E-07	19.09297	1.25E-05	0.005992	up	ATP5J	ES	1.4:1.5:2
60374	1.09E-07	16.55027	4.74E-05	0.001189	down	PAXBP1	AT	19
60375	1.09E-07	16.55027	4.74E-05	0.001189	up	PAXBP1	AT	16
60391	2.00E-06	14.18919	0.000165	0.021828	up	IFNAR2	ES	09:10.1
60450	1.11E-08	34.53081	4.20E-09	0.000121	up	CRYZL1	AT	8
60466	5.37E-08	16.55027	4.74E-05	0.000584	up	ITSN1	AT	43
60467	5.88E-08	24.72324	6.62E-07	0.00064	up	ITSN1	AT	24
60468	1.24E-09	27.81081	1.34E-07	1.35E-05	down	ITSN1	AT	32
60488	1.43E-15	50.15027	1.42E-12	1.56E-11	up	RCAN1	AP	1
60491	1.88E-12	24.72324	6.62E-07	2.05E-08	up	RCAN1	AP	7.1
60494	2.46E-19	34.95451	3.37E-09	2.67E-15	down	RCAN1	AP	5
60526	2.86E-09	26.32744	2.88E-07	3.12E-05	up	CBR1	AA	3.1:3.2
60804	2.87E-08	34.53081	4.20E-09	0.000313	up	C21orf33	AT	6
60805	2.88E-08	34.53081	4.20E-09	0.000314	down	C21orf33	AT	8
60896	2.06E-12	27.81081	1.34E-07	2.24E-08	down	COL6A2	AT	28.2
60897	2.06E-12	27.81081	1.34E-07	2.24E-08	up	COL6A2	AT	29
61012	5.90E-08	25.08186	5.49E-07	0.000642	down	MICAL3	AT	39
61015	2.55E-08	21.8173	3.00E-06	0.000278	up	MICAL3	AT	25.2
61028	4.57E-11	38.16324	6.51E-10	4.98E-07	down	DGCR6	AT	7
61029	4.57E-11	38.16324	6.51E-10	4.98E-07	up	DGCR6	AT	3.3
61091	3.83E-15	41.9773	9.23E-11	4.17E-11	up	TXNRD2	RI	19.2
61099	6.04E-14	42.91607	5.71E-11	6.57E-10	down	COMT	AT	9
61100	6.04E-14	45.00923	1.96E-11	6.57E-10	up	COMT	AT	8.3
61101	1.91E-10	31.08	2.48E-08	2.08E-06	up	COMT	AA	8.1
61138	3.75E-07	12.00973	0.000529	0.004085	up	RANBP1	RI	7.2:7.3
61140	2.27E-08	19.09297	1.25E-05	0.000247	up	RANBP1	RI	7.2
61146	4.48E-09	24.72324	6.62E-07	4.88E-05	down	DGCR6L	ES	3
61261	1.62E-07	16.26003	5.52E-05	0.001766	up	PPM1F	AT	9
61262	1.62E-07	16.84383	4.06E-05	0.001766	down	PPM1F	AT	8.2
61266	1.05E-06	8.195676	0.004199	0.011476	up	TOP3B	RI	16.2
61358	1.59E-07	27.81081	1.34E-07	0.001736	down	DDT	AT	7
61359	1.59E-07	27.81081	1.34E-07	0.00173	up	DDT	AT	6.2
61403	1.62E-07	24.01706	9.55E-07	0.001769	up	UPB1	AT	5
61404	1.66E-07	22.15422	2.52E-06	0.001811	down	UPB1	AT	12
61419	1.99E-07	31.08	2.48E-08	0.002171	down	SNRPD3	AT	9
61665	1.02E-12	38.16324	6.51E-10	1.11E-08	up	UQCR10	AD	1.2
61736	1.10E-20	50.15027	1.42E-12	1.20E-16	up	SEC14L2	AT	11.2
61738	1.10E-20	50.15027	1.42E-12	1.19E-16	down	SEC14L2	AT	12
61813	1.23E-07	21.8173	3.00E-06	0.001337	up	SMTN	ES	21.1:21.2
62070	1.15E-09	24.72324	6.62E-07	1.25E-05	up	TST	RI	1.2
62152	2.38E-16	50.15027	1.42E-12	2.59E-12	down	TRIOBP	AT	26
62153	2.38E-16	50.15027	1.42E-12	2.59E-12	up	TRIOBP	AT	19
62155	3.84E-07	21.8173	3.00E-06	0.00418	down	GCAT	AT	9.2
62157	3.84E-07	21.8173	3.00E-06	0.00418	up	GCAT	AT	10
62178	3.50E-08	21.8173	3.00E-06	0.000381	up	POLR2F	AT	4.2
62231	1.57E-09	31.08	2.48E-08	1.71E-05	up	CSNK1E	AT	14.2
62232	1.26E-09	34.53081	4.20E-09	1.37E-05	down	CSNK1E	AT	18
62234	1.22E-07	10.01189	0.001555	0.001323	down	CSNK1E	AA	14.1
62346	3.04E-14	31.08	2.48E-08	3.31E-10	up	SGSM3	RI	19.2
62572	2.87E-08	24.72324	6.62E-07	0.000312	down	TSPO	ES	3
62665	4.52E-10	27.81081	1.34E-07	4.92E-06	up	FBLN1	AT	17

62666	1.13E-13	38.16324	6.51E-10	1.23E-09	down	FBLN1	AT	22
62760	3.12E-06	14.18919	0.000165	0.03396	down	CRELD2	ES	6
62795	9.52E-13	41.9773	9.23E-11	1.04E-08	up	HDAC10	RI	18.2
62912	4.09E-09	31.08	2.48E-08	4.45E-05	up	RABL2B	AT	8
62913	4.09E-09	31.08	2.48E-08	4.45E-05	down	RABL2B	AT	12.3
62991	5.77E-08	19.09297	1.25E-05	0.000628	up	SETMAR	AT	2.5
62992	5.81E-08	19.09297	1.25E-05	0.000632	down	SETMAR	AT	3
63113	5.89E-07	16.55027	4.74E-05	0.006411	down	MTMR14	ES	19:20
63260	4.11E-06	19.09297	1.25E-05	0.044786	down	IL17RC	ES	12
63304	2.13E-18	41.9773	9.23E-11	2.32E-14	down	FANCD2	AT	10.2
63305	1.10E-07	21.8173	3.00E-06	0.001201	down	FANCD2	AT	44.2
63307	1.30E-18	41.9773	9.23E-11	1.42E-14	up	FANCD2	AT	45
63545	4.18E-09	21.48394	3.57E-06	4.55E-05	up	MRPS25	AD	3.2
63637	5.53E-07	21.8173	3.00E-06	0.006019	up	OXNAD1	AT	12
63696	1.01E-06	10.01189	0.001555	0.010984	down	SGOL1	AT	8.2
63698	1.01E-06	10.01189	0.001555	0.010984	up	SGOL1	AT	9
63793	4.14E-07	16.55027	4.74E-05	0.004506	up	AZI2	AT	8.2
63794	4.14E-07	16.55027	4.74E-05	0.004506	down	AZI2	AT	10
64015	2.02E-21	45.97297	1.20E-11	2.20E-17	up	ACAA1	AT	9.4
64016	2.43E-21	46.95535	7.26E-12	2.65E-17	down	ACAA1	AT	12
64017	4.59E-09	16.55027	4.74E-05	5.00E-05	up	ACAA1	AD	9.2:9.3
64018	2.21E-17	50.15027	1.42E-12	2.41E-13	down	ACAA1	ES	9.3
64222	1.28E-07	19.09297	1.25E-05	0.001399	down	RPL14	AD	1.2
64250	1.86E-08	21.8173	3.00E-06	0.000202	down	CTNNB1	RI	18.2:18.3
64251	3.44E-11	27.81081	1.34E-07	3.74E-07	down	CTNNB1	AA	18.3
64268	1.16E-08	31.08	2.48E-08	0.000127	down	TRAK1	AT	17.2
64269	2.49E-10	31.08	2.48E-08	2.72E-06	up	TRAK1	AT	19
64270	1.82E-12	31.08	2.48E-08	1.98E-08	down	TRAK1	AT	16.2
64313	6.73E-16	45.97297	1.20E-11	7.33E-12	down	HIGD1A	AP	2
64314	6.73E-16	45.97297	1.20E-11	7.33E-12	up	HIGD1A	AP	1
64358	2.94E-09	34.53081	4.20E-09	3.20E-05	down	TCAIM	AT	13
64359	2.94E-09	34.53081	4.20E-09	3.20E-05	up	TCAIM	AT	5
64375	2.90E-14	41.9773	9.23E-11	3.16E-10	down	ZNF197	AT	8
64376	2.90E-14	41.9773	9.23E-11	3.16E-10	up	ZNF197	AT	7
64541	6.67E-07	14.46107	0.000143	0.007263	down	DHX30	AT	27
64542	7.80E-09	24.72324	6.62E-07	8.50E-05	up	DHX30	AT	13.2
64549	3.79E-22	51.69673	6.48E-13	4.12E-18	down	MAP4	AT	6
64730	4.02E-08	21.48394	3.57E-06	0.000437	up	NCKIPSD	AT	14
64731	4.02E-08	22.15422	2.52E-06	0.000437	down	NCKIPSD	AT	13
64850	2.35E-13	38.16324	6.51E-10	2.56E-09	down	USP4	AT	24
64852	1.31E-12	34.53081	4.20E-09	1.43E-08	up	USP4	AT	7
64936	4.99E-09	19.09297	1.25E-05	5.43E-05	up	RBM6	RI	14.2
65034	1.25E-11	21.8173	3.00E-06	1.36E-07	up	NPRL2	RI	8.2
65083	9.74E-13	41.05644	1.48E-10	1.06E-08	up	HEMK1	RI	9.2
65125	1.72E-14	41.9773	9.23E-11	1.87E-10	up	PCBP4	AA	15.1
65150	1.07E-11	36.25104	1.73E-09	1.17E-07	down	ACY1	ES	13:14
65165	3.27E-12	38.50427	5.46E-10	3.56E-08	down	RPL29	AT	4
65166	3.53E-12	35.88091	2.10E-09	3.84E-08	up	RPL29	AT	3.4
65169	8.48E-11	35.28872	2.84E-09	9.24E-07	down	RPL29	ES	3.3
65200	5.91E-16	45.97297	1.20E-11	6.43E-12	down	GLYCTK	RI	6.4
65201	2.80E-08	27.81081	1.34E-07	0.000305	down	GLYCTK	RI	6.2
65211	4.03E-12	47.95691	4.36E-12	4.38E-08	down	GLYCTK	ES	3
65231	3.46E-10	34.95451	3.37E-09	3.77E-06	down	SMIM4	AD	1.2
65273	3.03E-08	28.57531	9.01E-08	0.00033	down	ITIH3	ES	13.2:14:15:
65811	1.60E-07	25.08186	5.49E-07	0.001737	down	FILIP1L	AT	5
65812	1.63E-07	24.36833	7.96E-07	0.001772	up	FILIP1L	AT	7.5
65822	2.11E-08	24.72324	6.62E-07	0.000229	up	NIT2	AD	3.2
66125	3.15E-16	34.53081	4.20E-09	3.43E-12	down	GTPBP8	AT	9

66126	3.15E-16	34.53081	4.20E-09	3.43E-12	up	GTPBP8	AT	8.2
66373	2.87E-10	26.69271	2.39E-07	3.12E-06	up	NDUFB4	RI	2.2
66378	8.87E-07	21.15409	4.24E-06	0.00966	up	NDUFB4	AD	1.2
66433	2.31E-07	24.72324	6.62E-07	0.002512	up	FAM162A	AT	5.2
66434	2.30E-07	24.72324	6.62E-07	0.002504	down	FAM162A	AT	6
66460	8.75E-07	19.09297	1.25E-05	0.009522	down	PDIA5	ES	11
66522	5.16E-11	34.53081	4.20E-09	5.61E-07	up	KALRN	AT	35
66664	7.22E-11	30.68198	3.04E-08	7.86E-07	up	ACAD9	AT	13.2
66665	7.16E-11	28.19112	1.10E-07	7.79E-07	down	ACAD9	AT	18
66688	2.83E-08	19.09297	1.25E-05	0.000308	up	KIAA1257	AT	9
66689	2.83E-08	19.09297	1.25E-05	0.000308	down	KIAA1257	AT	8.2
66793	1.41E-06	21.8173	3.00E-06	0.015305	up	NUDT16	RI	3.2
66900	5.74E-09	27.81081	1.34E-07	6.25E-05	down	PCCB	AT	18.2
66901	5.74E-09	27.81081	1.34E-07	6.25E-05	up	PCCB	AT	19.2
66960	3.55E-08	24.72324	6.62E-07	0.000387	up	ARMC8	AT	23
66961	3.55E-08	24.72324	6.62E-07	0.000387	down	ARMC8	AT	13.2
67032	6.10E-10	45.97297	1.20E-11	6.65E-06	up	RBP1	AT	3
67033	1.11E-09	41.9773	9.23E-11	1.21E-05	down	RBP1	AT	6
67080	1.34E-07	16.55027	4.74E-05	0.001462	down	RNF7	AD	1.2
67110	4.31E-08	19.09297	1.25E-05	0.000469	up	XRN1	AT	12.2
67111	4.31E-08	19.09297	1.25E-05	0.000469	down	XRN1	AT	42
67213	3.27E-20	50.15027	1.42E-12	3.57E-16	down	CP	AT	19
67214	3.29E-20	50.15027	1.42E-12	3.59E-16	up	CP	AT	21
67598	4.01E-08	19.09297	1.25E-05	0.000437	down	SEC62	ES	2
67724	1.38E-09	24.72324	6.62E-07	1.50E-05	down	TTC14	AT	5.7
67725	1.38E-09	24.72324	6.62E-07	1.50E-05	up	TTC14	AT	6
67760	4.44E-16	45.97297	1.20E-11	4.83E-12	up	DNAJC19	ES	5.1:5.2
67769	6.32E-08	19.09297	1.25E-05	0.000688	down	MCCC1	AP	2
67770	3.98E-07	16.55027	4.74E-05	0.004332	up	MCCC1	AP	1
67804	4.42E-10	21.8173	3.00E-06	4.81E-06	up	KLHL24	AT	8.2
67805	4.42E-10	21.8173	3.00E-06	4.81E-06	down	KLHL24	AT	9
67978	5.66E-17	46.95535	7.26E-12	6.16E-13	down	EHHADH	AT	7
67979	5.66E-17	45.4888	1.54E-11	6.16E-13	up	EHHADH	AT	2.2
68054	2.43E-09	13.92052	0.000191	2.65E-05	up	EIF4A2	ES	11
68055	1.83E-06	27.43434	1.63E-07	0.019905	up	EIF4A2	AA	9.1
68105	8.47E-17	38.16324	6.51E-10	9.22E-13	down	IL1RAP	AT	10.2
68106	1.71E-22	45.97297	1.20E-11	1.86E-18	up	IL1RAP	AT	14
68107	9.57E-15	34.53081	4.20E-09	1.04E-10	up	IL1RAP	AT	13
68230	4.63E-11	31.08	2.48E-08	5.04E-07	up	PCYT1A	AT	11.2
68231	4.63E-11	31.08	2.48E-08	5.04E-07	down	PCYT1A	AT	12
68238	2.95E-06	12.00973	0.000529	0.032164	down	TM4SF19	AT	7
68240	1.45E-07	21.8173	3.00E-06	0.001576	up	TM4SF19	AT	5.2
68338	5.54E-08	12.00973	0.000529	0.000603	up	RPL35A	AD	3.2
68344	1.25E-17	41.9773	9.23E-11	1.36E-13	up	ZNF141	AT	5
68345	1.25E-17	41.9773	9.23E-11	1.36E-13	down	ZNF141	AT	4.2
68383	9.74E-10	21.8173	3.00E-06	1.06E-05	up	MYL5	AD	5.2
68436	8.93E-10	31.08	2.48E-08	9.72E-06	down	SLC26A1	AT	4
68437	8.96E-10	31.08	2.48E-08	9.76E-06	up	SLC26A1	AT	5
68526	3.26E-12	38.16324	6.51E-10	3.55E-08	down	WHSC1	AT	13.5
68527	3.26E-12	38.16324	6.51E-10	3.55E-08	up	WHSC1	AT	26
68852	2.81E-11	33.50929	7.09E-09	3.06E-07	down	QDPR	AT	8.2
68853	2.92E-11	29.12869	6.77E-08	3.17E-07	up	QDPR	AT	9
68856	3.30E-09	41.9773	9.23E-11	3.59E-05	down	QDPR	ES	6
68940	2.22E-06	16.84383	4.06E-05	0.02419	down	GPR125	AT	14
68941	3.67E-06	21.8173	3.00E-06	0.03999	up	GPR125	AT	23
68942	2.91E-06	16.84383	4.06E-05	0.031653	down	GPR125	AT	13.2
69000	5.98E-08	16.55027	4.74E-05	0.000651	up	C4orf19	AT	6
69001	5.98E-08	16.55027	4.74E-05	0.000651	down	C4orf19	AT	5

69133	5.78E-08	19.09297	1.25E-05	0.000629	down	TMEM33	RI	8.2
69234	7.17E-09	24.72324	6.62E-07	7.81E-05	up	OCIAD1	ES	5
69310	3.61E-17	45.97297	1.20E-11	3.93E-13	up	FIP1L1	AT	19
69311	3.23E-17	45.97297	1.20E-11	3.51E-13	down	FIP1L1	AT	31
69498	3.27E-08	12.00973	0.000529	0.000356	up	MTHFD2L	AT	12
69528	4.02E-08	14.18919	0.000165	0.000437	up	THAP6	AT	8
69530	2.44E-11	24.72324	6.62E-07	2.66E-07	down	THAP6	AT	5
69531	2.75E-06	21.8173	3.00E-06	0.029977	up	THAP6	AT	7.3
69561	5.29E-14	45.97297	1.20E-11	5.76E-10	down	NAAA	AT	12
69562	6.09E-22	50.15027	1.42E-12	6.63E-18	up	NAAA	AT	8.2
69563	6.47E-08	31.08	2.48E-08	0.000705	up	NAAA	AT	3.2
69564	2.44E-12	41.9773	9.23E-11	2.65E-08	up	NAAA	AT	10
69616	4.34E-08	27.81081	1.34E-07	0.000472	up	11-Sep	AT	12.3
69618	9.79E-12	34.53081	4.20E-09	1.07E-07	down	11-Sep	AT	11.2
69694	4.44E-20	51.17628	8.44E-13	4.84E-16	down	RASGEF1B	AT	15
69695	4.44E-20	49.64457	1.84E-12	4.84E-16	up	RASGEF1B	AT	5.2
69705	1.19E-09	30.68198	3.04E-08	1.30E-05	up	HNRNPDL	ES	8
69715	4.05E-08	24.72324	6.62E-07	0.00044	up	SCD5	AT	6
69716	1.52E-09	34.53081	4.20E-09	1.66E-05	down	SCD5	AT	8
69735	3.85E-06	11.76263	0.000604	0.041892	up	SEC31A	AD	10.2
69905	1.19E-12	38.16324	6.51E-10	1.30E-08	up	FAM13A	AT	4.2
69906	6.14E-17	50.66081	1.10E-12	6.69E-13	down	FAM13A	AT	26
69909	2.01E-15	49.64457	1.84E-12	2.19E-11	up	FAM13A	AT	5.2
69971	3.98E-09	21.8173	3.00E-06	4.34E-05	down	PDLIM5	AT	25.3
69973	2.77E-16	37.72214	8.16E-10	3.01E-12	up	PDLIM5	AT	4
70298	5.25E-13	45.97297	1.20E-11	5.72E-09	up	RPL34	AT	7
70300	5.25E-13	45.97297	1.20E-11	5.72E-09	down	RPL34	AT	8
70445	2.53E-11	34.53081	4.20E-09	2.75E-07	up	SEC24D	ES	27
70504	2.53E-08	24.72324	6.62E-07	0.000275	up	EXOSC9	AD	10.2:10.3
71135	1.36E-06	27.81081	1.34E-07	0.014755	up	CBR4	AT	9
71136	2.04E-08	21.8173	3.00E-06	0.000222	up	CBR4	AT	5.2
71137	3.64E-15	45.97297	1.20E-11	3.96E-11	down	CBR4	AT	6
71416	2.39E-06	19.09297	1.25E-05	0.026004	up	FRG1	ES	2
71419	1.58E-07	24.72324	6.62E-07	0.001718	down	SDHA	ES	13:14
71654	6.39E-07	13.65504	0.00022	0.006959	up	SUB1	AP	1
71655	6.39E-07	15.58155	7.90E-05	0.006959	down	SUB1	AP	3
71658	3.56E-07	13.13347	0.00029	0.003872	up	SUB1	ES	4.1:4.2
71901	3.26E-14	41.9773	9.23E-11	3.55E-10	up	SEPP1	AP	2
71902	3.26E-14	41.9773	9.23E-11	3.55E-10	down	SEPP1	AP	1
71916	6.44E-10	46.4618	9.34E-12	7.01E-06	down	SEPP1	ES	3
71946	2.30E-12	41.9773	9.23E-11	2.50E-08	up	CCL28	AT	6
71947	2.30E-12	41.9773	9.23E-11	2.50E-08	down	CCL28	AT	5.2
72190	8.00E-10	38.16324	6.51E-10	8.71E-06	down	IPO11	AT	32
72191	8.00E-10	38.16324	6.51E-10	8.71E-06	up	IPO11	AT	35
72280	1.99E-10	31.08	2.48E-08	2.17E-06	up	MAST4	AT	19.2
72386	6.98E-07	14.18919	0.000165	0.007597	up	GTF2H2C	AT	9
72681	6.82E-08	21.8173	3.00E-06	0.000743	down	ATG10	AT	5
72692	7.16E-07	16.26003	5.52E-05	0.007792	up	RPS23	RI	3.3
72693	3.42E-08	16.55027	4.74E-05	0.000372	up	RPS23	RI	3.2:3.3:3.4:
72713	1.05E-07	14.18919	0.000165	0.001147	up	COX7C	RI	2.3
72715	7.60E-07	14.5863	0.000134	0.008273	up	COX7C	AD	2.2
72865	6.80E-08	27.81081	1.34E-07	0.00074	up	ERAP1	AT	19.2
72866	6.80E-08	27.81081	1.34E-07	0.00074	down	ERAP1	AT	20
73217	6.37E-08	13.39269	0.000253	0.000694	up	HINT1	ES	2.3:3.1:3.2
73218	3.56E-14	41.9773	9.23E-11	3.88E-10	down	HINT1	ES	3.2
73299	3.64E-08	24.72324	6.62E-07	0.000396	up	8-Sep	AT	12.3
73300	2.40E-08	31.08	2.48E-08	0.000261	down	8-Sep	AT	14
73412	1.42E-07	24.72324	6.62E-07	0.001543	up	SAR1B	AP	7.1

73415	2.88E-08	21.8173	3.00E-06	0.000314	down	SAR1B	AP	2
73420	3.52E-10	27.81081	1.34E-07	3.83E-06	up	SEC24A	AT	13
73421	3.52E-10	27.81081	1.34E-07	3.83E-06	down	SEC24A	AT	24
73565	2.12E-09	21.8173	3.00E-06	2.31E-05	up	CTNNA1	ES	20
73784	1.23E-15	41.9773	9.23E-11	1.33E-11	down	PCDHA9	AT	4
73785	1.23E-15	41.9773	9.23E-11	1.33E-11	up	PCDHA9	AT	1.2
74077	2.63E-06	21.8173	3.00E-06	0.028666	down	CD74	ES	8
74080	5.96E-10	28.19112	1.10E-07	6.49E-06	down	CD74	ES	6:7.1:8:9:1(
74081	2.17E-06	16.55027	4.74E-05	0.023573	down	CD74	ES	6:7.1:9:10.1
74090	1.20E-07	19.45756	1.03E-05	0.001301	up	RPS14	ES	6
74095	3.01E-11	30.2879	3.72E-08	3.28E-07	up	RPS14	AD	1.2:1.3
74096	1.27E-12	29.89768	4.55E-08	1.39E-08	up	RPS14	AD	1.2
74148	1.38E-09	41.9773	9.23E-11	1.51E-05	up	ANXA6	ES	22
74179	3.31E-11	34.7501	3.75E-09	3.61E-07	up	ATOX1	RI	6.2
74212	1.05E-08	21.8173	3.00E-06	0.000114	up	GALNT10	AT	15
74213	1.05E-08	21.8173	3.00E-06	0.000114	down	GALNT10	AT	8.2
74297	4.65E-08	24.72324	6.62E-07	0.000506	down	MRPL22	ES	2.2
74408	2.52E-06	24.72324	6.62E-07	0.027472	down	PWWP2A	AT	2.2
74409	2.52E-06	24.72324	6.62E-07	0.027388	up	PWWP2A	AT	4
74519	1.67E-08	34.53081	4.20E-09	0.000182	down	NPM1	AT	13
74520	1.69E-08	34.53081	4.20E-09	0.000183	up	NPM1	AT	11
74560	1.04E-06	19.09297	1.25E-05	0.011345	up	ERGIC1	AT	6.4
74575	5.72E-12	27.81081	1.34E-07	6.22E-08	down	CREBRF	AT	10
74576	5.72E-12	27.81081	1.34E-07	6.22E-08	up	CREBRF	AT	4.2
74698	2.11E-12	45.97297	1.20E-11	2.30E-08	up	ZNF346	AT	8.2
74699	2.11E-12	45.97297	1.20E-11	2.30E-08	down	ZNF346	AT	9
74762	8.14E-08	16.55027	4.74E-05	0.000886	up	GRK6	AT	13.2
74855	6.41E-18	46.95535	7.26E-12	6.98E-14	down	PHYKPL	ES	14
74891	8.94E-13	38.16324	6.51E-10	9.73E-09	down	ADAMTS2	AT	11
74892	8.94E-13	38.16324	6.51E-10	9.73E-09	up	ADAMTS2	AT	23
74933	1.26E-08	24.72324	6.62E-07	0.000137	down	SQSTM1	AP	1
74948	8.18E-16	41.9773	9.23E-11	8.91E-12	up	C5orf45	RI	7.2
74993	4.62E-13	41.51466	1.17E-10	5.03E-09	up	RNF130	AT	10
74994	4.62E-13	42.91607	5.71E-11	5.03E-09	down	RNF130	AT	9
75015	5.94E-10	34.53081	4.20E-09	6.47E-06	up	FLT4	AT	30.2
75016	5.94E-10	34.53081	4.20E-09	6.47E-06	down	FLT4	AT	31
75046	5.33E-07	14.18919	0.000165	0.005799	down	TRIM7	AT	8
75047	5.33E-07	14.18919	0.000165	0.005799	up	TRIM7	AT	3.3
75050	3.67E-09	27.43434	1.63E-07	3.99E-05	up	TRIM41	RI	6.4
75051	1.37E-07	27.43434	1.63E-07	0.001492	up	TRIM41	RI	6.2
75065	2.34E-06	11.16774	0.000832	0.025493	up	GNB2L1	AD	2.4
75124	1.97E-07	19.09297	1.25E-05	0.002141	up	GNB2L1	AD	1.2
75240	2.37E-07	24.72324	6.62E-07	0.002576	down	LYRM4	AT	3
75241	1.61E-07	31.08	2.48E-08	0.001754	up	LYRM4	AT	7
75285	3.77E-06	16.55027	4.74E-05	0.041	up	SLC35B3	AT	4.2
75356	1.48E-12	24.72324	6.62E-07	1.61E-08	up	HIVEP1	AT	3
75357	1.48E-12	24.72324	6.62E-07	1.61E-08	down	HIVEP1	AT	10
75387	1.97E-09	27.81081	1.34E-07	2.14E-05	down	GFOD1	AT	2
75388	1.97E-09	27.81081	1.34E-07	2.14E-05	up	GFOD1	AT	4
75529	9.48E-08	27.81081	1.34E-07	0.001033	up	ACOT13	ES	2
75605	7.46E-10	31.08	2.48E-08	8.12E-06	up	HIST1H2A(	AD	1.2
76000	4.46E-16	50.15027	1.42E-12	4.86E-12	up	MTCH1	AD	8.2
76139	8.75E-09	31.08	2.48E-08	9.53E-05	down	PRICKLE4	AA	10.1
76166	5.95E-07	21.8173	3.00E-06	0.006473	up	TAF8	AT	6.2
76167	8.89E-10	34.53081	4.20E-09	9.68E-06	down	TAF8	AT	11
76181	7.39E-07	14.73621	0.000124	0.008046	down	UBR2	AT	51
76182	7.39E-07	13.92052	0.000191	0.008046	up	UBR2	AT	14
76222	1.02E-08	21.98346	2.75E-06	0.000112	up	KLC4	AT	10.2

76223	1.02E-08	20.04919	7.55E-06	0.000111	down	KLC4	AT	20
76245	4.77E-11	31.08	2.48E-08	5.19E-07	down	PTK7	AT	8.2
76246	1.73E-10	31.08	2.48E-08	1.89E-06	up	PTK7	AT	21
76257	2.86E-14	37.72214	8.16E-10	3.11E-10	up	DNPH1	RI	3.2
76284	1.83E-07	11.51863	0.000689	0.001997	up	YIPF3	RI	4.8
76285	7.84E-08	13.92052	0.000191	0.000853	up	YIPF3	RI	4.6
76317	7.61E-07	19.09297	1.25E-05	0.008291	down	MRPS18A	ES	5
76510	1.10E-08	31.08	2.48E-08	0.00012	down	LRRC1	AT	12.2
76557	1.29E-06	19.09297	1.25E-05	0.014025	down	DST	AT	108
76558	6.11E-07	14.18919	0.000165	0.006654	up	DST	AT	37.2
76814	1.21E-08	34.53081	4.20E-09	0.000131	up	HMGN3	AD	5.2
77075	9.84E-10	19.09297	1.25E-05	1.07E-05	down	CCNC	AT	14
77077	8.17E-14	31.08	2.48E-08	8.90E-10	up	CCNC	AT	13.2
77342	1.75E-11	27.81081	1.34E-07	1.90E-07	up	DCBLD1	AT	15
77343	1.75E-11	27.81081	1.34E-07	1.90E-07	down	DCBLD1	AT	16
77354	2.35E-15	41.9773	9.23E-11	2.56E-11	up	MCM9	AT	7.2
77355	2.35E-15	41.9773	9.23E-11	2.56E-11	down	MCM9	AT	13
77774	2.18E-13	34.7501	3.75E-09	2.38E-09	up	SGK1	AT	2.2
77775	2.15E-13	40.43456	2.03E-10	2.35E-09	down	SGK1	AT	16
77890	4.27E-06	14.18919	0.000165	0.046489	down	AHI1	AT	27
77970	9.77E-09	27.43434	1.63E-07	0.000106	up	AIG1	AT	2
77972	1.55E-08	45.97297	1.20E-11	0.000169	down	AIG1	AT	10
77973	1.85E-07	31.08	2.48E-08	0.002012	down	AIG1	AT	5.2
77979	2.30E-08	16.55027	4.74E-05	0.000251	down	FUCA2	AT	7
77980	2.30E-08	16.55027	4.74E-05	0.000251	up	FUCA2	AT	6.2
78095	1.60E-06	24.72324	6.62E-07	0.017439	down	NUP43	AT	7.2
78096	1.60E-06	24.72324	6.62E-07	0.017439	up	NUP43	AT	8.3
78256	2.61E-08	18.78117	1.47E-05	0.000284	up	SERAC1	AT	17.2
78273	2.42E-07	14.18919	0.000165	0.002636	down	DYNLT1	AP	2.1
78274	2.42E-07	14.18919	0.000165	0.002636	up	DYNLT1	AP	1
78301	7.36E-08	19.09297	1.25E-05	0.000801	down	SOD2	AT	5.3
78303	7.36E-08	19.09297	1.25E-05	0.000801	up	SOD2	AT	8.3
78498	3.17E-15	38.16324	6.51E-10	3.46E-11	down	PDCD2	AT	7
78499	2.29E-14	45.97297	1.20E-11	2.49E-10	up	PDCD2	AT	3.3
78556	1.78E-17	45.97297	1.20E-11	1.94E-13	down	COX19	AT	13
78557	1.78E-17	45.97297	1.20E-11	1.94E-13	up	COX19	AT	3
78589	3.03E-09	31.08	2.48E-08	3.30E-05	down	PSMG3	AP	1
78655	2.88E-10	34.53081	4.20E-09	3.14E-06	up	WIPI2	AD	12.2
78662	1.94E-08	16.55027	4.74E-05	0.000211	up	TNRC18	AT	31
78663	1.93E-08	16.55027	4.74E-05	0.00021	down	TNRC18	AT	5.2
78666	3.52E-09	21.8173	3.00E-06	3.83E-05	down	FBXL18	AT	5
78667	4.52E-06	16.26003	5.52E-05	0.049229	up	FBXL18	AT	6
78800	9.00E-10	24.36833	7.96E-07	9.80E-06	up	PHF14	AT	19
78801	9.03E-10	25.44421	4.55E-07	9.83E-06	down	PHF14	AT	17
79038	1.19E-07	24.72324	6.62E-07	0.001298	up	HNRNPA2	AA	12.1
79040	7.58E-20	45.97297	1.20E-11	8.26E-16	down	CBX3	AP	1
79041	7.58E-20	45.97297	1.20E-11	8.26E-16	up	CBX3	AP	2
79121	1.86E-06	15.97308	6.42E-05	0.020242	up	GGCT	AP	1
79122	1.86E-06	14.73621	0.000124	0.020242	down	GGCT	AP	3
79246	3.72E-08	24.01706	9.55E-07	0.000405	up	KIAA0895	AT	4.2
79247	3.72E-08	26.18036	3.11E-07	0.000405	down	KIAA0895	AT	9
79318	4.22E-18	50.15027	1.42E-12	4.59E-14	down	PSMA2	AT	8.2
79352	9.18E-09	34.53081	4.20E-09	1.00E-04	up	MRPS24	RI	1.2
79354	3.93E-07	19.09297	1.25E-05	0.004276	down	URGCP	AP	3
79570	2.44E-11	41.9773	9.23E-11	2.66E-07	up	H2AFV	AT	5
79572	1.11E-11	41.9773	9.23E-11	1.21E-07	down	H2AFV	AT	6
79576	1.36E-12	34.53081	4.20E-09	1.48E-08	up	H2AFV	ES	2
79639	5.75E-10	27.81081	1.34E-07	6.27E-06	down	UPP1	ES	5:6.1:6.2:7

79736	8.92E-07	13.02964	0.000307	0.009707	down	SEC61G	AD	2.2
79743	2.86E-11	31.08	2.48E-08	3.11E-07	up	EGFR	AT	33
79853	3.10E-07	19.09297	1.25E-05	0.003374	down	GUSB	AT	12
79854	3.14E-07	19.09297	1.25E-05	0.003418	up	GUSB	AT	5.2
79866	3.04E-09	31.482	2.01E-08	3.31E-05	down	ASL	ES	13
79867	7.61E-09	27.81081	1.34E-07	8.29E-05	down	ASL	ES	8
79927	1.03E-21	50.15027	1.42E-12	1.12E-17	down	POM121	AT	17
79928	1.03E-21	50.15027	1.42E-12	1.12E-17	up	POM121	AT	18
79935	8.96E-10	16.26003	5.52E-05	9.76E-06	up	NSUN5	RI	9.2
80000	8.62E-08	21.8173	3.00E-06	0.000938	up	WBSCR22	AT	11
80001	1.31E-06	27.81081	1.34E-07	0.014246	down	WBSCR22	AT	14
80002	1.17E-12	33.69577	6.44E-09	1.27E-08	up	WBSCR22	ES	12
80093	6.08E-12	37.72214	8.16E-10	6.62E-08	up	GTF2IRD2	AT	16
80104	1.91E-13	34.53081	4.20E-09	2.08E-09	up	GTF2IRD2E	AT	16
80105	1.91E-13	34.53081	4.20E-09	2.08E-09	down	GTF2IRD2E	AT	17
80137	2.44E-09	27.06166	1.97E-07	2.65E-05	up	POR	RI	16.3:16.4
80207	1.63E-06	14.18919	0.000165	0.017799	up	PHTF2	AT	14.2
80356	4.96E-07	24.36833	7.96E-07	0.005403	up	SRI	AT	9.2
80357	4.96E-07	25.08186	5.49E-07	0.005403	down	SRI	AT	10
80361	1.08E-18	50.15027	1.42E-12	1.18E-14	down	STEAP4	AT	6
80362	1.08E-18	50.15027	1.42E-12	1.17E-14	up	STEAP4	AT	4.2
80441	6.06E-07	12.00973	0.000529	0.006597	up	RBM48	RI	4.2
80645	6.92E-16	45.97297	1.20E-11	7.53E-12	up	ATP5J2	AD	1.2
80902	2.46E-12	34.53081	4.20E-09	2.67E-08	up	LAMTOR4	RI	3.2
80979	4.93E-15	49.64457	1.84E-12	5.37E-11	up	TFR2	ES	10
81052	6.65E-13	41.9773	9.23E-11	7.24E-09	up	FIS1	AP	1
81053	6.65E-13	41.9773	9.23E-11	7.24E-09	down	FIS1	AP	2.1
81054	2.24E-12	41.9773	9.23E-11	2.44E-08	down	FIS1	ES	3
81177	1.83E-18	50.15027	1.42E-12	2.00E-14	up	PMPCB	RI	12.2
81180	1.23E-11	32.29817	1.32E-08	1.34E-07	down	PMPCB	ES	7
81267	8.38E-10	21.8173	3.00E-06	9.13E-06	down	KMT2E	AT	28.3
81268	8.38E-10	21.8173	3.00E-06	9.13E-06	up	KMT2E	AT	16.2
81357	2.17E-08	14.18919	0.000165	0.000237	down	BCAP29	AT	11
81747	2.06E-09	24.72324	6.62E-07	2.24E-05	up	STRIP2	AT	20.2
81748	2.06E-09	24.72324	6.62E-07	2.24E-05	down	STRIP2	AT	21
81886	1.95E-10	35.38241	2.71E-09	2.12E-06	down	CNOT4	AT	11
82016	2.88E-09	25.96581	3.48E-07	3.13E-05	up	NDUFB2	AP	4
82017	9.21E-20	45.97297	1.20E-11	1.00E-15	down	NDUFB2	AP	3
82018	1.64E-18	45.97297	1.20E-11	1.79E-14	up	NDUFB2	AP	6
82019	1.06E-09	23.66939	1.14E-06	1.16E-05	up	NDUFB2	AP	5
82026	1.02E-06	16.26003	5.52E-05	0.01106	up	NDUFB2	ES	7
82027	2.44E-09	34.53081	4.20E-09	2.66E-05	up	MRPS33	AP	2
82030	1.08E-09	41.9773	9.23E-11	1.18E-05	down	MRPS33	AP	3
82055	2.80E-15	34.53081	4.20E-09	3.05E-11	up	SSBP1	AD	1.2
82079	3.07E-06	10.64006	0.001107	0.033452	down	GSTK1	ES	6
82081	3.64E-09	28.96344	7.38E-08	3.96E-05	down	GSTK1	ES	4.3
82082	1.21E-18	41.9773	9.23E-11	1.32E-14	up	GSTK1	RI	4.2
82182	5.21E-13	41.9773	9.23E-11	5.67E-09	up	ZNF783	AT	16
82183	5.21E-13	41.9773	9.23E-11	5.67E-09	down	ZNF783	AT	7
82226	3.09E-13	21.8173	3.00E-06	3.36E-09	up	RARRES2	RI	1.3
82256	4.17E-06	19.09297	1.25E-05	0.045427	down	TMEM176I	AP	2.1
82257	5.55E-15	41.9773	9.23E-11	6.04E-11	up	TMEM176I	AP	4.1
82258	7.24E-07	21.8173	3.00E-06	0.007886	down	TMEM176I	ES	5
82259	9.43E-11	36.85262	1.27E-09	1.03E-06	up	TMEM176I	ES	2.2:4.2
82332	3.39E-09	16.55027	4.74E-05	3.69E-05	up	FASTK	RI	5.1
82333	4.55E-12	27.81081	1.34E-07	4.96E-08	up	FASTK	RI	5.8
82334	4.22E-13	27.81081	1.34E-07	4.59E-09	up	FASTK	RI	5.6
82335	5.45E-12	38.16324	6.51E-10	5.93E-08	up	FASTK	RI	5.4

82336	4.45E-10	24.72324	6.62E-07	4.85E-06	up	FASTK	RI	5.2
82337	9.29E-07	12.00973	0.000529	0.010114	down	FASTK	AD	3.2
82508	2.28E-11	31.08	2.48E-08	2.49E-07	up	DNAJB6	AT	11
82510	2.28E-11	31.08	2.48E-08	2.49E-07	down	DNAJB6	AT	9.2
82574	1.41E-07	16.55027	4.74E-05	0.001533	down	MCPH1	AT	8.2
82575	1.41E-07	16.55027	4.74E-05	0.001533	up	MCPH1	AT	14
82767	1.02E-08	27.43434	1.63E-07	0.000111	up	DLC1	AT	5.2
82778	3.39E-08	16.55027	4.74E-05	0.000369	up	MSR1	AT	10.2
82779	3.39E-08	16.55027	4.74E-05	0.000369	down	MSR1	AT	12
83007	4.56E-16	44.35894	2.73E-11	4.96E-12	down	SLC39A14	AT	11
83008	4.56E-16	44.06361	3.18E-11	4.96E-12	up	SLC39A14	AT	12
83043	5.10E-07	21.8173	3.00E-06	0.00555	down	BIN3	AT	11
83045	1.23E-06	21.8173	3.00E-06	0.013396	up	BIN3	AT	8
83135	4.12E-17	41.9773	9.23E-11	4.49E-13	up	ADRA1A	AT	8
83136	4.92E-13	34.53081	4.20E-09	5.35E-09	down	ADRA1A	AT	6
83139	1.65E-07	31.89318	1.63E-08	0.001796	down	ADRA1A	AT	4
83140	4.33E-07	27.81081	1.34E-07	0.004709	down	ADRA1A	AT	7.2
83154	8.27E-13	34.53081	4.20E-09	9.01E-09	up	PTK2B	AT	31.2
83155	8.27E-13	34.53081	4.20E-09	9.01E-09	down	PTK2B	AT	35
83168	9.87E-20	50.15027	1.42E-12	1.07E-15	up	CLU	AP	1
83170	3.33E-20	50.15027	1.42E-12	3.63E-16	down	CLU	AP	3.1
83284	3.69E-08	21.8173	3.00E-06	0.000402	up	DCTN6	ES	3
83387	7.03E-13	37.72214	8.16E-10	7.65E-09	up	PPAPDC1E	AT	4.2
83388	1.22E-12	38.16324	6.51E-10	1.33E-08	down	PPAPDC1E	AT	7
83515	4.10E-07	19.09297	1.25E-05	0.004462	up	GINS4	AT	6.3
83516	4.10E-07	19.09297	1.25E-05	0.004462	down	GINS4	AT	3.2
83887	8.60E-07	16.69146	4.40E-05	0.009369	up	RPS20	RI	2.2
83911	1.11E-06	12.00973	0.000529	0.012067	down	CHCHD7	AA	5.1
83968	5.73E-14	38.16324	6.51E-10	6.24E-10	up	ASPH	AT	22
83969	3.74E-13	41.9773	9.23E-11	4.08E-09	up	ASPH	AT	20.3
83970	6.46E-14	38.16324	6.51E-10	7.03E-10	down	ASPH	AT	33
84058	1.15E-07	16.55027	4.74E-05	0.00125	up	COPS5	ES	10
84147	4.30E-11	22.98466	1.63E-06	4.69E-07	up	RPL7	RI	7.2
84155	1.87E-10	38.16324	6.51E-10	2.04E-06	down	STAU2	AT	18.2
84156	4.25E-08	24.72324	6.62E-07	0.000463	down	STAU2	AT	10
84157	1.17E-11	41.9773	9.23E-11	1.27E-07	up	STAU2	AT	21
84200	1.22E-06	21.8173	3.00E-06	0.0133	up	TCEB1	AT	8
84201	1.22E-06	21.8173	3.00E-06	0.0133	down	TCEB1	AT	9
84264	3.42E-06	16.55027	4.74E-05	0.037275	down	MRPS28	AP	1
84265	3.42E-06	16.26003	5.52E-05	0.037275	up	MRPS28	AP	2
84266	4.23E-08	22.15422	2.52E-06	0.000461	down	MRPS28	ES	4.1:4.2
84270	9.28E-10	31.08	2.48E-08	1.01E-05	up	TPD52	AT	12
84271	9.28E-10	31.08	2.48E-08	1.01E-05	down	TPD52	AT	14
84329	3.48E-07	16.55027	4.74E-05	0.003789	up	C8orf59	AP	3.1
84330	3.47E-07	16.55027	4.74E-05	0.003776	down	C8orf59	AP	1
84338	2.09E-07	38.16324	6.51E-10	0.00227	up	C8orf59	ES	2.1:2.2
84339	1.80E-06	21.8173	3.00E-06	0.019611	up	C8orf59	ES	2.2
84402	4.23E-07	21.8173	3.00E-06	0.004603	down	DECR1	ES	8.1
84403	1.40E-07	24.36833	7.96E-07	0.001529	up	DECR1	AA	5.1
84592	2.70E-07	14.18919	0.000165	0.002944	up	NDUFAF6	AT	20
84593	2.70E-07	14.18919	0.000165	0.002944	down	NDUFAF6	AT	19
84894	6.73E-11	38.16324	6.51E-10	7.33E-07	down	ENY2	AD	1.2:1.3
85399	1.57E-18	45.97297	1.20E-11	1.71E-14	down	ZFP41	AT	6
85400	1.89E-13	30.68198	3.04E-08	2.06E-09	up	ZFP41	AT	4
85401	4.05E-08	16.55027	4.74E-05	0.00044	up	ZFP41	AT	3
85430	7.10E-14	45.4888	1.54E-11	7.73E-10	up	NAPRT1	RI	11.5:11.6:1
85435	4.55E-10	21.8173	3.00E-06	4.95E-06	up	EEF1D	AP	7.1
85436	5.79E-16	31.482	2.01E-08	6.30E-12	down	EEF1D	AP	1

85437	4.90E-13	27.81081	1.34E-07	5.34E-09	up	EEF1D	AP	2
85440	3.75E-10	19.40821	1.06E-05	4.08E-06	down	EEF1D	AA	12.1
85455	1.31E-07	11.76263	0.000604	0.001427	up	EEF1D	ES	5:7.2:8.1
85458	3.06E-08	14.46107	0.000143	0.000333	down	EEF1D	AA	8.1
85461	3.72E-08	13.13347	0.00029	0.000405	up	EEF1D	ES	05:07.2
85500	6.37E-15	28.19112	1.10E-07	6.94E-11	down	SCRIB	ES	36
85552	2.57E-10	14.46107	0.000143	2.80E-06	down	BOP1	AT	15
85553	2.57E-10	13.92052	0.000191	2.80E-06	up	BOP1	AT	4.4
85600	2.41E-14	38.16324	6.51E-10	2.63E-10	up	VPS28	RI	3.16
85601	1.45E-13	27.81081	1.34E-07	1.58E-09	up	VPS28	RI	3.14
85602	3.62E-17	41.05644	1.48E-10	3.94E-13	up	VPS28	RI	3.12
85603	2.97E-14	34.11125	5.20E-09	3.23E-10	up	VPS28	RI	3.1
85604	2.75E-14	41.9773	9.23E-11	2.99E-10	up	VPS28	RI	3.8
85608	1.96E-07	21.8173	3.00E-06	0.002136	up	VPS28	RI	3.4:3.5:3.6
85609	1.19E-09	30.68198	3.04E-08	1.29E-05	up	VPS28	AD	3.4:3.5
85610	5.11E-07	16.55027	4.74E-05	0.005559	up	VPS28	AD	3.4
85611	2.42E-06	10.01189	0.001555	0.026388	up	VPS28	RI	3.2
85633	3.88E-11	37.72214	8.16E-10	4.23E-07	up	C8orf82	RI	2.2
85652	1.57E-06	19.09297	1.25E-05	0.017118	down	ZNF517	AT	6
85692	1.28E-07	27.81081	1.34E-07	0.00139	down	CBWD1	RI	4.2
85698	1.73E-09	31.08	2.48E-08	1.88E-05	up	DOCK8	AT	18.2
85699	1.88E-11	27.81081	1.34E-07	2.05E-07	up	DOCK8	AT	28
85701	4.89E-11	34.53081	4.20E-09	5.32E-07	down	DOCK8	AT	55
85752	4.13E-14	41.9773	9.23E-11	4.50E-10	up	SPATA6L	AT	19
85753	9.33E-12	31.08	2.48E-08	1.02E-07	down	SPATA6L	AT	16.2
85794	8.67E-15	38.16324	6.51E-10	9.44E-11	down	KIAA1432	AT	26
85795	8.67E-15	38.16324	6.51E-10	9.44E-11	up	KIAA1432	AT	22.2
85912	1.74E-06	16.55027	4.74E-05	0.0189	up	SNAPC3	AT	10
85914	1.68E-06	16.84383	4.06E-05	0.018325	down	SNAPC3	AT	9.6
85919	1.31E-06	19.09297	1.25E-05	0.014292	down	PSIP1	AT	17
85920	1.31E-06	19.09297	1.25E-05	0.014292	up	PSIP1	AT	11.2
85938	4.32E-20	50.15027	1.42E-12	4.70E-16	down	CNTLN	AT	7.3
85939	6.17E-20	45.97297	1.20E-11	6.72E-16	up	CNTLN	AT	27
86183	3.85E-14	41.51466	1.17E-10	4.20E-10	up	DCTN3	RI	3.6:3.7
86190	2.43E-07	21.48394	3.57E-06	0.002642	up	DCTN3	RI	3.2
86210	4.18E-17	45.97297	1.20E-11	4.55E-13	down	IL11RA	AT	15
86211	4.18E-17	45.97297	1.20E-11	4.55E-13	up	IL11RA	AT	13.2
86237	1.26E-13	27.06166	1.97E-07	1.37E-09	up	STOML2	RI	8.2
86260	4.04E-06	19.09297	1.25E-05	0.043962	down	CCDC107	RI	3.8
86267	6.05E-13	34.53081	4.20E-09	6.58E-09	up	CCDC107	RI	3.2
86268	2.00E-20	45.97297	1.20E-11	2.17E-16	down	ARHGEF39	AP	1
86269	2.00E-20	45.97297	1.20E-11	2.17E-16	up	ARHGEF39	AP	3
86271	3.46E-21	50.15027	1.42E-12	3.76E-17	down	ARHGEF39	AT	11.4
86272	3.46E-21	50.15027	1.42E-12	3.76E-17	up	ARHGEF39	AT	12
86320	3.04E-06	16.55027	4.74E-05	0.033052	up	TMEM8B	AT	13.2
86321	3.04E-06	16.55027	4.74E-05	0.033052	down	TMEM8B	AT	16
86340	2.92E-13	31.482	2.01E-08	3.18E-09	down	GNE	RI	13.2
86444	5.64E-10	19.40821	1.06E-05	6.14E-06	down	EXOSC3	AT	4.2
86445	5.60E-10	18.78117	1.47E-05	6.09E-06	up	EXOSC3	AT	5
86488	3.72E-08	21.8173	3.00E-06	0.000405	up	CBWD6	AT	16
86489	2.61E-08	21.8173	3.00E-06	0.000284	down	CBWD6	AT	4.2
86499	1.65E-12	38.16324	6.51E-10	1.79E-08	down	CBWD5	AT	4.2
86648	7.67E-20	49.64457	1.84E-12	8.35E-16	up	VPS13A	AT	76
86649	9.29E-12	38.16324	6.51E-10	1.01E-07	down	VPS13A	AT	70
86655	2.48E-11	31.08	2.48E-08	2.71E-07	down	CEP78	AT	17
86656	2.48E-11	31.08	2.48E-08	2.71E-07	up	CEP78	AT	16.2
86755	1.20E-07	27.81081	1.34E-07	0.001312	down	ISCA1	AT	6
86756	1.20E-07	27.81081	1.34E-07	0.001307	up	ISCA1	AT	5

86817	5.34E-07	16.55027	4.74E-05	0.005817	up	GADD45G	RI	1.2
86876	2.53E-07	34.53081	4.20E-09	0.002753	up	ECM2	AT	10
86877	1.27E-07	34.53081	4.20E-09	0.001385	down	ECM2	AT	3.2
86898	3.85E-07	13.65504	0.00022	0.004193	up	C9orf89	RI	3.4
86903	1.57E-06	12.00973	0.000529	0.017116	up	C9orf89	RI	3.2
86973	1.93E-15	38.16324	6.51E-10	2.10E-11	up	CDC14B	AT	18
86974	1.93E-15	38.16324	6.51E-10	2.10E-11	down	CDC14B	AT	16
87022	1.28E-08	18.78117	1.47E-05	0.000139	up	XPA	ES	6
87112	6.30E-10	45.97297	1.20E-11	6.86E-06	down	SLC44A1	AT	17
87113	6.30E-10	45.97297	1.20E-11	6.86E-06	up	SLC44A1	AT	16.4
87219	2.18E-14	41.9773	9.23E-11	2.38E-10	up	PTGR1	AA	12.1
87223	5.17E-17	38.16324	6.51E-10	5.62E-13	down	DNAJC25	AT	5
87224	5.17E-17	38.16324	6.51E-10	5.62E-13	up	DNAJC25	AT	7
87276	4.72E-08	16.55027	4.74E-05	0.000514	up	HDHD3	AP	2.1
87277	4.72E-08	16.55027	4.74E-05	0.000514	down	HDHD3	AP	1
87302	9.25E-16	35.88091	2.10E-09	1.01E-11	up	AMBP	AA	6.1
87308	3.98E-06	19.09297	1.25E-05	0.043292	down	ORM1	AT	5
87309	4.31E-06	19.09297	1.25E-05	0.046888	up	ORM1	AT	2.2
87401	3.22E-10	27.81081	1.34E-07	3.51E-06	up	PHF19	AT	5.2
87402	3.22E-10	27.81081	1.34E-07	3.51E-06	down	PHF19	AT	16
87488	5.01E-08	19.09297	1.25E-05	0.000545	up	RC3H2	AT	10
87533	3.74E-10	24.72324	6.62E-07	4.08E-06	up	PSMB7	ES	5
87578	6.14E-07	25.08186	5.49E-07	0.006685	down	MAPKAP1	AT	9
87579	6.14E-07	24.36833	7.96E-07	0.006685	up	MAPKAP1	AT	15
87725	1.70E-10	24.72324	6.62E-07	1.85E-06	up	GOLGA2	AT	2.2
87726	1.71E-10	24.72324	6.62E-07	1.86E-06	down	GOLGA2	AT	26
87735	4.94E-16	50.15027	1.42E-12	5.38E-12	up	COQ4	AT	2.3
87754	2.07E-11	27.43434	1.63E-07	2.25E-07	up	ODF2	AT	22.2
87755	2.07E-11	28.19112	1.10E-07	2.25E-07	down	ODF2	AT	26
87824	1.38E-13	45.97297	1.20E-11	1.50E-09	up	CRAT	AT	4.2
87825	1.38E-13	45.97297	1.20E-11	1.50E-09	down	CRAT	AT	16
88070	2.80E-11	38.16324	6.51E-10	3.05E-07	up	ADAMTSL[illegible]	AP	1
88071	2.80E-11	38.16324	6.51E-10	3.05E-07	down	ADAMTSL[illegible]	AP	2
88079	8.43E-18	50.15027	1.42E-12	9.17E-14	up	SARDH	AT	10
88165	1.59E-06	24.72324	6.62E-07	0.017315	down	PMPCA	AT	13
88166	1.59E-06	24.72324	6.62E-07	0.017315	up	PMPCA	AT	6.2
88212	2.75E-10	34.53081	4.20E-09	2.99E-06	up	RABL6	AT	11
88213	2.75E-10	34.53081	4.20E-09	2.99E-06	down	RABL6	AT	19.2
88229	9.92E-16	41.9773	9.23E-11	1.08E-11	up	EDF1	RI	4.2
88230	2.19E-15	41.9773	9.23E-11	2.39E-11	up	EDF1	RI	4.2:4.3
88245	8.02E-10	29.75174	4.91E-08	8.74E-06	down	ABCA2	AT	46
88246	7.99E-10	27.43434	1.63E-07	8.70E-06	up	ABCA2	AT	9.2
88296	6.00E-16	50.15027	1.42E-12	6.53E-12	up	EXD3	AT	3
88298	1.15E-16	50.15027	1.42E-12	1.25E-12	down	EXD3	AT	27
88486	2.28E-10	38.16324	6.51E-10	2.49E-06	down	MSL3	AT	14
88487	2.28E-10	38.16324	6.51E-10	2.49E-06	up	MSL3	AT	10.2
88513	2.94E-06	14.46107	0.000143	0.032056	down	TRAPPC2	AT	6.3
88515	2.94E-06	16.26003	5.52E-05	0.032056	up	TRAPPC2	AT	5.3
88516	1.42E-07	10.01189	0.001555	0.001549	down	TRAPPC2	RI	6.2
88569	3.58E-07	21.8173	3.00E-06	0.003903	up	AP1S2	AT	5.2
88571	3.58E-07	21.8173	3.00E-06	0.003903	down	AP1S2	AT	6
88700	1.03E-07	25.81036	3.77E-07	0.001123	down	SAT1	ES	2.3
88735	2.26E-18	45.00923	1.96E-11	2.46E-14	up	GK	ES	23
88751	1.43E-11	31.08	2.48E-08	1.56E-07	down	TAB3	AT	12
88752	1.43E-11	31.08	2.48E-08	1.56E-07	up	TAB3	AT	10
88882	2.59E-09	14.18919	0.000165	2.82E-05	up	CXorf36	AT	3
88883	2.59E-09	14.18919	0.000165	2.82E-05	down	CXorf36	AT	6
88904	6.28E-09	27.81081	1.34E-07	6.84E-05	up	NDUFB11	AD	2.2

88921	3.93E-06	19.09297	1.25E-05	0.042785	down	ARAF	AT	16
88922	3.94E-06	19.09297	1.25E-05	0.042913	up	ARAF	AT	6.2
88925	7.38E-11	22.49476	2.11E-06	8.04E-07	down	TIMP1	AT	3.6
88926	7.39E-11	23.86987	1.03E-06	8.05E-07	up	TIMP1	AT	4
88927	1.97E-14	28.57531	9.01E-08	2.15E-10	down	TIMP1	RI	3.4
89098	8.35E-07	19.09297	1.25E-05	0.009093	up	PRICKLE3	AT	5.2
89099	8.35E-07	19.09297	1.25E-05	0.009093	down	PRICKLE3	AT	10.4
89222	3.23E-16	41.51466	1.17E-10	3.51E-12	up	HSD17B10	AD	5.2
89277	1.95E-06	31.08	2.48E-08	0.021248	down	FAM104B	AT	5
89278	1.95E-06	31.08	2.48E-08	0.021248	up	FAM104B	AT	4.2
89443	2.91E-15	45.00923	1.96E-11	3.17E-11	up	PIN4	RI	3.2
89448	2.03E-08	27.06166	1.97E-07	0.000221	up	RPS4X	AT	5
89449	2.03E-08	24.61647	7.00E-07	0.000221	down	RPS4X	AT	8
89563	6.25E-13	50.15027	1.42E-12	6.80E-09	down	CHM	AT	16
89564	6.25E-13	50.15027	1.42E-12	6.80E-09	up	CHM	AT	5
89602	4.95E-07	18.78117	1.47E-05	0.005386	up	SYTL4	AT	13.8
89603	4.95E-07	16.84383	4.06E-05	0.005386	down	SYTL4	AT	17
89806	1.51E-07	38.16324	6.51E-10	0.001646	up	TBC1D8B	AT	7.2
89808	2.00E-10	41.9773	9.23E-11	2.18E-06	down	TBC1D8B	AT	22
89965	1.06E-10	31.08	2.48E-08	1.15E-06	up	6-Sep	AT	11.2
89999	1.62E-08	31.08	2.48E-08	0.000176	up	LAMP2	AT	10
90000	1.53E-07	34.53081	4.20E-09	0.001668	down	LAMP2	AT	11
90156	2.33E-06	14.18919	0.000165	0.025415	up	FAM122B	AA	10.1
90290	3.00E-08	21.8173	3.00E-06	0.000326	up	IDS	AT	6.2
90291	2.49E-06	27.81081	1.34E-07	0.027057	down	IDS	AT	14
90464	2.53E-06	24.72324	6.62E-07	0.027554	down	BCAP31	AP	2
90465	2.52E-06	24.72324	6.62E-07	0.027471	up	BCAP31	AP	1.1
90494	1.50E-07	18.78117	1.47E-05	0.001634	up	IDH3G	RI	11.4
90495	2.56E-06	11.51863	0.000689	0.02786	up	IDH3G	RI	11.2
90499	2.17E-10	27.31395	1.73E-07	2.36E-06	down	SSR4	ES	4
90535	3.94E-06	11.76263	0.000604	0.042901	up	NAA10	AA	8.1:8.2
90622	6.51E-09	19.09297	1.25E-05	7.09E-05	down	FAM3A	AT	10
90623	6.54E-09	19.09297	1.25E-05	7.12E-05	up	FAM3A	AT	7.3
94134	3.61E-06	16.55027	4.74E-05	0.039355	up	SULT1A3	AP	2.1
94136	2.05E-09	27.81081	1.34E-07	2.24E-05	down	SULT1A3	AP	1.1
96728	1.52E-31	243.8095	5.81E-55	1.65E-27	up	ALB	ES	14
99558	2.80E-07	12.00973	0.000529	0.003046	up	CCT3	ME	2|3
152981	1.93E-07	24.72324	6.62E-07	0.002102	down	CD74	ES	6:7.1:8:9

from_exon	to_exon	novel_splic	pct_with_v	psi_range	std_psi
4	6.2	0	1	0.1119	0.0106
null	null	0	1	0.064	0.0108
null	null	0	1	0.064	0.0108
null	null	0	1	0.3331	0.0511
null	null	0	1	0.3331	0.0511
null	null	0	1	0.4721	0.0879
null	null	0	1	0.4721	0.0879
5	6.2	0	1	0.0719	0.011
6	7.2	0	1	0.3797	0.0647
null	null	0	1	0.9486	0.2557
null	null	0	1	0.8247	0.1079
null	null	0	1	0.7133	0.114
null	null	0	1	0.7133	0.114
null	null	0	1	0.4708	0.0674
null	null	0	1	0.4491	0.063
null	null	0	1	0.9555	0.159
null	null	0	1	0.9555	0.159
null	null	0	1	0.198	0.0106
null	null	0	1	0.198	0.0106
null	null	0	1	0.2615	0.0364
null	null	0	1	0.2615	0.0364
null	null	0	1	0.5714	0.0893
null	null	0	1	0.5714	0.0893
null	null	0	1	0.8533	0.1615
null	null	0	1	0.8533	0.1615
null	null	0	1	0.6962	0.1223
null	null	0	1	0.367	0.0376
null	null	0	1	0.2026	0.0206
null	null	0	1	0.5598	0.0944
null	null	0	1	0.5598	0.0944
null	null	0	1	0.4917	0.0983
null	null	0	1	0.4917	0.0983
8.4	8.6	0	1	0.1724	0.0252
null	null	0	1	0.9315	0.2267
null	null	0	1	0.9315	0.2267
null	null	0	1	0.9317	0.1595
null	null	0	1	0.2423	0.0416
null	null	0	1	0.407	0.067
null	null	0	1	0.3552	0.0485
11.1	11.3	0	1	0.9955	0.0957
4	6	0	1	0.3658	0.065
1	3	0	1	0.2325	0.0223
null	null	0	1	0.8999	0.2406
null	null	0	1	0.8999	0.228
null	null	0	1	0.4281	0.0701
null	null	0	1	0.4788	0.0868
null	null	0	1	0.7725	0.1931
null	null	0	1	0.7725	0.1931
3	5	0	1	0.1487	0.0146
null	null	0	1	0.2022	0.0288
null	null	0	1	0.2022	0.0288
null	null	0	1	0.0136	0.0019
null	null	0	1	0.0136	0.0019
null	null	0	1	0.5413	0.0876
null	null	0	1	0.5413	0.0876
null	null	0	1	0.8538	0.1223
null	null	0	1	0.8538	0.1223

null	null	0	1	0.457	0.0337
null	null	0	1	0.457	0.0337
7.1	7.3	0	1	0.109	0.0168
9.1	10.1	0	1	0.5813	0.0954
null	null	0	1	0.4982	0.0782
null	null	0	1	0.4982	0.0782
3.1	4	0	1	0.0143	0.002
1.1	2.1	0	1	0.0283	0.0037
1	3	0	1	0.8865	0.0864
8	10	0	1	0.9378	0.1783
null	null	0	1	0.149	0.0299
null	null	0	1	0.1539	0.0315
null	null	0	1	0.3093	0.0583
null	null	0	1	0.5393	0.1117
8.1	10	0	1	0.7132	0.1407
14.2	16.1	0	1	0.0649	0.0097
11	13	0	1	0.9735	0.2535
11	13	0	1	0.7125	0.1107
null	null	0	1	0.058	0.0107
1.1	2.2	0	1	0.4901	0.0328
null	null	0	1	0.1498	0.0249
null	null	0	1	0.1498	0.0249
null	null	0	1	0.5734	0.0704
null	null	0	1	0.8092	0.1794
null	null	0	1	0.8355	0.1843
null	null	0	1	0.0939	0.0094
null	null	0	1	0.5408	0.058
null	null	0	1	0.5408	0.058
4.2	5.2	0	1	0.3627	0.0502
4.1	7	0	1	0.5702	0.0944
null	null	0	1	0.6065	0.0479
null	null	0	1	0.6065	0.0479
5.1	5.3	0	1	0.3734	0.0227
null	null	0	1	0.7528	0.126
null	null	0	1	0.758	0.13
null	null	0	1	1	0.2111
null	null	0	1	1	0.2111
3	5	0	1	0.0386	0.0053
null	null	0	1	0.5918	0.1093
null	null	0	1	0.5918	0.1093
null	null	0	1	0.5227	0.0974
null	null	0	1	0.4517	0.0515
3	5	0	1	0.517	0.0414
null	null	0	1	0.6373	0.0452
null	null	0	1	0.78	0.0706
null	null	0	1	0.78	0.0706
null	null	0	1	0.1756	0.0236
null	null	0	1	0.1756	0.0236
null	null	0	1	0.0709	0.0092
null	null	0	1	0.0709	0.0092
null	null	0	1	0.3586	0.061
null	null	0	1	0.3586	0.061
null	null	0	1	0.712	0.1192
2.1	2.3	0	1	0.3381	0.0334
11	15	0	1	0.1593	0.0178
null	null	0	1	0.1248	0.0115
null	null	0	1	0.1248	0.0115
3	5	0	1	0.3498	0.0465

null	null	0	1	0.5902	0.1016
null	null	0	1	0.5863	0.0944
null	null	0	1	0.2711	0.0471
null	null	0	1	0.2442	0.0318
6.1	6.3	0	1	0.1656	0.011
4.1	4.3	0	1	0.4421	0.0542
20	23	0	1	0.7907	0.1532
20	23	0	1	0.7995	0.1571
3	5	0	1	0.629	0.0693
null	null	0	1	0.2327	0.0268
null	null	0	1	0.2327	0.0268
3	5	0	1	0.1429	0.0191
3	4.2	0	1	0.0291	0.0041
null	null	0	1	0.4113	0.064
null	null	0	1	0.4113	0.064
null	null	0	1	0.6606	0.1199
null	null	0	1	0.6606	0.1199
6	7.2	0	1	0.1413	0.0259
null	null	0	1	0.2637	0.0354
null	null	0	1	0.2637	0.0354
null	null	0	1	0.321	0.0627
null	null	0	1	0.321	0.0627
14.1	14.3	0	1	0.2526	0.0282
null	null	0	1	0.2853	0.0391
null	null	0	1	0.2853	0.0391
8	10	0	1	0.9961	0.2104
null	null	0	1	0.0304	0.0047
null	null	0	1	0.0304	0.0047
null	null	0	1	0.1675	0.0095
null	null	0	1	0.1675	0.0095
null	null	0	1	0.1002	0.01
null	null	0	1	0.1002	0.01
null	null	0	1	0.0595	0.0088
null	null	0	1	0.0595	0.0088
null	null	0	1	0.4099	0.0666
null	null	0	1	0.3957	0.0682
null	null	0	1	0.8896	0.2126
null	null	0	1	0.6422	0.1182
null	null	0	1	0.6422	0.1182
null	null	0	1	0.9524	0.2025
null	null	0	1	0.9524	0.2025
null	null	0	1	0.0606	0.0081
null	null	0	1	0.0606	0.0081
null	null	0	1	0.3042	0.0445
null	null	0	1	0.1594	0.0255
9.2	12	0	1	0.1295	0.0217
6	7.2	0	1	0.0745	0.014
5.3	6	0	1	0.0437	0.0074
null	null	0	1	0.1546	0.0249
null	null	0	1	0.1546	0.0249
null	null	0	1	0.3877	0.0667
null	null	0	1	0.3877	0.0667
null	null	0	1	0.3717	0.0738
null	null	0	1	0.3717	0.0738
1	4	1	1	0.6598	0.1218
null	null	0	1	0.8254	0.1673
null	null	0	1	0.8254	0.1673
null	null	0	1	0.6102	0.0969

null	null	0	1	0.5447	0.0475
null	null	0	1	0.5447	0.0475
null	null	0	1	0.7145	0.1214
null	null	0	1	0.7145	0.1214
null	null	0	1	0.4039	0.0841
null	null	0	1	0.4039	0.071
null	null	0	1	0.1226	0.0195
null	null	0	1	0.1445	0.0237
null	null	0	1	0.2672	0.0403
null	null	0	1	0.4621	0.059
null	null	0	1	0.4621	0.059
null	null	0	1	0.7945	0.0597
null	null	0	1	0.8429	0.161
null	null	0	1	0.8429	0.161
null	null	0	1	0.8697	0.1533
null	null	0	1	0.8814	0.1878
null	null	0	1	0.7894	0.1807
9.2	9.4	0	1	0.7034	0.1187
null	null	0	1	0.5783	0.1029
null	null	0	1	0.5783	0.1029
2.1	2.3	0	1	0.0734	0.0113
4	6	0	1	0.9743	0.244
null	null	0	1	0.9268	0.1494
null	null	0	1	0.9268	0.1494
null	null	0	1	0.9849	0.1268
null	null	0	1	0.9849	0.1268
null	null	0	1	0.7682	0.1312
null	null	0	1	0.7957	0.1464
null	null	0	1	0.8729	0.1841
null	null	0	1	0.8729	0.1841
null	null	0	1	0.4742	0.0805
null	null	0	1	0.4742	0.0805
null	null	0	1	0.0583	0.0109
null	null	0	1	0.0493	0.0085
null	null	0	1	0.6409	0.097
null	null	0	1	0.2078	0.0331
3	5	0	1	0.6643	0.1308
null	null	0	1	0.2317	0.0213
null	null	0	1	0.2317	0.0213
5.2	7	0	1	0.4932	0.0362
null	null	0	1	0.7869	0.143
null	null	0	1	0.7869	0.143
null	null	0	1	0.2798	0.0396
null	null	0	1	0.2798	0.0396
null	null	0	1	0.0763	0.0126
null	null	0	1	0.5959	0.1054
null	null	0	1	0.2407	0.0182
null	null	0	1	0.2407	0.0182
null	null	0	1	0.5162	0.0474
null	null	0	1	0.5162	0.0474
10.1	10.3	0	1	0.192	0.0335
6.1	6.5	0	1	0.3798	0.0732
6.1	6.3	0	1	0.472	0.0868
6.2	8.3	0	1	0.9649	0.0976
null	null	0	1	0.039	0.0063
null	null	0	1	0.4005	0.0582
null	null	0	1	0.4005	0.0582
null	null	0	1	0.0449	0.0074

null	null	0	1	0.0449	0.0074
2.3	3.4	0	1	0.0079	0.0013
null	null	0	1	0.0622	0.0102
null	null	0	1	0.0622	0.0102
5.3	6.2	0	1	0.535	0.0837
null	null	0	1	0.9987	0.1381
null	null	0	1	0.9978	0.1623
2.4	6.1	0	1	0.2418	0.0337
2.4	4	0	1	0.965	0.1332
null	null	0	1	0.94	0.2116
null	null	0	1	0.94	0.2116
null	null	0	1	0.031	0.0041
null	null	0	1	0.031	0.0041
12.1	12.4	0	1	0.9172	0.1787
null	null	0	1	0.0443	0.0056
null	null	0	1	0.0427	0.0053
8.1	8.3	0	1	0.2741	0.0381
6.11	6.13	0	1	0.4001	0.0293
null	null	0	1	0.0093	0.0014
3.1	3.3	0	1	0.1782	0.0198
null	null	0	1	0.3873	0.0615
null	null	0	1	0.3965	0.0621
null	null	0	1	0.0577	0.0089
null	null	0	1	0.0577	0.0089
22.2	22.4	0	1	0.1891	0.0257
1.1	2.1	0	1	0.0122	0.0019
null	null	0	1	0.1937	0.0231
3.2	4.2	0	1	0.0283	0.0015
null	null	0	1	0.84	0.1719
null	null	0	1	0.84	0.1719
5.1	5.3	0	1	0.0376	0.0053
8.1	8.3	0	1	0.1459	0.0238
3	5	0	1	0.1069	0.017
null	null	0	1	0.0916	0.0177
null	null	0	1	0.0916	0.0177
null	null	0	1	0.8008	0.1003
null	null	0	1	0.5854	0.0397
null	null	0	1	0.66	0.1214
null	null	0	1	0.66	0.1214
null	null	0	1	0.0932	0.0135
null	null	0	1	0.0932	0.0135
null	null	0	1	0.3529	0.0489
1.1	1.3	0	1	0.3598	0.0646
9	10.2	0	1	0.3855	0.0515
8	10.1	0	1	0.409	0.0403
6	8	0	1	0.2599	0.0442
1	4	1	1	0.4924	0.0862
3	6	0	1	0.0716	0.0047
17.1	17.3	0	1	0.2292	0.0371
16.2	17.3	0	1	0.1109	0.0161
1.1	1.3	0	1	0.6151	0.0871
5.1	6	0	1	0.2367	0.0407
null	null	0	1	0.5899	0.1097
null	null	0	1	0.5899	0.1097
null	null	0	1	0.4281	0.0404
null	null	0	1	0.4281	0.0404
8.2	8.4	0	1	0.0887	0.0112
6	8.2	0	1	0.2482	0.0282

5	6.2	0	1	0.1673	0.024
null	null	0	1	0.0424	0.005
1.1	2	0	1	0.3192	0.0454
1	3	0	1	0.1339	0.0215
null	null	0	1	0.0956	0.012
3.1	3.3	0	1	0.5301	0.0612
4.1	5	0	1	0.0699	0.0112
6.1	6.3	0	1	0.1674	0.0194
null	null	0	1	0.154	0.0187
null	null	0	1	0.154	0.0187
null	null	0	1	0.2803	0.042
null	null	0	1	0.2803	0.042
4.1	4.3	0	1	0.0935	0.0117
6	7.2	0	1	0.0955	0.0174
1	4.2	0	1	0.2783	0.0432
null	null	0	1	0.4047	0.073
null	null	0	1	0.4047	0.0733
null	null	0	1	0.9867	0.2622
null	null	0	1	0.9867	0.2622
4	6	0	1	0.1064	0.0113
4	7	0	1	0.0904	0.0126
null	null	0	1	0.1762	0.0286
null	null	0	1	0.4879	0.0497
6.1	6.3	0	1	0.6883	0.0548
5.1	5.3	0	1	0.4349	0.0559
null	null	0	1	0.2688	0.0206
null	null	0	1	0.2688	0.0206
null	null	0	1	0.297	0.0304
null	null	0	1	0.297	0.0304
null	null	0	1	0.0393	0.0028
null	null	0	1	0.0705	0.0048
null	null	0	1	0.1842	0.0186
null	null	0	1	0.1842	0.0182
null	null	0	1	0.6964	0.0923
null	null	0	1	0.6964	0.0923
null	null	0	1	0.1586	0.0249
null	null	0	1	0.0819	0.0054
3	5	0	1	0.8942	0.0712
4.2	4.4	0	1	0.9301	0.2025
1.5	3	0	1	0.0482	0.0033
null	null	0	1	0.0515	0.0083
null	null	0	1	0.0515	0.0083
null	null	0	1	0.6464	0.1351
null	null	0	1	0.6464	0.1351
null	null	0	1	0.691	0.1382
null	null	0	1	0.691	0.1382
5	7	0	1	0.2616	0.0447
null	null	0	1	0.2155	0.0353
null	null	0	1	0.2155	0.0353
null	null	0	1	0.1768	0.0221
null	null	0	1	0.1768	0.0221
null	null	0	1	0.0662	0.0087
null	null	0	1	0.3174	0.0411
2	3.2	0	1	0.1112	0.0179
null	null	0	1	0.0124	0.0017
null	null	0	1	0.0124	0.0017
2.2	3	0	1	0.2134	0.0338
5.1	5.3	0	1	0.1185	0.0166

null	null	0	1	0.2354	0.029
null	null	0	1	0.6372	0.1265
null	null	0	1	0.6077	0.1194
null	null	0	1	0.4255	0.0402
null	null	0	1	0.4255	0.0402
null	null	0	1	0.3809	0.0502
null	null	0	1	0.3436	0.0235
5.1	6	0	1	0.6066	0.0988
null	null	0	1	0.6382	0.0896
null	null	0	1	0.6271	0.0886
null	null	0	1	0.418	0.0623
null	null	0	1	0.418	0.0623
1.1	4	0	1	0.0752	0.0095
null	null	0	1	0.1107	0.0184
null	null	0	1	0.1107	0.0184
null	null	0	1	0.7349	0.1265
null	null	0	1	0.7349	0.1265
null	null	0	1	0.0374	0.0057
null	null	0	1	0.1878	0.0254
null	null	0	1	0.9949	0.2477
null	null	0	1	0.9949	0.247
null	null	0	1	0.9854	0.174
null	null	0	1	0.9867	0.1743
null	null	0	1	0.4379	0.0362
null	null	0	1	0.4379	0.0362
12.1	13	0	1	0.3446	0.0291
null	null	0	1	0.014	0.0013
null	null	0	1	0.014	0.0013
null	null	0	1	0.1008	0.0176
null	null	0	1	0.1008	0.0176
14.1	16.1	0	1	0.5916	0.0941
11.2	14.1	0	1	0.136	0.0142
11.2	14.1	0	1	0.3373	0.0369
7.2	9.1	0	1	0.3596	0.0631
null	null	0	1	0.2254	0.0413
4.1	4.3	0	1	0.08	0.012
7.2	9.1	0	1	0.1744	0.0244
6.1	9.2	0	1	0.7674	0.0989
null	null	0	1	0.1142	0.0171
null	null	0	1	0.1142	0.0171
4.1	4.3	0	1	0.0471	0.0071
null	null	0	1	0.0843	0.0122
2	5.2	0	1	0.6139	0.1525
1.1	1.3	0	1	0.0773	0.0122
null	null	0	1	0.1477	0.0196
null	null	0	1	0.01	0.0015
null	null	0	1	0.01	0.0015
null	null	0	1	0.3884	0.0429
null	null	0	1	0.3884	0.0429
null	null	0	1	0.1344	0.0132
null	null	0	1	0.1344	0.0125
null	null	0	1	0.9257	0.1879
null	null	0	1	0.9257	0.1879
null	null	0	1	0.3903	0.0637
null	null	0	1	0.3903	0.0637
null	null	0	1	0.444	0.0808
null	null	0	1	0.444	0.0808
17	18.2	0	1	0.0903	0.0108

9	12	0	1	0.2255	0.037
null	null	0	1	0.923	0.184
null	null	0	1	0.923	0.184
null	null	0	1	0.9851	0.1561
4	6	0	1	0.8556	0.0848
null	null	0	1	0.579	0.0909
null	null	0	1	0.579	0.0909
null	null	0	1	0.5502	0.1076
null	null	0	1	0.8473	0.1049
null	null	0	1	0.1306	0.0272
null	null	0	1	0.1306	0.0272
1	5	0	1	0.1932	0.0317
null	null	0	1	0.0727	0.0113
null	null	0	1	0.0727	0.0113
null	null	0	1	0.0639	0.0092
null	null	0	1	0.2823	0.0326
null	null	0	1	0.1351	0.0119
null	null	0	1	0.1351	0.0119
1	4	0	1	0.1145	0.0209
null	null	0	1	0.5619	0.1043
null	null	0	1	0.5619	0.1043
4.2	6.2	0	1	0.0647	0.0093
null	null	0	1	0.0176	0.0015
null	null	0	1	0.0176	0.0015
null	null	0	1	0.411	0.0678
null	null	0	1	0.411	0.0678
null	null	0	1	0.2908	0.0454
null	null	0	1	0.3417	0.0546
null	null	0	1	0.9821	0.1103
null	null	0	1	0.9821	0.1103
5.1	5.3	0	1	0.134	0.0232
2.2	3.3	0	1	0.3576	0.0464
2.2	3.3	0	1	0.0768	0.0115
2.1	3.2	0	1	0.4038	0.067
null	null	0	1	0.8085	0.1583
null	null	0	1	0.8085	0.1583
null	null	0	1	0.6145	0.0839
null	null	0	1	0.6145	0.0832
null	null	0	1	0.2714	0.0311
null	null	0	1	0.2714	0.0311
3.2	5	0	1	0.0841	0.0094
null	null	0	1	0.0739	0.0091
null	null	0	1	0.0739	0.0091
null	null	0	1	0.0797	0.0139
null	null	0	1	0.5039	0.0962
null	null	0	1	0.5039	0.0962
6	7.3	0	1	0.0249	0.0031
1.1	2	0	1	0.0563	0.0032
null	null	0	1	0.4144	0.0511
null	null	0	1	0.4144	0.0511
1	6	0	1	0.3756	0.0333
null	null	0	1	0.2349	0.0408
null	null	0	1	0.2349	0.0408
10.1	10.3	0	1	0.3697	0.0634
null	null	0	1	0.5203	0.0526
null	null	0	1	0.5203	0.0526
null	null	0	1	0.7822	0.147
null	null	0	1	0.3138	0.0562

null	null	0	1	0.6242	0.1322
null	null	0	1	0.161	0.0226
null	null	0	1	0.161	0.0226
null	null	0	1	0.7675	0.1285
null	null	0	1	0.7675	0.1285
4	6	0	1	0.3756	0.0651
14.1	14.4	0	1	0.3642	0.0503
null	null	0	1	0.6737	0.1017
null	null	0	1	0.6737	0.1017
15.1	15.3	0	1	1	0.1168
4.4	5	0	1	0.4828	0.0684
4.2	4.4	0	1	0.7041	0.1063
null	null	0	1	0.9832	0.2231
null	null	0	1	0.9832	0.2231
null	null	0	1	0.9793	0.2144
null	null	0	1	0.9793	0.2144
10.1	10.4	0	1	0.6433	0.0874
10.1	10.3	0	1	0.6791	0.1247
9.1	9.3	0	1	0.4241	0.0646
8.1	8.3	0	1	0.8241	0.1236
4.1	5	0	1	0.3252	0.0637
null	null	0	1	0.0951	0.0136
null	null	0	1	0.0951	0.0136
8.3	8.5	0	1	0.7146	0.1351
1.1	1.5	0	1	0.4179	0.0606
1.1	1.4	0	1	0.3655	0.0552
2	4	0	1	0.1575	0.021
5.1	5.3	0	1	0.4677	0.0934
10.1	10.3	0	1	0.1437	0.0224
null	null	0	1	0.035	0.0055
null	null	0	1	0.035	0.0055
3	5	0	1	0.0551	0.0079
3	7.1	0	1	0.54	0.0883
2	7.1	0	1	0.1286	0.0211
null	null	0	1	0.0262	0.0038
null	null	0	1	0.0262	0.0038
4	8.1	0	1	0.716	0.1389
null	null	0	1	0.1504	0.0148
null	null	0	1	0.1504	0.0148
10	12.1	0	1	0.4337	0.0339
10.1	10.4	0	1	0.0565	0.0087
2	3.3	0	1	0.442	0.0784
2	3.3	0	1	0.5676	0.1109
6.3	6.5	0	1	0.4589	0.0766
6.2	6.4	0	1	0.7654	0.1278
null	null	0	1	0.5899	0.0713
null	null	0	1	0.5899	0.0713
null	null	0	1	0.0392	0.0049
null	null	0	1	0.0392	0.0049
45	47	0	1	0.1699	0.0217
null	null	0	1	0.5776	0.108
null	null	0	1	0.5776	0.108
null	null	0	1	0.4303	0.0654
null	null	0	1	0.4303	0.0654
null	null	0	1	0.1871	0.0282
null	null	0	1	0.1871	0.0284
null	null	0	1	0.2167	0.0392
null	null	0	1	0.2167	0.0392

7.2	8.2	0	1	0.7034	0.1442
null	null	0	1	0.2394	0.0393
null	null	0	1	0.2394	0.0393
2.2	3	0	1	0.4959	0.0929
null	null	0	1	0.9664	0.2633
null	null	0	1	0.9664	0.2633
null	null	0	1	0.2334	0.0416
null	null	0	1	0.2334	0.0416
null	null	0	1	0.0419	0.0063
null	null	0	1	0.0419	0.0063
null	null	0	1	0.219	0.0297
null	null	0	1	0.6928	0.1087
null	null	0	1	0.4795	0.0897
null	null	0	1	0.4795	0.0897
2	5	0	1	0.0346	0.0059
21	23	0	1	0.6805	0.109
null	null	0	1	0.2386	0.0254
18.1	18.3	0	1	0.6461	0.1675
null	null	0	1	0.5063	0.0668
null	null	0	1	0.5063	0.0668
null	null	0	1	0.0413	0.0062
null	null	0	1	0.1169	0.0175
null	null	0	1	0.0766	0.012
null	null	0	1	0.3903	0.0318
null	null	0	1	0.1685	0.012
null	null	0	1	0.1685	0.012
3	5	0	1	0.5039	0.0968
null	null	0	1	0.2709	0.0412
null	null	0	1	0.2117	0.0337
null	null	0	1	0.3165	0.0203
null	null	0	1	0.0814	0.0126
null	null	0	1	0.0814	0.0126
null	null	0	1	0.4762	0.0439
null	null	0	1	0.4762	0.0439
null	null	0	1	0.0268	0.0042
null	null	0	1	0.0268	0.0042
null	null	0	1	0.7944	0.1246
null	null	0	1	0.7944	0.1246
null	null	0	1	0.1552	0.0233
null	null	0	1	0.1552	0.0233
null	null	0	1	0.0266	0.0048
null	null	0	1	0.0266	0.0048
15	16.2	0	1	0.09	0.0122
null	null	0	1	0.545	0.096
null	null	0	1	0.545	0.096
2	3.2	0	1	0.1055	0.0116
null	null	0	1	0.2472	0.0424
null	null	0	1	0.079	0.0128
null	null	0	1	0.2105	0.0346
10	12	0	1	0.1479	0.0104
null	null	0	1	0.2082	0.031
null	null	0	1	0.3302	0.0531
null	null	0	1	0.1571	0.028
null	null	0	1	0.2157	0.0314
null	null	0	1	0.2157	0.0314
null	null	0	1	0.6347	0.1737
null	null	0	1	0.8982	0.2081
null	null	0	1	0.8982	0.2081

null	null		0	1	0.7167	0.1184
null	null		0	1	0.5705	0.0819
null	null		0	1	0.6964	0.124
null	null		0	1	0.5384	0.0854
2		4.1	0	1	0.1084	0.0085
null	null		0	1	0.1873	0.0251
null	null		0	1	0.1873	0.0251
5		7	0	1	0.3293	0.0682
null	null		0	1	0.1098	0.0112
null	null		0	1	0.1098	0.0112
null	null		0	1	0.2931	0.0295
null	null		0	1	0.2931	0.0295
5.1		5.3	0	1	0.5956	0.1146
null	null		0	1	0.2288	0.0406
null	null		0	1	0.2288	0.0406
2		5	0	1	0.5209	0.0615
2		5	0	1	0.5621	0.0921
1		5	0	1	0.9106	0.1229
1		5	0	1	0.7906	0.1535
1		3	0	1	0.7101	0.0954
4.1		4.3	0	1	0.3654	0.0642
3		6	0	1	1	0.0633
6.1		6.3	0	1	0.1285	0.02
null	null		0	1	0.171	0.0235
null	null		0	1	0.171	0.0235
2.1		2.5	0	1	0.0269	0.0034
3.1		3.3	0	1	0.5252	0.0921
null	null		0	1	0.5338	0.0721
4.1		4.3	0	1	0.3627	0.062
8		9.3	0	1	0.6113	0.1465
1.5		3.2	0	1	0.483	0.0627
1.5		1.9	0	1	0.1672	0.0155
1.1		1.8	0	1	0.732	0.1396
1.1		1.9	0	1	0.5586	0.084
1.1		1.9	0	1	0.243	0.0354
1.1		1.9	0	1	0.2159	0.0329
1.1		1.9	0	1	0.2357	0.032
1.1		1.9	0	1	0.0581	0.0068
7		8.3	0	1	0.2596	0.0339
null	null		0	1	0.1068	0.0129
null	null		0	1	0.0838	0.0098
null	null		0	1	0.0838	0.0098
null	null		0	1	0.4338	0.0651
null	null		0	1	0.4338	0.0651
8.1		9	0	1	0.805	0.1719
8.1		9	0	1	0.8934	0.1517
null	null		0	1	0.836	0.1743
null	null		0	1	0.836	0.1743
5.1		5.3	0	1	0.0141	0.0022
null	null		0	1	0.7181	0.1345
null	null		0	1	0.7181	0.1345
null	null		0	1	0.592	0.1126
null	null		0	1	0.592	0.1126
null	null		0	1	0.9047	0.2596
null	null		0	1	0.9047	0.2567
null	null		0	1	0.0551	0.0087
null	null		0	1	0.0551	0.0087
6		8	0	1	0.19	0.0266

2.2	4.2	0	1	0.1609	0.0106
null	null	0	1	0.4383	0.0863
null	null	0	1	0.4383	0.0861
5	11	0	1	0.6748	0.0993
5	6.2	0	1	0.5689	0.1097
5.1	6	0	1	0.1015	0.0151
3.1	4.1	0	1	0.1488	0.0251
5	7.1	0	1	0.2096	0.0337
null	null	0	1	0.3025	0.0403
null	null	0	1	0.3025	0.0403
null	null	0	1	0.6	0.0751
null	null	0	1	0.6	0.0751
null	null	0	1	0.7298	0.1352
null	null	0	1	0.7298	0.1352
4.2	6	0	1	0.1191	0.0187
null	null	0	1	0.9869	0.1401
null	null	0	1	0.9869	0.1401
8.1	8.3	0	1	0.5381	0.0826
10.1	11	0	1	0.1772	0.0275
5.1	5.3	0	1	0.033	0.0056
4.1	5.1	0	1	0.1196	0.0158
null	null	0	1	0.3277	0.0637
null	null	0	1	0.3277	0.0637
null	null	0	1	0.155	0.0223
null	null	0	1	0.1617	0.0237
null	null	0	1	0.4318	0.0596
null	null	0	1	0.511	0.0712
null	null	0	1	0.4663	0.0856
null	null	0	1	0.4663	0.0856
null	null	0	1	0.1486	0.0215
null	null	0	1	0.1486	0.0215
null	null	0	1	0.013	0.001
null	null	0	1	0.013	0.001
null	null	0	1	0.0379	0.0068
null	null	0	1	0.0379	0.0068
null	null	0	1	0.2171	0.0413
null	null	0	1	0.2171	0.0413
null	null	0	1	0.9308	0.1068
null	null	0	1	0.9308	0.108
5	12	0	1	0.1913	0.0321
5	9	0	1	0.3839	0.0575
null	null	0	1	0.9254	0.2016
null	null	0	1	0.9254	0.2044
null	null	0	1	0.3539	0.0382
5.1	5.5	0	1	0.6954	0.1432
5.1	5.4	0	1	0.035	0.0056
1	5	0	1	0.888	0.1397
5.1	5.4	0	1	0.047	0.0053
1.1	1.3	0	1	0.0252	0.0025
null	null	0	1	0.6889	0.0996
null	null	0	1	0.6889	0.0996
null	null	0	1	0.2259	0.0398
null	null	0	1	0.3925	0.0695
null	null	0	1	0.3414	0.0465
null	null	0	1	0.2207	0.0348
null	null	0	1	0.2207	0.0348
null	null	0	1	0.2571	0.048
null	null	0	1	0.2571	0.048

6	9	0	1	0.053	0.0074
null	null	0	1	0.7352	0.1609
null	null	0	1	0.7352	0.1609
3	5	0	1	0.5283	0.0669
3	7	0	1	0.7089	0.082
null	null	0	1	0.552	0.1079
2.2	4	0	1	0.1249	0.0198
1.1	1.5	0	1	0.0935	0.014
null	null	0	1	0.4124	0.0499
null	null	0	1	0.0743	0.0138
null	null	0	1	0.0598	0.0099
null	null	0	1	0.1157	0.0208
3.2	5.2	0	1	0.2488	0.0334
1.1	2	0	1	0.5381	0.0917
null	null	0	1	0.7761	0.1237
null	null	0	1	0.8103	0.1272
null	null	0	1	0.2057	0.0205
null	null	0	1	0.2057	0.0205
5.1	5.3	0	1	0.4828	0.0719
3.3	3.5	0	1	0.7084	0.1267
null	null	0	1	0.006	0.001
null	null	0	1	0.5253	0.0869
null	null	0	1	0.5253	0.0869
null	null	0	1	0.157	0.0241
null	null	0	1	0.157	0.0241
null	null	0	1	0.0918	0.0106
null	null	0	1	0.0918	0.0106
3.1	3.3	0	1	0.0786	0.0114
null	null	0	1	0.5993	0.0625
null	null	0	1	0.1789	0.0149
8	10	0	1	0.1609	0.0091
null	null	0	1	0.6837	0.0959
null	null	0	1	0.6837	0.0959
4	6.1	0	1	0.3333	0.0315
null	null	0	1	0.0411	0.0061
null	null	0	1	0.1861	0.025
1	4	0	1	0.9521	0.2725
null	null	0	1	0.1364	0.0233
null	null	0	1	0.1364	0.0233
null	null	0	1	0.3109	0.0539
null	null	0	1	0.3109	0.0539
3	5	0	1	0.0262	0.0034
null	null	0	1	0.6284	0.1213
null	null	0	1	0.6284	0.1213
15.1	15.3	0	1	0.5433	0.0997
null	null	0	1	0.2107	0.0224
null	null	0	1	0.2107	0.0224
null	null	0	1	0.0742	0.008
null	null	0	1	0.0714	0.0068
null	null	0	1	0.0302	0.002
null	null	0	1	0.0188	0.0034
null	null	0	1	0.0188	0.0034
null	null	0	1	0.0285	0.0055
null	null	0	1	0.0285	0.0055
null	null	0	1	0.5953	0.0419
null	null	0	1	0.5953	0.0419
null	null	0	1	0.5369	0.0827
null	null	0	1	0.5369	0.0827

null	null	0	1	0.4342	0.0707
null	null	0	1	0.4586	0.0796
null	null	0	1	0.4586	0.0796
7.1	7.3	0	1	0.796	0.1268
5	6.2	0	1	0.1246	0.0196
2.2	4.2	0	1	0.113	0.0118
null	null	0	1	0.0527	0.0052
null	null	0	1	0.0527	0.0052
3	4.2	0	1	0.0336	0.0058
7.1	9	0	1	0.1078	0.0116
5.1	5.6	0	1	0.0855	0.0118
1	4	0	1	0.1663	0.0217
null	null	0	1	0.4352	0.0671
8.1	8.4	0	1	0.2555	0.0289
null	null	0	1	0.5131	0.0676
null	null	0	1	0.5176	0.0702
3.1	3.4	0	1	0.3021	0.0471
3.5	3.7	0	1	0.5945	0.0858
1	2.4	0	1	0.2786	0.0475
null	null	0	1	0.1057	0.0154
null	null	0	1	0.1057	0.0154
null	null	0	1	0.2576	0.0261
null	null	0	1	0.0369	0.0063
null	null	0	1	0.0369	0.0063
3	6	0	1	0.0152	0.0014
null	null	0	1	0.7393	0.1337
null	null	0	1	0.7393	0.1337
4.1	4.3	0	1	0.1625	0.0257
3.2	4.1	0	1	0.6586	0.1014
18.1	18.3	0	1	0.3059	0.0562
null	null	0	1	0.0404	0.0062
null	null	0	1	0.0404	0.0062
4.3	4.5	0	1	0.0814	0.0076
4.1	4.3	0	1	0.229	0.026
2.5	2.7	0	1	0.2023	0.0394
2.1	2.5	0	1	0.6422	0.0918
2.1	2.5	0	1	0.4206	0.0656
2.1	2.5	0	1	0.4705	0.0665
null	null	0	1	0.0695	0.0076
null	null	0	1	0.707	0.1414
3.2	4.3	0	1	0.3677	0.047
null	null	0	1	0.2183	0.0185
null	null	0	1	0.2183	0.0185
6	12	0	1	0.9964	0.2524
null	null	0	1	0.0423	0.0053
null	null	0	1	0.0416	0.0051
null	null	0	1	0.8507	0.1731
null	null	0	1	0.8512	0.1778
null	null	0	1	0.3596	0.0449
null	null	0	1	0.3777	0.0576
null	null	0	1	0.3777	0.0576
null	null	0	1	0.0065	0.0012
7.2	9	0	1	0.0343	0.0048
7.2	9	0	1	0.0361	0.0066
3.3	7.2	0	1	0.1534	0.0317
3.3	7.2	0	1	0.5399	0.097
3.3	7.2	0	1	0.4681	0.0637
3.3	7.2	0	1	0.333	0.055

3.3	6	0	1	0.1072	0.0192
3.2	7.2	0	1	0.2033	0.0455
3.2	7.2	0	1	0.3868	0.0563
3.2	6	0	1	0.1622	0.0256
6	8	0	1	0.7676	0.1638
15.2	17	0	1	0.2028	0.021
3.1	4.1	0	1	0.4565	0.0653
2	4.1	0	1	0.7958	0.1668
11.2	11.5	0	1	0.5584	0.105
null	null	0	1	0.0407	0.0067
null	null	0	1	0.0407	0.0067
null	null	0	1	0.9615	0.1774
null	null	0	1	0.9615	0.1774
null	null	0	1	0.6371	0.1145
null	null	0	1	0.6371	0.1145
1.1	4	0	1	0.1396	0.0249
null	null	0	1	0.0304	0.0037
null	null	0	1	0.0399	0.0049
1.1	4	0	1	0.3965	0.0222
null	null	0	1	0.6867	0.0853
null	null	0	1	0.6867	0.0853
11	13	0	1	0.7569	0.1268
null	null	0	1	0.3431	0.0593
null	null	0	1	0.3431	0.0593
null	null	0	1	0.2546	0.0385
null	null	0	1	0.2546	0.0385
null	null	0	1	0.8148	0.1441
null	null	0	1	0.6557	0.1363
null	null	0	1	0.5285	0.0963
null	null	0	1	0.6859	0.1101
null	null	0	1	0.2405	0.0382
null	null	0	1	0.1891	0.043
null	null	0	1	0.1891	0.043
null	null	0	1	0.2159	0.0213
null	null	0	1	0.2159	0.0213
4	6	0	1	0.1148	0.0127
5	10	0	1	0.5352	0.0741
null	null	0	1	0.2945	0.0357
7.2	9	0	1	0.2425	0.0326
3	7.2	0	1	0.2979	0.041
17.1	17.3	0	1	0.2309	0.0324
9	11	0	1	0.0603	0.0075
null	null	0	1	0.1468	0.0256
null	null	0	1	0.1468	0.0256
9.6	9.8	0	1	0.5165	0.0918
9.3	9.5	0	1	0.4659	0.08
null	null	0	1	0.6847	0.1211
null	null	0	1	0.6847	0.1211
9.1	9.4	0	1	0.1576	0.026
3.3	3.6	0	1	0.004	8.00E-04
null	null	0	1	0.0145	0.0025
null	null	0	1	0.8966	0.1578
null	null	0	1	0.8966	0.1578
4.2	5.1	0	1	0.381	0.064
5.1	5.3	0	1	0.2872	0.0516
4	5.2	0	1	0.0479	0.0056
1.1	2	0	1	0.071	0.0126
null	null	0	1	0.1176	0.0167

null	null	0	1	0.1176	0.0167
12	14	0	1	0.0604	0.0097
null	null	0	1	0.5931	0.1201
null	null	0	1	0.5931	0.1201
3.1	3.3	0	1	0.191	0.0361
10	12	0	1	0.2378	0.0328
null	null	0	1	0.1955	0.02
null	null	0	1	0.1955	0.02
null	null	0	1	0.5009	0.0356
null	null	0	1	0.5009	0.0356
null	null	0	1	0.0691	0.008
null	null	0	1	0.0691	0.008
null	null	0	1	0.0095	0.0014
null	null	0	1	0.0095	0.0014
4.1	4.3	0	1	0.079	0.0106
null	null	0	1	0.0798	0.0081
null	null	0	1	0.111	0.0156
null	null	0	1	0.111	0.0156
null	null	0	1	0.1539	0.0288
null	null	0	1	0.1539	0.0288
null	null	0	1	0.073	0.0133
null	null	0	1	0.073	0.0133
7	8.2	0	1	0.1702	0.0256
null	null	0	1	0.2687	0.0292
null	null	0	1	0.2687	0.0292
6.1	6.3	0	1	0.2727	0.0365
4.1	4.3	0	1	0.6978	0.0906
null	null	0	1	0.5152	0.0796
null	null	0	1	0.5152	0.0796
6.1	6.3	0	1	0.7435	0.1446
null	null	0	1	0.0587	0.0072
null	null	0	1	0.0587	0.0072
2.2	3	0	1	0.0285	0.0035
2.2	2.5	0	1	0.8594	0.1423
2.2	2.6	0	1	0.3345	0.0427
2.1	2.6	0	1	0.8976	0.1622
6.1	6.3	0	1	0.2327	0.02
null	null	0	1	0.54	0.0937
7	9.1	0	1	0.2341	0.0325
4	6	0	1	0.0237	0.0022
3	6	0	1	0.9809	0.0826
7.1	7.3	0	1	0.4406	0.0511
null	null	0	1	0.1876	0.0296
null	null	0	1	0.1876	0.0296
null	null	0	1	0.6135	0.116
null	null	0	1	0.6979	0.1046
null	null	0	1	0.2478	0.0125
2	7.1	0	1	0.5402	0.0442
3	5	0	1	0.3317	0.0516
null	null	0	1	0.3105	0.0549
null	null	0	1	0.3105	0.0549
5	6.2	0	1	0.3194	0.046
3	7	0	1	0.0304	0.0035
3	7	0	1	0.0528	0.0069
1.2	4.2	0	1	0.7367	0.0747
null	null	0	1	0.531	0.0782
null	null	0	1	0.531	0.0782
5.1	6.2	0	1	0.4567	0.0474

null	null	0	1	0.0166	0.0016
null	null	0	1	0.0567	0.0069
null	null	0	1	0.6152	0.1006
null	null	0	1	0.5739	0.0988
null	null	0	1	0.8906	0.17
null	null	0	1	0.8906	0.17
null	null	0	1	0.8534	0.1928
null	null	0	1	0.7432	0.1492
null	null	0	1	0.7432	0.1492
null	null	0	1	0.5972	0.059
null	null	0	1	0.4751	0.0927
5.3	5.5	0	1	0.1872	0.0298
5.1	5.3	0	1	0.1132	0.0196
10	12	0	1	0.6045	0.0834
null	null	0	1	0.0932	0.0147
null	null	0	1	0.0932	0.0147
2.3	5.2	0	1	0.0277	0.0034
3.1	3.3	0	1	0.0627	0.0103
6.1	6.3	0	1	0.3645	0.065
null	null	0	1	0.7759	0.1643
null	null	0	1	0.4201	0.0812
null	null	0	1	0.0305	0.0046
null	null	0	1	0.0305	0.0046
5	7.1	0	1	0.0557	0.0052
1.1	2	0	1	0.106	0.012
7.1	7.3	0	1	0.2398	0.0355
3.1	3.3	0	1	0.0073	0.0011
null	null	0	1	0.1189	0.0166
null	null	0	1	0.1189	0.0166
3.1	4	0	1	0.0171	0.0022
2	4	0	1	0.5483	0.0413
null	null	0	1	0.9796	0.2407
null	null	0	1	0.9796	0.2407
null	null	0	1	0.9193	0.2608
null	null	0	1	0.9193	0.2608
null	null	0	1	0.5729	0.0733
null	null	0	1	0.5729	0.0733
null	null	0	1	0.5937	0.0944
null	null	0	1	0.9672	0.1336
null	null	0	1	0.5464	0.0941
null	null	0	1	0.7861	0.1552
null	null	0	1	0.7861	0.1552
5.1	7	0	1	0.0225	0.0024
1	3.2	0	1	0.3854	0.0271
4.3	4.5	0	1	0.3102	0.0283
4.2	4.5	0	1	0.5104	0.0928
1.1	3	0	1	0.4498	0.0696
1.1	2	0	1	0.7212	0.1166
8	10.1	0	1	0.3181	0.0566
7	11	0	1	0.0735	0.0098
null	null	0	1	0.6839	0.1094
null	null	0	1	0.6839	0.1094
null	null	0	1	0.4807	0.0943
null	null	0	1	0.4807	0.0943
null	null	0	1	0.8548	0.2178
null	null	0	1	0.8548	0.2178
null	null	0	1	0.8643	0.2157
null	null	0	1	0.8643	0.2157

1	5	0	1	0.0972	0.0195
null	null	0	1	0.1778	0.0201
null	null	0	1	0.1778	0.0201
6.2	6.4	0	1	0.0145	0.0019
1	6	0	1	0.9144	0.1853
1	4	0	1	0.9699	0.2754
4	6.1	0	1	0.0422	0.0072
2.2	3.2	0	1	9.00E-04	1.00E-04
2.2	8.1	0	1	0.0742	0.0064
5.2	6.2	0	1	0.081	0.0124
4	6.2	0	1	0.1716	0.0176
13.1	13.3	0	1	0.7041	0.1152
13.1	13.4	0	1	0.4461	0.0354
null	null	0	1	0.5692	0.0965
null	null	0	1	0.7471	0.1417
1.1	3.2	0	1	0.3614	0.0573
null	null	0	1	0.0612	0.0107
null	null	0	1	0.5478	0.0816
null	null	0	1	0.5478	0.0816
3.2	5	0	1	0.4602	0.0602
null	null	0	1	0.7691	0.1252
null	null	0	1	0.7691	0.1252
4.1	4.3	0	1	0.3338	0.0598
4.1	4.5	0	1	0.027	0.0044
4.1	4.5	0	1	0.0245	0.0046
4.1	4.5	0	1	0.1836	0.0237
3	4.5	0	1	0.6518	0.1091
3	4.5	0	1	0.9335	0.1909
3	4.5	0	1	0.9208	0.1828
3	4.5	0	1	0.7599	0.1504
15.1	15.5	0	1	0.9028	0.1759
null	null	0	1	0.7053	0.158
null	null	0	1	0.7053	0.158
null	null	0	1	0.6802	0.146
null	null	0	1	0.6802	0.146
null	null	0	1	0.705	0.1527
null	null	0	1	0.705	0.1527
null	null	0	1	0.5145	0.0965
null	null	0	1	0.802	0.1825
null	null	0	1	0.4673	0.0829
null	null	0	1	0.233	0.0262
null	null	0	1	0.8341	0.1477
null	null	0	1	0.3211	0.0595
null	null	0	1	0.1287	0.0136
null	null	0	1	0.1199	0.0147
null	null	0	1	0.1199	0.0147
null	null	0	1	0.8366	0.1093
null	null	0	1	0.8366	0.1093
null	null	0	1	0.0227	0.0031
null	null	0	1	0.0227	0.0031
7	8.3	0	1	0.316	0.0332
null	null	0	1	0.5091	0.0954
null	null	0	1	0.6674	0.1168
null	null	0	1	0.2569	0.0145
1.1	1.3	0	1	0.0248	0.0041
null	null	0	1	0.9883	0.0947
null	null	0	1	0.2789	0.0459
null	null	0	1	0.4202	0.0715

null	null		0	1	0.4246	0.0723
null	null		0	1	0.9914	0.3367
null	null		0	1	0.9899	0.3348
null	null		0	1	0.3735	0.0809
null	null		0	1	0.4931	0.0873
null	null		0	1	0.5614	0.098
null	null		0	1	0.5614	0.0989
null	null		0	1	0.4143	0.0419
null	null		0	1	0.0742	0.0103
null	null		0	1	0.5232	0.0725
null	null		0	1	0.5232	0.0725
null	null		0	1	0.3957	0.0661
null	null		0	1	0.3957	0.0661
null	null		0	1	0.2063	0.0295
null	null		0	1	0.2063	0.0295
null	null		0	1	0.8083	0.0989
null	null		0	1	0.8095	0.0993
null	null		0	1	0.5005	0.0683
null	null		0	1	0.5112	0.0654
null	null		0	1	0.028	0.005
null	null		0	1	0.028	0.005
null	null		0	1	0.1293	0.0105
null	null		0	1	0.1293	0.0105
null	null		0	1	0.162	0.0174
null	null		0	1	0.162	0.0174
	4	7	0	1	0.6494	0.1038
	1	4	0	1	0.665	0.0976
	10	11.2	0	1	0.1437	0.0224
	2.3	2.5	0	1	0.4596	0.0482
null	null		0	1	0.0405	0.0074
null	null		0	1	0.0405	0.0074
	4.1	4.3	0	1	0.0238	0.0036
null	null		0	1	0.7703	0.1344
null	null		0	1	0.7554	0.1294
null	null		0	1	0.4994	0.0583
null	null		0	1	0.4994	0.0583
null	null		0	1	0.3586	0.0452
null	null		0	1	0.3586	0.0452
null	null		0	1	0.8172	0.084
null	null		0	1	0.8172	0.084
null	null		0	1	0.6435	0.1026
null	null		0	1	0.6435	0.1026
null	null		0	1	0.7522	0.0925
null	null		0	1	0.7522	0.0925
null	null		0	1	0.8546	0.1573
null	null		0	1	0.8546	0.1573
	3.1	3.3	0	1	0.3268	0.0226
	1	3	0	1	0.0645	0.0119
null	null		0	1	0.4196	0.0756
null	null		0	1	0.1667	0.0275
	8.1	11	0	1	0.9152	0.1841
	8.1	8.3	0	1	0.1013	0.0134
	6.2	8.1	0	1	0.1059	0.0072
null	null		0	1	0.2514	0.0441
null	null		0	1	0.2514	0.0441
null	null		0	1	0.72	0.0723
null	null		0	1	0.7118	0.0978
null	null		0	1	0.4553	0.0734

null	null	0	1	0.2062	0.0402
null	null	0	1	0.2062	0.0402
40.2	40.4	0	1	0.3888	0.0693
4.9	4.11	0	1	0.4531	0.0818
4.7	4.9	0	1	0.3194	0.0559
4.5	4.7	0	1	0.1365	0.0225
4.3	4.5	0	1	0.2991	0.0297
9.1	9.4	0	1	0.4983	0.0754
7	11	0	1	0.7134	0.136
7	11	0	1	0.6722	0.135
null	null	0	1	0.1209	0.0205
null	null	0	1	0.5065	0.0707
null	null	0	1	0.5065	0.0707
14.1	15	0	1	0.631	0.1134
9	11.2	0	1	0.9829	0.2011
null	null	0	1	0.6441	0.1153
null	null	0	1	0.6441	0.1153
null	null	0	1	0.6982	0.1098
null	null	0	1	0.6982	0.1098
7.1	8.2	0	1	0.5179	0.0661
null	null	0	1	0.6946	0.1213
null	null	0	1	0.6946	0.1213
null	null	0	1	0.3128	0.0435
null	null	0	1	0.3128	0.0435
null	null	0	1	0.3555	0.05
null	null	0	1	0.3555	0.05
7	9	0	1	0.1686	0.019
12.1	13	0	1	0.7621	0.1277
11	13	0	1	0.2101	0.0301
9.1	9.3	0	1	0.4639	0.0443
null	null	0	1	0.5129	0.051
null	null	0	1	0.5175	0.0609
4	6	0	1	0.608	0.0661
18.2	19	0	1	0.2402	0.0244
17	18.2	0	1	0.4841	0.052
null	null	0	1	0.4753	0.077
null	null	0	1	0.4753	0.077
5	8	0	1	0.9331	0.1912
1.2	1.5	0	1	0.1716	0.0185
1.1	1.5	0	1	0.3597	0.0439
1.1	1.5	0	1	0.2569	0.0337
3.1	3.3	0	1	0.6607	0.1135
2	5	0	1	0.5434	0.075
2	4.1	0	1	0.3659	0.0564
16	18	0	1	0.3212	0.0357
null	null	0	1	0.1088	0.014
null	null	0	1	0.1088	0.014
null	null	0	1	0.3795	0.0637
null	null	0	1	0.3795	0.0637
null	null	0	1	0.0947	0.0177
null	null	0	1	0.0947	0.0177
5	6.2	0	1	0.3078	0.0525
null	null	0	1	0.55	0.0807
null	null	0	1	0.55	0.0807
5	8.2	0	1	0.5543	0.0695
2.1	2.3	0	1	0.0082	0.0013
null	null	0	1	0.1087	0.0145
null	null	0	1	0.1087	0.0145

75	77	0	1	0.1746	0.0163
null	null	0	1	0.2022	0.0362
5.2	6.2	0	1	0.8978	0.2393
8.3	8.5	0	1	0.083	0.0134
null	null	0	1	0.8446	0.152
null	null	0	1	0.8446	0.152
1.5	3	0	1	0.0737	0.0141
1.3	3	0	1	0.6297	0.1143
null	null	0	1	0.5078	0.0734
null	null	0	1	0.5078	0.0734
8	10.2	0	1	0.3434	0.0414
null	null	0	1	0.4333	0.0632
null	null	0	1	0.0467	0.0069
null	null	0	1	0.7283	0.1136
null	null	0	1	0.7181	0.1129
null	null	0	1	0.52	0.1161
null	null	0	1	0.9586	0.2395
null	null	0	1	0.9459	0.2421
2.1	3.3	0	1	0.0486	0.006
null	null	0	1	0.2101	0.0372
null	null	0	1	0.2101	0.0372
null	null	0	1	0.4439	0.0583
null	null	0	1	0.4439	0.0583
null	null	0	1	0.3381	0.0523
null	null	0	1	0.2787	0.0416
null	null	0	1	0.6492	0.1084
null	null	0	1	0.6492	0.1084
19.1	19.3	0	1	0.1633	0.0276
null	null	0	1	0.0526	0.0093
null	null	0	1	0.0526	0.0093
7	8.2	0	1	0.0528	0.0075
7.1	7.4	0	1	0.2645	0.0441
7.1	7.3	0	1	0.1957	0.0298
2.2	4	0	1	0.1102	0.0134
null	null	0	1	0.3223	0.0517
null	null	0	1	0.3223	0.0517
16.1	16.3	0	1	0.8849	0.1672
null	null	0	1	0.0842	0.0096
null	null	0	1	0.0842	0.0096
null	null	0	1	0.8285	0.0834
null	null	0	1	0.8285	0.0834
null	null	0	1	0.4154	0.09
1.1	2	0	1	0.233	0.0426
null	null	0	1	0.4617	0.0658
null	null	0	1	0.4617	0.0658
20	22	0	1	0.1788	0.0262
1.1	1.3	0	1	0.1978	0.0322
null	null	0	1	0.3982	0.0616
null	null	0	1	0.3982	0.0616
null	null	0	1	0.6136	0.096
null	null	0	1	0.6136	0.096
null	null	0	1	0.1239	0.0206
null	null	0	1	0.7769	0.1251
null	null	0	1	0.7769	0.1249
13	14.2	0	1	0.524	0.1034
19.1	19.3	0	1	0.6973	0.1215
1	4	0	1	0.3573	0.0495
null	null	0	1	0.9004	0.1678

null	null	0	1	0.6672	0.1579
5	7	0	1	0.5692	0.0724
18.1	18.3	0	1	0.2797	0.0543
null	null	0	1	0.4698	0.069
null	null	0	1	0.4698	0.069
null	null	0	1	0.1621	0.0237
null	null	0	1	0.1621	0.0237
18	21	0	1	0.6313	0.0928
11	13	0	1	0.2658	0.0384
null	null	0	1	0.516	0.0795
null	null	0	1	0.39	0.0495
null	null	0	1	0.6061	0.1131
3.1	4.1	0	1	0.2108	0.0175
null	null	0	1	0.2335	0.0377
null	null	0	1	0.4668	0.0889
null	null	0	1	0.4668	0.0889
null	null	0	1	0.8052	0.1694
null	null	0	1	0.8052	0.1694
null	null	0	1	0.0851	0.0115
null	null	0	1	0.0883	0.0121
9.1	10	0	1	0.8611	0.1685
9.1	10	0	1	0.1946	0.0321
1.1	2	0	1	0.1815	0.0298
18.1	18.4	0	1	0.5746	0.0847
18.1	18.4	0	1	0.7296	0.1136
null	null	0	1	0.668	0.1051
null	null	0	1	0.6437	0.1079
null	null	0	1	0.0888	0.0137
null	null	0	1	0.1547	0.0225
null	null	0	1	0.1547	0.0225
null	null	0	1	0.7579	0.1281
null	null	0	1	0.7579	0.1281
null	null	0	1	0.4454	0.0824
null	null	0	1	0.4454	0.0824
null	null	0	1	0.0599	0.011
null	null	0	1	0.0505	0.0088
null	null	0	1	0.0449	0.0058
null	null	0	1	0.0906	0.0141
null	null	0	1	0.0906	0.0141
null	null	0	1	0.4018	0.0626
null	null	0	1	0.3743	0.0537
14.1	14.3	0	1	0.9362	0.1821
8.1	8.3	0	1	0.2469	0.0383
9.1	9.3	0	1	0.1768	0.0287
14	15.2	0	1	0.3749	0.069
12	15	0	1	0.0412	0.0064
null	null	0	1	0.0047	7.00E-04
null	null	0	1	0.0047	7.00E-04
3.1	4	0	1	0.0296	0.0026
6.3	6.5	0	1	0.5907	0.1
6.1	6.3	0	1	0.8046	0.1219
2	4	0	1	0.4583	0.054
1.1	2	0	1	0.3155	0.0556
13.1	19.2	0	1	0.2739	0.0175
null	null	0	1	0.8909	0.2125
null	null	0	1	0.8909	0.2124
3.1	4	0	1	0.1903	0.0173
null	null	0	1	0.7786	0.1627

null	null	0	1	0.7786	0.1627
2.1	2.3	0	1	0.0754	0.0114
1.1	2.1	0	1	0.0332	0.0045
null	null	0	1	0.0913	0.0134
null	null	0	1	0.0913	0.0134
10	12	0	1	0.2607	0.0341
null	null	0	1	0.4767	0.0647
null	null	0	1	0.0349	0.0074
null	null	0	1	0.0349	0.0074
null	null	0	1	0.6412	0.1162
null	null	0	1	0.6412	0.1162
3.1	3.3	0	1	0.1804	0.0306
null	null	0	1	0.3323	0.0502
null	null	0	1	0.3323	0.0502
null	null	0	1	0.4609	0.093
null	null	0	1	0.4609	0.093
null	null	0	1	0.4746	0.0713
null	null	0	1	0.4744	0.072
1.1	3	0	1	0.1629	0.0256
null	null	0	1	0.2085	0.0274
null	null	0	1	0.2085	0.0274
null	null	0	1	0.5362	0.0793
null	null	0	1	0.5362	0.0793
1	3	0	1	0.0722	0.0108
null	null	0	1	0.4045	0.0723
null	null	0	1	0.4045	0.0723
4	6	0	1	0.8522	0.071
null	null	0	1	0.9571	0.2304
null	null	0	1	0.9599	0.2276
null	null	0	1	0.7599	0.0784
null	null	0	1	0.7599	0.0784
null	null	0	1	0.0999	0.0109
null	null	0	1	0.0999	0.0109
10	12	0	1	0.6941	0.1264
8	9.2	0	1	0.058	0.01
null	null	0	1	0.8827	0.1683
null	null	0	1	0.3821	0.0328
null	null	0	1	0.8635	0.1644
null	null	0	1	0.4238	0.1117
null	null	0	1	0.4238	0.1117
null	null	0	1	0.7243	0.1108
null	null	0	1	0.6476	0.1429
3.1	5	0	1	0.0113	0.0018
null	null	0	1	1	0.2241
null	null	0	1	1	0.2241
5.1	6	0	1	0.3105	0.0397
null	null	0	1	0.8055	0.1259
null	null	0	1	0.8055	0.1259
null	null	0	1	0.1944	0.0336
null	null	0	1	0.1944	0.0336
null	null	0	1	0.0135	0.0021
null	null	0	1	0.0135	0.0021
5	7	0	1	0.2531	0.0272
null	null	0	1	0.0453	0.0049
null	null	0	1	0.2072	0.0279
null	null	0	1	0.0574	0.0062
null	null	0	1	0.9334	0.0917
null	null	0	1	0.9334	0.0917

8.1	8.3	0	1	0.7349	0.1358
4	6	0	1	0.9857	0.1239
null	null	0	1	0.5733	0.1417
null	null	0	1	0.5733	0.1413
null	null	0	1	0.7901	0.0742
null	null	0	1	0.7491	0.1046
null	null	0	1	0.7422	0.1124
null	null	0	1	0.1892	0.0318
null	null	0	1	0.6641	0.1128
null	null	0	1	0.1069	0.0119
null	null	0	1	0.1099	0.0105
null	null	0	1	0.4674	0.0952
null	null	0	1	0.2745	0.0417
null	null	0	1	0.2235	0.0292
null	null	0	1	0.1447	0.0209
null	null	0	1	0.1447	0.0209
7	9	0	1	0.5301	0.0968
null	null	0	1	0.5712	0.1172
null	null	0	1	0.6788	0.1513
10.1	11	0	1	0.9761	0.2598
null	null	0	1	0.1808	0.0339
null	null	0	1	0.3414	0.0587
null	null	0	1	0.3414	0.031
null	null	0	1	0.4501	0.0363
null	null	0	1	0.4276	0.0325
null	null	0	1	0.8607	0.1631
null	null	0	1	0.8607	0.1631
26	28	0	1	0.9931	0.1526
10.1	10.5	0	1	0.3288	0.044
null	null	0	1	0.1906	0.02
null	null	0	1	0.1118	0.0131
null	null	0	1	0.1955	0.025
1	3	0	1	0.3779	0.0659
12	15	0	1	0.0948	0.0107
null	null	0	1	0.1243	0.0123
null	null	0	1	0.1243	0.0123
3	5	0	1	0.0297	0.0042
null	null	0	1	0.1546	0.0208
null	null	0	1	0.1546	0.0208
2	4	0	1	0.0822	0.0062
null	null	0	1	0.651	0.1253
null	null	0	1	0.651	0.1253
null	null	0	1	0.4417	0.0713
null	null	0	1	0.4417	0.0713
null	null	0	1	0.4897	0.0641
null	null	0	1	0.1963	0.0177
null	null	0	1	0.4976	0.0776
3.2	3.4	0	1	0.013	0.0019
3.1	3.6	0	1	0.143	0.023
2.2	2.4	0	1	0.0108	0.0017
2.1	2.4	0	1	0.01	0.001
null	null	0	1	0.6025	0.0846
null	null	0	1	0.6025	0.0846
2.1	4	0	1	0.0278	0.0039
2.1	4	0	1	0.0282	0.0046
null	null	0	1	0.6892	0.0801
null	null	0	1	0.6793	0.0796
null	null	0	1	0.9451	0.1431

null	null	0	1	0.9166	0.1761
null	null	0	1	0.2894	0.0437
null	null	0	1	0.2894	0.0437
19	21	0	1	0.0375	0.0063
null	null	0	1	0.9827	0.2636
null	null	0	1	0.9827	0.2636
7.1	9	0	1	0.3405	0.0527
4	10.2	0	1	0.3465	0.0528
4	10.2	0	1	0.0499	0.0081
5	7	0	1	0.0108	0.0014
1.1	3	0	1	0.0619	0.0066
1.1	3	0	1	0.0564	0.0042
21	23	0	1	0.5208	0.094
6.1	6.3	0	1	0.0251	0.0029
null	null	0	1	0.161	0.0245
null	null	0	1	0.161	0.0245
1.3	3	0	1	0.3597	0.028
null	null	0	1	0.4784	0.0794
null	null	0	1	0.4784	0.0794
null	null	0	1	0.4547	0.0457
null	null	0	1	0.4547	0.0457
null	null	0	1	0.0788	0.0058
null	null	0	1	0.3938	0.0409
null	null	0	1	0.3938	0.0409
null	null	0	1	0.4505	0.0842
null	null	0	1	0.4505	0.0842
null	null	0	1	0.6813	0.0553
13	15	0	1	0.4327	0.0599
null	null	0	1	0.9626	0.145
null	null	0	1	0.9626	0.145
null	null	0	1	0.3574	0.0577
7.1	7.3	0	1	0.9113	0.1787
null	null	0	1	0.114	0.0147
null	null	0	1	0.114	0.0147
null	null	0	1	0.7986	0.139
null	null	0	1	0.7986	0.139
null	null	0	1	0.7605	0.1995
null	null	0	1	0.7605	0.1995
6.3	6.5	0	1	0.6777	0.1254
6.1	6.3	0	1	0.6782	0.114
2.3	3	0	1	0.0108	0.0015
1.1	2.1	0	1	0.0402	0.0049
null	null	0	1	0.486	0.0702
null	null	0	1	0.4766	0.07
null	null	0	1	0.0814	0.0117
null	null	0	1	0.789	0.071
null	null	0	1	0.789	0.071
null	null	0	1	0.4573	0.0851
null	null	0	1	0.4573	0.0851
1	3	0	1	0.0663	0.0086
1.1	2	0	1	0.8226	0.1054
8.1	9	0	1	0.7983	0.0984
9	10.2	0	1	0.3438	0.0593
null	null	0	1	0.8173	0.1597
null	null	0	1	0.7353	0.1469
null	null	0	1	0.2397	0.0204
null	null	0	1	0.2397	0.0204
null	null	0	1	0.035	0.0048

null	null	0	1	0.035	0.0048
null	null	0	1	0.7688	0.1249
null	null	0	1	0.7664	0.126
3.1	3.3	0	1	0.2793	0.0438
4.7	4.9	0	1	0.0236	0.0027
4.5	4.7	0	1	0.0508	0.0055
4	6	0	1	0.0759	0.0115
null	null	0	1	0.8795	0.12
null	null	0	1	0.3069	0.0387
null	null	0	1	0.2145	0.0196
5.1	6	0	1	0.5039	0.074
null	null	0	1	0.3708	0.0241
null	null	0	1	0.2929	0.0197
null	null	0	1	0.8006	0.1166
null	null	0	1	0.8006	0.1166
null	null	0	1	0.6731	0.113
null	null	0	1	0.6731	0.113
null	null	0	1	0.0319	0.0033
null	null	0	1	0.0319	0.0033
null	null	0	1	0.6702	0.1262
null	null	0	1	0.4974	0.0546
null	null	0	1	0.5413	0.0728
null	null	0	1	0.161	0.0176
null	null	0	1	0.0536	0.0072
null	null	0	1	0.0536	0.0072
null	null	0	1	0.4674	0.0866
null	null	0	1	0.4674	0.0866
null	null	0	1	0.133	0.0198
null	null	0	1	0.4324	0.0434
null	null	0	1	0.4324	0.0434
null	null	0	1	0.522	0.0787
null	null	0	1	0.522	0.0787
null	null	0	1	0.5606	0.1034
null	null	0	1	0.4406	0.0884
null	null	0	1	0.6998	0.1653
null	null	0	1	0.6998	0.1653
null	null	0	1	0.4319	0.0706
12.1	13	0	1	0.4542	0.081
null	null	0	1	0.4185	0.0362
null	null	0	1	0.4185	0.0362
null	null	0	1	0.6125	0.0864
null	null	0	1	0.5974	0.0898
null	null	0	1	0.3089	0.0554
null	null	0	1	0.3089	0.0554
11	12.2	0	1	0.2089	0.0338
null	null	0	1	0.3374	0.0491
null	null	0	1	0.3374	0.0491
null	null	0	1	0.636	0.0444
null	null	0	1	0.636	0.0444
null	null	0	1	0.8317	0.0954
null	null	0	1	0.8317	0.0954
null	null	0	1	0.5021	0.1027
1.1	1.3	0	1	0.2349	0.0341
null	null	0	1	0.5869	0.1141
null	null	0	1	0.5141	0.1136
null	null	0	1	0.5044	0.1123
1	3	0	1	0.1652	0.0272
4	9	0	1	0.8169	0.1482

2.1	3	0	1	0.1524	0.0226
null	null	0	1	0.199	0.0183
null	null	0	1	0.0237	0.0041
null	null	0	1	0.0237	0.0041
12	14	0	1	0.1424	0.0097
7	9	0	1	0.2112	0.0337
null	null	0	1	0.5879	0.1029
null	null	0	1	0.5879	0.1029
9.1	9.3	0	1	0.4746	0.0869
null	null	0	1	0.0971	0.0177
null	null	0	1	0.1704	0.0329
10.1	13	0	1	0.3104	0.0395
null	null	0	1	0.2293	0.0395
null	null	0	1	0.3701	0.0516
null	null	0	1	0.3701	0.0516
16.2	16.5	0	1	0.1048	0.0172
null	null	0	1	0.4184	0.059
null	null	0	1	0.2953	0.0458
null	null	0	1	0.2953	0.0458
null	null	0	1	0.9382	0.1465
null	null	0	1	0.9382	0.1465
4.1	4.3	0	1	0.965	0.1684
1.1	3.2	0	1	0.4749	0.0766
3.1	3.3	0	1	0.1215	0.0151
9	11	0	1	0.9945	0.1686
null	null	0	1	0.1578	0.0187
null	null	0	1	0.1578	0.0187
2.1	4	0	1	0.1002	0.0184
12.1	12.3	0	1	0.8412	0.0964
6	8	0	1	0.2825	0.0293
null	null	0	1	0.4555	0.052
null	null	0	1	0.4555	0.052
null	null	0	1	0.3499	0.0594
null	null	0	1	0.3985	0.0863
null	null	0	1	0.3985	0.0863
null	null	0	1	0.1567	0.0264
null	null	0	1	0.0214	0.0034
null	null	0	1	0.1382	0.0149
null	null	0	1	0.0807	0.0086
null	null	0	1	0.0208	0.0036
3	10	0	1	0.0095	0.0015
null	null	0	1	0.3016	0.0575
null	null	0	1	0.3022	0.0565
1.1	3	0	1	0.2743	0.046
5	7.1	0	1	0.0141	0.0014
4.1	5	0	1	0.0535	0.0081
4.1	4.3	0	1	0.3493	0.0514
null	null	0	1	0.9718	0.195
null	null	0	1	0.9718	0.195
1.2	1.4	0	1	0.7544	0.0574
null	null	0	1	0.455	0.0629
null	null	0	1	0.4378	0.0359
4.3	6	0	1	0.0684	0.0097
2.1	4.3	0	1	0.027	0.0038
5.9	5.11	0	1	0.1753	0.0237
5.7	5.9	0	1	0.1888	0.0354
5.5	5.7	0	1	0.2354	0.0416
5.3	5.5	0	1	0.3306	0.0529

5.1	5.3	0	1	0.2264	0.0373
3.1	4	0	1	0.1872	0.0317
null	null	0	1	0.6287	0.1257
null	null	0	1	0.6287	0.1257
null	null	0	1	0.5417	0.1083
null	null	0	1	0.5417	0.1083
null	null	0	1	0.0941	0.0092
null	null	0	1	0.5381	0.1054
null	null	0	1	0.5381	0.1054
null	null	0	1	0.0825	0.0087
null	null	0	1	0.0824	0.0087
null	null	0	1	0.2241	0.0434
null	null	0	1	0.2241	0.0425
null	null	0	1	0.9104	0.297
null	null	0	1	0.2563	0.0584
null	null	0	1	0.0404	0.0044
null	null	0	1	0.6591	0.1969
null	null	0	1	0.1726	0.0324
null	null	0	1	0.1726	0.0324
null	null	0	1	0.2454	0.0489
null	null	0	1	0.239	0.0478
2	4	0	1	0.8374	0.1611
null	null	0	1	0.6751	0.1317
null	null	0	1	0.6532	0.1281
null	null	0	1	0.774	0.1572
null	null	0	1	0.774	0.1572
2.1	2.3	0	1	0.0177	0.0028
4	5.2	0	1	0.459	0.0771
null	null	0	1	0.0766	0.0088
null	null	0	1	0.5949	0.1219
null	null	0	1	0.6225	0.1258
9	11.1	0	1	0.106	0.0142
7.1	7.3	0	1	0.0524	0.0069
null	null	0	1	0.8591	0.1824
null	null	0	1	0.2236	0.0278
null	null	0	1	0.8733	0.1918
null	null	0	1	0.1929	0.029
null	null	0	1	0.1929	0.029
null	null	0	1	0.1105	0.0138
null	null	0	1	0.1105	0.0138
1	5	0	1	0.2577	0.0266
null	null	0	1	0.3629	0.0474
null	null	0	1	0.3629	0.0474
null	null	0	1	0.2021	0.0298
null	null	0	1	0.2021	0.0298
1	3.2	0	1	0.6628	0.105
1	3.2	0	1	0.1923	0.0316
7	9	0	1	0.0217	0.0027
4.2	5.2	0	1	0.3685	0.0208
null	null	0	1	0.1141	0.0223
null	null	0	1	0.1141	0.0223
1.1	2.1	0	1	0.3639	0.0657
null	null	0	1	0.768	0.1173
null	null	0	1	0.5996	0.0867
null	null	0	1	0.5997	0.0958
11.4	11.8	0	1	0.6319	0.101
null	null	0	1	0.1309	0.0152
null	null	0	1	0.274	0.0391

null	null	0	1	0.1929	0.03
11	12.2	0	1	0.0234	0.0031
1	8.2	0	1	0.3786	0.0338
1	8.2	0	1	0.2071	0.0201
1	8.1	0	1	0.0922	0.0072
35	37	0	1	0.7203	0.1562
null	null	0	1	0.1648	0.0234
null	null	0	1	0.1648	0.0234
3.15	3.17	0	1	0.2818	0.0386
3.13	3.15	0	1	0.0719	0.0093
3.11	3.13	0	1	0.198	0.0247
3.9	3.11	0	1	0.1695	0.0232
3.7	3.9	0	1	0.1875	0.0283
3.3	3.7	0	1	0.0514	0.0097
3.3	3.7	0	1	0.0738	0.0124
3.3	3.7	0	1	0.0717	0.009
3.1	3.3	0	1	0.0681	0.0101
2.1	2.3	0	1	0.1378	0.0184
null	null	0	1	0.5803	0.108
4.1	4.3	0	1	0.9635	0.1887
null	null	0	1	0.6512	0.102
null	null	0	1	0.1618	0.0233
null	null	0	1	0.6921	0.1182
null	null	0	1	0.8744	0.1829
null	null	0	1	0.8744	0.181
null	null	0	1	0.444	0.05
null	null	0	1	0.444	0.05
null	null	0	1	0.5857	0.1255
null	null	0	1	0.5935	0.127
null	null	0	1	0.7891	0.0897
null	null	0	1	0.7891	0.0897
null	null	0	1	0.5619	0.1067
null	null	0	1	0.605	0.1094
3.5	3.8	0	1	0.0919	0.0085
3.1	3.3	0	1	0.0482	0.0078
null	null	0	1	0.3794	0.0753
null	null	0	1	0.3794	0.0753
8.1	8.3	0	1	0.1988	0.0147
3.7	3.9	0	1	0.5574	0.0718
3.1	3.3	0	1	0.2661	0.0449
null	null	0	1	0.9152	0.2382
null	null	0	1	0.9152	0.2382
null	null	0	1	0.8631	0.1637
null	null	0	1	0.8631	0.1637
null	null	0	1	0.8099	0.154
null	null	0	1	0.8099	0.154
13.1	13.3	0	1	0.8353	0.0798
null	null	0	1	0.8828	0.1213
null	null	0	1	0.8828	0.1213
null	null	0	1	0.7512	0.1454
null	null	0	1	0.7512	0.1456
null	null	0	1	0.6785	0.1252
null	null	0	1	0.7529	0.1134
null	null	0	1	0.6261	0.1212
null	null	0	1	0.7904	0.1516
null	null	0	1	0.7904	0.1516
null	null	0	1	0.0893	0.0164
null	null	0	1	0.0893	0.0164

1.1	1.3	0	1	0.206	0.0286
null	null	0	1	0.6781	0.1143
null	null	0	1	0.6781	0.1148
3.3	3.5	0	1	0.5828	0.0753
3.1	3.3	0	1	0.2978	0.0504
null	null	0	1	0.6639	0.0834
null	null	0	1	0.6639	0.0834
5	7	0	1	0.2162	0.0336
null	null	0	1	0.8759	0.2018
null	null	0	1	0.8759	0.2018
11	12.2	0	1	0.7182	0.0919
null	null	0	1	0.535	0.0937
null	null	0	1	0.535	0.0937
null	null	0	1	0.9222	0.1341
null	null	0	1	0.9222	0.1341
5	6.2	0	1	1	0.0487
null	null	0	1	0.4672	0.0291
null	null	0	1	0.4672	0.0291
null	null	0	1	0.7811	0.1589
null	null	0	1	0.7811	0.1589
null	null	0	1	0.1522	0.0157
4	6	0	1	0.0751	0.0078
null	null	0	1	0.5099	0.0842
null	null	0	1	0.5099	0.0842
null	null	0	1	0.2374	0.0229
null	null	0	1	0.2374	0.0229
null	null	0	1	0.463	0.0709
null	null	0	1	0.5877	0.0811
null	null	0	1	0.5877	0.0811
null	null	0	1	0.1583	0.0202
null	null	0	1	0.1583	0.0202
null	null	0	1	0.6635	0.1414
null	null	0	1	0.6635	0.1414
null	null	0	1	0.1308	0.0112
null	null	0	1	0.0819	0.0118
null	null	0	1	0.0819	0.0118
null	null	0	1	0.4095	0.0657
null	null	0	1	0.4095	0.0657
4.1	4.3	0	1	0.7354	0.1101
4.1	4.4	0	1	0.1355	0.0171
null	null	0	1	0.0492	0.0047
null	null	0	1	0.0492	0.0047
null	null	0	1	0.8683	0.2152
null	null	0	1	0.7729	0.1782
null	null	0	1	0.278	0.0451
null	null	0	1	0.278	0.0451
null	null	0	1	0.1172	0.0228
null	null	0	1	0.1172	0.0228
6.1	6.3	0	1	0.4608	0.0814
null	null	0	1	0.6378	0.1153
null	null	0	1	0.6378	0.1153
2.1	3	0	1	0.0608	0.0047
22	24	0	1	0.8512	0.1189
null	null	0	1	0.2974	0.0361
null	null	0	1	0.2974	0.0361
null	null	0	1	0.4326	0.0605
null	null	0	1	0.4326	0.0605
2.1	3	0	1	0.295	0.0378

null	null	0	1	0.1114	0.015
null	null	0	1	0.1114	0.015
null	null	0	1	0.0274	0.0028
null	null	0	1	0.0274	0.0028
3.3	3.5	0	1	0.0464	0.0054
null	null	0	1	0.3114	0.0548
null	null	0	1	0.3114	0.0548
5.1	6	0	1	0.0674	0.0134
null	null	0	1	0.5879	0.1042
null	null	0	1	0.5879	0.1042
3.1	3.3	0	1	0.0821	0.0089
null	null	0	1	0.02	0.003
null	null	0	1	0.02	0.003
null	null	0	1	0.7395	0.1456
null	null	0	1	0.7395	0.1456
null	null	0	1	0.3696	0.0448
null	null	0	1	0.3696	0.0448
null	null	0	1	0.6985	0.1375
null	null	0	1	0.5136	0.1074
null	null	0	1	0.1263	0.0209
null	null	0	1	0.6347	0.105
null	null	0	1	0.6554	0.1163
9	10.2	0	1	0.9353	0.1208
null	null	0	1	0.2697	0.0367
null	null	0	1	0.7599	0.1424
null	null	0	1	0.4372	0.0687
null	null	0	1	0.4372	0.0687
11.3	11.5	0	1	0.2401	0.0424
11.1	11.3	0	1	0.0439	0.0054
3	5	0	1	0.0286	0.0041
7	8.3	0	1	0.0291	0.0048
null	null	0	1	0.1194	0.0189
null	null	0	1	0.1194	0.0189
null	null	0	1	0.7378	0.1127
null	null	0	1	0.7678	0.1541
13	15	0	1	0.0156	8.00E-04
1	4	0	1	0.361	0.0599
4	10.2	1	1	0.5748	0.0913

Table S3. Statistics of splicing types of DASEs.

splice_type	event	gene
AA	59	54
AD	75	70
AP	184	99
AT	1026	538
ES	235	193
ME	3	3
RI	195	156

Table S4. Statistics of splicing types of DASEs in each subtype

cluster	group	AA	AD	AP	AT	ES	ME	RI
cluster1	up	21	40	77	592	75	1	142
	down	19	10	76	545	67	2	14
cluster3	up	31	37	147	832	105	0	123
	down	39	15	159	853	152	3	49
cluster4	up	61	96	164	881	136	1	226
	down	35	26	144	795	269	4	24

Table S5. Differential channel statistics.

	all	up	down
cluster1 vs. normal	755	272	483
cluster3 vs. normal	2726	977	1749
cluster4 vs. normal	3284	1173	2111

	logFC	AveExpr	t	P.Value	adj.P.Val	B	group
RICHERT_F	-0.85751	0.133387	-11.3853	4.99E-24	2.59E-22	43.9033	down
FAN_EMBF	-0.85009	-0.01126	-12.2308	1.00E-26	1.11E-24	50.03715	down
KUWANO_	-0.84315	0.048276	-13.8793	4.45E-32	2.47E-29	62.20898	down
GOCC_GR(	-0.80531	0.077491	-13.5447	5.50E-31	1.97E-28	59.72385	down
CHASSOT_	-0.79547	0.124599	-11.5479	1.52E-24	9.04E-23	45.07451	down
GOBP_NE(	-0.78238	0.019651	-11.3681	5.66E-24	2.88E-22	43.77908	down
SMID_BRE/	-0.77452	0.048829	-11.9163	1.02E-25	8.35E-24	47.74432	down
GOBP_NE(	-0.77024	0.019503	-11.2368	1.47E-23	6.72E-22	42.8366	down
REACTOM	-0.76626	0.116896	-11.4351	3.47E-24	1.88E-22	44.26161	down
REACTOM	-0.76041	0.172949	-13.5755	4.36E-31	1.68E-28	59.95291	down
REACTOM	-0.75542	0.134816	-13.4801	8.94E-31	3.04E-28	59.24477	down
GUTIERRE	-0.75353	0.081672	-11.8998	1.15E-25	9.34E-24	47.62414	down
GAUTSCHI	-0.74902	0.046306	-12.0861	2.92E-26	2.82E-24	48.98069	down
chr6p11	0.748495	-0.09097	9.923636	1.71E-19	2.95E-18	33.60251	up
GOBP_RES	0.747586	-0.03639	13.02544	2.70E-29	6.59E-27	55.87938	up
GOBP_NE(	-0.74457	0.092171	-10.8608	2.23E-22	7.66E-21	40.15508	down
HP_ENDO(	0.742776	-0.07241	14.49352	4.34E-34	3.79E-31	66.78077	up
GOBP_RES	-0.74129	0.056139	-13.1628	9.65E-30	2.70E-27	56.89502	down
REACTOM	-0.73705	0.105287	-11.5096	2.02E-24	1.14E-22	44.79833	down
REACTOM	-0.7361	0.192069	-13.0997	1.55E-29	4.03E-27	56.42818	down
GOMF_N_I	-0.73237	0.090052	-9.71976	7.06E-19	1.07E-17	32.20612	down
GOCC_HO	0.730464	-0.17572	11.52786	1.76E-24	1.02E-22	44.93003	up
GOBP_IMN	-0.72934	0.101411	-10.7701	4.27E-22	1.38E-20	39.51203	down
GOMF_LIP	-0.7233	0.092278	-13.6989	1.73E-31	7.74E-29	60.869	down
GOBP_REG	-0.72215	0.032162	-10.2307	1.98E-20	4.27E-19	35.7264	down
GOBP_CO	-0.72043	0.164071	-13.7729	9.90E-32	4.84E-29	61.41841	down
GOBP_T_H	-0.71577	0.048998	-11.5033	2.11E-24	1.19E-22	44.75297	down
GOBP_RES	-0.71394	0.073764	-9.81513	3.64E-19	5.87E-18	32.85792	down
GOMF_SPI	-0.71246	0.043353	-10.0119	9.23E-20	1.70E-18	34.2108	down
REACTOM	-0.70849	0.163742	-13.5492	5.32E-31	1.93E-28	59.75712	down
GOMF_HIS	0.708459	-0.05209	8.311321	8.86E-15	6.06E-14	22.91878	up
HP_CRYPT	-0.70708	0.019688	-11.5536	1.46E-24	8.73E-23	45.11578	down
GNF2_MKI	0.705648	-0.24991	9.725391	6.79E-19	1.03E-17	32.24453	up
ZNF211_T/	0.703287	-0.20202	9.983749	1.12E-19	2.03E-18	34.0164	up
IIZUKA_LIV	-0.7031	0.160489	-14.8722	2.50E-35	2.88E-32	69.60218	down
GOBP_NE(	-0.70282	0.050043	-11.405	4.32E-24	2.28E-22	44.04502	down
HP_CAUD/	-0.7024	0.176636	-11.3472	6.59E-24	3.31E-22	43.62911	down
GOBP_SM(	-0.70201	0.036675	-9.70711	7.70E-19	1.15E-17	32.11986	down
CROSBY_E	0.699534	-0.29495	9.767518	5.07E-19	7.93E-18	32.53219	up
GOBP_FEN	0.698187	-0.20325	10.41745	5.29E-21	1.32E-19	37.02976	up
GOBP_POS	-0.69741	0.01238	-10.8419	2.55E-22	8.66E-21	40.02122	down
CAO_BLO(	0.69704	-0.16318	11.7724	2.94E-25	2.13E-23	46.69901	up
MONTERC	0.691764	-0.24455	10.13023	4.03E-20	7.97E-19	35.02887	up
GNF2_RRN	0.691119	-0.25235	9.7216	6.97E-19	1.06E-17	32.21866	up
GNF2_HMI	0.690934	-0.2473	9.779754	4.66E-19	7.33E-18	32.61583	up
GOCC_CO	0.690555	-0.22921	11.25726	1.27E-23	5.90E-22	42.98353	up
GOBP_L_K	-0.69004	0.245907	-10.7673	4.36E-22	1.40E-20	39.49264	down
BLANCO_N	0.69002	-0.14276	10.40672	5.71E-21	1.41E-19	36.95459	up
BIOCARTA	-0.68987	0.206857	-11.9215	9.82E-26	8.10E-24	47.78184	down
EGUCHI_CI	0.688349	-0.23462	9.665807	1.02E-18	1.48E-17	31.83852	up
FINETTI_BF	0.68739	-0.24854	10.02271	8.56E-20	1.59E-18	34.28516	up
BIOCARTA	-0.68694	0.16456	-12.5589	8.76E-28	1.37E-25	52.44189	down
GOCC_DEI	0.686743	-0.1823	9.776843	4.75E-19	7.47E-18	32.59593	up
ANDERSOI	-0.68405	0.025403	-8.8305	2.95E-16	2.60E-15	26.26222	down
REACTOM	-0.68367	0.142321	-12.7821	1.66E-28	3.01E-26	54.08452	down
GOBP_NE(	-0.68339	0.125606	-11.7005	4.98E-25	3.41E-23	46.17797	down
GOBP_NO	-0.68336	0.103029	-11.2796	1.08E-23	5.14E-22	43.14383	down

passways	number	genes
GOBP_NEGATIVE_REGULATION_OF_MRNA_SPLICING_VIA_SPLICEOSOME	20	C1QBP
GOBP_POSITIVE_REGULATION_OF_MRNA_SPLICING_VIA_SPLICEOSOME	23	CELF3
GOBP_REGULATION_OF_ALTERNATIVE_MRNA_SPLICING_VIA_SPLICEOSOM	57	CELF1
GOBP_REGULATION_OF_MRNA_SPLICING_VIA_SPLICEOSOME	100	C1QBP
GOBP_RNA_SPLICING_VIA_ENDONUCLEOLYTIC_CLEAVAGE_AND_LIGATION	16	C2orf49
GOBP_RNA_SPLICING_VIA_TRANSESTERIFICATION_REACTIONS	384	AAR2
GOBP_SPLICEOSOMAL_COMPLEX_ASSEMBLY	78	AC126323.
GOBP_SPLICEOSOMAL_CONFORMATIONAL_CHANGES_TO_GENERATE_CA	6	ISY1
GOBP_SPLICEOSOMAL_TRI_SNRNP_COMPLEX_ASSEMBLY	25	AAR2
GOCC_CATALYTIC_STEP_2_SPLICEOSOME	87	ALYREF
GOCC_EXON_EXON_JUNCTION_COMPLEX	20	ALYREF
GOCC_PRESPLICEOSOME	17	LSM7
GOCC_SPLICEOSOMAL_COMPLEX	191	AAR2
GOCC_U12_TYPE_SPLICEOSOMAL_COMPLEX	29	AQR
GOCC_U2_TYPE_CATALYTIC_STEP_2_SPLICEOSOME	30	AQR
GOCC_U2_TYPE_SPLICEOSOMAL_COMPLEX	93	DHX15
GOMF_PRE_MRNA_3_SPLICE_SITE_BINDING	7	SLU7
GOMF_PRE_MRNA_5_SPLICE_SITE_BINDING	18	LINC01715
KEGG_SPLICEOSOME	127	ACIN1
LEE_METASTASIS_AND_ALTERNATIVE_SPLICING_DN	45	ABCC4
LEE_METASTASIS_AND_ALTERNATIVE_SPLICING_UP	74	ADRA1A
REACTOME_FGFR2_ALTERNATIVE_SPLICING	27	ESRP1
REACTOME_MRNA_SPLICING	188	ALYREF
REACTOME_MRNA_SPLICING_MINOR_PATHWAY	52	DDX23

Table S8. Top 10 most relevant displays in each subtype

	cluster3	cluster4
BIOCARTA_SLRP_PATHWAY	AIZARANI_LIVER_C21_STELLATE_CELLS_1	AIZARANI_LIVER_C21_STELLATE_CELLS_1
DESCARTES_FETAL_INTESTINE_MYELOID_CELLS	DESCARTES_FETAL_LIVER_STELLATE_CELLS	GOBP_RESPONSE_TO_MOLECULE_OF_FUNGAL_ORIGIN
DESCARTES_FETAL_STOMACH_MYELOID_CELLS	DESCARTES_FETAL_HEART_STROMAL_CELLS	DESCARTES_MAIN_FETAL_MYELOID_CELLS
GNF2_CD97	DESCARTES_MAIN_FETAL_STROMAL_CELLS	GNF2_PECAM1
DESCARTES_FETAL_ADRENAL_MYELOID_CELLS	BOQUEST_STEM_CELL_UP	GNF2_CARD15
SOTIRIOU_BREAST_CANCER_GRADE_1_VS_3_UP	BIOCARTA_SLRP_PATHWAY	QI_PBMC_ZOSTAVAX_AGE_50_75YO_CORRELATED_WITH_T_CELL_RESPONSES_EXPANSION_AND_CONTRACTION_1D_NOT_INFORMATIVE_OF_LONG_TERM_RESPONSES_POSITIVE
DESCARTES_FETAL_LUNG_MYELOID_CELLS	GOMF_SIALIC_ACID_BINDING	DESCARTES_FETAL_LIVER_STELLATE_CELLS
GNF2_CASP1	WONG_ENDMETRIUM_CANCER_DN	GNF2_CD1D

	p	adj.p
BAY.61.360	2.86E-20	4.00E-18
AZD6482	3.86E-20	5.40E-18
KIN001.13!	6.24E-19	8.73E-17
AZD7762	4.30E-18	6.02E-16
NVP.TAE68	1.05E-17	1.47E-15
GDC0941	1.95E-17	2.73E-15
EHT.1864	2.16E-17	3.02E-15
AG.014699	3.00E-17	4.20E-15
BMS.70816	5.84E-17	8.18E-15
Bosutinib	1.92E-16	2.68E-14
AS601245	2.04E-16	2.85E-14
Dasatinib	2.09E-16	2.93E-14
Midostauri	1.25E-15	1.75E-13
WZ.1.84	2.74E-15	3.83E-13
Elesclomol	3.59E-15	5.03E-13
Salubrinal	1.22E-14	1.71E-12
IPA.3	9.02E-14	1.26E-11
WO200909	1.70E-13	2.38E-11
Pazopanib	8.87E-13	1.24E-10
AKT.inhibit	1.40E-12	1.96E-10
BI.2536	1.91E-12	2.68E-10
Bortezomil	2.30E-12	3.22E-10
Cyclopami	2.37E-12	3.32E-10
CGP.60474	2.99E-12	4.18E-10
ABT.263	3.28E-12	4.59E-10
GW843682	3.81E-12	5.33E-10
Bexarotene	3.99E-12	5.59E-10
MS.275	4.77E-12	6.68E-10
PAC.1	6.94E-12	9.71E-10
Sorafenib	7.54E-12	1.06E-09
FH535	2.21E-11	3.09E-09
BX.795	2.28E-11	3.19E-09
SB590885	3.65E-11	5.12E-09
XMD8.85	3.83E-11	5.36E-09
JNJ.268541	5.56E-11	7.79E-09
SB.216763	6.65E-11	9.32E-09
DMOG	2.09E-10	2.93E-08
A.443654	2.23E-10	3.12E-08
RDEA119	2.90E-10	4.07E-08
PD.033299	3.10E-10	4.34E-08
Bryostatin.	3.60E-10	5.04E-08
OSI.906	3.88E-10	5.43E-08
Roscovitine	5.35E-10	7.49E-08
Docetaxel	5.50E-10	7.70E-08
Nutlin.3a	6.20E-10	8.67E-08
TW.37	6.78E-10	9.49E-08
NVP.BEZ23	1.03E-09	1.44E-07
Rapamycir	1.07E-09	1.49E-07
S.Trityl.L.cy	1.15E-09	1.61E-07
Bleomycin	1.48E-09	2.08E-07
Gefitinib	1.58E-09	2.21E-07
RO.3306	1.92E-09	2.69E-07
AZ628	2.14E-09	2.99E-07
AP.24534	5.46E-09	7.65E-07
Cytarabine	7.99E-09	1.12E-06
AICAR	1.01E-08	1.41E-06
PF.562271	1.26E-08	1.77E-06

ATRA	1.75E-08	2.45E-06
Gemcitabir	3.62E-08	5.06E-06
Vorinostat	3.64E-08	5.10E-06
VX.702	4.83E-08	6.76E-06
GSK26996:	4.94E-08	6.92E-06
NU.7441	5.94E-08	8.32E-06
BI.D1870	7.11E-08	9.95E-06
PLX4720	8.59E-08	1.20E-05
Erlotinib	1.01E-07	1.41E-05
AUY922	1.80E-07	2.52E-05
Etoposide	2.03E-07	2.85E-05
Paclitaxel	2.25E-07	3.14E-05
BMS.75480	2.98E-07	4.17E-05
WH.4.023	3.30E-07	4.62E-05
Sunitinib	3.68E-07	5.15E-05
CMK	4.82E-07	6.75E-05
Z.LLNle.CH	5.40E-07	7.56E-05
Shikonin	5.75E-07	8.06E-05
Tipifarnib	6.99E-07	9.79E-05
QS11	1.08E-06	0.000151
KU.55933	1.09E-06	0.000152
ZM.447439	1.28E-06	0.000179
Camptothe	1.44E-06	0.000202
JW.7.52.1	1.94E-06	0.000272
ABT.888	1.99E-06	0.000279
SL.0101.1	2.40E-06	0.000336
GSK.65039	2.74E-06	0.000383
CEP.701	3.01E-06	0.000421
Nilotinib	3.41E-06	0.000478
Lenalidom	3.83E-06	0.000536
AZD.0530	4.09E-06	0.000572
Metformin	4.52E-06	0.000633
GNF.2	4.71E-06	0.000659
X17.AAG	5.60E-06	0.000783
GW.44175(	7.35E-06	0.001028
JNK.Inhibit	7.64E-06	0.00107
CCT00709:	1.02E-05	0.001429
AZD6244	1.03E-05	0.001449
PD.032590	1.12E-05	0.001561
Epothilone	1.95E-05	0.002728
Thapsigarç	2.00E-05	0.002801
Doxorubici	2.46E-05	0.003451
JNK.9L	3.07E-05	0.004297
AZD.2281	3.75E-05	0.005252
Pyrimethar	6.76E-05	0.009466
CI.1040	6.90E-05	0.009666
BIBW2992	7.56E-05	0.010584
Bicalutami(	8.37E-05	0.011724
A.770041	9.00E-05	0.012605
X681640	9.02E-05	0.012633
Methotrex;	9.96E-05	0.013946
Cisplatin	0.000122	0.017028
Axitinib	0.000327	0.045847
AZD8055	0.000335	0.046908

REACTOME_FCGR_ACTIVATION	DESCARTES_MAIN_FETAL_MYELOID_CELLS	ERWIN_COHEN_PBMC_TC_83_AGE_18_45YO_NON_RESPONDERS_PREVIOUSLY_IMMUNIZED_24HR_DEG_CANONICAL_PATHWAY_MEMBERS_DN
DURANTE_ADULT_OLFACTORY_NEUROEPITHELIUM_MONOCYTES	GNF2_CARD15	GNF2_CD33

CELF4	DYRK1A	HNRNPA2	HNRNPK	PCBP4	PTBP1	RBM10	RBM42	RBMX
CELF4	CIRBP	DAZAP1	HMX2	HSPA8	NCBP1	NCL	NUP98	PRDX6
CELF2	CELF3	CELF4	CELF5	CELF6	DDX17	DDX5	DYRK1A	FAM172A
C9orf78	CELF1	CELF2	CELF3	CELF4	CELF5	CELF6	CIRBP	CWC22
CLP1	CPSF1	CPSF4	CSTF2	DDX1	ERN1	FAM98B	RTCB	RTRAF
AC126323.	ALYREF	AQR	BCAS2	BUD13	BUD31	C1QBP	C9orf78	CACTIN
CELF1	CELF2	CELF3	CELF4	CELF5	CELF6	CRNKL1	DDX1	DDX23
PRPF18	PRPF19	SNRNP200	XAB2	YJU2				
CD2BP2	DDX20	LSM2	PRPF19	PRPF3	PRPF31	PRPF6	PRPF8	RNU4-1
AQR	BCAS2	BUD31	CACTIN	CDC40	CDC5L	CRNKL1	CWC15	CWC22
CASC3	EIF4A3	MAGOH	MAGOHB	PNN	PYM1	R3HCC1	R3HCC1L	RBM8A
LUC7L	LUC7L2	LUC7L3	PRPF39	PRPF40A	PRPF40B	SF3A1	SF3A2	SF3B1
ADAR	AKAP17A	ALYREF	API5	AQR	BCAS2	BUD13	BUD31	C9orf78
BCAS2	BUD31	CDC40	CDC5L	CRNKL1	CWC15	CWC22	DHX8	EFTUD2
BCAS2	BUD13	BUD31	CASC3	CCDC12	CCDC130	CDC40	CDC5L	CRNKL1
LSM7	PDCD7	PHF5A	RBM41	RBMXL2	RBMXL3	RNPC3	SF3B1	SF3B2
U2AF1	U2AF1L4	U2AF1L5	U2AF2	ZRSR2	ZRSR2P1			
RNU1-4	RNU11	RNU4ATA(	RNU6ATA(	RNVU1-1	RNVU1-14	RNVU1-15	RNVU1-17	RNVU1-19
ALYREF	AQR	BCAS2	BUD31	CCDC12	CDC40	CDC5L	CHERP	CRNKL1
ADAMTS1:	API5	CEBPG	CELSR2	CLK4	COL9A3	CSF3	CSNK2A1	CYP19A1
ANKS1A	ANKS1B	BCL2A1	BID	BMF	BMP1	BNIP3	BTK	C6
ESRP2	FGFR2	GTF2F1	GTF2F2	HNRNPA1	HNRNPF	HNRNPH1	HNRNPM	NCBP1
AQR	BCAS2	BUD31	CASC3	CCAR1	CD2BP2	CDC40	CDC5L	CHERP
DDX42	EFTUD2	GTF2F1	GTF2F2	LSM2	NCBP1	NCBP2	PDCD7	POLR2A

RNPS1	SAP18	SFSWAP	SRSF10	SRSF12	SRSF4	SRSF6	SRSF7	SRSF9
PRPF19	RBM3	RBMX	RBMXL1	RBMY1B	SF3B4	SLC39A5	SNRNP70	SNW1
FMR1	FXR1	FXR2	HNRNPA1	HNRNPL	HNRNPU	KHDRBS1	KHDRBS2	KHDRBS3
DAZAP1	DDX17	DDX5	DYRK1A	FAM172A	FMR1	FXR1	FXR2	HMX2
TRPT1	TSEN15	TSEN2	TSEN34	TSEN54	ZBTB8OS			
CASC3	CCAR1	CD2BP2	CDC40	CDC5L	CDK13	CELF1	CELF2	CELF3
DDX39B	GCFC2	GEMIN2	GEMIN6	ISY1	KHDC4	LINC0171[illegible]	LUC7L	LUC7L2
RNU4-2	RNU4ATA[illegible]	RNU5A-1	RNU5B-1	RNU5D-1	RNU5E-1	RNU5F-1	RNU6-1	RNU6-7
CWC27	DDX23	DDX41	DDX5	DHX35	DHX38	DHX8	EFTUD2	EIF4A3
RNPS1	SAP18	SMG6	SRSF1	TDRD3	THRAP3	UPF1	UPF2	UPF3A
SNRNP70	SNRPB	SNRPC	SNRPG	SNRPGP15	SNRPN	U2AF2		
CACTIN	CASC3	CCDC12	CCDC130	CDC40	CDC5L	CIRBP	CRNKL1	CTNNBL1
PLRG1	PPIE	PPIL1	PRPF19	PRPF8	RBM22	SNRNP40	SNRPA1	SNRPB
CWC15	CWC22	CWC25	CWC27	DHX15	DHX16	DHX8	EFTUD2	EIF4A3
SF3B3	SF3B4	SF3B5	SF3B6	SNRNP25	SNRNP35	SNRNP48	SNRPB	SNRPD1
RNVU1-2[illegible]	RNVU1-3	RNVU1-4	RNVU1-6	RNVU1-7	RNVU1-8	SNRPC	WEE2-AS1	
CTNNBL1	CWC15	DDX23	DDX39B	DDX42	DDX46	DDX5	DHX15	DHX16
DHRS2	DVL3	EIF2AK1	EIF4G2	ERCC6	ERGIC1	FCHO1	GRB14	GSTM3
CAMK1	CCBE1	CCN1	CEACAM1	COL4A6	COL6A2	COL7A1	CSNK1E	CTSV
NCBP2	POLR2A	POLR2B	POLR2C	POLR2D	POLR2E	POLR2F	POLR2G	POLR2H
CLP1	CPSF1	CPSF2	CPSF3	CPSF4	CPSF7	CRNKL1	CSTF1	CSTF2
POLR2B	POLR2C	POLR2D	POLR2E	POLR2F	POLR2G	POLR2H	POLR2I	POLR2J

U2AF2								
STH	THRAP3	TRA2A	TRA2B					
MAGOH	MBNL1	MBNL2	MBNL3	MYOD1	NOVA1	NOVA2	NSRP1	PTBP1
HNRNPA1	HNRNPA2	HNRNPK	HNRNPL	HNRNPU	HSPA8	JMJD6	KHDRBS1	KHDRBS2

CELF4	CELF5	CELF6	CHERP	CIRBP	CLNS1A	CLP1	COIL	CPSF1
LUC7L3	NCBP1	NOL3	OVAAL	PRPF19	PRPF39	PRPF6	PSIP1	PTBP2

RNU6-9	RNU6ATA(	SART3	SRSF10	SRSF12	USP4			
ESS2	FRG1	GPATCH1	HNRNPA1	HNRNPA2	HNRNPA3	HNRNPC	HNRNPF	HNRNPH1
UPF3B								

CWC15	CWC22	CWC25	CWC27	CWF19L1	CWF19L2	DDX23	DDX39B	DDX41
SNRPB2	SNRPD1	SNRPD2	SNRPD3	SNRPE	SNRPF	SNRPG	SNW1	SRRM2
GCFC2	HTATSF1	IK	ISY1	LSM2	LSM3	LSM4	LSM5	LSM6
SNRPD2	SNRPD3	SNRPE	SNRPF	SNRPG	SNRPGP15	YBX1	ZCRB1	ZMAT5

DHX38	DHX8	EFTUD2	EIF4A3	HNRNPA1	HNRNPA1	HNRNPA1	HNRNPA3	HNRNPC
GTF2H1	HYAL1	LGR6	MAP2K5	MAPT	NAE1	NLRP1	NR1H3	PCDHA12
DAP3	DDR1	ELN	F2R	FASTK	FAT1	FZD1	GAS2	GRK3
POLR2I	POLR2J	POLR2K	POLR2L	PTBP1	RBFOX2	TIA1	TIAL1	
CSTF2T	CSTF3	CTNNBL1	CWC15	CWC22	CWC25	CWC27	DDX23	DDX42
POLR2K	POLR2L	PRPF6	PRPF8	RNPC3	SF3B1	SF3B2	SF3B3	SF3B4

PUF60	RBFOX1	RBFOX2	RBFOX3	RBM10	RBM11	RBM15	RBM15B	RBM24
KHDRBS3	LARP7	MAGOH	MBNL1	MBNL2	MBNL3	METTL16	MYOD1	NCBP1
CPSF2	CPSF3	CPSF4	CPSF7	CRNKL1	CSTF1	CSTF2	CSTF2T	CSTF3
PUF60	RBM5	RBMX	RNU1-4	RNU11	RNU2-1	RNU4ATA(	RNU6ATA(	RNVU1-1
HNRNPK	HNRNPM	HNRNPR	HNRNPU	ISY1	LSM2	LSM3	LSM7	MAGOH
DDX5	DHX15	DHX16	DHX32	DHX35	DHX38	DHX8	DQX1	EFTUD2
SYF2	XAB2							
LSM7	LSM8	LUC7L	LUC7L2	LUC7L3	MAGOHB	MFAP1	PHF5A	PLRG1
ZRSR2								
HNRNPK	HNRNPM	HNRNPU	HSPA1A	HSPA1B	HSPA1L	HSPA2	HSPA6	HSPA8
PCDHA5	PCDHAC2	PCDHGA1:	PDCD2	PHLDA2	PPARD	PTK2	SEMA3B	SLITRK4
HAPLN3	HIPK2	IL12RB1	IL3RA	ITGB5	L1CAM	LGALS1	LGALS3BP	LGI3
DDX46	DDX5	DHX15	DHX16	DHX38	DHX9	DNAJC8	EFTUD2	EIF4A3
SF3B5	SF3B6	SNRNP20(	SNRNP25	SNRNP35	SNRNP40	SNRNP48	SNRPB	SNRPD1

RBM25	RBM4	RBM5	RBM7	RBM8A	RBMX	RBMY1A1	REST	RNPS1
NCL	NOVA1	NOVA2	NSRP1	NUP98	PCBP4	PRDX6	PRPF19	PTBP1
CTNNBL1	CWC15	CWC22	CWC25	CWC27	CWF19L1	CWF19L2	DAZAP1	DBR1
RNVU1-14	RNVU1-15	RNVU1-17	RNVU1-19	RNVU1-2A	RNVU1-3	RNVU1-4	RNVU1-6	RNVU1-7
MAGOHB	MTREX	PABPC1	PLRG1	PNN	PPIE	PPIL1	PPIL3	PPWD1
EIF4A3	ESS2	FRG1	GCFC2	GEMIN2	GPATCH1	GPKOW	HNRNPA1	HNRNPA1
PPIE	PPIL1	PRPF18	PRPF19	PRPF3	PRPF31	PRPF38A	PRPF39	PRPF4
ISY1	LSM2	LSM3	LSM4	LSM5	LSM6	LSM7	LSM8	MAGOH
SOD1	SPP1	SVIL	TNFSF10	TSPAN5	TTBK1	UBP1	WDR77	
LRRC66	LTBR	LZTR1	MAP4K4	MCM7	MET	MFAP5	MGP	NAIP
ELAVL1	ELAVL2	FIP1L1	FUS	GCFC2	GPKOW	GTF2F1	GTF2F2	HNRNPA0
SNRPD2	SNRPD3	SNRPE	SNRPF	SNRPG	SNU13	SRSF1	SRSF2	SRSF6

SAP18	SMU1	SRRM4	SRSF12	SRSF6	THRAP3	TRA2B	WTAP	YTHDC1
PUF60	QKI	RBFOX1	RBFOX2	RBFOX3	RBM10	RBM11	RBM15	RBM15B
DCPS	DDX1	DDX17	DDX20	DDX23	DDX39A	DDX39B	DDX41	DDX42
RNVU1-8	SCAF11	SETX	SF1	SF3A1	SF3A2	SF3A3	SF3B1	SFSWAP
PRPF19	PRPF4B	PRPF6	PRPF8	RALY	RBM22	RBM8A	RBMX	SART1
HNRNPA2	HNRNPA3	HNRNPC	HNRNPF	HNRNPH1	HNRNPH3	HNRNPK	HNRNPM	HNRNPR
PRPF40A	PRPF40B	PRPF6	PRPF8	RBM22	RBM8A	RBMX2	RNF113A	RNF113B
MAGOHB	NCBP1	NCBP2	PCBP1	PHF5A	PLRG1	PPIE	PPIH	PPIL1
NLGN4X	NME6	NRCAM	P3H3	PDCD6IP	PIM1	PLAGL1	PLXNA2	PLXNB3
HNRNPA1	HNRNPA2	HNRNPA3	HNRNPC	HNRNPD	HNRNPF	HNRNPH1	HNRNPH2	HNRNPK
SRSF7	TXNL4A	YBX1	ZCRB1	ZMAT5	ZRSR2			

ZBTB7A	ZC3H10							
RBM23	RBM24	RBM25	RBM3	RBM39	RBM4	RBM42	RBM5	RBM7
DDX46	DDX5	DHX15	DHX16	DHX35	DHX38	DHX8	DHX9	DNAJC8
SLU7	SMN1	SMN2	SNRNP200	SNRPC	SNRPD1	SNRPD2	SNRPE	SNRPG
SF3A1	SF3A2	SF3A3	SF3B1	SF3B2	SF3B3	SF3B6	SLU7	SNRNP200
HNRNPU	HSPA8	HTATSF1	IK	ISY1	IVNS1ABP	KHDC4	LGALS3	LSM2
SART1	SF3A1	SF3A2	SF3A3	SF3B1	SF3B2	SF3B3	SF3B4	SF3B5
PQBP1	PRPF18	PRPF19	PRPF3	PRPF31	PRPF38A	PRPF38B	PRPF4	PRPF40A
PPP2R1A	PTK7	PTPRF	PTPRM	RPS6KL1	RTN4	SH2D4A	SHC1	SLC1A4
HNRNPL	HNRNPM	HNRNPR	HNRNPU	HNRNPUL1	HSPA8	ISY1	LSM2	LSM3

RBM8A	RBMX	RBMXL1	RBMY1A1	RBMY1B	REST	RNPS1	SAP18	SF1
DYRK1A	EFTUD2	EIF4A3	ELAVL1	ELAVL2	ESRP2	ESS2	FAM172A	FIP1L1
SRPK1	SRPK2	SRPK3	SRSF1	SRSF10	SRSF12	SRSF5	SRSF6	SRSF9
SNRNP40	SNRPA1	SNRPB	SNRPB2	SNRPD1	SNRPD2	SNRPD3	SNRPE	SNRPF
LSM3	LSM4	LSM5	LSM6	LSM7	LSM8	LUC7L	LUC7L2	LUC7L3
SF3B6	SMU1	SNIP1	SNRNP20C	SNRNP40	SNRNP70	SNRPA1	SNRPB	SNRPB2
PRPF40B	PRPF6	PRPF8	PUF60	RBM17	RBM22	RBM25	RBM8A	RBMX
SMAD3	SMO	SRPK2	STAB2	STK32C	TMEM45A	TNFRSF1A	TSPAN33	TSPAN9
LSM4	LSM5	LSM6	LSM7	LSM8	MAGOH	MAGOHB	MTREX	NCBP1

SF3B4	SFSWAP	SLC39A5	SMU1	SNRNP70	SNW1	SON	SRPK1	SRPK2
FMR1	FRG1	FUS	FXR1	FXR2	GCFC2	GEMIN2	GEMIN4	GEMIN5
TXNL4A	USP39	WEE2-AS1	YTHDC1	ZRSR2				
SNRPG	SNRPGP15	SNRPN	SNW1	SRRM1	SRRM2	SRSF1	SYF2	SYNCRIP
MAGOH	MAGOHB	MFAP1	MTREX	MYEF2	NCL	PABPC1	PDCD7	PHF5A
SNRPC	SNRPD1	SNRPD2	SNRPD3	SNRPE	SNRPF	SNRPG	SNRPGP15	SNRPN
SART1	SF3A1	SF3A2	SF3A3	SF3B1	SF3B2	SF3B3	SF3B4	SF3B5
WNK1								
NCBP2	NUDT21	PABPN1	PAPOLA	PCBP1	PCBP2	PCF11	PDCD7	PHF5A

SRRM4	SRSF10	SRSF12	SRSF3	SRSF4	SRSF6	SRSF7	SRSF9	STH
GEMIN6	GEMIN7	GEMIN8	GPATCH1	GPKOW	GTF2F1	GTF2F2	HMX2	HNRNPA0

TFIP11	U2AF1	WDR83	XAB2	ZCCHC8

PLRG1	PNN	PPIE	PPIH	PPIL1	PPIL3	PPP1R8	PPWD1	PRKRIP1
SNU13	SNW1	SRRM2	SYF2	TFIP11	TXNL4A	U2AF2	WBP4	XAB2

SF3B6	SLU7	SMNDC1	SNRNP20(	SNRNP27	SNRNP40	SNRNP70	SNRPA	SNRPA1

PLRG1	POLR2A	POLR2B	POLR2C	POLR2D	POLR2E	POLR2F	POLR2G	POLR2H

THRAP3	TIA1	TRA2A	TRA2B	U2AF2	WTAP	YTHDC1	ZBTB7A	ZC3H10
HNRNPA1	HNRNPA2	HNRNPA3	HNRNPC	HNRNPD	HNRNPF	HNRNPH1	HNRNPH2	HNRNPH3

PRPF18	PRPF19	PRPF3	PRPF31	PRPF38A	PRPF38B	PRPF39	PRPF4	PRPF40A
YJU2	ZMAT2							

SNRPB	SNRPB2	SNRPC	SNRPD1	SNRPD2	SNRPD3	SNRPE	SNRPF	SNRPG

POLR2I	POLR2J	POLR2K	POLR2L	PPIE	PPIH	PPIL1	PPIL3	PPIL4

HNRNPK	HNRNPL	HNRNPM	HNRNPR	HNRNPU	HNRNPUL1	HSPA8	HTATSF1	IK
PRPF40B	PRPF4B	PRPF6	PRPF8	PTBP2	RALY	RBM17	RBM22	RBM28
SNU13	SNW1	SRSF1	SRSF10	SRSF2	SRSF3	SRSF4	SRSF5	SRSF6
PPIL6	PPWD1	PQBP1	PRCC	PRPF19	PRPF3	PRPF31	PRPF38A	PRPF4

ISY1	JMJD6	KDM1A	KHDC4	KHDRBS1	KHDRBS2	KHDRBS3	KHSRP	LARP7
RBM3	RBM41	RBM5	RBM8A	RBMX	RBMX2	RBMXL1	RBMXL2	RBMXL3
SRSF7	SRSF8	SRSF9	SYF2	TCERG1	THOC1	THOC2	THOC3	TRA2A
PRPF40A	PRPF6	PRPF8	PTBP1	PUF60	RBM17	RBM22	RBM5	RBM8A

LINC0171[illegible]	LSM1	LSM2	LSM3	LSM4	LSM5	LSM6	LSM7	LSM8
RBMY1B	RHEB	RNF113A	RNF113B	RNPC3	SART1	SF1	SF3A1	SF3A2
TRA2B	TXNL4A	U2AF1	U2AF2	U2SURP	USP39	WBP11	XAB2	ZMAT2
RBMX	RNPC3	RNPS1	SART1	SF1	SF3A1	SF3A2	SF3A3	SF3B1

LUC7L	LUC7L2	LUC7L3	MAGOH	MAGOHB	MBNL1	MBNL2	MBNL3	METTL14
SF3A3	SF3B1	SF3B2	SF3B3	SF3B4	SF3B5	SF3B6	SLU7	SMNDC1
SF3B2	SF3B3	SF3B4	SF3B5	SF3B6	SLU7	SMNDC1	SNRNP200	SNRNP25

METTL16	METTL3	MFAP1	MPHOSPH	MTREX	MYOD1	NCBP1	NCBP2	NCBP2L
SMU1	SNIP1	SNRNP200	SNRNP25	SNRNP35	SNRNP40	SNRNP48	SNRNP70	SNRPA
SNRNP27	SNRNP35	SNRNP40	SNRNP48	SNRNP70	SNRPA	SNRPA1	SNRPB	SNRPB2

NCL	NOL3	NONO	NOVA1	NOVA2	NSRP1	NUDT21	NUP98	OVAAL
SNRPA1	SNRPB	SNRPB2	SNRPC	SNRPD1	SNRPD2	SNRPD3	SNRPE	SNRPF
SNRPC	SNRPD1	SNRPD2	SNRPD3	SNRPE	SNRPF	SNRPG	SNRPN	SNU13

PABPC1	PABPN1	PAPOLA	PCBP1	PCBP2	PCBP4	PCF11	PDCD7	PHF5A
SNRPG	SNRPGP15	SNRPN	SNU13	SNW1	SREK1	SRRM1	SRRM2	SRSF1
SNW1	SRRM1	SRRM2	SRRT	SRSF1	SRSF10	SRSF11	SRSF2	SRSF3

PLRG1	PNN	POLR2A	POLR2B	POLR2C	POLR2D	POLR2E	POLR2F	POLR2G
SUGP1	SYF2	SYNCRIP	TFIP11	TRA2A	TRA2B	TTF2	TXNL4A	TXNL4B
SRSF4	SRSF5	SRSF6	SRSF7	SRSF9	SUGP1	SYF2	SYMPK	TFIP11

POLR2H	POLR2I	POLR2J	POLR2K	POLR2L	PPIE	PPIH	PPIL1	PPIL3
U2AF1	U2AF1L4	U2AF1L5	U2AF2	UPF1	USP39	WBP11	WBP4	WDR83
TRA2B	TXNL4A	U2AF1	U2AF1L4	U2AF2	U2SURP	UPF3B	USP39	WBP11

PPWD1	PQBP1	PRCC	PRDX6	PRMT5	PRMT7	PRPF18	PRPF19	PRPF3
XAB2	YBX1	YJU2	ZCCHC8	ZCRB1	ZMAT2	ZMAT5	ZNF830	ZRSR2
WBP4	WDR33	XAB2	YBX1	ZCRB1	ZMAT5	ZRSR2		

PRPF31	PRPF38A	PRPF39	PRPF4	PRPF40A	PRPF40B	PRPF4B	PRPF6	PRPF8

ZRSR2P1

PSIP1	PSPC1	PTBP1	PTBP2	PUF60	QKI	RALY	RAVER1	RAVER2

RBFOX1	RBFOX2	RBFOX3	RBM10	RBM11	RBM14	RBM15	RBM15B	RBM17

RBM19	RBM22	RBM23	RBM24	RBM25	RBM3	RBM39	RBM4	RBM41

RBM42	RBM4B	RBM5	RBM6	RBM7	RBM8A	RBMX	RBMX2	RBMXL1

RBMXL2	RBMXL3	RBMY1A1	RBMY1B	RBMY1F	RBMY1J	REST	RNF113A	RNPC3

RNPS1	RNU1-4	RNU11	RNU2-1	RNU4-1	RNU4-2	RNU4ATA(	RNU5A-1	RNU5B-1

RNU5D-1	RNU5E-1	RNU5F-1	RNU6-1	RNU6-7	RNU6-9	RNU6ATA(	RNVU1-1	RNVU1-14

RNVU1-15	RNVU1-17	RNVU1-19	RNVU1-2A	RNVU1-3	RNVU1-4	RNVU1-6	RNVU1-7	RNVU1-8

RSRC1	SAP18	SART1	SART3	SCAF11	SCNM1	SETX	SF1	SF3A1

SF3A2	SF3A3	SF3B1	SF3B2	SF3B3	SF3B4	SF3B5	SF3B6	SFPQ

SFSWAP	SLC39A5	SLU7	SMN1	SMN2	SMNDC1	SMU1	SNIP1	SNRNP20C

SNRNP25	SNRNP27	SNRNP35	SNRNP40	SNRNP48	SNRNP70	SNRPA	SNRPA1	SNRPB

SNRPB2	SNRPC	SNRPD1	SNRPD2	SNRPD3	SNRPE	SNRPF	SNRPG	SNRPGP15

SNRPN	SNU13	SNUPN	SNW1	SON	SPEN	SRPK1	SRPK2	SRPK3

SRRM1	SRRM2	SRRM4	SRRT	SRSF1	SRSF10	SRSF11	SRSF12	SRSF2

SRSF3	SRSF4	SRSF5	SRSF6	SRSF7	SRSF8	SRSF9	STH	STRAP

SUGP1	SYF2	SYMPK	SYNCRIP	TFIP11	TGS1	THRAP3	TIA1	TRA2A

TRA2B	TXNL4A	TXNL4B	U2AF1	U2AF1L4	U2AF1L5	U2AF2	U2SURP	UBL5

UPF3B	USP39	USP4	USP49	WBP11	WBP4	WDR33	WDR77	WDR83

WEE2-AS1	WTAP	XAB2	YBX1	YJU2	YTHDC1	ZBTB7A	ZC3H10	ZCCHC8

ZCRB1	ZMAT2	ZMAT5	ZRSR2	ZRSR2P1

REACTOM	-0.68075	0.249599	-10.2039	2.40E-20	5.03E-19	35.54043	down
HOWARD_	0.679329	-0.25942	9.520764	2.78E-18	3.62E-17	30.85451	up
HOWARD_	-0.67898	0.049452	-11.2053	1.85E-23	8.33E-22	42.61093	down
GOBP_PRE	0.67856	-0.19888	9.56978	1.99E-18	2.67E-17	31.18636	up
GNF2_SM(	0.678268	-0.23663	9.713446	7.37E-19	1.11E-17	32.16305	up
GOBP_NE(	-0.67618	0.092556	-8.94671	1.36E-16	1.27E-15	27.0242	down
GNF2_CCN	0.675728	-0.2459	9.700891	8.04E-19	1.20E-17	32.07746	up
GNF2_CCN	0.675655	-0.25313	9.593039	1.69E-18	2.32E-17	31.34407	up
GOBP_DOI	0.675303	-0.15709	9.826461	3.37E-19	5.47E-18	32.93551	up
HP_DECRE	-0.67398	0.269682	-10.8836	1.89E-22	6.62E-21	40.3171	down
GOMF_IMI	-0.67314	0.033773	-9.08678	5.32E-17	5.38E-16	27.94888	down
SEMBA_FH	0.672867	-0.23804	9.749219	5.75E-19	8.91E-18	32.40717	up
GOBP_DOI	0.672375	-0.21977	9.700012	8.09E-19	1.20E-17	32.07147	up
GNF2_CEN	0.671692	-0.24813	9.674724	9.63E-19	1.40E-17	31.89922	up
BIOCARTA	-0.67106	0.036205	-10.4932	3.09E-21	8.12E-20	37.56054	down
GNF2_ESPI	0.671001	-0.24655	9.608055	1.53E-18	2.12E-17	31.44598	up
ZHAN_MU	0.67024	-0.23732	10.13684	3.84E-20	7.64E-19	35.07474	up
MODULE_:	0.669857	-0.20528	11.57976	1.21E-24	7.44E-23	45.3047	up
GNF2_CD(	0.668777	-0.25701	9.656855	1.09E-18	1.57E-17	31.77761	up
GNF2_CD(	0.668689	-0.2482	9.600383	1.61E-18	2.22E-17	31.3939	up
KUMAMO	0.667433	-0.2966	9.307804	1.19E-17	1.37E-16	29.42123	up
GOBP_NE(	-0.66734	0.028418	-11.4345	3.49E-24	1.89E-22	44.25704	down
GNF2_TTK	0.66525	-0.25318	9.468963	3.97E-18	4.99E-17	30.5046	up
GNF2_H2A	0.664817	-0.25574	9.483261	3.60E-18	4.57E-17	30.6011	up
GOBP_CEL	0.662603	-0.20427	9.543197	2.38E-18	3.15E-17	31.0063	up
GNF2_BUB	0.662082	-0.2576	9.807251	3.85E-19	6.16E-18	32.80396	up
MODULE_:	0.660711	-0.10981	9.110015	4.55E-17	4.67E-16	28.10291	up
GRAHAM_	-0.6607	-0.0246	-11.5647	1.35E-24	8.19E-23	45.19588	down
HP_EPISOI	0.659922	-0.03149	12.02738	4.50E-26	4.06E-24	48.55256	up
GNF2_CEN	0.657948	-0.25644	9.439134	4.87E-18	6.02E-17	30.30347	up
GOMF_HY	-0.65792	0.014953	-11.0014	8.08E-23	3.12E-21	41.15488	down
GOMF_RN.	0.657691	-0.2427	9.7064	7.74E-19	1.16E-17	32.11501	up
GOCC_FIBI	-0.65731	0.197872	-10.5329	2.33E-21	6.27E-20	37.83969	down
GOCC_ALF	0.656622	-0.23488	8.541177	1.99E-15	1.51E-14	24.38639	up
GOBP_NE(	-0.65576	-0.00024	-10.5759	1.71E-21	4.78E-20	38.14156	down
GOBP_NEU	-0.65358	0.169123	-10.9364	1.29E-22	4.74E-21	40.69207	down
REACTOM	-0.65253	0.118214	-11.2419	1.42E-23	6.50E-22	42.87311	down
GNF2_PCN	0.651921	-0.24445	9.561106	2.11E-18	2.82E-17	31.12758	up
GOBP_REL	-0.65134	0.083234	-12.2333	9.82E-27	1.09E-24	50.05558	down
GOBP_DE_	0.651218	-0.14539	9.79196	4.28E-19	6.78E-18	32.69931	up
REACTOM	-0.65104	0.100473	-10.3706	7.38E-21	1.77E-19	36.70207	down
GOMF_FO	0.649917	-0.11904	11.53206	1.71E-24	9.93E-23	44.96029	up
GLINSKY_(	0.649882	-0.19557	11.347	6.60E-24	3.31E-22	43.62769	up
GOMF_ALI	-0.64954	0.258113	-9.78628	4.45E-19	7.03E-18	32.66047	down
GOBP_AUT	-0.64888	-0.03456	-12.1854	1.40E-26	1.45E-24	49.70527	down
REACTOM	-0.64455	0.086602	-11.663	6.56E-25	4.34E-23	45.90652	down
GOCC_ME	-0.64304	0.246664	-10.4902	3.16E-21	8.28E-20	37.53944	down
REACTOM	-0.64283	0.23465	-10.59	1.55E-21	4.38E-20	38.24129	down
AMIT_DEL/	-0.64221	0.070463	-11.1524	2.71E-23	1.19E-21	42.23257	down
GOMF_FLA	0.641888	-0.09319	12.31298	5.44E-27	6.60E-25	50.63857	up
FARMER_B	0.641753	-0.21207	10.324	1.03E-20	2.38E-19	36.37657	up
MIR3683	-0.64159	0.071606	-12.5236	1.14E-27	1.72E-25	52.1831	down
REACTOM	0.640973	-0.12181	11.94672	8.15E-26	6.87E-24	47.9653	up
REACTOM	0.64008	-0.24402	8.774545	4.28E-16	3.67E-15	25.89706	up
HP_THIN_(	0.639626	-0.08911	11.72704	4.10E-25	2.88E-23	46.37017	up
MIR551A_I	-0.63952	-0.01116	-10.4886	3.19E-21	8.33E-20	37.52864	down
WP_MIR51	-0.63905	0.046366	-11.6929	5.27E-25	3.58E-23	46.12327	down
GOBP_NE(	-0.63885	0.041487	-9.92825	1.66E-19	2.87E-18	33.63421	down

REACTOM	-0.63877	0.102712	-12.4959	1.40E-27	2.02E-25	51.97937	down
GOMF_INT	-0.63597	0.144762	-12.0109	5.08E-26	4.53E-24	48.43275	down
GOMF_DN	0.635936	-0.2083	9.588941	1.74E-18	2.38E-17	31.31627	up
GNF2_MCI	0.634701	-0.25407	9.288483	1.36E-17	1.54E-16	29.2919	up
ZHONG_PI	0.633851	-0.21788	10.38964	6.45E-21	1.57E-19	36.83511	up
KANG_DO	0.633458	-0.2482	9.447304	4.60E-18	5.72E-17	30.35853	up
MODULE_	-0.63191	0.100709	-14.614	1.75E-34	1.66E-31	67.67827	down
GOBP_PLU	0.63072	0.062827	11.2955	9.60E-24	4.65E-22	43.25782	up
GOCC_CM	0.630589	-0.24603	8.503143	2.55E-15	1.91E-14	24.14213	up
GOCC_MIS	0.630196	-0.00034	10.66232	9.24E-22	2.76E-20	38.75067	up
GOBP_EST	-0.62844	0.058865	-10.9523	1.15E-22	4.30E-21	40.80554	down
BOYAULT_	-0.6273	0.051149	-10.5047	2.85E-21	7.54E-20	37.64133	down
HP_WIDEN	-0.62688	0.175049	-8.34641	7.06E-15	4.92E-14	23.14146	down
GOBP_POS	0.626399	-0.10347	12.49591	1.40E-27	2.02E-25	51.97951	up
GOBP_ATR	-0.62548	0.15721	-12.0506	3.79E-26	3.51E-24	48.72167	down
GOMF_MH	-0.62536	0.012591	-9.32649	1.05E-17	1.22E-16	29.54644	down
GOBP_POS	0.625196	-0.14684	11.84809	1.69E-25	1.31E-23	47.2483	up
GOMF_FU	-0.62516	0.148855	-11.0615	5.24E-23	2.14E-21	41.58344	down
NAGASHIN	-0.62513	0.049146	-12.046	3.92E-26	3.60E-24	48.68835	down
GAO_LAR	0.624751	-0.22919	9.696677	8.28E-19	1.23E-17	32.04874	up
GNF2_CD1	-0.62435	0.059449	-10.9181	1.47E-22	5.32E-21	40.56169	down
WP_AMIN	-0.62432	0.201504	-10.6503	1.01E-21	2.99E-20	38.66562	down
GOCC_DN	0.622923	-0.23627	8.611494	1.25E-15	9.89E-15	24.83944	up
GOCC_PR	-0.62253	0.213181	-7.73823	3.34E-13	1.80E-12	19.35462	down
REACTOM	-0.62253	0.213181	-7.73823	3.34E-13	1.80E-12	19.35462	down
GOBP_DO	0.621834	-0.21954	9.546001	2.34E-18	3.09E-17	31.02528	up
GNF2_CD1	-0.62154	0.043848	-10.324	1.03E-20	2.38E-19	36.37627	down
GOBP_NE	-0.62153	0.008966	-8.80719	3.45E-16	3.00E-15	26.10996	down
GOBP_CAL	0.621105	0.067113	11.40501	4.32E-24	2.28E-22	44.04479	up
SOUCEK_N	-0.62086	0.084773	-10.2414	1.84E-20	3.99E-19	35.80101	down
GOBP_POS	-0.62064	0.078551	-11.7471	3.54E-25	2.52E-23	46.51521	down
GOBP_REG	0.620564	0.045598	8.223937	1.55E-14	1.02E-13	22.36636	up
WP_MACR	-0.61977	0.018589	-9.17681	2.90E-17	3.10E-16	28.54668	down
GOBP_ASP	-0.61959	0.211109	-10.28	1.40E-20	3.14E-19	36.06985	down
BIOCARTA	-0.61933	0.203727	-10.7869	3.79E-22	1.25E-20	39.63118	down
GOBP_BRA	-0.61904	-0.03031	-14.2757	2.24E-33	1.68E-30	65.15841	down
RARB_TAR	-0.6186	0.186919	-7.90731	1.16E-13	6.69E-13	20.39143	down
GOCC_EXT	-0.61821	0.136922	-10.6175	1.27E-21	3.67E-20	38.43487	down
REICHERT_	0.617431	-0.20242	10.15468	3.39E-20	6.84E-19	35.19846	up
GOMF_GLY	-0.61697	0.210187	-11.5007	2.15E-24	1.21E-22	44.73423	down
ZNF302_T/	0.616966	-0.10592	9.635519	1.26E-18	1.79E-17	31.63253	up
DESCARTE	-0.61618	0.134429	-13.7806	9.34E-32	4.64E-29	61.47599	down
GOBP_REG	-0.61604	-0.01865	-7.60232	7.75E-13	3.98E-12	18.53062	down
NIKOLSKY_	0.615734	-0.12168	11.43346	3.51E-24	1.90E-22	44.24958	up
CAO_BLO	-0.61541	0.076314	-10.2075	2.34E-20	4.92E-19	35.56546	down
HP_SLOWI	0.615396	0.052596	11.94543	8.23E-26	6.88E-24	47.95585	up
GOCC_ENI	-0.61478	0.037824	-9.53841	2.46E-18	3.24E-17	30.97389	down
MT	-0.61413	0.18485	-8.40205	4.92E-15	3.51E-14	23.49561	down
REACTOM	0.614106	-0.15214	10.83669	2.65E-22	8.96E-21	39.98405	up
LOPEZ_ME	-0.61256	0.199261	-11.2528	1.31E-23	6.05E-22	42.95174	down
WP_COMF	-0.6122	0.192351	-11.6896	5.40E-25	3.64E-23	46.09889	down
GNF2_CKS	0.611766	-0.25035	9.433642	5.05E-18	6.22E-17	30.26647	up
GNF2_BUB	0.611137	-0.24799	9.118997	4.28E-17	4.42E-16	28.1625	up
GOCC_PR	-0.61028	0.178867	-8.11024	3.22E-14	2.01E-13	21.65232	down
REACTOM	0.610144	0.043682	9.500072	3.21E-18	4.12E-17	30.71464	up
GNF2_IGF1	-0.61009	0.222631	-12.5067	1.29E-27	1.90E-25	52.05892	down
GOCC_CO	0.609911	-0.19177	9.293592	1.31E-17	1.49E-16	29.32608	up
REACTOM	-0.60982	0.02152	-9.77609	4.78E-19	7.51E-18	32.59077	down

GOBP_ASS	0.608981	-0.17368	9.476202	3.78E-18	4.77E-17	30.55345	up
BIOCARTA	-0.60838	0.203142	-11.3995	4.50E-24	2.37E-22	44.00491	down
GOBP_REG	0.607988	-0.12463	12.19587	1.29E-26	1.37E-24	49.782	up
GOCC_ACT	-0.60795	0.05726	-11.9626	7.25E-26	6.19E-24	48.08065	down
GOBP_CEL	-0.60769	0.091607	-9.7112	7.49E-19	1.12E-17	32.14771	down
GOBP_NEG	-0.60662	0.096977	-12.4055	2.74E-27	3.63E-25	51.31662	down
LY_AGING_	0.606598	-0.27027	8.992826	1.00E-16	9.61E-16	27.32792	up
MODULE_3	0.606545	-0.19166	10.70488	6.82E-22	2.10E-20	39.05112	up
GOBP_LUN	-0.60636	0.124656	-11.2892	1.00E-23	4.83E-22	43.21238	down
GOBP_DET	-0.60609	0.149576	-10.9768	9.65E-23	3.67E-21	40.97953	down
GOMF_C_X	-0.60585	0.084105	-10.1017	4.92E-20	9.59E-19	34.83114	down
GOMF_BET	0.605328	0.021453	11.10255	3.89E-23	1.64E-21	41.87628	up
GOBP_SPIN	0.604493	-0.15956	10.38248	6.78E-21	1.64E-19	36.78509	up
GOBP_REG	-0.60449	0.02464	-8.92169	1.61E-16	1.48E-15	26.8598	down
GOBP_POS	-0.60372	0.094948	-11.0052	7.87E-23	3.05E-21	41.18152	down
GOBP_MO	-0.60338	0.248424	-10.0446	7.34E-20	1.38E-18	34.43609	down
GOBP_COI	-0.60237	0.159127	-11.0226	6.93E-23	2.73E-21	41.30609	down
ISHIDA_E2	0.601153	-0.24971	9.571608	1.96E-18	2.65E-17	31.19874	up
GOBP_LEU	-0.60096	0.277384	-9.96576	1.27E-19	2.27E-18	33.89247	down
GOCC_DN	0.60077	-0.19822	8.609393	1.27E-15	1.00E-14	24.82587	up
ERWIN_CO	-0.60074	0.058374	-9.23899	1.90E-17	2.10E-16	28.96115	down
GOBP_REG	-0.60048	-0.02977	-8.11285	3.16E-14	1.98E-13	21.6686	down
WANG_ME	0.599744	-0.17996	11.26859	1.17E-23	5.52E-22	43.06476	up
GOBP_MIT	0.599381	-0.1947	9.62046	1.40E-18	1.96E-17	31.53021	up
WP_ARACI	-0.59925	0.139145	-11.598	1.06E-24	6.65E-23	45.43664	down
GOMF_KIN	0.59757	-0.20473	9.993857	1.05E-19	1.90E-18	34.08609	up
GOBP_REG	0.59756	-0.00506	11.33394	7.26E-24	3.62E-22	43.53381	up
GOBP_LEU	-0.59733	0.072229	-11.0234	6.89E-23	2.71E-21	41.31177	down
HP_DECRE	-0.59687	0.015071	-8.92518	1.57E-16	1.45E-15	26.88268	down
GOBP_PEP	-0.59679	0.113684	-9.20462	2.40E-17	2.59E-16	28.73188	down
GOCC_MU	0.59671	0.142163	8.367776	6.15E-15	4.32E-14	23.27731	up
GESERICK_	-0.59655	0.1179	-10.7963	3.54E-22	1.17E-20	39.69806	down
AMIT_SERU	-0.59655	0.099532	-13.2722	4.25E-30	1.25E-27	57.70446	down
GOMF_CO	-0.59634	0.052025	-8.54512	1.94E-15	1.48E-14	24.41173	down
LI_WILMS_	0.595416	-0.21622	10.5525	2.02E-21	5.55E-20	37.97725	up
HP_LACTIC	-0.59519	0.115266	-11.2856	1.03E-23	4.95E-22	43.18709	down
GOBP_EPIT	-0.59472	0.107254	-11.0486	5.75E-23	2.33E-21	41.49095	down
CASP3_TAI	-0.59441	0.223448	-7.7625	2.87E-13	1.56E-12	19.50264	down
GOMF_MH	-0.59375	0.034693	-10.2267	2.04E-20	4.38E-19	35.69903	down
GOCC_AST	-0.59375	0.120865	-10.1333	3.94E-20	7.81E-19	35.05039	down
REACTOM	-0.5935	0.265688	-9.80056	4.03E-19	6.42E-18	32.75817	down
GOBP_KYN	-0.59319	0.218412	-11.1341	3.10E-23	1.34E-21	42.10161	down
WP_HFE_E	-0.59277	0.150207	-10.3649	7.68E-21	1.83E-19	36.66185	down
HP_HYPER	-0.59269	0.210512	-9.81261	3.71E-19	5.95E-18	32.84066	down
REACTOM	-0.59259	0.236003	-8.39165	5.27E-15	3.74E-14	23.42928	down
GOBP_REC	-0.59208	0.088263	-12.0764	3.13E-26	3.02E-24	48.91008	down
HP_DEEP_S	0.59206	-0.24133	9.931291	1.62E-19	2.82E-18	33.65516	up
GOMF_AC	-0.59163	0.259182	-9.68688	8.86E-19	1.30E-17	31.98198	down
HP_LYMPH	-0.59098	0.209147	-11.7674	3.05E-25	2.19E-23	46.6624	down
GOMF_INS	-0.59075	0.126116	-11.9817	6.30E-26	5.45E-24	48.21968	down
DESCARTE	-0.59066	0.032313	-9.2979	1.28E-17	1.45E-16	29.35489	down
GRAHAM_	-0.59059	0.027637	-11.2048	1.86E-23	8.35E-22	42.60724	down
MODULE_1	-0.59031	0.102199	-10.4251	5.01E-21	1.26E-19	37.08333	down
GOMF_IGG	-0.5903	0.003456	-10.3065	1.16E-20	2.66E-19	36.25451	down
HP_RECUR	-0.59017	0.286299	-9.54144	2.41E-18	3.18E-17	30.9944	down
WP_GASTF	0.589867	-0.16461	11.53595	1.66E-24	9.68E-23	44.98835	up
ZHENG_RE	-0.58976	0.120965	-10.5652	1.85E-21	5.13E-20	38.06648	down
GOBP_HIST	-0.58904	0.18813	-11.5098	2.01E-24	1.14E-22	44.80002	down

GOBP_POS	0.58899	-0.08122	9.624998	1.36E-18	1.91E-17	31.56104	up
REACTOM	0.588792	-0.23756	9.688531	8.76E-19	1.29E-17	31.99324	up
HP_LOW_F	-0.58843	0.224177	-9.24943	1.77E-17	1.97E-16	29.03081	down
GOBP_REL	-0.58796	0.071222	-10.8011	3.42E-22	1.13E-20	39.73181	down
DURANTE_	-0.58744	-0.0088	-9.69386	8.44E-19	1.25E-17	32.02956	down
CHEOK_RE	0.587091	0.028955	11.42892	3.63E-24	1.95E-22	44.21692	up
GOBP_MIT	0.586833	-0.14538	9.932659	1.61E-19	2.80E-18	33.66458	up
GOBP_REG	-0.58669	0.049954	-9.37984	7.30E-18	8.70E-17	29.90449	down
REACTOM	-0.58636	0.243915	-11.6055	1.00E-24	6.32E-23	45.49063	down
HP_DECRE	-0.5863	0.14443	-8.86296	2.38E-16	2.13E-15	26.47458	down
GOCC_CO	0.585632	-0.26709	8.35109	6.85E-15	4.78E-14	23.17121	up
HP_HYPOC	0.585202	-0.12118	11.0878	4.33E-23	1.81E-21	41.77099	up
GOMF_CX(	-0.58518	0.043254	-11.5559	1.44E-24	8.62E-23	45.13241	down
REACTOM	-0.58492	0.013436	-8.07876	3.93E-14	2.43E-13	21.45556	down
GOBP_DN/	0.584887	-0.15624	10.26366	1.57E-20	3.49E-19	35.95592	up
GOBP_NEC	-0.58488	-0.02372	-10.9258	1.39E-22	5.06E-21	40.61647	down
GOMF_MH	-0.58452	0.071947	-10.0355	7.82E-20	1.46E-18	34.37365	down
HP_NEOPL	0.584473	0.051568	12.39844	2.89E-27	3.78E-25	51.26461	up
GOMF_DN	0.58445	0.034626	8.808469	3.42E-16	2.98E-15	26.11833	up
TFDP2_TAI	0.584449	-0.1923	9.665084	1.03E-18	1.49E-17	31.8336	up
SANCHEZ_	-0.58435	0.000876	-12.1809	1.45E-26	1.50E-24	49.67266	down
MODULE_!	-0.5842	0.030052	-9.80007	4.04E-19	6.44E-18	32.75482	down
WP_NANC	-0.58381	0.023	-10.526	2.45E-21	6.57E-20	37.79113	down
ROSTY_CE	0.582879	-0.23901	9.309863	1.18E-17	1.35E-16	29.43502	up
GOCC_HAI	-0.58286	0.078474	-10.7245	5.92E-22	1.85E-20	39.18958	down
HP_ABNOI	0.582834	-0.06004	10.49592	3.03E-21	7.97E-20	37.57973	up
WHITFIELD	0.582699	-0.24974	8.941258	1.41E-16	1.32E-15	26.98838	up
GOMF_OX	-0.58263	0.221935	-11.4511	3.09E-24	1.69E-22	44.37686	down
GOBP_ANT	-0.58222	0.027813	-10.7532	4.82E-22	1.53E-20	39.39276	down
BIERIE_INF	-0.58153	0.044489	-8.12811	2.87E-14	1.81E-13	21.76415	down
FAN_EMBF	0.581265	-0.22665	9.477408	3.75E-18	4.74E-17	30.56158	up
GOBP_NEC	-0.58103	0.080541	-9.32312	1.07E-17	1.24E-16	29.52387	down
GOMF_MH	-0.58102	0.039837	-8.91206	1.72E-16	1.57E-15	26.79653	down
MODULE_4	-0.58064	0.050853	-10.4105	5.56E-21	1.38E-19	36.98095	down
HP_IMBAL	-0.58057	0.025637	-9.38843	6.88E-18	8.26E-17	29.96219	down
GOMF_PRI	0.579974	-0.25726	6.817704	8.36E-11	3.24E-10	13.9498	up
GOBP_TEL	0.579968	-0.07174	14.05844	1.15E-32	7.44E-30	63.54154	up
ACEVEDO_	-0.57986	0.118194	-17.0579	1.81E-42	5.84E-38	85.83152	down
CAIRO_HE	-0.57976	0.228823	-13.1197	1.33E-29	3.55E-27	56.57604	down
GOCC_MC	0.579663	-0.19987	9.054862	6.59E-17	6.55E-16	27.73759	up
SARTIPY_B	-0.57957	0.077698	-11.7885	2.61E-25	1.94E-23	46.81552	down
HP_HYPOI	0.579203	-0.05982	11.68463	5.60E-25	3.77E-23	46.06301	up
GOMF_ML	0.579074	-0.11717	8.685966	7.69E-16	6.27E-15	25.32128	up
GOBP_RES	-0.57906	0.071728	-9.69376	8.45E-19	1.25E-17	32.02887	down
GNF2_FEN	0.578359	-0.24371	9.224722	2.10E-17	2.29E-16	28.86592	up
ODONNEL	0.578235	-0.21255	10.18136	2.81E-20	5.79E-19	35.38362	up
GOMF_D_I	0.577669	-0.15688	9.861684	2.63E-19	4.37E-18	33.17698	up
HP_FOCAL	0.577583	0.076774	9.23471	1.96E-17	2.15E-16	28.93256	up
GOBP_DN/	0.577243	-0.20231	9.114996	4.40E-17	4.53E-16	28.13595	up
GOBP_NEC	-0.5771	0.09958	-9.70161	8.00E-19	1.19E-17	32.08235	down
GNF2_RRM	0.57704	-0.22741	9.570441	1.98E-18	2.66E-17	31.19083	up
REACTOM	0.577038	-0.16351	9.716898	7.20E-19	1.09E-17	32.18659	up
GNF2_CAR	-0.57689	0.043192	-9.59376	1.68E-18	2.31E-17	31.34898	down
KALMA_E2	0.576864	-0.26137	8.852399	2.55E-16	2.27E-15	26.40547	up
GNF2_RFC	0.576789	-0.23284	9.229148	2.03E-17	2.23E-16	28.89545	up
GOBP_CHI	0.576367	-0.1321	9.101928	4.80E-17	4.90E-16	28.04928	up
REACTOM	0.576166	-0.28787	8.55331	1.84E-15	1.41E-14	24.46443	up
HP_RECUR	-0.5759	0.019481	-7.94733	9.03E-14	5.28E-13	20.63866	down

HOEK_PBM	-0.57565	0.041048	-12.8227	1.23E-28	2.34E-26	54.38333	down
GOBP_NO	-0.57556	0.114702	-10.2429	1.82E-20	3.96E-19	35.81144	down
DURANTE_	-0.57553	0.00916	-8.24129	1.39E-14	9.18E-14	22.47579	down
GOBP_GLC	0.575468	-0.03453	8.167598	2.23E-14	1.43E-13	22.01186	up
GOBP_REG	-0.57522	0.007256	-8.69155	7.41E-16	6.07E-15	25.35751	down
GNF2_FOS	-0.57518	0.078089	-10.927	1.38E-22	5.03E-21	40.62521	down
MIR4315	0.574603	-0.09812	9.680347	9.27E-19	1.36E-17	31.9375	up
GOBP_FAT	-0.57395	0.12904	-11.826	1.98E-25	1.51E-23	47.08758	down
PASTURAL	-0.57375	-0.02206	-10.3539	8.30E-21	1.97E-19	36.58565	down
GOBP_ATF	-0.57374	0.128348	-11.4869	2.38E-24	1.34E-22	44.63446	down
HP_ABNOI	-0.57361	0.221479	-10.4749	3.52E-21	9.09E-20	37.43211	down
GOBP_FAT	-0.57359	0.270875	-10.3027	1.19E-20	2.72E-19	36.22803	down
GOBP_TRY	-0.57321	0.208416	-10.9709	1.01E-22	3.81E-21	40.93762	down
GOCC_CHI	0.572999	-0.25814	9.258031	1.67E-17	1.86E-16	29.08829	up
GNF2_CD3	-0.57299	0.04705	-9.87679	2.37E-19	3.97E-18	33.28068	down
HP_ABNOI	-0.5723	0.177216	-9.68183	9.17E-19	1.34E-17	31.94763	down
GOBP_NEG	-0.57169	0.008224	-11.261	1.23E-23	5.77E-22	43.01054	down
WP_REGUI	0.571363	-0.17422	9.793786	4.22E-19	6.70E-18	32.7118	up
HP_DECRE	-0.5712	0.215437	-10.6687	8.83E-22	2.65E-20	38.79583	down
GOBP_KIN	0.570918	-0.18941	10.65339	9.85E-22	2.93E-20	38.68773	up
ALONSO_I	-0.57075	0.093942	-9.5969	1.65E-18	2.27E-17	31.37026	down
HP_FOCAL	0.570575	0.000339	12.20521	1.21E-26	1.30E-24	49.85028	up
BOYAULT_	-0.57005	0.290302	-11.1735	2.33E-23	1.03E-21	42.38337	down
HP_PERCU	0.569464	0.065835	12.39354	2.99E-27	3.91E-25	51.22868	up
HP_ENTER	-0.56937	0.071485	-10.1331	3.94E-20	7.82E-19	35.04894	down
GOBP_QUI	-0.56915	0.18951	-9.16768	3.08E-17	3.28E-16	28.48594	down
GNF2_IL2R	-0.5691	0.030663	-9.96026	1.32E-19	2.35E-18	33.85456	down
HP_DISTAI	-0.56906	0.095973	-11.3695	5.60E-24	2.86E-22	43.78949	down
HP_SMALL	0.569035	-0.10355	11.22733	1.57E-23	7.18E-22	42.76899	up
WACKER_I	-0.56899	-0.02378	-12.9818	3.74E-29	8.68E-27	55.55719	down
GOBP_DEN	-0.56834	0.1002	-8.46618	3.25E-15	2.39E-14	23.90527	down
SOBOLEV_	-0.56812	0.064767	-10.5405	2.21E-21	5.98E-20	37.89305	down
GOBP_POS	-0.56786	0.163441	-9.01616	8.55E-17	8.32E-16	27.48189	down
GOBP_NEG	-0.56771	0.151905	-10.213	2.25E-20	4.76E-19	35.60309	down
GOMF_NA	-0.56768	0.126931	-11.8689	1.45E-25	1.14E-23	47.39963	down
LE_SKI_TAI	-0.56761	0.132973	-9.64964	1.15E-18	1.64E-17	31.72852	down
QI_PBMC_	0.567561	-0.22603	9.049256	6.85E-17	6.78E-16	27.70052	up
HP_ABNOI	-0.56755	0.180654	-10.3126	1.11E-20	2.56E-19	36.29721	down
HP_RETIN/	-0.56745	0.225004	-8.21819	1.61E-14	1.05E-13	22.33014	down
GOBP_MRI	0.567257	-0.2009	9.000667	9.49E-17	9.17E-16	27.37963	up
GOBP_TRA	0.56718	-0.18154	9.810409	3.76E-19	6.03E-18	32.82557	up
CROONQU	0.56689	-0.24142	9.456924	4.31E-18	5.38E-17	30.42339	up
MODULE_	0.56687	-0.20341	9.048633	6.88E-17	6.81E-16	27.6964	up
GOBP_NEG	-0.56659	0.125837	-12.0475	3.88E-26	3.58E-24	48.69886	down
LEE_SP4_TI	-0.5665	0.102568	-12.3472	4.22E-27	5.36E-25	50.88889	down
HP_ABNOI	0.565944	-0.10169	10.6149	1.30E-21	3.72E-20	38.41638	up
HP_DECRE	-0.56481	0.174699	-9.49538	3.31E-18	4.24E-17	30.68296	down
GOCC_PLA	-0.5647	0.162873	-13.1136	1.40E-29	3.66E-27	56.53056	down
DESCARTE	-0.56403	0.014626	-8.37928	5.71E-15	4.03E-14	23.35053	down
SOTIRIOU_	0.564022	-0.23873	9.464422	4.09E-18	5.13E-17	30.47396	up
GNF2_CKS	0.563637	-0.25804	9.274863	1.49E-17	1.68E-16	29.20079	up
GNF2_TNF	-0.56339	-0.0179	-9.07346	5.82E-17	5.82E-16	27.86064	down
GOBP_REG	-0.56319	-0.0261	-7.95007	8.88E-14	5.19E-13	20.65562	down
GOBP_STR	-0.56262	0.134241	-10.7415	5.24E-22	1.65E-20	39.31001	down
REACTOM	-0.56246	0.106965	-9.64896	1.15E-18	1.64E-17	31.72394	down
IIZUKA_LIV	0.562108	-0.11572	11.06125	5.25E-23	2.15E-21	41.58143	up
CROONQU	0.562058	-0.22253	9.484799	3.56E-18	4.53E-17	30.61148	up
HP_JEJUN/	0.561515	-0.1177	9.989344	1.08E-19	1.96E-18	34.05497	up

GOMF_C_/	-0.5614	0.24131	-9.47452	3.82E-18	4.82E-17	30.54212	down
GOBP_NE(	-0.56117	0.099138	-11.9551	7.67E-26	6.51E-24	48.02613	down
GOBP_REG	0.561074	-0.18479	10.3966	6.13E-21	1.50E-19	36.88385	up
MARTINEL	-0.56099	0.054747	-9.35537	8.62E-18	1.01E-16	29.74014	down
HP_DECRE	-0.56056	0.155698	-10.8495	2.41E-22	8.27E-21	40.07499	down
YAO_TEMI	-0.56043	0.164355	-14.8863	2.24E-35	2.68E-32	69.70724	down
KAMMING	0.560078	-0.21429	9.3759	7.50E-18	8.91E-17	29.87801	up
HP_RETICU	0.559898	-0.04282	10.13996	3.76E-20	7.49E-19	35.09637	up
SMID_BRE/	-0.55984	0.013207	-9.51323	2.93E-18	3.79E-17	30.80357	down
GOBP_MEI	0.559729	-0.15013	8.492136	2.74E-15	2.04E-14	24.07154	up
GOMF_AC	-0.55964	0.283055	-9.48215	3.63E-18	4.60E-17	30.59361	down
GNF2_MSI	0.559545	-0.22433	9.061655	6.30E-17	6.27E-16	27.78253	up
MCGOWA	0.559504	-0.04871	8.708882	6.61E-16	5.46E-15	25.46996	up
HP_CONCI	-0.55938	0.112465	-8.99388	9.93E-17	9.55E-16	27.33489	down
KOBAYASI	-0.55929	-0.02877	-10.5962	1.48E-21	4.20E-20	38.28436	down
GOBP_NAT	-0.55873	0.046618	-8.63323	1.09E-15	8.65E-15	24.97988	down
GOCC_ENI	0.558695	-0.11162	9.590052	1.73E-18	2.36E-17	31.32381	up
BOYAULT_	0.558537	-0.17138	11.2634	1.21E-23	5.68E-22	43.02757	up
REACTOM	-0.55838	0.176133	-10.4262	4.97E-21	1.25E-19	37.09096	down
GOMF_AR/	-0.5581	0.230641	-11.7699	2.99E-25	2.16E-23	46.68114	down
GOBP_THI	-0.55803	0.11614	-7.98219	7.25E-14	4.30E-13	20.85461	down
GOCC_GPI	0.55778	-0.15222	9.557562	2.16E-18	2.88E-17	31.10357	up
HP_ANOTI	0.55765	-0.21878	10.52563	2.45E-21	6.58E-20	37.78836	up
GOBP_COI	-0.55704	0.153478	-12.2595	8.08E-27	9.25E-25	50.24732	down
GOBP_NE(	-0.55684	0.005998	-9.52666	2.67E-18	3.49E-17	30.89436	down
GNF2_CD7	-0.55649	0.051782	-8.9949	9.86E-17	9.50E-16	27.34162	down
GNF2_CYP	-0.55594	0.232994	-11.5532	1.47E-24	8.74E-23	45.11291	down
GOBP_ALK	-0.55572	0.221364	-10.2354	1.92E-20	4.15E-19	35.7595	down
GNF2_S10(	-0.55554	-0.01626	-8.60862	1.28E-15	1.01E-14	24.82091	down
WIEMANN	-0.55542	0.094187	-11.4819	2.47E-24	1.38E-22	44.59822	down
REACTOM	0.555187	-0.17308	9.173812	2.96E-17	3.15E-16	28.52674	up
KONG_E2F	0.555153	-0.2224	9.38987	6.82E-18	8.20E-17	29.9719	up
GOMF_HYI	-0.5551	0.101539	-10.7577	4.67E-22	1.49E-20	39.42472	down
MODULE_2	-0.55475	0.097209	-11.2705	1.15E-23	5.46E-22	43.0784	down
WP_EICOS	-0.55453	0.258216	-10.2966	1.25E-20	2.83E-19	36.1853	down
PLASARI_T	-0.55436	0.072612	-10.0181	8.84E-20	1.63E-18	34.25314	down
WP_METH	-0.55423	0.193033	-12.1264	2.16E-26	2.14E-24	49.27451	down
REACTOM	-0.55412	0.281621	-10.9944	8.50E-23	3.27E-21	41.1048	down
GOBP_LUI	-0.55403	0.080802	-8.57123	1.63E-15	1.26E-14	24.57978	down
HP_THOR/	0.553939	0.019721	11.63965	7.78E-25	5.07E-23	45.73755	up
GOBP_NE(	-0.55303	0.079715	-9.33005	1.02E-17	1.19E-16	29.57027	down
HP_LIMBA	-0.55283	0.178044	-10.8962	1.73E-22	6.09E-21	40.40616	down
GOMF_URI	-0.55265	0.132422	-10.6406	1.08E-21	3.16E-20	38.59741	down
HP_ABNOI	-0.55246	0.019144	-11.6122	9.52E-25	6.04E-23	45.53919	down
GOMF_PEI	-0.55196	0.160832	-10.5565	1.97E-21	5.42E-20	38.00505	down
GOCC_MIT	0.551399	-0.16218	11.62493	8.67E-25	5.57E-23	45.6311	up
GOBP_NE(	-0.55101	-0.03446	-10.2474	1.76E-20	3.85E-19	35.84269	down
GOBP_NEU	-0.55101	0.121627	-12.9688	4.12E-29	9.36E-27	55.46091	down
GOMF_HIS	0.550897	-0.20621	8.689888	7.49E-16	6.13E-15	25.34671	up
REACTOM	0.550867	-0.22222	8.763302	4.61E-16	3.93E-15	25.82382	up
CHANG_P(	-0.55063	0.063319	-11.5402	1.61E-24	9.45E-23	45.01935	down
HP_CORTI(	0.55049	-0.09599	9.493997	3.34E-18	4.28E-17	30.6736	up
GOBP_POS	-0.55037	0.033051	-8.43752	3.91E-15	2.84E-14	23.72201	down
GOBP_PAF	-0.55013	0.072015	-12.951	4.71E-29	1.04E-26	55.3298	down
GOBP_HIS	-0.55008	0.190019	-9.81442	3.66E-19	5.89E-18	32.85302	down
REACTOM	-0.55008	0.190019	-9.81442	3.66E-19	5.89E-18	32.85302	down
GOBP_MO	-0.54989	0.026825	-8.63106	1.10E-15	8.77E-15	24.96584	down
GOBP_MU	0.549842	-0.17094	11.14222	2.92E-23	1.27E-21	42.15977	up

REACTOM	-0.54983	0.179	-11.4252	3.73E-24	2.00E-22	44.19027	down
GOMF_GL'	-0.54962	0.12684	-9.96422	1.29E-19	2.29E-18	33.88183	down
GOBP_POS	-0.54943	0.068197	-8.74305	5.27E-16	4.44E-15	25.69198	down
GOBP_REG	-0.54941	0.11026	-10.7815	3.94E-22	1.28E-20	39.59282	down
GOMF_CD	-0.5494	0.0781	-8.87712	2.17E-16	1.95E-15	26.56735	down
HOEK_MO	-0.54933	0.160514	-10.1313	4.00E-20	7.91E-19	35.03616	down
GOBP_REG	0.548627	-0.08128	11.05484	5.49E-23	2.23E-21	41.53575	up
PATIL_LIVE	0.548419	-0.14168	14.79694	4.40E-35	4.74E-32	69.04136	up
GOMF_CR(	0.548412	-0.13253	10.41888	5.24E-21	1.31E-19	37.03972	up
HP_HEMO	-0.54837	0.021683	-9.34828	9.05E-18	1.06E-16	29.69259	down
GOMF_GL(	-0.54835	-0.0289	-9.41012	5.94E-18	7.20E-17	30.10807	down
GOCC_TOI	0.548168	-0.01754	9.899522	2.02E-19	3.43E-18	33.43675	up
GOMF_EST	-0.54761	0.291151	-9.07767	5.66E-17	5.68E-16	27.88857	down
GNF2_RFC	0.547569	-0.21655	9.197759	2.52E-17	2.71E-16	28.68618	up
WP_SYNTH	-0.54751	0.261058	-10.4393	4.53E-21	1.14E-19	37.18272	down
GNF2_TNF	-0.54723	-0.01079	-8.26539	1.19E-14	7.96E-14	22.62803	down
GOMF_SIA	-0.54723	0.044025	-10.5926	1.52E-21	4.31E-20	38.25928	down
HP_CERVI(	-0.54713	-0.01278	-8.62763	1.13E-15	8.95E-15	24.94364	down
GOBP_REG	0.546973	-0.20602	8.826059	3.04E-16	2.67E-15	26.23322	up
GOMF_MIS	0.546845	-0.06887	10.21796	2.17E-20	4.61E-19	35.63789	up
HP_ELEVA	-0.54678	0.233133	-8.85503	2.51E-16	2.24E-15	26.42271	down
UZONYI_R	-0.54668	0.121543	-10.7499	4.94E-22	1.56E-20	39.3691	down
GOBP_SHC	-0.54663	0.303878	-9.95679	1.36E-19	2.39E-18	33.83064	down
BIOCARTA	-0.54616	0.091673	-7.5865	8.54E-13	4.35E-12	18.43526	down
GOMF_HA	-0.54606	0.054291	-9.43251	5.09E-18	6.26E-17	30.25881	down
REACTOM	-0.54574	0.090252	-12.929	5.55E-29	1.19E-26	55.16731	down
GOBP_DN/	0.545641	-0.18407	10.17858	2.86E-20	5.90E-19	35.36434	up
GOBP_NEC	-0.5456	0.042986	-9.51762	2.84E-18	3.69E-17	30.83326	down
WP_KETO(	-0.54557	0.177855	-11.2161	1.71E-23	7.75E-22	42.68877	down
GOMF_IMI	-0.54537	0.04245	-10.5178	2.59E-21	6.92E-20	37.73328	down
GNF2_HCK	-0.54508	0.013494	-9.36397	8.13E-18	9.61E-17	29.79785	down
GOBP_COI	-0.54497	0.089558	-8.39105	5.29E-15	3.76E-14	23.42551	down
GOBP_NEC	-0.54487	0.135523	-9.89529	2.08E-19	3.51E-18	33.40768	down
BOYAULT_	0.544679	-0.21584	10.99542	8.44E-23	3.25E-21	41.11217	up
GOCC_MI(	0.544656	-0.03348	10.33354	9.59E-21	2.25E-19	36.44317	up
GOMF_MU	0.543776	-0.01603	10.43031	4.83E-21	1.22E-19	37.11974	up
REACTOM	-0.54371	0.239579	-9.01241	8.77E-17	8.51E-16	27.45711	down
HP_BRAN(	0.543297	-0.16082	9.595552	1.66E-18	2.28E-17	31.36112	up
GOMF_DN	0.543083	-0.12786	9.392374	6.70E-18	8.07E-17	29.98873	up
GOBP_POS	-0.54306	0.120347	-10.2429	1.82E-20	3.96E-19	35.81113	down
GOMF_MIS	0.542959	-0.05487	11.0723	4.84E-23	2.00E-21	41.66034	up
GOBP_POS	0.542932	-0.20502	9.7954	4.18E-19	6.63E-18	32.72285	up
GOBP_SU(	-0.54214	0.303196	-8.65282	9.56E-16	7.69E-15	25.10654	down
GOMF_AN	0.542032	-0.16214	10.15956	3.27E-20	6.63E-19	35.23232	up
HP_ABSEN	0.541215	-0.09966	10.95564	1.12E-22	4.20E-21	40.82901	up
GOMF_ATI	0.540815	-0.15997	10.50089	2.92E-21	7.72E-20	37.61461	up
GOBP_PRC	0.540701	-0.20914	10.31338	1.11E-20	2.55E-19	36.30249	up
GOMF_RA(	-0.5406	-0.09198	-10.1646	3.16E-20	6.44E-19	35.26698	down
GOBP_NEC	-0.5404	0.123977	-9.03254	7.66E-17	7.51E-16	27.59002	down
GOMF_CEI	0.540351	-0.1106	9.891702	2.14E-19	3.60E-18	33.38304	up
GOBP_LIPI	-0.54021	0.262178	-10.2369	1.90E-20	4.11E-19	35.76966	down
LOPEZ_ME	0.540015	-0.24919	9.655852	1.10E-18	1.58E-17	31.77079	up
GOBP_POS	-0.53997	0.066535	-6.63624	2.36E-10	8.65E-10	12.93619	down
GOBP_REG	-0.53987	0.121234	-9.13918	3.74E-17	3.91E-16	28.2965	down
KAZMIN_P	-0.53973	0.005853	-8.66643	8.74E-16	7.07E-15	25.1947	down
WP_EV_RE	-0.53923	0.04237	-9.91988	1.76E-19	3.02E-18	33.57667	down
GOBP_LAB	-0.53922	0.001805	-13.8543	5.36E-32	2.93E-29	62.02355	down
REACTOM	0.53885	-0.19821	8.760035	4.71E-16	4.01E-15	25.80255	up

GOBP_ENE	-0.53879	0.203803	-7.75223	3.06E-13	1.66E-12	19.43994	down
FUJIWARA_	-0.53852	0.203187	-10.4123	5.49E-21	1.36E-19	36.99373	down
SCHUHMA	-0.5385	-0.01115	-9.49989	3.21E-18	4.12E-17	30.71338	down
HP_ABNOI	-0.53829	0.043981	-10.7036	6.88E-22	2.12E-20	39.04185	down
REACTOM	-0.53825	0.304941	-8.31293	8.76E-15	6.00E-14	22.92901	down
GOBP_REG	0.5382	-0.15244	9.659473	1.07E-18	1.54E-17	31.79542	up
GOBP_KIN	0.538081	-0.18239	10.7752	4.12E-22	1.33E-20	39.54841	up
GERHOLD_	-0.5379	0.028646	-11.75	3.46E-25	2.47E-23	46.53674	down
BLANCO_N	0.537887	-0.21135	9.500925	3.19E-18	4.10E-17	30.7204	up
GNF2_PEC	-0.53754	0.031617	-9.21238	2.28E-17	2.48E-16	28.78362	down
LI_PBMC_N	-0.53739	0.041402	-10.3249	1.02E-20	2.37E-19	36.3827	down
GOBP_MH	-0.53736	0.03918	-12.4707	1.69E-27	2.36E-25	51.79421	down
HP_SEVERI	0.536915	-0.10715	9.676392	9.52E-19	1.39E-17	31.91058	up
GOBP_POS	-0.5368	0.106318	-10.934	1.31E-22	4.82E-21	40.67506	down
GOBP_ISO	-0.53626	0.203506	-9.20549	2.39E-17	2.58E-16	28.73769	down
HP_UPPER_	-0.53617	0.028474	-9.3796	7.31E-18	8.71E-17	29.9029	down
GOBP_GLY	-0.53605	0.23113	-9.47721	3.75E-18	4.74E-17	30.56025	down
GOMF_INT	-0.5359	-0.01618	-9.6728	9.76E-19	1.42E-17	31.8861	down
WP_ARYLA	-0.53585	0.280307	-9.40506	6.14E-18	7.44E-17	30.07404	down
GOBP_OM	-0.53577	0.285723	-10.6915	7.50E-22	2.29E-20	38.95641	down
REACTOM	-0.53577	0.285723	-10.6915	7.50E-22	2.29E-20	38.95641	down
DESCARTE	-0.53557	0.006678	-8.69792	7.10E-16	5.84E-15	25.39885	down
GNF2_CEB	-0.53553	0.2077	-11.2799	1.08E-23	5.14E-22	43.14554	down
YU_MYC_T	0.535531	-0.21061	9.74675	5.85E-19	9.04E-18	32.39031	up
GOBP_HYF	-0.5354	-0.01741	-9.63762	1.24E-18	1.77E-17	31.64682	down
GOBP_CD4	-0.53527	0.105448	-12.2291	1.01E-26	1.12E-24	50.02489	down
GOBP_OXY	-0.535	0.063545	-11.6616	6.63E-25	4.37E-23	45.89621	down
GOCC_LOV	-0.53496	0.061979	-11.3019	9.16E-24	4.46E-22	43.30411	down
GOBP_REG	-0.53472	0.165197	-11.5459	1.55E-24	9.14E-23	45.06007	down
CAR_IGFBF	-0.53467	0.275592	-11.7168	4.42E-25	3.07E-23	46.29585	down
GOBP_REG	-0.53454	0.224806	-11.7178	4.39E-25	3.06E-23	46.30329	down
HP_HYPER	-0.53447	0.008691	-11.8853	1.28E-25	1.03E-23	47.51885	down
GOCC_CYT	0.534403	-0.09795	8.454666	3.50E-15	2.56E-14	23.8316	up
GOMF_TEL	0.534375	-0.08967	9.978884	1.16E-19	2.09E-18	33.98287	up
GOMF_RN	0.534375	-0.08967	9.978884	1.16E-19	2.09E-18	33.98287	up
HP_LEBER_	-0.53391	0.196557	-7.658	5.50E-13	2.88E-12	18.86717	down
NAKAYA_F	-0.53369	0.091683	-9.86793	2.52E-19	4.20E-18	33.21983	down
REACTOM	-0.53367	0.26241	-7.99838	6.54E-14	3.91E-13	20.95507	down
GOBP_CHC	0.533523	-0.07199	9.181505	2.81E-17	3.00E-16	28.57794	up
GOMF_BET	0.533397	-0.0576	7.078391	1.82E-11	7.73E-11	15.43703	up
WP_DEVEL	-0.53336	0.028651	-9.0369	7.44E-17	7.32E-16	27.61887	down
BURTON_A	-0.53292	0.06818	-10.4431	4.41E-21	1.11E-19	37.20968	down
WU_APOP	0.532816	-0.21062	9.532874	2.56E-18	3.36E-17	30.93643	up
REACTOM	0.532804	-0.211	9.885026	2.24E-19	3.76E-18	33.33719	up
REACTOM	-0.53273	0.275919	-7.69517	4.37E-13	2.32E-12	19.09261	down
DESCARTE	-0.53239	0.018631	-8.28814	1.03E-14	6.94E-14	22.77194	down
SCHAEFFEI	-0.53227	0.092175	-8.88382	2.07E-16	1.87E-15	26.61129	down
GOMF_NU	-0.53196	0.139354	-8.76531	4.55E-16	3.89E-15	25.83688	down
WANG_ES(	-0.53183	-0.09189	-8.06717	4.23E-14	2.60E-13	21.38318	down
GOBP_DNA	0.531818	-0.18412	9.579434	1.86E-18	2.52E-17	31.2518	up
GOBP_POS	0.531591	-0.12399	8.782841	4.05E-16	3.49E-15	25.95114	up
CHEN_HO)	-0.53099	0.081325	-10.2253	2.06E-20	4.42E-19	35.68889	down
HP_EYELID	0.530981	-0.08921	9.696681	8.28E-19	1.23E-17	32.04877	up
REACTOM	-0.53091	0.157436	-11.822	2.04E-25	1.55E-23	47.0588	down
GOBP_MIT	0.530867	-0.07339	13.00838	3.06E-29	7.35E-27	55.75336	up
GOCC_HIG	-0.53051	0.143346	-11.8899	1.24E-25	9.99E-24	47.55228	down
SIMBULAN	0.529903	-0.25348	9.597227	1.64E-18	2.26E-17	31.37249	up
GOBP_NEC	-0.5298	0.067494	-8.57844	1.56E-15	1.21E-14	24.62621	down

GOBP_TRY	-0.52962	0.203903	-11.3473	6.58E-24	3.31E-22	43.6297	down
GOMF_DN	0.529241	-0.18172	11.28447	1.04E-23	4.99E-22	43.17871	up
HP_RECUR	-0.52911	0.110105	-10.1109	4.61E-20	9.05E-19	34.89476	down
GOBP_RES	0.529032	-0.07746	12.21008	1.17E-26	1.26E-24	49.88585	up
AMIT_EGF_	-0.52881	0.090897	-11.9458	8.21E-26	6.88E-24	47.95828	down
BIOCARTA	-0.52876	0.268674	-9.99425	1.04E-19	1.90E-18	34.08877	down
HP_CENTR	-0.52864	0.214577	-7.8735	1.44E-13	8.14E-13	20.18306	down
REACTOM	0.528608	-0.12448	9.561537	2.10E-18	2.81E-17	31.1305	up
HP_INCRE	-0.52838	0.083823	-11.3794	5.21E-24	2.68E-22	43.86026	down
GOCC_CLA	0.528142	-0.09487	11.61213	9.52E-25	6.04E-23	45.5386	up
HP_FLAT_(	0.52806	-0.13803	13.98818	1.96E-32	1.22E-29	63.01889	up
BIOCARTA	-0.52796	0.108422	-13.285	3.86E-30	1.14E-27	57.79939	down
REN_BOUI	0.527788	-0.21487	9.149634	3.48E-17	3.67E-16	28.36596	up
REACTOM	-0.52777	0.12071	-13.0788	1.81E-29	4.56E-27	56.27341	down
GOMF_HEI	-0.52772	0.088004	-9.68894	8.73E-19	1.29E-17	31.99605	down
BIOCARTA	-0.52748	0.142511	-10.423	5.09E-21	1.28E-19	37.06844	down
GOBP_MIC	0.527378	-0.0797	8.491477	2.75E-15	2.05E-14	24.06731	up
AMIT_EGF_	-0.52732	0.071384	-12.0608	3.51E-26	3.30E-24	48.79641	down
GOBP_POS	-0.52719	0.095789	-10.3006	1.21E-20	2.76E-19	36.21333	down
HP_HYPOT	-0.52711	0.131842	-14.4507	6.00E-34	5.09E-31	66.46204	down
REACTOM	0.52702	-0.0696	9.638969	1.23E-18	1.75E-17	31.65598	up
GOBP_POS	-0.52702	0.043965	-8.16098	2.33E-14	1.49E-13	21.97032	down
DURANTE_	-0.52673	-0.03696	-8.05232	4.65E-14	2.84E-13	21.29062	down
GOBP_REG	-0.52658	0.096484	-9.2932	1.32E-17	1.50E-16	29.32344	down
chr8p22	-0.52629	0.124084	-9.33015	1.02E-17	1.19E-16	29.57095	down
REACTOM	0.525689	-0.23612	7.882026	1.36E-13	7.74E-13	20.23555	up
GOBP_CHI	0.525551	-0.10286	9.414794	5.75E-18	6.99E-17	30.13956	up
HP_CHON	-0.52555	0.09102	-11.254	1.30E-23	6.03E-22	42.96048	down
DESCARTE	-0.52544	0.021094	-8.13601	2.73E-14	1.73E-13	21.81368	down
chr8p12	-0.52498	0.017904	-9.13871	3.75E-17	3.92E-16	28.29337	down
GNF2_SMC	0.524853	-0.23099	8.888233	2.01E-16	1.82E-15	26.6402	up
BIOCARTA	0.524715	-0.21793	8.689383	7.52E-16	6.15E-15	25.34344	up
GOBP_REG	-0.52468	0.129583	-8.75934	4.73E-16	4.02E-15	25.798	down
GOBP_EPC	-0.52452	0.244798	-11.7789	2.80E-25	2.05E-23	46.74645	down
ZAK_PBMC	-0.52435	0.012662	-10.4911	3.14E-21	8.24E-20	37.5456	down
GOBP_HUI	-0.52417	0.1029	-11.3118	8.52E-24	4.18E-22	43.37481	down
GNF2_ITGE	-0.5241	-0.01454	-8.28436	1.05E-14	7.10E-14	22.74803	down
GOBP_NEC	-0.52397	0.010946	-8.85504	2.51E-16	2.24E-15	26.42275	down
GOBP_NEC	-0.52366	0.177223	-10.635	1.12E-21	3.28E-20	38.55802	down
GOBP_SPII	0.523624	-0.15839	10.12336	4.22E-20	8.34E-19	34.98129	up
GOMF_DN	0.523404	-0.19317	9.138329	3.76E-17	3.93E-16	28.29084	up
BIOCARTA	-0.52336	0.01941	-6.73841	1.32E-10	5.00E-10	13.50465	down
DURANTE_	-0.52299	-0.01009	-8.32014	8.37E-15	5.75E-14	22.97473	down
HP_SMALL	0.522638	-0.09968	9.175529	2.92E-17	3.12E-16	28.53817	up
GOBP_REG	-0.52256	0.031753	-7.89158	1.28E-13	7.32E-13	20.29442	down
MODULE_(	-0.52254	0.077587	-11.4595	2.91E-24	1.59E-22	44.43725	down
HP_MULTI	-0.52249	0.156574	-8.28484	1.05E-14	7.08E-14	22.75107	down
GOMF_NEI	0.522439	-0.02234	9.52434	2.71E-18	3.54E-17	30.8787	up
GOCC_FEM	0.522263	-0.04153	8.875164	2.19E-16	1.98E-15	26.55455	up
GNF2_CD9	-0.52217	0.024731	-8.31297	8.76E-15	6.00E-14	22.92925	down
GOMF_HEI	-0.52183	0.048537	-8.15966	2.35E-14	1.50E-13	21.96201	down
LIANG_SILI	0.52182	-0.23696	8.651804	9.63E-16	7.73E-15	25.09999	up
WP_SARSC	-0.52179	0.119565	-12.2036	1.22E-26	1.31E-24	49.83842	down
GOBP_COI	0.521175	-0.10646	9.510774	2.98E-18	3.85E-17	30.78697	up
MAINA_HY	-0.52112	-0.10481	-8.68344	7.82E-16	6.37E-15	25.30489	down
REACTOM	0.521012	-0.21321	8.91297	1.71E-16	1.56E-15	26.80251	up
GOBP_PRC	0.520959	-0.06351	9.31659	1.12E-17	1.29E-16	29.48008	up
GOMF_ENI	0.520955	-0.09948	10.71357	6.41E-22	1.99E-20	39.11254	up

GOBP_IND	-0.5207	0.115812	-8.89661	1.90E-16	1.73E-15	26.69517	down
ZHONG_PI	0.520608	-0.20682	9.479854	3.68E-18	4.67E-17	30.5781	up
FAN_EMBF	-0.5201	0.064756	-12.7293	2.46E-28	4.32E-26	53.69506	down
HP_ABNOI	-0.52009	0.008576	-9.96562	1.28E-19	2.27E-18	33.89147	down
GOBP_GLY	-0.51986	0.226251	-9.7131	7.39E-19	1.11E-17	32.16069	down
SASSON_F	-0.51981	0.106008	-12.1888	1.36E-26	1.42E-24	49.73025	down
WP_SULIN	-0.51978	0.149528	-9.73503	6.35E-19	9.71E-18	32.31031	down
HP_CONFE	0.519664	-0.07667	9.097332	4.95E-17	5.04E-16	28.01881	up
GOMF_MA	-0.5196	0.090228	-13.5955	3.76E-31	1.48E-28	60.10114	down
GOCC_PR(	0.519537	-0.22708	9.584633	1.79E-18	2.44E-17	31.28705	up
PUJANA_B	0.519503	-0.21963	9.599085	1.62E-18	2.24E-17	31.38509	up
HP_ETHYLI	-0.51927	0.220878	-9.07631	5.71E-17	5.73E-16	27.87956	down
GROSS_HII	-0.51926	0.165429	-8.8521	2.56E-16	2.28E-15	26.40349	down
HP_IMPAIF	-0.51924	-0.0653	-6.49231	5.31E-10	1.86E-09	12.14533	down
HP_MICRC	0.51919	-0.2365	9.689988	8.67E-19	1.28E-17	32.00317	up
MODULE_:	-0.51898	0.219134	-9.62649	1.34E-18	1.89E-17	31.57116	down
GOMF_AP_	-0.51894	0.114741	-10.7327	5.58E-22	1.75E-20	39.24783	down
GOBP_SPE	-0.51879	0.057398	-8.97722	1.11E-16	1.06E-15	27.22505	down
FRASOR_R	0.518668	-0.21151	9.796803	4.14E-19	6.58E-18	32.73245	up
GOBP_NE(	-0.51849	0.035181	-8.06334	4.33E-14	2.66E-13	21.35929	down
REACTOM	0.518284	-0.12471	10.57992	1.67E-21	4.68E-20	38.17007	up
chr1q22	0.518161	-0.15506	10.05338	6.90E-20	1.31E-18	34.49704	up
REACTOM	-0.51789	0.134499	-13.7248	1.42E-31	6.46E-29	61.06137	down
HP_ABNOI	0.51787	-0.00391	8.680828	7.95E-16	6.47E-15	25.28797	up
MODULE_!	-0.51783	0.226802	-9.56125	2.11E-18	2.82E-17	31.12856	down
REACTOM	-0.51779	-0.05454	-8.74115	5.34E-16	4.49E-15	25.67966	down
chr13q22	-0.51764	0.167591	-10.0573	6.71E-20	1.28E-18	34.5243	down
WP_PLATE	-0.51753	0.059548	-8.32529	8.09E-15	5.57E-14	23.00738	down
GOBP_POS	-0.51725	0.103249	-9.98407	1.12E-19	2.03E-18	34.01863	down
GOBP_MIT	-0.51717	0.103086	-7.52198	1.27E-12	6.32E-12	18.04759	down
GOBP_GLI/	-0.5171	0.050767	-11.1359	3.06E-23	1.33E-21	42.11428	down
ACEVEDO_	-0.51706	0.13148	-15.3246	8.22E-37	1.66E-33	72.9725	down
DESCARTE	-0.51705	0.051979	-11.2135	1.74E-23	7.89E-22	42.66986	down
MODULE_:	0.516609	-0.24735	8.60689	1.29E-15	1.02E-14	24.80972	up
REACTOM	-0.51653	0.027103	-7.6319	6.46E-13	3.35E-12	18.70924	down
AIZARANI_	-0.51646	0.22698	-11.5366	1.66E-24	9.66E-23	44.99284	down
GOBP_DN/	0.516435	-0.19297	10.64809	1.02E-21	3.02E-20	38.65032	up
GOBP_CEN	0.516305	-0.12742	9.922227	1.73E-19	2.97E-18	33.59282	up
GOBP_REG	-0.51597	0.026536	-9.60602	1.55E-18	2.15E-17	31.43219	down
GOBP_LEU	-0.51593	0.094309	-11.0046	7.90E-23	3.06E-21	41.17763	down
GOBP_REG	0.515849	0.033724	8.380266	5.67E-15	4.01E-14	23.3568	up
GOBP_NE(	-0.51581	0.161702	-9.68487	8.98E-19	1.32E-17	31.96828	down
GOBP_DN/	0.515803	-0.12593	7.032614	2.39E-11	9.98E-11	15.17328	up
GOBP_TOL	-0.51571	0.060257	-11.0949	4.11E-23	1.72E-21	41.82156	down
GOBP_POS	-0.51568	0.067139	-10.1247	4.18E-20	8.27E-19	34.99076	down
GOBP_ACU	-0.51548	0.131846	-12.8725	8.46E-29	1.69E-26	54.75033	down
REACTOM	-0.51533	0.160551	-10.8714	2.06E-22	7.16E-21	40.23	down
GREENBAU	0.515059	-0.19763	10.08154	5.67E-20	1.09E-18	34.69172	up
SMID_BRE/	-0.51503	0.032355	-10.6379	1.10E-21	3.22E-20	38.57865	down
REACTOM	-0.51487	0.234324	-8.23715	1.43E-14	9.41E-14	22.44967	down
GOBP_POS	-0.51475	0.213225	-8.76021	4.71E-16	4.01E-15	25.80367	down
OHASHI_A	0.51463	-0.24441	9.158974	3.27E-17	3.46E-16	28.42804	up
MONTERC	-0.51446	0.148093	-10.7755	4.11E-22	1.33E-20	39.5502	down
GOBP_ATT	0.514366	-0.13239	9.562694	2.09E-18	2.80E-17	31.13834	up
MODULE_`	-0.51432	0.218362	-10.9698	1.02E-22	3.84E-21	40.92944	down
GOBP_REG	-0.51425	-0.01758	-7.22735	7.53E-12	3.36E-11	16.30271	down
GOBP_TEL(	0.514242	-0.07662	12.27008	7.48E-27	8.64E-25	50.32463	up
WP_KYNUI	-0.51423	0.140555	-15.1617	2.81E-36	4.32E-33	71.75929	down

HP_EPISCL	-0.51406	0.110749	-9.51928	2.81E-18	3.65E-17	30.84451	down
GOBP_NEU	-0.51394	0.083447	-13.3862	1.81E-30	5.78E-28	58.54861	down
GOBP_POS	0.513757	-0.23573	9.549182	2.29E-18	3.04E-17	31.04682	up
WHITEFOR	0.513324	-0.21822	9.526847	2.67E-18	3.49E-17	30.89566	up
CAR_HPX	-0.51328	0.261367	-11.1136	3.59E-23	1.53E-21	41.95519	down
GOBP_SOM	0.513181	-0.06664	10.19523	2.55E-20	5.30E-19	35.47995	up
GOCC_T_C	-0.51315	-0.01886	-7.61306	7.25E-13	3.74E-12	18.59543	down
GOBP_REG	-0.51265	0.079091	-10.189	2.66E-20	5.52E-19	35.43694	down
GOMF_CYC	-0.51259	0.038904	-10.1071	4.74E-20	9.27E-19	34.86858	down
BIOCARTA	-0.51207	0.098353	-10.4204	5.18E-21	1.30E-19	37.05036	down
GOBP_ACT	0.51196	-0.1869	9.55655	2.18E-18	2.90E-17	31.09672	up
HP_ABNOI	0.511658	-0.11816	12.01997	4.75E-26	4.27E-24	48.49855	up
GOBP_TRY	-0.5115	0.156239	-9.1724	2.99E-17	3.18E-16	28.51732	down
REACTOM	-0.51147	0.002602	-7.79061	2.41E-13	1.33E-12	19.67445	down
GOBP_PEP	-0.5111	0.026537	-8.17815	2.08E-14	1.34E-13	22.07813	down
GOMF_AM	-0.51099	0.229763	-8.80323	3.54E-16	3.08E-15	26.08415	down
MODULE_	-0.51097	0.06606	-9.61015	1.50E-18	2.09E-17	31.46017	down
GOMF_GPI	0.510961	-0.05281	10.8769	1.98E-22	6.92E-21	40.26938	up
ZHONG_PI	0.510932	-0.22919	8.970365	1.16E-16	1.10E-15	27.17991	up
GOCC_MA	-0.51073	0.061917	-7.00727	2.77E-11	1.15E-10	15.02775	down
GOMF_H3_	0.510708	-0.07753	9.350931	8.89E-18	1.04E-16	29.71035	up
GOMF_TYI	-0.51052	0.05123	-8.81525	3.27E-16	2.86E-15	26.1626	down
GNF2_CAS	-0.51051	0.017998	-9.14501	3.59E-17	3.77E-16	28.33523	down
SOBOLEV_	-0.51033	0.152657	-8.71471	6.36E-16	5.27E-15	25.50783	down
MODULE_!	0.509966	-0.26093	7.954005	8.66E-14	5.07E-13	20.67997	up
REACTOM	-0.50972	0.24033	-9.3824	7.17E-18	8.57E-17	29.92166	down
GOBP_NEC	-0.5097	0.084849	-10.8603	2.23E-22	7.68E-21	40.15168	down
ZHOU_CEL	0.509695	-0.24194	8.75773	4.78E-16	4.06E-15	25.78754	up
MODULE_4	-0.50955	0.045959	-9.65252	1.12E-18	1.61E-17	31.74814	down
REACTOM	0.509442	-0.20851	8.60884	1.28E-15	1.01E-14	24.82231	up
GOBP_REG	-0.50944	0.17609	-8.63424	1.08E-15	8.61E-15	24.98638	down
MIR6746_5	0.509361	0.008783	8.835817	2.85E-16	2.52E-15	26.29701	up
MYLLYKAN	0.509208	-0.02688	9.926575	1.68E-19	2.90E-18	33.62273	up
GOBP_CHI	0.509204	-0.22032	9.403364	6.22E-18	7.53E-17	30.06265	up
DAZARD_U	-0.50916	0.026046	-9.8184	3.56E-19	5.75E-18	32.88029	down
GOCC_KIN	0.509015	-0.09825	8.799946	3.62E-16	3.14E-15	26.0627	up
GOBP_NEC	-0.50898	0.051425	-6.31621	1.41E-09	4.65E-09	11.19384	down
BIOCARTA	-0.50895	0.120729	-10.5586	1.94E-21	5.35E-20	38.02035	down
GOBP_REG	-0.50886	0.106533	-10.943	1.23E-22	4.55E-21	40.73934	down
GOMF_OX	-0.50865	0.064025	-10.4498	4.20E-21	1.07E-19	37.25653	down
GOMF_AR/	-0.50863	0.2507	-10.9849	9.11E-23	3.47E-21	41.03711	down
GOBP_POS	-0.50827	0.106792	-9.714	7.34E-19	1.11E-17	32.16682	down
GOBP_HIS	0.507906	-0.01787	8.142528	2.62E-14	1.66E-13	21.85453	up
TRAVAGLI	-0.50781	0.074382	-9.63373	1.28E-18	1.81E-17	31.62034	down
SALVADOI	-0.50772	0.121168	-12.5817	7.39E-28	1.16E-25	52.60945	down
GOBP_KET	-0.50744	0.22468	-10.8443	2.51E-22	8.54E-21	40.03788	down
GOBP_MIT	-0.50739	0.168821	-8.54595	1.93E-15	1.47E-14	24.41708	down
HP_STERO	0.507386	-0.09694	9.566424	2.03E-18	2.73E-17	31.16362	up
GOMF_UBI	-0.50728	0.245403	-9.02902	7.84E-17	7.68E-16	27.56678	down
REACTOM	-0.50725	0.217143	-9.29246	1.32E-17	1.50E-16	29.31853	down
DESERT_PE	-0.50723	0.253965	-11.1182	3.47E-23	1.49E-21	41.98839	down
DESCARTE	-0.50719	0.014035	-7.95776	8.46E-14	4.96E-13	20.70324	down
GOBP_CAF	-0.50699	0.319428	-8.63783	1.06E-15	8.42E-15	25.00959	down
REACTOM	0.506907	-0.20665	8.800218	3.61E-16	3.14E-15	26.06447	up
HAY_BONI	-0.50644	0.007916	-9.56209	2.09E-18	2.80E-17	31.13428	down
AIZARANI_	-0.50637	0.23713	-11.4105	4.16E-24	2.21E-22	44.08403	down
GOBP_CEL	-0.50636	0.133589	-12.2154	1.12E-26	1.22E-24	49.92452	down
MORI_IMM	0.506313	-0.19832	9.505402	3.09E-18	3.98E-17	30.75066	up

GOBP_MES	-0.50628	0.056109	-10.2913	1.29E-20	2.92E-19	36.14838	down
MYLLYKAN	0.506252	-0.077	10.42056	5.18E-21	1.30E-19	37.05152	up
HP_OROTI	-0.50619	0.166113	-8.96912	1.17E-16	1.11E-15	27.17173	down
GOBP_MIN	-0.50572	0.14981	-10.3378	9.31E-21	2.20E-19	36.47276	down
GOMF_3_5	0.505554	-0.12109	9.956758	1.36E-19	2.39E-18	33.83045	up
BOYAULT_	-0.50541	-0.02554	-7.63604	6.29E-13	3.27E-12	18.73425	down
GOMF_RRI	0.505335	-0.20555	6.42584	7.69E-10	2.63E-09	11.78406	up
GOBP_LEN	-0.50532	0.015733	-9.60631	1.54E-18	2.14E-17	31.43414	down
GOBP_T_C	-0.50522	0.027228	-7.59649	8.03E-13	4.11E-12	18.49546	down
GOBP_PRC	0.505149	-0.18587	10.06587	6.32E-20	1.21E-18	34.58333	up
MIKKELSEN	-0.50505	0.064752	-7.36451	3.30E-12	1.55E-11	17.10971	down
HP_TALL_C	0.504754	-0.01595	9.888493	2.19E-19	3.67E-18	33.36099	up
HP_WIDE_I	-0.5046	-0.06004	-8.74564	5.18E-16	4.38E-15	25.70885	down
GOMF_ALI	-0.50459	-0.02883	-9.98154	1.14E-19	2.06E-18	34.00117	down
GOBP_RES	-0.50459	0.155687	-8.6882	7.57E-16	6.19E-15	25.33574	down
GOBP_CEL	-0.50459	0.155687	-8.6882	7.57E-16	6.19E-15	25.33574	down
BRUNEAU_	-0.5045	0.042146	-10.7756	4.11E-22	1.33E-20	39.55098	down
GOBP_LEU	-0.50438	-0.0423	-8.92459	1.58E-16	1.46E-15	26.87885	down
BIOCARTA	0.504364	-0.20785	10.60989	1.34E-21	3.85E-20	38.38111	up
WP_DEREG	-0.50435	0.095389	-12.8575	9.46E-29	1.86E-26	54.63988	down
REACTOM	-0.50414	0.210075	-10.5488	2.08E-21	5.67E-20	37.95152	down
FIGUEROA	-0.50406	0.042999	-9.1168	4.35E-17	4.48E-16	28.14794	down
chr2q22	-0.50394	0.102204	-9.32421	1.07E-17	1.23E-16	29.53113	down
GOMF_AC	-0.50386	0.281277	-9.36915	7.85E-18	9.30E-17	29.83268	down
REACTOM	-0.50382	0.105422	-10.2438	1.81E-20	3.94E-19	35.81789	down
MIR4485_5	-0.50361	-0.02322	-10.5559	1.98E-21	5.43E-20	38.00093	down
GOBP_GLY	-0.5036	0.18629	-9.97551	1.19E-19	2.13E-18	33.95964	down
GOBP_B_C	-0.50348	0.100732	-9.89661	2.07E-19	3.49E-18	33.41676	down
GRAHAM_	0.50346	-0.21347	9.15095	3.45E-17	3.64E-16	28.3747	up
GNF2_FGR	-0.50341	-0.05507	-7.64778	5.85E-13	3.06E-12	18.80531	down
WP_FATTY	-0.50341	0.287381	-9.21324	2.27E-17	2.46E-16	28.78932	down
MODULE_1	0.503201	-0.23411	8.684889	7.74E-16	6.32E-15	25.3143	up
GOBP_CEL	0.503048	-0.15884	9.669952	9.96E-19	1.44E-17	31.86673	up
MAGRANG	-0.50304	0.016093	-16.1743	1.37E-39	8.82E-36	79.29139	down
GOBP_NEG	-0.50276	0.085303	-9.27638	1.48E-17	1.66E-16	29.21093	down
REACTOM	-0.50269	0.114445	-12.5144	1.22E-27	1.82E-25	52.11494	down
HP_LYMPH	-0.50269	0.030838	-9.18612	2.72E-17	2.92E-16	28.60864	down
HP_CONG	0.502613	-0.00371	9.448942	4.55E-18	5.66E-17	30.36957	up
GNF2_TST	-0.50261	0.224013	-10.7937	3.61E-22	1.19E-20	39.67942	down
HP_ABNOI	-0.50253	0.116809	-9.29352	1.31E-17	1.49E-16	29.32562	down
REACTOM	0.502467	-0.20712	8.511441	2.42E-15	1.81E-14	24.19537	up
GOBP_IMN	-0.5023	-0.01835	-8.22545	1.54E-14	1.01E-13	22.37592	down
GOBP_NEG	0.502241	-0.18506	9.793308	4.24E-19	6.72E-18	32.70853	up
GOBP_CEN	0.502037	-0.19918	10.20652	2.35E-20	4.94E-19	35.55838	up
GNF2_PTP	-0.50197	0.048949	-8.85553	2.50E-16	2.23E-15	26.42598	down
REACTOM	-0.50188	0.09377	-11.0256	6.79E-23	2.68E-21	41.32753	down
GOCC_LIP	-0.50173	0.127477	-8.86577	2.34E-16	2.09E-15	26.49302	down
HP_HYPOC	-0.5017	0.165417	-10.31	1.13E-20	2.60E-19	36.27863	down
KEGG_MIS	0.501696	-0.13556	10.16065	3.25E-20	6.59E-19	35.23984	up
GOBP_REG	-0.50167	-0.01203	-8.94875	1.34E-16	1.26E-15	27.03766	down
GOBP_NUC	-0.50153	0.29592	-8.76325	4.61E-16	3.93E-15	25.82351	down
HP_HYPER	-0.50137	0.194069	-9.47409	3.83E-18	4.83E-17	30.53918	down
GOBP_POS	0.501107	-0.09025	9.544687	2.36E-18	3.12E-17	31.01638	up
GNF2_GST	-0.50094	0.227928	-10.6482	1.02E-21	3.02E-20	38.65133	down
GOBP_BEN	-0.50066	0.170748	-12.2405	9.31E-27	1.05E-24	50.10799	down
DESCARTE	-0.50046	0.023685	-7.89832	1.23E-13	7.05E-13	20.33593	down
HP_HYPOI	0.50037	-0.14291	10.20825	2.32E-20	4.89E-19	35.57042	up
GOBP_MA	-0.50036	-0.07374	-7.29271	5.09E-12	2.33E-11	16.68611	down

GOBP_G2_	0.500341	-0.10115	11.93427	8.94E-26	7.39E-24	47.8747	up
SHANK_TA	-0.50022	0.093998	-11.0135	7.41E-23	2.89E-21	41.24062	down

	logFC	AveExpr	t	P.Value	adj.P.Val	B	group
GOMF_PRI	1.241709	0.128146	21.64532	1.20E-48	3.49E-46	100.2554	up
GOMF_SNI	1.15141	0.18834	25.13814	1.78E-56	5.73E-53	118.1762	up
GOBP_NEC	-1.04755	-0.03467	-17.8959	1.67E-39	5.46E-38	79.29658	down
GOBP_NEC	-1.04595	-0.12641	-23.4338	9.73E-53	8.72E-50	109.6241	down
BIOCARTA	1.043776	0.170917	27.17455	9.55E-61	7.71E-57	127.9303	up
GOCC_PRE	1.041795	0.197273	24.18415	2.10E-54	4.53E-51	113.4337	up
GOMF_RRI	1.037349	0.100258	15.69413	8.41E-34	1.14E-32	66.21575	up
CROSBY_E	1.034418	-0.14197	16.30811	2.06E-35	3.47E-34	69.91425	up
KUMAMOT	1.033195	-0.1195	16.28428	2.37E-35	3.96E-34	69.77138	up
GOCC_NU	1.033165	0.112171	23.64215	3.33E-53	3.84E-50	110.6889	up
GOMF_RN	1.019264	-0.06741	18.0383	7.28E-40	2.52E-38	80.12444	up
GOCC_U7_	1.015878	0.173972	23.61705	3.79E-53	4.22E-50	110.5609	up
HP_CAUD/	-1.01456	0.039242	-20.6351	2.94E-46	4.39E-44	94.78293	down
MIR3912_3	-1.01451	-0.18231	-27.2864	5.65E-61	6.07E-57	128.4517	down
ZNF774_T/	1.014441	0.076778	26.24395	8.04E-59	5.19E-55	123.5343	up
WP_CELLU	1.006678	0.208332	22.39332	2.23E-50	1.00E-47	104.2233	up
CHASSOT_	-1.00139	0.068367	-17.8581	2.08E-39	6.69E-38	79.07673	down
GOBP_REG	-0.99976	-0.10213	-23.5295	5.95E-53	5.64E-50	110.1138	down
GOCC_U4_	0.996979	0.151722	25.45226	3.78E-57	1.52E-53	119.7133	up
GOBP_CHI	0.996669	0.09115	19.78206	3.38E-44	2.83E-42	90.06037	up
GOCC_PIC	0.992433	0.206153	19.04793	2.16E-42	1.20E-40	85.9228	up
GOCC_CO	0.992353	0.036929	21.20732	1.28E-47	2.90E-45	97.89874	up
chr4q33	-0.98165	-0.01533	-16.9185	5.33E-37	1.16E-35	73.55347	down
GOBP_POS	-0.97863	-0.05427	-19.3122	4.79E-43	3.06E-41	87.41972	down
HP_REDUC	-0.97704	-0.13898	-21.3939	4.67E-48	1.20E-45	98.90564	down
GOCC_PH(	-0.95928	-0.15736	-17.6647	6.47E-39	1.90E-37	77.94793	down
HP_INCRE/	-0.95812	0.072815	-15.7702	5.30E-34	7.41E-33	66.67598	down
GOBP_CAF	-0.95467	0.070568	-14.3962	2.40E-30	2.16E-29	58.2894	down
GOCC_BOI	0.950806	0.187119	17.32573	4.76E-38	1.20E-36	75.95953	up
HOWARD_	0.950749	-0.14707	14.85609	1.41E-31	1.47E-30	61.11306	up
SEMBA_FH	0.94937	-0.1216	16.03048	1.10E-34	1.68E-33	68.24633	up
GOBP_NEC	0.949017	0.248408	14.59271	7.14E-31	6.83E-30	59.49783	up
HP_LIMBA	-0.94651	-0.02971	-22.3537	2.74E-50	1.18E-47	104.0148	down
MODULE_3	0.945419	0.139873	22.43078	1.83E-50	8.54E-48	104.4201	up
GNF2_MKI	0.936664	-0.16748	14.41387	2.15E-30	1.95E-29	58.39835	up
WP_SOMA	-0.93649	-0.05031	-22.7875	2.80E-51	1.64E-48	106.2853	down
GOBP_AUT	-0.93293	-0.181	-16.727	1.67E-36	3.35E-35	72.41593	down
GOBP_PRC	-0.93263	-0.12029	-16.7175	1.77E-36	3.53E-35	72.35954	down
GNF2_BUB	0.93261	-0.14406	15.21001	1.61E-32	1.87E-31	63.27515	up
GOBP_OVU	-0.92792	-0.06981	-20.4476	8.28E-46	1.04E-43	93.75262	down
GOCC_EKC	0.926556	0.128663	19.65678	6.84E-44	5.31E-42	89.35906	up
GOBP_COF	-0.92613	-0.16693	-19.6807	5.97E-44	4.72E-42	89.49306	down
GOMF_CO	-0.92613	-0.16693	-19.6807	5.97E-44	4.72E-42	89.49306	down
GOMF_MC	-0.92312	0.086561	-13.8259	8.22E-29	6.31E-28	54.7696	down
GOBP_AOI	-0.92185	-0.03235	-17.0792	2.05E-37	4.72E-36	74.50523	down
ZHAN_MU	0.921524	-0.13758	15.78163	4.95E-34	6.94E-33	66.74501	up
CAO_BLOC	0.920126	-0.0852	17.9607	1.14E-39	3.83E-38	79.67369	up
HP_RECUR	-0.91907	0.126018	-15.3762	5.83E-33	7.14E-32	64.28713	down
TFDP2_TAI	0.918422	-0.02801	18.07965	5.72E-40	2.01E-38	80.36428	up
GNF2_CDC	0.917845	-0.15861	14.63287	5.57E-31	5.40E-30	59.74444	up
GNF2_RRM	0.917748	-0.17138	14.33169	3.58E-30	3.14E-29	57.89236	up
HP_PECTU	-0.91413	-0.21794	-17.6638	6.50E-39	1.91E-37	77.94253	down
GOBP_COI	-0.91375	-0.03891	-20.0556	7.31E-45	7.32E-43	91.58476	down
GOCC_MIT	0.912334	0.113128	19.87463	2.01E-44	1.78E-42	90.5773	up
GOBP_REG	-0.91073	0.026254	-19.3245	4.47E-43	2.87E-41	87.48955	down
GOBP_LEU	-0.90874	0.132437	-14.7417	2.85E-31	2.86E-30	60.41209	down
REACTOM	-0.90831	0.024801	-17.9027	1.61E-39	5.26E-38	79.33631	down

HP_DECRE	-0.90826	0.181784	-15.2015	1.70E-32	1.96E-31	63.22345	down
GNF2_CCN	0.908045	-0.16667	14.25396	5.78E-30	4.98E-29	57.4134	up
GOCC_TH(	0.90101	0.040738	20.57125	4.18E-46	6.00E-44	94.43256	up
GNF2_ESPI	0.900549	-0.16152	14.36745	2.87E-30	2.56E-29	58.11258	up
KALMA_E2	0.899817	-0.10371	15.32706	7.87E-33	9.46E-32	63.98796	up
GOBP_FEM	0.899377	-0.14013	15.2583	1.20E-32	1.41E-31	63.56936	up
GNF2_CEN	0.897682	-0.16557	14.34592	3.27E-30	2.90E-29	57.98003	up
GNF2_HMI	0.897078	-0.1801	13.97409	3.28E-29	2.62E-28	55.68592	up
GOBP_OM	-0.89666	0.098264	-16.0833	7.97E-35	1.24E-33	68.56434	down
REACTOM	-0.89666	0.098264	-16.0833	7.97E-35	1.24E-33	68.56434	down
HP_LYMPH	-0.89617	0.064907	-20.6525	2.67E-46	4.07E-44	94.87811	down
GNF2_CEN	0.894728	-0.16519	14.03255	2.28E-29	1.85E-28	56.04715	up
GOBP_NU(	-0.89423	0.083501	-14.8895	1.15E-31	1.21E-30	61.31727	down
GOCC_HO	0.894214	-0.14115	16.31753	1.94E-35	3.30E-34	69.97068	up
GOBP_L_K\	-0.88997	0.182796	-13.8804	5.86E-29	4.57E-28	55.10666	down
GOCC_ME	0.889147	0.14906	18.72732	1.35E-41	6.37E-40	84.09492	up
GNF2_CCN	0.888545	-0.17263	13.91512	4.72E-29	3.73E-28	55.32139	up
KANG_DO.	0.888379	-0.1422	14.25046	5.91E-30	5.08E-29	57.39186	up
GNF2_MCI	0.888061	-0.14925	14.40068	2.33E-30	2.11E-29	58.31715	up
GOCC_BO.	0.886412	0.105625	14.98076	6.56E-32	7.08E-31	61.8758	up
GNF2_CD(	0.885595	-0.17144	13.90901	4.91E-29	3.86E-28	55.28364	up
GNF2_CKS	0.885363	-0.09984	16.7601	1.37E-36	2.80E-35	72.61294	up
GOCC_BA:	-0.88529	-0.21118	-17.3791	3.47E-38	9.05E-37	76.27356	down
GOBP_NE(	-0.88476	-0.093	-13.2067	3.88E-27	2.52E-26	50.9296	down
GOCC_U1_	0.883408	0.077085	23.81593	1.37E-53	2.10E-50	111.573	up
GOBP_HIS	-0.88334	-0.13222	-18.3172	1.44E-40	5.64E-39	81.73872	down
GOMF_HIS	-0.88334	-0.13222	-18.3172	1.44E-40	5.64E-39	81.73872	down
GOBP_PO:	-0.88322	-0.04242	-12.8692	3.20E-26	1.91E-25	48.83066	down
REACTOM	0.882522	-0.14131	14.63663	5.44E-31	5.28E-30	59.76752	up
MONTERC	0.881481	-0.18849	14.35031	3.19E-30	2.82E-29	58.00708	up
GOBP_PO:	-0.88074	-0.09698	-23.2446	2.59E-52	2.04E-49	108.6522	down
GOBP_PO:	-0.88021	0.009094	-18.214	2.62E-40	9.93E-39	81.14205	down
REACTOM	-0.88017	0.11952	-15.7154	7.39E-34	1.01E-32	66.34474	down
GNF2_TTK	0.880029	-0.1775	13.80051	9.62E-29	7.35E-28	54.61237	up
GOBP_OU	0.879246	0.057585	15.33103	7.68E-33	9.25E-32	64.01213	up
GOMF_INT	-0.87887	0.04712	-19.2933	5.34E-43	3.38E-41	87.31306	down
SOTIRIOU_	0.878605	-0.08538	17.35927	3.91E-38	1.00E-36	76.15691	up
GOBP_PO:	-0.87815	-0.12127	-17.1203	1.61E-37	3.74E-36	74.74805	down
chr11p12	-0.87779	-0.01663	-19.1865	9.79E-43	5.85E-41	86.70912	down
FINETTI_BF	0.87761	-0.19169	13.9996	2.80E-29	2.25E-28	55.8436	up
HP_ENDO(	0.876489	-0.05933	17.0005	3.27E-37	7.29E-36	74.03955	up
IIZUKA_LIV	-0.87526	0.11742	-18.7504	1.18E-41	5.66E-40	84.22695	down
GNF2_H2A	0.875064	-0.18307	13.673	2.13E-28	1.56E-27	53.82276	up
OHASHI_A	0.874993	-0.01951	15.81702	3.99E-34	5.68E-33	66.95889	up
GOBP_DO	0.872329	-0.15481	14.00069	2.78E-29	2.24E-28	55.8503	up
GOCC_CO	0.871553	-0.17889	16.51869	5.80E-36	1.07E-34	71.17414	up
WP_NICOT	-0.87147	0.089633	-14.213	7.45E-30	6.34E-29	57.16106	down
GOMF_PO	0.871157	0.022279	14.83705	1.59E-31	1.64E-30	60.99647	up
GNF2_CKS	0.87008	-0.13982	15.2841	1.02E-32	1.21E-31	63.72646	up
GOBP_REG	-0.86835	0.05546	-21.1819	1.47E-47	3.22E-45	97.76115	down
LY_AGING_	0.868092	-0.15706	14.17878	9.21E-30	7.75E-29	56.94983	up
GOBP_TRI(	-0.86784	-0.06892	-19.6636	6.58E-44	5.14E-42	89.39719	down
GNF2_PCN	0.86655	-0.16749	13.99971	2.79E-29	2.25E-28	55.84428	up
GOCC_LSN	0.863877	0.241488	15.65679	1.06E-33	1.41E-32	65.98969	up
GOBP_CILI	-0.8638	-0.06944	-19.088	1.72E-42	9.81E-41	86.15033	down
GOBP_NE(	-0.86373	0.011428	-17.703	5.17E-39	1.54E-37	78.172	down
GOBP_CAI	-0.86306	0.017572	-15.1897	1.82E-32	2.10E-31	63.15115	down
HESSON_T	0.861612	0.113767	20.93103	5.79E-47	1.09E-44	96.39951	up

GOBP_RET	-0.86139	-0.00544	-15.7255	6.95E-34	9.58E-33	66.40569	down
HP_ABNOI	-0.86116	-0.07311	-22.9285	1.34E-51	9.00E-49	107.0184	down
REACTOM	0.860962	0.088854	21.87418	3.52E-49	1.17E-46	101.477	up
BOYAULT_	-0.85865	0.155063	-13.6512	2.44E-28	1.78E-27	53.68754	down
BURTON_/	0.858561	-0.04442	20.84463	9.30E-47	1.64E-44	95.92867	up
HP_ABNOI	-0.85743	-0.01636	-16.646	2.71E-36	5.26E-35	71.93344	down
GNF2_SM(	0.856371	-0.18699	13.68632	1.96E-28	1.44E-27	53.90532	up
GOMF_K48	0.854804	0.074072	16.11256	6.68E-35	1.05E-33	68.74025	up
GOCC_CHI	0.853292	-0.1288	14.72949	3.07E-31	3.07E-30	60.33726	up
REACTOM	0.853135	0.082875	18.61559	2.57E-41	1.16E-39	83.45495	up
ISHIDA_E2	0.853068	-0.14239	15.17952	1.94E-32	2.22E-31	63.08928	up
GOBP_REG	0.849752	0.074302	18.05813	6.48E-40	2.26E-38	80.23947	up
GOCC_U6_	0.849582	0.163491	18.71176	1.48E-41	6.93E-40	84.00587	up
CROONQU	0.849501	-0.10988	16.1979	3.99E-35	6.43E-34	69.25303	up
ZHONG_PI	0.848387	-0.13911	15.66829	9.85E-34	1.32E-32	66.05931	up
GNF2_BUB	0.848124	-0.15175	14.0591	1.93E-29	1.58E-28	56.21111	up
HP_OVARI	-0.8481	-0.11787	-18.1716	3.35E-40	1.24E-38	80.89706	down
SMID_BRE/	-0.84782	0.079708	-15.4689	3.31E-33	4.17E-32	64.85022	down
GOBP_REG	-0.84744	-0.0683	-15.3701	6.05E-33	7.39E-32	64.24974	down
GOBP_ABS	0.847404	0.060487	17.37912	3.47E-38	9.05E-37	76.27362	up
GOCC_CM	0.8467	-0.16586	12.09494	4.05E-24	2.00E-23	44.01085	up
REACTOM	-0.84427	0.195265	-15.3859	5.50E-33	6.76E-32	64.3456	down
GOMF_N_I	-0.84414	0.090672	-10.8114	1.22E-20	4.59E-20	36.04702	down
WP_ESTRC	-0.8428	0.036496	-18.7147	1.45E-41	6.83E-40	84.02297	down
GOCC_MIT	-0.84279	0.034005	-8.24239	6.77E-14	1.62E-13	20.63926	down
GAO_LAR(	0.842	-0.14765	14.4141	2.15E-30	1.94E-29	58.39973	up
HP_IRREGU	-0.84192	-0.11846	-16.7073	1.88E-36	3.73E-35	72.29858	down
GOMF_FAT	-0.84116	0.144581	-12.2907	1.19E-24	6.16E-24	45.2298	down
GOCC_MIT	-0.841	0.114451	-13.0748	8.85E-27	5.58E-26	50.10935	down
REACTOM	-0.84087	-0.06449	-19.2873	5.52E-43	3.48E-41	87.27956	down
NIKOLSKY_	0.840208	-0.03435	18.91723	4.55E-42	2.37E-40	85.17918	up
GOCC_MU	0.839956	0.139812	21.24492	1.05E-47	2.41E-45	98.10202	up
GOMF_RIB	0.839956	0.139812	21.24492	1.05E-47	2.41E-45	98.10202	up
GOMF_RIB	0.839956	0.139812	21.24492	1.05E-47	2.41E-45	98.10202	up
GOMF_AC	-0.83985	0.152385	-12.677	1.06E-25	6.04E-25	47.63452	down
MINGUEZ_	-0.83886	0.118919	-13.7023	1.77E-28	1.32E-27	54.00413	down
HP_DECRE	-0.83883	0.094438	-15.5526	1.99E-33	2.58E-32	65.35829	down
GOMF_MIT	0.838411	0.010595	14.96734	7.12E-32	7.67E-31	61.79372	up
GOBP_MIT	-0.83837	-0.00144	-16.456	8.45E-36	1.52E-34	70.7997	down
REACTOM	-0.83782	0.214367	-12.3286	9.39E-25	4.91E-24	45.46568	down
GOCC_AST	-0.83765	0.018175	-18.6693	1.89E-41	8.70E-40	83.76289	down
GTCGATC_	-0.83749	-0.22959	-16.3836	1.31E-35	2.29E-34	70.36641	down
GOBP_PRE	0.837468	-0.16219	13.2075	3.87E-27	2.51E-26	50.93441	up
GOCC_RIB	0.836511	0.186892	18.10177	5.03E-40	1.79E-38	80.49256	up
GOBP_TRN	0.836256	0.150984	14.91526	9.80E-32	1.04E-30	61.47521	up
GOMF_BIC	-0.836	0.090295	-12.6294	1.43E-25	8.02E-25	47.33859	down
GOBP_SPH	-0.83526	0.043837	-16.17	4.72E-35	7.52E-34	69.08548	down
DESCARTE	-0.83513	0.050857	-24.4607	5.21E-55	1.20E-51	114.82	down
REACTOM	0.834929	-0.17915	11.98272	8.17E-24	3.95E-23	43.31232	up
ROSTY_CE	0.834781	-0.1298	14.67266	4.36E-31	4.28E-30	59.98868	up
GOCC_CO	0.834401	-0.16028	12.55503	2.28E-25	1.25E-24	46.87542	up
GOCC_ALI	0.833856	-0.18358	12.4394	4.70E-25	2.51E-24	46.15547	up
GOMF_GL	-0.83329	0.128466	-14.3511	3.17E-30	2.81E-29	58.01203	down
GOBP_RN/	0.832964	0.016323	15.45281	3.65E-33	4.58E-32	64.75253	up
BIOCARTA	-0.83293	0.182006	-15.2576	1.20E-32	1.41E-31	63.56511	down
GOBP_LUN	-0.83235	0.035332	-18.7128	1.47E-41	6.90E-40	84.01173	down
GOCC_DN	0.832239	-0.0529	15.33938	7.30E-33	8.83E-32	64.06295	up
GOBP_POS	-0.83182	0.05309	-13.6603	2.30E-28	1.69E-27	53.74389	down

GOCC_MIT	-0.82958	0.110081	-12.8234	4.25E-26	2.51E-25	48.54606	down
GNF2_FEN	0.829032	-0.13486	15.09731	3.21E-32	3.60E-31	62.58776	up
GOBP_NEC	-0.82836	-0.12142	-10.9424	5.39E-21	2.09E-20	36.85594	down
BIOCARTA	-0.82718	0.141309	-16.5534	4.71E-36	8.83E-35	71.38153	down
HP_DEEP_S	0.827142	-0.1444	15.91513	2.20E-34	3.24E-33	67.55115	up
RICHERT_F	-0.82694	0.242786	-11.1173	1.81E-21	7.30E-21	37.93775	down
GOMF_EST	-0.82694	0.159831	-13.5621	4.24E-28	3.01E-27	53.13541	down
SIMBULAN	0.82687	-0.10845	16.77955	1.22E-36	2.51E-35	72.7286	up
GOMF_NA	-0.82623	0.011673	-20.2932	1.95E-45	2.15E-43	92.90108	down
GOBP_NEC	-0.82608	-0.03502	-20.9544	5.10E-47	9.74E-45	96.52666	down
FARMER_B	0.82565	-0.15475	14.70308	3.61E-31	3.58E-30	60.17531	up
GOMF_LIG	-0.82408	0.124548	-12.1028	3.86E-24	1.91E-23	44.05979	down
GOCC_FIBI	-0.82403	0.153718	-13.7325	1.47E-28	1.10E-27	54.19142	down
LAKE_ADU	-0.82399	-0.05243	-17.7784	3.32E-39	1.02E-37	78.61207	down
GOCC_DN	0.823603	-0.1657	11.98999	7.81E-24	3.78E-23	43.35756	up
YAGUE_PR	-0.82345	0.016294	-19.9407	1.39E-44	1.29E-42	90.9454	down
GOBP_RES	-0.82333	0.074076	-11.87	1.65E-23	7.81E-23	42.61074	down
MODULE_	0.822881	-0.08978	16.58119	3.99E-36	7.58E-35	71.54724	up
CAO_BLOC	-0.82277	0.000467	-15.8266	3.77E-34	5.39E-33	67.017	down
GOBP_REG	-0.82231	-0.02076	-19.7388	4.31E-44	3.52E-42	89.81852	down
GOBP_NO	-0.82169	0.080689	-16.6648	2.42E-36	4.73E-35	72.04563	down
EGUCHI_CI	0.820989	-0.21663	12.46679	3.96E-25	2.13E-24	46.32602	up
GOBP_REG	-0.82098	-0.04717	-16.7821	1.20E-36	2.48E-35	72.74371	down
GOBP_AM	-0.82072	-0.07901	-16.873	6.99E-37	1.49E-35	73.28346	down
MODULE_	0.820469	-0.17327	15.33127	7.67E-33	9.24E-32	64.0136	up
MODULE_	0.818521	-0.10603	13.24116	3.13E-27	2.05E-26	51.14349	up
GOBP_DN	0.817595	-0.08245	16.46079	8.21E-36	1.48E-34	70.82822	up
GOMF_AC	-0.81741	0.12239	-14.0459	2.10E-29	1.71E-28	56.12933	down
GNF2_RRN	0.81689	-0.12572	15.36851	6.11E-33	7.45E-32	64.24011	up
WHITFIELD	0.816561	-0.15267	14.0807	1.69E-29	1.39E-28	56.34452	up
GOMF_C_X	-0.81615	-0.25029	-15.479	3.11E-33	3.94E-32	64.91178	down
GOMF_SPH	-0.81527	0.047935	-13.0643	9.45E-27	5.93E-26	50.04405	down
GOBP_NO	-0.81501	0.013115	-20.2385	2.64E-45	2.84E-43	92.59892	down
REACTOM	-0.81473	0.215383	-14.3065	4.18E-30	3.65E-29	57.73693	down
GOBP_REG	-0.81468	-0.06441	-15.4245	4.34E-33	5.41E-32	64.58059	down
GOBP_MO	-0.81417	0.169014	-13.97	3.36E-29	2.69E-28	55.66068	down
RHODES_U	0.813918	-0.04705	19.19659	9.24E-43	5.58E-41	86.76606	up
REACTOM	-0.81389	0.175133	-11.2959	5.96E-22	2.48E-21	39.04466	down
GOBP_NEC	-0.81255	0.015361	-17.0685	2.18E-37	5.01E-36	74.44215	down
GOCC_CYT	0.811771	0.26855	16.74314	1.52E-36	3.07E-35	72.51207	up
GOCC_EXT	-0.81087	0.071259	-14.5472	9.44E-31	8.88E-30	59.2185	down
GOCC_PRC	-0.81007	0.151412	-8.92294	1.23E-15	3.29E-15	24.60539	down
REACTOM	-0.81007	0.151412	-8.92294	1.23E-15	3.29E-15	24.60539	down
REACTOM	-0.80998	0.154106	-14.489	1.35E-30	1.25E-29	58.86047	down
REACTOM	-0.80918	0.181967	-12.7453	6.93E-26	4.01E-25	48.0598	down
GOBP_TRIC	-0.80869	-0.0804	-16.2515	2.89E-35	4.77E-34	69.57451	down
TOMIDA_L	0.807465	-0.02129	14.48783	1.36E-30	1.26E-29	58.8533	up
GOBP_TRA	0.807349	-0.01336	19.21267	8.43E-43	5.16E-41	86.85716	up
KUWANO_	-0.80705	0.159917	-15.6646	1.01E-33	1.35E-32	66.03725	down
GOBP_POS	0.80579	-0.03368	12.54321	2.45E-25	1.35E-24	46.80186	up
GOBP_POS	-0.80566	-0.07408	-18.0006	9.06E-40	3.08E-38	79.90568	down
HP_ABNOI	-0.80536	0.023276	-13.0686	9.20E-27	5.78E-26	50.07095	down
FAN_EMBR	0.804569	-0.13653	14.43017	1.95E-30	1.77E-29	58.49861	up
SCIAN_CEL	0.80418	-0.08098	15.6606	1.03E-33	1.38E-32	66.01278	up
GOBP_LEU	-0.80384	-0.00492	-17.1601	1.27E-37	3.01E-36	74.98347	down
ZERBINI_R	0.802192	-0.0622	16.56677	4.35E-36	8.21E-35	71.46118	up
GOBP_SHC	-0.80201	0.188577	-12.2174	1.88E-24	9.56E-24	44.77346	down
GNF2_RFC	0.800725	-0.14184	14.36929	2.83E-30	2.53E-29	58.12395	up

GOBP_COI	-0.80031	0.184922	-15.6516	1.09E-33	1.45E-32	65.95801	down
GOMF_SUI	0.799976	0.005335	14.71947	3.27E-31	3.25E-30	60.27585	up
GOBP_FAT	-0.7999	0.177935	-13.0076	1.35E-26	8.34E-26	49.69202	down
GOMF_RN.	-0.79907	-0.06121	-12.4502	4.39E-25	2.35E-24	46.22305	down
SCHMAHL_	-0.79834	-0.06194	-17.5753	1.09E-38	3.09E-37	77.42469	down
GOCC_SPL	0.797977	0.103442	18.34907	1.20E-40	4.79E-39	81.92229	up
LOPEZ_ME	-0.79745	0.138243	-15.0691	3.82E-32	4.23E-31	62.41553	down
HP_CORTI(	-0.79744	-0.07921	-16.1081	6.86E-35	1.08E-33	68.71321	down
HP_PROMI	-0.79731	-0.05535	-19.7491	4.07E-44	3.34E-42	89.87593	down
BIOCARTA	-0.79728	0.015523	-16.9574	4.23E-37	9.29E-36	73.78421	down
LOPEZ_ME	0.79723	-0.13197	16.87047	7.09E-37	1.52E-35	73.26869	up
CROONQU	0.796947	-0.12262	15.27759	1.07E-32	1.26E-31	63.68686	up
BOYAULT_	-0.79686	0.153052	-12.8198	4.35E-26	2.57E-25	48.52325	down
IIZUKA_LIV	0.79662	-0.01607	18.95011	3.77E-42	2.01E-40	85.36646	up
GOBP_AM	-0.79616	0.104023	-12.214	1.92E-24	9.75E-24	44.75186	down
REACTOM	-0.79539	0.154278	-11.3206	5.11E-22	2.14E-21	39.19797	down
TRAVAGLI	0.7952	-0.05854	16.83367	8.83E-37	1.86E-35	73.05017	up
GOBP_NE(	0.795075	-0.03995	18.89368	5.20E-42	2.69E-40	85.04494	up
GOBP_TRN	0.794622	0.145663	19.59449	9.71E-44	7.23E-42	89.00963	up
GROSS_HI	-0.79431	0.034049	-15.4507	3.70E-33	4.64E-32	64.73951	down
chr1q22	0.794175	-0.02292	19.65537	6.89E-44	5.33E-42	89.35115	up
GOBP_NE(	-0.79409	0.009166	-16.3085	2.05E-35	3.47E-34	69.91643	down
GOCC_AC	-0.79382	-0.00489	-16.4943	6.72E-36	1.23E-34	71.0285	down
GOMF_ALI	-0.79365	0.228375	-10.9553	4.97E-21	1.93E-20	36.93553	down
GOCC_TR/	0.793502	-0.00128	21.24988	1.02E-47	2.40E-45	98.12877	up
TURASHVI	-0.79332	-0.0229	-16.7242	1.70E-36	3.40E-35	72.3993	down
GOBP_ATR	-0.79304	0.109194	-18.7395	1.26E-41	5.98E-40	84.16483	down
GOBP_POS	-0.7926	-0.01718	-18.9444	3.89E-42	2.07E-40	85.33421	down
GOBP_ACY	-0.79098	0.032268	-14.2372	6.42E-30	5.49E-29	57.30989	down
GOBP_CEL	-0.79028	0.031632	-15.2373	1.36E-32	1.59E-31	63.44123	down
HP_ABSEN	0.790224	-0.07188	14.7052	3.57E-31	3.53E-30	60.18832	up
CAFFAREL_	-0.79022	-0.03052	-15.0458	4.40E-32	4.83E-31	62.27351	down
GOBP_REG	-0.78982	0.029175	-13.7997	9.67E-29	7.38E-28	54.60716	down
GOBP_PRE	-0.78909	-0.06265	-16.3749	1.38E-35	2.40E-34	70.31416	down
MODULE_	0.788852	-0.11759	14.80281	1.96E-31	2.00E-30	60.78668	up
GOCC_DN.	0.787932	-0.01266	22.21316	5.78E-50	2.36E-47	103.2742	up
GOBP_NEU	-0.78625	0.147504	-13.507	5.97E-28	4.18E-27	52.79403	down
MODULE_	0.786021	0.27302	13.84299	7.39E-29	5.70E-28	54.87524	up
GOBP_SUC	-0.78592	0.195235	-10.7068	2.32E-20	8.60E-20	35.40236	down
BIOCARTA	-0.78563	0.150523	-14.1282	1.26E-29	1.05E-28	56.63752	down
TCGA_GLI(	-0.78422	-0.11017	-18.6876	1.70E-41	7.89E-40	83.86741	down
GOMF_TRI	0.783442	0.13281	19.99367	1.03E-44	9.92E-43	91.24048	up
CAIRO_HE	-0.7833	0.151848	-16.3009	2.15E-35	3.61E-34	69.87112	down
WP_ARYLA	-0.78329	0.169231	-12.1164	3.54E-24	1.76E-23	44.14448	down
HP_MATEF	-0.78318	-0.01822	-13.2271	3.42E-27	2.23E-26	51.05638	down
RICKMAN_	0.781882	-0.0168	17.08111	2.03E-37	4.67E-36	74.51651	up
GOCC_GR(	-0.78172	0.176787	-14.7977	2.02E-31	2.06E-30	60.7555	down
GOMF_AC	-0.78167	0.192529	-11.2331	8.82E-22	3.63E-21	38.655	down
GOCC_TEL	0.780855	-0.04046	20.77011	1.40E-46	2.28E-44	95.52179	up
HP_SYNO\	-0.78083	-0.11173	-19.2621	6.37E-43	3.97E-41	87.13668	down
MORF_GP)	0.779261	0.153191	17.36834	3.70E-38	9.56E-37	76.21023	up
GOMF_PH(	-0.77895	-0.19286	-15.454	3.63E-33	4.55E-32	64.76002	down
chr8p22	-0.77731	0.009616	-16.9124	5.52E-37	1.20E-35	73.51753	down
GOBP_MRI	0.777099	-0.0408	12.86934	3.19E-26	1.91E-25	48.83178	up
REACTOM	0.776823	-0.12717	13.93491	4.18E-29	3.31E-28	55.44373	up
GOCC_U2_	0.776548	0.064644	18.12541	4.38E-40	1.58E-38	80.62955	up
WP_EICOS	-0.7765	0.166224	-13.3719	1.39E-27	9.37E-27	51.95511	down
GOMF_CY	-0.77649	-0.15072	-13.749	1.33E-28	9.98E-28	54.29327	down

HP_PROTE	-0.77637	0.129844	-11.6198	7.90E-23	3.54E-22	41.05541	down
REACTOM	-0.77624	0.088596	-14.6941	3.82E-31	3.77E-30	60.12036	down
HP_ABNOI	-0.776	0.057267	-13.3385	1.71E-27	1.14E-26	51.74784	down
GOBP_CHI	0.775361	-0.04269	20.13028	4.82E-45	4.97E-43	91.99928	up
GOBP_CAI	0.775299	0.059261	17.9173	1.47E-39	4.85E-38	79.42135	up
GOBP_NE(	0.775039	-0.03587	16.1418	5.60E-35	8.85E-34	68.91601	up
WP_SYNTH	-0.77473	0.164801	-13.1779	4.65E-27	2.99E-26	50.75067	down
HP_ABNOI	-0.77466	0.069914	-12.67	1.11E-25	6.30E-25	47.59114	down
GOMF_ON	-0.77433	-0.02067	-15.1473	2.36E-32	2.69E-31	62.89276	down
GOMF_AL(	-0.77426	0.148501	-10.95	5.14E-21	1.99E-20	36.90314	down
ZHOU_CEL	0.773881	-0.11688	14.56419	8.51E-31	8.05E-30	59.32268	up
GOCC_ME	-0.77355	0.225413	-12.9168	2.37E-26	1.44E-25	49.12725	down
HP_REDUC	-0.77345	-0.14608	-16.8072	1.03E-36	2.15E-35	72.89295	down
HP_DIGEN	-0.77344	-0.05699	-22.6482	5.81E-51	3.07E-48	105.559	down
GOBP_HIS	0.773252	-0.04987	16.0878	7.75E-35	1.21E-33	68.59132	up
HP_MICRC	0.771674	-0.12031	14.18057	9.11E-30	7.67E-29	56.96088	up
ODONNEL	0.771562	-0.14229	15.11641	2.86E-32	3.22E-31	62.70431	up
GOBP_MEI	-0.77135	-0.00565	-15.1271	2.67E-32	3.03E-31	62.76953	down
WP_BIOTII	-0.77111	0.112394	-11.6756	5.57E-23	2.53E-22	41.40186	down
GUTIERREZ	-0.76994	0.148683	-13.6481	2.48E-28	1.81E-27	53.6687	down
KEGG_DN/	0.769207	-0.06563	16.38702	1.28E-35	2.25E-34	70.38688	up
GOBP_MA	-0.76873	0.01132	-17.3574	3.95E-38	1.01E-36	76.14612	down
GOBP_REG	-0.7686	-0.06942	-18.7882	9.52E-42	4.67E-40	84.44326	down
GOMF_EST	-0.76802	0.113626	-13.1265	6.41E-27	4.07E-26	50.43121	down
REACTOM	0.767911	0.064109	14.10654	1.44E-29	1.19E-28	56.50408	up
GOMF_OX	-0.76761	0.157758	-13.9985	2.81E-29	2.26E-28	55.83709	down
GOBP_NIT	-0.76618	0.002882	-19.1477	1.22E-42	7.19E-41	86.48883	down
GESERICK_	-0.7661	0.065554	-16.7246	1.69E-36	3.40E-35	72.40151	down
HP_ABNOI	-0.76423	0.124186	-12.2328	1.71E-24	8.72E-24	44.86898	down
HP_BIFID_l	-0.76409	-0.06958	-15.8129	4.09E-34	5.81E-33	66.93421	down
REACTOM	-0.76405	0.109195	-11.3179	5.20E-22	2.18E-21	39.18078	down
GOBP_MRI	0.763681	-0.12743	14.94331	8.25E-32	8.83E-31	61.64676	up
GOBP_KYN	-0.76358	0.165154	-13.6892	1.92E-28	1.42E-27	53.92298	down
GOBP_NE(	-0.76346	0.033899	-16.59	3.79E-36	7.22E-35	71.59998	down
GOCC_HO	-0.76338	-0.04554	-13.9812	3.13E-29	2.51E-28	55.73007	down
GOBP_NE(	-0.76328	0.054348	-16.9919	3.44E-37	7.65E-36	73.98851	down
REACTOM	0.763209	-0.04679	12.05304	5.26E-24	2.58E-23	43.75001	up
WP_HFE_E	-0.76302	0.096991	-13.3906	1.23E-27	8.37E-27	52.07162	down
FAN_EMBI	0.761904	-0.09856	16.72672	1.67E-36	3.36E-35	72.41436	up
GOCC_NU	0.761729	0.017804	19.60897	8.95E-44	6.75E-42	89.09088	up
GOBP_REG	0.760833	-0.11871	14.54527	9.56E-31	8.98E-30	59.20643	up
chr9p23	-0.76067	-0.02184	-13.3794	1.32E-27	8.96E-27	52.0019	down
GOBP_REG	-0.76053	-0.1346	-14.7315	3.03E-31	3.03E-30	60.34975	down
GOMF_AM	-0.76043	0.11477	-12.4869	3.49E-25	1.89E-24	46.4511	down
HP_CRYPT	-0.76015	0.057199	-13.3617	1.48E-27	9.94E-27	51.89225	down
GOBP_NE(	-0.75955	-0.05667	-15.1953	1.76E-32	2.03E-31	63.1857	down
CTACTAG_	-0.75902	-0.04386	-19.9018	1.73E-44	1.56E-42	90.72857	down
WP_UREA_	-0.75881	0.109803	-12.1484	2.90E-24	1.45E-23	44.34387	down
YAO_TEMI	-0.75872	0.088917	-17.5831	1.04E-38	2.96E-37	77.47035	down
GOMF_N_/	-0.75848	-0.00288	-16.146	5.46E-35	8.64E-34	68.94113	down
YU_MYC_T	0.758226	-0.11616	16.44953	8.79E-36	1.58E-34	70.76089	up
GOBP_POS	-0.75801	-0.15891	-13.9426	3.98E-29	3.17E-28	55.49143	down
REACTOM	0.757708	-0.08119	15.0081	5.55E-32	6.03E-31	62.0429	up
AMIT_DEL/	-0.75716	0.059599	-15.6446	1.14E-33	1.51E-32	65.91588	down
WP_PENT(	0.757102	0.033087	14.3487	3.22E-30	2.85E-29	57.99712	up
GOBP_REG	-0.75704	0.062173	-13.1607	5.17E-27	3.32E-26	50.64383	down
GOMF_OX	-0.75643	-0.01718	-15.101	3.14E-32	3.52E-31	62.61049	down
FRASOR_R	0.756382	-0.10521	15.98345	1.46E-34	2.20E-33	67.96307	up

HP_PROLC	-0.75607	0.047858	-14.787	2.16E-31	2.19E-30	60.68976	down
GOBP_SKE	-0.75552	-0.03297	-15.5622	1.88E-33	2.44E-32	65.41616	down
UZONYI_R	-0.75539	0.037666	-16.59	3.79E-36	7.22E-35	71.59994	down
HP_ABNOI	-0.75517	-0.1554	-15.8109	4.14E-34	5.88E-33	66.92167	down
GOBP_CEL	-0.75506	0.041247	-19.9936	1.03E-44	9.92E-43	91.23989	down
GOBP_NU(	0.754844	0.134044	14.40183	2.32E-30	2.09E-29	58.32421	up
GOBP_CEL	0.754785	-0.21085	11.21554	9.84E-22	4.04E-21	38.5464	up
QI_PBMC_	0.75458	-0.15892	13.15835	5.25E-27	3.36E-26	50.62896	up
GOBP_DOI	0.754167	-0.19486	12.21863	1.87E-24	9.49E-24	44.78087	up
TURJANSK	-0.75345	-0.1398	-14.8346	1.61E-31	1.66E-30	60.98155	down
HP_SKULL_	-0.75302	-0.03779	-14.9725	6.90E-32	7.44E-31	61.82558	down
GOBP_NE(	-0.75211	0.016845	-16.6022	3.52E-36	6.73E-35	71.67235	down
REACTOM	-0.75176	0.227906	-12.477	3.71E-25	2.00E-24	46.38947	down
GOMF_TR/	-0.75171	-0.01338	-17.3616	3.85E-38	9.94E-37	76.17035	down
GOBP_POS	-0.7517	0.036146	-15.5034	2.69E-33	3.43E-32	65.05953	down
GOBP_EPC	-0.75162	0.146248	-14.1122	1.39E-29	1.15E-28	56.53925	down
MODULE_	0.751311	-0.1192	14.51663	1.14E-30	1.06E-29	59.0304	up
GOBP_IMN	-0.75129	0.162179	-12.4228	5.21E-25	2.78E-24	46.05195	down
HP_INCRE/	-0.75125	-0.00904	-17.706	5.08E-39	1.52E-37	78.18922	down
CASP3_TAI	-0.75113	0.179513	-8.88056	1.59E-15	4.21E-15	24.35526	down
GOBP_ON(	-0.75086	-0.01114	-15.6992	8.16E-34	1.11E-32	66.24652	down
MODULE_	-0.75005	0.161995	-12.7465	6.88E-26	3.98E-25	48.06722	down
HP_HYPER	-0.74956	0.105754	-11.0514	2.73E-21	1.08E-20	37.5302	down
GOCC_TRI	0.74899	-0.03646	13.37392	1.37E-27	9.26E-27	51.96797	up
GOBP_REG	-0.74848	0.088045	-13.0241	1.21E-26	7.56E-26	49.79456	down
HP_ANGIC	-0.74833	-0.02466	-18.9451	3.88E-42	2.06E-40	85.33776	down
GOBP_PRC	0.748144	-0.12549	16.70076	1.95E-36	3.87E-35	72.25987	up
REACTOM	0.747916	-0.19143	13.98982	2.97E-29	2.39E-28	55.78316	up
GOBP_REG	-0.74727	-0.08104	-15.6553	1.07E-33	1.42E-32	65.98053	down
REACTOM	-0.74722	-0.05478	-20.4178	9.76E-46	1.19E-43	93.58852	down
GOMF_LAI	-0.74717	-0.08103	-16.285	2.36E-35	3.95E-34	69.77588	down
GNF2_GLT	0.74705	0.303528	13.50125	6.19E-28	4.33E-27	52.75808	up
HP_RETIN/	-0.74696	0.162938	-9.7055	1.10E-17	3.37E-17	29.28507	down
GOCC_CYT	0.746637	-0.01043	14.30793	4.14E-30	3.62E-29	57.74604	up
GOBP_CEL	-0.74645	-0.08302	-12.989	1.51E-26	9.33E-26	49.57636	down
GOMF_PYI	0.746225	-0.05598	14.91523	9.81E-32	1.04E-30	61.475	up
GOCC_R2T	0.74621	0.053041	12.84201	3.79E-26	2.25E-25	48.66169	up
GOBP_NE(	-0.74568	0.15446	-15.5268	2.33E-33	3.00E-32	65.20142	down
HP_DYSGE	-0.7456	-0.10398	-16.5248	5.59E-36	1.04E-34	71.21088	down
KONG_E2F	0.74526	-0.15192	13.71765	1.61E-28	1.20E-27	54.09937	up
GOCC_CO	0.745037	-0.13035	15.83609	3.56E-34	5.09E-33	67.07409	up
REACTOM	-0.74484	-0.08512	-15.7363	6.51E-34	9.00E-33	66.47088	down
GNF2_SM(	0.744162	-0.13772	14.21087	7.55E-30	6.42E-29	57.14775	up
OHASHI_A	0.744037	-0.14327	13.90475	5.04E-29	3.96E-28	55.25724	up
GOBP_MIT	0.743861	-0.15952	13.23475	3.26E-27	2.13E-26	51.1037	up
REACTOM	-0.74293	0.221473	-13.5581	4.35E-28	3.08E-27	53.1106	down
REACTOM	-0.74288	-0.10295	-14.2304	6.69E-30	5.71E-29	57.26804	down
GOBP_EMI	-0.74283	-0.11531	-14.1327	1.23E-29	1.02E-28	56.66556	down
GOMF_LAI	0.742815	0.310424	11.88211	1.53E-23	7.27E-23	42.68631	up
HP_ADREN	0.742322	0.068237	15.92331	2.10E-34	3.09E-33	67.60052	up
GOBP_LIPI	-0.74224	0.182133	-12.1769	2.43E-24	1.22E-23	44.52125	down
GNF2_RFC	0.741836	-0.14249	14.20561	7.80E-30	6.62E-29	57.11533	up
TURJANSK	-0.74182	-0.06537	-14.3756	2.73E-30	2.44E-29	58.16264	down
GAUTSCHI	-0.7418	0.128753	-13.0464	1.06E-26	6.61E-26	49.93332	down
GRAHAM_	0.741767	-0.10519	15.03669	4.65E-32	5.09E-31	62.2176	up
MORF_FBL	0.741636	0.088489	18.54942	3.76E-41	1.65E-39	83.07523	up
WP_FATTY	-0.74122	0.179431	-11.8787	1.57E-23	7.41E-23	42.66481	down
LOPEZ_ME	-0.74068	0.150272	-12.3982	6.08E-25	3.22E-24	45.89918	down

chr13q22	-0.74006	0.071477	-17.9939	9.42E-40	3.20E-38	79.86687	down
HP_HEMAI	-0.73996	-0.05075	-22.1043	1.03E-49	3.82E-47	102.6984	down
DESERT_PE	-0.73993	0.149851	-13.4894	6.67E-28	4.64E-27	52.68469	down
MORI_LAR	0.739745	-0.07909	17.50359	1.67E-38	4.58E-37	77.00456	up
NAKAYA_F	-0.73959	0.008351	-15.7151	7.41E-34	1.01E-32	66.34249	down
REACTOM	0.739279	-0.14678	11.15027	1.48E-21	5.98E-21	38.14207	up
GOBP_NEC	-0.73909	-0.0591	-18.1816	3.16E-40	1.18E-38	80.95507	down
REACTOM	-0.73858	-0.04026	-15.9092	2.28E-34	3.35E-33	67.51519	down
FINETTI_BF	-0.73851	-0.2453	-15.2776	1.06E-32	1.26E-31	63.68721	down
REACTOM	-0.73778	-0.12698	-18.1828	3.14E-40	1.17E-38	80.96197	down
MODULE_2	-0.73749	0.164075	-12.4925	3.37E-25	1.82E-24	46.4861	down
ZNF214_TA	-0.73737	-0.02706	-11.1316	1.66E-21	6.69E-21	38.02645	down
GOMF_ARA	-0.73685	0.168117	-13.3115	2.02E-27	1.34E-26	51.58032	down
REACTOM	-0.7368	0.110173	-12.7445	6.97E-26	4.02E-25	48.05509	down
GOMF_OX	-0.7364	-0.01014	-19.122	1.41E-42	8.19E-41	86.34323	down
GOCC_LSN	0.736358	0.221143	13.29394	2.25E-27	1.49E-26	51.47135	up
AMIT_SERU	-0.73616	0.067337	-18.8187	7.99E-42	4.01E-40	84.61734	down
GOMF_ACT	-0.73558	-0.04719	-17.9512	1.21E-39	4.03E-38	79.61843	down
GOBP_DOU	0.735538	-0.18649	12.36405	7.52E-25	3.96E-24	45.68631	up
GOCC_PRC	-0.73526	0.15795	-9.76516	7.64E-18	2.37E-17	29.64605	down
REACTOM	-0.73511	0.13939	-11.7286	4.00E-23	1.84E-22	41.73183	down
LE_SKI_TAF	-0.73493	0.079125	-13.2259	3.45E-27	2.24E-26	51.04887	down
GOBP_NEU	-0.73469	0.055053	-19.1065	1.54E-42	8.91E-41	86.25512	down
HOEK_MO	-0.73442	0.092808	-14.1912	8.53E-30	7.21E-29	57.02666	down
WHITEFOF	0.734239	-0.12267	15.09514	3.25E-32	3.64E-31	62.57448	up
GOBP_U4_	0.734227	0.211722	14.07372	1.77E-29	1.45E-28	56.3014	up
GCM_TPT1	0.734019	0.288181	13.0439	1.07E-26	6.71E-26	49.91752	up
DESCARTE	-0.73395	-0.06276	-15.0909	3.34E-32	3.74E-31	62.54883	down
HP_ANOTI	0.733634	-0.15807	14.13419	1.21E-29	1.01E-28	56.6747	up
REACTOM	0.733312	-0.10376	14.50585	1.22E-30	1.13E-29	58.96409	up
LIANG_SILI	0.733144	-0.14874	13.01834	1.26E-26	7.82E-26	49.75859	up
REACTOM	0.733084	-0.12327	14.15113	1.09E-29	9.12E-29	56.77927	up
REACTOM	-0.73292	0.090475	-13.4507	8.49E-28	5.85E-27	52.44422	down
GOMF_LEU	-0.73243	-0.0381	-15.2483	1.27E-32	1.49E-31	63.50874	down
GOCC_SM	0.731258	0.115846	18.58545	3.06E-41	1.37E-39	83.28205	up
GOBP_SUC	0.73069	0.05168	16.96927	3.94E-37	8.69E-36	73.85457	up
BIOCARTA	-0.73068	0.192916	-12.7743	5.78E-26	3.37E-25	48.24049	down
GOMF_OX	-0.73059	0.026772	-14.5282	1.06E-30	9.93E-30	59.10157	down
KEGG_VAL	-0.73055	0.114771	-13.6957	1.85E-28	1.37E-27	53.96361	down
LEE_SP4_TI	-0.73034	0.050945	-19.1822	1.00E-42	5.98E-41	86.68462	down
GOBP_REG	-0.73021	0.04524	-12.2084	1.99E-24	1.01E-23	44.71737	down
HP_ABNOI	-0.72995	0.094133	-15.8938	2.51E-34	3.66E-33	67.42239	down
SMID_BREA	0.729431	-0.12532	13.36047	1.49E-27	1.00E-26	51.88443	up
GOBP_PEP	-0.72901	0.086491	-13.4424	8.93E-28	6.14E-27	52.39318	down
REACTOM	-0.72888	-0.00706	-18.2388	2.27E-40	8.66E-39	81.28571	down
HP_PONTC	-0.72865	-0.03015	-16.196	4.04E-35	6.49E-34	69.24193	down
HP_ABNOI	-0.7286	0.17655	-12.2429	1.61E-24	8.21E-24	44.93204	down
GOBP_RES	-0.72849	-0.05059	-16.1411	5.62E-35	8.88E-34	68.91153	down
HP_PROLC	-0.72837	-0.03757	-16.7989	1.09E-36	2.26E-35	72.84387	down
ZHONG_PI	0.728083	-0.13592	13.8986	5.23E-29	4.10E-28	55.21922	up
GOBP_DNA	0.7277	-0.16081	12.69144	9.71E-26	5.54E-25	47.72463	up
SARTIPY_B	-0.72748	0.038156	-18.1215	4.48E-40	1.61E-38	80.60713	down
GOCC_BOX	0.72721	0.074109	11.05804	2.62E-21	1.04E-20	37.57118	up
BENPORAT	0.72679	-0.07313	18.17254	3.33E-40	1.24E-38	80.90245	up
GOBP_POS	-0.7267	-0.13325	-12.6946	9.52E-26	5.44E-25	47.74404	down
HP_ASTERI	-0.72651	-0.0273	-12.895	2.72E-26	1.64E-25	48.99167	down
RARB_TAR	-0.72587	0.178781	-8.41112	2.53E-14	6.22E-14	21.61203	down
HP_ABNOI	-0.72535	0.144952	-10.3035	2.81E-19	9.66E-19	32.92486	down

MIR8074	-0.72511	-0.04664	-18.955	3.66E-42	1.96E-40	85.39447	down
GOBP_NEC	-0.72455	-0.00034	-16.7091	1.86E-36	3.70E-35	72.30949	down
GOBP_SER	-0.72438	-0.02241	-21.075	2.64E-47	5.46E-45	97.18187	down
GOBP_SNC	0.724043	0.122274	12.32556	9.57E-25	5.00E-24	45.44664	up
GOBP_NEC	-0.72399	0.140002	-11.8267	2.17E-23	1.01E-22	42.34154	down
HP_VASCU	-0.72321	0.006923	-15.4901	2.91E-33	3.70E-32	64.97904	down
BIOCARTA	-0.72311	0.188922	-13.3538	1.55E-27	1.04E-26	51.84294	down
GOCC_GPI	0.722654	-0.099	13.76184	1.22E-28	9.24E-28	54.37297	up
CAR_IGFBF	-0.72264	0.204438	-12.5353	2.58E-25	1.41E-24	46.75254	down
ZHOU_CEL	0.722227	-0.11982	14.57664	7.88E-31	7.49E-30	59.39916	up
REACTOM	-0.72218	0.11657	-13.6038	3.27E-28	2.35E-27	53.39408	down
HP_BUDD_	-0.72189	-0.1356	-13.8374	7.65E-29	5.89E-28	54.84094	down
GOBP_POS	-0.72155	-0.21817	-12.1822	2.35E-24	1.18E-23	44.55419	down
HP_MALIG	0.72146	0.224237	15.35144	6.78E-33	8.23E-32	64.13628	up
LI_WILMS_	0.72142	-0.19307	13.76431	1.21E-28	9.11E-28	54.38831	up
REACTOM	-0.72142	0.191298	-10.5199	7.39E-20	2.64E-19	34.25251	down
REACTOM	-0.72109	-0.0398	-15.7237	7.03E-34	9.67E-33	66.39488	down
HP_MAND	0.720836	-0.02486	17.37479	3.56E-38	9.26E-37	76.24814	up
GOBP_ANT	-0.72071	-0.00511	-14.9187	9.60E-32	1.02E-30	61.49629	down
HP_BRANC	0.720507	-0.0978	13.84115	7.48E-29	5.76E-28	54.86385	up
GCM_ANP	0.720019	0.122419	17.19575	1.03E-37	2.47E-36	75.19359	up
GCM_NPM	0.719901	0.194644	15.59992	1.49E-33	1.96E-32	65.64511	up
HP_HIGH_I	-0.71989	0.028685	-14.2318	6.63E-30	5.67E-29	57.27677	down
BLANCO_M	0.719615	-0.14473	13.73279	1.47E-28	1.10E-27	54.19312	up
BIOCARTA	-0.71961	-0.05441	-16.2169	3.56E-35	5.78E-34	69.36739	down
REACTOM	0.719467	0.259059	14.05931	1.93E-29	1.58E-28	56.21242	up
KEGG_RIBC	0.719398	0.303537	12.79595	5.05E-26	2.96E-25	48.37509	up
GOBP_POS	-0.71929	-0.04954	-12.9843	1.56E-26	9.60E-26	49.54685	down
WP_DNA_I	0.719277	-0.10623	14.344	3.31E-30	2.93E-29	57.96819	up
GOBP_LEU	-0.71907	0.010997	-17.3994	3.08E-38	8.11E-37	76.39286	down
HP_HYPOT	-0.71902	0.144812	-12.5175	2.88E-25	1.57E-24	46.64192	down
GOBP_REG	-0.7178	0.109897	-9.97382	2.13E-18	6.88E-18	30.9125	down
GOBP_MEI	0.71775	-0.07898	13.42032	1.03E-27	7.00E-27	52.25596	up
ANDERSEN	0.717666	0.110141	17.15283	1.32E-37	3.13E-36	74.94026	up
GOBP_RIBC	0.71654	0.207634	15.15828	2.21E-32	2.52E-31	62.95976	up
ZHONG_PI	0.716465	-0.1289	14.54957	9.31E-31	8.76E-30	59.23281	up
GOMF_TRA	-0.71604	-0.02271	-20.2926	1.95E-45	2.15E-43	92.89759	down
GOBP_POS	-0.71598	0.023297	-13.8203	8.51E-29	6.53E-28	54.73498	down
GOBP_NEC	-0.71597	0.083965	-17.2439	7.72E-38	1.88E-36	75.47776	down
GOBP_GLY	-0.71586	0.165608	-10.9836	4.17E-21	1.63E-20	37.1106	down
GOBP_POS	-0.71581	-0.07707	-17.0731	2.12E-37	4.89E-36	74.46928	down
ZHONG_PI	0.715565	-0.09786	15.54564	2.08E-33	2.69E-32	65.31598	up
GOBP_SUC	-0.71549	0.026253	-11.4968	1.70E-22	7.42E-22	40.2912	down
GOMF_LIP	-0.7153	0.172588	-16.0858	7.85E-35	1.22E-33	68.57934	down
GOBP_NUC	0.715209	0.111351	16.44424	9.07E-36	1.62E-34	70.72923	up
WP_CATAL	-0.71514	0.038549	-12.0865	4.27E-24	2.11E-23	43.95818	down
GOMF_N_I	-0.71514	0.038549	-12.0865	4.27E-24	2.11E-23	43.95818	down
YANAGISA	0.715076	0.188852	16.0606	9.14E-35	1.41E-33	68.42766	up
GOBP_ALK	-0.71491	0.124553	-13.4298	9.67E-28	6.61E-27	52.3147	down
HP_INCREA	-0.71489	0.126111	-15.3335	7.57E-33	9.13E-32	64.02739	down
GOBP_RRN	0.714784	0.090088	13.7147	1.64E-28	1.22E-27	54.08108	up
WP_DISOR	-0.71473	0.075069	-11.7385	3.76E-23	1.73E-22	41.79315	down
HP_RESPIR	-0.71469	0.145168	-10.7277	2.04E-20	7.59E-20	35.53112	down
WP_MOLY	-0.7146	0.119171	-10.3031	2.82E-19	9.68E-19	32.92255	down
ZHONG_PI	0.714504	-0.08417	16.00872	1.25E-34	1.91E-33	68.11532	up
REN_BOUN	0.714478	-0.14386	13.89276	5.43E-29	4.25E-28	55.18312	up
GOBP_VEN	-0.71435	0.015374	-16.7977	1.09E-36	2.27E-35	72.83636	down
MODULE_2	-0.71429	0.047271	-17.3797	3.46E-38	9.04E-37	76.2771	down

GOBP_NEC	-0.71422	0.065983	-13.5646	4.17E-28	2.97E-27	53.15086	down
BIOCARTA	0.71422	-0.12567	12.74057	7.14E-26	4.12E-25	48.03043	up
GOMF_AC	-0.71412	0.131347	-12.6732	1.09E-25	6.18E-25	47.61098	down
ZNF211_T/	0.714091	-0.2676	9.832076	5.08E-18	1.60E-17	30.05152	up
GOBP_POS	0.713867	-0.05128	12.53906	2.52E-25	1.38E-24	46.77601	up
REACTOM	0.713538	-0.00479	19.40825	2.78E-43	1.87E-41	87.96198	up
GOBP_DET	-0.71332	-0.03867	-12.124	3.38E-24	1.68E-23	44.1916	down
GOBP_PYR	0.713273	-0.00054	13.62677	2.83E-28	2.05E-27	53.53631	up
BIOCARTA	0.713172	0.069562	14.22593	6.88E-30	5.87E-29	57.24064	up
GOMF_FIB	-0.71282	-0.09458	-13.3403	1.69E-27	1.13E-26	51.75899	down
GOBP_OVU	-0.71237	0.003439	-20.5554	4.56E-46	6.35E-44	94.34572	down
HP_BLEEDI	-0.71217	-0.03077	-13.9584	3.61E-29	2.88E-28	55.58886	down
GOCC_RA(	0.711618	0.162373	12.99299	1.47E-26	9.11E-26	49.60093	up
GOCC_U12	0.711215	0.077287	21.49361	2.72E-48	7.26E-46	99.44193	up
GOBP_LON	-0.71119	0.06586	-15.519	2.44E-33	3.13E-32	65.15441	down
HP_DISTAL	-0.71109	0.059306	-15.9056	2.33E-34	3.42E-33	67.49344	down
GOBP_NEC	-0.71081	0.139295	-11.8141	2.35E-23	1.09E-22	42.2632	down
GOMF_AR/	-0.71072	0.167333	-12.549	2.37E-25	1.30E-24	46.83817	down
REACTOM	-0.71066	-0.05232	-17.327	4.72E-38	1.19E-36	75.96709	down
GOBP_T_H	-0.71064	0.126596	-12.859	3.41E-26	2.03E-25	48.76745	down
GOBP_ALK	-0.71039	0.174804	-12.6588	1.19E-25	6.73E-25	47.52145	down
REACTOM	-0.71	-0.1523	-14.1699	9.73E-30	8.15E-29	56.89502	down
WP_MIR51	-0.7097	0.064996	-12.7832	5.47E-26	3.19E-25	48.29598	down
KEGG_PRC	-0.70963	0.123378	-13.8296	8.03E-29	6.17E-28	54.79232	down
REACTOM	0.709374	-0.01173	19.00837	2.70E-42	1.46E-40	85.69797	up
GOBP_TRY	-0.70928	0.137813	-13.3497	1.59E-27	1.07E-26	51.81732	down
GOCC_DN	0.709217	-0.18744	11.78192	2.87E-23	1.33E-22	42.0631	up
WP_METH	-0.70902	0.146237	-14.8385	1.57E-31	1.62E-30	61.00555	down
HP_FLAT_(	0.707857	-0.07169	19.75799	3.87E-44	3.20E-42	89.92577	up
CHNG_MU	0.707803	0.263625	14.96389	7.27E-32	7.83E-31	61.77264	up
HP_HYPER	-0.70765	0.024374	-18.2803	1.78E-40	6.88E-39	81.52567	down
GOCC_BR(	0.707413	-0.06262	15.65342	1.08E-33	1.44E-32	65.96927	up
KAPOSI_LI	-0.70732	0.107296	-9.6166	1.89E-17	5.70E-17	28.74814	down
WP_VALPR	-0.70649	0.14609	-12.662	1.17E-25	6.60E-25	47.54148	down
GOBP_CEL	-0.70565	-0.02593	-15.3154	8.46E-33	1.01E-31	63.9168	down
REACTOM	0.70565	-0.12728	12.76852	6.00E-26	3.49E-25	48.20438	up
GOBP_CHI	0.705508	-0.14092	14.33748	3.45E-30	3.04E-29	57.92801	up
MIR6090	-0.70551	-0.07478	-16.212	3.67E-35	5.93E-34	69.33742	down
GOBP_NEC	-0.70494	0.121417	-11.7808	2.89E-23	1.34E-22	42.05592	down
HP_HYPOL	-0.70494	0.155606	-12.8271	4.16E-26	2.46E-25	48.56897	down
WANG_ME	0.704521	-0.17155	15.47925	3.11E-33	3.94E-32	64.9131	up
MORF_NM	0.704425	0.210357	15.34006	7.27E-33	8.80E-32	64.06709	up
GOMF_KIN	0.704165	-0.19487	13.6661	2.22E-28	1.63E-27	53.78001	up
GOMF_CIL	-0.70408	-0.01263	-16.941	4.66E-37	1.02E-35	73.68689	down
CHIANG_L	-0.70367	0.047821	-17.8147	2.68E-39	8.44E-38	78.82409	down
GOBP_TRY	-0.70306	0.180377	-12.0315	6.02E-24	2.94E-23	43.61573	down
CHIANG_L	-0.7028	0.160088	-11.1077	1.93E-21	7.73E-21	37.87845	down
GOBP_SPL	0.702678	0.073164	20.12473	4.97E-45	5.11E-43	91.96851	up
GOMF_UBI	0.702032	0.19078	12.73142	7.56E-26	4.36E-25	47.97347	up
GNF2_MSI	0.701906	-0.18645	12.54079	2.49E-25	1.37E-24	46.78677	up
HOLLEMAI	0.701796	0.175147	14.91185	1.00E-31	1.06E-30	61.45431	up
GCM_PFN1	0.701096	0.160305	16.53273	5.34E-36	9.92E-35	71.25798	up
HP_POROI	0.701089	0.017993	12.52012	2.83E-25	1.55E-24	46.65807	up
GOBP_POS	0.700796	-0.16303	14.26542	5.39E-30	4.65E-29	57.48407	up
HP_HYPO(	-0.70075	0.083382	-16.6222	3.12E-36	6.01E-35	71.79181	down
REACTOM	0.700621	0.026895	19.70041	5.35E-44	4.30E-42	89.60354	up
GOBP_REG	-0.70056	-0.01754	-11.1812	1.22E-21	4.97E-21	38.33368	down
GOMF_BET	0.700405	-0.00042	9.079396	4.84E-16	1.33E-15	25.53191	up

GOBP_ASF	-0.70037	0.220911	-12.0503	5.36E-24	2.63E-23	43.73266	down
MODULE_!	0.700312	-0.08269	16.62557	3.06E-36	5.90E-35	71.81188	up
GOCC_AN.	0.700311	0.012469	20.74392	1.62E-46	2.58E-44	95.37865	up
NAGASHIN	-0.70026	0.06332	-16.2787	2.45E-35	4.09E-34	69.73799	down
BOYAULT_	0.70019	-0.16757	14.73176	3.03E-31	3.03E-30	60.35121	up
REACTOM	-0.70001	-0.06132	-12.8937	2.74E-26	1.65E-25	48.98345	down
GOBP_NE(	-0.69972	-0.09712	-18.2176	2.57E-40	9.74E-39	81.16297	down
MATZUK_(	-0.69963	-0.00375	-19.9759	1.14E-44	1.08E-42	91.14133	down
GOBP_RES	-0.6996	-0.03303	-17.3954	3.16E-38	8.29E-37	76.36925	down
REACTOM	-0.69927	0.066247	-11.2035	1.06E-21	4.34E-21	38.47161	down
BIOCARTA	-0.69889	0.036427	-15.8126	4.10E-34	5.82E-33	66.93234	down
WP_CELLS_	-0.69889	0.036427	-15.8126	4.10E-34	5.82E-33	66.93234	down
GOBP_RES	-0.69884	0.008396	-17.6708	6.24E-39	1.84E-37	77.98368	down
GOBP_PO!	-0.69876	0.06057	-15.4785	3.13E-33	3.95E-32	64.90841	down
WP_TAMC	-0.69866	0.117769	-13.5735	3.95E-28	2.81E-27	53.20587	down
REACTOM	-0.69861	0.191615	-10.8975	7.12E-21	2.73E-20	36.57851	down
HP_LOWEI	0.69854	-0.00813	15.80781	4.22E-34	5.98E-33	66.90324	up
BIOCARTA	-0.69852	-0.06429	-15.7644	5.49E-34	7.66E-33	66.64089	down
GOBP_REG	0.698436	-0.01502	12.42558	5.12E-25	2.73E-24	46.06947	up
GOBP_CH(	-0.6978	0.160964	-10.3705	1.86E-19	6.47E-19	33.33558	down
REACTOM	-0.6978	0.160964	-10.3705	1.86E-19	6.47E-19	33.33558	down
MODY_HIF	0.69778	0.123423	19.95753	1.26E-44	1.18E-42	91.03934	up
GOBP_PO!	-0.6977	0.016285	-8.59645	8.53E-15	2.16E-14	22.68884	down
MONTERC	-0.69768	0.078038	-15.9796	1.49E-34	2.24E-33	67.93988	down
chr6q26	-0.69745	0.064963	-13.1581	5.26E-27	3.37E-26	50.62714	down
GOBP_PRC	0.697319	-0.00693	12.57651	1.99E-25	1.10E-24	47.0092	up
GLINSKY_(	0.697291	-0.23097	12.86214	3.34E-26	1.99E-25	48.78694	up
HP_HYPO(	0.697265	-0.10636	13.95869	3.60E-29	2.87E-28	55.59074	up
GOBP_C_T	-0.69724	0.059452	-11.8197	2.27E-23	1.06E-22	42.29794	down
WP_AMIN(	-0.69718	0.217123	-11.3622	3.94E-22	1.67E-21	39.45563	down
GOCC_WN	-0.69718	-0.11386	-17.6186	8.48E-39	2.44E-37	77.67844	down
chr4q32	-0.69718	0.013205	-19.4061	2.81E-43	1.89E-41	87.94988	down
HP_LOW_F	-0.69713	0.211949	-9.97556	2.11E-18	6.81E-18	30.92305	down
MODULE_:	-0.69711	0.099228	-15.8878	2.60E-34	3.79E-33	67.38634	down
BIOCARTA	-0.69698	-0.04063	-23.6598	3.05E-53	3.78E-50	110.7786	down
GOBP_OX\	-0.69675	0.002302	-15.8379	3.52E-34	5.04E-33	67.08499	down
GOBP_REG	-0.69659	0.013523	-16.501	6.45E-36	1.18E-34	71.06827	down
HP_INCRE/	-0.69644	-0.10706	-14.2742	5.10E-30	4.41E-29	57.53845	down
REACTOM	-0.69641	0.227497	-9.75509	8.13E-18	2.52E-17	29.58504	down
GOBP_REG	-0.69626	0.044617	-17.6301	7.93E-39	2.29E-37	77.74561	down
GOBP_NE(	-0.69608	0.149198	-10.4786	9.55E-20	3.38E-19	33.9984	down
KAMMING	0.696005	-0.1808	13.08487	8.31E-27	5.24E-26	50.17227	up
GOBP_PRC	0.695555	-0.02947	13.78079	1.09E-28	8.26E-28	54.4903	up
REACTOM	0.695514	-0.14352	13.43842	9.16E-28	6.29E-27	52.36826	up
GOBP_REG	-0.6953	0.138138	-8.87519	1.64E-15	4.35E-15	24.3236	down
ALONSO_I	-0.69506	0.069374	-14.7231	3.20E-31	3.18E-30	60.29828	down
REACTOM	-0.69498	0.244252	-10.0192	1.62E-18	5.25E-18	31.18859	down
GOBP_REG	0.694614	-0.02	15.82723	3.75E-34	5.37E-33	67.02055	up
HP_INCRE/	-0.69423	0.026899	-19.0183	2.55E-42	1.39E-40	85.75455	down
NIKOLSKY_	-0.6942	-0.02197	-13.9053	5.02E-29	3.95E-28	55.26038	down
MORI_IMN	0.694197	-0.12428	14.74438	2.80E-31	2.82E-30	60.42858	up
GOBP_EST.	0.694176	-0.09618	14.24839	5.99E-30	5.14E-29	57.37911	up
GOBP_NE(	0.694004	0.108007	15.71285	7.51E-34	1.03E-32	66.32906	up
LET_7D_3P	-0.69386	-0.02574	-18.743	1.23E-41	5.88E-40	84.18462	down
GOBP_COI	-0.69371	0.160031	-14.0384	2.20E-29	1.78E-28	56.08356	down
FAN_EMBF	-0.69338	0.182287	-10.7995	1.31E-20	4.93E-20	35.97368	down
REACTOM	-0.69314	-0.00189	-16.8212	9.51E-37	1.99E-35	72.97637	down
REACTOM	0.692887	-0.00112	13.54006	4.86E-28	3.44E-27	52.99874	up

GOBP_POS	0.692627	-0.03566	11.70581	4.61E-23	2.11E-22	41.58986	up
GOBP_NEG	-0.69243	0.084954	-12.0602	5.03E-24	2.47E-23	43.79455	down
GNF2_RAN	0.692382	0.036228	16.32501	1.86E-35	3.17E-34	70.01549	up
GOBP_ENZ	0.692344	0.109257	11.21133	1.01E-21	4.14E-21	38.52031	up
GSE15750_	0.692106	-0.09754	15.89285	2.52E-34	3.68E-33	67.41677	up
GOBP_LYS	0.692063	0.061798	14.21632	7.30E-30	6.22E-29	57.18137	up
HP_HYPOF	0.691945	0.054935	17.7521	3.87E-39	1.18E-37	78.45868	up
WP_KETOG	-0.69178	0.13594	-15.2831	1.03E-32	1.22E-31	63.72016	down
GOBP_SMO	-0.69171	0.116328	-10.6424	3.46E-20	1.26E-19	35.00578	down
HP_VISCEF	-0.69167	-0.00138	-17.2809	6.21E-38	1.54E-36	75.69535	down
REACTOM	-0.69125	-0.04244	-17.5751	1.09E-38	3.09E-37	77.42359	down
GOBP_PLA	-0.69097	0.023814	-12.619	1.53E-25	8.54E-25	47.27378	down
GCM_PSM	0.690752	0.118739	17.64691	7.18E-39	2.09E-37	77.84405	up
GOBP_RES	-0.69066	0.056583	-13.4288	9.73E-28	6.65E-27	52.30828	down
REACTOM	0.690367	0.296506	12.2649	1.40E-24	7.18E-24	45.06895	up
HP_GERMI	-0.68994	-0.03806	-17.4895	1.81E-38	4.94E-37	76.92195	down
GNF2_ST13	0.689827	0.220306	12.10945	3.70E-24	1.83E-23	44.10118	up
GOCC_RIB	0.689785	0.165322	15.25106	1.25E-32	1.47E-31	63.52527	up
MODULE_1	-0.68976	0.131215	-13.4308	9.61E-28	6.58E-27	52.32069	down
MINGUEZ_	0.689683	-0.06298	17.21683	9.06E-38	2.20E-36	75.31795	up
GOMF_5_3	0.689517	-0.0547	14.87168	1.28E-31	1.34E-30	61.2085	up
REACTOM	0.689446	0.063257	14.99076	6.17E-32	6.69E-31	61.9369	up
HP_UPPER_	-0.68924	-0.01904	-13.3118	2.02E-27	1.34E-26	51.58223	down
GOMF_ALC	-0.68881	0.175909	-10.6491	3.32E-20	1.21E-19	35.04698	down
MORF_ESP	0.688762	-0.06299	17.72193	4.62E-39	1.39E-37	78.28248	up
REACTOM	0.688592	-0.16131	13.85263	6.96E-29	5.39E-28	54.93487	up
REACTOM	0.688351	0.048508	19.46912	1.97E-43	1.38E-41	88.30487	up
HP_MASTC	-0.68832	0.049699	-13.2307	3.34E-27	2.18E-26	51.07847	down
HP_CENTR	-0.68829	0.161858	-9.37667	8.09E-17	2.34E-16	27.30545	down
GOBP_END	-0.68828	0.001876	-13.9649	3.47E-29	2.77E-28	55.62932	down
GOBP_EPIT	-0.68811	0.105987	-15.3238	8.03E-33	9.63E-32	63.96802	down
GOMF_VAS	-0.68766	-0.07764	-10.4474	1.16E-19	4.08E-19	33.80694	down
GOMF_MY	-0.68749	0.079451	-9.91061	3.14E-18	1.00E-17	30.52819	down
GOBP_SCA	0.687113	-0.01769	10.1901	5.66E-19	1.90E-18	32.23119	up
HARALAM	-0.68699	0.020444	-15.1886	1.83E-32	2.11E-31	63.14493	down
REACTOM	-0.68691	0.210952	-13.1443	5.74E-27	3.66E-26	50.54141	down
REICHERT_	0.686852	-0.21964	12.1842	2.32E-24	1.17E-23	44.56653	up
GOCC_PRE	0.686734	0.071576	18.04693	6.92E-40	2.40E-38	80.1745	up
GOCC_DEU	0.68635	-0.2537	10.0963	1.01E-18	3.32E-18	31.65856	up
DACOSTA_	-0.6861	-0.02893	-22.8069	2.53E-51	1.60E-48	106.3866	down
chr2q22	-0.68609	0.031782	-15.6549	1.07E-33	1.43E-32	65.97844	down
WP_BONE_	-0.68604	-0.091	-20.4366	8.80E-46	1.08E-43	93.69212	down
BIOCARTA	0.685957	-0.16373	12.27419	1.32E-24	6.80E-24	45.12682	up
HP_PREMA	-0.68593	0.085457	-13.2407	3.14E-27	2.05E-26	51.14082	down
GOBP_REG	0.685872	-0.03116	10.77647	1.51E-20	5.67E-20	35.83175	up
GOMF_DN	0.685788	-0.13115	17.66226	6.56E-39	1.92E-37	77.93379	up
GNF2_MSH	0.685589	-0.09629	14.104	1.46E-29	1.21E-28	56.48839	up
HP_BILATE	-0.68505	-0.09579	-14.8497	1.47E-31	1.52E-30	61.0739	down
MORF_FEN	0.684547	-0.00571	18.41113	8.36E-41	3.49E-39	82.27996	up
chr1p12	-0.6845	-0.00467	-15.0873	3.41E-32	3.82E-31	62.52636	down
GOMF_OX	-0.68444	0.18652	-10.7413	1.88E-20	7.00E-20	35.61481	down
GARGALO	0.684434	-0.06598	20.69755	2.09E-46	3.23E-44	95.12496	up
GOBP_DEC	0.684338	0.071821	13.66774	2.20E-28	1.61E-27	53.7902	up
GOMF_UBI	-0.68429	0.178786	-10.8061	1.26E-20	4.74E-20	36.0147	down
GOBP_KET	-0.68404	0.158351	-12.973	1.67E-26	1.03E-25	49.47644	down
WP_NEURO	-0.68388	0.018093	-16.2791	2.45E-35	4.08E-34	69.74022	down
GNF2_PA2	0.68358	-0.00428	16.46427	8.04E-36	1.45E-34	70.849	up
KEGG_LIM	-0.68354	0.157437	-11.0064	3.62E-21	1.42E-20	37.25164	down

HP_PAPILL	-0.68331	-0.2056	-12.313	1.04E-24	5.40E-24	45.36823	down
MIR6741_3	-0.68324	0.004065	-12.1424	3.01E-24	1.50E-23	44.3062	down
WP_COMF	-0.68307	0.208059	-12.8314	4.05E-26	2.40E-25	48.5954	down
GOBP_PRC	0.681968	0.118866	15.88404	2.66E-34	3.86E-33	67.3636	up
GCM_CSN	0.681902	0.12078	16.58092	4.00E-36	7.59E-35	71.54562	up
GOBP_NEC	-0.68169	0.111009	-11.9247	1.17E-23	5.62E-23	42.95147	down
GOCC_CH/	0.681433	0.005785	13.94673	3.88E-29	3.09E-28	55.51683	up
GOMF_C_/	-0.68139	0.218687	-9.68786	1.22E-17	3.74E-17	29.17846	down
GOBP_SCH	-0.68128	-0.06832	-10.7938	1.36E-20	5.10E-20	35.93862	down
GOBP_FAT	-0.68123	0.116276	-14.445	1.78E-30	1.62E-29	58.58975	down
GOCC_CTI	0.681064	-0.13143	12.17387	2.47E-24	1.24E-23	44.50223	up
HOLLEMAI	0.681053	0.181022	14.35189	3.16E-30	2.80E-29	58.01681	up
MODULE_2	-0.68061	0.119434	-14.2544	5.77E-30	4.97E-29	57.416	down
REACTOM	-0.68052	0.143553	-13.5296	5.19E-28	3.66E-27	52.93363	down
MIR3683	-0.68048	0.111888	-15.9215	2.12E-34	3.13E-33	67.58962	down
MODULE_3	-0.68021	0.133651	-16.3004	2.15E-35	3.62E-34	69.86781	down
MIR6506_3	-0.6799	-0.00366	-12.0572	5.13E-24	2.52E-23	43.77585	down
GNF2_MCI	0.67978	-0.10874	14.55857	8.81E-31	8.32E-30	59.28814	up
GOBP_OPS	-0.6797	0.100247	-14.4568	1.65E-30	1.51E-29	58.66243	down
GOCC_SN(	0.679451	0.147421	15.0954	3.25E-32	3.64E-31	62.57605	up
AMIT_SERU	-0.6793	-0.00347	-22.0013	1.78E-49	6.33E-47	102.1528	down
GOBP_REC	-0.67927	0.055252	-11.6451	6.74E-23	3.04E-22	41.21263	down
GOBP_REG	-0.67911	0.003717	-14.9122	9.99E-32	1.06E-30	61.45618	down
GOBP_EMI	-0.67909	-0.10963	-16.1951	4.06E-35	6.53E-34	69.23595	down
GOBP_PEP	0.679033	0.052071	14.09414	1.56E-29	1.28E-28	56.42748	up
GNF2_G22	0.678841	0.042447	17.74959	3.93E-39	1.19E-37	78.44399	up
GOBP_HIS	-0.67839	0.188997	-12.213	1.94E-24	9.81E-24	44.74556	down
KAUFFMAI	0.678183	-0.12368	14.02946	2.32E-29	1.88E-28	56.02808	up
GOCC_MIT	0.678079	0.274169	11.03351	3.06E-21	1.21E-20	37.41943	up
REACTOM	-0.67801	0.081192	-12.0023	7.23E-24	3.51E-23	43.43416	down
GOMF_RN	-0.67794	0.000628	-16.2249	3.39E-35	5.52E-34	69.41484	down
GOBP_ATF	-0.6779	0.117659	-16.1719	4.67E-35	7.45E-34	69.09659	down
chr18q22	-0.6779	0.026324	-17.2721	6.54E-38	1.62E-36	75.64375	down
chr13q31	-0.67783	0.02557	-15.087	3.42E-32	3.82E-31	62.52493	down
REACTOM	-0.67722	-0.03318	-23.8634	1.08E-53	1.77E-50	111.8136	down
RAMJAUN	-0.67714	-0.01923	-18.242	2.23E-40	8.52E-39	81.30398	down
REACTOM	-0.67681	-0.10113	-14.6163	6.17E-31	5.95E-30	59.64251	down
PLASARI_T	-0.67677	0.047626	-15.4062	4.86E-33	6.01E-32	64.46915	down
MARIADAS	-0.67658	-0.04953	-13.3758	1.35E-27	9.15E-27	51.97988	down
AMIT_EGF_	-0.6762	0.04645	-18.6007	2.80E-41	1.26E-39	83.36973	down
MODULE_3	0.675917	-0.20778	12.7842	5.44E-26	3.18E-25	48.30195	up
LE_NEURO	0.67578	-0.09595	16.77914	1.22E-36	2.51E-35	72.72616	up
GOBP_EST	-0.67577	-0.03393	-16.2395	3.11E-35	5.09E-34	69.50264	down
MODULE_3	0.675753	-0.12366	13.33784	1.71E-27	1.15E-26	51.74397	up
SONG_TAF	0.675408	-0.12942	13.41002	1.09E-27	7.46E-27	52.19201	up
GSE18893_	0.675317	-0.00118	20.90592	6.65E-47	1.23E-44	96.26275	up
GOBP_NEC	-0.67519	0.03722	-10.4289	1.30E-19	4.56E-19	33.69322	down
GOBP_REG	-0.67501	0.050496	-14.3002	4.34E-30	3.78E-29	57.69849	down
FUJIWARA	-0.67469	0.167301	-12.5381	2.53E-25	1.39E-24	46.77004	down
WP_NAD_I	-0.67454	-0.09171	-16.7805	1.21E-36	2.50E-35	72.73416	down
HP_NAEVL	0.674346	-0.04902	13.4124	1.08E-27	7.35E-27	52.2068	up
GOBP_XEN	-0.67428	0.049465	-10.2911	3.04E-19	1.04E-18	32.84898	down
GOCC_EUI	0.674012	0.109442	10.85317	9.38E-21	3.57E-20	36.30503	up
REACTOM	0.673882	0.097214	10.14704	7.37E-19	2.45E-18	31.96818	up
REACTOM	0.673861	-0.08634	11.55884	1.16E-22	5.10E-22	40.6765	up
MYLLYKAN	-0.67357	0.038187	-9.9347	2.71E-18	8.68E-18	30.67463	down
MT	-0.67353	0.208475	-8.3524	3.57E-14	8.67E-14	21.27263	down
GOBP_NEC	0.673438	-0.06301	11.64367	6.80E-23	3.07E-22	41.20355	up

HP_DECRE	-0.67342	0.07459	-11.4935	1.74E-22	7.57E-22	40.27088	down
GOBP_TEL	0.673344	0.063262	12.26431	1.40E-24	7.21E-24	45.06531	up
GOBP_RES	-0.67331	0.078375	-12.2123	1.94E-24	9.85E-24	44.74144	down
BIOCARTA	0.67331	-0.06046	15.09444	3.27E-32	3.66E-31	62.57025	up
GOCC_SM	0.673091	0.037746	20.50167	6.14E-46	8.19E-44	94.05028	up
MODULE_	0.672692	-0.13673	13.71988	1.59E-28	1.19E-27	54.11318	up
GOBP_FAT	-0.67231	0.11773	-11.7354	3.84E-23	1.77E-22	41.77351	down
GOBP_REG	0.672196	-0.08099	10.70697	2.32E-20	8.59E-20	35.40335	up
GOBP_REG	-0.67218	0.092021	-14.9594	7.48E-32	8.03E-31	61.7454	down
REACTOM	0.672023	0.001751	20.22473	2.85E-45	3.04E-43	92.52253	up
GOBP_BRA	-0.6719	0.103891	-12.5263	2.73E-25	1.49E-24	46.69666	down
GOBP_LEU	-0.67169	0.202649	-9.08649	4.64E-16	1.27E-15	25.57403	down
GOMF_LEU	-0.67169	0.202649	-9.08649	4.64E-16	1.27E-15	25.57403	down
HP_ABNOI	0.671646	0.031132	13.47913	7.11E-28	4.93E-27	52.62088	up
DESCARTE	-0.67157	0.021171	-17.2871	5.98E-38	1.49E-36	75.73238	down
HP_FINGEI	0.671268	-0.05112	12.28561	1.23E-24	6.35E-24	45.1979	up
REACTOM	-0.67126	0.067161	-15.3356	7.47E-33	9.02E-32	64.03985	down
REACTOM	0.671222	0.062923	12.33741	8.89E-25	4.66E-24	45.52046	up
GOBP_VAS	-0.6712	-0.04883	-13.2113	3.77E-27	2.45E-26	50.95808	down
GOMF_5_D	0.67075	-0.01183	11.5982	9.04E-23	4.03E-22	40.92099	up
HP_IRIS_AT	-0.67074	0.020207	-14.5036	1.24E-30	1.15E-29	58.95058	down
GOBP_DEN	-0.67049	0.090309	-11.5696	1.08E-22	4.78E-22	40.74351	down
GOBP_REG	-0.67048	-0.09482	-12.4492	4.42E-25	2.37E-24	46.21647	down
REACTOM	-0.67002	0.156747	-12.2015	2.08E-24	1.05E-23	44.67416	down
GOBP_FEM	0.669961	-0.07511	14.6175	6.12E-31	5.91E-30	59.65006	up
GOBP_LEY	-0.66966	-0.01264	-17.39	3.26E-38	8.54E-37	76.33768	down
HP_RIGHT_	-0.66958	0.009011	-21.1829	1.47E-47	3.22E-45	97.76659	down
GOMF_OX	-0.66943	0.077894	-11.591	9.46E-23	4.21E-22	40.8762	down
GOCC_U5_	0.669434	0.086776	12.6452	1.30E-25	7.31E-25	47.43678	up
WINNEPEI	0.669317	-0.09812	16.45653	8.43E-36	1.52E-34	70.80275	up
AIZARANI_	-0.66873	0.193597	-11.827	2.16E-23	1.01E-22	42.34328	down
MODULE_	0.668406	-0.1312	10.74922	1.79E-20	6.67E-20	35.66373	up
REACTOM	0.668265	-0.16805	11.8653	1.70E-23	8.04E-23	42.58172	up
MODULE_	0.66798	0.135968	7.131534	3.58E-11	7.26E-11	14.44797	up
GOCC_BO	0.666826	0.073161	10.00504	1.76E-18	5.71E-18	31.10248	up
GOCC_BO	0.666826	0.073161	10.00504	1.76E-18	5.71E-18	31.10248	up
GOMF_BO	0.666826	0.073161	10.00504	1.76E-18	5.71E-18	31.10248	up
HP_PULM	-0.66641	0.062158	-11.1548	1.44E-21	5.82E-21	38.16986	down
REACTOM	0.666249	-0.15189	12.38978	6.40E-25	3.39E-24	45.84654	up
GOBP_ME	-0.6662	0.000894	-15.0789	3.59E-32	4.00E-31	62.47521	down
MODULE_	-0.66609	0.173859	-11.9579	9.54E-24	4.59E-23	43.15794	down
POMEROY	0.665586	0.107736	18.18933	3.02E-40	1.14E-38	80.99962	up
MODULE_	-0.66556	0.180985	-11.589	9.58E-23	4.26E-22	40.86373	down
HP_BICARI	-0.66531	0.093107	-10.6081	4.29E-20	1.55E-19	34.79443	down
GOBP_CO	0.664957	-0.06365	12.7498	6.74E-26	3.90E-25	48.08786	up
MODULE_	0.664921	-0.08965	10.18467	5.85E-19	1.96E-18	32.19803	up
GOBP_PO	-0.66492	0.047365	-12.968	1.72E-26	1.06E-25	49.44541	down
PATIL_LIVE	0.664583	-0.12029	21.62087	1.37E-48	3.92E-46	100.1245	up
GOCC_POI	0.664531	0.185945	13.30324	2.13E-27	1.41E-26	51.52908	up
GOMF_PYI	-0.66416	0.036326	-11.6618	6.08E-23	2.75E-22	41.3161	down
KIM_GERM	-0.66412	0.020384	-15.047	4.37E-32	4.80E-31	62.28054	down
GOBP_ETH	-0.66397	0.157421	-11.1605	1.39E-21	5.62E-21	38.2054	down
REACTOM	-0.66397	0.157421	-11.1605	1.39E-21	5.62E-21	38.2054	down
HP_RENAL	0.663743	-0.04917	15.63715	1.19E-33	1.58E-32	65.87071	up
GOBP_END	-0.66372	-0.07458	-15.4208	4.44E-33	5.52E-32	64.55792	down
HP_ELEVA	0.663575	0.318136	12.21896	1.86E-24	9.48E-24	44.78291	up
GOBP_DE_	0.663565	-0.20453	10.7914	1.38E-20	5.18E-20	35.92387	up
GOMF_HY	-0.66354	0.086031	-13.595	3.45E-28	2.48E-27	53.33937	down

GOBP_POS	-0.66342	-0.08457	-12.3046	1.09E-24	5.67E-24	45.31633	down
REACTOM	0.663264	-0.03836	14.26062	5.55E-30	4.78E-29	57.45447	up
HP_CEREBI	-0.66324	0.131523	-10.9467	5.24E-21	2.03E-20	36.88289	down
HP_FULMII	-0.66321	0.002856	-12.304	1.10E-24	5.69E-24	45.31261	down
GOBP_LOC	-0.6631	0.000692	-18.1916	2.98E-40	1.12E-38	81.01297	down
WU_APOP	0.663089	-0.17811	13.17781	4.65E-27	2.99E-26	50.74991	up
GOBP_POS	-0.66306	-0.0003	-13.6479	2.49E-28	1.81E-27	53.66753	down
chr4q26	-0.66289	0.008894	-17.8935	1.69E-39	5.52E-38	79.28295	down
WP_CALOI	-0.66272	-0.02322	-14.3083	4.13E-30	3.61E-29	57.74809	down
HP_LEBER_	-0.66268	0.165167	-8.3551	3.51E-14	8.54E-14	21.28821	down
HP_ATROF	-0.66255	0.092401	-12.9712	1.69E-26	1.04E-25	49.46537	down
GARGALO'	-0.66246	-0.15155	-15.6476	1.12E-33	1.49E-32	65.93404	down
HP_EPISOI	-0.66198	0.093516	-14.7079	3.51E-31	3.48E-30	60.20501	down
RHODES_C	0.661945	-0.04005	17.86883	1.96E-39	6.33E-38	79.13921	up
GOBP_PEP	-0.66181	-0.04196	-13.7929	1.01E-28	7.68E-28	54.56522	down
GOBP_NEC	-0.6618	0.035251	-16.6676	2.38E-36	4.65E-35	72.06243	down
GOBP_ALA	-0.66144	0.222172	-9.15804	3.02E-16	8.40E-16	25.99949	down
GNF2_IGF1	-0.66138	0.251296	-11.4664	2.06E-22	8.92E-22	40.10215	down
BONOME_	-0.66118	-0.07835	-18.5498	3.75E-41	1.65E-39	83.07765	down
GOBP_LUN	-0.66116	0.066071	-11.5265	1.41E-22	6.21E-22	40.47548	down
REACTOM	-0.66101	0.151327	-12.3951	6.20E-25	3.28E-24	45.87967	down
GNF2_NPN	0.660683	0.04576	14.74549	2.78E-31	2.80E-30	60.43542	up
REACTOM	-0.66004	-0.04486	-14.3635	2.94E-30	2.62E-29	58.08853	down
MODULE_:	-0.6599	0.144346	-10.5504	6.13E-20	2.20E-19	34.43954	down
GOBP_POS	-0.65988	-0.04188	-13.8895	5.54E-29	4.33E-28	55.16309	down
GOBP_NEC	-0.65959	0.021734	-19.0097	2.68E-42	1.46E-40	85.70572	down
GOMF_UBI	0.659423	0.034471	17.17065	1.19E-37	2.84E-36	75.0455	up
GOBP_DET	-0.65941	0.176456	-11.3304	4.81E-22	2.02E-21	39.2586	down
LEE_EARLY	0.659283	-0.12093	14.3341	3.52E-30	3.10E-29	57.9072	up
GOBP_CYS	-0.65918	0.151696	-9.12593	3.66E-16	1.01E-15	25.80846	down
GOBP_TEL	0.659142	-0.02885	12.59922	1.73E-25	9.61E-25	47.15056	up
REACTOM	-0.65897	0.270467	-11.4535	2.23E-22	9.64E-22	40.02206	down
GOBP_MU	0.658931	-0.15445	14.05065	2.04E-29	1.66E-28	56.15893	up
GOBP_CAF	-0.65893	-0.01135	-12.5638	2.16E-25	1.19E-24	46.93008	down
GOBP_CO	0.658816	0.272712	12.50716	3.07E-25	1.67E-24	46.57739	up
GSE15750_	0.658697	-0.12589	14.63181	5.61E-31	5.43E-30	59.73798	up
HP_LIMB_F	0.658021	0.159101	11.77457	3.00E-23	1.39E-22	42.0174	up
BIOCARTA	-0.65796	0.087811	-13.7904	1.02E-28	7.80E-28	54.54993	down
WP_CYTOI	0.657947	0.297357	11.93089	1.13E-23	5.41E-23	42.98982	up
GOBP_AM	-0.65781	-0.16264	-14.2495	5.94E-30	5.11E-29	57.38616	down
REACTOM	0.657783	0.232422	13.72067	1.58E-28	1.18E-27	54.11803	up
FARMER_B	-0.65756	0.003378	-16.6403	2.80E-36	5.43E-35	71.89967	down
GOCC_CYT	0.657553	0.302742	11.75275	3.44E-23	1.59E-22	41.8817	up
GCM_APEX	0.657482	0.081246	16.48078	7.28E-36	1.33E-34	70.9477	up
GOMF_FU(	-0.65738	0.19192	-11.8554	1.81E-23	8.53E-23	42.52019	down
GOCC_SPE	-0.65711	-0.18543	-7.71815	1.37E-12	3.02E-12	17.66787	down
WP_COVIL	-0.65708	0.179616	-11.058	2.62E-21	1.04E-20	37.57092	down
BOYAULT_	-0.65705	0.160188	-11.9478	1.02E-23	4.88E-23	43.0952	down
HP_HEMO	-0.65693	0.093053	-13.5738	3.94E-28	2.81E-27	53.20793	down
REACTOM	0.656782	-0.16209	11.44508	2.35E-22	1.01E-21	39.97003	up
GOBP_PAF	-0.65665	-0.08085	-12.0557	5.18E-24	2.54E-23	43.76655	down
GOMF_1_F	-0.65645	-0.14143	-18.8875	5.39E-42	2.77E-40	85.01	down
AMIT_EGF_	-0.65643	-0.04375	-18.7833	9.80E-42	4.80E-40	84.41491	down
GOMF_PRI	-0.65642	0.059438	-12.7581	6.40E-26	3.72E-25	48.13973	down
GOMF_PR(	-0.65632	-0.03829	-9.00167	7.70E-16	2.08E-15	25.07097	down
HP_KETOT	-0.65611	0.108734	-12.394	6.24E-25	3.30E-24	45.87275	down
HP_ABNOI	0.655303	0.110335	12.25147	1.52E-24	7.79E-24	44.98536	up
GOBP_REP	0.655105	-0.12367	13.11157	7.03E-27	4.46E-26	50.33819	up

ZHANG_RE	-0.6549	-0.00137	-16.6497	2.65E-36	5.15E-35	71.95579	down
GOBP_VEN	-0.65465	-0.00428	-15.1803	1.93E-32	2.21E-31	63.09388	down
MUNSHI_M	0.65463	-0.00511	17.77095	3.47E-39	1.06E-37	78.56865	up
MODULE_1	0.654628	-0.18235	10.46051	1.07E-19	3.77E-19	33.88746	up
REACTOM	0.654575	0.023655	18.30768	1.52E-40	5.94E-39	81.68348	up
CAR_HPX	-0.65419	0.219675	-11.112	1.88E-21	7.53E-21	37.90487	down
HP_DECRE	-0.65387	0.114119	-8.67601	5.34E-15	1.37E-14	23.15364	down
NIKOLSKY_	0.653628	0.141864	10.13965	7.71E-19	2.56E-18	31.92307	up
GOBP_NU(	0.65338	0.202851	14.07191	1.79E-29	1.46E-28	56.29022	up
GOMF_DN	0.653359	-0.26245	10.76179	1.65E-20	6.19E-20	35.74126	up
GOCC_MC	0.653175	-0.21042	10.73598	1.94E-20	7.22E-20	35.58214	up
HP_PROLC	-0.65314	0.065974	-14.6835	4.08E-31	4.02E-30	60.05507	down
GOBP_REG	0.653108	-0.02908	12.55029	2.35E-25	1.29E-24	46.8459	up
WP_SARSC	-0.65274	0.085458	-15.1303	2.62E-32	2.97E-31	62.78885	down
GUTIERREZ	-0.65271	-0.00692	-14.7673	2.44E-31	2.46E-30	60.56892	down
KEGG_FAT	-0.65266	0.152677	-11.9844	8.09E-24	3.92E-23	43.32257	down
REACTOM	-0.65253	0.121564	-13.7393	1.41E-28	1.06E-27	54.2336	down
HP_DECRE	0.652463	0.113538	10.63078	3.72E-20	1.36E-19	34.93415	up
GOBP_KIN	0.652449	-0.19366	13.69699	1.83E-28	1.36E-27	53.97139	up
GOBP_REG	0.652417	-0.13129	12.03133	6.03E-24	2.94E-23	43.61486	up
GOBP_DNA	0.652071	-0.15516	14.6387	5.37E-31	5.21E-30	59.78025	up
GOMF_CR(	0.651906	-0.11966	12.39327	6.27E-25	3.32E-24	45.86824	up
GOMF_SM	0.651886	0.109047	12.57959	1.95E-25	1.08E-24	47.02835	up
GOBP_REG	-0.65188	0.114304	-13.3706	1.40E-27	9.44E-27	51.94709	down
SOUCEK_M	-0.65154	0.128424	-12.8607	3.37E-26	2.01E-25	48.77795	down
GOBP_REG	-0.65084	-0.05663	-14.4174	2.11E-30	1.91E-29	58.41986	down
MODULE_1	-0.65084	0.133056	-13.2371	3.21E-27	2.10E-26	51.11835	down
REACTOM	0.6508	0.072793	17.01116	3.07E-37	6.88E-36	74.10263	up
GOBP_PAR	-0.6504	0.061498	-17.3114	5.18E-38	1.30E-36	75.87502	down
REACTOM	-0.65027	0.197077	-9.59586	2.14E-17	6.43E-17	28.62309	down
GOMF_BET	-0.65017	0.000943	-12.2254	1.79E-24	9.11E-24	44.82329	down
GOBP_COI	0.650133	-0.02454	11.73656	3.81E-23	1.75E-22	41.78102	up
GOBP_REN	-0.64993	-0.11405	-12.2348	1.69E-24	8.62E-24	44.88125	down
ZWANG_C	-0.64966	-0.03518	-21.8221	4.65E-49	1.50E-46	101.1995	down
GOBP_NE(	-0.6496	0.011945	-18.3314	1.33E-40	5.24E-39	81.82014	down
OVSYANN	0.648984	0.019918	11.98054	8.28E-24	4.00E-23	43.2988	up
GOBP_NE(	0.648792	-0.11412	16.07955	8.15E-35	1.26E-33	68.54169	up
BEGUM_TA	-0.64838	-0.05399	-22.1592	7.70E-50	3.03E-47	102.9893	down
WP_BASE_	0.648242	0.021502	18.23411	2.33E-40	8.88E-39	81.25858	up
GOBP_RES	-0.64824	0.051424	-9.96493	2.25E-18	7.26E-18	30.85844	down
WP_BMP2V	-0.64815	-0.02694	-18.3581	1.14E-40	4.57E-39	81.97447	down
HP_HYPER	-0.64795	0.234703	-9.63642	1.67E-17	5.07E-17	28.86774	down
PUJANA_B	0.647306	-0.1874	12.1519	2.84E-24	1.42E-23	44.36541	up
GOBP_NEU	-0.64725	0.046937	-17.4965	1.74E-38	4.77E-37	76.96308	down
GOCC_SPA	-0.64709	0.039334	-18.1007	5.06E-40	1.80E-38	80.48652	down
HP_PROGF	-0.64657	0.097346	-12.5683	2.10E-25	1.16E-24	46.9582	down
MODULE_4	-0.64638	0.080471	-12.852	3.56E-26	2.12E-25	48.72387	down
GOBP_PRC	0.646339	0.252473	13.29174	2.29E-27	1.51E-26	51.45768	up
GOBP_EXT	-0.64626	-0.0917	-13.4232	1.01E-27	6.88E-27	52.27388	down
MODULE_1	-0.64626	0.182815	-11.628	7.50E-23	3.37E-22	41.10615	down
GOMF_ATI	-0.646	0.087523	-10.9103	6.58E-21	2.53E-20	36.65773	down
GOBP_CGN	-0.64592	0.015531	-10.3323	2.36E-19	8.13E-19	33.10146	down
GOCC_AM	0.645746	0.061178	9.083294	4.73E-16	1.30E-15	25.55506	up
HP_DECRE	-0.64547	0.084916	-14.5827	7.59E-31	7.23E-30	59.43641	down
GOBP_NE(	-0.64526	-0.06052	-15.3068	8.91E-33	1.06E-31	63.86447	down
REACTOM	-0.64515	0.243502	-10.2632	3.61E-19	1.23E-18	32.67819	down
GOBP_POL	0.645104	0.072544	11.98844	7.89E-24	3.82E-23	43.34795	up
WANG_RE	-0.64503	0.11461	-11.7502	3.50E-23	1.61E-22	41.86553	down

GOMF_C_/	-0.64462	0.096018	-13.7528	1.29E-28	9.75E-28	54.31714	down
MODULE_4	0.644468	-0.14791	14.18829	8.68E-30	7.33E-29	57.00851	up
GOBP_LYS	-0.64444	0.082606	-11.0174	3.38E-21	1.33E-20	37.32007	down
KEGG_HOI	0.644408	-0.11607	15.33016	7.72E-33	9.29E-32	64.00686	up
GOBP_REG	0.644398	-0.18682	13.19433	4.20E-27	2.71E-26	50.85256	up
MODULE_!	-0.64437	0.088434	-15.2306	1.42E-32	1.65E-31	63.40089	down
REACTOM	0.644338	0.051226	17.11407	1.67E-37	3.88E-36	74.71133	up
GOBP_COI	-0.64425	0.079171	-9.82825	5.20E-18	1.63E-17	30.0283	down
GOBP_LEU	-0.64412	0.200929	-8.93006	1.18E-15	3.16E-15	24.64743	down
HP_SPOTT	0.643765	0.036461	14.70943	3.48E-31	3.45E-30	60.21426	up
GOBP_REL	-0.64356	0.155944	-12.666	1.14E-25	6.45E-25	47.56601	down
GOCC_CH/	0.64346	-0.02779	10.13325	8.02E-19	2.66E-18	31.88399	up
GOMF_PO	-0.64313	0.065923	-20.6068	3.44E-46	5.09E-44	94.62759	down
GOBP_POS	-0.64306	0.090386	-15.5243	2.36E-33	3.04E-32	65.18657	down
BOYAULT_	-0.64304	0.132532	-11.8765	1.59E-23	7.51E-23	42.65164	down
GOBP_POS	-0.64302	-0.13278	-11.8012	2.54E-23	1.18E-22	42.18311	down
WP_ESTRC	-0.64275	-0.07903	-20.2751	2.15E-45	2.35E-43	92.80088	down
GOCC_PRE	0.642742	-0.07419	15.0036	5.70E-32	6.20E-31	62.01536	up
GOMF_AR(	-0.64267	0.122416	-13.1811	4.56E-27	2.94E-26	50.77006	down
REACTOM	-0.64247	0.123122	-14.1323	1.23E-29	1.02E-28	56.66311	down
GOBP_POS	0.642315	-0.19435	11.7637	3.21E-23	1.49E-22	41.94982	up
GOBP_SPII	0.642287	-0.19673	13.05064	1.03E-26	6.44E-26	49.9594	up
GOCC_SM_	0.642123	0.040303	20.15508	4.20E-45	4.37E-43	92.13678	up
BIOCARTA	-0.6421	0.17161	-11.9111	1.28E-23	6.10E-23	42.86668	down
REACTOM	0.641791	0.080721	10.76902	1.58E-20	5.93E-20	35.7858	up
GOBP_RES	-0.64174	-0.02629	-17.3568	3.96E-38	1.02E-36	76.14253	down
REACTOM	0.641526	0.287766	12.1196	3.47E-24	1.72E-23	44.16435	up
REACTOM	-0.64144	-0.09632	-14.847	1.49E-31	1.55E-30	61.05748	down
HP_REDUC	-0.64128	0.079114	-10.7037	2.37E-20	8.75E-20	35.38343	down
HARALAM	0.641005	0.023834	18.51796	4.51E-41	1.97E-39	82.89452	up
GOBP_REG	-0.64078	0.018297	-18.0891	5.41E-40	1.91E-38	80.41898	down
HU_GENO	0.640745	-0.06957	18.37642	1.02E-40	4.14E-39	82.07999	up
REACTOM	-0.64074	0.178524	-12.1272	3.31E-24	1.65E-23	44.21141	down
GOBP_RN/	0.640498	0.011646	16.97712	3.76E-37	8.32E-36	73.90105	up
HP_VIRAL_	0.640188	0.132685	15.00939	5.50E-32	5.99E-31	62.05074	up
HP_INCRE/	-0.63996	0.09466	-19.2158	8.29E-43	5.08E-41	86.87483	down
REACTOM	0.639646	-0.072	14.02956	2.32E-29	1.88E-28	56.02871	up
GOBP_REG	-0.63944	0.007096	-21.8053	5.09E-49	1.60E-46	101.11	down
GOMF_PH(	-0.6393	0.0631	-15.0778	3.62E-32	4.02E-31	62.46861	down
REACTOM	-0.63919	0.117742	-14.3019	4.30E-30	3.75E-29	57.70868	down
GOBP_KIN	0.639027	-0.17017	14.79643	2.04E-31	2.07E-30	60.74764	up
WENG_PO	-0.6389	0.099052	-15.4839	3.02E-33	3.83E-32	64.94116	down
GOCC_PR(	0.638869	-0.06369	15.04669	4.38E-32	4.81E-31	62.27868	up
MODULE_4	0.638819	-0.13584	13.44071	9.03E-28	6.20E-27	52.38249	up
MULLIGAN	-0.63868	0.059112	-12.5718	2.05E-25	1.13E-24	46.97968	down
GOBP_NE(	-0.63856	0.125405	-10.7729	1.54E-20	5.79E-20	35.80991	down
HP_PULM(	-0.63825	-0.02489	-9.86737	4.09E-18	1.30E-17	30.26564	down
HP_ACETA	0.638052	-0.01357	18.74599	1.21E-41	5.80E-40	84.20167	up
REACTOM	0.63803	0.000861	10.29252	3.01E-19	1.03E-18	32.85755	up
ACEVEDO_	-0.63791	0.139182	-19.9114	1.64E-44	1.50E-42	90.78213	down
GOMF_URI	-0.63785	0.132314	-12.2898	1.20E-24	6.20E-24	45.22377	down
GOBP_NE(	-0.63773	0.071005	-19.3389	4.12E-43	2.66E-41	87.57096	down
BIOCARTA	-0.6376	0.086692	-9.62575	1.79E-17	5.40E-17	28.80338	down
GOBP_CEL	0.637457	-0.01246	12.15163	2.84E-24	1.42E-23	44.36374	up
GOBP_DN/	0.637364	-0.16816	12.39331	6.26E-25	3.32E-24	45.86854	up
HP_FINGEI	0.637309	0.003838	11.17849	1.24E-21	5.05E-21	38.31682	up
GOBP_MO	-0.63725	-0.04159	-15.2013	1.70E-32	1.96E-31	63.22197	down
MORF_ERH	0.637053	0.116362	15.51486	2.50E-33	3.21E-32	65.12924	up

SOBOLEV_	-0.63699	0.07725	-12.9648	1.76E-26	1.08E-25	49.42531	down
KEGG_PRIM	-0.63689	0.144857	-10.6208	3.96E-20	1.44E-19	34.87298	down
BIOCARTA	0.636852	-0.07686	12.93465	2.12E-26	1.29E-25	49.23804	up
MODULE_4	-0.63653	0.133043	-13.8834	5.75E-29	4.48E-28	55.12511	down
HP_CORO	-0.63648	0.075118	-12.5545	2.29E-25	1.26E-24	46.8721	down
HP_IMPAIF	-0.63611	-0.02963	-12.9278	2.22E-26	1.35E-25	49.19538	down
BIOCARTA	-0.63582	-0.05045	-19.5258	1.43E-43	1.03E-41	88.624	down
HP_ABNOI	-0.63574	0.138333	-12.2893	1.20E-24	6.21E-24	45.22116	down
GOBP_PRC	-0.63552	0.009259	-16.4562	8.44E-36	1.52E-34	70.80077	down
GOBP_RIG	-0.63544	-0.08918	-13.595	3.46E-28	2.48E-27	53.33915	down
REACTOM	-0.63529	-0.05287	-10.595	4.65E-20	1.68E-19	34.71387	down
HP_WIDEN	-0.63516	0.23441	-7.83272	7.14E-13	1.60E-12	18.31024	down
GOBP_TRN	0.635097	0.17772	13.70637	1.73E-28	1.28E-27	54.02949	up
GOBP_DN/	0.635079	-0.18038	13.91206	4.81E-29	3.80E-28	55.3025	up
GOMF_RRI	0.635018	0.019915	10.30196	2.84E-19	9.74E-19	32.91536	up
WOO_LIVE	-0.63495	0.166067	-10.4224	1.35E-19	4.74E-19	33.65337	down
HP_OLIVO	-0.63489	-0.08711	-12.6358	1.37E-25	7.73E-25	47.37823	down
KOBAYASH	0.634882	-0.10443	13.98424	3.08E-29	2.47E-28	55.74868	up
GOBP_HIS	-0.6346	0.190099	-10.7336	1.97E-20	7.33E-20	35.56725	down
REACTOM	-0.6346	0.190099	-10.7336	1.97E-20	7.33E-20	35.56725	down
GOBP_THY	-0.63457	-0.15132	-12.188	2.26E-24	1.14E-23	44.59048	down
GOMF_1_F	-0.63405	-0.19101	-13.2119	3.76E-27	2.44E-26	50.96173	down
GOMF_SIN	0.633848	-0.15479	12.15747	2.74E-24	1.37E-23	44.40011	up
REACTOM	0.633699	0.005936	19.28221	5.68E-43	3.57E-41	87.2505	up
GOBP_RES	-0.63368	0.036905	-12.4343	4.85E-25	2.59E-24	46.12404	down
GROSS_HY	-0.63364	0.078622	-14.6277	5.75E-31	5.56E-30	59.71249	down
chr3q11	-0.63349	-0.01229	-15.7827	4.92E-34	6.90E-33	66.75136	down
HP_THIN_(	0.633431	-0.15953	13.17406	4.76E-27	3.06E-26	50.72657	up
GOBP_POS	-0.63321	0.087123	-17.7734	3.42E-39	1.05E-37	78.58291	down
GCM_ACT(	0.633069	0.106872	16.06304	9.01E-35	1.39E-33	68.44232	up
GCM_PPP1	0.632687	0.018195	15.30222	9.16E-33	1.09E-31	63.83677	up
GOMF_U1_	0.632592	0.012897	14.35134	3.17E-30	2.81E-29	58.01341	up
CHEMNITZ	0.632561	-0.08939	19.25205	6.74E-43	4.20E-41	87.07997	up
WP_REGUI	0.632353	-0.19235	12.94814	1.95E-26	1.19E-25	49.32198	up
GOBP_CEN	0.632101	-0.16363	14.2965	4.44E-30	3.87E-29	57.67558	up
GOBP_2_O	-0.6321	0.11808	-10.8283	1.09E-20	4.15E-20	36.15146	down
GOBP_DN/	0.632085	-0.18505	12.58957	1.84E-25	1.02E-24	47.09049	up
REACTOM	0.632012	0.161822	15.15413	2.27E-32	2.59E-31	62.93446	up
BIOCARTA	-0.63187	-0.00895	-15.1527	2.29E-32	2.60E-31	62.92575	down
GOCC_HAI	0.631792	-0.07949	12.83071	4.07E-26	2.41E-25	48.59141	up
HOFFMAN	0.631717	-0.11566	15.91916	2.15E-34	3.17E-33	67.5755	up
GOBP_REG	-0.63165	0.071784	-17.6606	6.63E-39	1.94E-37	77.92392	down
GOBP_DET	-0.63159	-0.00774	-11.4662	2.06E-22	8.93E-22	40.10133	down
GNF2_BUB	0.631387	0.003092	14.65433	4.88E-31	4.77E-30	59.8762	up
GOBP_EST.	0.630984	-0.06172	13.87708	5.98E-29	4.65E-28	55.08616	up
WP_CONS	-0.63095	0.038523	-13.7882	1.04E-28	7.90E-28	54.53595	down
GOMF_CX(	-0.63083	0.073139	-13.7987	9.73E-29	7.42E-28	54.60146	down
HP_TALIPE	0.630812	-0.0191	14.603	6.70E-31	6.43E-30	59.56105	up
GOCC_AST	-0.63073	0.006571	-24.0635	3.88E-54	7.83E-51	112.8258	down
GSE14415_	0.630692	-0.02926	18.05577	6.57E-40	2.29E-38	80.22581	up
GOBP_PRC	0.63051	-0.15381	14.7249	3.16E-31	3.15E-30	60.30911	up
KERLEY_RE	-0.63039	0.036256	-10.416	1.41E-19	4.93E-19	33.61444	down
WP_COMF	-0.63028	0.126613	-14.1445	1.14E-29	9.48E-29	56.73856	down
REACTOM	-0.63025	0.190456	-8.6892	4.94E-15	1.27E-14	23.23082	down
GOBP_SPE	-0.63011	0.036193	-12.504	3.13E-25	1.70E-24	46.55796	down
GOMF_3_F	-0.62995	0.182224	-8.76232	3.20E-15	8.34E-15	23.65951	down
HP_ERYTH	0.629808	0.296354	12.30777	1.07E-24	5.57E-24	45.3359	up
REACTOM	-0.62969	0.182187	-12.951	1.92E-26	1.17E-25	49.33974	down

HP_PERSIS	-0.62963	0.002284	-13.3917	1.23E-27	8.32E-27	52.07829	down
MARTINEL	-0.6296	0.066669	-11.5064	1.60E-22	7.00E-22	40.35105	down
MORF_NPI	0.629424	0.195933	12.70585	8.87E-26	5.09E-25	47.81434	up
GOBP_POS	-0.62941	0.014352	-13.8888	5.56E-29	4.35E-28	55.15866	down
MIR551A_I	-0.62936	0.061925	-10.5575	5.86E-20	2.11E-19	34.48334	down
THAKAR_P	-0.62913	0.03393	-12.1914	2.22E-24	1.12E-23	44.61147	down
HP_INCRE/	-0.62908	0.008291	-9.59958	2.09E-17	6.29E-17	28.64549	down
GOMF_AN	0.62905	-0.12836	12.52416	2.76E-25	1.51E-24	46.68325	up
GOBP_PRC	0.629018	0.159988	10.58828	4.84E-20	1.75E-19	34.67263	up
MIR1247_5	-0.62841	0.031287	-13.1813	4.55E-27	2.93E-26	50.7718	down
WANG_RE:	-0.62834	-0.06355	-12.3857	6.57E-25	3.47E-24	45.82129	down
GOBP_REG	-0.62806	-0.12473	-12.2837	1.24E-24	6.42E-24	45.18617	down
BOYAULT_	0.628046	-0.18244	13.59017	3.56E-28	2.55E-27	53.30943	up
GOBP_PRC	0.627997	-0.01335	12.16415	2.63E-24	1.32E-23	44.44168	up
GNF2_FOS	-0.62799	0.102117	-13.7983	9.76E-29	7.44E-28	54.59857	down
GSE9006_T	0.627963	0.134268	18.76738	1.07E-41	5.21E-40	84.324	up
GOBP_NEC	-0.62782	0.053188	-13.6057	3.23E-28	2.33E-27	53.40546	down
GOBP_POS	-0.6276	-0.10694	-17.1629	1.25E-37	2.96E-36	74.99994	down
GOCC_PO:	-0.62751	-0.13043	-11.7767	2.96E-23	1.38E-22	42.03054	down
GOBP_NEC	-0.62744	-0.00176	-9.84584	4.67E-18	1.47E-17	30.135	down
REACTOM	0.627405	0.082919	18.41663	8.10E-41	3.39E-39	82.31163	up
HP_CHROI	-0.62739	0.082309	-14.0454	2.10E-29	1.71E-28	56.12645	down
MSX2_TAR	0.627375	0.019418	17.06633	2.21E-37	5.07E-36	74.42913	up
SCHLOSSE	0.627295	0.110315	16.11112	6.74E-35	1.06E-33	68.73155	up
HP_PILOM	-0.62722	-0.20271	-13.4584	8.09E-28	5.59E-27	52.49216	down
GNF2_TPT:	0.627086	0.314841	9.887917	3.61E-18	1.15E-17	30.39038	up
GOCC_BLC	0.627065	0.054121	15.61852	1.33E-33	1.76E-32	65.75783	up
GOMF_INS	-0.62682	0.16303	-12.9644	1.76E-26	1.08E-25	49.42314	down
REACTOM	-0.6268	0.108712	-15.1863	1.86E-32	2.14E-31	63.13089	down
GOBP_CHE	-0.62676	-0.06589	-12.4698	3.88E-25	2.09E-24	46.34489	down
MORF_AN	0.626726	0.104181	15.91666	2.18E-34	3.22E-33	67.5604	up
WP_DEREC	-0.62663	0.065308	-17.774	3.41E-39	1.05E-37	78.58617	down
HARALAM	-0.62628	0.02349	-13.9662	3.44E-29	2.75E-28	55.63688	down
GOBP_TOL	-0.62622	0.03928	-15.8165	4.00E-34	5.69E-33	66.95577	down
GOCC_PEF	-0.62619	-0.04103	-10.4971	8.52E-20	3.03E-19	34.11189	down
REACTOM	0.626151	0.026908	13.87866	5.92E-29	4.61E-28	55.09589	up
GOBP_ELA	-0.62605	-0.0387	-11.1831	1.20E-21	4.92E-21	38.34533	down
GOCC_SPE	-0.626	-0.07718	-15.9833	1.46E-34	2.20E-33	67.9621	down
GOMF_HIS	0.625979	-0.21273	10.72585	2.07E-20	7.67E-20	35.51965	up
FU_INTER/	0.625953	0.024706	10.65405	3.22E-20	1.18E-19	35.07737	up
GOBP_FUN	0.625883	-0.10608	10.2025	5.24E-19	1.76E-18	32.30697	up
MODULE_:	-0.62577	0.127264	-11.8327	2.09E-23	9.79E-23	42.37862	down
BIOCARTA	-0.62572	0.136243	-12.0986	3.96E-24	1.96E-23	44.03387	down
GOBP_GLY	-0.62557	0.208935	-10.2795	3.26E-19	1.11E-18	32.77818	down
MORF_AC	0.625445	0.216697	12.68063	1.04E-25	5.91E-25	47.65737	up
RODRIGUE	-0.62541	-0.01583	-20.5531	4.62E-46	6.40E-44	94.3328	down
PID_ATR_P	0.625278	-0.14904	13.89762	5.27E-29	4.12E-28	55.21316	up
GOBP_REG	-0.62527	-0.1268	-10.0126	1.68E-18	5.46E-18	31.14831	down
GOCC_U2/	0.625081	0.054779	10.1001	9.84E-19	3.24E-18	31.68176	up
GOBP_REG	0.625065	-0.12511	15.79137	4.66E-34	6.58E-33	66.80387	up
HALLMARI	0.624932	-0.13056	13.95209	3.75E-29	2.99E-28	55.54995	up
GOBP_REG	0.624895	-0.05567	16.506	6.26E-36	1.15E-34	71.09836	up
GOMF_3_5	-0.62478	0.011238	-14.9362	8.62E-32	9.21E-31	61.60301	down
KEGG_BET/	-0.62458	0.13586	-13.0307	1.17E-26	7.27E-26	49.83537	down
GOMF_OX	0.624541	-0.12628	14.29202	4.57E-30	3.97E-29	57.64796	up
HP_DECRE	-0.62421	0.06139	-12.7929	5.15E-26	3.02E-25	48.35583	down
GSE14415_	0.624204	-0.10443	15.10795	3.01E-32	3.38E-31	62.65268	up
WP_METH	-0.62397	0.111536	-10.7114	2.26E-20	8.36E-20	35.43069	down

HERNAND	-0.62392	0.017673	-20.5306	5.23E-46	7.15E-44	94.20933	down
HP_PROXII	-0.62384	-0.05563	-11.8221	2.23E-23	1.04E-22	42.31307	down
GOMF_GLY	-0.62375	0.15271	-10.299	2.89E-19	9.91E-19	32.89728	down
GOBP_NEU	-0.62368	0.125064	-12.1754	2.45E-24	1.23E-23	44.51183	down
KEGG_TRY	-0.62363	0.130541	-13.7397	1.40E-28	1.05E-27	54.23608	down
WP_VITAM	-0.62355	0.051633	-9.53269	3.14E-17	9.34E-17	28.24254	down
HP_ETHYLI	-0.62334	0.204602	-10.0648	1.22E-18	4.00E-18	31.46678	down
PID_EPHRI	-0.62331	0.032118	-20.0383	8.05E-45	7.97E-43	91.48878	down
LU_TUMOI	-0.62321	0.032795	-12.7882	5.30E-26	3.10E-25	48.32669	down
GSE36476_	0.623128	-0.05478	17.45818	2.18E-38	5.90E-37	76.73807	up
GOBP_POS	-0.6231	0.13161	-10.233	4.35E-19	1.47E-18	32.49314	down
GOBP_HIS	0.622938	-0.11629	15.79666	4.52E-34	6.38E-33	66.8359	up
BIOCARTA	-0.6229	0.053996	-18.3154	1.45E-40	5.69E-39	81.72785	down
HP_ELEVA	-0.62274	0.059657	-14.4549	1.67E-30	1.53E-29	58.65085	down
MIR4445_3	-0.62273	-0.2062	-15.9559	1.72E-34	2.57E-33	67.79692	down
GOBP_REG	-0.6227	0.041591	-14.2362	6.45E-30	5.52E-29	57.30417	down
GOMF_PRI	0.622591	0.021512	11.72047	4.21E-23	1.93E-22	41.681	up
HP_HYPER	-0.62256	0.126323	-10.2227	4.63E-19	1.56E-18	32.43023	down
BURTON_A	0.622543	-0.14794	12.90735	2.52E-26	1.52E-25	49.06823	up
GOMF_DN	0.622368	-0.18076	11.92991	1.14E-23	5.44E-23	42.98372	up
GOBP_HIS	-0.6223	-0.05788	-11.0507	2.75E-21	1.09E-20	37.52589	down
GOMF_HIS	-0.6223	-0.05788	-11.0507	2.75E-21	1.09E-20	37.52589	down
GOMF_CO	-0.62186	0.096618	-8.80715	2.45E-15	6.44E-15	23.92293	down
KHETCHOU	-0.62185	0.091472	-10.9226	6.09E-21	2.35E-20	36.73366	down
GOBP_REG	-0.62157	-0.00777	-11.5285	1.40E-22	6.14E-22	40.48788	down
LAKE_ADU	-0.62148	-0.0811	-23.7298	2.13E-53	2.75E-50	111.1351	down
GOBP_AM	-0.62124	0.068878	-12.0422	5.63E-24	2.76E-23	43.6824	down
NADERI_BI	0.621235	-0.07336	17.13458	1.48E-37	3.46E-36	74.83248	up
GOMF_RN	-0.62119	-0.12992	-10.495	8.63E-20	3.07E-19	34.09904	down
GOBP_TYR	-0.62112	0.19796	-11.0121	3.49E-21	1.37E-20	37.28689	down
GOBP_REG	-0.62111	0.036302	-10.2862	3.13E-19	1.07E-18	32.81887	down
MORF_G22	0.620871	0.076294	15.41033	4.74E-33	5.87E-32	64.49439	up
GOCC_ENI	0.620818	-0.12767	12.26988	1.36E-24	6.98E-24	45.09997	up
HP_INABIL	-0.62027	0.030913	-12.4827	3.58E-25	1.93E-24	46.42524	down
KEGG_GLY	-0.62026	0.156819	-11.5221	1.45E-22	6.38E-22	40.44818	down
GOBP_ACT	0.620175	-0.16708	13.48053	7.05E-28	4.89E-27	52.62952	up
WP_KYNUI	-0.62006	0.12258	-15.9516	1.77E-34	2.63E-33	67.77126	down
GOBP_NEG	-0.61994	0.065246	-19.5981	9.51E-44	7.14E-42	89.02995	down
NAKAMUR	-0.61993	0.026061	-19.7552	3.93E-44	3.24E-42	89.91031	down
GOMF_ENI	0.619829	-0.08688	14.43022	1.94E-30	1.77E-29	58.49894	up
GOCC_PRE	0.619806	0.078839	9.673907	1.33E-17	4.06E-17	29.09412	up
GOBP_NEG	-0.61977	0.166762	-11.9806	8.28E-24	4.00E-23	43.29894	down
WP_MITOC	-0.61958	0.150967	-11.2468	8.10E-22	3.34E-21	38.74024	down
WEST_ADF	-0.61956	0.109064	-14.7784	2.27E-31	2.30E-30	60.63703	down
GOBP_HYD	-0.61946	0.115928	-14.6823	4.11E-31	4.04E-30	60.04799	down
REACTOM	0.61938	0.179857	13.90198	5.12E-29	4.02E-28	55.24014	up
GOMF_VA	-0.61901	-0.08247	-10.1094	9.29E-19	3.07E-18	31.73852	down
REACTOM	-0.61899	0.1294	-14.0141	2.56E-29	2.06E-28	55.93289	down
REACTOM	-0.61891	0.123151	-14.5192	1.12E-30	1.05E-29	59.04606	down
REACTOM	0.618859	-0.18773	11.2774	6.69E-22	2.78E-21	38.92977	up
GOBP_REG	-0.61874	0.060768	-13.6066	3.21E-28	2.31E-27	53.41123	down
CAIRO_HE	0.618672	-0.05304	17.16428	1.24E-37	2.94E-36	75.00789	up
GALIE_TUN	-0.61857	-0.07823	-12.0944	4.06E-24	2.01E-23	44.00746	down
GOMF_NF	-0.61853	0.023697	-12.3588	7.77E-25	4.09E-24	45.65387	down
REACTOM	-0.61849	0.145863	-10.3717	1.85E-19	6.42E-19	33.34261	down
GOBP_TES	0.618431	0.010563	12.5043	3.13E-25	1.70E-24	46.55959	up
GOBP_CAF	-0.61828	0.037538	-12.6022	1.70E-25	9.44E-25	47.16923	down
REACTOM	-0.61818	0.052169	-12.3653	7.46E-25	3.93E-24	45.69426	down

GOBP_BOX	0.618032	0.079518	10.66767	2.96E-20	1.09E-19	35.16127	up
MODULE_:	-0.61771	0.082902	-12.7871	5.34E-26	3.12E-25	48.31987	down
HP_WIDEN	0.61768	0.079465	14.16194	1.02E-29	8.55E-29	56.84591	up
GNF2_EIF3	0.617636	0.24069	11.62835	7.49E-23	3.36E-22	41.10835	up
SPIRA_SM(	-0.61763	0.011134	-18.7657	1.08E-41	5.24E-40	84.31411	down
GIAROLA_!	-0.61723	-0.04261	-10.6658	3.00E-20	1.10E-19	35.14979	down
REACTOM	-0.61719	0.209595	-10.1933	5.55E-19	1.86E-18	32.25074	down
GOBP_POS	-0.61714	-0.11136	-10.1926	5.57E-19	1.87E-18	32.24632	down
GARGALO'	-0.6171	-0.08792	-20.0395	7.99E-45	7.94E-43	91.49557	down
GSE13547_	0.617095	-0.12013	14.16503	1.00E-29	8.39E-29	56.86499	up
HEDVAT_E	-0.61702	0.10978	-14.1561	1.06E-29	8.85E-29	56.80972	down
GOBP_NE(	-0.61701	0.031417	-7.85869	6.16E-13	1.39E-12	18.45639	down
LAKE_ADU	-0.61693	-0.11972	-19.9885	1.06E-44	1.02E-42	91.21197	down
GOBP_POS	-0.61678	0.148695	-11.5262	1.42E-22	6.22E-22	40.47362	down
GNF2_FBL	0.616673	0.124985	14.26371	5.44E-30	4.70E-29	57.47355	up
HP_SEVERI	0.616619	-0.10911	12.17715	2.42E-24	1.22E-23	44.52262	up
VAN_DEN_	0.616518	-0.12141	12.81653	4.44E-26	2.62E-25	48.50315	up
GOBP_REG	0.616145	-0.08864	11.31223	5.39E-22	2.25E-21	39.14576	up
GOBP_BILE	-0.61612	0.036844	-13.9144	4.74E-29	3.74E-28	55.31715	down
HP_MULTII	-0.6159	-0.08478	-12.2701	1.35E-24	6.97E-24	45.10143	down
chr4q22	-0.61569	0.043683	-15.7605	5.62E-34	7.84E-33	66.61752	down
REACTOM	0.615608	0.041585	18.16587	3.46E-40	1.28E-38	80.86386	up
GOMF_ATI	0.615411	-0.16578	12.38543	6.58E-25	3.48E-24	45.81946	up
HP_SMOO	-0.61517	0.043798	-11.2854	6.36E-22	2.65E-21	38.97965	down
GOBP_POS	0.614971	0.10079	11.69666	4.89E-23	2.23E-22	41.53297	up
GOBP_MET	0.614864	-0.10962	16.58414	3.92E-36	7.46E-35	71.56482	up
ZHONG_PI	0.614786	-0.03057	19.13412	1.32E-42	7.67E-41	86.41204	up
GOBP_NE(	-0.61478	0.038621	-16.8998	5.95E-37	1.29E-35	73.44268	down
GOBP_REG	0.614779	-0.1321	11.63321	7.26E-23	3.26E-22	41.13854	up
HP_OROTI	-0.61474	0.145466	-10.7617	1.65E-20	6.19E-20	35.74042	down
CHIANG_L	0.614742	-0.14369	11.82796	2.15E-23	1.01E-22	42.34944	up
GOCC_PR(	0.61474	-0.06623	14.06346	1.88E-29	1.54E-28	56.23809	up
MODULE_'	-0.61472	0.049143	-13.2991	2.18E-27	1.45E-26	51.50365	down
GOCC_MIT	0.614717	-0.10712	15.10905	2.99E-32	3.36E-31	62.6594	up
GOBP_POS	0.614692	-0.08654	16.35588	1.54E-35	2.67E-34	70.20045	up
AZARE_ST/	-0.61453	-0.07519	-16.264	2.68E-35	4.45E-34	69.64965	down
HP_OSTEC	0.614142	0.185116	14.58549	7.46E-31	7.12E-30	59.4535	up
MORF_BUI	0.614044	-0.11991	14.18163	9.05E-30	7.63E-29	56.96742	up
GOBP_AN/	0.614004	0.055055	17.72613	4.51E-39	1.36E-37	78.307	up
KEGG_CON	-0.614	0.138616	-13.0687	9.19E-27	5.78E-26	50.07199	down
chr3p11	-0.61387	-0.04423	-11.1627	1.37E-21	5.55E-21	38.21912	down
REACTOM	-0.61378	0.154904	-11.5031	1.64E-22	7.14E-22	40.33047	down
REACTOM	0.613531	-0.05484	16.43284	9.71E-36	1.73E-34	70.66104	up
GOBP_REG	-0.61328	-0.0106	-11.488	1.80E-22	7.83E-22	40.23652	down
chr14q31	-0.61327	-0.06912	-13.7689	1.17E-28	8.87E-28	54.4168	down
GOCC_CUI	0.613163	-0.06044	12.233	1.71E-24	8.71E-24	44.87036	up
WP_CODE	-0.61302	0.054885	-10.7391	1.90E-20	7.09E-20	35.60127	down
HP_ABNOI	-0.61299	0.215937	-9.8361	4.96E-18	1.56E-17	30.07592	down
GOCC_PR(	0.612952	0.129833	10.21381	4.89E-19	1.65E-18	32.37611	up
SCHAEFFEI	-0.61285	-0.00369	-12.6988	9.27E-26	5.31E-25	47.77045	down
MORF_RAI	0.61283	0.12539	14.8778	1.23E-31	1.29E-30	61.24592	up
GOMF_TO.	-0.6128	0.061455	-14.56	8.73E-31	8.25E-30	59.29704	down
GOBP_CEL	-0.61275	0.114416	-15.2428	1.32E-32	1.54E-31	63.47503	down
GOBP_CAT	-0.61266	0.078653	-18.6773	1.80E-41	8.34E-40	83.80871	down
GOBP_PR(	0.612609	-0.09957	13.89922	5.21E-29	4.09E-28	55.22307	up
GOCC_U2_	0.612563	0.046628	17.73654	4.24E-39	1.28E-37	78.36782	up
GAUSSMA	-0.61229	-0.06694	-13.1072	7.23E-27	4.58E-26	50.31127	down
HP_PROLC	-0.61216	-0.00651	-16.1467	5.44E-35	8.61E-34	68.94538	down

GOMF_AP_	-0.61206	0.105808	-12.2276	1.77E-24	9.00E-24	44.83669	down
WP_EFFEC	-0.61195	0.06161	-12.6665	1.14E-25	6.43E-25	47.56917	down
VALK_AML	-0.61191	-0.01549	-17.3189	4.96E-38	1.25E-36	75.91913	down
GOBP_COI	-0.61184	0.174288	-13.3832	1.29E-27	8.76E-27	52.02531	down
REACTOM	-0.61159	-0.1459	-15.7536	5.86E-34	8.15E-33	66.57571	down
GOBP_INT	-0.61158	-0.11515	-10.7521	1.76E-20	6.56E-20	35.68153	down
VAN_DEN_	-0.61142	0.1056	-9.57917	2.37E-17	7.10E-17	28.52245	down
GOBP_CEL	-0.61136	0.018999	-13.7757	1.12E-28	8.51E-28	54.45857	down
GOMF_C_X	-0.61133	0.143176	-10.4663	1.03E-19	3.64E-19	33.9233	down
GOBP_LOI	-0.61103	0.084472	-16.6611	2.47E-36	4.83E-35	72.02354	down
GOCC_PRC	0.61092	-0.21937	11.1411	1.56E-21	6.32E-21	38.0853	up
LAKE_ADU	-0.61081	-0.10795	-18.4761	5.74E-41	2.46E-39	82.65399	down
GOBP_NEC	-0.61076	-0.17525	-10.7156	2.20E-20	8.16E-20	35.45644	down
REACTOM	-0.61065	0.132781	-11.6372	7.09E-23	3.19E-22	41.16325	down
REACTOM	-0.61053	0.049943	-15.2529	1.24E-32	1.45E-31	63.53668	down
WP_BLOOI	-0.61053	0.120026	-12.603	1.69E-25	9.40E-25	47.17419	down
BIERIE_INF	-0.61046	0.085254	-9.70966	1.07E-17	3.29E-17	29.31023	down
GOBP_POS	-0.61002	0.031057	-11.4873	1.81E-22	7.86E-22	40.2321	down
REACTOM	0.61001	0.247191	11.59083	9.47E-23	4.21E-22	40.8752	up
GOBP_POS	-0.61001	-0.00825	-19.5788	1.06E-43	7.81E-42	88.92152	down
REACTOM	-0.60986	0.103101	-10.0157	1.65E-18	5.36E-18	31.16753	down
GREENBAU	0.609843	-0.18718	12.52272	2.79E-25	1.52E-24	46.67431	up
GOBP_REG	-0.60974	0.145613	-11.7588	3.31E-23	1.53E-22	41.91948	down
LAKE_ADU	-0.60974	-0.11415	-18.3383	1.27E-40	5.07E-39	81.85989	down
GOBP_REG	-0.60967	-0.02959	-13.3864	1.27E-27	8.59E-27	52.04513	down
GOBP_FIBI	-0.60941	0.135761	-13.4536	8.34E-28	5.75E-27	52.46219	down
ELVIDGE_H	-0.60907	-0.11897	-12.2937	1.17E-24	6.05E-24	45.24849	down
TAKEDA_T.	-0.60898	0.051555	-16.0426	1.02E-34	1.57E-33	68.31946	down
HP_INTRAI	-0.60893	0.163099	-11.3346	4.69E-22	1.97E-21	39.28421	down
ZHENG_RE	-0.60869	0.169317	-11.0247	3.23E-21	1.27E-20	37.36509	down
PANAPAS/	-0.60833	-0.01435	-8.98817	8.35E-16	2.25E-15	24.99108	down
GOBP_CD4	-0.60823	0.111781	-15.4743	3.21E-33	4.04E-32	64.88312	down
GOBP_DRU	-0.60822	0.123968	-12.8116	4.58E-26	2.70E-25	48.47264	down
GOBP_RES	-0.60779	0.222792	-14.2647	5.41E-30	4.67E-29	57.47945	down
HP_EPISOI	-0.60765	0.001794	-13.8474	7.19E-29	5.56E-28	54.90233	down
GOBP_REG	-0.60764	0.120815	-11.9034	1.34E-23	6.39E-23	42.81906	down
MOREAUX	0.607452	0.011562	18.83812	7.15E-42	3.60E-40	84.72804	up
GOBP_TYR	-0.60714	0.245469	-7.89851	4.91E-13	1.11E-12	18.68094	down
REACTOM	-0.60714	0.245469	-7.89851	4.91E-13	1.11E-12	18.68094	down
GOBP_TRA	-0.60712	-0.10528	-14.2929	4.55E-30	3.95E-29	57.65327	down
HP_SPOTT	0.606995	0.005951	16.09465	7.44E-35	1.16E-33	68.63252	up
MODULE_2	-0.60677	0.002884	-14.7746	2.33E-31	2.36E-30	60.61356	down
REACTOM	-0.6066	0.125567	-10.6636	3.04E-20	1.11E-19	35.13632	down
GOBP_REC	-0.6065	0.139888	-13.9661	3.44E-29	2.75E-28	55.63668	down
WP_ALANI	-0.60629	0.184916	-10.7558	1.72E-20	6.42E-20	35.70418	down
GOBP_NAI	-0.606	0.092818	-13.5846	3.69E-28	2.64E-27	53.27476	down
GOBP_COI	-0.60597	-0.08886	-16.5038	6.35E-36	1.17E-34	71.08502	down
GNF2_CYP	-0.60583	0.256992	-10.2035	5.21E-19	1.75E-18	32.31287	down
CGTCTTA_	-0.60576	-0.14406	-11.9023	1.35E-23	6.44E-23	42.81179	down
GOMF_GLI	-0.60575	-0.05668	-14.4939	1.31E-30	1.21E-29	58.89033	down
REACTOM	0.60548	0.042318	17.70155	5.21E-39	1.55E-37	78.16343	up
REACTOM	-0.60543	-0.05976	-15.8859	2.63E-34	3.83E-33	67.37462	down
MORF_TPT	0.605317	0.250728	11.23926	8.49E-22	3.50E-21	38.69337	up
GOBP_NEC	0.605303	-0.10516	17.06742	2.20E-37	5.04E-36	74.43556	up
BOYAULT_	-0.60519	0.184199	-9.26066	1.63E-16	4.62E-16	26.61139	down
MISSIAGLI/	0.605167	-0.08916	14.68154	4.13E-31	4.06E-30	60.04316	up
GOBP_CEL	-0.60513	0.134938	-12.9313	2.17E-26	1.32E-25	49.21722	down
GOBP_TEL	0.605115	-0.11625	13.71046	1.68E-28	1.25E-27	54.0548	up

GOBP_BLC	-0.60506	0.164559	-10.9954	3.87E-21	1.52E-20	37.18401	down
REACTOM	-0.60503	0.035259	-17.6545	6.87E-39	2.00E-37	77.88848	down
GARGALO'	-0.60497	-0.07975	-12.082	4.39E-24	2.17E-23	43.93018	down
HP_HETER	-0.60496	-0.14468	-12.2196	1.86E-24	9.44E-24	44.78715	down
HP_JUVEN	0.604789	-0.11604	13.73842	1.42E-28	1.06E-27	54.22797	up
HSIAO_LIV	-0.60477	0.187147	-11.3873	3.37E-22	1.44E-21	39.61173	down
chr4p11	-0.6044	0.023331	-10.9979	3.81E-21	1.49E-20	37.19913	down
MODULE_:	-0.60404	0.154103	-12.3979	6.09E-25	3.23E-24	45.89689	down
GOBP_DN/	0.603961	-0.13473	12.84774	3.65E-26	2.17E-25	48.69734	up
GOBP_REG	-0.60375	0.174111	-12.5946	1.78E-25	9.88E-25	47.12186	down
REACTOM	0.603741	-0.08314	18.22866	2.41E-40	9.15E-39	81.22704	up
WP_SULIN	-0.60373	0.146851	-10.1349	7.94E-19	2.64E-18	31.89414	down
LUDWICZE	-0.60365	0.100369	-12.5493	2.36E-25	1.30E-24	46.8398	down
MODULE_4	-0.60332	0.081256	-16.3791	1.34E-35	2.35E-34	70.33942	down
GOBP_PHE	-0.60331	0.145984	-9.59502	2.15E-17	6.46E-17	28.61799	down
BURTON_/	0.603271	-0.07502	14.62465	5.86E-31	5.66E-30	59.69399	up
MIR548AL	-0.6031	-0.21418	-16.3769	1.36E-35	2.37E-34	70.32648	down
GOBP_REG	-0.60295	0.061792	-20.9631	4.86E-47	9.51E-45	96.57392	down
GOBP_RES	-0.60292	0.141754	-11.5279	1.40E-22	6.16E-22	40.4844	down
GOBP_CEL	-0.60292	0.141754	-11.5279	1.40E-22	6.16E-22	40.4844	down
GOBP_NEC	-0.6026	-0.05263	-12.0087	6.95E-24	3.38E-23	43.47401	down
GOBP_POS	0.602332	-0.03278	10.66643	2.99E-20	1.09E-19	35.15361	up
GOMF_NU	0.602228	-0.02752	11.52815	1.40E-22	6.15E-22	40.48585	up
GOBP_MO	-0.60198	0.048716	-9.978	2.08E-18	6.71E-18	30.93791	down
GOCC_PRC	0.601823	0.000299	11.31243	5.38E-22	2.25E-21	39.14701	up
HP_BRANC	0.601793	-0.13216	12.90116	2.62E-26	1.58E-25	49.02975	up
GOBP_POS	-0.60176	-0.08198	-13.4351	9.35E-28	6.41E-27	52.34796	down
GOMF_PEF	-0.60173	0.1845	-11.2882	6.25E-22	2.60E-21	38.99701	down
GOBP_MEI	-0.6017	-0.12591	-11.0508	2.74E-21	1.09E-20	37.52621	down
MORF_UN	0.601565	0.032403	14.42651	1.99E-30	1.81E-29	58.47613	up
GOBP_CEL	-0.60147	-0.00607	-13.9209	4.56E-29	3.60E-28	55.35701	down
GOMF_ENI	0.601247	0.02853	19.58578	1.02E-43	7.58E-42	88.96072	up
GOBP_AM	-0.60121	-0.15813	-12.5643	2.15E-25	1.19E-24	46.93346	down
GOMF_AM	-0.60121	-0.15813	-12.5643	2.15E-25	1.19E-24	46.93346	down
HP_RENAL	-0.60106	0.147768	-10.4959	8.58E-20	3.05E-19	34.10484	down
GOMF_STF	0.600946	0.284468	11.54491	1.26E-22	5.55E-22	40.58995	up
GOBP_NEC	0.600934	-0.06479	11.80937	2.42E-23	1.13E-22	42.23383	up
GOBP_REP	0.600786	-0.15861	10.83948	1.02E-20	3.88E-20	36.22052	up
REACTOM	0.600545	-0.02212	16.17131	4.69E-35	7.47E-34	69.09336	up
GOCC_NU	0.600522	0.146391	13.2609	2.77E-27	1.82E-26	51.26614	up
REACTOM	-0.60023	-0.03316	-18.4841	5.48E-41	2.36E-39	82.70015	down
LAKE_ADU	-0.60018	-0.13371	-17.0266	2.80E-37	6.32E-36	74.19388	down
WU_SILEN	-0.60017	0.027014	-18.4971	5.09E-41	2.20E-39	82.77436	down
REACTOM	-0.60016	-0.11346	-13.8106	9.04E-29	6.92E-28	54.67468	down
BOYAULT_	-0.60004	0.134483	-11.9982	7.42E-24	3.60E-23	43.40882	down
GOMF_TR/	-0.60002	-0.00791	-9.80182	6.11E-18	1.91E-17	29.86808	down
GOMF_TR/	-0.59988	0.009877	-14.8361	1.59E-31	1.65E-30	60.99093	down
GOBP_PYR	0.599848	0.123596	11.72596	4.07E-23	1.87E-22	41.71511	up
HP_RECUR	-0.59983	0.117302	-13.9987	2.81E-29	2.26E-28	55.83775	down
REACTOM	0.599689	-0.01945	14.57607	7.91E-31	7.52E-30	59.39563	up
HP_PROFC	-0.5996	-0.01265	-16.2079	3.76E-35	6.07E-34	69.31305	down
REACTOM	-0.59949	0.166259	-11.7399	3.73E-23	1.72E-22	41.80157	down
ZSCAN26_	0.599369	-0.13018	11.00089	3.74E-21	1.47E-20	37.21766	up
GOBP_PEP	0.599363	-0.02777	14.04207	2.15E-29	1.75E-28	56.10598	up
GOBP_PRC	0.59923	-0.05366	13.51359	5.74E-28	4.03E-27	52.83458	up
GOBP_CEL	0.599182	-0.14623	13.35138	1.58E-27	1.06E-26	51.82804	up
GOBP_FAT	-0.59899	0.18728	-9.60778	1.99E-17	6.00E-17	28.69497	down
BROWNE_	-0.59884	0.116872	-16.7563	1.40E-36	2.86E-35	72.59038	down

REACTOM	0.59868	0.043878	17.11372	1.67E-37	3.88E-36	74.70927	up
PALOMER(	-0.59859	-0.0679	-9.07039	5.11E-16	1.40E-15	25.47845	down
GOBP_NE(	-0.59855	0.028829	-19.4171	2.64E-43	1.79E-41	88.01181	down
JU_AGING_	-0.59852	0.053379	-11.1751	1.27E-21	5.16E-21	38.29576	down
REACTOM	0.598394	0.199938	14.35999	3.00E-30	2.67E-29	58.06668	up
GOBP_STR	-0.59817	0.168585	-10.6858	2.65E-20	9.75E-20	35.27289	down
REACTOM	-0.59816	0.031927	-15.4183	4.51E-33	5.60E-32	64.54272	down
chr16q13	-0.5981	0.106195	-11.8656	1.70E-23	8.02E-23	42.58352	down
chr3p13	-0.59785	-0.04302	-12.549	2.37E-25	1.30E-24	46.83801	down
KIM_LIVER_	-0.59784	0.162435	-9.88232	3.74E-18	1.18E-17	30.35638	down
HP_ASYMI	-0.59778	-0.10584	-12.715	8.38E-26	4.81E-25	47.87145	down
WIEMANN	-0.59765	0.123285	-11.3613	3.96E-22	1.68E-21	39.45038	down
GOBP_PRC	0.59734	-0.03366	14.0092	2.63E-29	2.12E-28	55.9029	up
GOBP_DRU	-0.59729	-0.01943	-11.7957	2.63E-23	1.23E-22	42.14866	down
GOBP_GLY	-0.59711	0.175507	-10.6402	3.51E-20	1.28E-19	34.9919	down
WP_FARNI	-0.59708	0.123684	-10.862	8.87E-21	3.38E-20	36.35976	down
GOMF_NU	-0.59707	0.150624	-10.4689	1.01E-19	3.59E-19	33.939	down
BIOCARTA	0.596923	-0.05279	15.04082	4.54E-32	4.98E-31	62.24278	up
REACTOM	0.596909	-0.05326	15.36214	6.35E-33	7.73E-32	64.20142	up
MORF_GSI	0.596764	0.014991	14.80385	1.94E-31	1.99E-30	60.79305	up
BURTON_A	-0.59664	0.080488	-13.8017	9.55E-29	7.30E-28	54.61962	down
GOBP_RIB(	0.596565	0.192068	12.39277	6.29E-25	3.33E-24	45.86517	up
KANG_DO)	-0.59654	-0.13619	-14.7137	3.39E-31	3.36E-30	60.24057	down
GOBP_PHA	-0.59653	0.061272	-18.0636	6.28E-40	2.20E-38	80.27095	down
GOMF_LO'	-0.59635	-0.00207	-15.1878	1.84E-32	2.12E-31	63.13987	down
GOCC_REF	0.596132	-0.02848	14.98283	6.48E-32	7.00E-31	61.88844	up
GSE13547_	0.596022	0.006476	17.61005	8.92E-39	2.55E-37	77.62833	up
GOBP_POS	-0.59597	0.072947	-11.3384	4.57E-22	1.93E-21	39.30812	down
GOBP_PH(	0.595901	0.027829	12.72396	7.92E-26	4.56E-25	47.92707	up
GENTILE_U	-0.59579	-0.13462	-17.1375	1.45E-37	3.41E-36	74.84961	down
chr4q31	-0.59577	0.028283	-15.5198	2.43E-33	3.12E-32	65.15906	down
GOBP_EST.	0.595748	0.254258	11.92077	1.20E-23	5.75E-23	42.92683	up
AIZARANI_	-0.59569	0.040721	-13.0323	1.15E-26	7.19E-26	49.84561	down
REACTOM	0.595531	0.052707	15.70479	7.89E-34	1.08E-32	66.28026	up
GOBP_GAI	0.595401	-0.01693	10.69153	2.56E-20	9.41E-20	35.30822	up
GOBP_NE(	-0.59521	-0.04679	-12.7677	6.03E-26	3.51E-25	48.199	down
GOBP_RRI	0.595201	0.092453	13.29069	2.30E-27	1.52E-26	51.45118	up
FAN_EMBF	-0.59517	0.064045	-11.6888	5.13E-23	2.33E-22	41.48394	down
REACTOM	0.594951	0.078525	16.06642	8.82E-35	1.37E-33	68.46271	up
HARALAM	-0.59492	-0.05466	-13.0973	7.69E-27	4.87E-26	50.24934	down
GOBP_POS	-0.59479	0.075866	-17.1206	1.60E-37	3.74E-36	74.74992	down
HP_THYRC	-0.5947	-0.05899	-14.1345	1.21E-29	1.01E-28	56.67679	down
HP_ABNOI	-0.5946	0.007787	-12.3443	8.51E-25	4.47E-24	45.56358	down
HP_ABNOI	-0.59428	-0.01208	-21.6811	9.92E-49	2.94E-46	100.4467	down
ZHAN_MU	0.594238	-0.0242	14.93466	8.70E-32	9.29E-31	61.59389	up
GOCC_ME:	-0.5942	-0.01084	-16.409	1.12E-35	1.98E-34	70.51845	down
GOBP_MIT	0.594037	-0.17843	11.71417	4.38E-23	2.00E-22	41.64182	up
GSE36476_	0.593718	-0.04591	18.09533	5.22E-40	1.85E-38	80.45522	up
WP_TFS_RI	-0.5936	-0.01832	-12.1978	2.13E-24	1.07E-23	44.65112	down
BIOCARTA	0.59354	-0.20006	14.24465	6.13E-30	5.26E-29	57.35605	up
MOLENAA	0.593288	-0.16371	12.43449	4.84E-25	2.59E-24	46.12492	up
WP_PHYSI(	-0.59307	0.033933	-19.22	8.09E-43	4.98E-41	86.89851	down
HP_ELEVA	-0.59303	-0.11889	-13.3239	1.87E-27	1.25E-26	51.6575	down
REACTOM	0.592858	-0.22059	11.96102	9.36E-24	4.51E-23	43.17732	up
GOMF_ALI	-0.5926	-0.08618	-16.4637	8.07E-36	1.46E-34	70.84556	down
LAKE_ADU	-0.59259	-0.14476	-15.2824	1.03E-32	1.22E-31	63.71614	down
GOMF_PH(	-0.59256	0.08394	-11.8174	2.30E-23	1.07E-22	42.28358	down
GOBP_ENE	-0.59244	0.2235	-7.60972	2.52E-12	5.48E-12	17.06384	down

KEGG_RETI	-0.59215	0.114463	-13.2047	3.93E-27	2.55E-26	50.91727	down
GOBP_MID	-0.59215	0.047638	-14.0768	1.73E-29	1.42E-28	56.32044	down
GOBP_REG	-0.592	-0.02445	-11.9743	8.61E-24	4.16E-23	43.26009	down
HP_UVEAL	-0.59193	0.00191	-12.0481	5.43E-24	2.66E-23	43.71904	down
GOBP_MH	-0.59193	0.058102	-13.8559	6.82E-29	5.28E-28	54.95526	down
ANDERSEN	-0.59193	0.170345	-10.0771	1.13E-18	3.72E-18	31.54133	down
SOTIRIOU_	-0.59187	-0.02698	-18.5095	4.74E-41	2.06E-39	82.8457	down
HP_JUVEN	-0.59182	-0.27726	-10.7551	1.72E-20	6.44E-20	35.70016	down
REACTOM	0.591809	-0.07883	12.62173	1.50E-25	8.41E-25	47.2907	up
GOBP_CEL	-0.59173	-0.11423	-11.1444	1.53E-21	6.19E-21	38.10573	down
GOBP_SPII	0.591692	-0.1668	13.3419	1.67E-27	1.12E-26	51.76918	up
GOMF_LA(	-0.59163	0.044578	-12.3855	6.58E-25	3.48E-24	45.81981	down
HP_INTEST	-0.59155	-0.05648	-12.5298	2.67E-25	1.46E-24	46.71821	down
REACTOM	0.591535	-0.01934	17.46651	2.07E-38	5.63E-37	76.78697	up
WP_EICOS	-0.5915	0.106696	-17.434	2.51E-38	6.71E-37	76.59614	down
WONG_EM	0.591368	0.014036	18.88796	5.38E-42	2.77E-40	85.01238	up
GOBP_COI	-0.59135	-0.07162	-12.5658	2.13E-25	1.18E-24	46.94259	down
WP_VITAM	-0.59132	-0.01589	-18.1024	5.01E-40	1.79E-38	80.49604	down
GOMF_EN(	-0.59132	0.19053	-9.00071	7.75E-16	2.09E-15	25.06528	down
GOBP_REG	0.591181	0.00964	17.45837	2.18E-38	5.90E-37	76.73923	up
GOBP_REG	-0.59107	-0.07256	-10.459	1.08E-19	3.80E-19	33.87842	down
GOCC_PLA	-0.59102	0.203644	-12.702	9.09E-26	5.21E-25	47.79019	down
SU_LIVER	-0.59096	0.175051	-11.1591	1.40E-21	5.67E-21	38.19664	down
GOBP_PRC	0.59093	0.146471	13.53494	5.02E-28	3.55E-27	52.96699	up
MIR5705	-0.59073	-0.01292	-10.9651	4.68E-21	1.82E-20	36.99621	down
GOBP_NEC	-0.59066	-0.01893	-18.8321	7.41E-42	3.72E-40	84.6935	down
GOMF_RN.	0.590564	-0.074	12.76376	6.18E-26	3.59E-25	48.17476	up
GOBP_POS	-0.59046	0.047162	-25.3828	5.31E-57	1.90E-53	119.3746	down
GOCC_PRC	0.590358	0.063533	11.59771	9.07E-23	4.04E-22	40.91797	up
GOBP_DRU	-0.59033	0.094182	-13.9228	4.50E-29	3.56E-28	55.36896	down
SHIPP_DLE	0.590284	0.000364	14.42419	2.02E-30	1.83E-29	58.46184	up
GOBP_BEN	-0.59024	0.162301	-12.7375	7.28E-26	4.20E-25	48.01105	down
GOBP_POS	0.590173	-0.01297	12.77712	5.68E-26	3.31E-25	48.25789	up
WP_DNA_I	0.589504	-0.11667	11.97688	8.48E-24	4.09E-23	43.27597	up
HP_PATCH	-0.58949	-0.05428	-10.2506	3.90E-19	1.32E-18	32.601	down
MIR154_3F	-0.58936	-0.11091	-22.7383	3.62E-51	1.95E-48	106.0292	down
HP_SHOUI	-0.58934	-0.01187	-9.45546	5.02E-17	1.47E-16	27.77815	down
GOBP_KET	-0.58929	0.116491	-13.5543	4.45E-28	3.15E-27	53.08728	down
BIOCARTA	-0.58918	-0.0386	-20.1081	5.45E-45	5.53E-43	91.8762	down
GOBP_POS	-0.5891	-0.11714	-9.82625	5.26E-18	1.65E-17	30.0162	down
GOBP_EST.	-0.58905	0.150474	-11.4388	2.45E-22	1.05E-21	39.93097	down
MCBRYAN	-0.58897	0.049087	-9.71782	1.02E-17	3.14E-17	29.35956	down
MIKKELSEI	-0.58888	0.060629	-9.89792	3.40E-18	1.08E-17	30.45114	down
HP_TAPER	0.588857	-0.0963	8.379297	3.05E-14	7.45E-14	21.42799	up
MORF_UBI	0.588837	0.104718	15.50097	2.73E-33	3.47E-32	65.04491	up
GOBP_ATT	0.588728	-0.1356	11.51336	1.54E-22	6.72E-22	40.39398	up
HP_HYPER	-0.58866	0.187244	-10.1845	5.86E-19	1.96E-18	32.19683	down
HOSHIDA_	-0.58851	0.165987	-11.3982	3.15E-22	1.35E-21	39.679	down
AMIT_EGF_	-0.58851	0.084813	-17.0411	2.57E-37	5.85E-36	74.27968	down
WEST_ADF	0.588472	-0.02469	17.71931	4.69E-39	1.41E-37	78.26721	up
HP_LOW_/	-0.58837	0.019069	-10.2328	4.35E-19	1.47E-18	32.49202	down
WEST_ADF	0.588143	-0.10568	14.6426	5.25E-31	5.10E-30	59.80421	up
GOMF_HIS	0.588123	-0.17258	12.44494	4.54E-25	2.43E-24	46.18998	up
GOBP_POS	0.588095	0.007738	12.04814	5.43E-24	2.66E-23	43.71954	up
GOBP_B_C	-0.58794	-0.03305	-9.47919	4.35E-17	1.28E-16	27.92073	down
GSE36476_	0.587912	-0.04788	17.29956	5.56E-38	1.39E-36	75.80547	up
MIR590_5F	-0.58775	-0.08034	-23.5508	5.33E-53	5.49E-50	110.2225	down
TIAN_BHLI	-0.58753	0.06303	-15.3174	8.35E-33	9.99E-32	63.92932	down

WP_PREGN	-0.58752	0.038207	-14.8238	1.72E-31	1.77E-30	60.91515	down
MIR4636	-0.5875	-0.07905	-12.462	4.08E-25	2.19E-24	46.29617	down
GOCC_SM	-0.5872	0.111004	-10.7383	1.91E-20	7.13E-20	35.59631	down
VECCHI_G/	-0.58714	0.039052	-23.7553	1.87E-53	2.62E-50	111.2648	down
BIOCARTA	-0.58711	0.069415	-12.4111	5.61E-25	2.98E-24	45.97914	down
GOBP_NEC	-0.58707	0.01924	-13.7444	1.36E-28	1.03E-27	54.26522	down
ZHAN_MU	-0.58704	-0.0299	-20.0311	8.38E-45	8.27E-43	91.44869	down
LY_AGING_	0.586951	-0.08741	15.12352	2.73E-32	3.09E-31	62.74768	up
DUTERTRE	0.586688	-0.12939	12.88751	2.85E-26	1.71E-25	48.94479	up
REACTOM	-0.58647	0.159497	-11.4522	2.25E-22	9.71E-22	40.0143	down
GOBP_CEL	-0.58632	-0.0938	-13.1463	5.66E-27	3.62E-26	50.55381	down
MODULE_3	0.58629	-0.09665	19.0494	2.14E-42	1.19E-40	85.93115	up
REACTOM	0.586247	0.212175	13.85897	6.69E-29	5.19E-28	54.97411	up
GOMF_MH	-0.58596	0.131521	-9.53649	3.07E-17	9.13E-17	28.26542	down
GOBP_BUD	-0.58595	-0.03543	-11.3781	3.57E-22	1.52E-21	39.55412	down
HP_HYPOF	0.585726	-0.02869	16.62931	2.99E-36	5.78E-35	71.83419	up
GOCC_EUK	0.585436	0.083538	9.476905	4.41E-17	1.30E-16	27.907	up
WP_BIOM/	-0.58511	0.214161	-9.15012	3.17E-16	8.79E-16	25.95236	down
GOMF_RET	-0.58494	0.100076	-14.9468	8.08E-32	8.65E-31	61.66835	down
GOBP_POS	0.58483	-0.04254	9.401746	6.95E-17	2.02E-16	27.45578	up
GOCC_CYT	0.584782	0.288837	10.9724	4.47E-21	1.74E-20	37.04155	up
GOMF_RN	0.584747	-0.08915	10.12418	8.48E-19	2.81E-18	31.82864	up
MODULE_5	-0.58466	0.020034	-12.3051	1.09E-24	5.66E-24	45.31937	down
REACTOM	0.584607	-0.11749	17.33053	4.63E-38	1.17E-36	75.98779	up
REACTOM	-0.58446	0.169977	-9.57608	2.42E-17	7.23E-17	28.50388	down
GOMF_EXC	0.584461	-0.06236	16.21693	3.56E-35	5.78E-34	69.36732	up
REACTOM	-0.58431	0.193922	-10.5206	7.37E-20	2.63E-19	34.25627	down
GOBP_NEC	-0.58431	-0.13249	-10.1597	6.82E-19	2.27E-18	32.04543	down
GOBP_GAS	-0.58414	0.026488	-13.4244	9.99E-28	6.83E-27	52.28152	down
CAIRO_HE	-0.58405	0.071358	-14.3999	2.35E-30	2.12E-29	58.31233	down
LAKE_ADU	-0.58397	-0.01269	-21.2746	8.91E-48	2.16E-45	98.26242	down
HP_THIRD_	-0.58394	-0.07154	-9.67524	1.32E-17	4.03E-17	29.10219	down
TANG_SEN	0.583805	-0.16293	12.19987	2.10E-24	1.06E-23	44.66408	up
GOBP_REG	-0.58378	0.070752	-16.2121	3.66E-35	5.93E-34	69.33853	down
AIZARANI_	-0.58346	0.235367	-10.2939	2.99E-19	1.02E-18	32.86594	down
GOMF_CA	0.583388	0.031129	14.90878	1.02E-31	1.08E-30	61.43551	up
MODULE_2	0.583282	0.04235	12.68519	1.01E-25	5.75E-25	47.68571	up
GOBP_NEC	-0.58328	0.098972	-11.076	2.35E-21	9.36E-21	37.68207	down
GOBP_NEC	-0.58322	-0.15681	-14.2036	7.90E-30	6.70E-29	57.10267	down
GNF2_UBE	0.58317	0.141369	12.82308	4.26E-26	2.52E-25	48.54389	up
GOBP_POS	-0.58311	0.024382	-10.9404	5.45E-21	2.11E-20	36.84374	down
REACTOM	-0.58311	0.058083	-11.5768	1.03E-22	4.58E-22	40.78825	down
TRAVAGLII	-0.5831	0.020395	-13.5597	4.30E-28	3.06E-27	53.12078	down
REACTOM	-0.58304	0.152737	-10.9622	4.76E-21	1.85E-20	36.97834	down
GOBP_REG	0.583028	-0.11068	16.20607	3.80E-35	6.13E-34	69.30211	up
HP_DEEP_\	-0.58288	0.030576	-14.6927	3.85E-31	3.80E-30	60.11146	down
GSE21063_	0.582865	-0.06787	15.86454	2.99E-34	4.33E-33	67.24585	up
BOYAULT_	0.582635	-0.15223	12.81411	4.51E-26	2.66E-25	48.48809	up
LAKE_ADU	-0.58262	-0.08408	-22.8033	2.57E-51	1.60E-48	106.368	down
HP_MENO	-0.58262	0.152713	-10.7652	1.62E-20	6.06E-20	35.76219	down
HP_NEURC	0.582601	0.081007	11.09637	2.07E-21	8.28E-21	37.80837	up
LOPEZ_ME	-0.58256	0.095326	-11.4694	2.02E-22	8.76E-22	40.12104	down
GOBP_NEC	-0.58255	0.139705	-9.32776	1.09E-16	3.12E-16	27.01252	down
AIZARANI_	-0.58247	0.23835	-10.1335	8.01E-19	2.66E-18	31.88556	down
QIU_PBMC	-0.58239	0.107841	-13.429	9.71E-28	6.64E-27	52.31012	down
GOBP_NEC	-0.58223	-0.01984	-16.9994	3.29E-37	7.34E-36	74.03307	down
GOMF_OX	-0.58208	0.055521	-18.2028	2.79E-40	1.06E-38	81.07749	down
WP_MAMI	-0.58202	-0.04463	-18.5017	4.95E-41	2.14E-39	82.80113	down

WP_COPPI	-0.582	0.023493	-19.9051	1.70E-44	1.54E-42	90.74721	down
BENPORAT	0.581987	-0.06507	11.81272	2.37E-23	1.10E-22	42.25466	up
GOMF_PH	-0.58195	-0.19276	-12.644	1.31E-25	7.36E-25	47.42923	down
DAUER_ST	-0.58187	0.049229	-14.6373	5.42E-31	5.26E-30	59.77177	down
LAKE_ADU	-0.58186	-0.09335	-23.3749	1.32E-52	1.15E-49	109.3221	down
HP_SUBDU	-0.58176	0.103895	-9.1451	3.26E-16	9.05E-16	25.92246	down
HERNAND	-0.58171	0.006803	-16.458	8.35E-36	1.51E-34	70.8113	down
HOWARD_	-0.58164	0.185313	-10.0099	1.71E-18	5.55E-18	31.13201	down
GOMF_IRC	-0.58158	0.079133	-13.8398	7.54E-29	5.81E-28	54.85555	down
GOBP_POS	-0.58155	-0.08109	-11.0938	2.10E-21	8.41E-21	37.79232	down
GOCC_CLA	0.581434	-0.1137	13.20584	3.91E-27	2.53E-26	50.92409	up
WP_ESTRC	-0.58135	0.103389	-11.1267	1.71E-21	6.89E-21	37.99598	down
GOMF_CY	-0.58132	-0.04708	-15.351	6.80E-33	8.25E-32	64.1335	down
GOBP_RIB	0.581286	0.108427	13.46518	7.75E-28	5.37E-27	52.53432	up
MORF_SAF	0.581246	0.100048	13.74344	1.37E-28	1.03E-27	54.25909	up
GOCC_STE	-0.58117	-0.03476	-9.33304	1.05E-16	3.02E-16	27.04413	down
HP_ABNOI	-0.58103	0.126639	-9.61611	1.89E-17	5.71E-17	28.7452	down
GOMF_AC	-0.58095	0.08685	-13.7639	1.21E-28	9.13E-28	54.38564	down
HP_NARRC	-0.58093	0.055331	-13.2388	3.18E-27	2.08E-26	51.12895	down
GOBP_NEC	0.580864	0.141078	12.26169	1.43E-24	7.32E-24	45.04896	up
GOBP_SER	-0.58067	0.174119	-9.4532	5.09E-17	1.49E-16	27.76459	down
GOBP_PRC	-0.58062	0.153821	-12.251	1.53E-24	7.82E-24	44.98214	down
GOBP_MIN	-0.58061	0.151778	-12.9338	2.13E-26	1.30E-25	49.23271	down
GOCC_GAI	0.580595	-0.04752	11.34939	4.27E-22	1.80E-21	39.37622	up
KYNG_NOI	-0.5804	-0.05203	-15.8766	2.78E-34	4.04E-33	67.31852	down
WP_PLATE	-0.58009	0.071038	-10.688	2.61E-20	9.61E-20	35.28669	down
MODULE_4	-0.58002	0.111411	-11.4888	1.79E-22	7.79E-22	40.24156	down
HONMA_D	0.579995	0.084715	14.63389	5.54E-31	5.36E-30	59.75074	up
GOBP_TRN	0.57996	-0.01764	13.64574	2.52E-28	1.84E-27	53.65388	up
GOMF_GL	-0.57993	0.163373	-10.835	1.05E-20	3.98E-20	36.19299	down
ASXL2_TAI	0.579882	-0.08965	18.40192	8.81E-41	3.65E-39	82.2269	up
HP_KETON	-0.57988	0.156726	-13.5545	4.45E-28	3.15E-27	53.08829	down
BIOCARTA	-0.57979	0.000781	-10.1982	5.38E-19	1.81E-18	32.28081	down
GOMF_AR	-0.5797	0.142187	-10.2875	3.10E-19	1.06E-18	32.8271	down
MIR487A_3	-0.57965	-0.10753	-22.5535	9.56E-51	4.90E-48	105.0635	down
HP_ADEN	0.579469	0.235328	13.13077	6.24E-27	3.97E-26	50.45758	up
GOCC_ARI	0.579389	0.031193	10.5112	7.81E-20	2.78E-19	34.19876	up
GOBP_TRA	-0.57932	-0.20602	-12.6663	1.14E-25	6.44E-25	47.56835	down
HP_SPONT	-0.57908	-0.08084	-11.6328	7.28E-23	3.27E-22	41.13631	down
GOCC_ORI	0.578943	-0.12561	11.17302	1.28E-21	5.22E-21	38.28294	up
STEIN_EST	-0.57888	0.050147	-17.0906	1.91E-37	4.43E-36	74.57285	down
GOCC_OLI	0.578857	0.059584	10.42394	1.34E-19	4.70E-19	33.66299	up
GOBP_NEC	-0.57879	0.148095	-10.4321	1.27E-19	4.47E-19	33.7133	down
MODULE_2	0.578634	0.099381	11.09094	2.14E-21	8.55E-21	37.77477	up
BIOCARTA	-0.57854	0.096188	-11.2758	6.76E-22	2.80E-21	38.91961	down
HP_ABNOI	-0.5785	-0.03294	-12.4401	4.68E-25	2.50E-24	46.15988	down
HP_PERINI	-0.57846	0.161252	-9.18285	2.60E-16	7.27E-16	26.14723	down
GOBP_SYN	0.578424	-0.05632	17.44199	2.40E-38	6.44E-37	76.64304	up
GOBP_NEF	-0.57838	-0.0676	-15.7099	7.65E-34	1.05E-32	66.31107	down
SARRIO_EI	0.578372	-0.10772	14.39871	2.36E-30	2.13E-29	58.30504	up
GOCC_SUI	-0.57836	-0.00507	-10.9601	4.83E-21	1.88E-20	36.96541	down
WP_DEGR	0.578163	-0.03505	10.92016	6.18E-21	2.39E-20	36.7187	up
IKEDA_MIF	-0.57814	-0.13455	-19.4392	2.33E-43	1.60E-41	88.13626	down
CHANG_P	-0.5781	0.101866	-13.2985	2.19E-27	1.45E-26	51.49942	down
GOMF_HY	-0.57808	0.136856	-10.2916	3.03E-19	1.04E-18	32.85197	down
GSE24634_	0.578033	0.020411	19.88913	1.85E-44	1.66E-42	90.65819	up
PID_IL5_PA	-0.57798	-0.06567	-15.3137	8.54E-33	1.02E-31	63.90676	down
GOMF_SO	-0.57796	-0.00305	-15.6251	1.28E-33	1.69E-32	65.79775	down

GOBP_MA	-0.57793	0.037537	-19.1452	1.24E-42	7.23E-41	86.47508	down
GOCC_RES	-0.57773	0.113537	-7.80186	8.51E-13	1.90E-12	18.13683	down
GOMF_SU(	-0.57773	0.113537	-7.80186	8.51E-13	1.90E-12	18.13683	down
GNF2_MLH	0.577716	-0.15742	11.93845	1.08E-23	5.16E-23	43.03686	up
GNF2_CD1	-0.5776	0.155593	-11.0014	3.73E-21	1.46E-20	37.22055	down
MODULE_:	-0.57747	0.018976	-13.6781	2.06E-28	1.52E-27	53.85417	down
GOBP_POS	0.577443	-0.24374	12.21868	1.87E-24	9.49E-24	44.78117	up
GOBP_NAT	-0.57738	0.091935	-9.84197	4.78E-18	1.50E-17	30.11152	down
FLORIO_NI	0.577371	-0.15599	11.84963	1.88E-23	8.83E-23	42.48426	up
GOCC_INC	0.577275	-0.07656	17.95681	1.17E-39	3.90E-38	79.6511	up
GOCC_CILI	0.576984	-0.14807	11.33672	4.62E-22	1.95E-21	39.29763	up
HP_ABNOI	-0.5768	0.046979	-11.41	2.93E-22	1.25E-21	39.75237	down
GOBP_LAB	-0.57679	0.032367	-16.3283	1.82E-35	3.11E-34	70.03538	down
GCM_AQP	-0.5767	-0.1017	-23.1502	4.22E-52	3.17E-49	108.1654	down
GOBP_NEC	-0.57667	-0.12787	-9.77809	7.06E-18	2.20E-17	29.72431	down
LEE_METAS	0.576481	-0.03261	14.07589	1.74E-29	1.43E-28	56.31485	up
TRAVAGLII	0.576457	0.066108	20.45992	7.73E-46	9.98E-44	93.82059	up
GOBP_GLC	-0.57645	-0.07976	-10.1588	6.86E-19	2.28E-18	32.03979	down
GOCC_CAI	-0.5764	0.026547	-9.63035	1.74E-17	5.25E-17	28.83114	down
GOBP_RES	0.5763	0.025351	9.84568	4.67E-18	1.47E-17	30.13403	up
GOBP_CEL	0.5763	0.025351	9.84568	4.67E-18	1.47E-17	30.13403	up
HP_HEPAT	-0.5762	0.160776	-11.0845	2.23E-21	8.89E-21	37.73473	down
NAKAYA_F	-0.57601	-0.04821	-19.136	1.30E-42	7.61E-41	86.42275	down
MORF_PCI	0.575914	0.022388	15.16202	2.16E-32	2.47E-31	62.98254	up
GOBP_REG	-0.57586	0.002295	-10.5284	7.02E-20	2.51E-19	34.30453	down
chr8p12	-0.57583	0.038047	-11.0398	2.94E-21	1.16E-20	37.45802	down
HP_ARTER	-0.57579	0.022722	-17.5075	1.63E-38	4.49E-37	77.02761	down
GOBP_LEU	-0.57568	0.10607	-11.0596	2.60E-21	1.03E-20	37.58094	down
GOBP_STR	0.575568	-0.21015	8.786158	2.78E-15	7.27E-15	23.79955	up
REACTOM	0.575459	-0.0773	18.53741	4.03E-41	1.76E-39	83.00626	up
HP_ACUTE	0.575373	-0.04799	10.14499	7.46E-19	2.48E-18	31.95567	up
MIR3161	-0.57535	-0.06417	-25.4645	3.56E-57	1.52E-53	119.773	down
MIR5694	-0.57523	-0.08131	-20.4958	6.34E-46	8.29E-44	94.01789	down
GOBP_L_SI	-0.57489	0.17477	-8.12338	1.35E-13	3.16E-13	19.95771	down
GOBP_L_A	-0.57482	0.045061	-12.5217	2.81E-25	1.53E-24	46.6677	down
GOBP_REG	-0.57479	0.150086	-11.8722	1.63E-23	7.71E-23	42.62467	down
SCHOEN_I	-0.57463	0.045831	-14.523	1.10E-30	1.02E-29	59.0698	down
BOYAULT_	-0.57458	0.078666	-12.9429	2.02E-26	1.23E-25	49.28952	down
GOBP_RRI	0.574572	0.110426	10.32874	2.41E-19	8.30E-19	33.0794	up
REACTOM	-0.57452	0.026387	-12.8999	2.64E-26	1.59E-25	49.02212	down
HP_INTERS	0.57434	-0.08676	10.06787	1.20E-18	3.93E-18	31.48524	up
GABRIELY_	-0.57423	-0.18031	-16.7981	1.09E-36	2.27E-35	72.83876	down
REACTOM	0.574217	-0.15093	11.07317	2.39E-21	9.52E-21	37.66477	up
GOBP_TUE	0.573993	-0.0043	14.35775	3.04E-30	2.71E-29	58.05284	up
REACTOM	0.573907	-0.07722	13.66059	2.30E-28	1.68E-27	53.74591	up
HP_FIBRO/	-0.57386	-0.03961	-13.5317	5.12E-28	3.61E-27	52.94721	down
HAY_BONI	0.573721	-0.06462	16.34003	1.70E-35	2.92E-34	70.10551	up
KEGG_BAS	0.573656	-0.0002	17.14943	1.35E-37	3.19E-36	74.92019	up
HP_CHOR(	0.573641	-0.01852	11.72988	3.97E-23	1.82E-22	41.73947	up
GOBP_CH/	0.573437	-0.07491	11.31108	5.42E-22	2.27E-21	39.13866	up
MIR192_5F	-0.57342	-0.08571	-19.5945	9.71E-44	7.23E-42	89.0098	down
HP_ABNOI	-0.57337	0.235524	-9.67326	1.34E-17	4.07E-17	29.09024	down
PATEL_SKII	-0.57332	-0.04484	-17.881	1.82E-39	5.92E-38	79.20984	down
GOBP_NEC	-0.57327	0.057173	-14.8889	1.15E-31	1.21E-30	61.31387	down
BIOCARTA	-0.57325	-0.11272	-22.4876	1.35E-50	6.51E-48	104.7184	down
TSAI_RESP	-0.57323	-0.00643	-13.8437	7.36E-29	5.68E-28	54.87991	down
GOBP_QUI	-0.57321	0.245732	-8.26981	5.77E-14	1.39E-13	20.79682	down
BIOCARTA	-0.57313	0.081827	-16.3363	1.74E-35	2.98E-34	70.08305	down

GOBP_REG	-0.57304	0.108427	-8.71673	4.19E-15	1.09E-14	23.39209	down
GOBP_REG	-0.57289	0.035182	-12.5201	2.83E-25	1.55E-24	46.65814	down
MARTIN_N	-0.57289	0.025716	-20.5211	5.52E-46	7.48E-44	94.15694	down
ODONNEL	0.572883	-0.13987	14.06138	1.91E-29	1.56E-28	56.2252	up
GOBP_NEG	-0.57283	0.11048	-8.88914	1.51E-15	4.01E-15	24.4059	down
PEDERSEN	-0.57266	0.011104	-12.3083	1.07E-24	5.55E-24	45.33949	down
HP_INCREA	-0.57265	-0.07769	-10.5042	8.15E-20	2.90E-19	34.15597	down
GOBP_NEG	0.572583	0.070066	10.86856	8.52E-21	3.25E-20	36.39999	up
GOBP_PYR	0.57238	0.028408	14.36149	2.97E-30	2.65E-29	58.07591	up
DESCARTE	-0.57236	-0.00758	-16.4298	9.89E-36	1.76E-34	70.64261	down
ZHU_SKIL_	-0.57225	-0.05367	-15.272	1.10E-32	1.30E-31	63.65301	down
GOBP_POS	-0.57194	0.116575	-11.9186	1.22E-23	5.83E-23	42.91337	down
GNF2_NS	0.571873	0.026235	11.97297	8.69E-24	4.19E-23	43.25165	up
GOBP_RES	-0.57186	0.077227	-19.5177	1.50E-43	1.07E-41	88.57825	down
AIZARANI_	-0.57186	0.025179	-13.7849	1.06E-28	8.05E-28	54.51594	down
GOBP_RES	-0.57185	0.003186	-15.8642	3.00E-34	4.33E-33	67.24391	down
FERRARI_R	-0.57174	0.032212	-14.3781	2.68E-30	2.41E-29	58.17815	down
GOBP_POS	0.571706	-0.11354	9.635028	1.69E-17	5.11E-17	28.85936	up
LAKE_ADU	-0.57168	-0.11028	-20.5379	5.03E-46	6.90E-44	94.24944	down
GOCC_SPA	-0.57165	0.022298	-18.3528	1.17E-40	4.70E-39	81.94377	down
GOBP_RIBO	0.57164	0.173717	12.76828	6.01E-26	3.49E-25	48.20293	up
MORF_CSN	0.571414	0.067514	15.34841	6.91E-33	8.38E-32	64.11788	up
WP_SIMPL	-0.57139	-0.09565	-13.6172	3.01E-28	2.17E-27	53.47714	down
GOCC_MA	-0.57135	0.074006	-7.77813	9.74E-13	2.17E-12	18.00367	down
GOBP_NEG	-0.57127	0.124723	-10.215	4.85E-19	1.64E-18	32.38306	down
GOMF_FIB	-0.57107	0.008414	-15.0893	3.37E-32	3.77E-31	62.5386	down
REACTOM	0.57106	-0.08852	12.30211	1.11E-24	5.75E-24	45.30065	up
MYLLYKAN	0.570868	-0.08594	12.21654	1.89E-24	9.61E-24	44.76787	up
GOBP_HEA	0.570826	0.151286	9.219482	2.09E-16	5.87E-16	26.36562	up
REACTOM	0.57081	-0.2449	11.4576	2.17E-22	9.40E-22	40.04775	up
BOYAULT_	0.570559	-0.1291	14.04681	2.09E-29	1.70E-28	56.13523	up
GOMF_ALI	-0.57052	0.107932	-11.2441	8.23E-22	3.40E-21	38.72367	down
REACTOM	-0.57052	-0.02063	-10.6525	3.26E-20	1.19E-19	35.06763	down
GOBP_URI	-0.57045	0.007843	-10.5903	4.78E-20	1.73E-19	34.68523	down
WP_LTF_DA	-0.57041	-0.02135	-14.6431	5.23E-31	5.08E-30	59.80738	down
GOBP_PYR	0.570396	0.02306	16.30217	2.13E-35	3.59E-34	69.87865	up
LI_WILMS_	-0.57035	-0.04474	-9.48102	4.30E-17	1.27E-16	27.93171	down
GOBP_MIT	0.570268	-0.21732	10.98871	4.04E-21	1.58E-20	37.14238	up
GOMF_DN	0.570176	-0.16588	11.47038	2.01E-22	8.71E-22	40.12711	up
GOBP_RES	-0.57017	-0.00925	-20.3597	1.35E-45	1.55E-43	93.26839	down
GOBP_POS	-0.57012	0.176848	-9.1428	3.31E-16	9.18E-16	25.90878	down
ROETH_TE	-0.5701	0.060455	-8.61744	7.54E-15	1.91E-14	22.81134	down
KEGG_MIS	0.569964	-0.14157	13.14551	5.69E-27	3.63E-26	50.5492	up
MORF_USP	0.56994	0.022143	13.75842	1.25E-28	9.42E-28	54.35183	up
GOCC_LAR	0.569713	0.280172	11.4267	2.64E-22	1.13E-21	39.85595	up
WP_GLUCU	-0.56951	0.018992	-11.5707	1.07E-22	4.75E-22	40.75025	down
GOBP_REG	0.569479	-0.09882	9.922333	2.93E-18	9.35E-18	30.59944	up
PID_IL6_7_	-0.56923	0.002617	-20.0299	8.44E-45	8.30E-43	91.44169	down
DESCARTE	-0.56895	0.165607	-11.4057	3.01E-22	1.29E-21	39.72532	down
GOBP_SPL	0.568921	-0.00586	14.46935	1.53E-30	1.40E-29	58.73968	up
GOBP_POS	-0.56882	0.015834	-11.6246	7.67E-23	3.44E-22	41.08506	down
WP_GASTR	0.568668	-0.23998	11.65882	6.19E-23	2.80E-22	41.29776	up
GOBP_CHE	-0.56838	-0.01165	-11.1721	1.29E-21	5.25E-21	38.27748	down
GOCC_PHO	-0.56833	-0.09016	-16.793	1.13E-36	2.33E-35	72.80842	down
LY_AGING_	0.568311	-0.04369	13.04698	1.05E-26	6.59E-26	49.93664	up
LI_DCP2_B	0.568284	0.160719	14.7179	3.30E-31	3.28E-30	60.26624	up
GOCC_CYT	0.568259	0.275035	10.69284	2.54E-20	9.34E-20	35.31626	up
GOBP_ASP	-0.56822	0.143417	-10.2874	3.11E-19	1.06E-18	32.82652	down

BIOCARTA	-0.56814	-0.04936	-10.2316	4.38E-19	1.48E-18	32.48469	down
GOBP_REG	0.568138	-0.10309	10.88277	7.80E-21	2.99E-20	36.48777	up
GOMF_OX	-0.56812	0.12474	-12.0305	6.06E-24	2.96E-23	43.60959	down
HP_RIGHT_	-0.56802	-0.02658	-17.8367	2.36E-39	7.52E-38	78.95199	down
HP_DEVIAT	-0.56793	-0.24275	-9.98436	2.00E-18	6.46E-18	30.97661	down
LAKE_ADU	-0.56792	-0.11518	-21.8067	5.05E-49	1.60E-46	101.1175	down
WP_COVID	-0.56791	0.058047	-11.9713	8.78E-24	4.23E-23	43.24117	down
MODULE_4	-0.56786	0.065208	-15.8861	2.63E-34	3.83E-33	67.3758	down
REACTOM	-0.56784	-0.03255	-14.4093	2.21E-30	2.00E-29	58.36992	down
SOBOLEV_	-0.56772	0.166881	-10.1769	6.14E-19	2.05E-18	32.15047	down
GOBP_INT	-0.56765	-0.08202	-15.6598	1.04E-33	1.39E-32	66.00818	down
GOBP_REG	0.567574	0.046174	14.37702	2.70E-30	2.42E-29	58.1715	up
HP_LOSS_(	-0.5673	0.025601	-15.9397	1.90E-34	2.82E-33	67.69906	down
GOBP_CAF	-0.5672	-0.01273	-10.7043	2.36E-20	8.73E-20	35.38693	down
GOBP_HIS	0.5671	-0.05856	10.2656	3.55E-19	1.21E-18	32.69284	up
GOMF_HIS	0.5671	-0.05856	10.2656	3.55E-19	1.21E-18	32.69284	up
WANG_IM	-0.56708	-0.03946	-17.0881	1.94E-37	4.49E-36	74.55806	down
GOBP_REG	-0.56703	0.034969	-13.3888	1.25E-27	8.46E-27	52.06047	down
REACTOM	-0.56697	0.160791	-10.6259	3.84E-20	1.40E-19	34.90387	down
GOBP_REG	-0.56689	0.052249	-28.7992	5.27E-64	1.30E-59	135.3653	down
GOMF_FLA	0.566808	-0.21022	11.42177	2.72E-22	1.17E-21	39.82535	up
GOBP_ASS	0.566696	-0.26523	10.07627	1.14E-18	3.74E-18	31.53643	up
REACTOM	-0.56649	0.065607	-15.2904	9.85E-33	1.17E-31	63.76482	down
MORF_DAI	0.566439	0.066184	14.74573	2.78E-31	2.80E-30	60.43688	up
REACTOM	-0.56643	0.100928	-12.555	2.28E-25	1.26E-24	46.87498	down
GNF2_DAF	0.566422	0.162964	12.59004	1.83E-25	1.02E-24	47.0934	up
GOBP_PUF	0.566379	0.082927	10.23408	4.31E-19	1.46E-18	32.50004	up
GOBP_RIB(	0.566254	0.034584	10.55317	6.02E-20	2.16E-19	34.45675	up
GOBERT_C	0.566204	-0.11283	15.47076	3.28E-33	4.13E-32	64.86152	up
REACTOM	-0.56619	0.037612	-11.4537	2.23E-22	9.63E-22	40.02361	down
HP_MACR(	-0.56619	0.088861	-12.3195	9.94E-25	5.19E-24	45.40896	down
GOBP_VIT/	-0.56604	0.005261	-9.6913	1.20E-17	3.67E-17	29.19925	down
GOMF_PH(	-0.56602	0.005995	-11.4387	2.45E-22	1.05E-21	39.93022	down
GOBP_DEV	-0.56599	-0.03065	-11.3135	5.34E-22	2.24E-21	39.15388	down
GOBP_RES	-0.56594	0.146253	-9.38438	7.72E-17	2.23E-16	27.35166	down
ZHANG_RI	0.565857	0.065505	15.90232	2.38E-34	3.49E-33	67.47388	up
GOBP_NEC	-0.56576	0.177899	-9.87943	3.80E-18	1.21E-17	30.33887	down
GOBP_POS	-0.56567	-0.12219	-10.92	6.19E-21	2.39E-20	36.71784	down
GOMF_NEI	-0.56559	0.110108	-9.19346	2.44E-16	6.83E-16	26.21045	down
HP_BROAD	-0.56556	-0.00423	-15.5455	2.08E-33	2.69E-32	65.31541	down
BIOCARTA	-0.56521	0.031248	-14.4987	1.27E-30	1.18E-29	58.92016	down
LEE_LIVER_	-0.5649	0.120613	-13.6571	2.35E-28	1.72E-27	53.72412	down
HP_INTERN	-0.5649	-0.10095	-12.5959	1.76E-25	9.81E-25	47.12974	down
GOBP_REG	-0.56487	0.013196	-12.6686	1.12E-25	6.35E-25	47.58261	down
REACTOM	0.564859	-0.0476	17.42228	2.69E-38	7.17E-37	76.52726	up
REACTOM	0.56483	-0.02579	16.22417	3.41E-35	5.54E-34	69.41077	up
GOBP_FRU	-0.56451	0.207737	-7.26943	1.68E-11	3.47E-11	15.19389	down
REACTOM	-0.56451	0.207737	-7.26943	1.68E-11	3.47E-11	15.19389	down
WP_IL1_AN	-0.5643	0.026345	-13.0454	1.06E-26	6.65E-26	49.9269	down
MORI_PRE	0.564171	-0.06155	15.97373	1.54E-34	2.32E-33	67.90449	up
SASSON_F	-0.56416	0.129998	-12.4968	3.28E-25	1.78E-24	46.51271	down
GOMF_TAI	0.564158	-0.06174	8.213996	7.98E-14	1.90E-13	20.47629	up
HP_CEREBI	-0.56403	-0.00273	-10.3682	1.89E-19	6.56E-19	33.32121	down
GARGALO\	-0.56381	-0.06641	-16.7213	1.73E-36	3.45E-35	72.38221	down
GOBP_POS	-0.56373	0.01336	-18.7898	9.44E-42	4.64E-40	84.45202	down
GOBP_AM	-0.5637	0.142153	-13.2419	3.12E-27	2.04E-26	51.14794	down
GOBP_REG	0.563698	-0.21742	12.69015	9.79E-26	5.58E-25	47.71659	up
HP_PULM(	-0.56359	0.032177	-17.847	2.22E-39	7.11E-38	79.01188	down

LANDIS_EF	-0.5635	0.050757	-16.8604	7.53E-37	1.60E-35	73.20876	down
GOBP_POS	-0.56345	-0.01345	-12.5107	3.01E-25	1.63E-24	46.59956	down
FOURNIER	0.563423	-0.13503	11.89084	1.45E-23	6.89E-23	42.74065	up
VECCHI_G	0.563411	-0.04428	18.55402	3.66E-41	1.62E-39	83.10165	up
HP_EPISCL	-0.56329	0.13086	-9.71052	1.07E-17	3.28E-17	29.31544	down
HP_DECRE	-0.56327	0.220669	-8.14996	1.16E-13	2.72E-13	20.10957	down
GOBP_POS	0.56325	0.293549	10.06995	1.18E-18	3.88E-18	31.49788	up
MORF_MA	0.563155	0.095992	13.53358	5.06E-28	3.57E-27	52.95859	up
GOBP_NEG	-0.5631	0.13007	-8.71802	4.16E-15	1.08E-14	23.39967	down
GOCC_U2_	0.562967	0.071598	14.24101	6.26E-30	5.37E-29	57.33359	up
BIOCARTA	0.562681	-0.15904	8.34791	3.66E-14	8.90E-14	21.24673	up
RIZ_ERYTH	-0.56245	0.023942	-21.1558	1.70E-47	3.63E-45	97.61986	down
REACTOM	0.562288	-0.05101	18.66942	1.88E-41	8.70E-40	83.76344	up
GOBP_ETH	-0.56208	0.128194	-12.8236	4.25E-26	2.51E-25	48.54715	down
GOBP_NEG	-0.56193	-0.04612	-11.2358	8.67E-22	3.57E-21	38.6719	down
GOCC_VAC	0.561906	0.01003	15.66821	9.85E-34	1.32E-32	66.05882	up
SOX11_TA	0.5619	-0.04811	18.60636	2.71E-41	1.22E-39	83.40202	up
GOBP_REG	-0.56187	0.049842	-11.7344	3.86E-23	1.78E-22	41.7676	down
MORF_PPF	0.561845	0.119339	13.75516	1.28E-28	9.61E-28	54.33162	up
GOMF_SYN	-0.56178	-0.02726	-10.0806	1.11E-18	3.64E-18	31.5631	down
GOBP_ALP	-0.56172	0.136813	-12.0757	4.57E-24	2.25E-23	43.8908	down
GOBP_MAT	0.561233	0.078192	12.72778	7.74E-26	4.45E-25	47.95085	up
OHM_EMB	-0.56118	-0.01387	-11.8318	2.10E-23	9.84E-23	42.37363	down
SMID_BRE	-0.56104	0.054731	-10.8918	7.37E-21	2.83E-20	36.54369	down
GOCC_CAT	0.561037	-0.00673	15.96844	1.60E-34	2.39E-33	67.87259	up
HP_CEREBI	-0.56101	0.128451	-11.7494	3.51E-23	1.62E-22	41.86085	down
chr9q21	-0.56101	0.021063	-17.3451	4.25E-38	1.08E-36	76.07343	down
GOBP_TOX	-0.56086	0.004536	-14.5145	1.16E-30	1.08E-29	59.01725	down
TONKS_TA	-0.5608	0.039134	-9.14736	3.22E-16	8.94E-16	25.9359	down
GOBP_ARC	-0.56063	0.196558	-10.5811	5.07E-20	1.83E-19	34.62838	down
GOBP_NEG	-0.56051	-0.03276	-10.9906	3.99E-21	1.56E-20	37.15398	down
GOBP_NEG	-0.56046	-0.05123	-11.6067	8.57E-23	3.83E-22	40.97365	down
GOBP_PRO	-0.56043	-0.17362	-10.3443	2.19E-19	7.57E-19	33.17457	down
GOMF_L_C	-0.56039	0.013257	-11.5257	1.42E-22	6.24E-22	40.47088	down
REACTOM	0.560337	-0.08735	16.64229	2.77E-36	5.37E-35	71.91158	up
GOBP_NEG	0.560212	0.038256	13.51098	5.83E-28	4.09E-27	52.81845	up
HP_INTUSS	-0.56003	-0.02736	-12.7462	6.90E-26	3.99E-25	48.06538	down
HP_CHON	-0.56001	0.122259	-13.4385	9.15E-28	6.28E-27	52.3689	down
chr3p26	-0.55991	0.008729	-13.7401	1.40E-28	1.05E-27	54.23856	down
GSE5679_C	0.559763	-0.00578	18.37361	1.04E-40	4.20E-39	82.06376	up
GOBP_RET	-0.5597	0.071508	-17.833	2.41E-39	7.67E-38	78.93062	down
GOBP_CEL	-0.55966	0.007524	-12.3685	7.32E-25	3.86E-24	45.71394	down
GSE15930_	0.559443	0.060358	17.37827	3.49E-38	9.08E-37	76.26862	up
WP_UREA_	-0.55938	0.109846	-10.9391	5.50E-21	2.13E-20	36.83575	down
HP_ABNOI	-0.55937	-0.02655	-20.969	4.71E-47	9.26E-45	96.6059	down
SESTO_RES	-0.55935	-0.12719	-17.7735	3.42E-39	1.05E-37	78.58362	down
GOBP_UDI	-0.55929	0.067317	-10.1082	9.36E-19	3.09E-18	31.7312	down
GOMF_UD	-0.55929	0.067317	-10.1082	9.36E-19	3.09E-18	31.7312	down
ABRAHAM	-0.5592	0.043324	-17.9051	1.58E-39	5.19E-38	79.35049	down
HP_TIGER_	0.559181	0.139918	10.5534	6.01E-20	2.16E-19	34.45812	up
BIOCARTA	-0.55908	-0.22384	-10.8164	1.18E-20	4.46E-20	36.07794	down
DESCARTE	-0.55885	0.07771	-11.2957	5.97E-22	2.49E-21	39.0434	down
GOBP_SEN	0.558751	0.02877	10.71186	2.25E-20	8.34E-20	35.43348	up
REACTOM	0.558711	-0.10551	14.16401	1.01E-29	8.44E-29	56.85874	up
HP_FLAREI	-0.55859	0.009712	-9.30768	1.23E-16	3.50E-16	26.89243	down
HP_ABNOI	-0.5584	0.077886	-11.9336	1.11E-23	5.32E-23	43.00685	down
HP_EPISOI	0.558378	-0.16821	9.949173	2.48E-18	7.97E-18	30.76259	up
LAKE_ADU	-0.55828	-0.09141	-23.3609	1.42E-52	1.20E-49	109.25	down

GOBP_NEC	-0.55812	-0.18549	-9.801	6.14E-18	1.92E-17	29.86312	down
GOMF_NEI	0.558108	0.19277	12.19976	2.10E-24	1.06E-23	44.66341	up
GOBP_ETH	-0.55796	0.108024	-14.3143	3.98E-30	3.49E-29	57.78548	down
REACTOM	-0.5579	0.197033	-8.48369	1.66E-14	4.11E-14	22.03267	down
GOBP_REG	-0.55789	0.039569	-12.2911	1.19E-24	6.15E-24	45.23199	down
MIR215_5F	-0.55777	-0.09212	-18.584	3.08E-41	1.38E-39	83.27377	down
GOBP_POS	-0.55753	-0.05054	-20.9158	6.30E-47	1.18E-44	96.31633	down
MIR1244	-0.55753	-0.09096	-21.8059	5.07E-49	1.60E-46	101.1134	down
REACTOM	-0.55752	0.154395	-9.40219	6.93E-17	2.01E-16	27.45843	down
GOMF_MH	-0.55745	0.123085	-7.69018	1.60E-12	3.52E-12	17.51169	down
GOBP_REG	0.557314	-0.09857	12.3549	7.97E-25	4.19E-24	45.62934	up
GOBP_FEV	-0.55726	0.013719	-12.1166	3.54E-24	1.76E-23	44.14578	down
GOBP_VIR/	0.557179	0.006309	15.46921	3.31E-33	4.16E-32	64.85212	up
GOMF_PH	-0.55717	0.104352	-11.0918	2.13E-21	8.51E-21	37.77995	down
REACTOM	0.557078	0.29115	10.56482	5.60E-20	2.02E-19	34.52833	up
GSE17974_	0.557034	0.017333	20.91444	6.35E-47	1.18E-44	96.30914	up
GOBP_TRY	-0.55683	0.178703	-9.25874	1.65E-16	4.67E-16	26.59995	down
WP_SIGNA	-0.55674	-0.01336	-20.3575	1.36E-45	1.56E-43	93.2562	down
GOMF_SO	-0.55664	0.050284	-13.6224	2.91E-28	2.10E-27	53.50948	down
HP_ABNOI	-0.55658	-0.05625	-13.3011	2.16E-27	1.43E-26	51.5157	down
BURTON_/	-0.5564	0.070586	-12.5892	1.84E-25	1.02E-24	47.0884	down
MIR933	-0.55639	-0.12072	-10.432	1.27E-19	4.47E-19	33.71255	down
HP_HEMIH	-0.55638	-0.0401	-16.4715	7.70E-36	1.40E-34	70.89211	down
BIOCARTA	-0.55637	0.019508	-16.9029	5.85E-37	1.27E-35	73.46093	down
GAO_LARC	-0.55633	-0.03481	-17.002	3.24E-37	7.24E-36	74.04826	down
GOMF_SIA	-0.55631	0.094593	-10.8573	9.14E-21	3.48E-20	36.33027	down
GNF2_CD1	-0.55623	0.152193	-9.79517	6.36E-18	1.98E-17	29.8278	down
GRAHAM_	-0.55621	0.026625	-12.7713	5.89E-26	3.43E-25	48.22177	down
REACTOM	0.556143	-0.14158	12.63965	1.34E-25	7.56E-25	47.40227	up
REACTOM	-0.55611	-0.10726	-20.5385	5.01E-46	6.90E-44	94.25288	down
chr3p12	-0.55589	-0.05194	-14.078	1.72E-29	1.41E-28	56.32787	down
BIOCARTA	-0.55579	0.020833	-16.7525	1.43E-36	2.92E-35	72.5677	down
MORF_PHI	0.555739	-0.00909	18.27268	1.86E-40	7.18E-39	81.48142	up
MORF_AP2	0.555703	0.113908	13.7906	1.02E-28	7.79E-28	54.55102	up
HP_ENTER	-0.5557	0.139664	-12.1002	3.92E-24	1.94E-23	44.04338	down
HP_DECRE	-0.55569	0.217043	-9.61037	1.96E-17	5.91E-17	28.71057	down
REACTOM	-0.5556	-0.03932	-9.15753	3.03E-16	8.42E-16	25.99645	down
GOCC_RN/	0.555588	0.082118	12.71405	8.43E-26	4.84E-25	47.86538	up
REACTOM	0.555588	0.082118	12.71405	8.43E-26	4.84E-25	47.86538	up
HP_CHROI	-0.55553	0.001456	-11.2126	1.00E-21	4.11E-21	38.52801	down
HP_ACIDEI	-0.55553	0.148221	-10.7368	1.93E-20	7.19E-20	35.58697	down
TURASHVI	-0.55547	-0.10474	-11.5937	9.30E-23	4.14E-22	40.89308	down
WP_IRON_	-0.55545	0.024252	-13.0694	9.15E-27	5.75E-26	50.07634	down
GOBP_POS	-0.55545	0.115628	-8.66233	5.78E-15	1.48E-14	23.07364	down
HP_PROLC	-0.55539	-0.01245	-15.4286	4.24E-33	5.28E-32	64.60524	down
HP_ABNOI	0.555305	-0.0658	12.9341	2.13E-26	1.30E-25	49.23461	up
HP_SIRENC	-0.55514	0.001556	-11.5115	1.55E-22	6.79E-22	40.38247	down
GOBP_POS	-0.55486	0.016611	-13.2684	2.64E-27	1.74E-26	51.31255	down
GOBP_MA	-0.55478	-0.02715	-9.66124	1.44E-17	4.37E-17	29.01759	down
GOBP_POS	0.554616	-0.17411	12.63114	1.42E-25	7.94E-25	47.34928	up
GOMF_C_I	0.554582	0.167063	9.41466	6.43E-17	1.87E-16	27.53324	up
GOBP_UTF	0.554523	0.010556	11.66454	5.97E-23	2.70E-22	41.33327	up
HP_AORTI	-0.55444	-0.03702	-13.5933	3.49E-28	2.50E-27	53.32895	down
HP_ABNOI	-0.55438	-0.12707	-8.82703	2.18E-15	5.75E-15	24.03992	down
GOMF_CEI	-0.55429	-0.05961	-9.46097	4.86E-17	1.43E-16	27.81125	down
MATZUK_E	-0.5542	-0.07208	-14.8239	1.72E-31	1.77E-30	60.91589	down
GOBP_POS	-0.55419	0.049275	-12.4263	5.10E-25	2.72E-24	46.0739	down
GSE35543_	0.554099	-0.05353	17.94401	1.26E-39	4.18E-38	79.57672	up

DESCARTE	-0.55399	0.03679	-16.1338	5.87E-35	9.27E-34	68.86791	down
WP_MAMN	-0.5539	0.027175	-13.1764	4.69E-27	3.02E-26	50.74128	down
REACTOM	0.553829	-0.20454	9.94366	2.57E-18	8.23E-18	30.72907	up
GOBP_POS	-0.55365	0.080624	-10.7024	2.39E-20	8.82E-20	35.37527	down
HOEK_T_C	0.553615	-0.01901	12.51227	2.98E-25	1.62E-24	46.60919	up
GOBP_PRC	-0.55359	0.034115	-14.4214	2.05E-30	1.86E-29	58.44471	down
GOBP_ISO	-0.55354	0.247426	-8.44558	2.07E-14	5.11E-14	21.81162	down
CHEN_LVA	-0.55353	0.038358	-17.0743	2.11E-37	4.86E-36	74.4762	down
GNF2_CD3	-0.55339	0.119599	-10.7119	2.25E-20	8.34E-20	35.43346	down
HP_ABNOI	-0.55336	0.020537	-13.1778	4.65E-27	2.99E-26	50.75015	down
REACTOM	0.553356	-0.00186	16.06707	8.79E-35	1.36E-33	68.46662	up
HP_INCRE	-0.55335	0.065991	-14.9924	6.11E-32	6.62E-31	61.94689	down
HALLMARI	0.553285	0.060636	11.58213	9.99E-23	4.44E-22	40.8212	up
WP_THE_E	0.553268	-0.06142	15.37175	5.99E-33	7.33E-32	64.25985	up
GOBP_GTF	0.553119	0.057586	11.26408	7.27E-22	3.01E-21	38.84724	up
GOBP_NEC	-0.55298	0.099372	-17.5774	1.08E-38	3.05E-37	77.43724	down
GOBP_REG	-0.55297	-0.02859	-13.8852	5.69E-29	4.44E-28	55.13654	down
GOBP_NEC	-0.55294	-0.0831	-10.3101	2.70E-19	9.28E-19	32.96544	down
LAKE_ADU	-0.55274	-0.06199	-21.7999	5.24E-49	1.62E-46	101.0815	down
BONOME_	0.552719	0.077009	16.076	8.33E-35	1.29E-33	68.52033	up
PHONG_TI	-0.55264	0.029011	-12.6392	1.35E-25	7.58E-25	47.39927	down
HOFT_PBM	-0.55262	0.083176	-11.7439	3.64E-23	1.68E-22	41.82691	down
GOBP_RES	-0.55256	-0.05915	-16.559	4.56E-36	8.56E-35	71.41498	down
GOCC_SPII	0.552556	-0.09928	15.87127	2.87E-34	4.16E-33	67.28648	up
GSE23568_	0.552383	0.02386	20.44922	8.20E-46	1.04E-43	93.76169	up
MIR490_5F	-0.55238	-0.07777	-20.2797	2.10E-45	2.30E-43	92.82645	down
HP_PERITC	-0.55236	0.011971	-12.9403	2.05E-26	1.25E-25	49.27321	down
KYNG_ENV	-0.55227	-0.06527	-17.5844	1.04E-38	2.94E-37	77.47814	down
GOBP_CEL	-0.55223	-0.0025	-20.5683	4.25E-46	6.04E-44	94.41631	down
CUI_TCF21	-0.55217	-0.0925	-22.7476	3.45E-51	1.89E-48	106.0778	down
HP_CORNI	-0.55213	-0.02834	-10.5142	7.66E-20	2.73E-19	34.21701	down
WP_TRYPT	-0.5519	0.137552	-12.4159	5.44E-25	2.89E-24	46.0093	down
GOCC_PRE	0.551608	0.079925	10.65072	3.29E-20	1.20E-19	35.05691	up
WP_EICOS	-0.55156	0.031061	-15.8551	3.17E-34	4.57E-33	67.18885	down
GOMF_ENI	0.551536	0.02472	18.33028	1.33E-40	5.27E-39	81.81394	up
HP_CALCIF	-0.55144	-0.08808	-11.3041	5.67E-22	2.37E-21	39.09527	down
GOBP_NEC	-0.55143	-0.00344	-9.37262	8.29E-17	2.39E-16	27.28116	down
GOCC_CO	0.551425	-0.11828	8.851291	1.89E-15	4.99E-15	24.18277	up
NAKAMUR	0.551367	-0.0913	14.69717	3.75E-31	3.70E-30	60.13907	up
HP_ENCEP	-0.55133	0.01246	-13.4253	9.94E-28	6.79E-27	52.28668	down
GOBP_GLY	-0.55126	0.125885	-12.381	6.77E-25	3.57E-24	45.79175	down
GOMF_GLY	-0.55126	0.125885	-12.381	6.77E-25	3.57E-24	45.79175	down
HP_NIGHT	-0.5512	0.040749	-13.3057	2.10E-27	1.39E-26	51.54427	down
HP_VISUAI	-0.55108	0.057869	-12.1521	2.83E-24	1.42E-23	44.36682	down
GOMF_CD	-0.55104	0.133901	-9.16195	2.95E-16	8.21E-16	26.02278	down
GOCC_FAS	-0.55094	-0.02291	-12.2976	1.14E-24	5.91E-24	45.27228	down
HP_STAPES	-0.55092	0.10524	-11.5959	9.17E-23	4.09E-22	40.90669	down
MIR95_3P	-0.55087	-0.05318	-10.2666	3.53E-19	1.20E-18	32.69923	down
HP_PROGF	-0.55076	-0.00585	-10.0537	1.31E-18	4.27E-18	31.3988	down
GOBP_REG	0.550718	-0.03627	8.423905	2.35E-14	5.78E-14	21.68603	up
GOCC_HIS	0.550686	-0.08159	10.55297	6.03E-20	2.16E-19	34.45552	up
HP_MITOC	-0.55065	0.104657	-9.21466	2.15E-16	6.04E-16	26.33685	down
GOCC_RIB	0.550616	0.271894	10.83782	1.03E-20	3.92E-20	36.21027	up
PID_ERBB4	-0.55051	-0.05783	-23.9047	8.71E-54	1.56E-50	112.023	down
GOBP_POS	-0.55045	-0.12156	-9.59278	2.18E-17	6.55E-17	28.60453	down
KOBAYASH	-0.55041	-0.08098	-11.6503	6.53E-23	2.94E-22	41.24495	down
GSE14415_	0.550363	0.031204	18.63738	2.27E-41	1.03E-39	83.57991	up
REACTOM	-0.55036	-0.06308	-9.89306	3.50E-18	1.11E-17	30.42159	down

GOBP_CAN	0.55035	0.001852	8.494029	1.56E-14	3.88E-14	22.0927	up
GOBP_NEC	-0.5501	0.020713	-13.8058	9.32E-29	7.12E-28	54.64484	down
ABE_VEGF/	-0.55006	0.090153	-15.672	9.62E-34	1.30E-32	66.08186	down
GSE36476_	0.549996	-0.02515	17.25699	7.15E-38	1.75E-36	75.55473	up
GOBP_POS	-0.54986	0.066893	-9.76879	7.48E-18	2.32E-17	29.66798	down
GSE19888_	0.549697	0.022523	21.63325	1.28E-48	3.70E-46	100.1908	up
COLLER_M	-0.54959	0.061665	-9.27136	1.53E-16	4.33E-16	26.67533	down
DASU_IL6_	-0.54955	-0.003	-13.0056	1.36E-26	8.45E-26	49.67915	down
GOCC_CO	0.549526	-0.2956	8.497305	1.53E-14	3.81E-14	22.11173	up
GOBP_SER	-0.54946	0.01227	-17.5862	1.03E-38	2.92E-37	77.48864	down
HP_MACR(	-0.54941	0.041242	-11.0259	3.20E-21	1.26E-20	37.37235	down
HP_SUBCU	-0.5494	-0.02584	-10.4026	1.53E-19	5.33E-19	33.53215	down
SA_REG_C/	0.549275	-0.15492	11.5465	1.25E-22	5.50E-22	40.59984	up
PICCALUG.	-0.54915	-0.01594	-15.9537	1.74E-34	2.60E-33	67.78389	down
GSE19888_	0.549073	0.026384	21.89228	3.20E-49	1.09E-46	101.5734	up
GOMF_PR(	0.548766	0.011524	12.8762	3.06E-26	1.83E-25	48.87446	up
GRASEMAI	0.548709	-0.03981	10.97261	4.46E-21	1.74E-20	37.04284	up
GOBP_GLU	-0.54865	-0.03676	-11.5958	9.18E-23	4.09E-22	40.90615	down
HP_PENOS	-0.54855	-0.04333	-11.2491	7.98E-22	3.30E-21	38.75433	down
MODULE_4	-0.54854	0.123482	-14.0611	1.91E-29	1.56E-28	56.22374	down
HP_DECRE	-0.54854	-0.09442	-12.9078	2.51E-26	1.52E-25	49.07082	down
HP_EEG_W	-0.54841	-0.05013	-12.807	4.71E-26	2.77E-25	48.44394	down
WP_NUCLI	-0.5484	0.067118	-15.6542	1.07E-33	1.43E-32	65.97428	down
KEGG_BUT	-0.54823	0.133662	-11.3237	5.01E-22	2.11E-21	39.21667	down
GOMF_NEI	-0.54823	0.079283	-12.082	4.39E-24	2.17E-23	43.93022	down
HP_THROM	-0.54795	0.143325	-10.9486	5.18E-21	2.01E-20	36.89416	down
HP_HALIT(	-0.54791	0.147095	-11.5683	1.09E-22	4.82E-22	40.73544	down
MUELLER_	0.547906	0.003126	19.79038	3.23E-44	2.72E-42	90.10686	up
EPPERT_CE	-0.54773	-0.03004	-16.3119	2.01E-35	3.41E-34	69.93694	down
ZHAN_MU	-0.54767	-0.03101	-19.0903	1.69E-42	9.72E-41	86.16333	down
PID_IL1_PA	-0.54762	-0.09196	-19.6848	5.84E-44	4.64E-42	89.51586	down
MODULE_2	0.547551	-0.17246	14.48322	1.40E-30	1.29E-29	58.82496	up
GOBP_EST.	-0.54739	-0.04213	-18.633	2.32E-41	1.06E-39	83.55489	down
GOBP_PEN	-0.5473	0.048144	-13.9034	5.08E-29	3.99E-28	55.24865	down
GOMF_3_5	0.547265	-0.02973	12.96592	1.75E-26	1.07E-25	49.43258	up
HP_ELEVAT	-0.54721	0.289485	-7.83105	7.21E-13	1.62E-12	18.30083	down
GSE24634_	0.547132	-0.05103	16.33885	1.71E-35	2.94E-34	70.09842	up
GOBP_VAS	-0.54713	-0.04367	-16.3411	1.69E-35	2.90E-34	70.11218	down
GINESTIER	0.547075	-0.04868	17.25513	7.23E-38	1.77E-36	75.54378	up
GOBP_CHI	0.546918	-0.12836	16.21619	3.57E-35	5.80E-34	69.36288	up
GOBP_OUT	-0.54687	-0.02187	-14.2275	6.81E-30	5.82E-29	57.25001	down
MATHEW_	0.546682	-0.17742	8.627093	7.12E-15	1.81E-14	22.86769	up
GOBP_RIB(	0.546553	0.132898	10.9951	3.88E-21	1.52E-20	37.18186	up
HP_JOINT_	-0.54647	0.078388	-11.6713	5.73E-23	2.60E-22	41.37525	down
MIR503_5F	-0.54642	-0.09321	-23.804	1.46E-53	2.13E-50	111.5124	down
BIOCARTA	-0.54639	-0.07478	-9.34777	9.63E-17	2.77E-16	27.1323	down
MODULE_1	-0.54626	0.012597	-11.1603	1.39E-21	5.63E-21	38.20445	down
ZHENG_FC	-0.54622	-0.00362	-12.2176	1.88E-24	9.55E-24	44.77461	down
MIR4536_3	-0.5462	-0.02479	-15.4162	4.57E-33	5.68E-32	64.52987	down
REACTOM	-0.5462	-0.13737	-12.8168	4.43E-26	2.62E-25	48.50477	down
chr4p14	-0.54619	-0.01233	-12.5948	1.78E-25	9.87E-25	47.12331	down
HP_CEREBI	-0.54602	-0.08039	-9.56218	2.63E-17	7.85E-17	28.42009	down
GOBP_PLA	-0.54581	0.144583	-11.6935	4.98E-23	2.27E-22	41.51315	down
REACTOM	0.5458	-0.09974	13.77352	1.14E-28	8.62E-28	54.44532	up
GOBP_ZIN	-0.54578	-0.00682	-12.4805	3.63E-25	1.96E-24	46.41169	down
TAGGTCA_	-0.54564	-0.07816	-19.1494	1.21E-42	7.13E-41	86.49895	down
GOMF_OX	-0.5456	0.044815	-11.4341	2.52E-22	1.08E-21	39.9017	down
GOBP_NEC	-0.54553	0.111785	-9.43007	5.86E-17	1.71E-16	27.62571	down

GOBP_ATT	0.545419	-0.13003	14.08099	1.69E-29	1.39E-28	56.34632	up
GSE22886_	0.545371	-0.02835	16.75091	1.45E-36	2.94E-35	72.55827	up
FIGUEROA	-0.54533	0.02857	-10.512	7.77E-20	2.77E-19	34.20378	down
TIAN_TNF_	-0.5453	0.072583	-12.3813	6.75E-25	3.57E-24	45.79385	down
WP_SEROT	-0.54527	0.043262	-16.5714	4.23E-36	8.01E-35	71.48878	down
QI_PBMC_	-0.54525	0.104808	-9.51519	3.50E-17	1.04E-16	28.13725	down
GSE9006_H	0.545178	0.112885	17.40978	2.90E-38	7.67E-37	76.45385	up
GOBP_ICO	-0.54512	0.139809	-10.1095	9.29E-19	3.07E-18	31.73893	down
GOBP_NEC	-0.54506	0.089977	-9.4499	5.19E-17	1.52E-16	27.74477	down
GOMF_TRA	-0.54505	-0.02437	-16.7905	1.14E-36	2.36E-35	72.79382	down
GNF2_HPX	-0.54504	0.251358	-9.30677	1.23E-16	3.52E-16	26.88694	down
GOBP_REG	0.544975	-0.08661	9.177832	2.68E-16	7.49E-16	26.11735	up
HP_CONCA	0.544881	0.091217	16.30976	2.04E-35	3.45E-34	69.92409	up
OSADA_AS	-0.54482	0.038565	-12.2003	2.09E-24	1.06E-23	44.667	down
PID_GLYPI	-0.54475	-0.0147	-14.1978	8.19E-30	6.93E-29	57.06702	down
GOCC_END	-0.54472	0.148667	-8.20228	8.54E-14	2.03E-13	20.40914	down
MIR21_5P	-0.5447	-0.08265	-22.7933	2.71E-51	1.62E-48	106.3158	down
HP_SYSTO	-0.54428	0.029786	-14.2243	6.95E-30	5.93E-29	57.2304	down
HINATA_N	-0.54426	0.030365	-10.1507	7.21E-19	2.40E-18	31.99069	down
IIZUKA_LIV	-0.54425	0.093359	-9.85516	4.41E-18	1.39E-17	30.19154	down
REACTOM	0.544206	-0.02767	14.05239	2.02E-29	1.64E-28	56.16969	up
WENG_PO	-0.5442	0.058049	-15.7527	5.90E-34	8.19E-33	66.56988	down
YAMASHIT	-0.5442	0.131189	-11.5096	1.57E-22	6.86E-22	40.37088	down
CREIGHTO	0.54419	0.120962	11.60506	8.66E-23	3.87E-22	40.96367	up
HP_SPLIT_	0.544173	0.079573	12.32998	9.31E-25	4.87E-24	45.47416	up
BLANCO_M	0.544013	-0.31252	9.232856	1.93E-16	5.43E-16	26.4454	up
SCHAEFFE	-0.54399	0.139419	-10.7702	1.57E-20	5.89E-20	35.79292	down
BIOCARTA	-0.54399	-0.00877	-11.128	1.70E-21	6.84E-21	38.00447	down
REACTOM	-0.54387	0.101415	-11.1944	1.12E-21	4.59E-21	38.41556	down
GOBP_BAS	0.543742	-0.07777	10.48867	8.97E-20	3.19E-19	34.06037	up
RAX2_TAR	0.543651	0.055435	22.2077	5.95E-50	2.40E-47	103.2453	up
OUYANG_	-0.54352	0.005844	-18.9366	4.07E-42	2.15E-40	85.28949	down
SMID_BRE	-0.54352	0.035412	-14.2015	8.00E-30	6.78E-29	57.08972	down
MODULE_	-0.54346	-0.02066	-15.9375	1.92E-34	2.85E-33	67.6861	down
TRAVAGLI	-0.54343	0.026931	-15.5404	2.14E-33	2.77E-32	65.28411	down
GSE39556_	0.543331	-0.11855	13.76402	1.21E-28	9.13E-28	54.38649	up
MIR32_3P	-0.54329	-0.09259	-22.336	3.01E-50	1.28E-47	103.9215	down
HP_HYPOT	-0.54328	0.175557	-15.4563	3.58E-33	4.49E-32	64.77393	down
GOCC_TRI	-0.54326	0.074767	-9.89084	3.55E-18	1.13E-17	30.40811	down
GOBP_REG	-0.54321	0.139995	-9.9372	2.67E-18	8.55E-18	30.68979	down
GOBP_REG	0.543181	-0.03603	10.82551	1.11E-20	4.22E-20	36.13426	up
GOBP_CEL	-0.5431	-0.02356	-13.6996	1.80E-28	1.34E-27	53.98732	down
ERWIN_CC	-0.54303	0.159453	-9.41812	6.29E-17	1.83E-16	27.55403	down
GOBP_DOI	-0.54301	-0.03651	-8.00204	2.71E-13	6.25E-13	19.26696	down
GOMF_NEI	-0.54294	-0.03286	-9.90666	3.22E-18	1.03E-17	30.50419	down
GOMF_PH	-0.54292	-0.07677	-12.4116	5.59E-25	2.97E-24	45.98258	down
GOBP_IRES	0.542907	0.000643	10.48176	9.36E-20	3.32E-19	34.01793	up
GOMF_PRC	-0.5429	0.06114	-8.77162	3.03E-15	7.91E-15	23.71414	down
GSE30962_	0.542894	-0.11159	14.58215	7.62E-31	7.25E-30	59.43301	up
HALLMARI	0.542818	0.032726	13.54219	4.80E-28	3.39E-27	53.01195	up
GOBP_REG	-0.54253	0.058551	-12.5052	3.11E-25	1.69E-24	46.56506	down
REACTOM	-0.54251	0.100017	-14.1378	1.19E-29	9.88E-29	56.69705	down
MODULE_	-0.5425	-0.01238	-14.1814	9.06E-30	7.64E-29	56.96593	down
HP_NONIN	0.54248	0.16737	13.14182	5.82E-27	3.72E-26	50.52626	up
GSE21063_	0.542459	-0.02966	21.47749	2.97E-48	7.86E-46	99.3553	up
HP_ABSEN	0.542386	0.133762	16.6744	2.28E-36	4.48E-35	72.10285	up
chr3p24	-0.54236	-0.02396	-15.6099	1.40E-33	1.85E-32	65.70538	down
GOBP_P38	-0.54223	0.031828	-21.6778	1.01E-48	2.96E-46	100.4291	down

GSE39110_	0.542053	-0.0704	15.98893	1.41E-34	2.13E-33	67.99606	up
BIOCARTA	0.541962	-0.02463	15.36746	6.15E-33	7.49E-32	64.23372	up
MORF_PPI	0.541812	0.163226	11.90161	1.36E-23	6.46E-23	42.80762	up
HP_MIXED	-0.54154	0.154594	-7.46485	5.68E-12	1.21E-11	16.26285	down
GOBP_NEG	-0.54146	0.029381	-8.8343	2.09E-15	5.51E-15	24.08271	down
REACTOM	0.541398	0.019807	11.11059	1.89E-21	7.60E-21	37.89638	up
MIR192_3F	-0.54125	-0.08097	-22.0724	1.22E-49	4.48E-47	102.5298	down
HP_CHRONI	0.541247	-0.13627	9.381822	7.84E-17	2.27E-16	27.33632	up
MIR184	-0.54122	-0.00285	-13.2936	2.26E-27	1.50E-26	51.46906	down
GOBP_NUC	0.541094	-0.04311	14.07978	1.70E-29	1.39E-28	56.33884	up
MODULE_1	0.541013	0.055035	10.65217	3.26E-20	1.19E-19	35.06581	up
GOBP_ACU	-0.54101	0.168056	-13.6583	2.33E-28	1.71E-27	53.73156	down
IKEDA_MIF	-0.54089	-0.12906	-15.464	3.41E-33	4.29E-32	64.82072	down
GOBP_BRA	-0.5407	0.086553	-12.896	2.70E-26	1.63E-25	48.99779	down
GOMF_VIT	-0.54068	0.120112	-12.7737	5.80E-26	3.38E-25	48.23689	down
GOBP_REF	-0.54054	-0.0145	-16.3413	1.68E-35	2.90E-34	70.11284	down
YAGUE_PR	-0.54053	-0.1597	-10.9397	5.48E-21	2.12E-20	36.83947	down
BUSSLINGI	0.540521	0.225701	11.95914	9.47E-24	4.56E-23	43.1656	up
REACTOM	0.540494	-0.05549	19.79363	3.17E-44	2.68E-42	90.12505	up
HP_ABNOI	0.540248	-0.07489	12.68584	1.01E-25	5.73E-25	47.68981	up
HALLMARI	-0.54005	-0.08934	-21.9326	2.58E-49	8.90E-47	101.7876	down
DELYS_TH\	-0.54001	0.040844	-19.2648	6.27E-43	3.92E-41	87.15213	down
IRITANI_M	0.539954	0.087233	12.80864	4.67E-26	2.74E-25	48.45408	up
GOMF_NU	-0.53991	0.109177	-9.71547	1.03E-17	3.18E-17	29.34533	down
GOBP_NEG	-0.53989	0.075083	-9.65331	1.51E-17	4.58E-17	28.9697	down
GOMF_LIP	-0.53988	0.085382	-9.54562	2.91E-17	8.65E-17	28.32036	down
REACTOM	-0.53986	-0.07996	-13.7622	1.22E-28	9.22E-28	54.37546	down
GOMF_WA	-0.53963	0.097869	-13.8442	7.34E-29	5.67E-28	54.8825	down
GOBP_REG	-0.53962	0.049006	-28.7058	8.04E-64	1.30E-59	134.9461	down
GOCC_PHO	-0.53946	-0.1371	-10.0302	1.51E-18	4.92E-18	31.25584	down
VILLANUE\	-0.53945	0.168587	-9.20525	2.27E-16	6.38E-16	26.28076	down
GOBP_POS	-0.53933	0.007044	-16.2257	3.38E-35	5.50E-34	69.42008	down
HP_FAILUF	-0.53932	-0.09173	-10.6811	2.73E-20	1.00E-19	35.24391	down
GOBP_ACT	-0.53928	0.067052	-13.9886	2.99E-29	2.40E-28	55.77569	down
GOMF_TRA	-0.53925	0.138407	-11.5803	1.01E-22	4.48E-22	40.80954	down
KEGG_SPLI	0.539219	0.014687	15.59113	1.57E-33	2.06E-32	65.59186	up
GOBP_POS	-0.53907	0.097667	-9.78936	6.59E-18	2.05E-17	29.79263	down
WENG_PO	-0.53904	0.119097	-14.2856	4.75E-30	4.12E-29	57.60845	down
GSE14415_	0.538946	-0.05914	15.79134	4.66E-34	6.58E-33	66.80373	up
MODULE_S	0.538898	0.12207	10.37573	1.80E-19	6.27E-19	33.36735	up
GOCC_DN	0.538872	-0.08826	12.56292	2.17E-25	1.20E-24	46.92456	up
HP_MULTI	-0.53887	0.199672	-7.7517	1.13E-12	2.51E-12	17.85558	down
GOMF_SHO	-0.53887	-0.01308	-10.1123	9.12E-19	3.02E-18	31.75633	down
HP_COMM	-0.53872	-0.04096	-14.4632	1.59E-30	1.45E-29	58.70164	down
GOBP_POS	-0.5387	0.029366	-9.80997	5.81E-18	1.82E-17	29.91749	down
ACEVEDO_	-0.53869	0.170473	-14.9129	9.95E-32	1.05E-30	61.46055	down
GOMF_TEL	0.538668	-0.14213	9.819085	5.50E-18	1.72E-17	29.97275	up
GOMF_RN	0.538668	-0.14213	9.819085	5.50E-18	1.72E-17	29.97275	up
GNF2_HPN	-0.5386	0.253471	-9.19726	2.38E-16	6.68E-16	26.2331	down
GOMF_UBI	0.538478	0.254517	8.694065	4.79E-15	1.24E-14	23.25933	up
HP_PROMI	-0.53833	-0.1103	-14.7925	2.08E-31	2.12E-30	60.72366	down
HP_ABNOI	-0.53822	-0.01476	-17.6934	5.46E-39	1.62E-37	78.11596	down
REACTOM	0.538203	-0.12822	12.93704	2.09E-26	1.27E-25	49.25293	up
REACTOM	-0.53801	0.084947	-9.96087	2.31E-18	7.44E-18	30.8337	down
GRANDVA	-0.53799	0.012632	-10.2135	4.90E-19	1.65E-18	32.37437	down
GOBP_MIT	0.537934	-0.061	18.38215	9.88E-41	4.02E-39	82.11299	up
GOBP_POL	0.537921	0.159787	9.946718	2.52E-18	8.08E-18	30.74766	up
GSE21546_	0.537911	-0.04809	21.10524	2.24E-47	4.69E-45	97.34592	up

RAY_TARG	-0.53772	-0.05038	-13.7148	1.64E-28	1.22E-27	54.08169	down
MIR553	-0.53772	-0.08051	-13.1771	4.67E-27	3.00E-26	50.74557	down
HP_NEURC	-0.53761	0.015596	-12.6461	1.29E-25	7.27E-25	47.44258	down
GOBP_MEI	-0.53757	0.153106	-7.20333	2.42E-11	4.95E-11	14.83545	down
GOBP_MA	-0.53756	0.038276	-14.5228	1.10E-30	1.03E-29	59.06818	down
WAESCH_/	0.537496	-0.07963	14.91932	9.56E-32	1.02E-30	61.50005	up
GOMF_LIP	-0.53748	0.016225	-15.0626	3.97E-32	4.38E-31	62.37567	down
GOBP_DOI	-0.53743	0.092071	-14.2404	6.29E-30	5.39E-29	57.33002	down
GOBP_REG	-0.53743	0.037201	-11.7842	2.83E-23	1.31E-22	42.07707	down
GOBP_POS	-0.5373	0.089294	-10.6438	3.44E-20	1.25E-19	35.01412	down
MORF_RRI	0.53712	-0.10482	14.19106	8.54E-30	7.21E-29	57.02557	up
HOEK_T_C	0.537086	-0.06528	7.786997	9.26E-13	2.06E-12	18.05339	up
GOCC_LIP	-0.53701	0.155697	-9.7906	6.54E-18	2.04E-17	29.80009	down
HP_ABNOI	0.536943	0.271626	11.63937	6.99E-23	3.15E-22	41.17686	up
GOCC_CAT	0.536929	0.006761	11.78457	2.82E-23	1.31E-22	42.07956	up
GNF2_GST	-0.53683	0.255657	-9.05005	5.77E-16	1.57E-15	25.35778	down
BARIS_THY	0.536824	0.049295	16.25406	2.85E-35	4.70E-34	69.5901	up
GOBP_REG	0.536776	-0.12798	14.90253	1.06E-31	1.12E-30	61.39731	up
HP_RETICU	0.536755	-0.05348	10.13404	7.98E-19	2.65E-18	31.88882	up
HP_ABNOI	-0.53673	-0.04572	-14.4803	1.43E-30	1.32E-29	58.80687	down
OHGUCHI_	-0.53672	0.146558	-11.9052	1.33E-23	6.32E-23	42.83011	down
GNF2_HAT	0.536612	-0.08811	12.55202	2.32E-25	1.28E-24	46.85668	up
HP_DECRE	-0.53653	0.252246	-8.2244	7.51E-14	1.79E-13	20.53599	down
MIR6718_5	-0.53646	-0.10799	-20.4466	8.32E-46	1.04E-43	93.74733	down
JECHLINGE	-0.53643	-0.00367	-19.615	8.65E-44	6.57E-42	89.1246	down
HP_TENDC	-0.53643	0.17342	-10.1199	8.71E-19	2.88E-18	31.80233	down
GOBP_NEC	-0.5363	0.076903	-8.76264	3.19E-15	8.33E-15	23.66141	down
GOCC_STA	0.536105	-0.00812	13.79937	9.69E-29	7.39E-28	54.6053	up
GOBP_TEL	0.536023	-0.10555	13.71115	1.68E-28	1.25E-27	54.05908	up
GOBP_REG	-0.53595	-0.05968	-10.9068	6.72E-21	2.59E-20	36.63626	down
GOMF_MC	-0.53592	0.006766	-13.2839	2.40E-27	1.59E-26	51.40918	down
HP_RIB_SE	0.53562	0.019003	12.52095	2.82E-25	1.54E-24	46.66327	up
VERNELL_F	-0.53557	-0.03983	-19.6964	5.47E-44	4.39E-42	89.58114	down
GOMF_LOI	-0.53557	0.124585	-10.0161	1.65E-18	5.35E-18	31.16955	down
GOBP_NEC	-0.53557	0.068985	-14.6007	6.79E-31	6.51E-30	59.54696	down
GOMF_D_(	-0.53541	0.028231	-13.6386	2.63E-28	1.91E-27	53.60937	down
REACTOM	0.535374	0.021795	13.64278	2.57E-28	1.87E-27	53.63554	up
GNF2_CAR	-0.53537	0.130904	-9.53614	3.08E-17	9.15E-17	28.26331	down
TRAVAGLI	-0.53536	0.018713	-15.8782	2.76E-34	4.00E-33	67.32805	down
REACTOM	-0.53536	0.09824	-8.63154	6.94E-15	1.77E-14	22.89366	down
GNF2_LCA	-0.53532	0.25152	-9.14526	3.26E-16	9.05E-16	25.92341	down
GOCC_PIN	-0.53527	-0.27167	-9.92229	2.93E-18	9.35E-18	30.59918	down
GOMF_CA	-0.53525	-0.02571	-10.7099	2.28E-20	8.44E-20	35.42143	down
GOMF_1_F	-0.53517	-0.19715	-10.8982	7.09E-21	2.72E-20	36.5829	down
GOBP_REG	-0.53511	-0.00256	-14.9945	6.03E-32	6.54E-31	61.95971	down
GOBP_TOL	-0.5351	-0.02539	-17.0284	2.77E-37	6.26E-36	74.20475	down
HP_ABNOI	-0.53507	0.080054	-9.27939	1.45E-16	4.13E-16	26.72326	down
GOMF_ENI	0.534985	0.107028	12.49318	3.35E-25	1.82E-24	46.49034	up
GROSS_HY	-0.53492	0.079767	-16.6023	3.52E-36	6.73E-35	71.67344	down
HP_IMMO	0.534873	0.004589	11.0482	2.79E-21	1.11E-20	37.51027	up
ZHENG_FC	-0.53482	-0.08851	-17.7849	3.20E-39	9.88E-38	78.65028	down
MEINHOLI	0.534788	-0.12291	14.3139	3.99E-30	3.49E-29	57.78277	up
GOCC_RN/	0.534623	0.139049	12.00393	7.16E-24	3.48E-23	43.44438	up
TSUNODA	-0.53462	0.0831	-15.7221	7.10E-34	9.75E-33	66.38505	down
CHUANG_	0.534569	-0.0587	12.04773	5.44E-24	2.67E-23	43.71697	up
REACTOM	-0.53448	-0.04184	-15.0706	3.78E-32	4.19E-31	62.42443	down
DESCARTE	-0.53444	0.131345	-8.60609	8.06E-15	2.04E-14	22.74508	down
TAATGTG_	-0.53439	-0.11834	-21.7986	5.28E-49	1.62E-46	101.0744	down

WP_VITAM	-0.53439	0.12769	-14.886	1.17E-31	1.23E-30	61.29586	down
GOBP_VIR	0.534341	0.138681	14.17157	9.63E-30	8.07E-29	56.90538	up
chr5q21	-0.53432	-0.04344	-10.1737	6.26E-19	2.09E-18	32.13101	down
TURASHVI	-0.53419	0.016556	-16.8581	7.63E-37	1.62E-35	73.19529	down
BIOCARTA	-0.53418	-0.11942	-13.8246	8.29E-29	6.36E-28	54.76148	down
HP_ASYMI	-0.53414	0.015546	-8.82893	2.16E-15	5.68E-15	24.05109	down
HP_CHRO	-0.5341	-0.02867	-13.4623	7.89E-28	5.46E-27	52.51643	down
GOCC_OU	0.534082	-0.05055	12.62788	1.44E-25	8.10E-25	47.32897	up
GOBP_PER	-0.53404	-0.11224	-12.868	3.22E-26	1.92E-25	48.82361	down
REACTOM	0.533967	0.02207	16.85079	7.97E-37	1.69E-35	73.15183	up
GNF2_TST	-0.53392	0.255001	-9.06139	5.39E-16	1.47E-15	25.42502	down
REACTOM	0.533761	-0.01236	16.47977	7.33E-36	1.34E-34	70.94164	up
HP_NARR	0.533733	-0.12054	13.05596	9.95E-27	6.24E-26	49.9925	up
GOBP_NE	-0.53371	0.175756	-12.4702	3.87E-25	2.09E-24	46.34715	down
GOCC_CO	0.533682	-0.13846	15.10359	3.09E-32	3.47E-31	62.62608	up
HP_ABNO	-0.53364	0.090569	-11.2326	8.85E-22	3.64E-21	38.65189	down
MOOTHA_	-0.53351	0.166089	-10.1172	8.86E-19	2.93E-18	31.78595	down
MAHADEV	-0.53346	0.044483	-12.9467	1.97E-26	1.20E-25	49.31315	down
HSD17B8_	0.533414	-0.11203	15.78362	4.89E-34	6.87E-33	66.75704	up
GOBP_DIC	-0.53326	0.114323	-12.5705	2.07E-25	1.14E-24	46.97185	down
WU_APOP	0.533234	-0.10373	12.38895	6.44E-25	3.41E-24	45.84137	up
HP_ABNO	0.53317	0.003516	10.83308	1.06E-20	4.03E-20	36.18099	up
PEPPER_C	-0.53317	0.068755	-16.0215	1.16E-34	1.77E-33	68.19228	down
GOBP_NE	0.533156	-0.01201	15.41519	4.60E-33	5.71E-32	64.52391	up
PEPPER_C	-0.53307	0.025942	-18.4736	5.83E-41	2.49E-39	82.63936	down
GOMF_EN	0.53305	-0.01692	22.53855	1.03E-50	5.12E-48	104.9853	up
GOBP_OR	-0.53298	0.140337	-12.055	5.20E-24	2.55E-23	43.76205	down
MODULE_	-0.53289	0.0291	-11.4248	2.67E-22	1.14E-21	39.84433	down
GNF2_APE	0.532859	-0.02088	13.15238	5.45E-27	3.49E-26	50.59188	up
GOBP_MA	-0.53286	0.055828	-9.76298	7.75E-18	2.40E-17	29.63281	down
MIR203A_	-0.53283	-0.13483	-18.6831	1.74E-41	8.08E-40	83.84206	down
chr12q12	-0.53282	-0.08629	-12.5053	3.11E-25	1.69E-24	46.56556	down
GOBP_NE	-0.53271	0.075087	-11.053	2.71E-21	1.07E-20	37.54029	down
GOMF_IM	-0.53264	0.198083	-7.72784	1.30E-12	2.86E-12	17.72206	down
ZAK_PBM	-0.53258	-0.16667	-12.9216	2.30E-26	1.40E-25	49.15688	down
GOMF_CA	-0.53245	-0.0562	-12.4152	5.46E-25	2.91E-24	46.00478	down
GSE21546_	0.532452	-0.03568	19.43686	2.36E-43	1.62E-41	88.12321	up
GOBP_AD	-0.53245	0.062177	-12.4373	4.76E-25	2.54E-24	46.14265	down
KEGG_DRU	-0.53234	0.109587	-12.0463	5.49E-24	2.69E-23	43.70825	down
FAN_OVA	0.532309	0.216168	12.85487	3.50E-26	2.08E-25	48.74175	up
REACTOM	-0.53226	0.10253	-13.4776	7.18E-28	4.98E-27	52.61146	down
AIZARANI_	-0.53219	-0.07195	-15.0721	3.75E-32	4.15E-31	62.43407	down
MIR4735_	-0.53217	-0.0897	-21.1983	1.35E-47	3.00E-45	97.8498	down
GOMF_UB	0.532128	0.105502	16.68147	2.19E-36	4.31E-35	72.14497	up
REACTOM	-0.53207	0.050343	-9.48182	4.28E-17	1.26E-16	27.93656	down
GSE45837_	0.532058	0.179006	13.88397	5.73E-29	4.47E-28	55.12878	up
ANDERSO	-0.53195	0.19865	-6.58373	6.76E-10	1.27E-09	11.55882	down
GOBP_RES	-0.53195	0.09956	-10.08	1.11E-18	3.66E-18	31.55899	down
REACTOM	-0.53188	0.12448	-13.0039	1.38E-26	8.53E-26	49.66906	down
MODULE_	0.531748	0.12761	9.712233	1.06E-17	3.24E-17	29.32577	up
GOBP_RR	0.531657	0.000896	10.06921	1.19E-18	3.90E-18	31.4934	up
FAN_EMB	-0.53165	0.110858	-13.635	2.69E-28	1.95E-27	53.58719	down
REACTOM	-0.53165	-0.00999	-11.627	7.55E-23	3.39E-22	41.09997	down
GOBP_PO	-0.5315	-0.03584	-14.6068	6.54E-31	6.29E-30	59.5846	down
MIR203B_	-0.53133	-0.1065	-20.3582	1.36E-45	1.56E-43	93.26028	down
GOMF_TO	-0.53132	0.072026	-12.329	9.37E-25	4.90E-24	45.46796	down
GOBP_MO	-0.5313	0.149889	-11.4038	3.04E-22	1.30E-21	39.71413	down
GOBP_ME	-0.53109	0.10867	-11.3981	3.15E-22	1.35E-21	39.67863	down

BARRIER_C	-0.53107	-0.00871	-21.5589	1.91E-48	5.19E-46	99.79232	down
GOBP_GLU	-0.53088	0.020098	-11.7647	3.19E-23	1.48E-22	41.95575	down
chr8q24	0.530843	-0.03607	11.33028	4.81E-22	2.02E-21	39.25772	up
DESCARTE	-0.53084	0.096258	-12.28	1.27E-24	6.57E-24	45.16273	down
GOBP_TRA	0.530678	-0.26487	9.579925	2.36E-17	7.06E-17	28.52703	up
GSE5679_C	0.530665	-0.00546	18.3368	1.28E-40	5.10E-39	81.8515	up
HP_VENOL	-0.53061	0.034292	-20.899	6.91E-47	1.26E-44	96.22485	down
CHEN_HO)	-0.53057	0.13661	-10.5876	4.87E-20	1.76E-19	34.66822	down
MIR875_5F	-0.53055	-0.12896	-18.8604	6.30E-42	3.20E-40	84.85504	down
GOBP_POS	-0.53049	-0.01966	-17.756	3.79E-39	1.15E-37	78.4813	down
REACTOM	-0.53048	0.196436	-8.0102	2.59E-13	5.97E-13	19.31325	down
GOBP_RRN	0.530454	0.035101	13.47654	7.22E-28	5.01E-27	52.6048	up
FOXR2_TA	0.530367	0.067335	17.57071	1.12E-38	3.17E-37	77.398	up
ZIRN_TRET	-0.53035	-0.02555	-11.8683	1.67E-23	7.90E-23	42.60012	down
BIOCARTA	0.530303	-0.11037	9.701567	1.13E-17	3.45E-17	29.26128	up
HP_ABSEN	-0.53028	-0.04857	-10.4455	1.17E-19	4.13E-19	33.79547	down
GOMF_TRI	0.530235	-0.01693	11.61121	8.33E-23	3.73E-22	41.00184	up
GOBP_WO	-0.5302	-0.13338	-15.7908	4.68E-34	6.60E-33	66.80041	down
HP_REDUC	-0.52998	0.066253	-9.439	5.55E-17	1.62E-16	27.67932	down
MIR15A_3F	-0.52993	-0.07827	-17.851	2.17E-39	6.96E-38	79.03536	down
MODULE_4	-0.5299	0.168537	-11.4502	2.28E-22	9.83E-22	40.00161	down
GOBP_MIT	0.529765	-0.11836	16.09307	7.51E-35	1.17E-33	68.62303	up
GOBP_POS	-0.52968	-0.0167	-16.2738	2.53E-35	4.20E-34	69.70875	down
HP_FEMAL	-0.52961	0.009356	-14.3839	2.59E-30	2.32E-29	58.21358	down
GOBP_DN/	0.52948	0.106176	9.426584	5.98E-17	1.74E-16	27.60479	up
GOBP_INSI	-0.52937	0.032378	-22.5372	1.04E-50	5.12E-48	104.9784	down
QIU_PBMC	-0.52918	0.043155	-16.0696	8.65E-35	1.34E-33	68.48205	down
WAMUNY(	-0.52917	-0.08015	-19.6848	5.84E-44	4.64E-42	89.51614	down
GOCC_KIN	0.528908	-0.01215	11.14574	1.52E-21	6.14E-21	38.11399	up
NIKOLSKY_	0.528885	-0.03008	11.22221	9.44E-22	3.88E-21	38.58772	up
DESERT_ST	0.528878	-0.11905	11.44409	2.37E-22	1.02E-21	39.96389	up
GOMF_OX	-0.52866	0.096479	-10.9393	5.49E-21	2.13E-20	36.83689	down
GOBP_REG	-0.52863	-0.01481	-14.9351	8.68E-32	9.27E-31	61.59634	down
LI_PBMC_N	-0.5286	0.123608	-12.8477	3.66E-26	2.17E-25	48.69683	down
HP_HYPER	-0.52851	0.129452	-8.65204	6.15E-15	1.57E-14	23.01344	down
GOBP_REG	-0.52824	0.081574	-10.0618	1.24E-18	4.07E-18	31.44837	down
GOBP_MA	-0.52816	0.003717	-10.2616	3.64E-19	1.24E-18	32.66829	down
GOBP_RES	-0.52816	0.106835	-16.7012	1.95E-36	3.86E-35	72.26257	down
GOBP_MO	0.52815	0.034368	10.53543	6.72E-20	2.40E-19	34.3477	up
MORF_BUI	0.528057	-0.15224	12.49081	3.40E-25	1.84E-24	46.47557	up
HP_DICARI	-0.52805	0.156134	-10.3891	1.66E-19	5.79E-19	33.44961	down
REACTOM	0.527901	-0.19021	8.451191	2.00E-14	4.95E-14	21.84413	up
GTAAGAT_	-0.52773	-0.07639	-18.865	6.13E-42	3.12E-40	84.88127	down
GSE14415_	0.527539	-0.06747	15.03361	4.74E-32	5.19E-31	62.19878	up
GOBP_NEC	-0.52741	-0.03915	-14.6851	4.04E-31	3.98E-30	60.06486	down
GOBP_BILE	-0.52726	0.083696	-13.4596	8.03E-28	5.55E-27	52.49949	down
chr5q22	-0.52706	-0.00504	-10.7017	2.40E-20	8.86E-20	35.37061	down
HP_ATYPIC	-0.52697	0.006613	-21.1286	1.97E-47	4.18E-45	97.47265	down
GOBP_HIST	0.526903	-0.0146	15.2502	1.26E-32	1.48E-31	63.52001	up
WP_EV_RE	-0.52689	0.106532	-10.329	2.40E-19	8.29E-19	33.08079	down
DESCARTE	-0.52689	0.186001	-10.5395	6.55E-20	2.35E-19	34.37249	down
chr6q24	-0.52681	0.079503	-12.6398	1.34E-25	7.55E-25	47.40294	down
REACTOM	0.526778	-0.05664	19.23842	7.29E-43	4.52E-41	87.00286	up
DESCARTE	-0.52677	-0.05543	-8.9325	1.16E-15	3.11E-15	24.66187	down
GSE14769_	-0.5267	-0.00579	-20.8893	7.28E-47	1.31E-44	96.17208	down
MORF_BUI	0.526685	0.00046	15.65469	1.07E-33	1.43E-32	65.97696	up
GOBP_IND	-0.52668	0.165723	-10.0902	1.05E-18	3.44E-18	31.62132	down
REACTOM	0.526664	-0.13967	10.55338	6.01E-20	2.16E-19	34.45801	up

REACTOM	0.526476	-0.06794	13.61039	3.14E-28	2.26E-27	53.43477	up
GOMF_MA	-0.52645	0.139444	-16.0845	7.91E-35	1.23E-33	68.57168	down
AIZARANI_	-0.5263	-0.04796	-14.5727	8.07E-31	7.66E-30	59.37519	down
GOBP_NEC	0.526198	-0.04426	8.529413	1.27E-14	3.17E-14	22.29836	up
GOBP_DN/	0.526193	0.006793	14.86781	1.31E-31	1.37E-30	61.18476	up
GOMF_OX	-0.52614	0.066041	-12.756	6.48E-26	3.76E-25	48.12647	down
HP_CONFE	0.526114	-0.12615	11.39868	3.14E-22	1.34E-21	39.68207	up
GOBP_POS	-0.52594	0.058744	-10.7189	2.16E-20	8.00E-20	35.47667	down
HP_ABNOI	-0.52593	0.07402	-9.7779	7.07E-18	2.20E-17	29.72319	down
MODULE_8	-0.52584	-0.02848	-15.0467	4.38E-32	4.81E-31	62.27896	down
ATAAGCT_	-0.52582	-0.1136	-19.9099	1.65E-44	1.51E-42	90.77396	down
GOBP_PEP	-0.5258	0.069583	-9.0444	5.97E-16	1.63E-15	25.32421	down
HP_BAND_	-0.52573	0.003551	-11.0042	3.67E-21	1.44E-20	37.23835	down
HP_CORTI	0.525503	-0.16984	9.437766	5.59E-17	1.63E-16	27.67191	up
GOBP_INT	-0.52538	-0.00499	-11.46	2.14E-22	9.27E-22	40.06268	down
GOBP_MA	0.525326	0.064755	9.653117	1.51E-17	4.59E-17	28.96855	up
PID_ANGI	-0.52531	-0.07802	-19.1279	1.37E-42	7.93E-41	86.37656	down
GSE6259_3	-0.52523	0.003445	-19.602	9.31E-44	7.00E-42	89.05183	down
GOBP_POS	-0.52519	0.069328	-12.5799	1.95E-25	1.08E-24	47.03035	down
LAKE_ADU	-0.52517	-0.12841	-18.5334	4.13E-41	1.80E-39	82.98308	down
GOBP_ATT	0.525095	-0.1351	13.88938	5.54E-29	4.33E-28	55.16223	up
FAELT_B_C	0.525077	-0.06825	13.44724	8.67E-28	5.97E-27	52.423	up
SCIAN_CEL	0.525042	-0.01281	10.19313	5.55E-19	1.86E-18	32.24967	up
LAKE_ADU	-0.52502	-0.08867	-19.8503	2.30E-44	2.03E-42	90.44162	down
WP_HUMA	-0.52495	0.122876	-14.464	1.58E-30	1.45E-29	58.7067	down
WP_TRANS	-0.52472	0.042916	-13.331	1.79E-27	1.20E-26	51.7013	down
GOBP_RRN	0.524669	0.015805	13.39243	1.22E-27	8.29E-27	52.08284	up
GOMF_AN	0.524587	-0.23003	10.37823	1.77E-19	6.18E-19	33.38269	up
LAU_APOF	-0.52459	0.029054	-10.9606	4.81E-21	1.87E-20	36.96888	down
GOBP_TRN	0.524572	0.096655	12.48818	3.46E-25	1.87E-24	46.45922	up
WALLACE_	0.524564	0.126356	13.52742	5.26E-28	3.71E-27	52.92039	up
HP_TYPE_1	0.524487	0.001331	9.808524	5.87E-18	1.83E-17	29.90873	up
GOBP_COI	-0.5244	0.048721	-15.6851	8.89E-34	1.20E-32	66.16137	down
HP_ABNOI	-0.52438	0.079719	-8.83993	2.02E-15	5.33E-15	24.11583	down
MIR6739_3	-0.52436	-0.07936	-23.5407	5.61E-53	5.49E-50	110.171	down
GSE7568_I	0.524251	-0.01583	21.2631	9.49E-48	2.27E-45	98.20024	up
GOBP_EMI	-0.52419	0.010614	-15.3129	8.58E-33	1.03E-31	63.90164	down
STK33_NO	-0.52414	-0.05125	-19.8942	1.80E-44	1.62E-42	90.68671	down
LI_WILMS_	-0.52408	-0.00568	-20.7427	1.63E-46	2.59E-44	95.37188	down
GOBP_RES	-0.52401	0.035909	-16.1826	4.38E-35	7.02E-34	69.16106	down
GOMF_7S_	0.523986	0.02586	9.890492	3.55E-18	1.13E-17	30.40602	up
HP_INTERI	-0.52392	-0.01105	-14.0885	1.61E-29	1.32E-28	56.39275	down
GOBP_IND	-0.52389	0.089401	-15.3323	7.62E-33	9.18E-32	64.01999	down
GRAHAM_	0.523852	-0.06106	14.15156	1.09E-29	9.10E-29	56.78188	up
GOBP_RES	-0.52369	0.0435	-10.3085	2.73E-19	9.37E-19	32.95564	down
MARSON_	0.523681	-0.08902	17.48906	1.82E-38	4.95E-37	76.9193	up
REACTOM	-0.52362	-0.1471	-15.9101	2.27E-34	3.34E-33	67.52069	down
HP_DISTAL	-0.52361	0.13913	-9.77718	7.10E-18	2.21E-17	29.71884	down
GOBP_RES	-0.5236	-0.05128	-16.8803	6.69E-37	1.43E-35	73.3273	down
GOCC_EN	0.523597	0.054843	9.311275	1.20E-16	3.43E-16	26.91391	up
HP_HYDRC	-0.52349	0.08802	-14.1116	1.40E-29	1.15E-28	56.53544	down
LEE_LIVER_	-0.52322	0.102208	-12.9347	2.12E-26	1.29E-25	49.23839	down
HP_LACK_(	0.523183	0.020781	9.767648	7.53E-18	2.34E-17	29.6611	up
REACTOM	0.523066	-0.10491	12.00815	6.97E-24	3.39E-23	43.47059	up
REACTOM	-0.52302	0.084586	-13.1049	7.33E-27	4.64E-26	50.29691	down
GOCC_DIH	-0.523	0.054441	-9.37784	8.03E-17	2.32E-16	27.31245	down
GOBP_GU/	0.522788	0.047515	11.62346	7.72E-23	3.46E-22	41.07798	up
REACTOM	-0.52275	0.030412	-11.4237	2.69E-22	1.15E-21	39.83743	down

LEE_LIVER_	0.522654	-0.02861	13.39395	1.21E-27	8.21E-27	52.0923	up
MORF_SOI	0.522589	0.059105	13.91435	4.75E-29	3.75E-28	55.31661	up
WP_AFLAT	-0.52255	0.191444	-7.87798	5.52E-13	1.25E-12	18.56512	down
CTAGGAA_	-0.52226	-0.08746	-21.9332	2.57E-49	8.90E-47	101.7908	down
GOBP_VEN	-0.5222	-0.00471	-14.3676	2.86E-30	2.56E-29	58.11347	down
TURASHVI	-0.52219	-0.01764	-20.0056	9.66E-45	9.42E-43	91.30663	down
MIR8084	-0.52207	-0.1016	-21.1723	1.55E-47	3.34E-45	97.70909	down
GOBP_RES	-0.52206	0.060048	-8.32015	4.31E-14	1.04E-13	21.0866	down
HP_HEMO	-0.52196	0.096262	-9.51878	3.42E-17	1.01E-16	28.1588	down
REACTOM	-0.52167	0.045509	-9.6584	1.46E-17	4.45E-17	29.00044	down
MODULE_8	-0.52165	-0.00183	-12.6846	1.01E-25	5.77E-25	47.68221	down
GOMF_STE	-0.52145	0.124785	-11.4918	1.76E-22	7.65E-22	40.26	down
GOMF_DN	0.521383	-0.10636	10.46745	1.02E-19	3.62E-19	33.93008	up
GOBP_CH(	-0.52135	0.119518	-9.50321	3.76E-17	1.11E-16	28.06514	down
GOBP_SEQ	-0.52127	0.029038	-14.5096	1.19E-30	1.11E-29	58.98693	down
HP_DECRE	-0.52126	-0.07092	-10.4025	1.53E-19	5.34E-19	33.53172	down
REACTOM	-0.52124	-0.08381	-10.3027	2.83E-19	9.70E-19	32.91973	down
REACTOM	-0.52121	0.020235	-11.1715	1.29E-21	5.26E-21	38.27376	down
GOMF_O_I	-0.52119	0.04516	-10.0233	1.58E-18	5.13E-18	31.21375	down
HP_ANURI	-0.52115	0.080441	-9.59349	2.17E-17	6.52E-17	28.60879	down
WP_PHASE	-0.52102	0.149726	-8.18209	9.60E-14	2.27E-13	20.29342	down
YBX3_TAR(	0.520885	-0.05594	18.31628	1.45E-40	5.67E-39	81.73314	up
CHEN_LVA	-0.52084	0.059735	-15.9502	1.78E-34	2.65E-33	67.76266	down
MODULE_2	0.520814	-0.14199	11.83412	2.07E-23	9.71E-23	42.38775	up
GOBP_REG	-0.52078	0.036546	-12.4761	3.73E-25	2.01E-24	46.38388	down
GOCC_RIB	0.520773	0.236168	11.08732	2.19E-21	8.74E-21	37.75236	up
GOBP_XYL	-0.52072	0.217339	-7.10427	4.16E-11	8.39E-11	14.30136	down
REACTOM	-0.52072	0.217339	-7.10427	4.16E-11	8.39E-11	14.30136	down
HP_LACTI(	-0.52069	0.227068	-9.61622	1.89E-17	5.71E-17	28.74588	down
GOBP_MIT	0.52067	-0.10591	12.44226	4.61E-25	2.47E-24	46.17332	up
REACTOM	-0.52063	0.163912	-10.5774	5.18E-20	1.87E-19	34.6059	down
MORF_EIF3	0.520601	0.11545	11.56031	1.15E-22	5.06E-22	40.68561	up
REICHERT_	0.520507	-0.17262	9.690531	1.20E-17	3.69E-17	29.19458	up
ICHIBA_GR	-0.52028	0.055615	-18.1573	3.64E-40	1.34E-38	80.81432	down
GSE27241_	0.520216	-0.13009	13.23641	3.23E-27	2.10E-26	51.11401	up
GOBP_INSI	-0.5202	0.046755	-10.5646	5.61E-20	2.02E-19	34.52673	down
QIU_PBMC	-0.52018	0.127631	-14.6604	4.70E-31	4.60E-30	59.91319	down
MODULE_4	-0.52013	0.169634	-11.4166	2.81E-22	1.20E-21	39.79333	down
GOMF_INS	-0.52002	-0.00961	-9.83712	4.93E-18	1.55E-17	30.08212	down
chr21q21	-0.52	-0.03781	-12.956	1.86E-26	1.14E-25	49.37106	down
GOCC_MH	0.519977	0.013666	8.016397	2.50E-13	5.77E-13	19.34845	up
GOBP_FAT	-0.51977	0.140996	-11.2718	6.93E-22	2.87E-21	38.89484	down
HOELZEL_I	-0.51956	-0.00703	-19.5426	1.30E-43	9.46E-42	88.71806	down
HP_CEREBI	-0.51937	0.034684	-16.518	5.83E-36	1.07E-34	71.16987	down
HALLMARI	0.519345	0.058524	19.80966	2.89E-44	2.48E-42	90.21463	up
GOBP_EPIT	-0.51918	-0.04327	-14.0375	2.21E-29	1.79E-28	56.07755	down
GOCC_EPS	0.519153	0.048859	9.088802	4.57E-16	1.26E-15	25.58777	up
GSE17301_	0.519148	-0.04982	20.98121	4.40E-47	8.72E-45	96.6725	up
GOMF_FO	0.518923	-0.17379	10.80973	1.23E-20	4.64E-20	36.03693	up
GOBP_MIT	0.5188	0.094488	12.22712	1.77E-24	9.02E-24	44.83374	up
ICHIBA_GR	-0.51874	0.127905	-15.0805	3.56E-32	3.96E-31	62.48524	down
GOBP_SH(	-0.51873	0.170589	-9.17098	2.79E-16	7.80E-16	26.07654	down
LAKE_ADU	-0.51861	-0.1088	-19.0278	2.42E-42	1.33E-40	85.80868	down
GOBP_POS	0.518473	-0.15534	8.239871	6.87E-14	1.64E-13	20.62478	up
GOBP_TOL	-0.51846	0.04225	-9.68147	1.27E-17	3.88E-17	29.13983	down
HP_ABNOI	-0.51844	0.140376	-11.8972	1.40E-23	6.64E-23	42.78002	down
HP_ENLAR	0.518166	0.051611	9.075299	4.96E-16	1.36E-15	25.50759	up
GOBP_MA	0.518069	0.013492	11.57674	1.03E-22	4.58E-22	40.78771	up

REACTOM	-0.5178	-0.07163	-14.8436	1.52E-31	1.58E-30	61.03628	down
REACTOM	-0.51777	0.079772	-8.00511	2.66E-13	6.15E-13	19.28434	down
GOBP_POS	-0.51768	-0.10499	-8.96035	9.86E-16	2.65E-15	24.82644	down
HP_ORCHI	-0.51766	0.071181	-11.3991	3.13E-22	1.34E-21	39.68474	down
GOCC_NU	0.517583	-0.01068	11.95251	9.87E-24	4.74E-23	43.12433	up
WAMUNY(	-0.51749	-0.04273	-18.1829	3.14E-40	1.17E-38	80.96225	down
GOBP_POS	0.517377	-0.08067	8.685791	5.04E-15	1.30E-14	23.21088	up
PEDERSEN	-0.51735	0.013268	-20.4506	8.14E-46	1.03E-43	93.7695	down
GSE2405_(	0.517306	0.179642	13.01497	1.29E-26	7.98E-26	49.73764	up
GRAHAM_	-0.51727	0.022607	-15.2895	9.90E-33	1.17E-31	63.75928	down
chr2q24	-0.51723	-0.02248	-16.5793	4.04E-36	7.66E-35	71.53612	down
HP_ABNOI	0.517208	0.245696	11.08565	2.21E-21	8.83E-21	37.74199	up
HP_PURE_I	0.517185	0.317157	9.789231	6.60E-18	2.05E-17	29.79182	up
HP_CUTAN	-0.51715	-0.14449	-13.2627	2.74E-27	1.80E-26	51.27752	down
GOBP_HIS	-0.5171	0.128002	-10.111	9.20E-19	3.04E-18	31.74807	down
REACTOM	0.517069	-0.0084	19.61003	8.89E-44	6.72E-42	89.09688	up
GOCC_TR/	0.517059	-0.03187	8.550873	1.12E-14	2.80E-14	22.42325	up
GOBP_NEL	-0.51705	-0.01549	-9.78933	6.60E-18	2.05E-17	29.7924	down
GOBP_CAF	0.516988	0.021776	12.83308	4.01E-26	2.37E-25	48.60616	up
FURUKAW	0.516987	-0.13619	13.28799	2.34E-27	1.55E-26	51.43441	up
HARALAM	-0.51683	0.002751	-16.3236	1.87E-35	3.19E-34	70.00709	down
HP_CHROI	0.516792	-0.06238	10.07081	1.18E-18	3.86E-18	31.50312	up
chr21q11	-0.51675	-0.02811	-9.10282	4.20E-16	1.16E-15	25.67108	down
GOBP_GLL	-0.51666	0.093633	-13.185	4.45E-27	2.87E-26	50.79463	down
BIOCARTA	-0.51663	0.058352	-15.6081	1.42E-33	1.87E-32	65.69471	down
HP_TRACT	-0.51655	-0.02956	-10.1963	5.44E-19	1.83E-18	32.26901	down
GOBP_REG	-0.51652	0.100971	-9.73028	9.46E-18	2.92E-17	29.43491	down
GOBP_L_SI	-0.51652	0.079138	-9.90409	3.27E-18	1.04E-17	30.48862	down
MIR4662A_	-0.5165	-0.05404	-19.9325	1.45E-44	1.34E-42	90.89996	down
GOMF_AD	-0.51635	0.055334	-10.6282	3.78E-20	1.38E-19	34.91821	down
REACTOM	-0.51635	0.055334	-10.6282	3.78E-20	1.38E-19	34.91821	down
GOBP_CEL	-0.51622	-0.0217	-16.8534	7.85E-37	1.66E-35	73.16727	down
REACTOM	-0.51616	-0.05418	-10.8192	1.16E-20	4.38E-20	36.09509	down
GOBP_NE(	-0.51616	0.058059	-12.8435	3.75E-26	2.23E-25	48.67129	down
GOBP_POS	-0.51616	-0.04729	-12.6052	1.66E-25	9.28E-25	47.18798	down
GOMF_ALI	-0.51608	0.012139	-9.12285	3.73E-16	1.03E-15	25.79014	down
SALVADOI	-0.51603	0.16817	-12.3013	1.11E-24	5.78E-24	45.29566	down
VANHARA	-0.51598	0.036846	-16.4655	7.98E-36	1.44E-34	70.85646	down
GOCC_SPL	0.515933	-0.00103	17.28046	6.22E-38	1.54E-36	75.69301	up
GOBP_CAF	-0.51591	0.018415	-11.0372	2.99E-21	1.18E-20	37.44249	down
HOFFMAN	-0.51585	0.014672	-11.5605	1.14E-22	5.05E-22	40.68695	down
REACTOM	-0.51584	0.035912	-9.79175	6.50E-18	2.02E-17	29.8071	down
GOBP_FLA	-0.5158	0.003201	-8.63345	6.86E-15	1.75E-14	22.9048	down
PID_PLK1_I	0.515782	-0.17298	12.19142	2.22E-24	1.12E-23	44.61146	up
GOBP_POS	-0.51574	-0.03331	-9.1148	3.91E-16	1.08E-15	25.74225	down
GOBP_LEN	-0.51572	0.061073	-9.56119	2.64E-17	7.89E-17	28.41416	down
AMIT_EGF_	-0.51569	0.046048	-10.439	1.22E-19	4.29E-19	33.75551	down
ZAMORA_	0.515616	0.018169	17.01652	2.97E-37	6.70E-36	74.13442	up
MIR6806_5	-0.5156	-0.03157	-20.6698	2.43E-46	3.72E-44	94.97283	down
REACTOM	0.515586	0.070837	14.72337	3.19E-31	3.18E-30	60.29973	up
MODULE_!	-0.51552	0.034666	-10.4057	1.50E-19	5.24E-19	33.55103	down
GOBP_POS	-0.51548	0.046849	-13.0886	8.12E-27	5.13E-26	50.19567	down
WP_ANGI(	-0.51547	-0.18494	-13.9932	2.91E-29	2.34E-28	55.80425	down
GOBP_PRC	-0.51546	-0.1285	-12.6947	9.52E-26	5.44E-25	47.74462	down
GOCC_HFE	-0.51544	-0.01307	-12.5758	2.00E-25	1.11E-24	47.00496	down
GOMF_CH	-0.51543	-0.02567	-11.5394	1.30E-22	5.74E-22	40.55579	down
GOMF_GPI	0.515312	-0.10281	12.98022	1.60E-26	9.84E-26	49.52153	up
HP_ABNOI	-0.51531	-0.0176	-13.538	4.93E-28	3.48E-27	52.98572	down

MIR3664_5	-0.5153	-0.06079	-22.0571	1.33E-49	4.81E-47	102.4488	down
GOBP_POS	-0.51528	0.069479	-7.92703	4.17E-13	9.50E-13	18.84203	down
GOBP_NEG	-0.51526	-0.02371	-12.9631	1.78E-26	1.09E-25	49.41497	down
HP_ADENO	0.515257	0.190298	12.51861	2.86E-25	1.56E-24	46.64867	up
BIOCARTA	-0.51515	0.01909	-16.32	1.91E-35	3.26E-34	69.98577	down
MIR5585_5	-0.51514	-0.06796	-24.857	7.17E-56	1.93E-52	116.7903	down
GOMF_CO	-0.51506	0.083549	-8.46421	1.86E-14	4.59E-14	21.91963	down
HP_CEPHA	-0.51501	-0.06986	-12.1339	3.18E-24	1.58E-23	44.25317	down
HAHTOLA_	-0.51483	0.074588	-15.467	3.35E-33	4.22E-32	64.83891	down
GOBP_REG	-0.51481	0.092702	-11.6988	4.82E-23	2.20E-22	41.54631	down
REACTOM	0.514747	0.057682	14.26464	5.41E-30	4.67E-29	57.47925	up
HP_AMEGA	-0.51474	-0.21038	-11.2188	9.64E-22	3.96E-21	38.56682	down
BIOCARTA	-0.51456	0.077536	-7.54178	3.70E-12	7.95E-12	16.68728	down
REACTOM	-0.51454	0.087557	-14.8996	1.08E-31	1.14E-30	61.37961	down
MIR127_5P	-0.51443	-0.10898	-20.6461	2.77E-46	4.19E-44	94.84291	down
GOBP_REL	-0.51442	0.181632	-9.62449	1.80E-17	5.44E-17	28.79575	down
MIR6824_5	-0.51435	-0.05867	-20.7977	1.20E-46	2.02E-44	95.67262	down
GOBP_SPH	-0.51433	-0.04043	-16.6098	3.36E-36	6.46E-35	71.71771	down
BIOCARTA	-0.51429	0.02632	-16.0439	1.01E-34	1.55E-33	68.32722	down
GOBP_RES	0.514267	-0.08979	8.890789	1.49E-15	3.97E-15	24.4156	up
HP_INCREA	-0.51418	0.013775	-9.32362	1.11E-16	3.19E-16	26.98773	down
MIR6502_5	-0.51413	-0.04819	-21.0594	2.87E-47	5.91E-45	97.0972	down
GOCC_MIT	0.513986	-0.24448	10.97358	4.44E-21	1.73E-20	37.0488	up
BIOCARTA	-0.5139	0.170112	-10.6315	3.71E-20	1.35E-19	34.93881	down
GOMF_S_M	-0.51389	0.133016	-10.1456	7.43E-19	2.47E-18	31.95966	down
GOMF_TAI	0.513884	-0.01142	7.049136	5.62E-11	1.12E-10	14.00568	up
BIOCARTA	-0.51388	-0.03425	-10.4519	1.13E-19	3.97E-19	33.83468	down
TESAR_ALK	0.513873	-0.09443	11.77273	3.04E-23	1.41E-22	42.00596	up
GOBP_GLY	0.513848	0.062883	9.515179	3.50E-17	1.04E-16	28.13715	up
REACTOM	0.513764	0.009645	15.6956	8.34E-34	1.13E-32	66.22465	up
NAKAYAM	0.513733	-0.13719	12.27699	1.30E-24	6.68E-24	45.14427	up
HP_REDUC	-0.5137	-0.13074	-11.3946	3.22E-22	1.37E-21	39.65679	down
GOBP_DEN	-0.51367	-0.04153	-11.6683	5.84E-23	2.64E-22	41.35638	down
GOBP_GLC	-0.51365	-0.04321	-9.77507	7.20E-18	2.23E-17	29.70603	down
MODULE_3	0.513625	-0.12493	14.60095	6.78E-31	6.50E-30	59.54846	up
GOBP_NEG	-0.51358	-0.12006	-10.3129	2.65E-19	9.12E-19	32.98267	down
GOBP_CAL	-0.51357	0.016629	-14.2865	4.73E-30	4.10E-29	57.614	down
HP_ABNOI	-0.5135	0.276086	-7.92699	4.17E-13	9.50E-13	18.8418	down
GOBP_DET	-0.51346	0.04629	-10.5128	7.73E-20	2.76E-19	34.20883	down
GOBP_VEN	-0.51344	0.00652	-15.1748	2.00E-32	2.29E-31	63.06068	down
HP_FASTIN	-0.51343	0.108596	-11.5933	9.32E-23	4.15E-22	40.89031	down
GOBP_NEG	-0.51339	-0.02079	-17.0121	3.05E-37	6.85E-36	74.10845	down
JI_METAST	-0.51339	0.020758	-14.5902	7.25E-31	6.93E-30	59.48228	down
HP_SEGME	-0.51338	0.074169	-11.5773	1.03E-22	4.57E-22	40.79093	down
GOBP_URE	-0.51337	0.126026	-11.153	1.45E-21	5.88E-21	38.15908	down
WRN_TARG	0.513289	-0.00249	11.70568	4.62E-23	2.11E-22	41.58904	up
HP_KETOS	-0.51324	0.089276	-16.3136	1.99E-35	3.37E-34	69.9474	down
MEBARKI_I	0.513166	-0.13376	12.88871	2.83E-26	1.70E-25	48.9523	up
GOCC_SM.	0.513153	0.251085	9.702796	1.12E-17	3.43E-17	29.26872	up
BIOCARTA	-0.5131	-0.05866	-20.8822	7.57E-47	1.36E-44	96.13327	down
HP_ABNOI	0.513063	-0.11219	10.16549	6.58E-19	2.20E-18	32.08086	up
GOMF_MH	-0.51305	0.145779	-7.61948	2.39E-12	5.19E-12	17.11805	down
VILLANUE\	0.513033	-0.16999	11.00225	3.71E-21	1.46E-20	37.22609	up
GOBP_MET	0.513022	-0.09698	18.03537	7.40E-40	2.56E-38	80.10745	up
GOCC_CHI	0.512927	-0.06286	8.634867	6.80E-15	1.73E-14	22.91309	up
MORF_PSM	0.512923	0.114385	13.52231	5.43E-28	3.82E-27	52.88867	up
WP_NANC	-0.51292	0.131195	-9.7209	1.00E-17	3.08E-17	29.3782	down
GOCC_SM.	-0.51289	-0.12539	-11.0862	2.20E-21	8.80E-21	37.74524	down

chr2q34	-0.51273	-0.05984	-11.5965	9.14E-23	4.07E-22	40.91048	down
GOBP_SEN	0.512694	-0.04768	9.070526	5.10E-16	1.40E-15	25.47925	up
REACTOM	-0.51269	0.062903	-12.7086	8.72E-26	5.00E-25	47.83168	down
HP_OROF/	-0.51265	0.025795	-17.8399	2.32E-39	7.39E-38	78.97084	down
MORF_AT(	0.512536	0.130365	12.30918	1.06E-24	5.52E-24	45.34465	up
WACKER_I	-0.51248	0.073202	-10.7435	1.85E-20	6.91E-20	35.62854	down
WP_DEVEL	-0.51235	0.078038	-13.0766	8.75E-27	5.51E-26	50.121	down
GOMF_PH(	-0.51229	-0.0688	-7.16421	3.00E-11	6.10E-11	14.62409	down
REACTOM	0.512263	0.205775	10.62939	3.76E-20	1.37E-19	34.92556	up
BOQUEST_	-0.51222	0.008767	-14.6468	5.11E-31	4.98E-30	59.8301	down
MIR6882_5	-0.51214	-0.10126	-20.767	1.42E-46	2.31E-44	95.50498	down
MIR3914	-0.51211	-0.10231	-20.4044	1.05E-45	1.28E-43	93.51486	down
LEE_LIVER_	-0.51206	0.141455	-9.57762	2.39E-17	7.16E-17	28.51316	down
GSE13547_	0.512053	0.009562	18.26408	1.96E-40	7.53E-39	81.43174	up
GOBP_TRA	-0.51198	-0.1255	-10.9882	4.05E-21	1.59E-20	37.13898	down
REACTOM	0.51197	0.146382	14.6445	5.19E-31	5.04E-30	59.81586	up
REACTOM	0.511925	-0.02664	17.49219	1.78E-38	4.88E-37	76.93766	up
GOBP_SER	-0.51192	0.104785	-10.0586	1.27E-18	4.15E-18	31.42896	down
GOBP_AN	-0.51179	0.054663	-11.7255	4.08E-23	1.87E-22	41.71216	down
GNF2_CEB	-0.51177	0.279171	-8.57396	9.74E-15	2.46E-14	22.5577	down
MATSUMI	-0.51175	0.079744	-9.89712	3.41E-18	1.09E-17	30.44626	down
GOBP_LEU	-0.51163	0.020011	-12.167	2.58E-24	1.30E-23	44.45954	down
SANSOM_	0.511467	0.024016	22.43431	1.79E-50	8.50E-48	104.4387	up
QIU_PBMC	-0.51145	0.054966	-13.4568	8.17E-28	5.64E-27	52.48238	down
MODULE_	0.511442	-0.20886	10.29748	2.92E-19	1.00E-18	32.88794	up
GOBP_POS	-0.51135	0.069301	-13.9609	3.55E-29	2.84E-28	55.6044	down
REACTOM	-0.51134	-0.19476	-12.1324	3.20E-24	1.60E-23	44.24399	down
PIEPOLI_L(	-0.51132	0.015403	-14.6758	4.28E-31	4.20E-30	60.00818	down
TCOF1_TA	0.511198	0.022508	13.21936	3.59E-27	2.33E-26	51.00809	up
MIR7848_3	-0.51119	-0.03138	-17.0493	2.45E-37	5.58E-36	74.3285	down
REACTOM	-0.51117	0.022864	-11.8711	1.64E-23	7.76E-23	42.61783	down
chr13q33	-0.5111	0.0329	-10.5368	6.66E-20	2.38E-19	34.3563	down
LI_PBMC_N	-0.51106	0.108288	-13.7601	1.24E-28	9.33E-28	54.36205	down
GOBP_VIR	-0.51104	0.038613	-10.2623	3.63E-19	1.23E-18	32.6728	down
HP_LARGE	0.511	-0.06381	12.74244	7.06E-26	4.07E-25	48.0421	up
HP_EXSTR(	-0.51081	-0.03472	-11.444	2.37E-22	1.02E-21	39.96353	down
GOBP_POS	-0.51067	-0.04714	-10.4336	1.26E-19	4.43E-19	33.72235	down
GOMF_OX	-0.5105	0.073334	-12.6745	1.08E-25	6.13E-25	47.61894	down
GOBP_POS	-0.51049	-0.00721	-20.0424	7.87E-45	7.83E-43	91.51154	down
GOCC_DYI	0.510464	0.02313	12.20024	2.10E-24	1.06E-23	44.66638	up
GSE15930_	0.510421	0.012413	19.71922	4.81E-44	3.91E-42	89.70885	up
GOBP_INT	0.510421	-0.16578	11.68666	5.20E-23	2.36E-22	41.47076	up
GSE29617_	-0.51037	-0.0369	-17.808	2.79E-39	8.72E-38	78.78497	down
GOBP_REG	-0.51037	-0.05196	-9.08986	4.54E-16	1.25E-15	25.59406	down
LAKE_ADU	-0.51035	-0.11734	-19.2172	8.22E-43	5.05E-41	86.88267	down
WP_OXIDA	-0.51028	0.101167	-12.6721	1.10E-25	6.22E-25	47.60432	down
SHANK_TA	-0.51021	0.139093	-10.8323	1.07E-20	4.05E-20	36.17649	down
HOEK_PBN	-0.51012	0.144796	-12.3947	6.21E-25	3.29E-24	45.87693	down
GOBP_T_H	-0.51006	0.130046	-11.8611	1.75E-23	8.24E-23	42.55536	down
GOMF_HEI	-0.51001	0.154589	-8.76031	3.24E-15	8.44E-15	23.64773	down
GINESTIER	-0.50999	-0.10091	-15.5232	2.38E-33	3.06E-32	65.17988	down
KORKOLA_	-0.50981	0.064551	-12.2229	1.82E-24	9.26E-24	44.80752	down
BIOCARTA	-0.50961	-0.12105	-17.2624	6.92E-38	1.71E-36	75.58641	down
GOBP_REG	-0.50955	-0.02771	-11.9764	8.50E-24	4.11E-23	43.27316	down
GOBP_DOI	-0.50951	0.020292	-12.1389	3.08E-24	1.54E-23	44.28431	down
GOBP_ADE	-0.5095	-0.01537	-15.6182	1.33E-33	1.76E-32	65.75595	down
MIR3074_3	-0.50943	-0.10528	-18.9126	4.67E-42	2.43E-40	85.15308	down
REACTOM	0.509421	-0.0184	10.79182	1.37E-20	5.17E-20	35.92643	up

GOMF_C_(	-0.50935	0.006666	-9.77528	7.19E-18	2.23E-17	29.7073	down
MIR3184_3	-0.50924	-0.08229	-19.9613	1.24E-44	1.16E-42	91.06014	down
GSE37605_	-0.50918	0.00763	-19.3801	3.26E-43	2.16E-41	87.80312	down
GOBP_MRI	0.509127	0.007378	9.036152	6.27E-16	1.70E-15	25.27533	up
DURANTE_	0.509067	0.082371	15.13404	2.56E-32	2.91E-31	62.81188	up
GOMF_TEL	-0.50901	0.027524	-10.2767	3.32E-19	1.13E-18	32.76087	down
MIR4696	-0.50885	-0.08101	-20.3729	1.25E-45	1.47E-43	93.34102	down
HAN_JNK_	-0.50883	0.057936	-14.2777	4.99E-30	4.32E-29	57.55976	down
HALLMARI	-0.50882	0.090486	-11.6962	4.90E-23	2.23E-22	41.5304	down
MODULE_1	0.508799	-0.13794	11.55285	1.20E-22	5.29E-22	40.63924	up
GOMF_FAT	-0.50879	0.122039	-11.5094	1.57E-22	6.88E-22	40.36926	down
GOBP_ATF	-0.50862	-0.02115	-9.94556	2.54E-18	8.14E-18	30.74061	down
GOBP_SNF	0.50859	0.049971	10.95065	5.12E-21	1.99E-20	36.90708	up
GOBP_POS	0.508582	-0.19636	7.600857	2.65E-12	5.75E-12	17.01461	up
GOBP_NEG	-0.50856	0.038577	-14.3532	3.13E-30	2.78E-29	58.02477	down
FRASOR_R	-0.50845	-0.15572	-12.8414	3.80E-26	2.26E-25	48.65792	down
GOBP_VAS	-0.50842	0.021924	-15.85	3.27E-34	4.71E-33	67.15835	down
HP_HYPER	-0.5084	0.013525	-14.9993	5.85E-32	6.36E-31	61.98889	down
REACTOM	0.508379	0.055379	16.44068	9.27E-36	1.66E-34	70.70792	up
LAKE_ADU	-0.50834	-0.10273	-17.5145	1.56E-38	4.32E-37	77.06858	down
GOBP_MO	-0.50833	-0.01134	-15.045	4.42E-32	4.85E-31	62.26851	down
MODULE_1	-0.50832	0.081224	-12.4769	3.72E-25	2.00E-24	46.3887	down
GOBP_BILE	-0.50829	0.051766	-9.61611	1.89E-17	5.71E-17	28.74521	down
FUJII_YBX1	0.508217	-0.14703	12.82445	4.23E-26	2.50E-25	48.55248	up
REACTOM	-0.50819	-0.06419	-15.9896	1.40E-34	2.12E-33	68.00026	down
HP_BROAD	-0.50805	-0.08349	-13.7144	1.64E-28	1.22E-27	54.07931	down
GOBP_EST.	0.508047	0.054134	13.28722	2.35E-27	1.55E-26	51.42963	up
GOCC_NU	0.507976	-0.04526	17.97296	1.07E-39	3.58E-38	79.74496	up
WP_ETHAN	-0.50784	-0.03798	-13.2969	2.21E-27	1.47E-26	51.48955	down
HP_RETINA	-0.5078	0.168342	-9.3149	1.17E-16	3.36E-16	26.93559	down
GOBP_COI	-0.50777	-0.06519	-9.82493	5.31E-18	1.66E-17	30.00818	down
NAGY_STA	0.507743	-0.05812	12.90715	2.52E-26	1.52E-25	49.06697	up
GOBP_REG	-0.50773	-0.01476	-11.8554	1.81E-23	8.53E-23	42.52037	down
MODULE_3	0.50765	0.056437	10.339	2.26E-19	7.81E-19	33.14226	up
chr3q28	-0.50752	0.018917	-10.3257	2.45E-19	8.46E-19	33.06065	down
HP_ABNOI	-0.50749	0.017837	-12.9797	1.60E-26	9.87E-26	49.5181	down
DESCARTE	-0.5074	0.027405	-10.7903	1.39E-20	5.21E-20	35.91703	down
GOBP_PLA	-0.50737	0.025539	-15.9022	2.38E-34	3.49E-33	67.47337	down
HALLMARI	-0.50702	0.044544	-13.1702	4.88E-27	3.13E-26	50.70258	down
GOBP_PRC	-0.50685	-0.11082	-11.5648	1.11E-22	4.92E-22	40.71326	down
ERWIN_CC	-0.50672	-0.04737	-16.1911	4.16E-35	6.68E-34	69.21192	down
HP_METAF	0.50664	-0.15255	11.39473	3.22E-22	1.37E-21	39.65756	up
REACTOM	-0.50659	-0.03228	-14.4572	1.65E-30	1.51E-29	58.66518	down
ZERBINI_RI	-0.50651	0.114823	-10.339	2.26E-19	7.81E-19	33.14231	down
HP_HYPOG	0.50627	-0.07559	11.17886	1.24E-21	5.04E-21	38.31915	up
GOBP_REG	-0.5062	0.043162	-14.8498	1.47E-31	1.52E-30	61.07438	down
GOBP_MIT	0.50619	-0.18664	9.824576	5.32E-18	1.67E-17	30.00604	up
MORF_EIF3	0.506126	0.035787	13.84202	7.44E-29	5.74E-28	54.86926	up
GOBP_REG	-0.50612	-0.04652	-12.4868	3.49E-25	1.89E-24	46.45064	down
GOBP_EPIT	-0.50611	-0.01961	-14.2427	6.20E-30	5.32E-29	57.344	down
GOBP_NEG	-0.50607	-0.09886	-10.3438	2.19E-19	7.59E-19	33.17182	down
GOBP_REG	-0.50603	0.030183	-16.0015	1.31E-34	1.99E-33	68.07173	down
GOBP_POS	-0.50599	0.017825	-9.64277	1.61E-17	4.88E-17	28.90606	down
PID_IFNG_	-0.50587	-0.08364	-18.1741	3.30E-40	1.23E-38	80.91148	down
GOBP_REG	-0.50584	0.005958	-13.8882	5.58E-29	4.36E-28	55.1549	down
BUSSLINGI	-0.50571	0.02262	-19.6251	8.17E-44	6.25E-42	89.18134	down
REACTOM	0.505566	0.036735	14.647	5.11E-31	4.97E-30	59.83117	up
GOCC_ROI	0.505513	0.108497	14.88833	1.16E-31	1.21E-30	61.31037	up

HP_HAMA	-0.50548	-0.06282	-12.6605	1.18E-25	6.66E-25	47.53202	down
GOBP_LIPI	-0.50543	0.013887	-17.9507	1.21E-39	4.03E-38	79.61532	down
CHEN_PD(	-0.50539	-0.00292	-12.6843	1.01E-25	5.78E-25	47.68042	down
DESCARTE	-0.50527	0.006669	-15.8231	3.85E-34	5.49E-33	66.99564	down
GOBP_FLA	-0.50524	-0.05412	-7.90374	4.76E-13	1.08E-12	18.71049	down
BASAKI_YE	0.50524	-0.05334	16.95696	4.24E-37	9.31E-36	73.78164	up
REACTOM	0.505195	-0.07561	14.39231	2.46E-30	2.21E-29	58.26567	up
GOCC_TR/	0.505178	-0.12379	12.98576	1.54E-26	9.51E-26	49.55599	up
GOMF_CEI	0.505077	-0.19032	11.75067	3.49E-23	1.61E-22	41.86878	up
GOBP_REG	-0.50507	-0.12175	-9.07974	4.83E-16	1.32E-15	25.53395	down
CHUNG_BI	-0.50505	-0.04562	-15.1125	2.92E-32	3.30E-31	62.68031	down
REACTOM	-0.50503	0.143332	-10.4544	1.11E-19	3.91E-19	33.84996	down
GOBP_POS	-0.50495	-0.04561	-19.3351	4.21E-43	2.72E-41	87.5492	down
BIOCARTA	-0.50495	0.067402	-11.2418	8.35E-22	3.44E-21	38.70941	down
REACTOM	0.504938	0.142141	11.64135	6.90E-23	3.11E-22	41.18918	up
WINZEN_D	-0.50492	0.001791	-17.4523	2.26E-38	6.09E-37	76.70369	down
XU_HGF_SI	0.504911	-0.12925	12.5861	1.88E-25	1.04E-24	47.06891	up
GOBP_CLE	0.504898	0.033818	11.30143	5.76E-22	2.40E-21	39.07882	up
MIR6074	-0.50486	-0.08995	-21.1271	1.99E-47	4.19E-45	97.4645	down
NGO_MAL	0.504809	0.038752	10.95884	4.86E-21	1.89E-20	36.95774	up
GOBP_FAT	-0.50478	0.143741	-10.7518	1.76E-20	6.57E-20	35.6799	down
PID_HIF1_T	-0.50477	-0.00661	-20.1142	5.27E-45	5.37E-43	91.90985	down
HP_HEAT_I	-0.50477	-0.0238	-14.7052	3.57E-31	3.53E-30	60.18857	down
WP_IRINO	-0.50474	0.023559	-10.0092	1.72E-18	5.57E-18	31.12754	down
GOBP_AG(	-0.50474	0.096228	-12.3531	8.06E-25	4.23E-24	45.61788	down
AIZARANI_	-0.50471	0.021259	-11.8958	1.41E-23	6.69E-23	42.77119	down
WP_RAS_A	-0.50451	0.016462	-16.3246	1.86E-35	3.18E-34	70.01328	down
MIR4315	0.504498	-0.20483	8.24252	6.76E-14	1.62E-13	20.63999	up
HALLMARI	-0.5045	-0.03923	-16.8171	9.75E-37	2.04E-35	72.95164	down
GOCC_TR(	0.504476	0.081469	9.571969	2.48E-17	7.41E-17	28.47908	up
NIKOLSKY_	0.504473	0.022022	11.71022	4.49E-23	2.05E-22	41.61725	up
REACTOM	0.504244	-0.07132	12.12764	3.30E-24	1.64E-23	44.21439	up
REACTOM	0.504162	-0.1295	8.334419	3.96E-14	9.61E-14	21.1689	up
chr6q12	-0.50399	-0.01847	-8.07767	1.76E-13	4.09E-13	19.697	down
MEISSNER_	0.503912	0.055988	15.50144	2.72E-33	3.46E-32	65.04782	up
GOBP_NAI	0.503879	-0.09623	10.58338	4.99E-20	1.80E-19	34.64251	up
PLASARI_T	-0.50387	0.030865	-8.86036	1.79E-15	4.73E-15	24.23622	down
GOMF_INH	-0.50368	-0.03897	-8.67971	5.22E-15	1.34E-14	23.17528	down
HASEGAW	-0.50363	0.106264	-9.93984	2.63E-18	8.42E-18	30.70587	down
MIR656_5F	-0.50363	-0.07148	-21.2723	9.02E-48	2.17E-45	98.24986	down
DESCARTE	-0.5036	0.152364	-11.4029	3.06E-22	1.31E-21	39.70813	down
CHIANG_L	-0.50346	0.126596	-9.81632	5.59E-18	1.75E-17	29.95599	down
DELPUECH	0.503361	-0.07186	17.62558	8.14E-39	2.34E-37	77.71922	up
GSE24634_	0.503339	-0.08596	13.25161	2.94E-27	1.92E-26	51.20844	up
MORF_MT	0.503329	0.026794	15.63115	1.23E-33	1.63E-32	65.83436	up
HP_RIGHT_	-0.50331	-0.0825	-10.3609	1.98E-19	6.85E-19	33.27623	down
REACTOM	0.503297	-0.03147	16.64972	2.65E-36	5.15E-35	71.95585	up
MIKHAYLC	0.503269	-0.05752	9.076569	4.92E-16	1.35E-15	25.51513	up
HP_ABNOI	-0.50318	0.054445	-14.5157	1.15E-30	1.07E-29	59.02496	down
MATZUK_(	-0.50312	0.057735	-10.3199	2.54E-19	8.76E-19	33.02493	down
HP_CARDI	-0.50311	0.03855	-11.8834	1.52E-23	7.21E-23	42.69461	down
HP_ABNOI	-0.50305	-0.19462	-11.1534	1.45E-21	5.87E-21	38.16158	down
GOCC_REF	0.503032	-0.11275	13.76045	1.23E-28	9.31E-28	54.36441	up
GAVIN_FO	0.502995	-0.09769	13.25084	2.95E-27	1.93E-26	51.20368	up
REACTOM	0.502978	-0.01455	10.10767	9.39E-19	3.10E-18	31.72795	up
FUNG_IL2_	-0.50296	-0.03232	-10.2394	4.18E-19	1.41E-18	32.53277	down
GOBP_HUI	-0.50294	0.171487	-11.3132	5.35E-22	2.24E-21	39.15162	down
GOBP_REG	-0.5029	0.04451	-10.0057	1.75E-18	5.69E-18	31.1068	down

TIMOFEEV.	0.502848	0.107208	12.25751	1.47E-24	7.51E-24	45.02298	up
MODULE_:	0.502826	0.342763	7.815063	7.90E-13	1.77E-12	18.21097	up
GOBP_AM	-0.5028	0.06197	-15.3667	6.18E-33	7.52E-32	64.22931	down
GHANDHI_	-0.50275	0.048913	-15.1221	2.76E-32	3.12E-31	62.7391	down
MIR621	-0.50271	-0.08541	-17.5207	1.51E-38	4.18E-37	77.10506	down
GOBP_POS	-0.5027	0.059658	-8.7403	3.65E-15	9.47E-15	23.53029	down
GOCC_MIC	-0.50267	0.142717	-9.70342	1.11E-17	3.42E-17	29.27247	down
HAHTOLA_	-0.50267	0.042829	-12.2362	1.67E-24	8.54E-24	44.8905	down
MANALO_	0.502544	-0.06355	14.96006	7.45E-32	8.01E-31	61.74923	up
GGAANCG	0.502505	0.063118	17.26236	6.92E-38	1.71E-36	75.58638	up
GOBP_REG	-0.5024	0.158825	-12.4438	4.57E-25	2.45E-24	46.18293	down
GOBP_POS	-0.50227	-0.01089	-17.1574	1.29E-37	3.05E-36	74.96706	down
REACTOM	0.50225	0.19263	9.557925	2.70E-17	8.04E-17	28.39447	up
STK33_UP	-0.50223	-0.03735	-18.3354	1.30E-40	5.13E-39	81.84358	down
EPPERT_HS	-0.50222	-0.04905	-19.1528	1.19E-42	7.01E-41	86.51797	down
GOMF_D_I	0.502221	-0.26751	8.325411	4.18E-14	1.01E-13	21.11696	up
GOBP_ARC	-0.50219	0.096643	-11.5347	1.34E-22	5.91E-22	40.52635	down
GOMF_CA	-0.50214	0.068661	-12.5065	3.09E-25	1.68E-24	46.57335	down
MODULE_!	0.50211	0.225522	8.817076	2.31E-15	6.08E-15	23.98134	up
ZZZ3_TAR(	0.502068	-0.00899	20.77525	1.36E-46	2.24E-44	95.54987	up
MODULE_:	-0.50201	0.155541	-10.1428	7.57E-19	2.51E-18	31.94205	down
REACTOM	0.501784	0.053314	10.93043	5.80E-21	2.24E-20	36.78216	up
GOCC_DYS	-0.50176	0.018426	-10.7879	1.41E-20	5.29E-20	35.90224	down
ZNF19_TAI	0.501697	-0.07847	12.12185	3.42E-24	1.70E-23	44.17838	up
VARELA_ZI	-0.50166	0.164716	-11.2601	7.45E-22	3.08E-21	38.8225	down
HP_BREAS	0.501658	-0.16747	14.55136	9.21E-31	8.68E-30	59.2438	up
MASRI_RES	-0.50164	0.005521	-13.3795	1.32E-27	8.95E-27	52.00241	down
GOBP_CH(	-0.50161	5.90E-05	-9.85322	4.46E-18	1.41E-17	30.17979	down
GOMF_CIL	-0.50159	-0.07228	-11.5839	9.88E-23	4.39E-22	40.83236	down
GOBP_PEP	-0.50157	-0.07435	-11.2923	6.10E-22	2.54E-21	39.02207	down
RUVBL2_T/	0.501539	-0.06625	14.63755	5.41E-31	5.25E-30	59.77319	up
WP_SIGNA	-0.50154	-0.11409	-19.0868	1.73E-42	9.86E-41	86.14357	down
REACTOM	-0.50152	-0.06922	-14.6791	4.19E-31	4.12E-30	60.02807	down
REACTOM	0.501501	-0.0748	16.64006	2.81E-36	5.43E-35	71.8983	up
GOBP_NE(	-0.5014	-0.06974	-10.665	3.01E-20	1.10E-19	35.14475	down
GOBP_RES	-0.50132	-0.0823	-14.1057	1.45E-29	1.20E-28	56.49898	down
HP_INCRE/	-0.50127	0.12121	-9.87681	3.86E-18	1.22E-17	30.32297	down
HP_ABNOI	-0.50121	-0.0088	-16.7654	1.33E-36	2.72E-35	72.64467	down
WONG_EN	-0.50099	0.029228	-10.8934	7.30E-21	2.80E-20	36.55352	down
LU_AGING_	-0.50095	-0.0363	-21.2036	1.31E-47	2.94E-45	97.87841	down
TOMLINS_	0.500847	-0.02988	13.73159	1.48E-28	1.11E-27	54.18567	up
GSE5142_I	0.500695	0.018098	18.44394	6.91E-41	2.92E-39	82.46886	up
BILD_CTNI	-0.50067	-0.16428	-14.8616	1.36E-31	1.42E-30	61.14676	down
HP_LARGE_	-0.50061	0.023925	-12.2919	1.18E-24	6.12E-24	45.23717	down
HOEGERK(	-0.50059	-0.02209	-15.4301	4.20E-33	5.24E-32	64.61426	down
GOBP_REG	0.500532	-0.13228	9.898288	3.39E-18	1.08E-17	30.45336	up
GOBP_NE(	0.500374	-0.06155	8.525294	1.30E-14	3.25E-14	22.27441	up
HP_THOR/	-0.50035	0.008152	-9.16921	2.82E-16	7.88E-16	26.066	down
HP_ALVEO	-0.50034	0.130551	-9.45013	5.19E-17	1.52E-16	27.74618	down
HP_HYPER	-0.50031	0.061031	-11.1171	1.82E-21	7.31E-21	37.93659	down
GOBP_POS	-0.50021	0.081045	-10.7799	1.48E-20	5.55E-20	35.85309	down
MAHADEV	-0.50018	0.059237	-13.4851	6.85E-28	4.76E-27	52.65813	down
PEDERSEN	-0.50017	-0.01843	-13.1363	6.03E-27	3.84E-26	50.49175	down
WP_NCRN	-0.50014	-0.07083	-12.7837	5.45E-26	3.19E-25	48.29867	down
TRAVAGLII	-0.50013	0.132153	-9.76309	7.74E-18	2.40E-17	29.63349	down
GARGALO'	-0.50008	-0.07447	-11.4473	2.32E-22	1.00E-21	39.98388	down
GOBP_PRC	0.500074	-0.07285	7.306628	1.37E-11	2.84E-11	15.39632	up
LANDIS_BI	-0.50006	0.045264	-13.5448	4.72E-28	3.34E-27	53.02802	down

SAENZ_DE	-0.50006	0.101993	-9.4768	4.41E-17	1.30E-16	27.90635	down

	logFC	AveExpr	t	P.Value	adj.P.Val	B	group
REACTOM	-1.37549	-0.11616	-35.6885	1.29E-73	7.19E-71	157.6197	down
GOMF_ALI	-1.32559	-0.09943	-29.0017	3.60E-62	5.35E-60	131.3325	down
HP_DECRE	-1.31333	-0.0609	-36.9188	1.55E-75	1.54E-72	162.0242	down
GOBP_SUC	-1.30746	-0.12599	-30.0924	3.66E-64	6.49E-62	135.9144	down
GOBP_SHC	-1.30717	-0.12162	-36.6886	3.52E-75	3.07E-72	161.2094	down
REACTOM	-1.30635	-0.12654	-31.9514	1.93E-67	4.86E-65	143.4475	down
GOBP_TYR	-1.30176	-0.1924	-36.4266	8.97E-75	6.73E-72	160.2768	down
REACTOM	-1.30176	-0.1924	-36.4266	8.97E-75	6.73E-72	160.2768	down
REACTOM	-1.2815	-0.10672	-36.1004	2.90E-74	1.87E-71	159.1079	down
GOMF_FA	-1.2781	-0.12036	-36.9145	1.57E-75	1.54E-72	162.0092	down
GOMF_MC	-1.27746	-0.12279	-34.2168	3.02E-71	1.23E-68	152.1859	down
GOCC_ME	-1.27433	-0.08259	-36.352	1.17E-74	8.22E-72	160.0104	down
GOBP_FAT	-1.27214	-0.11091	-36.3241	1.30E-74	8.90E-72	159.9105	down
GOBP_ALA	-1.25945	-0.15163	-31.4008	1.74E-66	3.83E-64	141.2513	down
BOYAULT_	-1.25873	-0.12911	-54.0039	8.36E-98	2.70E-93	212.8363	down
GOBP_L_K	-1.25815	-0.03631	-29.851	1.00E-63	1.71E-61	134.9108	down
REACTOM	-1.25507	-0.08224	-35.9006	5.98E-74	3.51E-71	158.3879	down
REACTOM	-1.25221	-0.05051	-33.906	9.78E-71	3.85E-68	151.0142	down
BOYAULT_	-1.25165	-0.08091	-33.2401	1.25E-69	4.11E-67	148.4754	down
GOBP_LEU	-1.24992	-0.06868	-31.9333	2.07E-67	5.11E-65	143.3758	down
CROSBY_E	1.249222	-0.02591	22.91929	5.14E-50	2.40E-48	103.3563	up
MINGUEZ_	-1.24786	-0.1279	-44.6655	1.52E-86	7.00E-83	187.2104	down
GNF2_IGF1	-1.24577	-0.11364	-47.0087	1.49E-89	1.20E-85	194.0717	down
HP_ELEVA	-1.24326	-0.15069	-26.6155	1.28E-57	1.22E-55	120.8653	down
HP_INCRE	-1.23607	-0.08605	-28.8223	7.75E-62	1.10E-59	130.5673	down
WP_FATTY	-1.23498	-0.12475	-37.6317	1.26E-76	1.57E-73	164.5213	down
GOBP_MO	-1.23168	-0.08391	-31.2672	2.99E-66	6.30E-64	140.7141	down
REACTOM	-1.23012	-0.12305	-26.9692	2.60E-58	2.60E-56	122.4565	down
GOMF_AC	-1.22989	-0.08111	-24.0807	1.76E-52	1.03E-50	109.0349	down
REACTOM	-1.22989	-0.10354	-23.6483	1.43E-51	7.79E-50	106.9401	down
HP_RECUR	-1.22913	-0.05462	-32.811	6.57E-69	1.93E-66	146.8185	down
CAR_IGFBI	-1.22852	-0.10805	-40.8725	2.25E-81	6.60E-78	175.3941	down
GOMF_AC	-1.22612	-0.07963	-25.424	3.03E-55	2.35E-53	115.399	down
GNF2_CYP	-1.22542	-0.13211	-43.5781	4.21E-85	1.51E-81	183.916	down
GOBP_LEU	-1.22069	-0.13904	-29.3152	9.51E-63	1.48E-60	132.6621	down
GOMF_LEU	-1.22069	-0.13904	-29.3152	9.51E-63	1.48E-60	132.6621	down
WP_ARYLA	-1.21953	-0.09658	-31.3901	1.82E-66	3.97E-64	141.2087	down
HP_HYPER	-1.21882	-0.12175	-26.7649	6.51E-58	6.31E-56	121.5389	down
REACTOM	-1.2173	-0.10194	-23.4631	3.53E-51	1.84E-49	106.0361	down
MODULE_	-1.21597	-0.12388	-38.4746	6.83E-78	9.18E-75	167.4234	down
HP_ABNOI	-1.21419	-0.15639	-29.5677	3.28E-63	5.42E-61	133.7255	down
BIOCARTA	-1.21131	-0.04504	-39.1524	6.80E-79	1.10E-75	169.7183	down
MODULE_	-1.20947	-0.12602	-37.6932	1.02E-76	1.31E-73	164.7349	down
MONTERC	1.209419	0.004644	24.77392	6.42E-54	4.34E-52	112.3462	up
CHIANG_L	-1.20774	-0.15224	-46.2972	1.18E-88	7.63E-85	192.0215	down
ZNF211_T	1.207691	0.037097	22.81567	8.60E-50	3.94E-48	102.8416	up
GOMF_LIG	-1.20669	-0.10545	-25.4547	2.63E-55	2.05E-53	115.5415	down
REACTOM	-1.20621	-0.07037	-34.7997	3.41E-72	1.59E-69	154.3602	down
REACTOM	-1.20544	-0.02033	-33.4738	5.08E-70	1.78E-67	149.3709	down
FINETTI_BI	1.204672	0.000961	24.51257	2.22E-53	1.44E-51	111.1047	up
GOMF_ES	-1.20444	-0.06678	-27.9835	2.92E-60	3.50E-58	126.9425	down
GOMF_C_	-1.20227	-0.1045	-24.6819	9.93E-54	6.60E-52	111.9098	down
REACTOM	-1.20178	-0.11965	-31.6308	6.93E-67	1.60E-64	142.1723	down
GNF2_MKI	1.198831	-0.01839	21.78644	1.53E-47	5.53E-46	97.65537	up
REACTOM	-1.19786	-0.06698	-34.6687	5.55E-72	2.49E-69	153.8741	down
WP_NICO	-1.19553	-0.1012	-33.0692	2.41E-69	7.49E-67	147.8175	down
CAR_HPX	-1.19486	-0.117	-39.3016	4.11E-79	7.29E-76	170.2191	down

HOWARD_	1.194634	-0.01018	23.72068	1.00E-51	5.53E-50	107.2926	up
GOBP_OM	-1.19146	-0.07297	-36.6203	4.49E-75	3.71E-72	160.9667	down
REACTOM	-1.19146	-0.07297	-36.6203	4.49E-75	3.71E-72	160.9667	down
GOBP_CAF	-1.19111	-0.06139	-24.7311	7.86E-54	5.27E-52	112.1435	down
GOMF_AC	-1.18899	-0.10059	-30.5218	6.22E-65	1.18E-62	137.6845	down
GNF2_RRM	1.188899	-0.01601	22.46728	4.90E-49	2.04E-47	101.101	up
HP_LOW_F	-1.18554	-0.08976	-23.1708	1.48E-50	7.24E-49	104.6004	down
GOMF_AL(	-1.18291	-0.09956	-21.1487	4.07E-46	1.30E-44	94.3751	down
GOBP_LEU	-1.18124	-0.13367	-29.4017	6.60E-63	1.06E-60	133.0271	down
EGUCHI_CI	1.181231	-0.00111	23.34876	6.18E-51	3.14E-49	105.4756	up
GNF2_HMI	1.180775	-0.01609	22.40008	6.87E-49	2.82E-47	100.7635	up
KUMAMOT	1.179617	-0.04789	20.05452	1.27E-43	3.29E-42	88.62623	up
GOCC_MIT	-1.17813	-0.08558	-25.4719	2.42E-55	1.91E-53	115.6216	down
REACTOM	-1.17789	-0.1312	-26.3992	3.41E-57	3.14E-55	119.8852	down
WP_EICOS	-1.17455	-0.0749	-28.5981	2.03E-61	2.73E-59	129.6057	down
GNF2_CCN	1.173767	-0.01461	21.94546	6.83E-48	2.55E-46	98.4654	up
GNF2_TTK	1.173175	-0.00696	22.69462	1.57E-49	6.89E-48	102.2385	up
BIOCARTA	-1.1726	-0.08319	-31.4165	1.64E-66	3.66E-64	141.3146	down
GNF2_CDC	1.171854	-0.01439	22.15674	2.34E-48	9.22E-47	99.53664	up
HP_PROTE	-1.17088	-0.10899	-21.6259	3.49E-47	1.22E-45	96.83437	down
GOBP_LIPI	-1.17055	-0.07946	-28.2475	9.24E-61	1.17E-58	128.0916	down
RICHERT_F	-1.1702	0.038446	-20.0082	1.63E-43	4.18E-42	88.37944	down
HP_ABNOI	-1.17009	-0.13919	-27.2967	6.02E-59	6.41E-57	123.9175	down
AIZARANI_	-1.16768	-0.11562	-40.1034	2.82E-80	7.00E-77	172.8819	down
AIZARANI_	-1.16656	-0.1282	-40.0087	3.86E-80	8.90E-77	172.5699	down
GNF2_H2A	1.166477	-0.01354	22.04262	4.17E-48	1.60E-46	98.95868	up
GNF2_CEB	-1.16631	-0.13482	-36.8276	2.14E-75	1.98E-72	161.7018	down
HP_ABNOI	-1.16405	-0.12844	-31.144	4.91E-66	1.02E-63	140.2173	down
GOMF_3_H	-1.16327	-0.15023	-25.165	1.02E-54	7.38E-53	114.1887	down
GNF2_CCN	1.163194	-0.01432	22.21655	1.73E-48	6.88E-47	99.83888	up
GNF2_GST	-1.16251	-0.13898	-36.866	1.87E-75	1.78E-72	161.8378	down
LOPEZ_ME	-1.16126	-0.10633	-26.7615	6.61E-58	6.38E-56	121.5238	down
GOBP_GLY	-1.15941	-0.12395	-31.2866	2.76E-66	5.90E-64	140.7923	down
GNF2_HPN	-1.15739	-0.13665	-36.9851	1.23E-75	1.28E-72	162.2582	down
GOMF_AL(	-1.15547	-0.11185	-25.2783	5.98E-55	4.46E-53	114.7188	down
CAIRO_HE	-1.15547	-0.07229	-51.064	1.85E-94	2.98E-90	205.2421	down
REACTOM	-1.15482	-0.05165	-34.8652	2.67E-72	1.27E-69	154.6025	down
GNF2_HPX	-1.15462	-0.13263	-35.5263	2.34E-73	1.26E-70	157.0296	down
WP_MOLY	-1.15403	-0.15028	-27.1142	1.36E-58	1.38E-56	123.1048	down
WP_SYNTH	-1.15392	-0.0641	-25.42	3.08E-55	2.38E-53	115.3804	down
GNF2_TST	-1.1538	-0.13594	-35.91	5.78E-74	3.51E-71	158.4217	down
GOCC_MIT	-1.15293	-0.08125	-23.2962	8.00E-51	3.99E-49	105.2177	down
GNF2_CEN	1.151208	-0.01883	21.53362	5.60E-47	1.91E-45	96.36106	up
GOCC_CO	1.151106	-0.017	26.20459	8.27E-57	7.35E-55	118.9986	up
GNF2_LCA	-1.15039	-0.13626	-35.7465	1.05E-73	6.03E-71	157.8301	down
REACTOM	-1.15033	-0.13337	-22.645	2.01E-49	8.72E-48	101.9907	down
HP_RESPIR	-1.15021	-0.12174	-22.1463	2.47E-48	9.66E-47	99.48377	down
GNF2_CDC	1.149811	-0.01984	21.70988	2.27E-47	8.04E-46	97.26428	up
GNF2_BUB	1.148117	-0.02522	21.70806	2.29E-47	8.10E-46	97.25498	up
BOYAULT_	-1.14808	-0.15504	-34.4078	1.47E-71	6.17E-69	152.9016	down
WP_BIOTIN	-1.14765	-0.11487	-26.6121	1.30E-57	1.24E-55	120.8501	down
GNF2_ESPI	1.147519	-0.02148	20.82151	2.24E-45	6.69E-44	92.67178	up
AIZARANI_	-1.1464	-0.11741	-37.0911	8.43E-76	9.38E-73	162.6313	down
GNF2_CEN	1.145963	-0.02462	21.04844	6.86E-46	2.14E-44	93.85445	up
GNF2_SM(	1.145894	-0.01827	21.36959	1.30E-46	4.29E-45	95.51692	up
GOBP_ISO	-1.1446	-0.12432	-27.0477	1.83E-58	1.86E-56	122.8077	down
GOBP_XYL	-1.14452	-0.17645	-22.7403	1.25E-49	5.58E-48	102.4662	down
REACTOM	-1.14452	-0.17645	-22.7403	1.25E-49	5.58E-48	102.4662	down

DESERT_PE	-1.14318	-0.0955	-42.4953	1.24E-83	3.99E-80	180.5628	down
GOMF_EN(	-1.14161	-0.15384	-28.1356	1.50E-60	1.86E-58	127.6055	down
HP_ABNOI	-1.14125	-0.10355	-22.6427	2.04E-49	8.81E-48	101.9793	down
GOMF_AR/	-1.13983	-0.09549	-34.9076	2.28E-72	1.10E-69	154.7593	down
GOMF_DN	1.139146	0.038555	25.19448	8.85E-55	6.49E-53	114.3267	up
REACTOM	-1.13853	-0.11689	-26.1026	1.32E-56	1.15E-54	118.5324	down
GOBP_QUI	-1.13614	-0.10727	-23.9945	2.67E-52	1.55E-50	108.6194	down
WP_VALPF	-1.13503	-0.11646	-33.5895	3.27E-70	1.18E-67	149.8126	down
HP_WIDEN	-1.13466	-0.07593	-17.4569	1.95E-37	3.18E-36	74.37922	down
GOBP_COI	-1.13387	-0.01372	-35.0567	1.31E-72	6.43E-70	155.3094	down
GNF2_PCN	1.133192	-0.01389	22.21967	1.70E-48	6.78E-47	99.85462	up
GOMF_AR/	-1.1312	-0.07151	-31.7468	4.36E-67	1.03E-64	142.6349	down
WOO_LIVE	-1.12987	-0.1413	-32.3655	3.76E-68	1.03E-65	145.0801	down
HP_GLUTA	-1.12929	-0.15477	-23.6332	1.54E-51	8.35E-50	106.8668	down
GOCC_CO	1.129201	0.012376	22.70533	1.49E-49	6.55E-48	102.2919	up
HP_ETHYLI	-1.12871	-0.10982	-28.9963	3.68E-62	5.45E-60	131.3094	down
LY_AGING_	1.128541	-0.00752	21.94534	6.83E-48	2.55E-46	98.46479	up
ZHAN_MU	1.128361	-0.02413	23.13904	1.73E-50	8.42E-49	104.4436	up
GOMF_UBI	-1.12697	-0.09347	-24.4199	3.45E-53	2.18E-51	110.6626	down
CASP3_TAI	-1.12647	-0.04742	-16.2617	1.79E-34	2.40E-33	67.55508	down
GOBP_AM	-1.12544	-0.09193	-26.5832	1.48E-57	1.41E-55	120.7191	down
WP_AMIN(	-1.12507	-0.04522	-20.6294	6.11E-45	1.77E-43	91.66544	down
REACTOM	1.124368	-0.00999	20.79289	2.60E-45	7.74E-44	92.52214	up
MODULE_	-1.12352	-0.1128	-33.9148	9.46E-71	3.77E-68	151.0474	down
GOBP_FRU	-1.12227	-0.1421	-20.8305	2.13E-45	6.40E-44	92.71864	down
REACTOM	-1.12227	-0.1421	-20.8305	2.13E-45	6.40E-44	92.71864	down
GOCC_DEI	1.122116	0.014013	22.75031	1.19E-49	5.34E-48	102.5162	up
HP_ABNOI	-1.12138	-0.06224	-25.295	5.53E-55	4.14E-53	114.7969	down
GOMF_OX	-1.12099	-0.05452	-39.2949	4.20E-79	7.29E-76	170.1964	down
GOBP_NU(	-1.12093	-0.0435	-21.736	1.99E-47	7.09E-46	97.3978	down
GOCC_CM	1.120557	-0.0071	19.98598	1.84E-43	4.68E-42	88.2611	up
KANG_DO)	1.118446	-0.01288	22.67066	1.77E-49	7.72E-48	102.1189	up
GOBP_EPC	-1.11488	-0.07283	-34.5708	8.00E-72	3.54E-69	153.5097	down
WP_COMF	-1.11279	-0.0558	-33.1495	1.77E-69	5.71E-67	148.127	down
MODULE_:	-1.11266	-0.13503	-30.0287	4.77E-64	8.36E-62	135.6502	down
GOMF_BIC	-1.11221	-0.07023	-22.5877	2.68E-49	1.15E-47	101.7041	down
BIOCARTA	-1.11158	-0.04719	-31.894	2.42E-67	5.88E-65	143.2197	down
GNF2_MCI	1.108471	-0.0262	20.7261	3.68E-45	1.08E-43	92.17257	up
MT	-1.1077	-0.05849	-17.3265	4.07E-37	6.50E-36	73.64226	down
BIOCARTA	-1.1074	-0.03539	-29.3466	8.32E-63	1.31E-60	132.795	down
HP_RETIN/	-1.10646	-0.05379	-17.8926	1.69E-38	2.97E-37	76.82633	down
HP_ABNOI	-1.10565	-0.09754	-25.4392	2.82E-55	2.19E-53	115.4698	down
KEGG_PRII	-1.10522	-0.14517	-38.755	2.62E-78	4.02E-75	168.3769	down
REACTOM	-1.10513	0.002892	-29.4201	6.10E-63	9.95E-61	133.1047	down
GOBP_KYN	-1.10485	-0.03934	-28.0375	2.30E-60	2.79E-58	127.178	down
GOBP_ALK	-1.10425	-0.06511	-28.3923	4.93E-61	6.36E-59	128.7186	down
GOMF_AC	-1.1042	-0.10602	-27.8589	5.03E-60	5.78E-58	126.3974	down
GLINSKY_(	1.103607	0.017346	28.30485	7.20E-61	9.22E-59	128.3401	up
WP_BIOM/	-1.10287	-0.10919	-24.0001	2.59E-52	1.51E-50	108.6465	down
GOBP_ENE	-1.10238	-0.0946	-18.014	8.57E-39	1.54E-37	77.50391	down
GOBP_CYS	-1.10087	-0.12049	-20.5682	8.43E-45	2.39E-43	91.34381	down
GOBP_REG	-1.10077	-0.10967	-18.0051	9.00E-39	1.61E-37	77.45449	down
BLANCO_I	1.10032	0.03684	24.14188	1.31E-52	7.77E-51	109.3297	up
WP_UREA_	-1.09838	-0.0937	-21.0063	8.53E-46	2.64E-44	93.63547	down
GOCC_ALI	1.097771	-0.031	19.49254	2.59E-42	6.02E-41	85.61485	up
KEGG_LIM(	-1.09746	-0.09614	-24.5582	1.79E-53	1.17E-51	111.3221	down
WP_COVIE	-1.09682	-0.09135	-23.9133	3.95E-52	2.26E-50	108.2267	down
BOYAULT_	-1.0967	-0.11072	-30.2963	1.57E-64	2.87E-62	136.7573	down

ANDERSEN	-1.0966	-0.14434	-39.2884	4.29E-79	7.29E-76	170.1748	down
GOBP_FAT	-1.09515	-0.12171	-26.3281	4.71E-57	4.29E-55	119.5619	down
GOBP_GLY	-1.09445	-0.06425	-20.7533	3.19E-45	9.42E-44	92.31495	down
GOCC_PR(	-1.09349	-0.0144	-14.9465	3.87E-31	4.27E-30	59.87347	down
REACTOM	-1.09349	-0.0144	-14.9465	3.87E-31	4.27E-30	59.87347	down
MODULE_!	-1.09336	-0.08202	-31.6615	6.13E-67	1.42E-64	142.2949	down
GOBP_TRY	-1.09305	-0.05719	-27.9862	2.88E-60	3.47E-58	126.9542	down
WP_ARACI	-1.09222	-0.10569	-29.1471	1.94E-62	2.91E-60	131.9505	down
WP_AFLAT	-1.09136	-0.16654	-28.7558	1.03E-61	1.41E-59	130.2826	down
GOBP_TYR	-1.09072	-0.09325	-32.0693	1.21E-67	3.17E-65	143.9138	down
GOCC_PR(	-1.09029	-0.05615	-16.541	3.58E-35	5.00E-34	69.16378	down
RARB_TAR	-1.08929	-0.04098	-15.6552	6.04E-33	7.43E-32	64.03351	down
GOMF_OX	-1.08914	-0.06103	-31.4855	1.24E-66	2.82E-64	141.5911	down
GOBP_CEL	1.08826	-0.01122	22.51758	3.81E-49	1.61E-47	101.3532	up
GOMF_GL'	-1.08747	-0.0178	-21.8253	1.26E-47	4.56E-46	97.85342	down
GOBP_KET	-1.0871	-0.08814	-35.2764	5.85E-73	3.04E-70	156.1165	down
GOBP_ASF	-1.08643	-0.01416	-22.7331	1.30E-49	5.76E-48	102.4306	down
GAO_LAR(	1.08617	-0.0081	24.43244	3.25E-53	2.06E-51	110.7224	up
HP_HYPER	-1.08593	-0.09588	-20.4457	1.61E-44	4.48E-43	90.69886	down
HSIAO_LIV	-1.08554	-0.1117	-36.4318	8.81E-75	6.73E-72	160.2954	down
GOBP_CH(	-1.08368	-0.07405	-21.5763	4.50E-47	1.55E-45	96.58022	down
REACTOM	-1.08368	-0.07405	-21.5763	4.50E-47	1.55E-45	96.58022	down
KEGG_FAT	-1.08242	-0.1119	-33.2824	1.06E-69	3.56E-67	148.6381	down
ZHONG_PI	1.081265	-0.00705	24.36478	4.50E-53	2.83E-51	110.399	up
HP_DECRE	-1.08027	-0.08911	-21.4219	9.94E-47	3.32E-45	95.78627	down
GNF2_BUB	1.079588	-0.0206	20.33122	2.94E-44	8.04E-43	90.09434	up
KAPOSI_LI'	-1.07931	-0.11846	-18.8017	1.10E-40	2.27E-39	81.85847	down
SEMBA_FH	1.07785	-0.05974	19.99667	1.73E-43	4.43E-42	88.31809	up
GOBP_HIS	-1.07677	-0.05459	-26.5567	1.67E-57	1.58E-55	120.5995	down
IIZUKA_LIV	-1.07658	0.006494	-36.0941	2.97E-74	1.88E-71	159.0853	down
GOBP_ETH	-1.07247	-0.09307	-23.7572	8.41E-52	4.67E-50	107.4698	down
REACTOM	-1.07247	-0.09307	-23.7572	8.41E-52	4.67E-50	107.4698	down
ISHIDA_E2	1.072259	-0.01933	23.1867	1.37E-50	6.72E-49	104.6786	up
KEGG_VAL	-1.07116	-0.09005	-31.4515	1.42E-66	3.21E-64	141.4551	down
GNF2_CKS	1.069581	-0.02996	20.85238	1.90E-45	5.73E-44	92.83304	up
GOMF_EST	-1.06931	-0.06477	-25.1732	9.78E-55	7.12E-53	114.2272	down
MODULE_:	1.06866	-0.03061	23.50386	2.89E-51	1.52E-49	106.2354	up
MODULE_:	-1.06762	-0.07205	-30.8074	1.93E-65	3.89E-63	138.8521	down
chr6p11	1.066923	0.021487	20.44847	1.58E-44	4.42E-43	90.7135	up
GOBP_DOI	1.066684	-0.04835	21.58154	4.38E-47	1.51E-45	96.60703	up
VILLANUE'	-1.06579	-0.16139	-36.4272	8.95E-75	6.73E-72	160.2791	down
HP_CENTR	-1.06444	-0.06704	-17.6324	7.25E-38	1.23E-36	75.36717	down
GOCC_CO	1.064319	0.026638	20.59998	7.13E-45	2.05E-43	91.51094	up
HP_HYPOT	-1.06404	-0.06314	-22.5756	2.85E-49	1.22E-47	101.6437	down
WHITFIELD	1.063677	-0.01061	21.89431	8.86E-48	3.24E-46	98.20518	up
REACTOM	-1.05933	-0.13538	-22.2445	1.50E-48	6.02E-47	99.98007	down
REACTOM	1.059262	-0.00603	23.61339	1.69E-51	9.18E-50	106.7702	up
GOBP_SER	-1.05921	-0.12383	-25.9696	2.43E-56	2.05E-54	117.9225	down
KIM_LIVER_	-1.05868	-0.12361	-24.1034	1.58E-52	9.28E-51	109.1444	down
KEGG_PR(	-1.05852	-0.08731	-32.6888	1.06E-68	3.08E-66	146.3434	down
REACTOM	-1.05777	-0.01454	-30.5139	6.42E-65	1.21E-62	137.6523	down
HOSHIDA_	-1.05768	-0.12568	-44.0518	9.82E-86	3.96E-82	185.3599	down
REACTOM	1.055666	-0.04888	19.8809	3.22E-43	8.01E-42	87.70015	up
REACTOM	-1.0551	-0.05192	-20.5827	7.81E-45	2.22E-43	91.4199	down
LOPEZ_ME	-1.05445	-0.01067	-27.3467	4.82E-59	5.19E-57	124.1393	down
BIOCARTA	-1.05153	-0.07999	-28.5937	2.07E-61	2.77E-59	129.5869	down
FARMER_B	1.051502	-0.02673	22.4729	4.76E-49	1.99E-47	101.1292	up
GOCC_DN	1.051359	-0.0364	18.62882	2.85E-40	5.67E-39	80.90943	up

GOMF_AN	-1.05104	-0.05685	-22.8596	6.91E-50	3.19E-48	103.0599	down
SU_LIVER	-1.04985	-0.10988	-33.6944	2.19E-70	8.21E-68	150.212	down
REACTOM	-1.04933	-0.10982	-21.7216	2.14E-47	7.59E-46	97.32426	down
LI_WILMS_	1.048665	0.003269	25.22021	7.85E-55	5.81E-53	114.4472	up
REACTOM	-1.04715	-0.09713	-28.7562	1.03E-61	1.41E-59	130.2843	down
GOBP_BLC	-1.04696	-0.109	-33.6635	2.46E-70	9.13E-68	150.0942	down
WP_METH	-1.04652	-0.05705	-32.5739	1.66E-68	4.65E-66	145.8955	down
REICHERT_	1.046351	-0.00154	23.26626	9.27E-51	4.61E-49	105.0703	up
HP_DEEP_	1.043591	-0.02253	22.91384	5.28E-50	2.46E-48	103.3293	up
ROSTY_CE	1.042561	-0.01375	22.09741	3.16E-48	1.22E-46	99.23636	up
WP_MITO	-1.042	-0.1096	-24.8822	3.85E-54	2.62E-52	112.8579	down
GOMF_GL	-1.04189	-0.10497	-24.1081	1.54E-52	9.09E-51	109.167	down
GOBP_L_SI	-1.04115	-0.11533	-19.074	2.49E-41	5.36E-40	83.34639	down
MODULE_	-1.04042	-0.08107	-30.2235	2.13E-64	3.81E-62	136.4567	down
GOCC_PRO	1.039388	0.045319	27.19954	9.28E-59	9.69E-57	123.4853	up
HP_DECRE	-1.03931	-0.12161	-20.062	1.22E-43	3.17E-42	88.66606	down
FUJIWARA	-1.03726	-0.05308	-29.1085	2.28E-62	3.41E-60	131.7867	down
REACTOM	-1.03673	-0.07255	-30.92	1.22E-65	2.49E-63	139.3099	down
GOBP_MEI	-1.03602	-0.15878	-22.4197	6.22E-49	2.56E-47	100.8622	down
HP_HYPOL	-1.03585	-0.04349	-22.7781	1.04E-49	4.71E-48	102.6546	down
GOBP_VIT	-1.03466	-0.15458	-25.6368	1.13E-55	9.14E-54	116.387	down
MODULE_	1.034589	0.010031	26.08919	1.40E-56	1.22E-54	118.4709	up
GOCC_MA	1.03424	0.094953	23.07909	2.33E-50	1.12E-48	104.1476	up
GOBP_ARC	-1.03415	-0.09859	-31.9827	1.70E-67	4.37E-65	143.5713	down
BIOCARTA	-1.03158	0.027284	-26.3908	3.54E-57	3.24E-55	119.8469	down
GOBP_2_O	-1.03067	-0.12669	-26.401	3.38E-57	3.12E-55	119.8931	down
REACTOM	-1.03052	-0.06239	-26.7676	6.43E-58	6.25E-56	121.5511	down
GOCC_FIBI	-1.03028	0.03842	-20.8258	2.19E-45	6.55E-44	92.69397	down
WP_TAMC	-1.02927	-0.08127	-32.6105	1.44E-68	4.10E-66	146.0382	down
GOBP_PHE	-1.02898	-0.11706	-22.5836	2.74E-49	1.17E-47	101.6836	down
WP_GASTI	1.028716	0.046216	31.76434	4.06E-67	9.64E-65	142.7047	up
WP_ALANI	-1.0273	-0.07503	-26.9898	2.37E-58	2.39E-56	122.5486	down
GOBP_ALK	-1.02568	-0.06122	-28.7129	1.24E-61	1.69E-59	130.0986	down
REACTOM	-1.02543	-0.10466	-25.1601	1.04E-54	7.54E-53	114.1656	down
REACTOM	-1.02475	-0.10301	-22.7397	1.26E-49	5.59E-48	102.4634	down
GOBP_REG	-1.02449	-0.10539	-20.8777	1.67E-45	5.06E-44	92.96534	down
GOMF_AN	-1.02294	-0.12168	-22.9482	4.46E-50	2.09E-48	103.4995	down
WP_FARNI	-1.02194	-0.13898	-30.0255	4.84E-64	8.43E-62	135.6366	down
GOCC_CHI	1.021642	-0.03881	19.44491	3.34E-42	7.71E-41	85.35776	up
REACTOM	-1.02105	-0.18881	-20.9733	1.01E-45	3.12E-44	93.46351	down
KEGG_GLY	-1.02023	-0.08913	-28.5458	2.54E-61	3.32E-59	129.3807	down
REACTOM	-1.0197	-0.06684	-29.3626	7.78E-63	1.24E-60	132.8622	down
GOBP_HIS	-1.01927	-0.04557	-21.4472	8.73E-47	2.93E-45	95.91656	down
REACTOM	-1.01927	-0.04557	-21.4472	8.73E-47	2.93E-45	95.91656	down
REACTOM	-1.0185	0.015434	-20.8872	1.59E-45	4.82E-44	93.01494	down
MODULE_	-1.01828	-0.09104	-36.3847	1.04E-74	7.65E-72	160.1273	down
GOBP_GLY	-1.01807	-0.08462	-24.6044	1.43E-53	9.42E-52	111.5418	down
BOYAULT_	1.016319	0.022023	28.76462	9.92E-62	1.37E-59	130.3203	up
OHASHI_A	1.016174	0.016706	22.60045	2.52E-49	1.08E-47	101.7681	up
REACTOM	-1.01545	-0.09229	-29.4728	4.89E-63	8.00E-61	133.3269	down
REACTOM	-1.01491	-0.05731	-27.3585	4.58E-59	4.94E-57	124.1917	down
HP_LEBER_	-1.01465	-0.04859	-15.6995	4.66E-33	5.78E-32	64.29192	down
REACTOM	-1.01452	0.052323	-17.8795	1.82E-38	3.19E-37	76.75276	down
BOYAULT_	-1.01428	-0.09421	-29.3584	7.92E-63	1.25E-60	132.8448	down
GOCC_PLA	-1.01383	-0.05782	-32.591	1.55E-68	4.39E-66	145.9621	down
GOBP_BRA	-1.01324	-0.10275	-27.8016	6.47E-60	7.35E-58	126.1463	down
GNF2_FEN	1.011262	-0.0353	19.91064	2.74E-43	6.88E-42	87.85906	up
HP_DECRE	-1.01021	-0.06586	-22.0616	3.79E-48	1.46E-46	99.05491	down

REACTOM	-1.01001	-0.05076	-36.1458	2.46E-74	1.62E-71	159.2712	down
GOBP_ALD	-1.00862	-0.11523	-29.2104	1.48E-62	2.27E-60	132.2188	down
GOCC_HIG	-1.00837	-0.10035	-28.8488	6.92E-62	9.87E-60	130.6805	down
FAN_EMBF	1.008225	-0.02247	21.25644	2.33E-46	7.53E-45	94.93267	up
GOBP_DOI	1.005485	-0.04865	21.20298	3.08E-46	9.86E-45	94.65609	up
GOBP_TRY	-1.0047	-0.03811	-29.1768	1.71E-62	2.60E-60	132.0764	down
HP_DECRE	-1.00433	0.005969	-18.8959	6.59E-41	1.38E-39	82.37423	down
GOBP_TRI(	-1.00433	-0.11068	-18.8589	8.07E-41	1.67E-39	82.17171	down
KONG_E2F	1.00355	-0.00097	23.4754	3.32E-51	1.74E-49	106.0962	up
GOBP_THF	-1.00303	-0.10272	-20.2891	3.67E-44	9.87E-43	89.87155	down
HP_CEREBI	-1.00278	-0.07414	-21.2793	2.07E-46	6.74E-45	95.05107	down
HP_HYPER	-1.00249	-0.06844	-22.9551	4.30E-50	2.02E-48	103.5341	down
GOBP_HOI	-1.0017	-0.18549	-18.2517	2.29E-39	4.28E-38	78.82619	down
GNF2_RFC	1.001567	-0.02952	19.59893	1.46E-42	3.46E-41	86.188	up
REACTOM	1.000892	0.07355	31.8691	2.68E-67	6.45E-65	143.1211	up
GOBP_SHC	-1.00058	-0.13093	-29.1575	1.86E-62	2.81E-60	131.9948	down
REACTOM	0.998638	0.004998	17.41274	2.50E-37	4.05E-36	74.12979	up
HP_ABNOI	-0.99557	-0.08115	-26.0938	1.37E-56	1.19E-54	118.4918	down
REACTOM	-0.99462	-0.11292	-26.6166	1.27E-57	1.22E-55	120.87	down
WP_ESTRC	-0.99382	-0.04246	-29.3439	8.42E-63	1.32E-60	132.7835	down
GOBP_NEU	-0.99379	0.030502	-17.6117	8.14E-38	1.37E-36	75.25132	down
MODULE_3	-0.9933	-0.09746	-30.6891	3.13E-65	6.16E-63	138.3694	down
REACTOM	-0.99302	-0.0915	-30.6994	3.00E-65	5.94E-63	138.4114	down
GOBP_ASS	0.992126	-0.0015	20.10366	9.82E-44	2.56E-42	88.88769	up
GOBP_NEC	-0.99206	-0.06119	-24.5426	1.92E-53	1.26E-51	111.2478	down
LIANG_SILI	0.991973	0.002831	21.006	8.55E-46	2.64E-44	93.63373	up
MOOTHA_	-0.9916	-0.11964	-26.8014	5.52E-58	5.43E-56	121.7033	down
GOBP_MEI	0.991426	0.059856	19.43529	3.52E-42	8.11E-41	85.30583	up
ODONNEL	0.991195	-0.01709	22.66906	1.79E-49	7.77E-48	102.1109	up
GOMF_MY	-0.99102	-0.10224	-18.5238	5.08E-40	9.95E-39	80.33079	down
SIMBULAN	0.991008	-0.0206	25.15193	1.08E-54	7.78E-53	114.1273	up
MODULE_2	-0.99076	-0.06672	-25.8086	5.09E-56	4.24E-54	117.1815	down
GNF2_CKS	0.990631	-0.05161	18.88876	6.85E-41	1.43E-39	82.33527	up
CHIANG_L	0.990296	0.086499	33.51628	4.32E-70	1.53E-67	149.5333	up
GOCC_MC	0.990033	-0.00627	20.22685	5.11E-44	1.36E-42	89.54172	up
GOMF_HIS	0.989743	0.009538	20.15074	7.64E-44	2.01E-42	89.13788	up
REACTOM	-0.98953	-0.1512	-28.9679	4.16E-62	6.07E-60	131.1888	down
GOBP_TRA	0.98925	0.021237	18.63386	2.77E-40	5.51E-39	80.93714	up
GOBP_DOI	0.988621	-0.03871	19.24463	9.88E-42	2.20E-40	84.27378	up
HP_ABNOI	-0.98858	-0.07747	-17.9342	1.34E-38	2.38E-37	77.05841	down
WP_DISOR	-0.98827	-0.08649	-18.562	4.12E-40	8.10E-39	80.54167	down
GOBP_PRE	0.987384	-0.08383	16.91778	4.14E-36	6.21E-35	71.32063	up
GNF2_MSH	0.987328	-0.01686	20.00262	1.68E-43	4.30E-42	88.34983	up
LOPEZ_ME	0.987266	-0.02659	23.29703	7.96E-51	3.98E-49	105.2216	up
GOCC_SPH	-0.9868	-0.14009	-18.2799	1.96E-39	3.68E-38	78.98249	down
WU_APOP	0.986238	0.016893	25.55872	1.62E-55	1.29E-53	116.0252	up
GOBP_STR	0.985724	0.043438	20.83022	2.14E-45	6.41E-44	92.71729	up
GOBP_REG	-0.98535	-0.00146	-23.3757	5.41E-51	2.76E-49	105.6076	down
CROONQU	0.985206	-0.04104	20.86977	1.74E-45	5.26E-44	92.92384	up
REACTOM	-0.98484	-0.11077	-24.3379	5.11E-53	3.18E-51	110.2702	down
KAMMING	0.984609	-0.00902	20.79817	2.53E-45	7.54E-44	92.54978	up
HP_CONCI	-0.98374	-0.0928	-18.0534	6.88E-39	1.24E-37	77.72383	down
QI_PBMC_	0.983527	-0.02727	19.28065	8.12E-42	1.82E-40	84.46908	up
KEGG_BET	-0.98347	-0.08327	-34.2703	2.47E-71	1.02E-68	152.3864	down
DESCARTE	-0.98234	-0.09024	-26.8905	3.70E-58	3.66E-56	122.1037	down
REACTOM	-0.98205	-0.12501	-23.6588	1.36E-51	7.42E-50	106.9914	down
GOBP_NEC	-0.98155	-0.06843	-20.2812	3.83E-44	1.03E-42	89.82968	down
HP_HYPER	-0.98154	-0.09291	-19.5807	1.61E-42	3.79E-41	86.08971	down

GOMF_AR(	-0.98012	-0.08236	-30.3706	1.16E-64	2.12E-62	137.0636	down
GOBP_COI	-0.97933	-0.00987	-27.868	4.84E-60	5.57E-58	126.4372	down
GOBP_POS	0.979317	0.046848	25.48658	2.26E-55	1.79E-53	115.69	up
HP_LACTIC	-0.97903	-0.05911	-21.7388	1.96E-47	6.99E-46	97.41236	down
KEGG_TRY	-0.97893	-0.08628	-39.7095	1.05E-79	2.25E-76	171.5796	down
PUJANA_B	0.978189	0.012994	24.44078	3.13E-53	1.99E-51	110.7622	up
REACTOM	-0.97817	-0.02628	-19.0996	2.17E-41	4.70E-40	83.48571	down
GOMF_HIS	0.977901	0.033563	11.86451	4.17E-23	2.73E-22	41.39364	up
GOBP_L_PI	-0.97781	-0.13752	-24.9926	2.28E-54	1.61E-52	113.3785	down
REACTOM	-0.97781	-0.13752	-24.9926	2.28E-54	1.61E-52	113.3785	down
GOBP_ASF	-0.97761	-0.10984	-26.9874	2.39E-58	2.41E-56	122.5383	down
GOBP_REG	-0.97686	-0.0947	-15.5698	9.94E-33	1.20E-31	63.53482	down
HP_INCRE/	-0.97617	-0.02748	-27.9367	3.58E-60	4.22E-58	126.7378	down
WP_METH	-0.97544	-0.10278	-21.8949	8.83E-48	3.24E-46	98.20797	down
GOBP_MIT	0.975075	-0.02615	20.64498	5.63E-45	1.63E-43	91.74728	up
WP_PHASI	-0.97435	-0.1332	-24.3465	4.91E-53	3.07E-51	110.3117	down
WP_CEREB	-0.97403	-0.15653	-19.1003	2.16E-41	4.69E-40	83.48932	down
REACTOM	-0.97321	-0.08768	-24.5559	1.81E-53	1.18E-51	111.3112	down
REACTOM	-0.97278	-0.09565	-21.3579	1.38E-46	4.55E-45	95.45646	down
GOBP_REG	-0.97211	-0.07612	-26.288	5.65E-57	5.08E-55	119.379	down
REACTOM	-0.9715	-0.14496	-27.9752	3.03E-60	3.62E-58	126.9061	down
HP_ABNOI	-0.97101	-0.05182	-18.2926	1.82E-39	3.44E-38	79.0526	down
HP_BICARI	-0.97056	-0.09021	-19.9191	2.62E-43	6.59E-42	87.90428	down
GOCC_MIT	0.969802	0.04021	28.97935	3.96E-62	5.81E-60	131.2375	up
HP_ANOTI	0.969674	-0.02133	23.08216	2.30E-50	1.10E-48	104.1628	up
REACTOM	-0.96773	-0.03046	-23.0291	2.98E-50	1.42E-48	103.9003	down
GOCC_CO	0.967603	-0.00263	23.97549	2.92E-52	1.69E-50	108.5275	up
GNF2_RRN	0.967561	-0.0464	19.04751	2.88E-41	6.16E-40	83.20195	up
LEE_LIVER_	-0.96625	-0.14222	-32.3519	3.96E-68	1.07E-65	145.0267	down
SOTIRIOU_	0.965634	-0.04886	20.13343	8.38E-44	2.19E-42	89.04592	up
HP_HEPAT	-0.96363	-0.07802	-23.532	2.52E-51	1.34E-49	106.3729	down
HP_PROLC	-0.96324	-0.0696	-23.9526	3.26E-52	1.88E-50	108.4168	down
REACTOM	-0.96239	-0.01473	-22.1403	2.54E-48	9.91E-47	99.45336	down
GOBP_CEL	-0.96195	-0.08329	-33.1346	1.87E-69	5.99E-67	148.0697	down
GOCC_RES	-0.96183	-0.12306	-16.3417	1.13E-34	1.54E-33	68.01675	down
GOMF_SU(	-0.96183	-0.12306	-16.3417	1.13E-34	1.54E-33	68.01675	down
SMID_BRE/	0.961539	0.008965	22.24208	1.52E-48	6.08E-47	99.96772	up
CROONQL	0.961507	-0.03382	20.65741	5.28E-45	1.53E-43	91.8125	up
MODULE_4	-0.96142	-0.1	-34.5464	8.76E-72	3.77E-69	153.4187	down
GOBP_XEN	-0.96138	-0.12185	-21.2823	2.04E-46	6.64E-45	95.06652	down
HP_INTRAI	-0.96123	-0.0521	-21.0092	8.41E-46	2.60E-44	93.65025	down
REACTOM	-0.96103	-0.19368	-23.4906	3.08E-51	1.62E-49	106.1708	down
GOBP_FEN	0.959936	-0.1213	14.52514	4.69E-30	4.81E-29	57.37955	up
GOBP_TET	-0.95912	-0.11605	-20.5827	7.81E-45	2.22E-43	91.42003	down
GOBP_THF	-0.95867	-0.19637	-17.4745	1.76E-37	2.89E-36	74.47862	down
GOBP_LYS	-0.9586	-0.10698	-23.4485	3.79E-51	1.97E-49	105.9644	down
GOBP_MIT	-0.95854	-0.11857	-17.9881	9.90E-39	1.76E-37	77.35946	down
KALMA_E2	0.958472	-0.08612	16.20685	2.45E-34	3.26E-33	67.23798	up
GOBP_COI	-0.95771	-0.03667	-38.5336	5.58E-78	7.83E-75	167.6243	down
REACTOM	-0.95755	-0.21832	-18.9044	6.29E-41	1.32E-39	82.42098	down
HP_DECRE	-0.95752	-0.02286	-18.4484	7.70E-40	1.50E-38	79.91457	down
GOBP_REG	-0.95648	-0.04149	-32.9164	4.36E-69	1.32E-66	147.2271	down
MODULE_5	0.95625	-0.03517	17.37376	3.11E-37	5.02E-36	73.90956	up
WP_CYSTE	-0.95619	-0.12225	-24.3067	5.94E-53	3.65E-51	110.121	down
GOBP_PRC	-0.95615	-0.07714	-32.0426	1.34E-67	3.50E-65	143.8086	down
HP_ACIDEI	-0.95584	-0.09942	-26.0526	1.66E-56	1.42E-54	118.3032	down
GOBP_BEN	-0.95516	-0.06153	-31.2717	2.93E-66	6.22E-64	140.7325	down
HP_ABNOI	-0.955	-0.03554	-28.5601	2.39E-61	3.17E-59	129.4424	down

BLANCO_N	0.954138	-0.00865	23.34728	6.22E-51	3.16E-49	105.4684	up
GOBP_POS	0.954061	-0.01201	25.46467	2.51E-55	1.97E-53	115.5881	up
HP_PERINE	-0.9537	-0.06957	-17.8827	1.78E-38	3.13E-37	76.77102	down
DESCARTE	-0.95331	-0.07928	-23.1341	1.78E-50	8.60E-49	104.4194	down
REACTOM	-0.95279	-0.12113	-20.9309	1.26E-45	3.86E-44	93.24256	down
HP_JEJUNA	0.952742	0.065739	19.95929	2.12E-43	5.37E-42	88.11875	up
HP_LYMPH	-0.95214	0.048988	-24.8892	3.72E-54	2.54E-52	112.8911	down
GOBP_ICO	-0.95201	-0.11235	-23.3325	6.69E-51	3.37E-49	105.396	down
GOMF_KIN	0.951133	-0.05035	21.00246	8.71E-46	2.68E-44	93.61535	up
MODULE_:	0.949834	-0.03092	19.36433	5.17E-42	1.18E-40	84.92225	up
GOBP_POS	0.949216	-0.00943	19.18686	1.35E-41	2.98E-40	83.96013	up
HP_DICARI	-0.94916	-0.10567	-27.2687	6.82E-59	7.22E-57	123.7932	down
KEGG_CON	-0.94831	-0.06477	-35.1995	7.76E-73	3.97E-70	155.8344	down
ZHONG_PI	0.948053	-0.0095	20.69604	4.31E-45	1.26E-43	92.01511	up
HP_TENDC	-0.94797	-0.08197	-21.041	7.13E-46	2.22E-44	93.81552	down
HP_KETON	-0.94748	-0.06909	-33.0624	2.48E-69	7.62E-67	147.7915	down
GOBP_MO	-0.94737	-0.10856	-35.6907	1.28E-73	7.19E-71	157.6275	down
HP_ANGIC	-0.94712	-0.12801	-23.5892	1.91E-51	1.03E-49	106.6521	down
GOBP_REG	-0.94655	-0.06244	-22.9699	4.00E-50	1.89E-48	103.6074	down
GOBP_REG	-0.9465	-0.02363	-20.0199	1.53E-43	3.93E-42	88.44194	down
HP_ABNOI	-0.94422	-0.05321	-20.6365	5.89E-45	1.70E-43	91.70288	down
GOMF_RN	0.943458	-0.14	16.79072	8.55E-36	1.25E-34	70.59498	up
GOMF_FU(	-0.94324	0.021037	-17.8129	2.63E-38	4.59E-37	76.38025	down
GOBP_FAT	-0.94256	-0.04266	-20.3874	2.18E-44	6.02E-43	90.39107	down
GOBP_DRU	-0.94242	-0.07948	-31.0114	8.41E-66	1.74E-63	139.6808	down
GOBP_REG	-0.94239	0.02715	-23.9192	3.83E-52	2.20E-50	108.2555	down
GOBP_POS	-0.94152	-0.06124	-18.3791	1.13E-39	2.17E-38	79.53189	down
GOCC_INT	-0.94115	-0.1068	-14.5306	4.54E-30	4.67E-29	57.41201	down
YAMASHIT	-0.94106	-0.11447	-39.4994	2.11E-79	4.26E-76	170.8802	down
REACTOM	-0.94023	-0.13284	-26.5695	1.57E-57	1.49E-55	120.6574	down
REACTOM	-0.9402	-0.13067	-28.4742	3.46E-61	4.50E-59	129.0719	down
WP_NAD_I	-0.93993	-0.1149	-21.2187	2.84E-46	9.11E-45	94.73735	down
REACTOM	0.939479	0.065396	21.40186	1.10E-46	3.67E-45	95.68326	up
SANDERS(	-0.93944	-0.15829	-25.219	7.89E-55	5.83E-53	114.4414	down
GOCC_HO	0.939316	-0.13225	17.4183	2.42E-37	3.93E-36	74.1612	up
GOBP_POS	-0.93907	0.002348	-17.7276	4.25E-38	7.26E-37	75.90232	down
GOBP_NE(	-0.93863	-0.05933	-18.2513	2.29E-39	4.29E-38	78.82367	down
GNF2_SM(	0.938359	-0.02843	18.97006	4.40E-41	9.32E-40	82.77952	up
GOMF_PH	-0.93784	-0.13374	-16.5055	4.39E-35	6.10E-34	68.9596	down
GOBP_DNA	0.937457	-0.04102	18.87762	7.28E-41	1.51E-39	82.27431	up
GOBP_NEU	-0.93742	-0.06472	-25.3151	5.03E-55	3.78E-53	114.8908	down
GOBP_KIN	0.936167	-0.02405	24.195	1.01E-52	6.05E-51	109.5851	up
GOCC_MI(	-0.93511	-0.12703	-28.6224	1.83E-61	2.47E-59	129.7102	down
GOBP_REG	-0.9333	-0.06982	-19.66	1.05E-42	2.51E-41	86.51652	down
REACTOM	-0.93282	-0.06056	-35.4991	2.58E-73	1.37E-70	156.9305	down
ZHOU_CEL	0.932234	-0.03158	19.81787	4.51E-43	1.11E-41	87.36299	up
REACTOM	0.932057	-0.01871	22.01349	4.84E-48	1.84E-46	98.81091	up
REACTOM	-0.93183	-0.10888	-20.5614	8.74E-45	2.47E-43	91.30813	down
GOMF_FOI	0.931159	-0.01831	21.02902	7.58E-46	2.36E-44	93.75348	up
GNF2_RFC	0.930142	-0.03698	18.15987	3.81E-39	6.99E-38	78.31607	up
WP_COMF	-0.92933	-0.05347	-34.7295	4.43E-72	2.04E-69	154.0996	down
WP_TRYPT	-0.92895	-0.09505	-36.7157	3.19E-75	2.86E-72	161.3054	down
WANG_ME	0.928475	-0.042	23.33792	6.52E-51	3.28E-49	105.4225	up
GOBP_SPII	0.928015	-0.02559	21.96209	6.28E-48	2.36E-46	98.54989	up
REACTOM	-0.92741	-0.06551	-17.5756	9.98E-38	1.66E-36	75.0481	down
GREENBAU	0.925743	0.004331	22.17561	2.13E-48	8.40E-47	99.63204	up
REACTOM	-0.92567	-0.06522	-33.2687	1.12E-69	3.72E-67	148.5855	down
MODULE_!	0.925652	0.020825	18.82521	9.70E-41	2.00E-39	81.98743	up

GOBP_FAT	-0.9256	-0.11106	-34.4782	1.13E-71	4.80E-69	153.1645	down
WP_BLOOI	-0.92553	-0.07088	-30.6826	3.21E-65	6.28E-63	138.343	down
HP_CAUD/	-0.92493	0.120718	-17.4999	1.53E-37	2.52E-36	74.62148	down
GOMF_ME	-0.92447	-0.17242	-21.1246	4.62E-46	1.46E-44	94.25007	down
GOBP_POS	0.922338	-0.03497	21.81969	1.30E-47	4.69E-46	97.82503	up
GOBP_FAT	-0.92215	-0.11616	-32.137	9.25E-68	2.45E-65	144.1811	down
REACTOM	0.922069	-0.05046	17.56479	1.06E-37	1.76E-36	74.98712	up
MODULE_:	-0.9216	-0.10587	-24.2048	9.68E-53	5.81E-51	109.6322	down
WP_VITAM	-0.92148	-0.12787	-19.1965	1.28E-41	2.84E-40	84.01267	down
GOBP_ALP	-0.92146	-0.08431	-30.3943	1.05E-64	1.94E-62	137.1609	down
REACTOM	-0.92144	-0.14062	-21.6308	3.40E-47	1.19E-45	96.85961	down
REACTOM	-0.92132	-0.02935	-24.4978	2.38E-53	1.54E-51	111.0341	down
GOBP_RES	-0.9213	-0.11135	-18.425	8.76E-40	1.70E-38	79.78539	down
GOBP_ORG	-0.92109	-0.09989	-37.5564	1.64E-76	1.96E-73	164.2595	down
KHETCHOU	-0.92065	-0.08864	-22.7917	9.69E-50	4.40E-48	102.7223	down
GOBP_KET	-0.92018	-0.12226	-23.871	4.84E-52	2.76E-50	108.022	down
WHITEFOF	0.920086	-0.01859	21.58582	4.28E-47	1.48E-45	96.62896	up
REACTOM	-0.9194	-0.0423	-31.3872	1.84E-66	3.99E-64	141.1968	down
REACTOM	0.919386	-0.00642	18.71622	1.76E-40	3.56E-39	81.38977	up
MODULE_2	-0.91881	-0.02902	-36.0436	3.56E-74	2.21E-71	158.9037	down
GOMF_ATI	-0.91851	-0.07494	-19.2142	1.16E-41	2.58E-40	84.10845	down
HP_KETOT	-0.9184	-0.04685	-23.8855	4.51E-52	2.58E-50	108.0923	down
HP_MICRC	0.917813	-0.0429	19.8065	4.79E-43	1.18E-41	87.30214	up
GOCC_FEN	0.917438	0.085751	23.58855	1.91E-51	1.03E-49	106.649	up
chr4q33	-0.91732	0.048938	-15.1006	1.56E-31	1.76E-30	60.78203	down
HP_ABNOI	-0.91693	-0.07853	-16.8571	5.85E-36	8.70E-35	70.97408	down
DESCARTE	-0.91685	-0.07539	-22.0982	3.15E-48	1.21E-46	99.24035	down
WP_COMF	-0.91628	-0.09499	-20.5818	7.85E-45	2.23E-43	91.4153	down
WANG_RE	-0.91525	-0.04639	-24.1806	1.09E-52	6.47E-51	109.5157	down
GOCC_DN	0.915223	-0.06967	14.7579	1.18E-30	1.26E-29	58.75902	up
KEGG_RETI	-0.91519	-0.08209	-31.9459	1.97E-67	4.92E-65	143.4257	down
GOMF_QU	-0.91468	-0.12625	-28.0773	1.94E-60	2.38E-58	127.3515	down
GOMF_OX	-0.91424	-0.08738	-30.7062	2.92E-65	5.81E-63	138.4393	down
REACTOM	-0.91369	-0.13316	-18.7401	1.55E-40	3.14E-39	81.52072	down
REN_BOUI	0.912894	-0.03115	19.63628	1.19E-42	2.84E-41	86.38883	up
GOBP_RES	-0.91281	-0.046	-19.2873	7.83E-42	1.76E-40	84.5054	down
GOBP_CEL	-0.91281	-0.046	-19.2873	7.83E-42	1.76E-40	84.5054	down
GOBP_NEG	-0.91264	-0.03825	-16.9662	3.14E-36	4.76E-35	71.59688	down
GOBP_DET	-0.91138	0.027657	-19.5228	2.20E-42	5.13E-41	85.77817	down
REACTOM	-0.91112	-0.10605	-19.5009	2.47E-42	5.77E-41	85.65976	down
MODULE_4	-0.91039	-0.03051	-24.7641	6.72E-54	4.53E-52	112.2998	down
HP_OROTI	-0.91016	-0.03261	-17.5893	9.24E-38	1.55E-36	75.12503	down
MODULE_4	0.909854	0.009952	26.49295	2.22E-57	2.09E-55	120.3106	up
ACEVEDO_	-0.90974	-0.05853	-44.9031	7.42E-87	3.99E-83	187.9208	down
GOBP_SUC	-0.90869	-0.08304	-17.2709	5.57E-37	8.85E-36	73.32738	down
GOCC_MIT	-0.90827	0.010678	-11.7507	8.32E-23	5.33E-22	40.70561	down
MODULE_:	0.908068	-0.03443	18.71935	1.73E-40	3.51E-39	81.40695	up
GOBP_TRIG	-0.90778	-0.08478	-19.5825	1.59E-42	3.76E-41	86.09956	down
REACTOM	-0.90771	-0.04228	-27.9233	3.80E-60	4.44E-58	126.6795	down
VILLANUE\	0.907324	0.074714	35.11939	1.04E-72	5.18E-70	155.5401	up
REACTOM	-0.90715	0.046311	-16.5331	3.74E-35	5.23E-34	69.1186	down
GOMF_C_/	-0.90689	-0.05982	-31.8964	2.40E-67	5.87E-65	143.2293	down
HP_HEMO	-0.90617	-0.05402	-24.2682	7.14E-53	4.34E-51	109.9366	down
GOBP_NEG	-0.90562	0.067486	-20.6279	6.16E-45	1.78E-43	91.6575	down
GOBP_CHI	0.905268	-0.02713	21.10075	5.23E-46	1.65E-44	94.12611	up
MODULE_!	0.905092	-0.00096	23.3196	7.13E-51	3.58E-49	105.3325	up
BOYAULT_	0.904858	-0.01677	23.76614	8.05E-52	4.49E-50	107.5135	up
GOBP_PHY	-0.90444	-0.18437	-18.6751	2.21E-40	4.41E-39	81.16364	down

REACTOM	-0.90443	-0.06914	-29.4828	4.69E-63	7.72E-61	133.3686	down
ZHOU_CEL	0.904307	-0.01791	20.38387	2.23E-44	6.13E-43	90.37257	up
GOBP_FIBI	-0.90431	-0.0421	-32.6761	1.11E-68	3.20E-66	146.2941	down
OHGUCHI_	-0.90419	-0.08016	-31.4148	1.65E-66	3.66E-64	141.3075	down
WP_KETOC	-0.9041	0.013667	-22.4254	6.05E-49	2.50E-47	100.8906	down
GOBP_POS	-0.90378	-0.08847	-17.8444	2.21E-38	3.86E-37	76.55635	down
REACTOM	0.90254	-0.0422	19.13841	1.76E-41	3.85E-40	83.69679	up
BIOCARTA	0.902409	-0.03864	19.15168	1.64E-41	3.59E-40	83.76897	up
GOBP_AM	-0.90237	-0.09976	-25.3883	3.58E-55	2.75E-53	115.2324	down
ACEVEDO_	-0.9013	-0.01754	-49.6985	7.57E-93	8.14E-89	201.575	down
GOMF_MU	0.900882	0.018902	14.97373	3.29E-31	3.65E-30	60.03424	up
GOBP_SUL	-0.90054	-0.1975	-16.1634	3.15E-34	4.16E-33	66.98674	down
GOBP_REG	0.899766	-0.03547	21.138	4.31E-46	1.37E-44	94.31939	up
ZHONG_PI	0.899427	-0.02628	21.02289	7.83E-46	2.43E-44	93.72161	up
GOMF_D_I	0.898709	-0.02113	17.7822	3.13E-38	5.41E-37	76.20818	up
MODULE_4	-0.89843	-0.06868	-24.3114	5.81E-53	3.58E-51	110.1434	down
GOCC_PRC	-0.89717	-0.0733	-27.9019	4.17E-60	4.84E-58	126.5859	down
GOBP_HIS	0.897101	0.070131	19.28471	7.95E-42	1.79E-40	84.49107	up
KEGG_BUT	-0.89707	-0.08068	-27.1384	1.22E-58	1.26E-56	123.2127	down
GOBP_RIB	-0.89646	-0.06656	-19.1244	1.90E-41	4.13E-40	83.62081	down
REACTOM	0.895763	-0.03537	18.77305	1.29E-40	2.64E-39	81.70157	up
REACTOM	-0.89552	0.042686	-18.1397	4.26E-39	7.80E-38	78.20382	down
BIOCARTA	0.895433	0.044501	19.48542	2.69E-42	6.24E-41	85.57641	up
HP_MIXED	-0.89521	-0.06304	-15.022	2.48E-31	2.77E-30	60.31899	down
GOBP_DE_	0.894857	-0.06927	16.7087	1.37E-35	1.97E-34	70.12564	up
GOMF_TRA	-0.89424	-0.08014	-27.1327	1.25E-58	1.28E-56	123.1876	down
WENG_PO	-0.89414	-0.05234	-31.1933	4.03E-66	8.44E-64	140.4162	down
HP_MULTI	-0.89402	-0.01899	-14.8428	7.14E-31	7.75E-30	59.26136	down
REACTOM	-0.89327	-0.14407	-25.0205	2.00E-54	1.41E-52	113.5098	down
GOMF_NU	-0.89312	-0.02827	-18.3834	1.10E-39	2.12E-38	79.5556	down
GOBP_DE_	-0.89297	-0.07905	-19.9178	2.64E-43	6.63E-42	87.89726	down
REACTOM	-0.89241	-0.11205	-25.7433	6.89E-56	5.64E-54	116.8797	down
GOBP_SUL	-0.89171	-0.07893	-22.2812	1.25E-48	5.03E-47	100.1649	down
HP_MENO	-0.89159	-0.03491	-19.7808	5.49E-43	1.34E-41	87.16445	down
FAN_EMBI	0.891323	-0.03181	21.71756	2.18E-47	7.74E-46	97.30357	up
REACTOM	-0.89098	-0.13593	-16.6271	2.18E-35	3.10E-34	69.65821	down
GOBP_DN/	0.889915	-0.02916	20.59665	7.26E-45	2.08E-43	91.49348	up
LEE_LIVER_	-0.8898	-0.07777	-33.6122	2.99E-70	1.10E-67	149.8992	down
GOBP_REG	-0.88941	-0.04855	-18.1906	3.21E-39	5.94E-38	78.4869	down
GOBP_PRC	0.88921	-0.05085	20.42509	1.79E-44	4.96E-43	90.59016	up
HP_ABNOI	-0.8892	-0.09211	-25.3316	4.66E-55	3.52E-53	114.9678	down
WP_REGUI	0.888925	-0.03994	18.96101	4.62E-41	9.77E-40	82.73011	up
BIOCARTA	-0.88805	-0.09188	-30.0484	4.40E-64	7.75E-62	135.7318	down
REACTOM	-0.88778	-0.10168	-18.9426	5.11E-41	1.08E-39	82.62961	down
GOMF_DN	0.887487	-0.02256	19.71032	8.01E-43	1.93E-41	86.78656	up
GOBP_SPH	-0.88697	0.029296	-20.4058	1.98E-44	5.48E-43	90.48847	down
VARELA_ZI	-0.88607	-0.07382	-30.4891	7.11E-65	1.33E-62	137.5506	down
GOMF_S_N	-0.88599	-0.09723	-22.7615	1.13E-49	5.07E-48	102.5721	down
HP_ABNOI	-0.88581	-0.10554	-21.2644	2.24E-46	7.25E-45	94.97369	down
GOBP_CEN	0.88557	-0.01323	24.45831	2.88E-53	1.84E-51	110.8459	up
GOBP_MIT	0.885568	-0.00241	21.92535	7.57E-48	2.81E-46	98.36311	up
BIOCARTA	0.885158	-0.02397	21.38532	1.20E-46	3.98E-45	95.59805	up
HP_THRON	-0.88491	-0.06329	-20.17	6.90E-44	1.82E-42	89.24036	down
HP_ABNOI	-0.88466	-0.08427	-22.3966	6.99E-49	2.87E-47	100.7458	down
GOBP_ACY	-0.88435	-0.01038	-16.0586	5.78E-34	7.54E-33	66.37957	down
HP_HYPER	-0.884	-0.08967	-17.0112	2.43E-36	3.71E-35	71.85291	down
GOBP_XEN	-0.88376	-0.0979	-21.8496	1.11E-47	4.05E-46	97.9773	down
SCIAN_CEL	0.883671	-0.04766	17.02125	2.30E-36	3.51E-35	71.91018	up

GOBP_REG	-0.88335	-0.09204	-22.7692	1.08E-49	4.89E-48	102.6102	down
GOBP_URE	-0.88319	-0.11102	-19.3763	4.84E-42	1.11E-40	84.98675	down
GOBP_REV	-0.88302	-0.06249	-19.249	9.64E-42	2.15E-40	84.29746	down
GOBP_AM	-0.88292	-0.05257	-31.8657	2.71E-67	6.48E-65	143.1076	down
REACTOM	-0.8818	-0.05908	-26.2731	6.05E-57	5.42E-55	119.3109	down
GOBP_DRU	-0.88158	-0.08174	-34.5549	8.49E-72	3.70E-69	153.4504	down
HP_EPISOI	-0.88155	-0.03415	-23.3979	4.85E-51	2.49E-49	105.7167	down
GOMF_OX	-0.88154	-0.04475	-16.7519	1.07E-35	1.55E-34	70.37298	down
REACTOM	-0.88153	-0.03083	-18.6266	2.88E-40	5.73E-39	80.89733	down
GOBP_ARC	-0.88135	-0.18351	-15.9829	8.97E-34	1.16E-32	65.94053	down
IIZUKA_LIV	-0.88101	-0.11321	-21.0772	5.90E-46	1.86E-44	94.00402	down
HP_INCRE	-0.88063	-0.14308	-16.5031	4.45E-35	6.18E-34	68.94585	down
YU_MYC_T	0.880622	-0.05389	18.81133	1.05E-40	2.15E-39	81.91142	up
GOBP_MEI	0.880599	0.010528	22.00586	5.03E-48	1.91E-46	98.7722	up
BIOCARTA	0.880456	-0.03388	17.63835	7.01E-38	1.19E-36	75.4009	up
HP_MITOC	-0.88031	-0.11662	-16.3067	1.38E-34	1.87E-33	67.81456	down
REACTOM	-0.87994	-0.03247	-25.3581	4.12E-55	3.12E-53	115.0915	down
GOBP_TRY	-0.87987	-0.01866	-17.8872	1.74E-38	3.06E-37	76.79599	down
GOCC_GR	-0.87983	0.130843	-16.7141	1.33E-35	1.91E-34	70.15675	down
MODULE_	-0.87977	-0.05237	-25.7847	5.69E-56	4.70E-54	117.0709	down
GOBP_CAI	-0.87977	0.026429	-15.4405	2.12E-32	2.52E-31	62.77788	down
GOBP_NEC	-0.87932	-0.1141	-15.7658	3.17E-33	3.96E-32	64.6786	down
GOBP_BIO	-0.8791	-0.14759	-20.0185	1.54E-43	3.96E-42	88.43431	down
GOBP_LEU	-0.87894	-0.078	-23.5125	2.77E-51	1.47E-49	106.2778	down
FRASOR_R	0.877975	-0.04342	20.70214	4.17E-45	1.22E-43	92.04706	up
HP_CEREBI	-0.87793	-0.06483	-23.1067	2.03E-50	9.82E-49	104.2841	down
MODULE_	0.87744	0.017476	19.88175	3.20E-43	7.98E-42	87.70466	up
HP_ABNOI	-0.87723	0.020911	-15.62	7.41E-33	9.06E-32	63.82818	down
BURTON_	0.877111	0.003392	21.72319	2.12E-47	7.54E-46	97.33235	up
HP_ABNOI	-0.87711	-0.08104	-28.5669	2.32E-61	3.09E-59	129.4713	down
GOMF_HIS	0.876765	0.001696	19.74096	6.80E-43	1.65E-41	86.95091	up
HP_ABNOI	-0.87604	-0.10715	-28.8188	7.86E-62	1.11E-59	130.5523	down
WP_UREA_	-0.87589	-0.08321	-21.5342	5.58E-47	1.91E-45	96.36419	down
WP_ESTRC	-0.87517	-0.07441	-21.8974	8.72E-48	3.21E-46	98.22086	down
REACTOM	0.874977	-0.03137	18.16522	3.69E-39	6.80E-38	78.3458	up
REACTOM	-0.87457	-0.09699	-23.5086	2.82E-51	1.49E-49	106.2584	down
GOBP_FAT	-0.87412	-0.14809	-20.5983	7.20E-45	2.06E-43	91.50207	down
MATHEW_	0.873938	0.022923	18.21727	2.77E-39	5.15E-38	78.63492	up
MOLENAA	0.873795	0.005163	22.83897	7.66E-50	3.52E-48	102.9574	up
GOBP_OPS	-0.87364	-0.01034	-22.4562	5.18E-49	2.15E-47	101.0453	down
GOMF_ALI	-0.87352	-0.07609	-27.8357	5.57E-60	6.38E-58	126.2958	down
GOMF_ALI	-0.87351	-0.1038	-20.9128	1.39E-45	4.23E-44	93.14853	down
WP_MITO	-0.87325	-0.19706	-15.2501	6.47E-32	7.47E-31	61.66116	down
HP_RENAL	-0.87278	-0.01521	-15.7173	4.20E-33	5.23E-32	64.39548	down
HP_SMALL	0.872281	0.061632	17.60383	8.51E-38	1.43E-36	75.20679	up
GOBP_NEC	-0.87131	-0.00789	-21.6416	3.22E-47	1.13E-45	96.91503	down
GOBP_POS	-0.87109	-0.06147	-14.9057	4.92E-31	5.41E-30	59.63261	down
SONG_TAI	0.869935	-0.01834	19.05132	2.82E-41	6.04E-40	83.22272	up
HP_SUBDU	-0.8698	-0.07014	-19.0714	2.53E-41	5.44E-40	83.33233	down
GOBP_REG	-0.86969	-0.02107	-19.711	7.99E-43	1.93E-41	86.78998	down
GOMF_AN	0.869039	-0.018	17.24232	6.55E-37	1.03E-35	73.16575	up
WP_CATAI	-0.86813	-0.04461	-16.2906	1.51E-34	2.04E-33	67.72176	down
GOMF_N_I	-0.86813	-0.04461	-16.2906	1.51E-34	2.04E-33	67.72176	down
GOMF_SIN	0.86812	-0.01692	20.28381	3.78E-44	1.01E-42	89.84346	up
GOBP_G_C	0.868023	0.080869	18.84757	8.59E-41	1.78E-39	82.1099	up
HP_PROLC	-0.86771	-0.05864	-21.9189	7.82E-48	2.90E-46	98.33007	down
GOBP_ARC	-0.86712	-0.06726	-34.672	5.48E-72	2.49E-69	153.8863	down
BIOCARTA	-0.86668	-0.06753	-21.0568	6.56E-46	2.06E-44	93.898	down

HP_INCREA	-0.86655	-0.13177	-15.9753	9.37E-34	1.21E-32	65.89682	down
GRAHAM_	0.866447	-0.04106	18.71184	1.81E-40	3.64E-39	81.36571	up
REACTOM	0.866418	-0.05623	20.06023	1.24E-43	3.20E-42	88.65664	up
CHASSOT_	-0.86637	0.179062	-15.5949	8.59E-33	1.05E-31	63.68126	down
GOBP_REP	0.86606	-0.00144	22.71061	1.45E-49	6.39E-48	102.3183	up
MODULE_3	0.865743	-0.01554	18.34168	1.39E-39	2.65E-38	79.32465	up
REACTOM	0.864886	-0.04176	17.25135	6.22E-37	9.82E-36	73.21686	up
REACTOM	0.864875	0.027375	21.21979	2.82E-46	9.06E-45	94.74308	up
GOBP_KET	-0.86469	-0.04915	-26.4456	2.76E-57	2.56E-55	120.0959	down
VAN_DEN_	-0.86443	-0.04497	-13.0348	3.52E-26	2.85E-25	48.4601	down
HP_APLAS	0.863612	0.043604	21.91488	7.98E-48	2.96E-46	98.30985	up
KEGG_DRU	-0.86335	-0.09352	-30.6208	4.14E-65	8.00E-63	138.0902	down
REACTOM	0.862219	-0.04158	17.28273	5.21E-37	8.28E-36	73.3946	up
GOBP_CHI	0.862155	0.049603	18.01964	8.30E-39	1.49E-37	77.53543	up
HP_RETINA	-0.8618	-0.05028	-19.584	1.58E-42	3.73E-41	86.10738	down
GOBP_KIN	0.861749	-0.03992	22.90961	5.39E-50	2.51E-48	103.3083	up
MODULE_4	0.861745	-0.00546	20.87127	1.72E-45	5.22E-44	92.93166	up
GOMF_VIT	-0.86161	-0.07625	-36.3622	1.13E-74	8.11E-72	160.0466	down
SAENZ_DE	-0.86148	-0.12163	-24.9311	3.05E-54	2.12E-52	113.0887	down
GOBP_PLA	-0.86118	-0.04804	-32.3791	3.56E-68	9.82E-66	145.1334	down
HP_HYPOL	-0.8607	-0.11912	-20.7748	2.85E-45	8.46E-44	92.42741	down
ZHONG_PI	0.860595	-0.01989	20.66832	4.98E-45	1.45E-43	91.86972	up
DESERT_ST	0.860528	0.084557	33.88645	1.05E-70	4.10E-68	150.9403	up
GOBP_POS	0.860508	0.055257	17.1792	9.36E-37	1.46E-35	72.8078	up
PID_ATR_P	0.86003	-0.01066	22.53194	3.54E-49	1.50E-47	101.4252	up
LEE_LIVER_	-0.85978	-0.08986	-33.2024	1.44E-69	4.70E-67	148.3308	down
GOBP_ETH	-0.85976	-0.05256	-24.2427	8.07E-53	4.88E-51	109.8142	down
WP_ULTRA	0.859517	0.036029	13.53189	1.77E-27	1.56E-26	51.4493	up
HP_HYPOI	0.859477	0.027372	22.22345	1.67E-48	6.67E-47	99.87372	up
GOMF_STE	-0.85926	-0.08299	-30.8866	1.40E-65	2.84E-63	139.1742	down
GOBP_SPIN	0.858895	0.018729	18.59988	3.34E-40	6.62E-39	80.75013	up
OHASHI_A	0.858499	-0.05023	17.43165	2.25E-37	3.65E-36	74.23659	up
DESCARTE	-0.85718	-0.06607	-22.7047	1.49E-49	6.56E-48	102.289	down
GOBP_DNA	0.857112	-0.03671	21.86338	1.04E-47	3.78E-46	98.0477	up
GOBP_MRI	0.856138	-0.08475	14.89667	5.19E-31	5.69E-30	59.57938	up
HP_ABNOI	-0.85564	-0.0493	-19.2907	7.69E-42	1.74E-40	84.52382	down
MORI_IMM	0.855421	-0.03529	19.59576	1.48E-42	3.51E-41	86.17093	up
FLORIO_NI	0.855366	0.01161	21.51797	6.07E-47	2.07E-45	96.28066	up
GOCC_TRI	-0.85479	-0.11542	-18.5157	5.31E-40	1.04E-38	80.28656	down
HP_DISTAL	-0.85479	-0.06428	-17.8044	2.76E-38	4.80E-37	76.33281	down
GOBP_SER	-0.85465	-0.10588	-23.3465	6.25E-51	3.16E-49	105.4646	down
MODULE_4	-0.85428	-0.11384	-28.8665	6.41E-62	9.24E-60	130.7562	down
GOBP_LON	-0.85399	-0.01075	-25.0588	1.67E-54	1.19E-52	113.6901	down
GOBP_REG	0.85379	-0.07564	16.35854	1.02E-34	1.40E-33	68.11363	up
TANG_SEN	0.853769	-0.0007	20.78949	2.64E-45	7.87E-44	92.50437	up
GOBP_DNA	0.853497	-0.0555	19.175	1.44E-41	3.18E-40	83.89573	up
CHIANG_L	-0.85236	-0.0328	-30.4397	8.71E-65	1.62E-62	137.3475	down
REACTOM	-0.852	-0.03992	-22.9684	4.03E-50	1.90E-48	103.5997	down
LEE_LIVER_	-0.85194	-0.09375	-33.5427	3.90E-70	1.40E-67	149.6342	down
HP_HYPOC	-0.8517	-0.08939	-24.0504	2.04E-52	1.19E-50	108.8892	down
GOBP_RES	0.851303	-0.06346	17.16825	9.96E-37	1.55E-35	72.74564	up
HP_HYPOC	-0.85095	0.001715	-20.6691	4.96E-45	1.44E-43	91.87381	down
GOBP_RES	-0.85064	0.078744	-19.0601	2.69E-41	5.76E-40	83.27075	down
BURTON_A	0.850453	-0.06931	21.63377	3.35E-47	1.17E-45	96.87473	up
HP_ABNOI	-0.85033	-0.08881	-23.4696	3.42E-51	1.78E-49	106.068	down
HALLMARI	-0.85032	-0.09234	-36.1724	2.24E-74	1.50E-71	159.3668	down
GOMF_ARY	-0.85025	-0.02052	-16.743	1.12E-35	1.63E-34	70.32228	down
GOMF_ATI	0.850173	-0.02716	17.92015	1.45E-38	2.56E-37	76.9801	up

GOMF_GLY	-0.84977	0.001133	-15.0758	1.80E-31	2.03E-30	60.63604	down
GOMF_INS	-0.84955	0.032498	-20.3171	3.17E-44	8.61E-43	90.01961	down
HP_ORGAI	-0.84938	-0.11642	-25.8162	4.92E-56	4.11E-54	117.2164	down
LEE_EARLY	0.848527	-0.01293	21.69921	2.40E-47	8.45E-46	97.2097	up
YAO_TEMI	-0.84843	0.04791	-24.3453	4.94E-53	3.09E-51	110.3057	down
GOBP_NEC	-0.84829	-0.06005	-18.5028	5.70E-40	1.11E-38	80.21515	down
HP_ABNOI	0.84778	0.010043	20.0691	1.18E-43	3.05E-42	88.70385	up
GOBP_POS	-0.84637	-0.086	-18.3857	1.09E-39	2.10E-38	79.56802	down
GOMF_LIP	-0.84557	0.104211	-18.2526	2.28E-39	4.26E-38	78.83085	down
HP_ASTERI	-0.84499	-0.08775	-15.2647	5.94E-32	6.87E-31	61.74674	down
GOBP_LIPI	-0.84457	-0.0936	-32.8494	5.66E-69	1.69E-66	146.9673	down
WP_CODE	-0.84437	-0.08157	-18.7259	1.67E-40	3.39E-39	81.44284	down
GOBP_STR	-0.84332	0.022821	-18.236	2.49E-39	4.66E-38	78.739	down
HP_HALITC	-0.84257	-0.03201	-19.4909	2.61E-42	6.07E-41	85.60584	down
REACTOM	-0.84208	-0.0801	-22.7724	1.07E-49	4.82E-48	102.6263	down
GOBP_PRC	-0.84201	-0.14078	-15.1961	8.89E-32	1.02E-30	61.34377	down
REACTOM	-0.84201	-0.14078	-15.1961	8.89E-32	1.02E-30	61.34377	down
REACTOM	-0.84193	-0.1372	-25.3583	4.11E-55	3.12E-53	115.0926	down
HP_ABNOI	-0.84176	-0.10642	-15.2089	8.25E-32	9.46E-31	61.41892	down
GOBP_PRC	0.841561	-0.03096	21.14716	4.11E-46	1.31E-44	94.36686	up
MODULE_!	-0.84121	-0.15722	-18.1019	5.25E-39	9.58E-38	77.9934	down
GOBP_MIT	0.840358	-0.0547	16.65597	1.85E-35	2.65E-34	69.82353	up
GOBP_POS	-0.84011	-0.18498	-18.7738	1.29E-40	2.63E-39	81.70596	down
GOBP_HIG	-0.83955	-0.05546	-19.3074	7.03E-42	1.59E-40	84.61398	down
GOBP_DN/	0.839273	-0.05141	18.32432	1.53E-39	2.91E-38	79.22855	up
GOMF_URI	-0.83889	0.016143	-20.0453	1.34E-43	3.45E-42	88.57724	down
GOBP_RES	-0.83853	-0.11123	-27.9545	3.31E-60	3.91E-58	126.8158	down
HP_ABNOI	0.838384	0.029633	24.35156	4.79E-53	3.01E-51	110.3358	up
GOBP_CYS	-0.83798	-0.12582	-19.9445	2.29E-43	5.78E-42	88.04007	down
GOMF_OX	-0.83781	-0.14033	-19.8367	4.07E-43	1.01E-41	87.46355	down
REACTOM	-0.83749	-0.07999	-25.2522	6.75E-55	5.01E-53	114.5969	down
REICHERT_	0.837441	0.021607	17.76609	3.42E-38	5.90E-37	76.11794	up
VAN_DEN_	0.837433	0.008172	21.1896	3.30E-46	1.06E-44	94.58681	up
REACTOM	-0.83721	-0.06223	-24.8947	3.62E-54	2.49E-52	112.9171	down
GOBP_IMN	-0.83651	0.123928	-14.9735	3.30E-31	3.65E-30	60.03276	down
MODULE_:	-0.8365	-0.04527	-22.7282	1.33E-49	5.88E-48	102.4059	down
GSE15750_	0.836318	-0.02543	20.86195	1.81E-45	5.47E-44	92.88301	up
GOBP_ADI	-0.83632	-0.07116	-18.1222	4.69E-39	8.57E-38	78.10637	down
GOBP_MU	0.835982	-0.05437	17.77447	3.27E-38	5.64E-37	76.16489	up
HP_ABSEN	0.835927	-0.06021	15.55772	1.07E-32	1.29E-31	63.46402	up
HP_MATEF	-0.8355	-0.03435	-16.2929	1.49E-34	2.02E-33	67.73478	down
HP_FASTIN	-0.8347	-0.08861	-21.2884	1.98E-46	6.45E-45	95.09772	down
HP_PROGI	-0.83451	-0.0101	-17.4754	1.75E-37	2.88E-36	74.48314	down
HOEK_MO	-0.83438	0.044588	-16.7717	9.54E-36	1.39E-34	70.48607	down
GOCC_CILI	0.83428	0.00608	17.56989	1.03E-37	1.72E-36	75.0158	up
GOCC_CO	0.834151	-0.08864	13.88197	2.17E-28	2.02E-27	53.54673	up
GOCC_CTI	0.834124	-0.04746	15.68116	5.19E-33	6.41E-32	64.18492	up
HP_COROI	-0.834	-0.03879	-17.1858	9.02E-37	1.41E-35	72.84548	down
GNF2_MCI	0.833897	-0.02404	17.92288	1.42E-38	2.52E-37	76.99539	up
HP_HYPER	-0.83387	-0.11577	-22.7611	1.13E-49	5.07E-48	102.5701	down
HP_HYPOT	-0.83384	-0.00099	-27.9296	3.69E-60	4.33E-58	126.707	down
WP_EFFEC	-0.83371	-0.06863	-21.334	1.56E-46	5.11E-45	95.33335	down
GOMF_OX	-0.83299	-0.02312	-20.3142	3.22E-44	8.73E-43	90.00428	down
GOBP_NEC	-0.83279	-0.0455	-17.1822	9.20E-37	1.44E-35	72.82502	down
GOBP_NEC	-0.83181	-0.09464	-20.5339	1.01E-44	2.84E-43	91.16376	down
SALVADOI	-0.83144	-0.02517	-22.9342	4.77E-50	2.24E-48	103.4302	down
KAUFFMAI	0.830936	-0.03984	18.23069	2.57E-39	4.79E-38	78.70943	up
GOBP_POS	-0.83089	-0.11708	-17.9887	9.87E-39	1.76E-37	77.36302	down

GOBP_SPIN	0.83087	-0.02477	19.01243	3.49E-41	7.43E-40	83.0107	up
GOMF_DN	0.830613	-0.05264	22.06111	3.80E-48	1.46E-46	99.05245	up
REACTOM	-0.83027	-0.05742	-33.0902	2.23E-69	6.97E-67	147.8987	down
WP_PPAR_	-0.83015	-0.06586	-24.1432	1.30E-52	7.73E-51	109.3362	down
GOBP_MEI	0.82922	0.01319	22.74495	1.22E-49	5.47E-48	102.4895	up
LEE_LIVER_	-0.82914	-0.08479	-32.3863	3.46E-68	9.63E-66	145.1615	down
GOBP_CAF	-0.82894	-0.08533	-21.8707	9.99E-48	3.65E-46	98.08475	down
GOBP_FUN	0.8288	0.011581	16.03284	6.71E-34	8.73E-33	66.23048	up
YAGUE_PR	-0.82853	0.031808	-18.2005	3.04E-39	5.64E-38	78.54159	down
GOBP_POS	-0.82787	-0.08493	-23.7965	6.95E-52	3.91E-50	107.6609	down
WP_SULIN	-0.82744	0.015155	-18.3163	1.60E-39	3.03E-38	79.18439	down
HP_PREMA	-0.82718	0.009273	-15.7951	2.67E-33	3.35E-32	64.84873	down
GOCC_MIT	0.827078	0.022479	22.26697	1.34E-48	5.39E-47	100.0933	up
GOMF_TES	-0.82619	-0.15818	-18.4185	9.08E-40	1.76E-38	79.7496	down
GOBP_POS	-0.82607	-0.04767	-14.2553	2.33E-29	2.30E-28	55.77492	down
REACTOM	-0.82595	-0.11838	-17.584	9.52E-38	1.59E-36	75.09515	down
HALLMARI	-0.82574	-0.104	-29.9992	5.39E-64	9.35E-62	135.5277	down
REACTOM	0.825499	-0.07992	15.35893	3.42E-32	4.00E-31	62.30003	up
GOBP_MRI	0.825497	-0.02708	15.85927	1.84E-33	2.33E-32	65.22245	up
GOBP_POS	0.823687	0.003832	17.2656	5.74E-37	9.09E-36	73.29764	up
HP_XANTH	-0.82326	-0.09261	-20.6145	6.61E-45	1.90E-43	91.58731	down
GOBP_REG	-0.823	-0.07133	-26.8212	5.05E-58	4.98E-56	121.7923	down
CAIRO_LIV	-0.82239	-0.06149	-33.7212	1.97E-70	7.50E-68	150.3139	down
MODULE_4	-0.82231	-0.04203	-27.7535	8.00E-60	9.02E-58	125.9352	down
FOURNIER	0.822147	0.02036	21.15326	3.98E-46	1.27E-44	94.39849	up
HP_ABSEN	0.82152	0.014208	22.92334	5.04E-50	2.36E-48	103.3764	up
REACTOM	0.821172	0.006747	23.27587	8.84E-51	4.41E-49	105.1176	up
HP_SMALL	0.821115	-0.01155	19.56292	1.77E-42	4.16E-41	85.99412	up
GOBP_MEI	-0.82106	-0.06778	-21.3916	1.16E-46	3.86E-45	95.63044	down
MODULE_5	0.820517	-0.02052	21.73977	1.95E-47	6.97E-46	97.41705	up
HP_ABNOI	-0.82028	-0.0677	-24.4756	2.65E-53	1.71E-51	110.9285	down
MODULE_3	-0.82027	0.058106	-21.5159	6.13E-47	2.09E-45	96.26981	down
GOBP_HYD	-0.82022	-0.00048	-22.2993	1.14E-48	4.62E-47	100.2562	down
GOBP_POS	-0.81997	-0.05914	-22.5334	3.52E-49	1.50E-47	101.4326	down
WP_DRUG_	-0.81984	-0.13746	-25.2179	7.93E-55	5.84E-53	114.4364	down
HP_METHY	-0.81946	-0.07233	-21.4	1.11E-46	3.70E-45	95.67378	down
ZHONG_PI	0.818434	-0.0329	19.20163	1.25E-41	2.76E-40	84.04039	up
GOBP_ECT	0.818338	0.081123	16.30883	1.36E-34	1.84E-33	67.82694	up
ZHENG_RE	-0.81814	0.047006	-17.9787	1.04E-38	1.86E-37	77.30682	down
REACTOM	0.817962	0.093235	17.77782	3.21E-38	5.54E-37	76.18369	up
GOBP_BILE	-0.81723	-0.13753	-21.7714	1.66E-47	5.95E-46	97.57837	down
HP_ABNOI	-0.81672	-0.07574	-18.7361	1.58E-40	3.21E-39	81.49874	down
WP_DNA_I	0.81655	-0.0594	16.71581	1.31E-35	1.90E-34	70.16638	up
REACTOM	-0.81624	-0.06046	-22.1158	2.88E-48	1.11E-46	99.32963	down
BIOCARTA	-0.81616	-4.08E-05	-21.283	2.03E-46	6.62E-45	95.07007	down
HP_INCREA	-0.81581	-0.03386	-21.9614	6.30E-48	2.36E-46	98.54623	down
GOMF_NU	-0.81551	-0.05771	-17.3869	2.89E-37	4.66E-36	73.98377	down
HEDVAT_E	-0.81534	-0.00509	-26.3972	3.44E-57	3.16E-55	119.8758	down
HP_ABNOI	-0.81497	-0.07622	-19.2574	9.22E-42	2.06E-40	84.34283	down
GOBP_POS	0.814843	-0.00699	15.39452	2.77E-32	3.27E-31	62.5087	up
GNF2_MLH	0.814694	-0.01651	16.68196	1.59E-35	2.29E-34	69.97244	up
MORI_LAR	0.814683	-0.04726	19.6429	1.15E-42	2.75E-41	86.42444	up
GOMF_AN	-0.81382	-0.14546	-16.5694	3.04E-35	4.27E-34	69.32679	down
HP_PORTA	-0.81376	-0.06675	-20.2822	3.81E-44	1.02E-42	89.83498	down
LOPEZ_ME	-0.81347	-0.04154	-17.9241	1.41E-38	2.51E-37	77.00245	down
GOBP_REG	-0.81342	-0.0272	-26.3017	5.31E-57	4.80E-55	119.4416	down
GOCC_DIH	-0.81319	-0.12233	-17.1909	8.76E-37	1.37E-35	72.87442	down
ZHOU_CEL	0.812959	0.062467	18.14616	4.11E-39	7.53E-38	78.23987	up

TRAVAGLII	0.81287	-0.06522	17.23688	6.75E-37	1.06E-35	73.13491	up
KUWANO_	-0.81261	0.174746	-15.0957	1.60E-31	1.81E-30	60.75329	down
HP_JOINT_	-0.8124	-0.08207	-22.1106	2.96E-48	1.14E-46	99.30335	down
GOBP_RES	-0.81219	-0.13139	-18.2052	2.96E-39	5.50E-38	78.56795	down
MULLIGAN	-0.81172	-0.03883	-21.6652	2.85E-47	1.00E-45	97.03592	down
REACTOM	-0.81157	-0.09112	-33.347	8.27E-70	2.84E-67	148.8859	down
GOBP_ACU	-0.81133	0.004618	-25.7812	5.78E-56	4.77E-54	117.0549	down
GOMF_PYF	-0.81114	-0.04408	-12.7113	2.48E-25	1.89E-24	46.50939	down
HALLMARI	0.810797	-0.02397	18.92142	5.73E-41	1.20E-39	82.51383	up
REACTOM	-0.81061	-0.06195	-35.1214	1.04E-72	5.18E-70	155.5474	down
WP_CONS	-0.8105	-0.06383	-23.5031	2.90E-51	1.53E-49	106.2317	down
GOMF_3_5	0.810353	0.01322	21.44333	8.90E-47	2.99E-45	95.89683	up
LUDWICZE	-0.8102	-0.02016	-18.541	4.62E-40	9.07E-39	80.42575	down
REACTOM	0.810078	-0.00298	19.63107	1.23E-42	2.92E-41	86.36084	up
TOMIDA_L	0.809877	-0.03818	16.95589	3.33E-36	5.04E-35	71.53787	up
GOBP_REG	-0.8098	0.010169	-13.733	5.29E-28	4.80E-27	52.65516	down
GOMF_INT	-0.80957	0.112271	-17.8904	1.71E-38	3.01E-37	76.81381	down
REACTOM	-0.80953	-0.12149	-16.9729	3.02E-36	4.59E-35	71.6347	down
CAIRO_HE	-0.80934	-0.06182	-40.2413	1.79E-80	4.80E-77	173.3356	down
PUJANA_B	0.808868	0.004314	20.26681	4.13E-44	1.11E-42	89.75345	up
MODULE_1	0.807746	-0.09773	11.04735	5.86E-21	3.30E-20	36.46094	up
REACTOM	-0.8075	-0.11913	-24.1988	9.97E-53	5.95E-51	109.6031	down
KOBAYASH	0.807283	-0.00682	20.23696	4.84E-44	1.29E-42	89.5953	up
GOBP_DNA	0.807009	-0.10802	13.73929	5.10E-28	4.63E-27	52.69277	up
REACTOM	0.806926	-0.01271	17.49208	1.60E-37	2.62E-36	74.57748	up
GOMF_RET	-0.80686	-0.06124	-24.4667	2.76E-53	1.77E-51	110.8862	down
GOBP_POS	0.806399	-0.02304	22.74604	1.22E-49	5.45E-48	102.4949	up
WP_FATTY	-0.80634	-0.09199	-23.2503	1.00E-50	4.98E-49	104.9917	down
GOBP_BILE	-0.80619	-0.08566	-32.3202	4.49E-68	1.21E-65	144.902	down
WP_TRYPT	-0.80607	-0.06255	-24.0056	2.53E-52	1.47E-50	108.6729	down
KEGG_PER	-0.8055	-0.13537	-24.0563	1.98E-52	1.16E-50	108.9174	down
GOCC_DN	0.805435	-0.08935	14.37227	1.16E-29	1.17E-28	56.47125	up
GOBP_DNA	0.805392	-0.01609	17.46545	1.86E-37	3.04E-36	74.42729	up
MODULE_2	0.805387	-0.01729	23.8767	4.71E-52	2.69E-50	108.0497	up
MODULE_4	-0.80534	-0.06523	-20.5349	1.00E-44	2.83E-43	91.16874	down
HP_DECRE	-0.80492	-0.07721	-15.9259	1.25E-33	1.60E-32	65.60984	down
RHODES_U	0.804241	-0.07194	17.35524	3.46E-37	5.55E-36	73.80486	up
REACTOM	0.803847	0.006804	23.96917	3.01E-52	1.74E-50	108.497	up
REACTOM	-0.80369	-0.08676	-35.9011	5.97E-74	3.51E-71	158.3895	down
HP_FOCAL	-0.80356	-0.15515	-19.2495	9.62E-42	2.15E-40	84.29999	down
GOBP_ACT	0.803054	-0.06232	17.73472	4.08E-38	7.00E-37	75.94202	up
WP_OXIDA	-0.803	-0.07754	-32.232	6.36E-68	1.70E-65	144.5554	down
GOBP_AOI	-0.80289	0.066084	-14.1396	4.65E-29	4.50E-28	55.08527	down
GOMF_FLA	0.802508	-0.06989	18.20002	3.05E-39	5.65E-38	78.53916	up
NAKAYAM	0.802495	0.038863	25.1315	1.19E-54	8.51E-53	114.0315	up
GOBP_POS	-0.802	-0.00818	-27.5372	2.07E-59	2.29E-57	124.9825	down
ZNF302_TA	0.801844	-0.06369	15.71315	4.31E-33	5.35E-32	64.37152	up
MODULE_9	-0.80124	-0.13643	-26.9016	3.52E-58	3.49E-56	122.1535	down
PUJANA_X	0.800252	0.018836	21.73005	2.05E-47	7.30E-46	97.36742	up
GOMF_AN	0.800137	-0.03147	18.83213	9.34E-41	1.93E-39	82.02536	up
GOBP_NEG	-0.79999	-0.10884	-23.3188	7.16E-51	3.59E-49	105.3286	down
SA_REG_CA	0.799611	-0.00467	22.03328	4.37E-48	1.67E-46	98.91134	up
GOBP_PER	-0.79955	-0.12593	-24.881	3.87E-54	2.63E-52	112.8526	down
GOBP_PYR	-0.79942	-0.06212	-28.6313	1.76E-61	2.38E-59	129.7486	down
GOMF_ACT	-0.79941	-0.04195	-19.1771	1.42E-41	3.14E-40	83.90725	down
HP_DECRE	-0.79928	-0.0382	-17.9142	1.50E-38	2.64E-37	76.94673	down
HP_ABNOI	-0.7991	-0.06988	-18.7433	1.52E-40	3.09E-39	81.53817	down
HP_AMBIG	-0.79892	-0.09295	-21.3126	1.75E-46	5.70E-45	95.22266	down

WP_FATTY	-0.79871	-0.13679	-18.1901	3.22E-39	5.95E-38	78.4841	down
GOMF_FAT	-0.79869	-0.05487	-19.6794	9.46E-43	2.27E-41	86.62079	down
REACTOM	0.798524	-0.07811	15.07074	1.86E-31	2.09E-30	60.60612	up
GOBP_URE	-0.79823	-0.1276	-15.8841	1.59E-33	2.02E-32	65.36701	down
HOFFMAN	0.797575	-0.02223	21.62543	3.50E-47	1.22E-45	96.83204	up
GOBP_CHC	-0.79713	-0.04792	-16.1315	3.79E-34	4.98E-33	66.80202	down
GOBP_HUI	-0.79704	-0.00829	-26.4521	2.68E-57	2.50E-55	120.1253	down
GSE15750_	0.796445	-0.0455	19.63152	1.22E-42	2.91E-41	86.36329	up
WP_KYNUI	-0.79628	0.022142	-27.709	9.72E-60	1.09E-57	125.7397	down
GOBP_PIGI	-0.79608	-0.10166	-17.8579	2.05E-38	3.59E-37	76.63185	down
GOBP_REG	-0.79605	-0.09516	-16.7592	1.02E-35	1.49E-34	70.41445	down
GOMF_OX	-0.79603	-0.09746	-30.1153	3.33E-64	5.94E-62	136.0091	down
KIM_GERM	-0.79581	-0.05009	-19.1469	1.68E-41	3.68E-40	83.74325	down
GOBP_CEL	-0.79557	-0.15005	-16.9887	2.76E-36	4.20E-35	71.72462	down
BIOCARTA	-0.79444	-0.049	-30.7286	2.66E-65	5.34E-63	138.5306	down
WP_HFE_E	-0.79379	0.094422	-16.4784	5.13E-35	7.12E-34	68.80356	down
GOCC_KIN	0.793612	0.022483	17.79631	2.89E-38	5.02E-37	76.28728	up
MODULE_:	0.793149	-0.12793	13.07986	2.68E-26	2.20E-25	48.7316	up
GOBP_ACY	-0.79224	-0.10868	-24.4387	3.16E-53	2.01E-51	110.7524	down
GOBP_NAI	-0.7922	-0.05801	-28.1312	1.53E-60	1.89E-58	127.5861	down
GOCC_NSI	0.792076	0.057506	16.98803	2.77E-36	4.22E-35	71.721	up
REACTOM	0.79174	-0.04568	13.76421	4.39E-28	4.00E-27	52.842	up
GESERICK_	-0.79166	0.066437	-18.243	2.40E-39	4.49E-38	78.77765	down
HP_STROK	-0.79133	-0.09288	-16.5925	2.66E-35	3.75E-34	69.45923	down
ODONNEL	0.791291	-0.01092	22.15276	2.39E-48	9.39E-47	99.51649	up
CAO_BLOC	0.791137	-0.19012	16.03407	6.67E-34	8.67E-33	66.2376	up
REACTOM	-0.79096	-0.10339	-29.3863	7.04E-63	1.13E-60	132.9624	down
REACTOM	-0.79094	0.046462	-19.8913	3.04E-43	7.61E-42	87.75569	down
FISCHER_G	0.790858	0.023582	25.80689	5.13E-56	4.27E-54	117.1734	up
HP_ANURI	-0.7908	-0.08301	-18.6199	2.99E-40	5.94E-39	80.86059	down
GOMF_2_II	-0.7902	-0.16182	-19.4807	2.75E-42	6.39E-41	85.55115	down
WP_ATR_S	0.789987	0.001613	17.60865	8.29E-38	1.39E-36	75.23388	up
LEE_LIVER_	-0.78975	-0.0794	-29.7236	1.70E-63	2.88E-61	134.3787	down
WENG_PO	-0.78973	-0.03162	-27.7105	9.66E-60	1.09E-57	125.7462	down
HP_METHY	-0.78925	-0.08179	-21.8655	1.03E-47	3.74E-46	98.05835	down
HP_PRECC	-0.78905	-0.09084	-17.4078	2.57E-37	4.16E-36	74.10193	down
BIOCARTA	-0.78862	0.003194	-16.086	4.93E-34	6.45E-33	66.53878	down
GOMF_Y_F	0.788548	0.000623	15.03653	2.27E-31	2.54E-30	60.40453	up
WP_MITOC	-0.78825	-0.14788	-16.3376	1.15E-34	1.57E-33	67.99298	down
WP_OXYST	-0.78813	-0.11801	-28.1223	1.59E-60	1.96E-58	127.5473	down
HP_ABNOI	-0.78811	-0.06868	-22.5005	4.15E-49	1.75E-47	101.2678	down
GSE13547_	0.787283	-0.02355	21.17564	3.54E-46	1.13E-44	94.51447	up
HOUSTIS_I	-0.78698	-0.10591	-22.496	4.24E-49	1.78E-47	101.2448	down
PID_PLK1_I	0.78659	-0.00865	21.46271	8.06E-47	2.72E-45	95.99656	up
GOBP_FAT	-0.78656	0.06334	-15.9579	1.04E-33	1.34E-32	65.79555	down
GOCC_TRI	-0.78622	-0.12126	-20.1778	6.62E-44	1.75E-42	89.28129	down
HP_ATROF	-0.78611	0.027181	-14.5887	3.21E-30	3.35E-29	57.75661	down
HP_MITOC	-0.78607	-0.03586	-15.2965	4.93E-32	5.72E-31	61.93351	down
REACTOM	0.785958	-0.1006	13.11925	2.12E-26	1.74E-25	48.96877	up
GUTIERREZ	-0.78523	0.156331	-12.542	6.91E-25	5.10E-24	45.48739	down
HP_DECRE	-0.78476	0.017573	-15.4489	2.02E-32	2.40E-31	62.82718	down
GOBP_DNA	0.78431	-0.01653	11.80957	5.82E-23	3.78E-22	41.06162	up
MODULE_!	-0.78418	-0.18098	-22.1799	2.08E-48	8.23E-47	99.65372	down
GOBP_POS	0.783973	0.011446	14.64118	2.35E-30	2.47E-29	58.06781	up
GOBP_REP	0.783535	-0.05349	14.84709	6.96E-31	7.56E-30	59.28648	up
HP_APLAS	0.782839	-0.00478	18.17673	3.47E-39	6.39E-38	78.40976	up
GOBP_VER	-0.78256	-0.13424	-18.2456	2.36E-39	4.42E-38	78.79241	down
GOBP_REC	-0.78239	0.039358	-18.5758	3.81E-40	7.51E-39	80.61763	down

GOBP_REG	0.78238	-0.08808	18.55253	4.34E-40	8.52E-39	80.48935	up
DUTERTRE	0.782343	-0.01555	20.53405	1.01E-44	2.84E-43	91.16428	up
GOCC_CH	-0.78213	-0.1082	-16.4842	4.96E-35	6.89E-34	68.83738	down
RICKMAN_	0.78204	-0.03457	16.2812	1.60E-34	2.15E-33	67.66747	up
GSE14415_	0.782002	-0.01607	23.39474	4.93E-51	2.53E-49	105.7012	up
GOBP_SER	-0.78195	-0.05913	-17.5113	1.43E-37	2.37E-36	74.68567	down
GOMF_PEI	-0.78178	0.081154	-18.3517	1.31E-39	2.51E-38	79.38003	down
GOBP_PRC	-0.78174	-0.08224	-15.4285	2.27E-32	2.69E-31	62.70803	down
PATIL_LIVE	0.781207	-0.05963	29.36113	7.83E-63	1.24E-60	132.8561	up
GOMF_OX	-0.78081	-0.09568	-17.7632	3.48E-38	5.98E-37	76.10193	down
CHIANG_L	-0.7803	-0.09831	-22.2713	1.31E-48	5.28E-47	100.1149	down
BENPORAT	0.780294	-0.05494	18.86454	7.82E-41	1.62E-39	82.20279	up
KEGG_HIST	-0.78002	-0.08405	-26.0097	2.02E-56	1.71E-54	118.1063	down
HP_TRACH	0.779265	0.00723	18.43099	8.48E-40	1.64E-38	79.81866	up
GOCC_BLC	-0.77926	-0.05512	-31.3491	2.15E-66	4.62E-64	141.0436	down
GOBP_DN/	0.77914	-0.00935	14.61081	2.82E-30	2.95E-29	57.8878	up
GOBP_RET	-0.77891	0.067883	-13.6013	1.17E-27	1.04E-26	51.86583	down
GOBP_PYR	-0.77848	-0.07462	-21.8406	1.16E-47	4.23E-46	97.93149	down
REACTOM	-0.77827	-0.05796	-15.0453	2.16E-31	2.42E-30	60.45619	down
GOBP_RES	-0.77785	0.021376	-16.2534	1.87E-34	2.51E-33	67.5071	down
XU_HGF_SI	0.777551	0.036527	22.28131	1.25E-48	5.03E-47	100.1656	up
GOBP_DN/	0.775997	0.021978	13.15228	1.74E-26	1.44E-25	49.16761	up
REACTOM	0.775997	0.021978	13.15228	1.74E-26	1.44E-25	49.16761	up
GOBP_REG	-0.77567	0.067362	-14.5197	4.84E-30	4.96E-29	57.34739	down
BIOCARTA	0.775176	0.036763	17.14941	1.11E-36	1.72E-35	72.63874	up
MONTERC	-0.77455	0.043987	-19.5719	1.69E-42	3.96E-41	86.04262	down
MEBARKI_I	0.774116	0.024225	26.96682	2.63E-58	2.62E-56	122.4459	up
GOBP_BILE	-0.77369	-0.05155	-20.7953	2.56E-45	7.65E-44	92.53488	down
HP_ABNOI	-0.77353	-0.13909	-19.7341	7.05E-43	1.71E-41	86.91426	down
MODULE_1	0.773428	0.034306	18.02449	8.08E-39	1.45E-37	77.56244	up
HP_LOSS_(	0.773192	0.029623	15.48122	1.67E-32	1.99E-31	63.01653	up
MODULE_3	-0.77317	-0.06071	-18.799	1.12E-40	2.30E-39	81.84362	down
GOBP_POS	-0.7731	0.057871	-15.9905	8.58E-34	1.11E-32	65.98466	down
HP_PROLC	-0.77308	-0.05	-16.7634	1.00E-35	1.46E-34	70.4387	down
GOBP_NEC	-0.77245	-0.03557	-15.8873	1.56E-33	1.99E-32	65.38522	down
REACTOM	0.772413	-0.08895	14.17138	3.85E-29	3.74E-28	55.27498	up
HP_HYPO(	-0.7724	-0.09348	-13.1985	1.31E-26	1.10E-25	49.44573	down
GOBP_CEL	0.77239	-0.04728	18.61354	3.10E-40	6.15E-39	80.8253	up
REACTOM	-0.77237	0.084969	-15.1361	1.27E-31	1.43E-30	60.99069	down
GOBP_REG	-0.77223	-0.00518	-28.0587	2.10E-60	2.56E-58	127.2707	down
GOBP_NAI	-0.77217	-0.0014	-21.1418	4.22E-46	1.34E-44	94.33906	down
GOBP_POS	0.771892	-0.07414	11.72842	9.52E-23	6.07E-22	40.57118	up
GOBP_HIG	-0.77189	-0.07536	-23.1673	1.51E-50	7.35E-49	104.5831	down
REACTOM	0.771843	0.038335	16.69737	1.46E-35	2.10E-34	70.06072	up
DESCARTE	-0.77179	0.111136	-21.3303	1.59E-46	5.20E-45	95.31445	down
TFAM_TAR	-0.77169	-0.01568	-14.1923	3.40E-29	3.31E-28	55.39961	down
GOBP_LON	-0.77158	-0.00598	-25.3589	4.10E-55	3.12E-53	115.0953	down
GOBP_ARC	-0.77148	-0.06701	-23.1732	1.47E-50	7.17E-49	104.6118	down
GOMF_HEI	-0.77146	-0.00379	-13.8859	2.12E-28	1.97E-27	53.57027	down
WP_EICOS	-0.77142	0.003204	-27.2921	6.15E-59	6.52E-57	123.8972	down
KEGG_FOL	-0.77132	-0.0873	-16.9254	3.96E-36	5.95E-35	71.36418	down
GOCC_INT	-0.77116	-0.17124	-14.2845	1.96E-29	1.94E-28	55.94905	down
GOCC_EXT	-0.77101	0.115708	-11.7424	8.74E-23	5.60E-22	40.65568	down
GOBP_HIS	0.770781	0.035405	24.60484	1.43E-53	9.42E-52	111.5439	up
ICHIBA_GR	-0.77059	-0.02403	-24.2834	6.64E-53	4.05E-51	110.0092	down
GOBP_RES	-0.76932	-0.04266	-15.6761	5.34E-33	6.59E-32	64.15566	down
HP_PYRID(	0.767896	-0.02123	15.90378	1.42E-33	1.81E-32	65.4812	up
HP_INCRE/	-0.7678	0.026141	-23.8491	5.39E-52	3.05E-50	107.916	down

GOBP_FOI	-0.7677	-0.05968	-14.7574	1.18E-30	1.27E-29	58.75602	down
HP_HYPER	-0.76674	-0.15854	-13.6415	9.16E-28	8.19E-27	52.10655	down
WP_THE_A	-0.7663	-0.1764	-18.0496	7.03E-39	1.27E-37	77.70245	down
WP_RETIN	0.766278	0.005774	18.99672	3.80E-41	8.08E-40	82.92502	up
GOMF_AC	-0.76613	-0.14448	-14.8537	6.69E-31	7.28E-30	59.32548	down
GOBP_WA	-0.76606	-0.08572	-15.2271	7.41E-32	8.52E-31	61.52615	down
REACTOM	-0.76596	-0.06822	-21.7892	1.51E-47	5.46E-46	97.66961	down
GOBP_NEC	0.765878	-0.0528	20.44061	1.65E-44	4.60E-43	90.67201	up
GOBP_ATF	-0.76553	0.145211	-18.7127	1.80E-40	3.62E-39	81.37062	down
HP_ABSEN	0.765234	-0.01287	16.82168	7.17E-36	1.06E-34	70.77196	up
GOBP_NEC	-0.76512	-0.07763	-13.3283	6.01E-27	5.12E-26	50.22672	down
HP_PERSIS	-0.76507	-0.07141	-19.3829	4.67E-42	1.07E-40	85.02273	down
GOMF_OX	-0.76458	-0.1351	-22.3624	8.30E-49	3.39E-47	100.5742	down
GOBP_REG	-0.76428	-0.19901	-11.526	3.24E-22	2.00E-21	39.34857	down
GOBP_REG	0.763665	-0.04925	21.43886	9.11E-47	3.05E-45	95.87384	up
GOBP_RES	-0.76366	-0.09124	-13.7943	3.67E-28	3.36E-27	53.02195	down
SARRIO_EI	0.763588	-0.00048	22.39233	7.14E-49	2.93E-47	100.7245	up
SOBOLEV_	-0.76347	0.052548	-16.0548	5.91E-34	7.70E-33	66.35798	down
GOBP_CITI	-0.76323	-0.10626	-13.7597	4.51E-28	4.11E-27	52.81493	down
GOBP_NEC	-0.76303	-0.06253	-19.436	3.51E-42	8.09E-41	85.30951	down
GOBP_OX	-0.76297	-0.13608	-18.2578	2.21E-39	4.14E-38	78.85963	down
KEGG_DN	0.762844	-0.08735	14.69782	1.68E-30	1.79E-29	58.40339	up
WINNEPEN	0.762369	-0.0529	17.63742	7.05E-38	1.19E-36	75.39566	up
GOBP_ANI	-0.76228	-0.08612	-27.4527	3.02E-59	3.30E-57	124.6089	down
MODULE_	-0.76197	-0.10331	-22.6645	1.83E-49	7.94E-48	102.0881	down
GOMF_PRI	-0.76143	0.006144	-17.2165	7.58E-37	1.19E-35	73.01948	down
GOCC_SM	-0.7612	0.01126	-15.718	4.19E-33	5.20E-32	64.39991	down
WP_VITAM	-0.76117	-0.00759	-27.8853	4.48E-60	5.19E-58	126.513	down
BIOCARTA	-0.76103	-0.07733	-22.4649	4.96E-49	2.06E-47	101.0891	down
GOBP_FAT	-0.76095	-0.10751	-21.229	2.69E-46	8.66E-45	94.79071	down
LI_WILMS_	0.760858	0.012012	24.2084	9.51E-53	5.73E-51	109.6495	up
GOBP_STE	-0.76034	-0.09394	-14.4792	6.16E-30	6.28E-29	57.10657	down
MODULE_	0.760033	-0.04299	13.10961	2.24E-26	1.84E-25	48.91071	up
HP_XANTH	-0.75989	-0.06424	-20.4717	1.40E-44	3.91E-43	90.83583	down
GOBP_NEC	-0.75971	-0.05055	-15.855	1.89E-33	2.39E-32	65.19778	down
REACTOM	-0.75951	0.049926	-15.2511	6.44E-32	7.43E-31	61.66699	down
HALLMAR	0.759083	0.015196	21.25233	2.38E-46	7.68E-45	94.91141	up
GOCC_FEN	0.759043	0.046426	14.30536	1.73E-29	1.72E-28	56.07315	up
HALLMAR	-0.75892	-0.09867	-26.0167	1.95E-56	1.66E-54	118.1387	down
GOCC_LO	-0.75876	-0.01593	-16.4666	5.48E-35	7.60E-34	68.73598	down
REACTOM	-0.75824	-0.12506	-14.066	7.21E-29	6.91E-28	54.64648	down
VERNELL_I	0.757828	0.017657	19.09122	2.27E-41	4.91E-40	83.44004	up
GNF2_MSI	0.757672	-0.06508	14.29772	1.81E-29	1.80E-28	56.02766	up
REACTOM	-0.75703	-0.00901	-19.8739	3.34E-43	8.31E-42	87.66247	down
ZERBINI_R	0.756933	-0.10997	14.5478	4.10E-30	4.24E-29	57.51401	up
REACTOM	-0.75686	-0.05175	-27.1226	1.31E-58	1.34E-56	123.1424	down
GOMF_OX	-0.75673	-0.1387	-13.6407	9.20E-28	8.23E-27	52.10188	down
GOBP_COI	-0.75654	0.084213	-15.7928	2.71E-33	3.40E-32	64.83548	down
GOBP_L_A	-0.75648	-0.07049	-19.8674	3.46E-43	8.59E-42	87.62805	down
MORF_BUI	0.756288	-0.04144	16.61413	2.35E-35	3.33E-34	69.58358	up
GOBP_EST	0.756074	-0.07179	15.8295	2.19E-33	2.76E-32	65.04926	up
MORF_BUI	0.755688	-0.01626	18.97519	4.28E-41	9.07E-40	82.8075	up
WP_NUCLI	-0.75529	-0.0549	-30.2725	1.74E-64	3.15E-62	136.6592	down
GOMF_OX	-0.75423	-0.03814	-15.6706	5.52E-33	6.80E-32	64.12357	down
HP_ABNOI	0.754065	-0.03201	19.71509	7.81E-43	1.89E-41	86.81214	up
GOBP_ASF	-0.75366	-0.07931	-23.796	6.97E-52	3.92E-50	107.6583	down
HP_TAPER	0.753578	-0.00263	11.94805	2.52E-23	1.67E-22	41.89856	up
GOBP_L_SI	-0.7535	-0.06318	-16.3242	1.25E-34	1.69E-33	67.91574	down

SASSON_F	-0.75334	0.01986	-17.23	7.02E-37	1.10E-35	73.09583	down
BROWNE_	-0.75322	0.030154	-24.3419	5.02E-53	3.13E-51	110.2893	down
GOBP_NEC	-0.75314	0.076152	-19.7168	7.74E-43	1.87E-41	86.82154	down
GOMF_INT	-0.75283	-0.12543	-15.2406	6.84E-32	7.89E-31	61.60543	down
GOBP_HIS	0.752776	-0.04608	20.61731	6.51E-45	1.88E-43	91.60198	up
GOMF_FO	0.752708	-0.0336	17.43073	2.26E-37	3.67E-36	74.23138	up
MODULE_	-0.75233	-0.03186	-31.9955	1.62E-67	4.18E-65	143.622	down
GOBP_ETH	-0.75216	-0.00553	-22.1365	2.59E-48	1.01E-46	99.43415	down
REACTOM	-0.75213	-0.07734	-15.0856	1.70E-31	1.92E-30	60.69361	down
HP_GENER	-0.7521	-0.12509	-18.6855	2.09E-40	4.17E-39	81.22081	down
GOBP_RET	-0.75184	-0.0876	-29.5831	3.07E-63	5.11E-61	133.7903	down
GOBP_AN	0.751679	0.008818	11.43225	5.72E-22	3.46E-21	38.78227	up
WP_METAI	-0.75135	-0.07974	-17.91	1.53E-38	2.70E-37	76.92358	down
KEGG_MET	-0.75134	-0.09565	-23.4729	3.36E-51	1.76E-49	106.084	down
GOBP_MIT	-0.75129	0.07435	-12.8918	8.35E-26	6.58E-25	47.5985	down
HP_DECRE	-0.75085	0.031133	-19.7221	7.52E-43	1.82E-41	86.84984	down
KAPOSI_LI	0.749851	0.04609	18.939	5.21E-41	1.10E-39	82.6099	up
GOCC_XY_	0.749649	0.017825	22.8	9.30E-50	4.24E-48	102.7636	up
GOBP_CYT	-0.74964	-0.11033	-16.2653	1.75E-34	2.35E-33	67.57542	down
GOMF_MIS	0.749418	-0.00627	14.63692	2.41E-30	2.53E-29	58.04256	up
HP_ABNOI	-0.74911	-0.08395	-21.6183	3.63E-47	1.26E-45	96.79536	down
GOBP_MIT	0.749097	-0.04023	16.01435	7.47E-34	9.70E-33	66.12325	up
GOBP_MA	-0.74865	0.041945	-14.372	1.16E-29	1.17E-28	56.46944	down
GOBP_HYI	-0.74842	-0.06605	-14.7875	9.90E-31	1.06E-29	58.93388	down
REACTOM	-0.74831	-0.18216	-17.1212	1.30E-36	2.01E-35	72.47852	down
BIOCARTA	0.748312	0.006549	20.32415	3.05E-44	8.31E-43	90.05691	up
GOBP_REG	0.748214	0.072751	20.22004	5.29E-44	1.41E-42	89.50559	up
REACTOM	-0.74807	-0.0835	-33.3041	9.76E-70	3.31E-67	148.7212	down
GOCC_LIP	-0.74765	0.030982	-12.7731	1.71E-25	1.32E-24	46.88245	down
HP_DEEP_	-0.74755	-0.0632	-23.224	1.14E-50	5.64E-49	104.8623	down
HP_ABNOI	-0.74747	-0.04691	-24.893	3.65E-54	2.51E-52	112.9091	down
GOBP_VAL	-0.74734	-0.12706	-17.4517	2.01E-37	3.27E-36	74.34956	down
GOBP_GLU	-0.74642	-0.04398	-22.6393	2.07E-49	8.95E-48	101.9621	down
GOBP_REG	0.745101	0.00238	15.57734	9.51E-33	1.15E-31	63.5787	up
WP_DNA_I	0.744927	0.003065	27.9639	3.18E-60	3.77E-58	126.8568	up
REACTOM	-0.74483	-0.12144	-18.7374	1.57E-40	3.19E-39	81.50601	down
WP_NEUR	-0.7444	-0.00564	-21.3381	1.53E-46	5.02E-45	95.35461	down
GOBP_MIC	0.744389	-0.00525	15.85493	1.89E-33	2.39E-32	65.1972	up
GOBP_NEC	-0.74401	0.016457	-23.7302	9.59E-52	5.30E-50	107.3388	down
HP_ABNOI	-0.74396	-0.10286	-20.723	3.74E-45	1.10E-43	92.15652	down
ZNF214_T	-0.74387	-0.01444	-11.9945	1.90E-23	1.28E-22	42.1795	down
GOBP_REG	-0.74377	-0.11478	-24.2365	8.31E-53	5.01E-51	109.7845	down
HP_EPISCL	-0.74343	0.026576	-12.5853	5.32E-25	3.95E-24	45.74936	down
PID_FANC	0.743358	0.007558	19.03734	3.05E-41	6.50E-40	83.14655	up
GSE27241_	0.742984	0.002897	23.06212	2.53E-50	1.21E-48	104.0637	up
HP_INABIL	-0.74285	-0.03463	-16.5289	3.83E-35	5.36E-34	69.09401	down
BUSSLING	-0.74258	-0.05252	-29.24	1.31E-62	2.01E-60	132.3441	down
GOBP_MIN	-0.74232	0.059876	-17.1155	1.34E-36	2.07E-35	72.44624	down
GOMF_NU	0.7422	-0.0015	16.3204	1.27E-34	1.73E-33	67.89372	up
KEGG_HOI	0.741927	-0.06738	18.56556	4.04E-40	7.95E-39	80.56116	up
HP_ELEVA	-0.74161	-0.12214	-19.3431	5.79E-42	1.32E-40	84.80737	down
PID_AURO	0.741364	-0.0131	19.61098	1.37E-42	3.24E-41	86.25279	up
REACTOM	-0.7411	0.022952	-19.7452	6.65E-43	1.62E-41	86.97385	down
CHEMNITZ	0.740918	-0.03338	23.72706	9.74E-52	5.37E-50	107.3236	up
GOBP_GLU	-0.74075	-0.12365	-20.2011	5.85E-44	1.55E-42	89.40496	down
GOBP_DN	0.740649	-0.05963	17.55959	1.09E-37	1.81E-36	74.95785	up
GOBP_IND	-0.73905	0.039646	-15.4834	1.65E-32	1.97E-31	63.0295	down
CHEN_HO	-0.73887	0.013267	-16.3693	9.60E-35	1.32E-33	68.17565	down

LIAO_HAV	0.738855	0.078669	26.10712	1.29E-56	1.13E-54	118.553	up
REACTOM	0.73845	0.003013	16.32625	1.23E-34	1.67E-33	67.92742	up
BIOCARTA	0.738396	-0.02537	15.51337	1.38E-32	1.66E-31	63.20468	up
GOBP_REG	0.73813	-0.05347	12.31394	2.75E-24	1.96E-23	44.11	up
GOBP_IND	-0.73801	-0.0407	-25.293	5.58E-55	4.17E-53	114.7876	down
GOBP_REL	-0.73793	0.109276	-13.9914	1.13E-28	1.07E-27	54.20101	down
GOBP_NU(	-0.73784	-0.14877	-15.627	7.12E-33	8.72E-32	63.86889	down
REACTOM	0.737593	-0.00854	23.07812	2.34E-50	1.12E-48	104.1428	up
BOYAULT_	0.737506	-0.06482	14.81731	8.30E-31	8.97E-30	59.11047	up
MODULE_!	-0.73746	0.042618	-20.3684	2.41E-44	6.64E-43	90.29094	down
HP_FLUCTI	-0.73728	-0.14273	-17.4022	2.65E-37	4.29E-36	74.07033	down
GOBP_PUT	-0.73722	-0.06739	-16.1915	2.68E-34	3.56E-33	67.14898	down
GOBP_EST	-0.73695	-0.08215	-22.8067	8.99E-50	4.10E-48	102.797	down
GOBP_PRC	-0.73671	-0.04133	-20.5507	9.24E-45	2.61E-43	91.25205	down
GOCC_TEL	0.736663	-0.08704	17.31346	4.38E-37	6.99E-36	73.56858	up
WP_PREGI	-0.73649	-0.04525	-21.9805	5.72E-48	2.16E-46	98.64338	down
BOYAULT_	0.736317	-0.03434	20.86835	1.75E-45	5.29E-44	92.91644	up
GOBP_PER	-0.73618	-0.12979	-21.9686	6.07E-48	2.28E-46	98.58308	down
GOBP_LOV	-0.73611	0.000276	-19.4912	2.60E-42	6.06E-41	85.60757	down
WP_DNA_I	0.735776	0.006839	21.26231	2.26E-46	7.31E-45	94.96303	up
GOCC_CY(	0.735745	0.052613	14.78449	1.01E-30	1.08E-29	58.91634	up
GOBP_DIC	-0.73568	-0.00513	-19.9522	2.20E-43	5.56E-42	88.08086	down
YAMASHIT	-0.73526	-0.18761	-18.6687	2.29E-40	4.57E-39	81.12888	down
ZSCAN26_	0.735026	-0.05565	14.6842	1.82E-30	1.93E-29	58.32272	up
FUJII_YBX1	0.734817	-0.01127	21.72602	2.09E-47	7.44E-46	97.3468	up
REACTOM	0.733953	-0.03373	21.07414	6.00E-46	1.88E-44	93.98794	up
GOBP_UM	-0.73352	-0.05694	-14.0351	8.68E-29	8.28E-28	54.46173	down
HP_HYPER	-0.73346	0.023763	-23.0037	3.38E-50	1.60E-48	103.7746	down
GOBP_DIC	-0.73335	-0.06987	-19.7434	6.71E-43	1.63E-41	86.96408	down
GOMF_DN	0.733266	0.014809	21.91247	8.08E-48	2.98E-46	98.29758	up
chr9p23	-0.73315	0.013444	-13.2138	1.20E-26	1.00E-25	49.53813	down
WP_DOPA	-0.7331	-0.01226	-17.63	7.35E-38	1.24E-36	75.35375	down
REACTOM	0.732846	0.019571	20.09748	1.01E-43	2.63E-42	88.85484	up
HP_INTERS	0.732272	0.002824	15.21035	8.18E-32	9.38E-31	61.42765	up
GOMF_URI	-0.73223	-0.04502	-14.5818	3.35E-30	3.48E-29	57.71598	down
WP_SOMA	-0.73104	0.104704	-14.4905	5.76E-30	5.88E-29	57.17384	down
HOWARD_	-0.73096	0.101494	-12.2165	4.95E-24	3.46E-23	43.52127	down
MANNO_N	0.73075	0.020599	24.08507	1.72E-52	1.01E-50	109.0562	up
FUJIWARA	-0.73064	-0.07306	-16.0254	7.01E-34	9.11E-33	66.1872	down
HP_CHROI	0.730138	-0.0258	15.1667	1.06E-31	1.20E-30	61.17096	up
GOBP_DIC	-0.73006	-0.07182	-25.6975	8.51E-56	6.95E-54	116.668	down
GOCC_PLA	-0.73004	-0.03175	-26.9293	3.11E-58	3.09E-56	122.2779	down
WP_HUMA	-0.72991	0.001572	-28.1915	1.18E-60	1.48E-58	127.8482	down
KEGG_TYR	-0.7299	-0.08606	-29.9213	7.46E-64	1.28E-61	135.2037	down
GOBP_MET	0.729698	-0.04898	19.85341	3.73E-43	9.22E-42	87.55317	up
GOMF_FAT	-0.72945	-0.11194	-23.443	3.89E-51	2.02E-49	105.9375	down
GOBP_ALP	-0.7293	-0.07511	-25.6826	9.12E-56	7.43E-54	116.599	down
HP_BLEEDI	-0.72896	-0.02542	-12.7015	2.63E-25	2.00E-24	46.45045	down
GOBP_RES	-0.72879	0.057682	-11.7572	8.00E-23	5.13E-22	40.74498	down
YANG_BRE	-0.72856	-0.07628	-29.9596	6.36E-64	1.10E-61	135.3631	down
WP_LIVER_	-0.72836	-0.11179	-13.3738	4.57E-27	3.92E-26	50.50016	down
GOBP_PUF	-0.72769	-0.13727	-15.6256	7.18E-33	8.78E-32	63.86079	down
MODULE_:	-0.72763	0.087529	-14.3424	1.39E-29	1.39E-28	56.29348	down
GOBP_SMA	-0.72743	-0.07211	-33.3874	7.08E-70	2.46E-67	149.0407	down
GOBP_MA	0.727392	0.05035	14.26632	2.18E-29	2.16E-28	55.84067	up
KEGG_PHE	-0.72727	-0.07803	-23.2039	1.26E-50	6.21E-49	104.7634	down
HP_DUOD	0.727033	-0.0016	17.17389	9.65E-37	1.50E-35	72.77768	up
MODULE_:	-0.72628	-0.07545	-37.1239	7.51E-76	8.65E-73	162.7466	down

GOBP_VIT/	-0.72627	-0.08601	-13.1157	2.16E-26	1.78E-25	48.94756	down
GOCC_TR/	0.726177	-0.0632	15.35053	3.59E-32	4.20E-31	62.25078	up
HP_INCRE/	-0.72614	-0.03374	-25.178	9.56E-55	6.98E-53	114.2498	down
HP_HIGH_I	-0.72603	0.041146	-15.8679	1.75E-33	2.22E-32	65.27235	down
GOCC_HA	0.725838	-0.03277	14.3912	1.04E-29	1.04E-28	56.58381	up
GOBP_URC	-0.72573	-0.08236	-16.0939	4.71E-34	6.18E-33	66.58427	down
REACTOM	-0.72562	-0.07527	-13.8877	2.10E-28	1.95E-27	53.58091	down
GOBP_PYR	-0.72559	-0.05646	-26.251	6.69E-57	5.96E-55	119.2105	down
HP_ABNOI	0.725552	0.084863	20.36063	2.52E-44	6.90E-43	90.2498	up
WEST_ADF	-0.72552	0.05431	-22.1334	2.63E-48	1.02E-46	99.41844	down
HP_PATELI	0.725236	0.028452	21.56467	4.77E-47	1.65E-45	96.52045	up
REACTOM	0.725207	0.004441	18.04862	7.07E-39	1.28E-37	77.69695	up
REACTOM	0.724981	-0.06849	11.98111	2.06E-23	1.38E-22	42.09842	up
GOMF_OX	-0.7247	-0.05431	-23.1604	1.56E-50	7.59E-49	104.5491	down
GOMF_DN	0.724629	-0.07846	15.21696	7.86E-32	9.03E-31	61.4665	up
HP_APLAS	0.72458	0.042949	27.29708	6.01E-59	6.41E-57	123.9192	up
GOBERT_C	0.724531	-0.0228	22.38987	7.23E-49	2.96E-47	100.7121	up
HP_JUVEN	0.724253	-0.05217	19.06827	2.57E-41	5.52E-40	83.31507	up
GOBP_REG	-0.7241	-0.00322	-28.9639	4.23E-62	6.15E-60	131.1717	down
GOMF_CA	-0.72406	-0.25836	-13.8132	3.27E-28	3.01E-27	53.13518	down
GOMF_ME	-0.72337	-0.1206	-18.131	4.47E-39	8.17E-38	78.15581	down
GOCC_HA	-0.72329	0.060843	-14.5072	5.21E-30	5.34E-29	57.27292	down
WENG_PO	-0.72259	-0.04553	-22.3383	9.37E-49	3.81E-47	100.4528	down
HP_AMINC	-0.72246	-0.08345	-23.6339	1.53E-51	8.34E-50	106.8701	down
GOCC_SPI	0.722268	0.008452	25.78507	5.68E-56	4.70E-54	117.0727	up
GROSS_HY	-0.7222	0.035506	-15.9922	8.50E-34	1.10E-32	65.99483	down
GOBP_REC	-0.72214	0.042897	-13.2323	1.07E-26	8.99E-26	49.64887	down
AMIT_SERU	-0.72212	0.093289	-18.6673	2.31E-40	4.60E-39	81.12079	down
GOBP_REL	-0.72183	0.058497	-13.8895	2.07E-28	1.93E-27	53.5915	down
GOMF_LOI	-0.7218	0.015707	-13.1016	2.35E-26	1.93E-25	48.86275	down
BIOCARTA	0.721691	0.067207	18.3581	1.27E-39	2.43E-38	79.41559	up
HP_HYPOK	-0.72167	-0.07168	-16.2544	1.86E-34	2.50E-33	67.51246	down
GOBP_REG	0.721595	-0.07668	16.61885	2.29E-35	3.25E-34	69.61065	up
BIOCARTA	0.721548	-0.01352	20.94429	1.18E-45	3.60E-44	93.31248	up
GOBP_REG	0.72152	0.069744	13.86281	2.43E-28	2.25E-27	53.43215	up
ABRAMSO	0.721503	0.020028	16.85498	5.92E-36	8.79E-35	70.9622	up
GOBP_NEC	0.72137	-0.03935	12.89612	8.13E-26	6.41E-25	47.62446	up
GOBP_CHI	0.720895	-0.09583	19.95013	2.22E-43	5.62E-42	88.06987	up
GOBP_SCH	-0.72076	-0.07843	-11.6078	1.98E-22	1.23E-21	39.84265	down
HP_BRANC	0.720629	-0.11419	13.24915	9.68E-27	8.15E-26	49.75052	up
REACTOM	-0.7201	-0.13265	-13.165	1.61E-26	1.33E-25	49.24427	down
chr16q13	-0.71988	0.040662	-14.7548	1.20E-30	1.29E-29	58.74062	down
HP_ABNOI	0.719343	0.066913	18.42305	8.86E-40	1.71E-38	79.7748	up
MODULE_2	-0.71909	-0.07565	-26.2571	6.50E-57	5.81E-55	119.2383	down
GOMF_MC	-0.71902	-0.06829	-32.962	3.66E-69	1.11E-66	147.4036	down
chr6q26	-0.71902	0.066875	-12.8466	1.10E-25	8.57E-25	47.32593	down
GOCC_CO	0.719006	-0.03013	20.7151	3.90E-45	1.14E-43	92.11498	up
HP_DECRE	-0.71894	-0.09012	-11.9353	2.72E-23	1.80E-22	41.82165	down
GOBP_EST	0.718929	0.026973	16.26024	1.80E-34	2.42E-33	67.54644	up
GOCC_PRE	0.718865	-0.03937	15.57983	9.38E-33	1.14E-31	63.59323	up
HP_RECUR	-0.71848	-0.04674	-19.8913	3.04E-43	7.61E-42	87.75578	down
REACTOM	-0.71843	-0.07396	-16.9401	3.65E-36	5.50E-35	71.44785	down
GOBP_REG	-0.71813	-0.0799	-19.2679	8.70E-42	1.95E-40	84.40021	down
LE_NEURO	0.718047	-0.08391	16.23895	2.04E-34	2.72E-33	67.42349	up
LIAO_MET/	0.717771	0.02448	38.61823	4.18E-78	6.13E-75	167.9125	up
KEGG_BIO!	-0.71758	-0.08123	-15.501	1.49E-32	1.78E-31	63.13213	down
GOCC_MIT	0.717411	-0.05438	17.02652	2.23E-36	3.41E-35	71.94016	up
GOBP_REG	0.717268	-0.03696	13.99949	1.07E-28	1.02E-27	54.24919	up

WP_FOLAT	-0.71695	-0.0225	-29.2488	1.26E-62	1.94E-60	132.3815	down
RANKIN_A	-0.71685	-0.05746	-11.0441	5.98E-21	3.37E-20	36.44125	down
HOEK_B_C	-0.71684	-0.07288	-9.80256	1.03E-17	4.62E-17	29.01647	down
HP_KETOS	-0.71665	-0.03128	-28.9911	3.77E-62	5.55E-60	131.2876	down
GOMF_HIS	0.716619	-0.00174	14.34106	1.40E-29	1.40E-28	56.28558	up
WEST_ADR	0.716615	-0.03557	18.69093	2.03E-40	4.06E-39	81.25085	up
GOBP_CEL	-0.71648	0.060961	-22.0184	4.72E-48	1.80E-46	98.83562	down
HP_INCRE	-0.71647	-0.10603	-15.3266	4.13E-32	4.82E-31	62.11045	down
HP_GENER	-0.71615	-0.08784	-15.0363	2.28E-31	2.54E-30	60.40305	down
WP_SELEN	-0.71593	-0.03924	-28.294	7.55E-61	9.63E-59	128.2928	down
GOBP_NEC	0.715833	-0.0038	16.86171	5.70E-36	8.48E-35	71.00062	up
MODULE_1	0.715772	-0.03157	22.142	2.52E-48	9.85E-47	99.46208	up
REACTOM	0.715677	-0.03218	15.97917	9.17E-34	1.18E-32	65.9191	up
HP_ABNOI	-0.71541	-0.09641	-12.716	2.41E-25	1.84E-24	46.53825	down
GOMF_H4_	0.71536	-0.03614	19.97787	1.92E-43	4.88E-42	88.21785	up
HP_CALCIU	-0.71534	-0.05692	-18.3559	1.28E-39	2.46E-38	79.40324	down
HP_ASYMN	0.715313	-0.01077	14.74409	1.28E-30	1.37E-29	58.67733	up
GOBP_RET	-0.71504	-0.01673	-28.1394	1.48E-60	1.84E-58	127.6218	down
HP_RECUR	-0.71474	-0.00782	-14.9552	3.68E-31	4.06E-30	59.92467	down
BIOCARTA	-0.7145	-0.05734	-23.0819	2.30E-50	1.10E-48	104.1613	down
GOBP_NEC	0.714467	-0.048	22.00269	5.11E-48	1.93E-46	98.75612	up
TDRD3_TA	0.714242	0.016982	10.3074	5.05E-19	2.48E-18	32.02002	up
GOBP_CHA	0.714233	0.003554	14.37166	1.17E-29	1.17E-28	56.46763	up
GOMF_CYC	0.714166	0.035316	18.30208	1.73E-39	3.27E-38	79.10531	up
MODULE_2	-0.71411	-0.09749	-27.3626	4.50E-59	4.87E-57	124.21	down
REACTOM	0.713822	0.008975	18.0561	6.78E-39	1.23E-37	77.73861	up
GOBP_PYR	-0.71321	-0.04391	-14.4982	5.50E-30	5.62E-29	57.21977	down
GOMF_LIP	-0.71318	-0.0997	-12.0526	1.34E-23	9.07E-23	42.53029	down
MYLLYKAN	0.712944	-0.00659	17.68398	5.43E-38	9.25E-37	75.65721	up
HP_CORTIC	0.712853	-0.06	14.83067	7.67E-31	8.32E-30	59.18943	up
GOBP_REG	0.711442	-0.02665	21.95967	6.35E-48	2.38E-46	98.53762	up
FOURNIER	-0.71116	-0.05636	-23.5838	1.96E-51	1.05E-49	106.6257	down
BIOCARTA	-0.71106	0.056096	-21.6049	3.88E-47	1.35E-45	96.72655	down
GOBP_REG	0.710837	-0.04088	20.92385	1.31E-45	3.99E-44	93.20595	up
MORF_ESP	0.710581	-0.06454	16.13208	3.78E-34	4.97E-33	66.80545	up
GOBP_COI	0.710384	0.004059	14.94407	3.92E-31	4.33E-30	59.85925	up
GOBP_POS	0.710259	0.035501	25.57618	1.49E-55	1.20E-53	116.1062	up
HP_VENTR	-0.71005	-0.11424	-16.0909	4.80E-34	6.28E-33	66.5668	down
HP_METAF	0.70985	-0.03197	19.09784	2.19E-41	4.74E-40	83.47608	up
GOMF_SO	-0.70977	-0.03659	-19.5608	1.79E-42	4.20E-41	85.98277	down
GOMF_IRC	-0.70976	0.009061	-19.1745	1.45E-41	3.18E-40	83.89283	down
PID_ATM_I	0.709759	0.002289	21.6118	3.75E-47	1.30E-45	96.76219	up
GOMF_FM	-0.70972	-0.13142	-25.1541	1.07E-54	7.73E-53	114.1376	down
KERLEY_RE	-0.70959	-0.00084	-12.5707	5.81E-25	4.31E-24	45.66078	down
GOBP_IND	-0.70926	-0.01918	-28.7821	9.20E-62	1.29E-59	130.3951	down
GOCC_LAT	0.709071	0.048299	17.31928	4.24E-37	6.77E-36	73.60149	up
GOBP_ALD	-0.70853	-0.10643	-15.9449	1.12E-33	1.44E-32	65.72042	down
GOBP_POS	-0.70843	-0.00972	-14.1575	4.18E-29	4.06E-28	55.19217	down
REACTOM	-0.70837	0.069985	-16.8046	7.90E-36	1.16E-34	70.67439	down
WP_AMINO	-0.70825	-0.08563	-21.3366	1.54E-46	5.05E-45	95.34659	down
GOBP_HIST	0.707975	-0.11	12.84301	1.12E-25	8.75E-25	47.30416	up
GOMF_FAI	-0.70731	-0.09991	-23.4299	4.15E-51	2.15E-49	105.8735	down
REACTOM	-0.7071	-0.1288	-13.1206	2.10E-26	1.73E-25	48.97712	down
GOBP_REP	0.706899	-0.00393	22.14944	2.43E-48	9.52E-47	99.49972	up
GOBP_B_C	-0.70689	0.032107	-14.1715	3.84E-29	3.74E-28	55.27566	down
GOBP_NEC	-0.70686	0.158089	-10.8308	2.17E-20	1.17E-19	35.15764	down
GOMF_RET	-0.70674	0.034235	-17.1225	1.29E-36	2.00E-35	72.48596	down
GOBP_POS	-0.70641	0.0083	-13.8351	2.87E-28	2.65E-27	53.26633	down

HP_ABNOI	-0.70619	-0.07456	-25.1789	9.52E-55	6.96E-53	114.2538	down
REACTOM	0.706161	-0.00414	18.58987	3.53E-40	6.97E-39	80.69501	up
GOBP_POS	0.705851	0.03378	13.99462	1.11E-28	1.05E-27	54.22012	up
GOBP_CEN	0.705586	-0.0696	15.4829	1.65E-32	1.98E-31	63.02638	up
HP_DECRE	-0.70536	-0.11937	-17.0634	1.81E-36	2.77E-35	72.14974	down
GOBP_CEL	-0.70513	0.090974	-15.4095	2.54E-32	3.00E-31	62.59638	down
GOMF_CR(	0.705063	-0.09999	13.70739	6.17E-28	5.57E-27	52.50169	up
REACTOM	-0.70421	0.148203	-11.4449	5.30E-22	3.22E-21	38.8586	down
GOBP_CHI	0.703993	0.005465	20.75416	3.18E-45	9.39E-44	92.31954	up
GOBP_CRD	0.703855	0.042275	11.45208	5.07E-22	3.08E-21	38.90196	up
WP_EXRN/	0.703749	0.080463	14.01562	9.75E-29	9.27E-28	54.34555	up
GOBP_MEI	-0.70328	0.056248	-13.6448	8.98E-28	8.03E-27	52.12679	down
WIEMANN	-0.70317	0.06832	-12.9367	6.37E-26	5.05E-25	47.86936	down
GOBP_HIS	0.70315	0.01696	15.82763	2.21E-33	2.79E-32	65.03835	up
GOMF_HIS	0.70315	0.01696	15.82763	2.21E-33	2.79E-32	65.03835	up
GOBP_REG	0.703035	0.050203	11.1358	3.44E-21	1.97E-20	36.99392	up
GOCC_FAI	0.702941	0.001996	15.32086	4.27E-32	4.98E-31	62.07668	up
GOMF_AP_	-0.70294	0.060694	-13.1615	1.64E-26	1.36E-25	49.2229	down
KEGG_ARG	-0.7029	-0.06731	-22.1198	2.82E-48	1.09E-46	99.34983	down
HP_NEON/	0.702829	-0.01007	12.36511	2.01E-24	1.45E-23	44.41913	up
GOBP_POS	-0.70255	0.144748	-13.0406	3.40E-26	2.76E-25	48.49528	down
HP_CYSTA	-0.7024	-0.12175	-12.314	2.74E-24	1.95E-23	44.11056	down
GOBP_TRA	0.702272	-0.10014	12.9695	5.22E-26	4.18E-25	48.06673	up
GSE21670_	0.702066	0.000863	24.88925	3.72E-54	2.54E-52	112.8914	up
GOMF_BU	-0.7017	-0.16721	-17.495	1.57E-37	2.58E-36	74.59383	down
GOBP_DET	-0.7017	-0.01481	-11.3591	8.90E-22	5.31E-21	38.34084	down
HP_PRELIN	-0.70164	-0.10453	-16.6593	1.81E-35	2.60E-34	69.84249	down
MODULE_:	0.701526	-0.01446	22.71397	1.43E-49	6.31E-48	102.335	up
GOMF_PYI	0.701457	-0.10215	13.27218	8.43E-27	7.10E-26	49.88901	up
HP_CHROI	-0.70146	0.048475	-13.6975	6.54E-28	5.90E-27	52.44259	down
HU_GENO	0.70138	-0.04478	19.91438	2.69E-43	6.75E-42	87.87903	up
GOBP_CILI	-0.7011	0.056104	-13.7746	4.12E-28	3.77E-27	52.90408	down
LY_AGING_	0.701016	-0.02664	18.19448	3.14E-39	5.82E-38	78.50839	up
GOBP_ATT	0.700695	-0.0329	19.40916	4.05E-42	9.32E-41	85.16466	up
GOBP_REG	-0.70065	-0.075	-23.7091	1.06E-51	5.84E-50	107.2362	down
GOBP_REG	0.700461	-0.03002	15.24861	6.53E-32	7.54E-31	61.65249	up
GOBP_NEC	-0.7004	0.14048	-12.5125	8.26E-25	6.05E-24	45.30977	down
KEGG_PYR	-0.70034	-0.0845	-22.2516	1.45E-48	5.81E-47	100.016	down
HP_COMA	-0.69967	-0.05201	-26.0387	1.77E-56	1.51E-54	118.2396	down
MODULE_:	0.699307	-0.03655	28.91874	5.13E-62	7.42E-60	130.9791	up
HP_HYPER	-0.69921	-0.06476	-28.5493	2.50E-61	3.30E-59	129.3957	down
HP_ABNOI	-0.69904	-0.06167	-23.2694	9.12E-51	4.54E-49	105.0859	down
GSE39556_	0.699023	-0.02972	17.88991	1.71E-38	3.01E-37	76.81113	up
GOBP_FOF	0.69883	-0.0471	19.88988	3.07E-43	7.66E-42	87.74813	up
UZONYI_R	-0.69877	0.09175	-15.7545	3.38E-33	4.22E-32	64.61275	down
NR4A2_TA	0.698628	0.04567	13.49649	2.19E-27	1.92E-26	51.23682	up
MODULE_:	0.698531	-0.02617	19.59407	1.50E-42	3.54E-41	86.16181	up
MISSIAGLI	0.698424	-0.04234	16.41462	7.40E-35	1.02E-33	68.43677	up
MODULE_:	-0.69833	-0.03073	-25.1145	1.29E-54	9.19E-53	113.9519	down
GOMF_C_X	-0.69822	0.100639	-11.5381	3.01E-22	1.86E-21	39.42161	down
HP_LARYN	0.698086	0.036109	14.24869	2.43E-29	2.39E-28	55.73569	up
MODULE_:	0.697822	0.008424	22.47317	4.76E-49	1.99E-47	101.1305	up
GOBP_TRI(	-0.69762	-0.08513	-21.0817	5.77E-46	1.82E-44	94.02709	down
BURTON_/	0.697564	-0.02748	17.88621	1.75E-38	3.07E-37	76.79041	up
WP_7OXO	-0.69751	-0.15286	-17.8745	1.87E-38	3.28E-37	76.72486	down
REACTOM	-0.69751	0.007327	-19.1319	1.82E-41	3.98E-40	83.66142	down
HP_CHON	-0.69743	0.045689	-15.7381	3.72E-33	4.63E-32	64.51703	down
HP_ACUTE	-0.69704	-0.11029	-15.1681	1.05E-31	1.19E-30	61.17919	down

BENPORAT	0.696986	-0.00358	18.59957	3.35E-40	6.63E-39	80.74842	up
KEGG_PPA	-0.69698	-0.06496	-24.8674	4.12E-54	2.80E-52	112.7882	down
HP_BIRTH_	0.696964	0.00811	17.69492	5.10E-38	8.71E-37	75.71867	up
GOBP_FEM	0.696919	-0.07289	15.66749	5.62E-33	6.92E-32	64.10516	up
HP_MICRO	-0.6967	-0.05866	-19.1612	1.55E-41	3.41E-40	83.82069	down
GOBP_URA	-0.69648	-0.02721	-16.8227	7.12E-36	1.05E-34	70.77806	down
GOMF_CO	-0.69636	-0.02259	-12.6112	4.55E-25	3.40E-24	45.90552	down
FURUKAW	0.696308	-0.03138	20.32496	3.04E-44	8.28E-43	90.06119	up
REACTOM	0.696271	-0.00699	24.31927	5.59E-53	3.46E-51	110.1812	up
GOBP_MIT	0.696206	-0.01852	28.0744	1.96E-60	2.40E-58	127.3389	up
GOBP_POS	-0.69614	0.111177	-12.8719	9.41E-26	7.39E-25	47.47863	down
GOMF_STE	-0.69596	-0.10671	-17.5658	1.05E-37	1.75E-36	74.993	down
PUJANA_B	0.69595	0.014176	23.19731	1.30E-50	6.40E-49	104.7309	up
HP_ABNOI	-0.69505	-0.0382	-18.1835	3.34E-39	6.16E-38	78.44732	down
HP_ABNOI	-0.69487	-0.06122	-28.547	2.53E-61	3.32E-59	129.3858	down
REACTOM	-0.69475	-0.09432	-20.6974	4.28E-45	1.25E-43	92.02205	down
GOMF_SNI	0.694408	-0.13518	10.70039	4.76E-20	2.52E-19	34.37414	up
REACTOM	0.694175	-0.01641	17.81557	2.60E-38	4.52E-37	76.39515	up
GOMF_PEI	-0.69375	-0.18182	-11.3776	7.96E-22	4.77E-21	38.45253	down
GOBP_NEC	-0.69365	0.083935	-16.7899	8.59E-36	1.26E-34	70.59034	down
HP_BRANC	0.693179	-0.08648	13.18452	1.43E-26	1.19E-25	49.36164	up
GOBP_REG	0.693063	0.083592	16.66444	1.76E-35	2.52E-34	69.87204	up
STEIN_ESR	0.693015	-0.00229	17.90169	1.60E-38	2.83E-37	76.87695	up
GOCC_BRC	0.692673	-0.08838	14.11522	5.38E-29	5.19E-28	54.94004	up
GROSS_HII	-0.69259	0.118353	-13.9073	1.86E-28	1.74E-27	53.69833	down
HP_TALL_C	0.692422	0.042285	18.36857	1.20E-39	2.30E-38	79.47353	up
VANTVEER	0.692235	-0.01024	20.80233	2.47E-45	7.39E-44	92.57154	up
DESCARTE	-0.69202	-0.07286	-19.9868	1.83E-43	4.66E-42	88.26554	down
GOMF_WA	-0.69182	0.011226	-22.4242	6.08E-49	2.51E-47	100.8846	down
GOBP_MEI	0.69167	-0.04672	20.47661	1.36E-44	3.82E-43	90.86179	up
GOBP_MID	-0.69161	-0.00352	-18.6865	2.08E-40	4.15E-39	81.22631	down
GOBP_GLY	-0.69159	0.047218	-15.7497	3.48E-33	4.34E-32	64.58454	down
GOMF_GLY	-0.69159	0.047218	-15.7497	3.48E-33	4.34E-32	64.58454	down
NADERI_BI	0.691552	-0.04183	19.6998	8.48E-43	2.04E-41	86.73006	up
GOBP_POS	0.691522	-0.01052	14.6611	2.09E-30	2.21E-29	58.18585	up
AMIT_DEL	-0.69148	0.119617	-14.0218	9.39E-29	8.95E-28	54.38273	down
CAO_BLOC	-0.69131	-0.11616	-9.61648	3.12E-17	1.35E-16	27.91668	down
NAKAYAM	-0.69121	-0.04757	-27.1763	1.03E-58	1.06E-56	123.382	down
HP_ABNOI	-0.69116	-0.01676	-13.718	5.79E-28	5.23E-27	52.56546	down
GOBP_FAT	-0.69091	-0.07438	-33.1027	2.12E-69	6.71E-67	147.9469	down
HP_PROXII	-0.69084	-0.14768	-16.5083	4.32E-35	6.01E-34	68.97555	down
GOBP_CHY	-0.69044	-0.04915	-12.1378	7.97E-24	5.51E-23	43.0457	down
GOBP_BILE	-0.69039	-0.06912	-27.4658	2.84E-59	3.12E-57	124.6673	down
GOBP_DRU	-0.69038	-0.06631	-15.5776	9.50E-33	1.15E-31	63.58049	down
BOQUEST_	-0.69019	0.010205	-22.2206	1.70E-48	6.75E-47	99.85916	down
GOBP_CHI	0.689998	-0.04781	22.04224	4.18E-48	1.60E-46	98.9568	up
REACTOM	0.689921	-0.04185	16.65049	1.91E-35	2.73E-34	69.79208	up
GOBP_NEC	-0.68988	-0.08265	-11.749	8.40E-23	5.38E-22	40.69573	down
GOBP_RES	0.689601	0.012488	13.416	3.55E-27	3.07E-26	50.75343	up
REACTOM	-0.68936	-0.09173	-29.6953	1.92E-63	3.22E-61	134.2603	down
CGGTGTG_	-0.6892	-0.0604	-12.9631	5.43E-26	4.34E-25	48.02838	down
HP_ABNOI	0.689157	-0.06459	13.75227	4.71E-28	4.29E-27	52.77052	up
PETROVA_	0.688731	0.01773	21.39231	1.16E-46	3.85E-45	95.63407	up
KEGG_ASC	-0.68873	-0.07245	-14.3482	1.34E-29	1.34E-28	56.32812	down
GOMF_H3_	0.688652	-0.02637	15.81628	2.36E-33	2.97E-32	64.9723	up
HP_ABNOI	-0.68856	-0.1115	-19.0861	2.34E-41	5.04E-40	83.41235	down
SERVITJA_I	-0.68838	-0.03022	-30.5569	5.38E-65	1.03E-62	137.8284	down
BIOCARTA	0.688334	0.026338	15.16457	1.07E-31	1.22E-30	61.15843	up

GOMF_U4_	0.688237	0.016505	12.20156	5.42E-24	3.78E-23	43.43084	up
GOBP_CAT	-0.68784	0.043761	-22.5152	3.85E-49	1.63E-47	101.3412	down
REACTOM	-0.68784	-0.07734	-18.4443	7.88E-40	1.53E-38	79.89219	down
MIR11400	0.687807	-0.03559	14.39979	9.87E-30	9.93E-29	56.6349	up
WP_FLUOI	-0.68761	-0.11443	-13.1166	2.15E-26	1.77E-25	48.95272	down
MODULE_	-0.68746	-0.12241	-16.8443	6.30E-36	9.32E-35	70.90109	down
HOSHIDA_	-0.68703	-0.07636	-25.9903	2.21E-56	1.87E-54	118.0174	down
chr11p12	-0.68691	0.127577	-12.8007	1.45E-25	1.12E-24	47.04929	down
HP_ARTHF	0.68682	-0.00947	13.03517	3.52E-26	2.85E-25	48.4624	up
GOBP_REG	0.686622	-0.03933	19.8011	4.93E-43	1.21E-41	87.2732	up
GOBP_NEC	-0.68654	0.115885	-13.1788	1.48E-26	1.23E-25	49.3272	down
TFDP2_TAI	0.686343	-0.19993	11.27775	1.46E-21	8.54E-21	37.84982	up
GOBP_ATT	0.68609	-0.05101	15.68041	5.21E-33	6.44E-32	64.18053	up
REACTOM	-0.68603	-0.04946	-13.6793	7.30E-28	6.56E-27	52.33352	down
FISCHER_D	0.685783	-0.01353	19.73618	6.98E-43	1.70E-41	86.92528	up
HP_PROLC	-0.6852	-0.04002	-18.1027	5.23E-39	9.54E-38	77.998	down
SHETH_LIV	-0.68514	-0.05977	-37.0352	1.03E-75	1.10E-72	162.4346	down
HP_ACHOI	-0.68508	-0.03757	-15.3991	2.70E-32	3.19E-31	62.53551	down
WP_SARSC	-0.68507	0.079351	-18.1944	3.14E-39	5.82E-38	78.50777	down
GOBP_TEL	0.68503	-0.07675	18.29084	1.84E-39	3.47E-38	79.04305	up
GOBP_POS	-0.68489	-0.02513	-22.6146	2.34E-49	1.01E-47	101.8388	down
GOBP_URA	-0.68483	-0.12488	-12.9828	4.82E-26	3.87E-25	48.14689	down
GOBP_CYT	-0.68474	-0.02673	-20.96	1.09E-45	3.33E-44	93.39407	down
HP_VASCU	-0.68474	0.048469	-16.3083	1.37E-34	1.85E-33	67.82382	down
MODULE_	-0.68472	-0.14083	-18.6491	2.55E-40	5.08E-39	81.0209	down
ZERBINI_RI	-0.68455	0.010611	-14.2195	2.89E-29	2.83E-28	55.5619	down
GOBP_LON	-0.68433	-0.0709	-30.9804	9.54E-66	1.96E-63	139.5549	down
CHUANG_	0.683652	0.026037	16.75338	1.06E-35	1.54E-34	70.3814	up
WP_EICOS	-0.6831	-0.04188	-25.8724	3.79E-56	3.18E-54	117.4753	down
HP_METAE	-0.68291	-0.10196	-23.7665	8.04E-52	4.49E-50	107.515	down
HP_ABNOI	0.682355	0.005184	20.94725	1.16E-45	3.55E-44	93.32787	up
REACTOM	0.682331	-0.01098	16.47645	5.18E-35	7.19E-34	68.79263	up
GOMF_OX	0.682308	-0.10298	13.6795	7.29E-28	6.56E-27	52.33451	up
GOBP_POS	-0.68212	0.087357	-13.3559	5.09E-27	4.36E-26	50.39246	down
HSD17B8_	0.682045	-0.02756	22.15096	2.41E-48	9.46E-47	99.50741	up
GOBP_STE	-0.68184	-0.05126	-14.9024	5.02E-31	5.51E-30	59.61321	down
WP_MED_	0.681758	0.006187	13.0277	3.68E-26	2.97E-25	48.41739	up
GOBP_QUI	-0.68144	-0.16935	-18.3492	1.33E-39	2.55E-38	79.3664	down
GOMF_STE	-0.68121	-0.14394	-18.9414	5.14E-41	1.09E-39	82.62286	down
MINGUEZ_	0.680541	-0.08468	15.16595	1.06E-31	1.21E-30	61.16655	up
REACTOM	-0.68042	-0.00856	-25.5703	1.53E-55	1.23E-53	116.0788	down
HP_NARRC	0.679976	-0.03763	19.08692	2.33E-41	5.02E-40	83.41663	up
REACTOM	-0.67961	0.018861	-17.7955	2.90E-38	5.03E-37	76.28292	down
BIOCARTA	0.679592	-0.03741	20.83596	2.07E-45	6.23E-44	92.74728	up
HP_ABNOI	-0.67955	-0.17096	-14.0557	7.68E-29	7.34E-28	54.58456	down
LE_SKI_TAI	-0.67946	0.131997	-12.8094	1.37E-25	1.07E-24	47.10155	down
REACTOM	-0.67942	-0.07531	-24.9287	3.09E-54	2.14E-52	113.0777	down
GOBP_GLY	-0.67931	-0.18185	-12.1293	8.40E-24	5.78E-23	42.9943	down
GOCC_UN	-0.67926	-0.05727	-12.0488	1.37E-23	9.27E-23	42.50729	down
FAN_EMBF	-0.67917	0.207374	-11.1495	3.16E-21	1.82E-20	37.07646	down
MODULE_	0.679111	-0.05094	17.58746	9.33E-38	1.56E-36	75.11469	up
GOBP_INT	0.678472	-0.06816	17.81459	2.61E-38	4.54E-37	76.38966	up
GOMF_OX	-0.67824	-0.10064	-27.339	4.99E-59	5.35E-57	124.1051	down
HP_BREAS	0.678075	-0.0642	21.95295	6.58E-48	2.46E-46	98.50345	up
ZNF416_TA	0.67805	-0.004	10.03404	2.60E-18	1.22E-17	30.39034	up
HP_EYELID	0.677328	-0.06117	13.27921	8.08E-27	6.82E-26	49.93131	up
GOBP_MIT	0.677278	-0.03453	21.6247	3.51E-47	1.22E-45	96.82829	up
REACTOM	-0.67725	-0.07604	-14.8732	5.96E-31	6.51E-30	59.44078	down

HP_PRIMA	0.677249	0.003982	15.27236	5.68E-32	6.58E-31	61.79198	up
GOMF_OX	-0.6772	0.045186	-15.9231	1.27E-33	1.62E-32	65.59355	down
HP_ABNOI	-0.6772	-0.13671	-14.4182	8.85E-30	8.95E-29	56.74412	down
GOMF_CEI	0.676971	-0.09007	13.55632	1.53E-27	1.35E-26	51.59586	up
GOCC_GPI	0.676926	-0.14526	12.38959	1.74E-24	1.25E-23	44.56702	up
LANDIS_EI	-0.67687	-0.07777	-23.7826	7.44E-52	4.16E-50	107.5934	down
GOCC_PAI	0.676715	0.099961	10.57216	1.03E-19	5.32E-19	33.60476	up
REACTOM	-0.67657	-0.03196	-21.5254	5.84E-47	1.99E-45	96.31893	down
GOBP_RIB(	-0.67645	0.002075	-16.378	9.13E-35	1.25E-33	68.22569	down
GOBP_PRII	-0.6764	-0.06859	-30.5913	4.67E-65	8.97E-63	137.9696	down
GOBP_VER	-0.67628	-0.06104	-14.4455	7.52E-30	7.65E-29	56.90655	down
GOBP_SCA	0.67626	-0.04045	9.515445	5.67E-17	2.41E-16	27.32147	up
HP_BICARI	-0.67621	-0.11314	-12.8459	1.10E-25	8.61E-25	47.32146	down
GSE30962_	0.676071	-0.03739	19.30231	7.22E-42	1.63E-40	84.58648	up
GOBP_VER	-0.67563	-0.08035	-20.1756	6.70E-44	1.77E-42	89.26983	down
WU_APOP	0.675456	-0.02342	17.44047	2.14E-37	3.48E-36	74.28635	up
LE_EGR2_T	0.67519	0.032182	22.35384	8.66E-49	3.53E-47	100.5309	up
GOBP_URE	-0.67515	0.032545	-15.4837	1.64E-32	1.97E-31	63.03125	down
chr13q22	-0.67494	0.130739	-14.4946	5.62E-30	5.74E-29	57.19834	down
GOBP_NE(	-0.67482	0.04372	-21.0128	8.25E-46	2.56E-44	93.66934	down
REACTOM	0.674712	0.005673	26.08342	1.44E-56	1.24E-54	118.4444	up
LEE_LIVER_	-0.67469	-0.08758	-25.6441	1.09E-55	8.85E-54	116.4212	down
REACTOM	0.674456	-0.00564	19.95382	2.18E-43	5.52E-42	88.08955	up
MODULE_	0.674433	0.037691	12.60374	4.76E-25	3.55E-24	45.86041	up
GOCC_KIN	0.674427	-0.0316	21.07181	6.07E-46	1.91E-44	93.97587	up
GOBP_REG	-0.67441	0.098437	-13.2856	7.77E-27	6.57E-26	49.96971	down
HP_ENDO(	0.674248	-0.21089	12.55691	6.31E-25	4.66E-24	45.57768	up
GOBP_NE(	-0.67417	-0.03291	-19.8546	3.70E-43	9.16E-42	87.55976	down
GOBP_REG	-0.67411	-0.01592	-9.42646	9.59E-17	4.02E-16	26.79843	down
MODULE_	-0.67339	-0.04216	-31.9784	1.73E-67	4.40E-65	143.5545	down
GOBP_POS	0.673272	-0.10088	11.77999	6.96E-23	4.49E-22	40.88285	up
QIU_PBMC	-0.6732	0.062093	-18.093	5.52E-39	1.00E-37	77.94419	down
GOMF_CA	-0.6731	-0.0819	-24.4691	2.73E-53	1.76E-51	110.8973	down
WANG_CL	-0.67303	-0.11869	-18.7104	1.82E-40	3.66E-39	81.35788	down
VAN_DEN_	0.672954	0.011131	18.23268	2.54E-39	4.74E-38	78.72047	up
HP_LIMBA	-0.67272	0.169974	-13.0586	3.05E-26	2.48E-25	48.6033	down
GOBP_ME	-0.6727	-0.0707	-17.6035	8.53E-38	1.43E-36	75.20502	down
GOBP_CH(	0.672596	-0.049	12.83175	1.20E-25	9.34E-25	47.23631	up
HP_ABNOI	-0.67259	-0.04436	-17.79	3.00E-38	5.19E-37	76.25175	down
GOBP_DIA	-0.67253	-0.09966	-15.9457	1.11E-33	1.43E-32	65.72503	down
WP_IRON_	-0.67236	-0.03909	-16.8675	5.52E-36	8.21E-35	71.03356	down
HP_CLUBB	0.672338	-0.02189	17.78455	3.09E-38	5.35E-37	76.22138	up
REACTOM	0.672141	-0.04049	18.3134	1.62E-39	3.08E-38	79.16806	up
GOBP_ALD	-0.67211	-0.05848	-18.1192	4.77E-39	8.71E-38	78.09	down
HP_PROXII	-0.67208	-0.09361	-18.2219	2.70E-39	5.02E-38	78.66091	down
GOCC_TH(	0.672028	-0.12877	11.30457	1.24E-21	7.30E-21	38.01166	up
GOBP_MIT	0.67192	-0.00702	25.53697	1.79E-55	1.42E-53	115.9242	up
GOBP_DN/	0.671786	-0.03009	20.65794	5.26E-45	1.53E-43	91.81525	up
GOBP_CEL	-0.67164	-0.08522	-24.9127	3.33E-54	2.30E-52	113.0021	down
GOBP_REG	-0.67146	-0.05275	-23.8187	6.24E-52	3.52E-50	107.7685	down
GNF2_CD1	-0.67124	0.107896	-12.117	9.05E-24	6.22E-23	42.91986	down
REACTOM	-0.67123	0.009104	-13.8634	2.42E-28	2.25E-27	53.4354	down
GOBP_HIG	-0.67121	-0.10784	-12.0943	1.04E-23	7.10E-23	42.78271	down
CHEN_ETV	0.671069	0.024144	20.29619	3.54E-44	9.53E-43	89.90899	up
GOMF_TO	-0.67079	0.037746	-15.3809	3.00E-32	3.53E-31	62.42893	down
GOMF_CO	-0.67076	-0.00656	-16.8902	4.85E-36	7.23E-35	71.16314	down
GOBP_REG	0.670715	-0.1343	12.18676	5.93E-24	4.13E-23	43.3414	up
COULOUA	-0.67069	-0.06678	-27.6448	1.29E-59	1.44E-57	125.4572	down

GOBP_TEL(	0.670258	-0.08771	13.93089	1.62E-28	1.52E-27	53.83929	up
WP_IRINO	-0.67008	-0.07243	-15.8347	2.12E-33	2.68E-32	65.07949	down
MODULE_3	-0.66969	-0.06726	-19.6438	1.15E-42	2.74E-41	86.4292	down
GOBP_NEC	0.669649	0.065811	17.58472	9.48E-38	1.59E-36	75.09925	up
KEGG_ALA	-0.6696	-0.02569	-20.6674	5.01E-45	1.45E-43	91.86489	down
GOMF_MH	-0.66953	0.060481	-10.4742	1.86E-19	9.40E-19	33.01782	down
GOBP_CEL	-0.66936	-0.08674	-17.8054	2.75E-38	4.78E-37	76.33797	down
WP_GLUC	-0.66914	-0.03279	-14.1044	5.74E-29	5.52E-28	54.87566	down
HP_RECUR	-0.66907	0.085975	-17.66	6.21E-38	1.06E-36	75.52243	down
GOBP_POS	0.668786	-0.04455	16.33912	1.14E-34	1.56E-33	68.00165	up
GOBP_EPIT	-0.66854	0.134439	-14.2053	3.14E-29	3.07E-28	55.47694	down
HP_ABNOI	-0.66834	0.013634	-14.7174	1.50E-30	1.59E-29	58.5191	down
GOBP_CEN	0.668165	0.018772	16.81233	7.56E-36	1.11E-34	70.71853	up
HP_RENAL	-0.66804	-0.11482	-14.4071	9.45E-30	9.52E-29	56.67807	down
HALLMARI	-0.668	-0.04356	-29.1488	1.92E-62	2.90E-60	131.9579	down
WP_CALOI	-0.66769	-0.0113	-15.1914	9.14E-32	1.04E-30	61.31616	down
GOBP_NEC	-0.66764	-0.06642	-19.2812	8.10E-42	1.82E-40	84.47218	down
GOCC_ESC	0.667639	0.054989	19.31183	6.86E-42	1.55E-40	84.63801	up
MODULE_1	-0.66758	-0.12726	-15.1226	1.37E-31	1.55E-30	60.91159	down
MODULE_1	0.667519	0.009438	23.58023	1.99E-51	1.07E-49	106.6084	up
GSE37532_	0.66736	-0.02935	19.97473	1.95E-43	4.96E-42	88.2011	up
PID_BARD1	0.666629	0.024658	17.52671	1.31E-37	2.17E-36	74.77267	up
GOBP_NCI	0.666225	0.01133	15.69145	4.89E-33	6.05E-32	64.24492	up
GOBP_REG	-0.66617	0.04922	-14.2061	3.13E-29	3.06E-28	55.48197	down
LY_AGING_	0.666161	0.006956	16.48358	4.97E-35	6.91E-34	68.83361	up
GOMF_STE	-0.666	-0.02001	-19.493	2.58E-42	6.01E-41	85.61731	down
HP_SEGME	-0.6659	-0.01329	-15.6526	6.13E-33	7.54E-32	64.01835	down
GOCC_CO	0.665801	-0.0151	16.90109	4.55E-36	6.81E-35	71.22541	up
GOBP_POS	0.665758	-0.06737	15.45805	1.91E-32	2.28E-31	62.8809	up
FERREIRA_	0.665734	-0.00226	20.32773	2.99E-44	8.17E-43	90.07587	up
GOBP_RES	-0.66571	-0.16166	-16.5551	3.30E-35	4.63E-34	69.24467	down
HP_SIMPLI	0.665705	-0.0131	24.68137	9.95E-54	6.60E-52	111.9075	up
GOMF_RN	0.665619	-0.03869	16.15742	3.26E-34	4.31E-33	66.95209	up
KEGG_CITI	-0.66557	-0.10878	-14.4134	9.10E-30	9.18E-29	56.71559	down
PIONTEK_I	-0.66554	-0.08738	-22.8078	8.94E-50	4.09E-48	102.8024	down
GOCC_MIC	0.665515	-0.02374	12.36357	2.03E-24	1.46E-23	44.40988	up
REACTOM	-0.66522	-0.09727	-10.6629	5.96E-20	3.13E-19	34.14886	down
LI_PBMC_N	-0.6652	0.04685	-19.2732	8.46E-42	1.89E-40	84.42883	down
GOMF_DN	0.665155	-0.02478	14.2178	2.92E-29	2.85E-28	55.55167	up
REACTOM	0.665002	-0.00606	20.3476	2.70E-44	7.38E-43	90.18093	up
HP_STERO	0.664543	-0.05889	14.44145	7.70E-30	7.83E-29	56.88251	up
GOMF_PR(	-0.66438	-0.02853	-9.16683	4.42E-16	1.76E-15	25.27927	down
GOBP_NEC	-0.66435	-0.03333	-16.9361	3.73E-36	5.61E-35	71.42511	down
HP_ENTER	-0.66433	0.081714	-13.2334	1.06E-26	8.94E-26	49.65557	down
LEE_LIVER_	0.664172	0.051483	22.19591	1.92E-48	7.62E-47	99.73461	up
GOBP_REG	0.664074	0.006034	19.33535	6.04E-42	1.37E-40	84.76538	up
MIR4681	0.663886	-0.01695	13.26948	8.57E-27	7.22E-26	49.87276	up
GOBP_GRA	-0.66378	-0.01881	-11.6727	1.33E-22	8.42E-22	40.23434	down
GOBP_REG	0.663708	-0.0295	14.85857	6.50E-31	7.08E-30	59.35434	up
GOBP_PYR	-0.66362	-0.06377	-12.9642	5.39E-26	4.31E-25	48.0347	down
GOBP_REG	-0.66336	0.00577	-12.1827	6.08E-24	4.23E-23	43.31684	down
HP_URETH	0.663162	-0.05088	16.75797	1.03E-35	1.50E-34	70.40768	up
REACTOM	0.663107	-0.03938	19.7185	7.67E-43	1.86E-41	86.83044	up
HP_LOW_A	-0.66275	-0.01586	-11.8712	4.01E-23	2.63E-22	41.43427	down
BIOCARTA	-0.66265	0.121295	-14.3722	1.16E-29	1.17E-28	56.47098	down
GOBP_DNA	0.662317	0.018763	17.00706	2.49E-36	3.79E-35	71.82937	up
HP_CERVIC	0.66221	0.083921	11.65234	1.51E-22	9.48E-22	40.1115	up
GOMF_DN	0.662039	0.019799	21.55972	4.90E-47	1.68E-45	96.49505	up

GOMF_N_/	-0.66202	0.077192	-12.7433	2.05E-25	1.57E-24	46.70275	down
GOBP_NAI	-0.66198	-0.07232	-12.6383	3.86E-25	2.90E-24	46.06908	down
GOMF_NA	-0.66181	-0.11051	-19.1837	1.37E-41	3.03E-40	83.94304	down
GOBP_HIS	-0.66174	0.045752	-11.9309	2.79E-23	1.85E-22	41.79521	down
GOBP_MIT	-0.6615	-0.2056	-12.9897	4.62E-26	3.72E-25	48.18837	down
GOMF_RN	-0.66146	0.046541	-9.31393	1.86E-16	7.63E-16	26.13871	down
GOMF_THI	-0.66115	-0.1611	-10.4862	1.73E-19	8.76E-19	33.08963	down
HP_NONK	-0.66109	-0.06527	-17.2297	7.03E-37	1.10E-35	73.09438	down
REACTOM	0.660993	0.004551	15.62921	7.03E-33	8.62E-32	63.88171	up
GOMF_MH	-0.66092	0.096166	-10.1934	1.00E-18	4.82E-18	31.33929	down
SANCHEZ_	-0.66079	0.025046	-17.7289	4.22E-38	7.22E-37	75.9095	down
BURTON_/	-0.66022	-0.11399	-23.5929	1.87E-51	1.01E-49	106.6703	down
HP_RENAL	0.660173	-0.06666	15.44422	2.07E-32	2.46E-31	62.79991	up
GOBP_POS	-0.65999	0.007837	-14.0321	8.84E-29	8.43E-28	54.44366	down
LEE_LIVER_	-0.65976	-0.06059	-27.2071	8.98E-59	9.43E-57	123.5188	down
REACTOM	0.659713	-0.02606	16.73976	1.14E-35	1.66E-34	70.30345	up
GOBP_BRU	-0.65955	0.006397	-12.1788	6.22E-24	4.33E-23	43.29304	down
GOBP_REG	-0.65953	-0.03645	-24.3006	6.11E-53	3.74E-51	110.0917	down
GOBP_NEC	0.659395	-0.12879	11.23032	1.94E-21	1.13E-20	37.56376	up
REACTOM	0.659195	0.102746	12.65727	3.44E-25	2.60E-24	46.18353	up
REACTOM	-0.65913	-0.10139	-19.7394	6.86E-43	1.67E-41	86.94248	down
GOBP_RET	0.659115	-0.03396	13.42782	3.31E-27	2.87E-26	50.82444	up
GOCC_U1_	0.659105	-0.08898	12.55911	6.23E-25	4.60E-24	45.59092	up
HP_THIN_(	0.658957	-0.15741	12.71194	2.47E-25	1.88E-24	46.51351	up
GOMF_O_I	-0.65893	-0.03251	-13.1239	2.06E-26	1.70E-25	48.99658	down
MIR3683	-0.65878	0.141548	-13.6314	9.73E-28	8.69E-27	52.04593	down
HP_ADREN	-0.65863	-0.15825	-14.0035	1.05E-28	9.95E-28	54.27302	down
GOCC_HIS	0.658262	-0.02421	11.39065	7.35E-22	4.42E-21	38.53113	up
GOBP_REG	-0.65824	0.142822	-11.4387	5.50E-22	3.33E-21	38.82101	down
GOMF_5_3	0.658149	-0.09086	13.17891	1.48E-26	1.23E-25	49.3279	up
CHIANG_L	-0.6581	-0.11434	-17.4587	1.93E-37	3.15E-36	74.38916	down
GOBP_DN/	0.657747	-0.0087	19.9636	2.07E-43	5.25E-42	88.14177	up
GOCC_CHI	0.657715	-0.01304	22.11524	2.89E-48	1.12E-46	99.32664	up
GOBP_ACE	-0.65728	-0.0799	-17.9265	1.40E-38	2.47E-37	77.01556	down
GOBP_ARA	-0.65711	-0.04951	-27.5571	1.90E-59	2.11E-57	125.0707	down
GOMF_VEI	-0.65708	-0.06923	-17.3981	2.71E-37	4.38E-36	74.04687	down
GOMF_OX	-0.65699	0.020104	-19.0316	3.14E-41	6.70E-40	83.11504	down
GOBP_UDI	-0.65693	0.016594	-11.7722	7.30E-23	4.70E-22	40.83547	down
GOMF_UD	-0.65693	0.016594	-11.7722	7.30E-23	4.70E-22	40.83547	down
MCBRYAN	-0.65657	0.000966	-25.4618	2.54E-55	1.99E-53	115.5748	down
HP_ARACH	-0.65638	-0.05017	-18.5951	3.43E-40	6.78E-39	80.72368	down
GOBP_EST	-0.65588	0.120474	-13.1992	1.31E-26	1.09E-25	49.45007	down
GOCC_PEF	0.655819	0.076171	19.62037	1.30E-42	3.09E-41	86.30331	up
HP_PSYCH	-0.65555	-0.05419	-13.034	3.54E-26	2.86E-25	48.45542	down
ABBUD_LIF	-0.65526	-0.02279	-18.3383	1.42E-39	2.70E-38	79.30581	down
GOBP_CHI	0.654965	0.044625	19.10241	2.14E-41	4.64E-40	83.50096	up
REACTOM	0.65496	-0.05129	16.54396	3.52E-35	4.92E-34	69.18073	up
GAUTSCHI	-0.65488	0.202229	-11.8954	3.46E-23	2.28E-22	41.58066	down
TSUNODA	-0.65485	0.017128	-25.3677	3.94E-55	3.01E-53	115.1366	down
RUIZ_TNC_	0.654746	-0.00959	19.66017	1.05E-42	2.51E-41	86.51724	up
chr4q32	-0.65473	0.056748	-18.0376	7.52E-39	1.35E-37	77.63529	down
GOMF_DN	0.654449	-0.01115	19.26457	8.86E-42	1.98E-40	84.38192	up
WP_PPAR_	-0.65434	-0.06984	-22.2922	1.18E-48	4.78E-47	100.2203	down
GOBP_NEL	-0.65429	-0.013	-25.6326	1.15E-55	9.29E-54	116.3678	down
HP_ABNOI	-0.65419	-0.07611	-23.0028	3.40E-50	1.61E-48	103.7701	down
GOMF_UN	0.654144	0.037951	12.483	9.87E-25	7.22E-24	45.1313	up
WP_DNA_I	0.654078	-0.08815	12.27685	3.44E-24	2.43E-23	43.88585	up
MODULE_:	0.654022	0.013992	20.59488	7.33E-45	2.10E-43	91.48419	up

GOBP_REG	0.65394	-0.0676	10.44815	2.17E-19	1.09E-18	32.86182	up
GOBP_POS	0.653718	0.05423	18.02793	7.93E-39	1.43E-37	77.58162	up
REACTOM	-0.65362	0.070133	-11.3836	7.67E-22	4.60E-21	38.48869	down
GOBP_PRII	-0.65356	-0.05197	-18.5962	3.41E-40	6.74E-39	80.72964	down
REACTOM	-0.65354	0.009578	-17.6394	6.97E-38	1.18E-36	75.4066	down
GOBP_POS	-0.65349	0.127168	-8.0443	2.85E-13	9.22E-13	18.85992	down
GOBP_HIS	0.653466	0.01258	10.99597	8.00E-21	4.46E-20	36.1515	up
GOCC_ORI	0.653435	-0.09039	12.58766	5.24E-25	3.90E-24	45.76332	up
HP_ACEPH	0.653367	0.007619	12.7677	1.77E-25	1.36E-24	46.84993	up
ZHANG_AI	-0.65334	-0.04549	-23.4229	4.30E-51	2.22E-49	105.8392	down
WP_THE_E	0.653333	-0.00899	18.8205	9.96E-41	2.05E-39	81.96167	up
HP_ABNOI	-0.65295	-0.0787	-18.9205	5.76E-41	1.21E-39	82.50857	down
MIR4315	0.652908	-0.11984	11.18798	2.51E-21	1.45E-20	37.30847	up
GOBP_NO	-0.65288	0.209247	-12.7875	1.57E-25	1.21E-24	46.96932	down
GOMF_LA(	-0.65277	0.018336	-13.8128	3.28E-28	3.02E-27	53.13303	down
GOBP_ATF	-0.65264	-0.1258	-12.0537	1.33E-23	9.01E-23	42.53736	down
GOBP_BILE	-0.65262	-0.09188	-19.8616	3.57E-43	8.85E-42	87.59674	down
GOBP_MIC	0.65256	-0.00944	27.18902	9.73E-59	1.01E-56	123.4385	up
SUMI_HNF	-0.65239	-0.04581	-14.7888	9.82E-31	1.06E-29	58.94207	down
CAIRO_HE	0.652188	-0.03137	20.71388	3.93E-45	1.15E-43	92.1086	up
REACTOM	0.65167	-0.02658	19.00032	3.73E-41	7.93E-40	82.94464	up
GOBP_POS	-0.65145	-0.06658	-27.0362	1.92E-58	1.95E-56	122.7566	down
GOBP_NE(	0.651408	-0.15155	15.5146	1.37E-32	1.65E-31	63.21186	up
GOCC_ENI	0.651404	-0.12196	12.41934	1.45E-24	1.05E-23	44.74681	up
GOMF_TES	-0.65125	-0.16854	-13.5546	1.54E-27	1.36E-26	51.58575	down
CHIANG_L	-0.65114	0.042058	-15.5194	1.34E-32	1.60E-31	63.23969	down
GOBP_PRC	0.651065	-0.01328	23.77779	7.61E-52	4.26E-50	107.57	up
GOCC_TRI	0.650921	-0.11735	9.994231	3.29E-18	1.53E-17	30.15365	up
GOCC_CHI	0.650858	-0.00541	18.12287	4.67E-39	8.55E-38	78.11031	up
GOBP_SIST	0.650708	-0.02357	21.70257	2.36E-47	8.31E-46	97.22692	up
REACTOM	0.650706	0.01961	14.78804	9.87E-31	1.06E-29	58.93733	up
GOBP_NE(	-0.65066	0.01282	-17.4314	2.25E-37	3.65E-36	74.23534	down
GOMF_OX	-0.65063	-0.02435	-25.5084	2.04E-55	1.62E-53	115.7916	down
MORF_RRI	0.650623	-0.04327	15.50127	1.48E-32	1.78E-31	63.13384	up
WP_UREA_	-0.65056	-0.07387	-14.2201	2.88E-29	2.82E-28	55.56511	down
GOBP_NEU	-0.65044	-0.01663	-16.1829	2.82E-34	3.73E-33	67.09924	down
REACTOM	-0.65041	0.027636	-15.34	3.82E-32	4.46E-31	62.18886	down
ONGUSAH	-0.65041	-0.00774	-17.7783	3.20E-38	5.53E-37	76.18657	down
ASXL2_TAI	0.650358	-0.05707	21.37134	1.29E-46	4.26E-45	95.52595	up
GOBP_NE(	-0.6503	0.169968	-11.043	6.02E-21	3.39E-20	36.43483	down
GOBP_CEL	-0.64973	-0.06199	-31.6764	5.77E-67	1.35E-64	142.3543	down
GOMF_AC	-0.64954	-0.08658	-23.3879	5.10E-51	2.61E-49	105.6676	down
HP_CARDI	-0.64925	-0.045	-15.2012	8.63E-32	9.87E-31	61.37406	down
GOBP_RNA	0.649249	-0.12219	9.742208	1.48E-17	6.55E-17	28.65928	up
GOBP_PTE	-0.64923	-0.11264	-15.6286	7.05E-33	8.64E-32	63.87808	down
GOBP_REG	0.649103	0.033485	11.35977	8.87E-22	5.29E-21	38.34476	up
HP_MACR(	-0.64909	-0.01359	-14.5682	3.63E-30	3.76E-29	57.63519	down
GOMF_PRI	0.649044	-0.28567	8.210495	1.11E-13	3.71E-13	19.79237	up
GOBP_FEM	0.648989	-0.02813	18.49328	6.01E-40	1.17E-38	80.16261	up
GOMF_PH	-0.64892	0.071454	-20.1781	6.61E-44	1.75E-42	89.28333	down
KAZMIN_P	-0.64885	-0.00817	-12.0305	1.53E-23	1.03E-22	42.39712	down
HP_ANGIC	-0.64827	0.057513	-16.3021	1.41E-34	1.91E-33	67.7883	down
LOPEZ_ME	-0.64818	-0.05425	-16.334	1.18E-34	1.60E-33	67.97233	down
GOMF_HIS	0.648056	0.055601	18.91924	5.80E-41	1.22E-39	82.50191	up
SHEDDEN_	0.648001	-0.01726	18.28886	1.86E-39	3.51E-38	79.03208	up
REACTOM	0.647914	-0.05159	13.50661	2.06E-27	1.81E-26	51.29759	up
ANDERSOI	-0.64786	0.135423	-7.67813	2.21E-12	6.72E-12	16.83195	down
GOMF_FLA	-0.64768	-0.11048	-23.6646	1.32E-51	7.23E-50	107.0197	down

WEINBERG	-0.64738	0.019073	-11.8719	3.99E-23	2.62E-22	41.43825	down
MATZUK_I	-0.64726	-0.08792	-17.8217	2.51E-38	4.38E-37	76.4295	down
KEGG_MIS	0.647226	-0.10435	12.99088	4.59E-26	3.69E-25	48.19556	up
RHODES_C	0.647215	-0.06475	16.98934	2.75E-36	4.19E-35	71.7285	up
GOMF_NA	-0.647	-0.08067	-21.4069	1.07E-46	3.58E-45	95.709	down
GOBP_PRC	-0.64683	-0.04743	-18.6907	2.03E-40	4.06E-39	81.2497	down
GOBP_HYD	-0.64679	-0.05631	-10.8955	1.47E-20	8.02E-20	35.54701	down
REACTOM	0.646742	-0.06897	17.33549	3.86E-37	6.19E-36	73.69319	up
FLECHNER	-0.64663	-0.04908	-27.2426	7.66E-59	8.08E-57	123.677	down
GOBP_DET	-0.64662	-0.04522	-31.9447	1.98E-67	4.92E-65	143.421	down
GOBP_CAI	-0.64658	-0.05478	-14.8841	5.59E-31	6.12E-30	59.50504	down
GOBP_NO	-0.64654	-0.06731	-18.7027	1.90E-40	3.82E-39	81.31558	down
GOBP_RES	-0.64653	-0.10457	-24.6763	1.02E-53	6.75E-52	111.8832	down
GOBP_RES	-0.64629	-0.0215	-11.7417	8.78E-23	5.62E-22	40.65129	down
GOBP_REG	-0.64616	-0.10204	-12.8274	1.23E-25	9.59E-25	47.20987	down
GOBP_POS	0.646103	-0.01808	10.73892	3.77E-20	2.01E-19	34.60555	up
GOCC_MIC	-0.64596	-0.10863	-21.5047	6.49E-47	2.20E-45	96.21264	down
PID_HNF3I	-0.64594	-0.01494	-17.771	3.33E-38	5.74E-37	76.14555	down
GOBP_DOI	-0.64581	0.033868	-18.7175	1.75E-40	3.54E-39	81.39697	down
GOBP_CEL	-0.64561	-0.07828	-28.0408	2.27E-60	2.76E-58	127.1926	down
HP_ABNOI	-0.64559	-0.05426	-22.9183	5.17E-50	2.41E-48	103.3515	down
REACTOM	0.645452	0.014982	14.80173	9.10E-31	9.81E-30	59.01832	up
GOMF_PH	-0.64544	0.063087	-13.7196	5.73E-28	5.19E-27	52.5746	down
REACTOM	-0.64532	-0.02945	-26.5217	1.95E-57	1.84E-55	120.4406	down
GOBP_POS	-0.64529	-0.06784	-18.7412	1.54E-40	3.13E-39	81.52688	down
GARGALO	0.645047	-0.10723	17.01841	2.33E-36	3.56E-35	71.89401	up
MEBARKI_I	-0.64501	-0.07582	-27.3781	4.20E-59	4.56E-57	124.2785	down
KEGG_DRU	-0.64494	-0.10266	-18.5975	3.39E-40	6.70E-39	80.73716	down
MODULE_	-0.64466	0.06816	-19.59	1.53E-42	3.62E-41	86.14003	down
WP_STATII	-0.64457	-0.06079	-14.3499	1.33E-29	1.33E-28	56.33795	down
TURASHVI	-0.64439	0.092088	-12.2898	3.18E-24	2.26E-23	43.96386	down
GOBP_RES	0.644367	-0.06935	16.44323	6.27E-35	8.68E-34	68.60146	up
HP_GLOM	-0.64435	-0.07515	-13.0445	3.32E-26	2.70E-25	48.51855	down
MODULE_	-0.64415	-0.15172	-15.0181	2.54E-31	2.82E-30	60.29594	down
GOBP_RES	-0.6441	0.023537	-25.0343	1.88E-54	1.33E-52	113.5749	down
GOBP_PEP	-0.64385	0.158533	-11.3516	9.31E-22	5.55E-21	38.29547	down
MODULE_	-0.64374	-0.03026	-19.7139	7.86E-43	1.90E-41	86.80597	down
GOBP_ATR	-0.64369	0.155398	-16.297	1.46E-34	1.97E-33	67.75841	down
GOBP_NEC	-0.6434	-0.04581	-10.6338	7.10E-20	3.72E-19	33.97423	down
HP_BRANC	0.643316	-0.04777	13.4294	3.27E-27	2.84E-26	50.83394	up
GOBP_PRC	-0.64327	-0.02468	-20.4355	1.69E-44	4.71E-43	90.64506	down
GOBP_NEU	-0.64316	-0.06615	-21.9889	5.48E-48	2.07E-46	98.68601	down
GOBP_SON	0.643126	-0.04701	14.37595	1.14E-29	1.14E-28	56.49309	up
HP_FRONT	0.642865	0.097549	13.52721	1.82E-27	1.60E-26	51.42121	up
GOBP_CEL	-0.6428	-0.01566	-24.275	6.91E-53	4.21E-51	109.9693	down
GOMF_MA	-0.64273	0.075849	-19.6991	8.51E-43	2.05E-41	86.72607	down
GOMF_HY	-0.64264	-0.06623	-26.1726	9.57E-57	8.46E-55	118.8523	down
GOBP_NEC	-0.64206	0.16016	-10.0627	2.19E-18	1.03E-17	30.56109	down
PID_FOXM	0.642017	0.033894	20.58691	7.64E-45	2.18E-43	91.4423	up
HP_THIN_I	0.641802	0.036839	22.14605	2.47E-48	9.66E-47	99.48255	up
HP_ABNOI	-0.64103	-0.07112	-10.8348	2.11E-20	1.15E-19	35.18197	down
TRAVAGLII	-0.64086	0.048744	-12.5828	5.40E-25	4.01E-24	45.73373	down
HAY_BONI	0.640849	-0.03408	18.31659	1.60E-39	3.03E-38	79.18574	up
GNF2_HAT	0.640791	-0.03262	13.13413	1.94E-26	1.60E-25	49.05835	up
GOBP_VER	-0.64075	-0.07999	-13.3549	5.12E-27	4.38E-26	50.38631	down
REACTOM	0.640595	0.016807	15.37689	3.08E-32	3.61E-31	62.40532	up
GOBP_PRC	-0.6405	-0.06505	-15.5253	1.29E-32	1.55E-31	63.27461	down
WP_TUMC	0.64	0.012755	21.76803	1.69E-47	6.04E-46	97.56144	up

KEGG_POR	-0.63975	-0.08884	-15.8508	1.93E-33	2.44E-32	65.17331	down
GOBP_NEC	-0.63948	0.211509	-9.45716	8.00E-17	3.37E-16	26.97877	down
GOCC_LEV	-0.63946	-0.12419	-14.5764	3.46E-30	3.59E-29	57.68355	down
REACTOM	0.639412	-0.01621	22.49625	4.24E-49	1.78E-47	101.2463	up
WP_HOMC	0.639117	-0.01905	16.176	2.93E-34	3.88E-33	67.05957	up
GOBP_MIT	0.638975	-0.07021	22.52747	3.62E-49	1.54E-47	101.4028	up
GOBP_REG	0.638844	-0.05828	13.46779	2.60E-27	2.27E-26	51.06452	up
GOMF_MH	-0.6388	0.075722	-8.92111	1.86E-15	7.09E-15	23.85194	down
MODULE_4	-0.63878	-0.0405	-13.8691	2.34E-28	2.18E-27	53.46987	down
HP_INCRE	0.638741	0.009113	13.33681	5.71E-27	4.87E-26	50.27759	up
KEGG_STEI	-0.63868	-0.09819	-18.1526	3.96E-39	7.27E-38	78.27591	down
GOBP_GLU	0.63867	-0.06066	11.21602	2.12E-21	1.23E-20	37.4775	up
HP_DILATE	0.638657	0.004031	14.77236	1.08E-30	1.16E-29	58.84461	up
REACTOM	-0.63824	-0.0688	-13.1198	2.11E-26	1.74E-25	48.97221	down
MODULE_4	-0.63782	-0.02321	-16.8411	6.41E-36	9.48E-35	70.88292	down
GOBP_UBI	-0.63777	-0.16176	-13.9871	1.16E-28	1.09E-27	54.17503	down
REACTOM	0.637767	-0.05413	10.87713	1.64E-20	8.93E-20	35.43631	up
HP_MACR	-0.63736	0.055515	-12.3353	2.41E-24	1.72E-23	44.23903	down
GOBP_G2_	0.636869	-0.07562	21.89477	8.84E-48	3.24E-46	98.20753	up
ERWIN_CC	-0.63686	0.110842	-9.94138	4.51E-18	2.08E-17	29.83969	down
HP_MICRC	-0.63661	-0.0178	-23.9818	2.83E-52	1.64E-50	108.5579	down
GOBP_PYR	-0.63647	-0.05568	-11.0929	4.45E-21	2.53E-20	36.73511	down
GOMF_PYI	-0.63647	-0.05568	-11.0929	4.45E-21	2.53E-20	36.73511	down
chr1q22	0.636332	-0.14372	13.73143	5.34E-28	4.85E-27	52.64566	up
GOCC_NU	0.636315	-0.03271	11.18341	2.58E-21	1.49E-20	37.28089	up
CLAUS_PG	-0.63631	-0.04246	-16.3534	1.05E-34	1.44E-33	68.08426	down
HP_APLAS	-0.63628	-0.00679	-15.4906	1.58E-32	1.89E-31	63.07127	down
GOBP_STE	-0.63608	-0.06778	-22.5747	2.86E-49	1.22E-47	101.6394	down
GOBP_SUL	-0.63601	-0.07981	-17.1352	1.20E-36	1.86E-35	72.55792	down
GSE24634_	0.635544	-0.01148	18.79965	1.12E-40	2.29E-39	81.8474	up
CAO_BLOC	-0.63544	-0.07335	-9.02937	9.89E-16	3.83E-15	24.47947	down
GOBP_CILI	0.63532	0.009055	24.51995	2.14E-53	1.39E-51	111.1399	up
HP_MYOG	-0.63524	-0.02179	-18.0419	7.34E-39	1.32E-37	77.65955	down
GOMF_5_D	0.635233	-0.05025	10.78326	2.89E-20	1.55E-19	34.87197	up
GOBP_REG	-0.63508	-0.00488	-12.1736	6.42E-24	4.46E-23	43.26191	down
GOBP_NAI	0.634828	-0.02258	14.90603	4.91E-31	5.40E-30	59.63463	up
GOBP_ATT	0.634808	-0.11909	12.43111	1.35E-24	9.81E-24	44.81789	up
TRAVAGLI	-0.63464	-0.02456	-16.3863	8.71E-35	1.20E-33	68.27356	down
HP_REDUC	-0.63451	0.106107	-11.1616	2.94E-21	1.69E-20	37.14945	down
HP_VISUAI	-0.63434	0.016315	-14.6301	2.51E-30	2.64E-29	58.0021	down
GOMF_HYI	-0.63433	-0.09532	-15.8921	1.52E-33	1.93E-32	65.41339	down
GOBP_DNA	0.634329	0.027395	13.93485	1.58E-28	1.48E-27	53.86294	up
GOMF_MU	0.634025	-0.02609	12.59668	4.96E-25	3.70E-24	45.81781	up
HP_IMBAL	-0.63381	0.064416	-11.4375	5.54E-22	3.36E-21	38.81388	down
CAIRO_HE	0.633542	-0.0575	16.11817	4.10E-34	5.38E-33	66.72489	up
GOMF_CH	0.633508	0.005886	13.17493	1.51E-26	1.26E-25	49.30397	up
HP_CEREBI	-0.63335	-0.02757	-22.7418	1.24E-49	5.55E-48	102.4739	down
GOBP_REG	0.633284	-0.09559	18.07937	5.95E-39	1.08E-37	77.86823	up
GOBP_CHY	-0.63313	-0.13544	-11.2905	1.35E-21	7.93E-21	37.92682	down
MODULE_3	-0.63312	-0.09391	-14.7651	1.13E-30	1.21E-29	58.80157	down
GOCC_ENI	-0.6331	-0.0517	-17.5057	1.48E-37	2.44E-36	74.65419	down
GOMF_OX	-0.63302	-0.04744	-19.6108	1.37E-42	3.24E-41	86.25203	down
HP_HEMAI	-0.63279	-0.0334	-18.8665	7.74E-41	1.61E-39	82.21335	down
HP_PROLC	-0.6327	-0.05004	-17.252	6.20E-37	9.78E-36	73.22078	down
GOMF_AM	-0.63243	-0.04056	-23.0536	2.64E-50	1.26E-48	104.0218	down
GOBP_HYD	-0.63236	-0.04889	-25.4007	3.38E-55	2.60E-53	115.2903	down
GOBP_REG	-0.63235	-0.01779	-13.0999	2.38E-26	1.95E-25	48.85231	down
GOBP_COI	-0.63223	-0.00942	-19.884	3.16E-43	7.89E-42	87.71692	down

MARIADAS	-0.63216	-0.00518	-12.8307	1.21E-25	9.40E-25	47.23012	down
GOBP_POS	-0.63199	0.131179	-11.3743	8.12E-22	4.86E-21	38.43265	down
BURTON_A	-0.6319	0.034203	-14.8288	7.75E-31	8.41E-30	59.17818	down
GNF2_FOS	-0.6315	0.114187	-11.8789	3.83E-23	2.51E-22	41.48062	down
HP_ABNOI	0.631444	-0.00024	19.63945	1.17E-42	2.80E-41	86.40593	up
LANDIS_EF	-0.63144	0.019454	-21.9372	7.12E-48	2.65E-46	98.42328	down
GOBP_POS	0.631265	0.018746	13.66442	7.98E-28	7.16E-27	52.24414	up
GOMF_EPC	-0.63125	-0.17102	-12.5337	7.27E-25	5.35E-24	45.43734	down
GOBP_PRC	0.631135	-0.10152	13.77931	4.01E-28	3.67E-27	52.9324	up
GOCC_MIS	0.631102	-0.07943	10.68805	5.12E-20	2.70E-19	34.30004	up
NAGASHIN	-0.63097	0.124387	-14.0915	6.20E-29	5.95E-28	54.79859	down
HP_MAND	0.630748	-0.09992	15.75729	3.33E-33	4.16E-32	64.62878	up
GOBP_SUL	-0.63065	-0.08925	-19.7148	7.82E-43	1.89E-41	86.81057	down
HP_CHILDI	-0.6306	-0.08191	-17.8958	1.66E-38	2.92E-37	76.84398	down
GOBP_MEI	0.630593	-0.03915	12.69423	2.75E-25	2.09E-24	46.40663	up
MODULE_!	-0.63047	-0.16994	-12.7143	2.44E-25	1.86E-24	46.52759	down
HP_HYPOC	0.630405	-0.16577	11.39747	7.06E-22	4.25E-21	38.57227	up
HP_DECRE	0.630257	-0.03736	12.28728	3.23E-24	2.29E-23	43.94888	up
GSE25088_	0.630167	-0.00896	20.4959	1.23E-44	3.46E-43	90.96346	up
MODULE_!	-0.63015	-0.10366	-14.8161	8.36E-31	9.03E-30	59.1034	down
GOBP_NEC	0.630087	-0.1066	9.892846	6.03E-18	2.75E-17	29.5516	up
REACTOM	-0.63008	-0.0322	-20.123	8.86E-44	2.32E-42	88.99059	down
FIRESTEIN_	0.630059	0.026808	14.34049	1.40E-29	1.40E-28	56.28217	up
GROSS_HY	-0.62962	0.030406	-18.9698	4.40E-41	9.33E-40	82.77829	down
GOMF_TRA	-0.62961	0.004245	-14.8071	8.82E-31	9.52E-30	59.04983	down
GOBP_SPII	0.629528	-0.01015	28.22291	1.03E-60	1.30E-58	127.9847	up
HP_NEOPL	0.62945	0.006921	12.02113	1.62E-23	1.09E-22	42.34032	up
WHITFIELD	0.629396	-0.02782	24.20491	9.68E-53	5.81E-51	109.6327	up
HP_ABNOI	-0.6293	-0.0657	-11.1127	3.95E-21	2.25E-20	36.85472	down
SMID_BREA	-0.62927	0.038837	-13.0699	2.85E-26	2.33E-25	48.67142	down
MCCOLLU	0.629118	0.006086	12.34464	2.28E-24	1.63E-23	44.29547	up
HP_STUTTI	-0.62911	-0.03208	-12.1781	6.25E-24	4.34E-23	43.2891	down
WP_TRANS	-0.62894	-0.01287	-18.3903	1.06E-39	2.04E-38	79.59353	down
GOBP_TRIC	-0.62893	-0.06287	-23.8591	5.13E-52	2.91E-50	107.9645	down
GOBP_REG	-0.62892	-0.00676	-11.9285	2.83E-23	1.88E-22	41.78061	down
REACTOM	0.628872	0.013917	14.33564	1.45E-29	1.44E-28	56.25333	up
AMIT_EGF_	-0.62874	0.072099	-17.0825	1.62E-36	2.49E-35	72.25857	down
GOMF_D_C	-0.62869	-0.0202	-15.3988	2.71E-32	3.19E-31	62.53359	down
REACTOM	0.628668	-0.08042	13.65003	8.70E-28	7.79E-27	52.15788	up
HP_STAPES	-0.62836	0.067468	-12.1038	9.80E-24	6.72E-23	42.84017	down
GOCC_NU	0.628249	0.02582	16.51689	4.11E-35	5.73E-34	69.02517	up
GOBP_CEL	0.628007	-0.03318	12.57397	5.70E-25	4.22E-24	45.68066	up
GOBP_NEC	0.627923	-0.03339	21.54029	5.41E-47	1.85E-45	96.39528	up
WP_TCA_C	-0.62786	-0.11741	-11.8133	5.69E-23	3.70E-22	41.08408	down
GOMF_CY(	-0.62767	0.028876	-12.3677	1.98E-24	1.43E-23	44.43504	down
SCHAEFFEI	-0.62764	0.097452	-12.482	9.93E-25	7.26E-24	45.12554	down
GOBP_ATF	0.627524	-0.02265	22.61217	2.37E-49	1.02E-47	101.8267	up
GOMF_DIA	0.627497	0.013964	13.98199	1.19E-28	1.13E-27	54.14462	up
WP_MIRNA	0.627439	0.032355	11.61393	1.90E-22	1.19E-21	39.87948	up
GOMF_RA(	-0.62742	-0.02731	-22.2825	1.24E-48	5.01E-47	100.1716	down
GOBP_REG	-0.62737	0.094193	-9.25745	2.60E-16	1.05E-15	25.80831	down
GOCC_MIT	0.627256	0.075584	11.2971	1.30E-21	7.63E-21	37.96656	up
KEGG_PEN	-0.62717	-0.05974	-14.2227	2.83E-29	2.78E-28	55.58108	down
REACTOM	-0.62706	-0.02129	-16.7136	1.33E-35	1.92E-34	70.15354	down
HP_ENLAR	-0.62683	-0.04515	-18.5869	3.59E-40	7.08E-39	80.67859	down
GOCC_INT	-0.62682	-0.0938	-17.6512	6.52E-38	1.11E-36	75.47301	down
GOCC_AST	-0.62656	0.174597	-13.3043	6.95E-27	5.89E-26	50.08246	down
GOMF_RN	0.626481	-0.07537	9.889702	6.14E-18	2.80E-17	29.53295	up

GOBP_EST	0.626442	-0.0791	13.38199	4.35E-27	3.74E-26	50.54915	up
GOBP_NU(	-0.62595	-0.06476	-13.7882	3.80E-28	3.48E-27	52.98565	down
REACTOM	-0.62595	-0.11259	-16.1073	4.36E-34	5.72E-33	66.662	down
GOBP_REG	-0.62591	0.089947	-18.8572	8.14E-41	1.69E-39	82.16259	down
HOEK_T_C	0.625778	-0.01988	9.528129	5.26E-17	2.24E-16	27.39612	up
GOMF_HYI	-0.62568	0.125822	-12.3044	2.91E-24	2.07E-23	44.05243	down
HP_LOSS_(	-0.62555	0.000684	-20.487	1.29E-44	3.62E-43	90.91674	down
GOMF_IMI	-0.62554	0.149834	-8.83484	3.07E-15	1.15E-14	23.35349	down
GOCC_CHI	0.625502	-0.02452	21.90707	8.30E-48	3.06E-46	98.27012	up
GOBP_PEP	0.625158	0.037845	9.012339	1.09E-15	4.22E-15	24.38063	up
GOMF_PR(	0.625158	0.037845	9.012339	1.09E-15	4.22E-15	24.38063	up
GAVIN_FO	0.625081	-0.02979	16.41166	7.52E-35	1.04E-33	68.41971	up
KAUFFMAI	-0.62482	-0.13028	-13.5764	1.35E-27	1.20E-26	51.71646	down
GOBP_DUI	-0.62481	-0.0799	-12.9019	7.85E-26	6.20E-25	47.65939	down
REACTOM	0.624545	-0.09643	11.75822	7.95E-23	5.10E-22	40.75129	up
GOBP_REG	0.624333	0.057873	9.356066	1.45E-16	6.01E-16	26.38551	up
HP_WIDE_I	0.624275	-0.01976	14.41968	8.77E-30	8.88E-29	56.75313	up
GNF2_CD1	-0.62412	0.120752	-10.1637	1.19E-18	5.73E-18	31.16262	down
HP_ABNOI	0.624053	-0.03278	19.13176	1.82E-41	3.98E-40	83.66063	up
TIAN_BHLI	-0.62397	0.052762	-14.6716	1.97E-30	2.08E-29	58.24797	down
GOCC_ENI	-0.62396	0.109583	-9.0602	8.26E-16	3.22E-15	24.65857	down
HP_FULMII	-0.62396	0.043543	-11.6217	1.82E-22	1.14E-21	39.92633	down
SHEPARD_	0.623936	0.017444	23.93172	3.61E-52	2.08E-50	108.316	up
REACTOM	-0.62389	-0.0117	-18.4946	5.97E-40	1.16E-38	80.17014	down
MODULE_!	-0.62386	0.077763	-16.1951	2.62E-34	3.49E-33	67.16984	down
MODULE_!	-0.62386	0.007903	-15.4499	2.01E-32	2.38E-31	62.83294	down
BIOCARTA	-0.62368	0.101311	-13.7268	5.49E-28	4.98E-27	52.61781	down
WP_CELLS_	-0.62368	0.101311	-13.7268	5.49E-28	4.98E-27	52.61781	down
HP_CHROI	0.623556	-0.06992	11.46771	4.61E-22	2.81E-21	38.99634	up
HP_SKULL_	-0.6235	0.063656	-12.4819	9.94E-25	7.26E-24	45.12437	down
HP_ABNOI	-0.62343	-0.10481	-19.8476	3.84E-43	9.50E-42	87.52211	down
REACTOM	-0.62341	-0.07284	-22.6836	1.66E-49	7.27E-48	102.1836	down
BIOCARTA	0.623395	0.043904	14.15093	4.35E-29	4.21E-28	55.15306	up
GOBP_NE(	-0.62331	0.066272	-21.9722	5.96E-48	2.24E-46	98.60136	down
ICHIBA_GF	-0.62321	0.000561	-22.9003	5.65E-50	2.62E-48	103.2621	down
MORI_PRE	0.623201	-0.03605	17.58389	9.52E-38	1.59E-36	75.09461	up
WP_HEME_	-0.62315	-0.15446	-12.9756	5.04E-26	4.03E-25	48.10321	down
GOBP_HIS	0.622734	0.071535	11.52531	3.26E-22	2.00E-21	39.34417	up
GOBP_NE(	-0.62259	0.212913	-9.65735	2.45E-17	1.07E-16	28.15783	down
GOBP_TEL	0.622546	-0.06152	18.26583	2.11E-39	3.97E-38	78.90439	up
GOBP_NE(	-0.62232	0.027045	-20.6946	4.34E-45	1.27E-43	92.00761	down
GOBP_POI	-0.62199	-0.1136	-18.5022	5.72E-40	1.12E-38	80.21203	down
HP_DECRE	-0.62189	-0.17916	-13.0046	4.23E-26	3.40E-25	48.27836	down
MIR6741_3	-0.62186	0.059603	-11.3901	7.38E-22	4.43E-21	38.52809	down
HP_CORTI(	-0.62177	0.053261	-12.384	1.80E-24	1.30E-23	44.53308	down
GOMF_RN	-0.62165	0.052725	-13.1428	1.84E-26	1.52E-25	49.11043	down
HP_OPTIC_	-0.62165	-0.1612	-16.3752	9.28E-35	1.27E-33	68.20984	down
GOBP_LUN	-0.62134	0.191585	-11.1556	3.05E-21	1.75E-20	37.11298	down
HP_INCRE	-0.62132	-0.00431	-16.2002	2.55E-34	3.39E-33	67.1995	down
GOBP_POS	0.621242	0.036407	9.789891	1.11E-17	4.98E-17	28.94144	up
GOMF_RA(	-0.62116	0.037839	-18.8527	8.35E-41	1.73E-39	82.13794	down
HP_ABNOI	-0.621	-0.15105	-14.1239	5.11E-29	4.94E-28	54.99175	down
REACTOM	0.620886	0.006391	24.3162	5.67E-53	3.51E-51	110.1665	up
GOBP_CEL	-0.62081	-0.04589	-19.3073	7.03E-42	1.59E-40	84.61323	down
GOBP_5S_(	0.620736	-0.01872	11.82121	5.43E-23	3.53E-22	41.13195	up
GOCC_TRA	0.620736	-0.01872	11.82121	5.43E-23	3.53E-22	41.13195	up
GOBP_OVI	-0.62071	0.15119	-11.9557	2.40E-23	1.60E-22	41.94481	down
GOBP_NU(	0.620614	0.005536	20.21144	5.54E-44	1.47E-42	89.46	up

LUCAS_HN	-0.62056	-0.05031	-17.3649	3.27E-37	5.27E-36	73.85927	down
GOBP_HOI	0.620366	-0.05161	22.29282	1.18E-48	4.77E-47	100.2236	up
GOBP_ZYN	-0.62027	-0.0175	-27.5236	2.20E-59	2.43E-57	124.9227	down
HP_ABNOI	0.620143	-0.05427	12.79425	1.50E-25	1.16E-24	47.01009	up
SOUCEK_N	-0.61995	0.16386	-11.3432	9.80E-22	5.83E-21	38.24452	down
MODULE_!	-0.61992	-0.01526	-13.3926	4.09E-27	3.52E-26	50.61259	down
REACTOM	0.619909	0.005851	15.09621	1.60E-31	1.80E-30	60.75608	up
MANNO_N	0.619896	0.025999	21.63456	3.34E-47	1.17E-45	96.8788	up
GOMF_IRC	-0.61975	-0.04279	-34.0144	6.49E-71	2.62E-68	151.4238	down
HP_ACUTE	0.619444	-0.03248	10.75626	3.40E-20	1.81E-19	34.70974	up
GOMF_VIT	-0.6193	-0.06992	-27.9177	3.89E-60	4.53E-58	126.6547	down
GERHOLD_	-0.61885	0.044007	-16.9939	2.68E-36	4.08E-35	71.75468	down
MODULE_!	-0.61871	-0.02067	-13.4045	3.80E-27	3.28E-26	50.68444	down
WP_TCA_C	-0.6184	-0.10143	-12.2404	4.29E-24	3.02E-23	43.66546	down
BERENJEN	-0.61807	-0.04546	-22.6492	1.97E-49	8.55E-48	102.0119	down
GOBP_DITI	-0.61801	-0.01931	-15.8698	1.73E-33	2.19E-32	65.28353	down
BENPORAT	0.617958	-0.00677	22.14135	2.53E-48	9.87E-47	99.45879	up
REACTOM	-0.61792	-0.07739	-12.4295	1.36E-24	9.91E-24	44.80828	down
GRANDVA	-0.61789	-0.02704	-13.6092	1.11E-27	9.90E-27	51.91283	down
REACTOM	0.617859	-0.07981	14.89978	5.10E-31	5.59E-30	59.59774	up
CAFFAREL_	-0.61785	0.099642	-11.4076	6.64E-22	4.00E-21	38.63363	down
GOBP_NEC	-0.6178	0.121384	-12.8566	1.03E-25	8.09E-25	47.38621	down
GOBP_NEC	0.617742	0.034502	16.10742	4.36E-34	5.72E-33	66.66264	up
GOBP_NEC	-0.61771	0.041372	-17.5728	1.01E-37	1.69E-36	75.03199	down
REACTOM	0.617536	-0.01877	14.2641	2.21E-29	2.18E-28	55.82749	up
HP_HYPOL	-0.61751	-0.04804	-15.1024	1.54E-31	1.74E-30	60.79278	down
GOBP_LIPI	-0.61741	-0.05482	-30.2494	1.91E-64	3.44E-62	136.5639	down
GOMF_ELE	-0.6173	-0.17585	-16.624	2.22E-35	3.16E-34	69.64002	down
GOBP_HEN	-0.61729	-0.11893	-19.1048	2.11E-41	4.58E-40	83.51375	down
GOBP_TRN	0.617282	0.009525	14.06035	7.46E-29	7.14E-28	54.6126	up
GOMF_ATI	0.617017	0.015974	17.86334	1.99E-38	3.48E-37	76.66254	up
GOCC_CO	0.616938	-0.08828	11.50403	3.70E-22	2.27E-21	39.21564	up
HP_EPISOI	-0.61659	0.034618	-13.4725	2.53E-27	2.21E-26	51.09302	down
HP_ABNOI	-0.61657	0.061135	-13.5659	1.44E-27	1.28E-26	51.65313	down
HP_SHOUI	-0.61643	-0.01602	-11.5246	3.27E-22	2.01E-21	39.33962	down
STEIN_EST	-0.61621	0.039102	-17.9232	1.42E-38	2.52E-37	76.99736	down
GOBP_CHI	0.616053	-0.17916	9.402497	1.10E-16	4.61E-16	26.65779	up
REACTOM	0.615884	-0.06416	17.08517	1.60E-36	2.45E-35	72.27376	up
GROSS_ELI	-0.61579	0.016657	-17.8508	2.13E-38	3.73E-37	76.59233	down
GAZIN_EPI	0.615315	-0.00429	24.43347	3.24E-53	2.05E-51	110.7273	up
GOMF_THI	-0.61524	-0.07871	-13.3661	4.79E-27	4.10E-26	50.4537	down
GSE39110_	0.615098	-0.03529	17.64964	6.58E-38	1.12E-36	75.46434	up
HP_PATELI	0.615051	0.007157	14.14236	4.57E-29	4.43E-28	55.10192	up
MIR1204	-0.61504	-0.08434	-13.0706	2.84E-26	2.32E-25	48.67572	down
GOBP_POS	0.614884	0.020966	20.37668	2.31E-44	6.36E-43	90.33459	up
REACTOM	-0.61478	-0.01652	-18.0131	8.61E-39	1.54E-37	77.49883	down
MARKEY_F	0.614491	-0.02653	19.84388	3.92E-43	9.68E-42	87.50216	up
GOBP_LOC	-0.61446	0.047482	-16.7157	1.31E-35	1.90E-34	70.16601	down
HP_GEOGI	-0.61439	-0.05091	-10.8975	1.45E-20	7.93E-20	35.55865	down
GOMF_5_3	0.614385	-0.02937	15.84857	1.96E-33	2.47E-32	65.16021	up
GOCC_IGA	-0.61428	-0.1057	-6.83496	2.12E-10	5.53E-10	12.32361	down
REACTOM	0.614255	-0.08566	13.15629	1.69E-26	1.40E-25	49.19173	up
GOBP_EMI	-0.61392	-0.0145	-10.5139	1.46E-19	7.47E-19	33.25538	down
HP_SEVERI	0.61389	-0.12497	10.56083	1.10E-19	5.68E-19	33.53682	up
GOBP_C_T	-0.61387	0.129601	-10.5039	1.55E-19	7.91E-19	33.19586	down
GOBP_POS	0.613784	-0.03553	12.36997	1.96E-24	1.41E-23	44.44853	up
MOROSET	-0.61375	-0.02969	-19.2755	8.35E-42	1.87E-40	84.44121	down
GOBP_OXY	-0.61299	0.072696	-12.4551	1.17E-24	8.50E-24	44.96308	down

AMIT_EGF_	-0.61297	0.103025	-16.0181	7.31E-34	9.50E-33	66.14487	down
GOBP_PRC	-0.61277	0.109045	-10.1445	1.34E-18	6.41E-18	31.04799	down
GOBP_NE(	-0.61276	0.122592	-11.5602	2.64E-22	1.63E-21	39.55472	down
GSE21063_	0.61274	-0.06177	15.65503	6.04E-33	7.43E-32	64.03243	up
GOBP_ACU	-0.61268	0.032087	-23.5837	1.96E-51	1.05E-49	106.6254	down
GOBP_LON	-0.61246	-0.0245	-27.7917	6.76E-60	7.65E-58	126.1031	down
GOMF_TET	-0.6123	-0.0531	-35.6648	1.41E-73	7.71E-71	157.5334	down
KEGG_GLY	-0.61184	-0.03687	-21.5071	6.41E-47	2.18E-45	96.22497	down
GOBP_MIT	-0.61176	-0.09065	-12.5609	6.16E-25	4.56E-24	45.60162	down
CHEN_LVA	-0.61159	0.013251	-21.1349	4.38E-46	1.39E-44	94.30315	down
HP_CEREBI	-0.61135	-0.02061	-11.3276	1.08E-21	6.38E-21	38.15063	down
REACTOM	-0.61126	-0.05998	-22.7315	1.31E-49	5.80E-48	102.4225	down
HP_NARR(	-0.61125	0.048889	-14.1067	5.66E-29	5.45E-28	54.88944	down
SHANK_TA	-0.61123	0.085055	-12.9506	5.85E-26	4.66E-25	47.95293	down
MUNSHI_N	0.611038	-0.04842	17.15774	1.06E-36	1.65E-35	72.686	up
GOBP_PRC	0.610931	-0.04221	18.50388	5.67E-40	1.11E-38	80.22109	up
GOMF_EX(	0.610891	-0.05853	15.94425	1.12E-33	1.44E-32	65.71638	up
REACTOM	0.610666	-0.00647	23.91817	3.85E-52	2.21E-50	108.2504	up
GOBP_DEN	-0.61044	0.144685	-10.0221	2.79E-18	1.30E-17	30.3196	down
REACTOM	-0.6103	-0.06051	-11.3042	1.24E-21	7.31E-21	38.0095	down
GEORGES_	0.610025	0.041308	21.1777	3.51E-46	1.12E-44	94.52514	up
KEGG_NIC	-0.61002	-0.00684	-21.913	8.06E-48	2.98E-46	98.30011	down
GAUCHER_	-0.60995	0.040851	-12.7274	2.25E-25	1.72E-24	46.607	down
REACTOM	-0.60988	-0.0411	-14.6835	1.83E-30	1.94E-29	58.31883	down
GOBP_REG	0.609867	-0.03141	23.94701	3.35E-52	1.93E-50	108.3899	up
GOBP_L_SI	-0.60986	-0.023	-11.198	2.36E-21	1.37E-20	37.36872	down
GOBP_CEL	-0.60982	-0.00842	-19.1317	1.82E-41	3.98E-40	83.66008	down
HP_FLAT_(	0.609804	-0.15163	16.19078	2.69E-34	3.57E-33	67.14505	up
GOBP_PHE	-0.60975	-0.01217	-17.6929	5.16E-38	8.80E-37	75.70715	down
GOBP_SPIN	0.609545	-0.00343	26.18632	8.99E-57	7.97E-55	118.9152	up
GOBP_THI(	-0.60948	-0.10097	-19.9244	2.55E-43	6.42E-42	87.9324	down
MODULE_2	-0.60935	0.003755	-23.7513	8.65E-52	4.80E-50	107.4416	down
WANG_RE(	0.609318	-0.02407	16.73021	1.21E-35	1.75E-34	70.24878	up
GOBP_DN/	0.609124	0.06544	16.11945	4.06E-34	5.34E-33	66.73232	up
MIR1247_5	-0.60901	0.058271	-12.0702	1.20E-23	8.18E-23	42.63672	down
HP_HYPER	-0.60889	-0.09701	-8.21367	1.09E-13	3.65E-13	19.81023	down
GOBP_TRI(	-0.6088	-0.09125	-13.2996	7.15E-27	6.05E-26	50.05411	down
GOBP_GLU	-0.60872	-0.00586	-12.2402	4.29E-24	3.02E-23	43.66408	down
GOBP_POS	0.608256	0.022058	9.946425	4.38E-18	2.02E-17	29.86962	up
GOBP_REG	0.60825	-0.08078	14.66704	2.02E-30	2.13E-29	58.22107	up
GOBP_CEL	-0.60821	-0.07465	-10.6284	7.34E-20	3.84E-19	33.94208	down
GOMF_PO	-0.60814	0.103377	-16.5988	2.57E-35	3.62E-34	69.49542	down
REACTOM	0.608057	-0.03329	19.40201	4.21E-42	9.67E-41	85.12598	up
REACTOM	0.607927	0.031954	14.38352	1.09E-29	1.09E-28	56.53813	up
GOBP_CH(	-0.60783	-0.13146	-13.7685	4.28E-28	3.91E-27	52.86765	down
GOBP_MO	-0.60773	-0.06009	-29.409	6.40E-63	1.04E-60	133.058	down
GNF2_CD7	-0.60771	0.088836	-9.35233	1.49E-16	6.14E-16	26.36362	down
HP_NIGHT	-0.60764	0.01664	-15.4343	2.20E-32	2.61E-31	62.74158	down
GOMF_N_I	-0.60759	0.263801	-7.6527	2.54E-12	7.70E-12	16.69252	down
HALLMARI	-0.60747	-0.10566	-22.1392	2.56E-48	9.96E-47	99.44776	down
KEGG_GLY	-0.60737	-0.13152	-16.1062	4.39E-34	5.75E-33	66.65589	down
IIZUKA_LIV	-0.60725	-0.07066	-13.8944	2.01E-28	1.88E-27	53.6209	down
HASEGAW	-0.60706	0.050508	-13.3494	5.29E-27	4.52E-26	50.35357	down
WP_DERE(	-0.60698	0.092407	-14.1155	5.37E-29	5.19E-28	54.9417	down
GOBP_NU(	0.60696	-0.02468	22.26119	1.38E-48	5.54E-47	100.0641	up
REACTOM	0.60676	-0.0319	17.52216	1.35E-37	2.23E-36	74.74702	up
GOMF_AR(	-0.60676	-0.09578	-12.1504	7.39E-24	5.12E-23	43.12178	down
GOBP_NE(	-0.60674	0.143143	-10.0516	2.34E-18	1.10E-17	30.495	down

GSE36476_	0.606665	-0.07973	15.16115	1.09E-31	1.24E-30	61.13831	up
HP_GLYCC	-0.60657	-0.03289	-20.4213	1.83E-44	5.05E-43	90.57003	down
FARMER_B	-0.60644	0.051648	-14.6918	1.74E-30	1.85E-29	58.36765	down
GOCC_U4_	0.606416	-0.12506	9.866898	7.03E-18	3.19E-17	29.3977	up
HP_EXERCI	-0.60624	-0.00383	-15.6888	4.97E-33	6.14E-32	64.22919	down
GOBP_HIS	0.606242	-0.01996	21.22078	2.81E-46	9.03E-45	94.74822	up
GOBP_PRC	-0.60595	-0.109	-10.6015	8.63E-20	4.48E-19	33.78043	down
GOBP_REG	0.605941	-0.01577	16.6966	1.47E-35	2.11E-34	70.05632	up
GOCC_SPA	-0.60571	0.081031	-16.6124	2.37E-35	3.36E-34	69.57347	down
GOMF_HA	-0.60554	0.084649	-11.1521	3.11E-21	1.79E-20	37.09228	down
GOBP_FLA	-0.60545	-0.04965	-11.6432	1.59E-22	1.00E-21	40.05619	down
REACTOM	-0.60541	0.002904	-18.8149	1.03E-40	2.11E-39	81.93077	down
GOBP_ORG	-0.6053	-0.05866	-28.8533	6.79E-62	9.73E-60	130.6995	down
HP_RECUR	-0.60472	-0.11678	-11.8016	6.11E-23	3.96E-22	41.01353	down
REACTOM	-0.60472	-0.04159	-11.2982	1.29E-21	7.58E-21	37.97326	down
GOBP_HYD	-0.60419	-0.02512	-20.3107	3.28E-44	8.87E-43	89.98578	down
WP_OXIDA	-0.60416	-0.03786	-20.4239	1.80E-44	4.99E-43	90.58412	down
GOBP_MO	-0.60403	-0.06249	-28.7902	8.89E-62	1.25E-59	130.4299	down
GOMF_MIS	0.603985	-0.08383	13.8097	3.34E-28	3.07E-27	53.11435	up
HP_PROMI	-0.60395	0.088625	-13.1711	1.55E-26	1.29E-25	49.28062	down
GOMF_LEU	-0.60392	0.062213	-10.4933	1.65E-19	8.41E-19	33.13217	down
GOBP_POS	-0.60391	0.083528	-17.6798	5.56E-38	9.46E-37	75.63379	down
BIOCARTA	0.603753	-0.13911	9.622285	3.01E-17	1.31E-16	27.95093	up
NAKAMUR	0.603578	-0.06994	14.58293	3.33E-30	3.46E-29	57.72243	up
WP_MIR51	-0.60352	0.150271	-11.5586	2.66E-22	1.65E-21	39.54553	down
GOMF_PH	-0.60335	0.087051	-11.8341	5.02E-23	3.27E-22	41.20966	down
GOBP_RES	-0.60318	0.070116	-19.7655	5.96E-43	1.45E-41	87.08261	down
MODULE_2	-0.60308	-0.00898	-14.8828	5.63E-31	6.16E-30	59.49767	down
GOBP_HYD	-0.60265	-0.0768	-17.033	2.15E-36	3.29E-35	71.9772	down
GOBP_ADH	-0.6025	0.028786	-15.2696	5.77E-32	6.68E-31	61.77598	down
REACTOM	0.602453	0.03016	12.63154	4.02E-25	3.01E-24	46.02821	up
GOCC_NU	0.602404	0.104766	11.79042	6.54E-23	4.23E-22	40.94587	up
GSE14415_	0.602181	-0.03098	16.72804	1.22E-35	1.77E-34	70.23636	up
GOBP_REG	-0.60218	-0.07668	-12.4148	1.49E-24	1.08E-23	44.71917	down
GOMF_STF	0.602105	0.053267	14.9538	3.70E-31	4.09E-30	59.91665	up
KEGG_CELI	0.602069	-0.0225	20.10472	9.76E-44	2.54E-42	88.89329	up
GOCC_CO	0.601875	-0.01901	20.66925	4.96E-45	1.44E-43	91.87463	up
GOMF_TO	-0.6017	0.000729	-17.5561	1.11E-37	1.85E-36	74.9384	down
MODULE_4	-0.6016	0.056243	-17.9382	1.31E-38	2.32E-37	77.0811	down
GOBP_REC	0.601549	-0.00913	20.17081	6.87E-44	1.81E-42	89.24445	up
GOBP_NEG	0.601394	-0.05039	14.53837	4.33E-30	4.46E-29	57.45808	up
GOBP_MIT	0.601017	0.03811	18.69021	2.03E-40	4.07E-39	81.24689	up
HP_ELEVA	-0.60093	0.088072	-12.5284	7.50E-25	5.52E-24	45.40532	down
GOBP_RES	-0.60089	0.072707	-12.5602	6.19E-25	4.58E-24	45.59759	down
GOBP_THY	-0.60083	-0.04082	-14.9413	3.99E-31	4.39E-30	59.84303	down
GOBP_POS	0.600757	-0.11124	9.464243	7.67E-17	3.24E-16	27.02038	up
GOBP_NU	-0.60058	-0.1223	-13.9145	1.78E-28	1.67E-27	53.74123	down
HP_HYPOC	0.600145	-0.02611	15.12592	1.34E-31	1.52E-30	60.931	up
GNF2_DEK	0.600121	0.027388	12.35694	2.12E-24	1.52E-23	44.36981	up
GOBP_PYR	-0.60008	0.024046	-13.2718	8.45E-27	7.12E-26	49.88661	down
HP_INCRE	-0.59997	-0.06643	-22.003	5.10E-48	1.93E-46	98.75788	down
MANNO_M	0.599874	0.001925	19.97376	1.96E-43	4.98E-42	88.19596	up
GOBP_ME	-0.59959	-0.19249	-9.45367	8.17E-17	3.44E-16	26.95823	down
MEINHOLI	0.599535	-0.09302	14.01349	9.87E-29	9.38E-28	54.33284	up
LEE_SP4_TI	-0.59922	0.152912	-13.0422	3.37E-26	2.74E-25	48.50457	down
SARTIPY_B	-0.59914	0.138246	-14.4512	7.27E-30	7.40E-29	56.94038	down
HP_PERIPH	-0.59913	0.023669	-14.1669	3.95E-29	3.84E-28	55.24822	down
ACEVEDO_	-0.59904	-0.11793	-19.8836	3.17E-43	7.90E-42	87.7148	down

GOBP_POS	-0.59874	0.009911	-18.0317	7.77E-39	1.40E-37	77.60257	down
WP_CELL_(	0.598719	-0.02296	20.15715	7.39E-44	1.94E-42	89.17192	up
GOMF_PYI	-0.5986	-0.04484	-11.5393	2.99E-22	1.85E-21	39.42847	down
GOMF_DIA	-0.59856	-0.1181	-8.28625	7.23E-14	2.45E-13	20.21968	down
GOCC_REF	0.598485	-0.06217	15.60911	7.90E-33	9.65E-32	63.76433	up
REACTOM	0.598361	-0.07542	15.22866	7.34E-32	8.45E-31	61.53528	up
GOBP_INT	-0.59834	-0.00373	-14.4147	9.03E-30	9.12E-29	56.72343	down
GOMF_NA	-0.59825	-0.22328	-11.5313	3.14E-22	1.94E-21	39.38008	down
GOMF_MA	-0.59807	-0.04339	-11.3432	9.80E-22	5.83E-21	38.2446	down
GOBP_ARC	-0.59794	-0.06452	-16.7692	9.67E-36	1.41E-34	70.47205	down
GOBP_POS	0.59789	0.022835	11.23079	1.93E-21	1.13E-20	37.56657	up
GOBP_RES	-0.59779	0.023533	-18.3629	1.24E-39	2.37E-38	79.44203	down
GOBP_CEN	0.597701	0.020277	19.79131	5.19E-43	1.27E-41	87.22076	up
GOBP_POS	0.597697	-0.04035	16.36004	1.01E-34	1.39E-33	68.12231	up
GOBP_CAN	0.597485	0.019929	11.19572	2.39E-21	1.38E-20	37.35513	up
HP_LEYDIG	-0.59742	-0.01379	-10.7287	4.01E-20	2.13E-19	34.54437	down
HP_HYPOI	0.597403	-0.12121	11.98438	2.02E-23	1.35E-22	42.11819	up
HP_MAST(	-0.59735	0.124592	-12.1133	9.25E-24	6.35E-23	42.89715	down
GOBP_ANI	-0.59732	-0.04003	-14.5392	4.31E-30	4.44E-29	57.46326	down
HP_DECRE	-0.59712	-0.0234	-18.9503	4.90E-41	1.04E-39	82.67163	down
HP_INCRE/	-0.59699	-0.16375	-12.9375	6.33E-26	5.03E-25	47.87412	down
GOBP_MA	0.596988	0.023808	14.44966	7.34E-30	7.46E-29	56.93127	up
GOBP_CHI	0.596866	-0.02618	21.81782	1.31E-47	4.73E-46	97.81551	up
GOBP_POS	-0.59686	0.135133	-13.6673	7.85E-28	7.04E-27	52.26126	down
GOBP_POS	-0.59683	-0.09152	-8.31873	6.00E-14	2.05E-13	20.40335	down
BOYAULT_	0.596778	0.016206	14.106	5.68E-29	5.47E-28	54.88503	up
PYEON_HI	0.596687	-0.01294	17.69851	5.00E-38	8.54E-37	75.7388	up
GOBP_SIAI	-0.59654	-0.0784	-8.75995	4.74E-15	1.75E-14	22.9219	down
GOMF_SIA	-0.59654	-0.0784	-8.75995	4.74E-15	1.75E-14	22.9219	down
GOBP_SEN	0.596282	0.006144	13.03007	3.63E-26	2.93E-25	48.4317	up
GOBP_CEL	-0.59625	0.038381	-15.8914	1.53E-33	1.94E-32	65.40912	down
GOBP_NE(	-0.59604	0.121664	-9.42298	9.79E-17	4.10E-16	26.77799	down
GOBP_PH(	-0.5959	-0.03137	-16.8252	7.02E-36	1.04E-34	70.79201	down
BIOCARTA	-0.59585	0.169969	-10.5587	1.12E-19	5.75E-19	33.52386	down
HP_FINGEI	0.595767	-0.11556	11.40003	6.95E-22	4.18E-21	38.58777	up
GOMF_PO	0.595711	-0.17677	9.387673	1.21E-16	5.01E-16	26.57082	up
VECCHI_G/	-0.59544	0.047068	-28.2554	8.93E-61	1.13E-58	128.1255	down
HP_REDU(	-0.59542	0.123597	-8.27834	7.56E-14	2.56E-13	20.17502	down
HP_INCRE/	-0.59537	0.071463	-12.07	1.20E-23	8.19E-23	42.63548	down
GOBP_NE(	0.595298	-0.00317	12.09942	1.01E-23	6.89E-23	42.81353	up
MODULE_	-0.59518	-0.02028	-23.5166	2.72E-51	1.44E-49	106.2976	down
GOMF_ON	-0.59509	0.113601	-11.1602	2.97E-21	1.71E-20	37.1407	down
HP_NONC	-0.59491	-0.04891	-15.6357	6.77E-33	8.30E-32	63.91958	down
HP_AORTI	-0.59483	-0.15529	-11.3892	7.42E-22	4.46E-21	38.52243	down
HP_LOWEI	0.594793	-0.09156	10.73747	3.80E-20	2.02E-19	34.59687	up
REACTOM	0.594753	0.006995	25.25617	6.63E-55	4.93E-53	114.6155	up
GOMF_NC	-0.59461	0.01742	-8.58371	1.31E-14	4.71E-14	21.91082	down
GOBP_CD4	-0.59438	0.134686	-13.4098	3.68E-27	3.18E-26	50.71608	down
GOMF_OX	-0.5943	-0.0965	-28.4469	3.89E-61	5.05E-59	128.9544	down
GOBP_GLU	-0.59409	-0.0338	-18.8461	8.65E-41	1.79E-39	82.10211	down
GOBP_CAT	-0.59407	0.011687	-15.5716	9.84E-33	1.19E-31	63.54507	down
WP_COPPI	-0.59405	0.028956	-27.542	2.03E-59	2.25E-57	125.0039	down
chr4p11	-0.59401	0.0439	-11.1474	3.20E-21	1.84E-20	37.06393	down
BURTON_/	-0.59399	0.095845	-13.3888	4.18E-27	3.60E-26	50.58993	down
GOBP_DN/	0.593964	-0.02536	20.76733	2.97E-45	8.78E-44	92.38845	up
MODULE_	0.59383	0.011486	19.07824	2.44E-41	5.25E-40	83.36935	up
GOBP_NE(	0.593782	-0.05868	10.60628	8.38E-20	4.36E-19	33.80935	up
GOBP_REG	-0.59374	-0.09771	-10.8737	1.67E-20	9.12E-20	35.4155	down

MIR184	-0.59367	-0.02459	-15.3961	2.75E-32	3.24E-31	62.51793	down
GOBP_ON	-0.59365	0.038406	-15.5254	1.29E-32	1.55E-31	63.27523	down
GOBP_REG	-0.59343	0.127403	-14.2006	3.23E-29	3.16E-28	55.44933	down
REACTOM	-0.59327	-0.12822	-15.0804	1.76E-31	1.97E-30	60.66274	down
REACTOM	0.593238	0.026128	23.33908	6.48E-51	3.27E-49	105.4281	up
HP_NEOPL	0.593203	-0.00817	16.61602	2.33E-35	3.30E-34	69.59441	up
GOBP_SEQ	-0.59307	-0.00574	-16.8027	7.99E-36	1.17E-34	70.66339	down
MIR4520_5	-0.59305	-0.08279	-12.5441	6.82E-25	5.04E-24	45.50004	down
WP_ATM_S	0.592792	0.001953	20.60877	6.81E-45	1.96E-43	91.55717	up
VANTVEER	0.592771	-0.01399	21.89921	8.64E-48	3.18E-46	98.23011	up
GOBP_STE	-0.59272	-0.06289	-21.336	1.55E-46	5.06E-45	95.34341	down
GOBP_NEC	0.592663	-0.06294	12.65355	3.52E-25	2.65E-24	46.16107	up
KEGG_LINC	-0.59264	-0.03282	-18.9862	4.03E-41	8.55E-40	82.86735	down
GOBP_VAS	-0.59259	-0.03694	-24.2448	7.99E-53	4.84E-51	109.8241	down
GOBP_REG	0.59258	0.122864	9.524815	5.36E-17	2.28E-16	27.37661	up
HUMMERI	-0.59246	0.02842	-16.9918	2.72E-36	4.13E-35	71.74235	down
GOMF_CIL	-0.59237	0.07611	-12.6148	4.45E-25	3.33E-24	45.9273	down
HP_SKELET	-0.5923	-0.13216	-12.6541	3.51E-25	2.64E-24	46.16452	down
REACTOM	-0.5923	-0.04769	-26.7685	6.40E-58	6.25E-56	121.5554	down
GOBP_GLC	0.592285	-0.09636	7.897003	6.52E-13	2.06E-12	18.0396	up
GOBP_HEA	-0.59227	0.050629	-14.7469	1.26E-30	1.35E-29	58.69378	down
GOCC_MIT	0.592222	-0.01217	25.21278	8.12E-55	5.97E-53	114.4124	up
GOBP_DN/	0.591776	-0.00241	22.97713	3.86E-50	1.82E-48	103.6431	up
HP_TYPE_1	0.591673	0.033039	11.01365	7.19E-21	4.02E-20	36.25796	up
HP_RENAL	-0.59157	-0.10028	-20.518	1.10E-44	3.08E-43	91.07985	down
GOBP_URI	-0.59152	0.007178	-10.7171	4.30E-20	2.28E-19	34.47447	down
VECCHI_G/	0.591163	-0.03911	20.97168	1.02E-45	3.14E-44	93.4551	up
GOBP_CEL	-0.59103	-0.04475	-28.8153	7.98E-62	1.12E-59	130.5373	down
GOBP_INT	0.591012	-0.04462	16.39825	8.13E-35	1.12E-33	68.3425	up
HP_SUBEP	-0.59101	-0.15294	-10.2206	8.50E-19	4.11E-18	31.50163	down
HP_PULMC	-0.59098	0.027241	-17.9913	9.73E-39	1.74E-37	77.37717	down
QI_PBMC_	-0.5907	0.08771	-9.72704	1.62E-17	7.15E-17	28.5696	down
GOBP_PRC	-0.5907	-0.05799	-11.9952	1.89E-23	1.27E-22	42.18341	down
GOMF_TEL	0.590658	-0.12063	12.18212	6.10E-24	4.24E-23	43.31337	up
GOMF_RN	0.590658	-0.12063	12.18212	6.10E-24	4.24E-23	43.31337	up
GOMF_NA	-0.5906	0.183798	-11.4741	4.44E-22	2.71E-21	39.03511	down
GOBP_HIS	0.590574	-0.0102	15.13237	1.29E-31	1.46E-30	60.96897	up
HP_ABNO	-0.5905	-0.10307	-19.4798	2.77E-42	6.41E-41	85.54597	down
GOCC_NA	-0.59019	-0.21995	-10.9712	9.28E-21	5.15E-20	36.00255	down
GOBP_CEL	-0.59009	-0.10072	-20.291	3.64E-44	9.79E-43	89.88136	down
GOMF_SEL	-0.5897	-0.1444	-10.6099	8.20E-20	4.27E-19	33.83087	down
MANN_RE	0.589697	0.054897	11.34225	9.86E-22	5.86E-21	38.23903	up
NADLER_C	-0.58948	-0.09243	-22.4974	4.21E-49	1.78E-47	101.2519	down
MODULE_	-0.58937	0.070358	-15.2857	5.25E-32	6.09E-31	61.87009	down
GOBP_R_L	0.589257	0.008048	11.97799	2.10E-23	1.40E-22	42.07958	up
LANDIS_EF	-0.58917	-0.03178	-26.474	2.42E-57	2.27E-55	120.2247	down
GOBP_REG	0.58912	-0.08562	11.7174	1.02E-22	6.47E-22	40.5046	up
chr4q22	-0.58898	0.075125	-14.2737	2.09E-29	2.07E-28	55.8846	down
GOBP_TEL	0.588819	-0.09313	13.92765	1.65E-28	1.55E-27	53.81991	up
LANDIS_BF	-0.58876	-0.00099	-19.0962	2.21E-41	4.78E-40	83.46739	down
MORF_SM	0.588756	-0.00181	12.77053	1.74E-25	1.34E-24	46.86703	up
GOCC_DN	0.588644	-0.16028	13.29336	7.42E-27	6.28E-26	50.01636	up
MIR4537	0.588594	-0.02924	9.517193	5.61E-17	2.38E-16	27.33176	up
GOBP_FRU	-0.58857	-0.08649	-15.1332	1.29E-31	1.46E-30	60.97386	down
MODULE_	-0.58846	-0.06358	-17.1531	1.09E-36	1.69E-35	72.65944	down
HP_BROAD	-0.5884	-0.02868	-12.7793	1.65E-25	1.27E-24	46.91974	down
GOMF_LOI	-0.58808	-0.04208	-18.0898	5.62E-39	1.02E-37	77.92626	down
GOMF_AD	-0.58794	0.020575	-13.8025	3.49E-28	3.20E-27	53.07125	down

REACTOM	-0.58794	0.020575	-13.8025	3.49E-28	3.20E-27	53.07125	down
GUILLAUM	-0.58784	-0.01484	-18.8929	6.70E-41	1.40E-39	82.35789	down
REACTOM	0.587833	0.02379	16.6341	2.10E-35	2.99E-34	69.69814	up
GOBP_SER	-0.58781	0.082962	-16.0453	6.24E-34	8.13E-33	66.30286	down
HP_POST_I	-0.5878	-0.08453	-12.888	8.54E-26	6.72E-25	47.57529	down
HP_ABNOI	-0.58776	0.058017	-10.829	2.19E-20	1.18E-19	35.1471	down
GOCC_SPII	0.587678	-0.02947	20.75031	3.24E-45	9.56E-44	92.29936	up
GOBP_REG	-0.58754	0.122808	-10.6486	6.50E-20	3.41E-19	34.06314	down
AIZARANI_	-0.58752	-0.04576	-23.6531	1.40E-51	7.62E-50	106.9639	down
WP_SARSC	-0.58744	-0.03619	-12.8148	1.33E-25	1.03E-24	47.13401	down
REACTOM	-0.58742	0.06494	-14.7753	1.06E-30	1.14E-29	58.86184	down
GOBP_MIT	0.5874	-0.03431	23.34219	6.38E-51	3.23E-49	105.4434	up
BRACHAT_	-0.58738	-0.02878	-16.8134	7.51E-36	1.11E-34	70.72468	down
REACTOM	0.587027	-0.10898	10.44974	2.15E-19	1.08E-18	32.87132	up
MARSON_	0.586642	-0.06005	17.8877	1.73E-38	3.05E-37	76.79874	up
GOBP_REG	-0.5865	-0.01445	-22.3548	8.62E-49	3.52E-47	100.5356	down
GOBP_PLA	-0.58647	0.107566	-9.83908	8.30E-18	3.75E-17	29.23277	down
MODULE_!	-0.58617	0.102636	-11.1995	2.34E-21	1.35E-20	37.37801	down
BIOCARTA	-0.58602	0.009984	-9.95155	4.25E-18	1.96E-17	29.90003	down
REACTOM	0.586014	-0.11311	10.55509	1.14E-19	5.88E-19	33.50242	up
GOBP_MET	0.585642	-0.06148	19.84449	3.91E-43	9.65E-42	87.50545	up
SCHMAHL_	-0.58557	0.094674	-12.6104	4.57E-25	3.41E-24	45.90079	down
HP_RAGGE	-0.58547	-0.10251	-14.665	2.04E-30	2.16E-29	58.20878	down
HP_CLITOI	0.585293	-0.018	21.38179	1.22E-46	4.05E-45	95.57983	up
HP_PROLII	-0.58522	0.025892	-13.3962	4.00E-27	3.44E-26	50.63466	down
WP_ELECT	-0.58515	-0.18813	-11.4129	6.43E-22	3.88E-21	38.66536	down
WP_METAI	-0.58499	-0.05026	-29.8332	1.08E-63	1.83E-61	134.8367	down
GOBP_MA1	-0.58472	-0.03628	-8.88375	2.31E-15	8.75E-15	23.6359	down
GOBP_POS	-0.58464	0.007437	-25.9084	3.21E-56	2.70E-54	117.6411	down
MCGOWA	0.584615	-0.10312	9.372065	1.32E-16	5.48E-16	26.47929	up
HP_HYPER	-0.58459	0.028137	-17.2091	7.91E-37	1.24E-35	72.97713	down
REACTOM	-0.58458	-0.04387	-25.6114	1.27E-55	1.02E-53	116.2697	down
REACTOM	-0.58445	0.121491	-12.5651	6.01E-25	4.45E-24	45.62698	down
GOBP_POS	0.584432	-0.04742	14.6079	2.87E-30	3.00E-29	57.87055	up
GOBP_REG	0.584326	-0.05021	12.74021	2.09E-25	1.60E-24	46.68405	up
GOBP_THF	-0.5843	-0.06729	-11.8726	3.97E-23	2.61E-22	41.44247	down
YU_BAP1_1	0.58422	-0.04725	15.18012	9.77E-32	1.11E-30	61.2499	up
GOBP_L_A	-0.58403	0.052206	-11.6984	1.14E-22	7.24E-22	40.3899	down
HP_OVERF	0.584	0.016723	13.40633	3.76E-27	3.25E-26	50.69538	up
GOMF_CD	-0.58393	0.125106	-9.48244	6.89E-17	2.91E-16	27.12733	down
GOBP_T_H	-0.58391	0.225258	-9.91609	5.25E-18	2.40E-17	29.68954	down
chr8p22	-0.58388	0.153173	-11.8188	5.50E-23	3.58E-22	41.11757	down
GOMF_HIS	0.583832	-0.01026	11.98101	2.06E-23	1.38E-22	42.09779	up
HP_HYPER	-0.58377	0.044207	-12.477	1.02E-24	7.48E-24	45.09488	down
GOBP_POS	0.583684	-0.07964	14.24043	2.55E-29	2.50E-28	55.68647	up
BIOCARTA	0.583649	-0.13416	11.38749	7.50E-22	4.50E-21	38.51204	up
REACTOM	0.583562	-0.10535	10.85323	1.89E-20	1.03E-19	35.29257	up
GOMF_UBI	-0.58348	-0.21161	-9.54382	4.79E-17	2.05E-16	27.48846	down
HP_PERITC	-0.58332	0.004455	-13.0263	3.71E-26	3.00E-25	48.40874	down
WP_OXIDA	-0.58318	-0.20899	-11.5725	2.45E-22	1.52E-21	39.62944	down
GOBP_REG	-0.58304	-0.08203	-25.3797	3.72E-55	2.85E-53	115.1922	down
HP_APLAS	0.58294	0.049231	16.76134	1.01E-35	1.48E-34	70.42697	up
BIOCARTA	0.58286	-0.07558	13.42406	3.38E-27	2.93E-26	50.8019	up
RUAN_RES	-0.58268	0.007898	-13.5268	1.82E-27	1.61E-26	51.4185	down
REACTOM	0.582681	-0.03897	19.36628	5.11E-42	1.17E-40	84.93281	up
MODULE_2	-0.58262	0.149224	-12.8347	1.18E-25	9.18E-25	47.25417	down
GOBP_NEC	-0.58262	-0.01999	-18.8878	6.89E-41	1.44E-39	82.3301	down
GOBP_REG	-0.58236	0.082061	-12.8089	1.38E-25	1.07E-24	47.09841	down

GOMF_SH(	-0.58234	-0.02903	-12.2105	5.14E-24	3.59E-23	43.48493	down
REACTOM	-0.58195	-0.04017	-18.0416	7.35E-39	1.32E-37	77.65773	down
ZNF774_T/	0.581915	-0.2277	11.75371	8.17E-23	5.24E-22	40.72401	up
HP_ENCEP	-0.58187	0.005197	-15.6525	6.13E-33	7.54E-32	64.01768	down
HP_SPASTI	-0.58179	-0.06309	-14.029	9.00E-29	8.58E-28	54.42547	down
GOBP_REG	-0.5817	0.154511	-12.0133	1.70E-23	1.14E-22	42.29281	down
HP_SCLER(	0.581434	-0.00137	10.87985	1.61E-20	8.78E-20	35.45266	up
HP_ABNOI	-0.5814	0.128511	-12.6746	3.10E-25	2.35E-24	46.28815	down
HP_THOR/	0.581396	-0.03246	11.76669	7.55E-23	4.85E-22	40.80246	up
REACTOM	0.581356	-0.06421	16.29116	1.51E-34	2.03E-33	67.72494	up
GOMF_FA	-0.58135	-0.01746	-20.3013	3.44E-44	9.29E-43	89.9363	down
REACTOM	0.581311	-0.1134	10.68189	5.32E-20	2.80E-19	34.2631	up
GOMF_CA	-0.5813	-0.08874	-13.1793	1.47E-26	1.23E-25	49.33035	down
GOMF_DN	0.581172	0.052542	11.51681	3.43E-22	2.11E-21	39.29284	up
GOBP_RES	-0.58111	0.250418	-7.48905	6.27E-12	1.84E-11	15.80022	down
GOBP_NEU	-0.58108	-0.02762	-17.028	2.21E-36	3.38E-35	71.9483	down
REACTOM	-0.58085	0.000923	-21.6782	2.67E-47	9.40E-46	97.10206	down
GOBP_COI	-0.5808	-0.00684	-16.1141	4.19E-34	5.51E-33	66.70123	down
GOBP_PH(	-0.58077	-0.10296	-13.9985	1.08E-28	1.02E-27	54.24348	down
GOBP_MIT	0.580679	-0.05118	19.55232	1.87E-42	4.39E-41	85.93707	up
GOBP_NE(	-0.58059	0.154295	-8.34162	5.27E-14	1.81E-13	20.53292	down
HP_NYSTA	0.580534	-0.00965	11.92872	2.83E-23	1.88E-22	41.78174	up
GOBP_NU(	0.58027	0.000464	21.46076	8.14E-47	2.75E-45	95.98655	up
GSE12845_	0.580173	-0.01501	19.38765	4.55E-42	1.04E-40	85.04839	up
BIOCARTA	-0.58013	0.009904	-13.3716	4.63E-27	3.97E-26	50.48693	down
GOMF_NU	0.579994	0.003042	15.62347	7.27E-33	8.89E-32	63.84819	up
GOBP_TRI(	-0.57989	-0.04452	-19.445	3.34E-42	7.71E-41	85.35831	down
WHITFIELD	0.579824	-0.02124	22.84344	7.49E-50	3.44E-48	102.9796	up
GOBP_CEL	-0.57978	-0.04974	-10.9328	1.17E-20	6.45E-20	35.77136	down
BOYAULT_	-0.5797	0.161422	-11.7722	7.30E-23	4.70E-22	40.83604	down
GOBP_PRC	-0.57969	-0.12275	-12.3567	2.12E-24	1.52E-23	44.36826	down
GSE35543_	0.579627	-0.04959	18.02829	7.91E-39	1.42E-37	77.58362	up
GOBP_SIGI	0.579544	-0.06031	14.74275	1.29E-30	1.38E-29	58.66938	up
REACTOM	0.579499	-0.00782	23.49275	3.05E-51	1.60E-49	106.1811	up
HP_CHROI	0.579186	-0.01335	9.827239	8.91E-18	4.01E-17	29.16263	up
WP_G1_TC	0.57908	-0.0323	18.332	1.47E-39	2.79E-38	79.27109	up
REACTOM	-0.57903	0.005762	-13.0517	3.18E-26	2.59E-25	48.56189	down
GOBP_POS	-0.57882	0.067661	-11.1169	3.85E-21	2.20E-20	36.88005	down
HP_EPISOI	-0.57877	0.034466	-13.4006	3.89E-27	3.35E-26	50.66108	down
GOBP_REG	0.578684	-0.02829	10.50327	1.56E-19	7.94E-19	33.19189	up
GOMF_OX	-0.57864	-0.10504	-18.9369	5.27E-41	1.11E-39	82.59833	down
GOMF_MC	-0.57849	-0.02852	-24.3445	4.96E-53	3.09E-51	110.3018	down
GOBP_G0_	0.578458	0.001839	19.94554	2.28E-43	5.76E-42	88.04536	up
GOBP_AM	-0.57843	0.024274	-18.7824	1.23E-40	2.51E-39	81.75311	down
HP_EPISPA	0.578415	-0.008	19.19576	1.29E-41	2.84E-40	84.0085	up
REACTOM	0.578293	-0.03693	20.0036	1.67E-43	4.28E-42	88.35504	up
GOBP_CEL	-0.57827	-0.04373	-27.1155	1.35E-58	1.38E-56	123.1107	down
BUSSLINGI	-0.57814	-0.10886	-17.4427	2.11E-37	3.44E-36	74.29902	down
HP_DISTAI	-0.57803	0.162088	-11.3964	7.10E-22	4.27E-21	38.56584	down
HARALAM	-0.57795	0.069233	-12.011	1.72E-23	1.16E-22	42.27904	down
GOCC_CEL	-0.57791	0.02835	-14.6239	2.61E-30	2.73E-29	57.96536	down
FAN_EMBI	-0.57772	0.093042	-15.3486	3.63E-32	4.25E-31	62.23968	down
HP_VENOU	-0.57755	0.01589	-23.541	2.41E-51	1.28E-49	106.4169	down
GOBP_MIT	0.577532	-0.02909	23.47257	3.37E-51	1.76E-49	106.0824	up
GOCC_CEI	0.577518	0.101803	13.31176	6.64E-27	5.64E-26	50.12702	up
GOCC_HEI	-0.57749	0.064984	-11.2496	1.73E-21	1.01E-20	37.67998	down
GOBP_MO	-0.57748	0.030527	-11.3246	1.10E-21	6.50E-21	38.13279	down
MODULE_/	0.577394	0.061203	19.09678	2.20E-41	4.77E-40	83.47033	up

GOBP_ACY	-0.57738	-0.03066	-10.2512	7.07E-19	3.45E-18	31.6846	down
GOBP_INSI	-0.57734	0.021485	-10.8115	2.43E-20	1.31E-19	35.04169	down
GOBP_REG	0.577292	0.032872	16.959	3.27E-36	4.95E-35	71.55564	up
GOCC_NU	0.57711	-0.06443	14.15713	4.19E-29	4.06E-28	55.19002	up
OHGUCHI_	-0.57703	0.011016	-24.6496	1.16E-53	7.65E-52	111.7569	down
GOBP_NEU	-0.57692	0.174444	-13.318	6.40E-27	5.44E-26	50.16473	down
BIOCARTA	-0.57679	0.098221	-14.6541	2.18E-30	2.29E-29	58.14453	down
WP_BENZC	-0.57662	-0.19561	-10.5074	1.52E-19	7.76E-19	33.21639	down
GOMF_OX	-0.57659	-0.22856	-12.0998	1.00E-23	6.88E-23	42.81578	down
HP_HYPOF	0.576575	0.004214	10.75042	3.52E-20	1.87E-19	34.67463	up
TRAVAGLII	-0.57652	0.093904	-11.3219	1.11E-21	6.60E-21	38.11639	down
CHEMELLC	-0.57644	0.062008	-11.1715	2.77E-21	1.60E-20	37.20897	down
BOYAULT_	-0.57635	0.090649	-13.7527	4.70E-28	4.28E-27	52.77319	down
MODULE_4	-0.57626	-0.09665	-16.5493	3.41E-35	4.78E-34	69.21131	down
GOBP_PRC	0.576253	-0.1016	9.297445	2.05E-16	8.39E-16	26.04222	up
GOBP_PRC	-0.57608	-0.13768	-12.5577	6.28E-25	4.64E-24	45.58253	down
GOBP_PAF	-0.57589	-0.01332	-9.28611	2.19E-16	8.95E-16	25.9759	down
REACTOM	0.575869	0.023015	24.30538	5.98E-53	3.67E-51	110.1147	up
GOBP_KILL	-0.57579	-0.03345	-18.6108	3.15E-40	6.24E-39	80.81001	down
GOBP_OXY	-0.57564	0.10476	-14.9704	3.36E-31	3.71E-30	60.01451	down
GOCC_SPII	0.575613	-0.09691	15.53471	1.22E-32	1.47E-31	63.32949	up
REACTOM	0.575581	0.008624	14.7056	1.61E-30	1.71E-29	58.44946	up
GOCC_DN.	0.575525	-0.07674	13.27964	8.06E-27	6.80E-26	49.93386	up
GOBP_AM	0.575182	-0.02593	11.00297	7.67E-21	4.28E-20	36.19364	up
GOBP_GLU	0.575182	-0.02593	11.00297	7.67E-21	4.28E-20	36.19364	up
GOBP_POS	-0.57512	-0.01161	-13.9552	1.40E-28	1.32E-27	53.98449	down
GOBP_REG	-0.57512	0.067544	-18.328	1.50E-39	2.85E-38	79.24918	down
GOBP_TET	-0.57503	-0.0732	-21.4004	1.11E-46	3.70E-45	95.67569	down
GOMF_EST	-0.57483	-0.00319	-10.9449	1.09E-20	6.01E-20	35.84409	down
HP_ALVEO	-0.57479	0.093569	-10.7208	4.21E-20	2.23E-19	34.49667	down
GOBP_LON	-0.57463	-0.04999	-11.5117	3.54E-22	2.17E-21	39.26175	down
REACTOM	-0.57462	-0.11258	-11.1789	2.65E-21	1.53E-20	37.25371	down
PID_HNF3/	-0.57456	0.014255	-18.6156	3.06E-40	6.08E-39	80.83667	down
HP_EPISOI	0.574518	-0.17047	10.83389	2.13E-20	1.15E-19	35.17628	up
GOBP_MIS	0.574073	-0.06893	14.64652	2.28E-30	2.40E-29	58.09945	up
GOBP_PRC	0.573998	-0.02397	17.44339	2.10E-37	3.43E-36	74.30284	up
MODULE_:	-0.57395	-0.03113	-22.7922	9.67E-50	4.40E-48	102.7248	down
GOBP_REG	-0.57387	-0.03084	-12.4862	9.69E-25	7.08E-24	45.15035	down
GSE14415_	0.573696	-0.08074	14.41129	9.22E-30	9.29E-29	56.70326	up
GAO_SMA	-0.57365	-0.03802	-18.7398	1.55E-40	3.15E-39	81.51897	down
GOCC_CHI	0.573654	-0.03634	20.57235	8.25E-45	2.34E-43	91.36576	up
GOBP_NEC	-0.57356	0.195173	-11.6133	1.91E-22	1.19E-21	39.87572	down
REACTOM	0.573542	0.027223	10.38357	3.20E-19	1.59E-18	32.4754	up
GOCC_HIS	0.573534	0.003511	20.0495	1.31E-43	3.38E-42	88.59955	up
GOBP_MEI	0.573409	-0.04771	11.363	8.69E-22	5.19E-21	38.36423	up
GOBP_STE	-0.57314	-0.06434	-25.7558	6.50E-56	5.33E-54	116.9378	down
GOMF_MF	-0.57285	0.125808	-7.52061	5.27E-12	1.55E-11	15.9716	down
GOBP_PER	-0.57285	-0.09279	-15.8119	2.42E-33	3.05E-32	64.94673	down
REACTOM	-0.57281	-0.07728	-13.3531	5.18E-27	4.43E-26	50.37545	down
GOBP_ORC	-0.57245	-0.0472	-24.9358	2.99E-54	2.08E-52	113.1109	down
GOBP_SIST	0.572443	0.022798	18.70792	1.85E-40	3.71E-39	81.34418	up
DESCARTE	-0.5724	0.011458	-25.769	6.11E-56	5.03E-54	116.9987	down
HP_ABNOI	-0.57231	-0.07804	-16.7202	1.28E-35	1.85E-34	70.19133	down
GOBP_CHF	0.572229	0.007409	23.10441	2.06E-50	9.92E-49	104.2727	up
HP_HYPER	-0.5721	-0.04966	-17.4611	1.90E-37	3.11E-36	74.40284	down
MODULE_S	0.572007	0.003042	21.74374	1.91E-47	6.83E-46	97.43738	up
GSE36476_	0.57179	-0.0718	14.8958	5.22E-31	5.72E-30	59.57428	up
GOBP_DEN	-0.57166	0.06601	-12.6102	4.58E-25	3.41E-24	45.89944	down

GOMF_RN	0.571453	-0.01399	10.026	2.72E-18	1.27E-17	30.34256	up
KEGG_TAU	-0.57126	0.072448	-15.9177	1.31E-33	1.67E-32	65.56218	down
GOBP_REG	-0.57108	-0.06101	-18.8799	7.19E-41	1.50E-39	82.28672	down
MITSIADES	0.571043	0.018854	19.04623	2.90E-41	6.20E-40	83.19499	up
GOMF_GLI	-0.57092	-0.09096	-16.5347	3.71E-35	5.18E-34	69.12747	down
GRAHAM_	0.570636	-0.0426	14.36122	1.24E-29	1.24E-28	56.40549	up
DAUER_ST	-0.57058	0.06987	-14.1879	3.49E-29	3.40E-28	55.37373	down
HP_REDUC	-0.57057	0.05049	-9.55951	4.37E-17	1.87E-16	27.58088	down
GOBP_PRC	0.570511	0.034628	15.73827	3.72E-33	4.63E-32	64.51794	up
HP_DECRE	-0.57043	-0.03681	-14.8027	9.05E-31	9.76E-30	59.02401	down
REACTOM	0.570098	-0.09281	14.57926	3.40E-30	3.53E-29	57.7007	up
REACTOM	0.570027	0.021753	13.16304	1.63E-26	1.35E-25	49.2324	up
GOBP_POS	-0.56995	0.051973	-12.6546	3.50E-25	2.64E-24	46.16773	down
HP_ABNOI	-0.56991	-0.08972	-15.5466	1.14E-32	1.37E-31	63.39926	down
GOBP_ORC	-0.56988	-0.06996	-25.4212	3.07E-55	2.37E-53	115.3855	down
GOCC_NU	0.569818	-0.025	19.2053	1.22E-41	2.71E-40	84.06031	up
SMID_BRE	-0.56971	0.279954	-8.87595	2.42E-15	9.14E-15	23.59083	down
GOBP_NEC	-0.56966	0.08183	-13.1785	1.48E-26	1.23E-25	49.32526	down
GOBP_REG	-0.56933	0.053526	-11.9378	2.68E-23	1.78E-22	41.8365	down
REACTOM	-0.56933	-0.00497	-16.7843	8.87E-36	1.30E-34	70.55825	down
BIOCARTA	-0.5693	-0.05102	-11.2455	1.77E-21	1.03E-20	37.65515	down
GOMF_HEI	-0.56926	0.083667	-8.5583	1.52E-14	5.42E-14	21.76557	down
DELYS_THY	-0.56903	0.034313	-24.4458	3.05E-53	1.95E-51	110.7863	down
GNF2_IL2R	-0.56892	0.102734	-9.47514	7.19E-17	3.04E-16	27.08444	down
GSE14415_	0.56885	-0.05201	15.33027	4.04E-32	4.72E-31	62.13193	up
REACTOM	-0.56875	-0.06904	-23.1978	1.30E-50	6.39E-49	104.7331	down
HMCES_TA	-0.56855	-0.05228	-11.6664	1.39E-22	8.73E-22	40.1964	down
GOMF_PEI	-0.56853	-0.13396	-9.17012	4.34E-16	1.73E-15	25.29846	down
IM_SREBF1	-0.56849	-0.03065	-12.1537	7.25E-24	5.02E-23	43.1413	down
GOBP_SEN	0.568477	0.022326	11.03411	6.35E-21	3.57E-20	36.38118	up
GOCC_CHA	0.568468	-0.09126	8.774081	4.37E-15	1.62E-14	23.00324	up
GOBP_PHA	-0.56843	0.093182	-14.2575	2.30E-29	2.27E-28	55.78805	down
REACTOM	-0.56837	-0.07121	-14.5357	4.40E-30	4.53E-29	57.44251	down
GOMF_OX	-0.56807	-0.15102	-14.0229	9.33E-29	8.89E-28	54.38929	down
HP_MACU	-0.56798	-0.06582	-13.2176	1.17E-26	9.81E-26	49.56051	down
NAKAMUR	-0.5679	0.074069	-21.2056	3.03E-46	9.73E-45	94.66973	down
GOMF_MI(	0.56785	0.023566	26.76815	6.41E-58	6.25E-56	121.5537	up
GOCC_PEF	-0.56783	0.011233	-8.90316	2.06E-15	7.85E-15	23.74815	down
GOBP_NEC	0.567802	0.051487	12.30769	2.85E-24	2.03E-23	44.07224	up
WP_VITAM	-0.56775	-0.04707	-22.455	5.21E-49	2.16E-47	101.0393	down
GOBP_PRC	-0.56771	-0.15886	-12.9991	4.37E-26	3.52E-25	48.2453	down
BIOCARTA	-0.56764	0.003378	-16.0691	5.44E-34	7.10E-33	66.44069	down
GOBP_NEC	-0.56753	0.17877	-10.2815	5.90E-19	2.89E-18	31.86508	down
HP_ROTAF	-0.56746	-0.04147	-16.1583	3.25E-34	4.29E-33	66.95726	down
BLANCO_N	-0.56745	-0.0278	-20.1174	9.12E-44	2.38E-42	88.96092	down
HARALAM	-0.56734	0.113958	-11.6615	1.43E-22	8.99E-22	40.16687	down
GOBP_TEL	0.567172	-0.04252	19.92008	2.61E-43	6.56E-42	87.90947	up
GOBP_POS	-0.56712	-0.00891	-17.9031	1.59E-38	2.81E-37	76.88509	down
GOBP_CEL	-0.56708	-0.05452	-18.9115	6.05E-41	1.27E-39	82.45969	down
REACTOM	0.567056	0.008694	16.1428	3.55E-34	4.68E-33	66.86751	up
DESCARTE	0.566951	0.005279	13.20358	1.27E-26	1.07E-25	49.47631	up
LUI_THYRC	-0.56689	-0.04019	-16.8914	4.81E-36	7.19E-35	71.16992	down
GOBP_VEN	-0.56687	0.127608	-11.5953	2.13E-22	1.33E-21	39.76662	down
BUSSLINGI	-0.56684	-0.11413	-17.7652	3.44E-38	5.92E-37	76.11314	down
HOFFMAN	-0.56677	-0.00666	-14.666	2.03E-30	2.15E-29	58.21464	down
REACTOM	0.566694	0.017373	15.06829	1.89E-31	2.11E-30	60.59169	up
NIKOLSKY_	0.566284	-0.2317	10.89393	1.48E-20	8.09E-20	35.5374	up
HP_BLURR	-0.56625	0.016961	-16.4093	7.63E-35	1.05E-33	68.40625	down

GOBP_REG	0.566217	-0.03754	24.70025	9.10E-54	6.06E-52	111.9971	up
GOMF_HYI	-0.5662	-0.0036	-13.3842	4.30E-27	3.69E-26	50.56261	down
GOBP_UNS	-0.56619	-0.04985	-25.9182	3.07E-56	2.59E-54	117.6863	down
BASAKI_YE	0.566168	-0.02526	19.22527	1.10E-41	2.44E-40	84.16869	up
GOBP_REG	0.565753	-0.11727	10.79222	2.73E-20	1.47E-19	34.92581	up
HU_FETAL_	0.565593	0.018237	20.09967	1.00E-43	2.61E-42	88.86647	up
GOMF_CA	-0.5655	-0.01577	-28.5575	2.42E-61	3.20E-59	129.4311	down
HP_ABNOI	0.565464	0.003339	12.90409	7.75E-26	6.12E-25	47.6725	up
MORF_MS	0.565371	-0.06228	12.1542	7.22E-24	5.01E-23	43.14461	up
HP_LARGE_	-0.56534	-0.00673	-14.601	2.99E-30	3.12E-29	57.82967	down
GOCC_MLI	0.565327	-0.04454	16.35459	1.05E-34	1.43E-33	68.09088	up
GOBP_TOI	0.565265	0.058393	13.58511	1.28E-27	1.14E-26	51.76857	up
GOBP_CEL	0.565185	-0.03294	22.00488	5.05E-48	1.92E-46	98.76721	up
GOBP_PRC	-0.56509	-0.10865	-14.0577	7.58E-29	7.25E-28	54.59707	down
GOMF_OX	-0.56501	-0.03374	-23.1356	1.76E-50	8.56E-49	104.4267	down
LOPEZ_ME	0.564985	0.060313	13.96995	1.28E-28	1.21E-27	54.07272	up
GOMF_HIS	0.564879	-0.04836	15.477	1.71E-32	2.04E-31	62.99182	up
RUAN_RES	-0.56472	-0.09328	-19.2974	7.42E-42	1.68E-40	84.55973	down
REACTOM	0.564701	-0.09992	13.97607	1.23E-28	1.17E-27	54.10929	up
FAN_EMBF	0.564668	-0.01359	10.44931	2.15E-19	1.09E-18	32.86875	up
GOBP_CEL	-0.56461	-0.01227	-33.8394	1.26E-70	4.84E-68	150.7623	down
GOBP_POS	-0.56459	-0.05649	-15.3508	3.58E-32	4.19E-31	62.25259	down
GOMF_EN(	-0.56459	-0.15864	-10.0651	2.16E-18	1.02E-17	30.57529	down
GNF2_CAR	-0.56458	0.124137	-9.37264	1.32E-16	5.46E-16	26.48269	down
HP_ABNOI	-0.56448	0.026079	-18.0703	6.26E-39	1.14E-37	77.81769	down
HOLLEMAI	0.56445	-0.01634	11.57848	2.36E-22	1.47E-21	39.66532	up
MORF_DN	0.56433	-0.05344	14.25356	2.36E-29	2.32E-28	55.76471	up
HP_ABNOI	-0.56431	-0.04602	-26.1474	1.07E-56	9.46E-55	118.7373	down
GOBP_AUT	-0.56431	0.080053	-8.18757	1.27E-13	4.21E-13	19.66334	down
STEGER_AI	-0.56421	-0.00364	-15.5923	8.72E-33	1.06E-31	63.66613	down
HP_ABNOI	-0.56409	-0.14165	-13.3791	4.43E-27	3.80E-26	50.53186	down
REACTOM	0.564042	-0.00996	14.01352	9.87E-29	9.38E-28	54.333	up
HP_APLAS	0.563861	-0.01594	8.734	5.51E-15	2.03E-14	22.77261	up
BIOCARTA	-0.56372	0.027444	-18.8104	1.05E-40	2.16E-39	81.90617	down
HP_ABNOI	0.563696	0.011184	10.71476	4.36E-20	2.31E-19	34.46042	up
GOBP_OVI	-0.56368	0.116418	-14.6054	2.91E-30	3.04E-29	57.85542	down
GSE24634_	0.563569	-0.05285	16.63265	2.11E-35	3.01E-34	69.68977	up
HP_PULM(	-0.56345	0.144348	-9.16859	4.38E-16	1.74E-15	25.28956	down
GOMF_FAT	-0.56326	-0.02961	-18.8472	8.60E-41	1.78E-39	82.1078	down
HP_EPISOI	-0.56325	-0.02801	-17.3118	4.42E-37	7.05E-36	73.55926	down
HP_THICK_	0.563189	-0.06368	15.03586	2.28E-31	2.55E-30	60.40061	up
GCM_RAD	0.563119	0.011961	12.53149	7.36E-25	5.42E-24	45.42416	up
HP_MILD_I	-0.56301	0.016246	-16.5921	2.67E-35	3.76E-34	69.45715	down
REACTOM	0.562894	0.068256	8.157247	1.50E-13	4.97E-13	19.49284	up
GOBP_TOX	-0.56274	0.016132	-17.4771	1.74E-37	2.85E-36	74.49297	down
GNF2_CD3	-0.56267	0.126207	-9.819	9.36E-18	4.21E-17	29.11385	down
GOBP_NU(	-0.56248	-0.08495	-18.5814	3.70E-40	7.29E-39	80.64848	down
TOYOTA_T	0.56247	0.000422	24.32466	5.45E-53	3.38E-51	110.207	up
GOMF_NEI	0.562163	-0.06256	8.670998	7.94E-15	2.90E-14	22.41076	up
GOBP_SPE	-0.56216	-0.16075	-10.4143	2.66E-19	1.33E-18	32.65935	down
GOBP_CHI	0.56213	-0.04788	20.30382	3.40E-44	9.19E-43	89.94936	up
VANHARA	-0.56206	0.01867	-20.1439	7.93E-44	2.08E-42	89.10131	down
GOBP_REG	-0.56199	-0.03266	-22.3794	7.62E-49	3.12E-47	100.6596	down
HP_ABNOI	-0.56191	-0.06323	-24.3017	6.08E-53	3.72E-51	110.0972	down
GOBP_NIT	-0.56189	0.153252	-11.8781	3.84E-23	2.53E-22	41.47574	down
MIKHAYLC	0.561879	-0.03091	10.90238	1.41E-20	7.71E-20	35.58825	up
HP_WIDEL	0.561712	0.123232	11.65692	1.47E-22	9.23E-22	40.13917	up
GOBP_TER	-0.56146	-0.04699	-22.4954	4.26E-49	1.79E-47	101.2422	down

HP_FETAL_	-0.56138	-0.18386	-13.459	2.74E-27	2.39E-26	51.01146	down
HP_LACTIC	-0.56129	-0.1373	-14.163	4.04E-29	3.93E-28	55.22512	down
REACTOM	-0.56128	-0.12965	-13.4008	3.89E-27	3.35E-26	50.66218	down
HP_PROGF	-0.56126	-0.08612	-14.4184	8.84E-30	8.94E-29	56.74556	down
GOCC_CIA	0.561226	-0.0363	6.327282	2.94E-09	7.00E-09	9.738334	up
GOMF_OX	-0.56119	-0.0589	-28.7676	9.79E-62	1.36E-59	130.3333	down
GOBP_COI	-0.56117	-0.07593	-12.1711	6.52E-24	4.53E-23	43.24693	down
GOMF_BIL	-0.56117	-0.08385	-11.1436	3.28E-21	1.88E-20	37.04066	down
HP_TRIAN	0.561139	-0.01442	19.24038	1.01E-41	2.25E-40	84.25073	up
GOMF_OX	-0.56098	0.094302	-13.868	2.36E-28	2.19E-27	53.46341	down
CHANG_P(	-0.56063	0.126441	-11.9532	2.44E-23	1.62E-22	41.92945	down
GOBP_NE(	0.560593	0.014738	20.87616	1.68E-45	5.10E-44	92.95721	up
GOBP_PLA	0.560549	0.036869	12.66117	3.36E-25	2.54E-24	46.20709	up
GOBP_B_C	0.560301	0.071108	11.38446	7.64E-22	4.58E-21	38.49377	up
ABE_VEGF	-0.56023	0.096105	-17.5687	1.04E-37	1.73E-36	75.00918	down
HP_ACUTE	0.560191	-0.02214	20.11145	9.42E-44	2.46E-42	88.9291	up
GOBP_AEF	-0.56012	-0.15082	-13.5982	1.19E-27	1.06E-26	51.84723	down
GOBP_DIS	-0.56005	0.076217	-10.46	2.02E-19	1.02E-18	32.93265	down
SMID_BRE	-0.56004	0.030479	-16.6893	1.53E-35	2.19E-34	70.01441	down
WENG_PO	-0.56004	-0.08264	-16.0755	5.24E-34	6.85E-33	66.4775	down
GOMF_TRI	-0.56002	-0.04457	-19.7999	4.96E-43	1.22E-41	87.26694	down
GOBP_ICO	-0.55997	-0.03139	-28.1898	1.19E-60	1.48E-58	127.8407	down
HALLMARI	-0.55996	-0.15672	-12.3506	2.20E-24	1.58E-23	44.33133	down
GOMF_OX	-0.55994	-0.07809	-12.9332	6.50E-26	5.16E-25	47.84768	down
GOBP_VIT	-0.55987	-0.1646	-12.3041	2.91E-24	2.07E-23	44.05063	down
GOBP_WA	-0.55984	0.041112	-18.93	5.47E-41	1.15E-39	82.56063	down
GOMF_ENI	0.559783	-0.1401	12.83902	1.15E-25	8.96E-25	47.28011	up
GOBP_RES	0.559686	0.001376	10.39034	3.07E-19	1.53E-18	32.51585	up
GOBP_CEL	0.559686	0.001376	10.39034	3.07E-19	1.53E-18	32.51585	up
GOBP_REG	-0.55945	0.097059	-11.3142	1.17E-21	6.90E-21	38.06955	down
MODULE_	0.559365	0.033666	19.48334	2.72E-42	6.31E-41	85.56519	up
GOMF_OX	-0.55934	-0.083	-8.16253	1.46E-13	4.83E-13	19.52255	down
GOMF_NIT	-0.55934	-0.083	-8.16253	1.46E-13	4.83E-13	19.52255	down
HP_GENER	-0.55929	-0.00112	-18.1748	3.50E-39	6.46E-38	78.39894	down
WAKASUG	0.559252	-0.06123	15.60692	8.00E-33	9.77E-32	63.75153	up
GOMF_MI	0.559187	-0.19016	8.301235	6.63E-14	2.25E-13	20.30441	up
MYLLYKAN	0.559177	0.107539	10.41925	2.58E-19	1.29E-18	32.6888	up
GOBP_PO	-0.5591	0.240937	-8.55764	1.53E-14	5.44E-14	21.76177	down
AMIT_SERU	-0.55909	0.090232	-17.7546	3.65E-38	6.28E-37	76.05329	down
GOBP_PO	0.559013	-0.00609	16.79352	8.42E-36	1.23E-34	70.611	up
GOBP_NU	0.558877	-0.04029	12.45241	1.19E-24	8.64E-24	44.94654	up
GOMF_HIG	0.558866	0.017598	10.80843	2.48E-20	1.34E-19	35.02321	up
GOBP_VIT	-0.55882	0.005755	-16.689	1.53E-35	2.20E-34	70.01264	down
CAO_BLOC	-0.55878	-0.05888	-12.3745	1.90E-24	1.37E-23	44.4761	down
GOBP_NE(	-0.55873	0.115667	-8.82378	3.28E-15	1.23E-14	23.28964	down
IKEDA_MIF	0.558679	0.021343	10.49151	1.67E-19	8.50E-19	33.12142	up
GOBP_PO	-0.55856	0.15314	-11.0831	4.72E-21	2.68E-20	36.67608	down
BIDUS_ME	0.558545	0.020758	16.93906	3.67E-36	5.53E-35	71.44195	up
HP_HYPER	-0.55853	-0.04812	-11.1859	2.54E-21	1.47E-20	37.29596	down
GOBP_GLU	-0.55847	-0.04437	-19.0368	3.06E-41	6.52E-40	83.14342	down
GOBP_CEN	0.55841	-0.02719	15.51096	1.40E-32	1.68E-31	63.19055	up
SOX11_TA	0.558397	-0.06323	16.60061	2.54E-35	3.59E-34	69.50599	up
HP_ABNOI	-0.55832	-0.00125	-16.596	2.61E-35	3.68E-34	69.47927	down
GOBP_CEL	0.558301	0.005066	8.697331	6.82E-15	2.50E-14	22.5619	up
GOBP_REG	-0.55821	0.035968	-13.8658	2.39E-28	2.22E-27	53.4503	down
NAKAYA_F	-0.558	0.002527	-10.3746	3.38E-19	1.68E-18	32.42144	down
HP_ACNE	-0.55795	-0.00252	-23.545	2.36E-51	1.26E-49	106.4364	down
GOBP_PRC	0.557521	-0.1056	10.2792	5.98E-19	2.93E-18	31.85161	up

GSE19941_	0.557359	-0.04748	21.002	8.73E-46	2.69E-44	93.61293	up
GOBP_POS	0.557332	-0.07005	8.817916	3.39E-15	1.27E-14	23.25584	up
GOCC_AST	0.557261	0.08071	14.24146	2.53E-29	2.49E-28	55.69262	up
GOBP_PHA	-0.55724	0.037337	-18.854	8.29E-41	1.72E-39	82.14518	down
GOBP_LIPI	-0.55722	-0.00382	-13.1097	2.24E-26	1.84E-25	48.91104	down
HP_PROGF	-0.55719	0.002554	-8.17463	1.36E-13	4.52E-13	19.59053	down
HP_ABNOI	-0.55702	0.007622	-20.2899	3.66E-44	9.84E-43	89.87547	down
GNF2_PTP	-0.55695	0.07666	-9.37781	1.28E-16	5.30E-16	26.51299	down
GOBP_REG	-0.55693	-0.11356	-10.6051	8.44E-20	4.39E-19	33.80215	down
YUAN_ZNI	0.556696	-0.01062	14.19658	3.31E-29	3.23E-28	55.42519	up
HOLLEMAI	0.556659	-0.0222	12.23305	4.48E-24	3.14E-23	43.62118	up
GOBP_CEL	-0.55664	-0.04414	-16.7386	1.15E-35	1.67E-34	70.29659	down
GOBP_RES	-0.55648	-0.0458	-10.915	1.30E-20	7.15E-20	35.66439	down
GOBP_REG	-0.55635	0.059033	-10.7342	3.88E-20	2.06E-19	34.5769	down
MIR6090	-0.5562	0.038446	-11.3118	1.18E-21	6.99E-21	38.05542	down
GOBP_NEC	-0.55617	-0.00866	-10.2222	8.42E-19	4.07E-18	31.51164	down
HP_ABNOI	-0.55612	-0.00219	-14.7634	1.14E-30	1.22E-29	58.79129	down
DELPUECH	0.555975	-0.04915	15.49312	1.56E-32	1.86E-31	63.08614	up
GOCC_GAI	0.555905	-0.07684	10.44167	2.26E-19	1.14E-18	32.82302	up
GOBP_POS	-0.55587	0.000538	-9.90308	5.67E-18	2.59E-17	29.61232	down
REACTOM	0.555805	-0.04235	13.56249	1.47E-27	1.30E-26	51.63291	up
GOBP_CEL	-0.55548	-0.06558	-11.4644	4.71E-22	2.87E-21	38.97663	down
GOBP_MIT	0.555459	-0.03736	12.3364	2.40E-24	1.71E-23	44.2457	up
GOBP_REG	-0.55539	0.231926	-8.27803	7.57E-14	2.56E-13	20.17328	down
GOBP_INT	-0.55531	-0.08883	-10.8925	1.49E-20	8.16E-20	35.52874	down
GOMF_FEF	-0.55525	-0.02839	-21.509	6.35E-47	2.16E-45	96.23435	down
chr4q25	-0.55514	0.021735	-13.3533	5.17E-27	4.42E-26	50.37693	down
NAGY_TFT	0.55511	-0.09797	16.29639	1.46E-34	1.98E-33	67.75518	up
GOMF_BIL	-0.55499	-0.10562	-16.9533	3.38E-36	5.11E-35	71.52308	down
GOMF_RN	0.554928	-0.01328	12.8489	1.08E-25	8.46E-25	47.33971	up
GOMF_IMI	-0.55492	0.105196	-11.87	4.04E-23	2.65E-22	41.42697	down
GOBP_TEL	0.554906	-0.11171	9.652759	2.51E-17	1.10E-16	28.13075	up
AIZARANI_	-0.55491	0.027743	-17.1408	1.16E-36	1.81E-35	72.5899	down
GOMF_3_E	-0.55489	-0.1354	-9.85475	7.56E-18	3.43E-17	29.32569	down
ZHONG_PI	0.554487	-0.08383	13.93652	1.56E-28	1.47E-27	53.87293	up
GINESTIER	0.554287	-0.00863	24.25471	7.62E-53	4.62E-51	109.8717	up
REACTOM	0.554272	-0.05194	20.36579	2.45E-44	6.72E-43	90.27706	up
MODULE_	-0.55421	0.009218	-19.1378	1.76E-41	3.86E-40	83.6933	down
BIOCARTA	0.554125	-0.03387	15.13892	1.24E-31	1.41E-30	61.00753	up
FAN_EMBF	-0.55403	0.104402	-11.0715	5.07E-21	2.86E-20	36.60657	down
HP_CHOR	-0.55402	-0.04802	-16.5902	2.70E-35	3.80E-34	69.44638	down
GOBP_ELE	-0.55401	-0.17086	-14.7887	9.83E-31	1.06E-29	58.94121	down
MATSUMI	-0.55398	0.063972	-9.3204	1.79E-16	7.35E-16	26.1766	down
HP_ABNOI	-0.55397	0.027712	-20.1353	8.30E-44	2.17E-42	89.05606	down
WP_VITAM	-0.55394	-0.00866	-16.2542	1.87E-34	2.50E-33	67.51163	down
GOBP_FAT	-0.55385	-0.00425	-24.3119	5.79E-53	3.57E-51	110.1461	down
GOBP_VIRI	-0.55382	0.02247	-12.2548	3.93E-24	2.77E-23	43.75287	down
HP_HYPOF	-0.55371	0.050461	-8.94074	1.66E-15	6.34E-15	23.96558	down
REACTOM	0.553669	-0.05124	13.24565	9.89E-27	8.32E-26	49.72944	up
HP_ULCER	-0.55355	0.007503	-12.2643	3.71E-24	2.62E-23	43.81018	down
GOCC_PLA	-0.55352	0.010527	-20.2338	4.92E-44	1.31E-42	89.57848	down
HP_LETHA	-0.55341	-0.09796	-19.3315	6.17E-42	1.40E-40	84.74452	down
GUTIERREZ	-0.5532	0.072715	-11.9315	2.78E-23	1.85E-22	41.79849	down
GOBP_DNA	0.553194	-0.00863	20.13548	8.29E-44	2.17E-42	89.05677	up
HP_VISCEF	-0.55311	0.104532	-13.0403	3.41E-26	2.76E-25	48.49339	down
GOBP_WA	-0.5531	-0.03542	-22.7726	1.07E-49	4.82E-48	102.6271	down
GOBP_T_H	-0.55304	0.113744	-12.3459	2.26E-24	1.62E-23	44.30308	down
GOCC_CLA	0.553021	-0.14547	11.55915	2.65E-22	1.64E-21	39.54853	up

MODULE_:	0.552693	-0.13714	9.17241	4.28E-16	1.71E-15	25.31183	up
GOBP_NEC	-0.55254	0.095319	-9.99368	3.30E-18	1.54E-17	30.15038	down
BLUM_RES	0.552445	-0.01594	17.54064	1.21E-37	2.01E-36	74.85114	up
GOBP_REG	0.552433	0.051433	15.0363	2.28E-31	2.54E-30	60.40316	up
HOSHIDA_	-0.55242	-0.00949	-22.6804	1.69E-49	7.38E-48	102.1675	down
SMID_BRE/	-0.55228	0.042137	-17.1409	1.16E-36	1.81E-35	72.59058	down
GOMF_TYF	-0.55226	0.088627	-9.98966	3.38E-18	1.57E-17	30.12646	down
GOMF_SO	-0.5522	0.026915	-15.6059	8.05E-33	9.83E-32	63.74537	down
GOBP_POS	-0.55198	0.039784	-12.7736	1.70E-25	1.31E-24	46.88565	down
GOCC_NPI	0.551971	0.070701	14.83558	7.45E-31	8.08E-30	59.21844	up
GCM_TPR	0.551142	0.013899	13.48097	2.40E-27	2.10E-26	51.14362	up
HP_LACRII	0.551104	0.027471	15.75048	3.46E-33	4.32E-32	64.58911	up
WP_STERC	-0.55109	-0.16863	-11.4012	6.90E-22	4.16E-21	38.59472	down
GOBP_REG	-0.55104	0.091306	-12.5422	6.90E-25	5.09E-24	45.48891	down
GOBP_NEC	-0.55077	0.094297	-14.3787	1.12E-29	1.12E-28	56.5092	down
GERHOLD_	-0.55066	-0.05413	-18.3748	1.16E-39	2.22E-38	79.50795	down
REACTOM	0.550486	-0.01466	16.07174	5.36E-34	7.00E-33	66.45598	up
GOBP_NO	-0.55044	0.026949	-13.7193	5.74E-28	5.20E-27	52.57287	down
MODULE_:	0.550432	-0.05385	8.607418	1.15E-14	4.12E-14	22.04643	up
GOBP_NEC	-0.55042	0.033077	-13.3997	3.91E-27	3.37E-26	50.6556	down
HP_BRUSH	-0.55033	-0.15136	-11.9888	1.97E-23	1.32E-22	42.14507	down
REACTOM	-0.55032	-0.12056	-8.43569	3.08E-14	1.07E-13	21.06677	down
GOCC_TR/	0.550256	-0.10601	14.08931	6.28E-29	6.03E-28	54.78546	up
HP_FIBULA	0.550214	-0.01269	12.18993	5.82E-24	4.05E-23	43.36055	up
BIOCARTA	0.550041	-0.01665	11.15654	3.03E-21	1.74E-20	37.11891	up
GOLUB_AL	-0.55	0.0007	-13.9972	1.09E-28	1.03E-27	54.23534	down
GOBP_NEC	-0.54988	0.000534	-18.216	2.79E-39	5.19E-38	78.62798	down
GOBP_OLE	-0.54972	-0.05281	-25.1537	1.07E-54	7.73E-53	114.1356	down
GOBP_VIT/	-0.54959	-0.03011	-26.7024	8.63E-58	8.31E-56	121.2576	down
GOMF_AN	-0.54946	-0.05448	-23.7441	8.96E-52	4.96E-50	107.4065	down
MODULE_:	0.549443	-0.0293	20.25443	4.41E-44	1.18E-42	89.68786	up
GOMF_OX	-0.54931	0.134806	-10.4898	1.69E-19	8.58E-19	33.11145	down
GOCC_CO	0.54927	-0.00276	21.01702	8.07E-46	2.51E-44	93.69109	up
GOBP_NEL	-0.54926	-0.05632	-18.6891	2.05E-40	4.09E-39	81.24085	down
SCHERER_I	-0.54913	0.030299	-13.9465	1.47E-28	1.39E-27	53.93262	down
GOBP_POS	-0.54908	0.111195	-10.6892	5.09E-20	2.68E-19	34.3071	down
GOBP_NEL	-0.54906	0.12559	-12.3785	1.86E-24	1.34E-23	44.50005	down
GOBP_POS	-0.54905	-0.09648	-13.4491	2.91E-27	2.53E-26	50.952	down
REACTOM	-0.54904	-0.11544	-14.6877	1.79E-30	1.89E-29	58.34324	down
GOBP_IRO	-0.54902	0.024564	-18.022	8.19E-39	1.47E-37	77.54877	down
GOCC_ME	0.548978	-0.02471	17.34277	3.71E-37	5.94E-36	73.73435	up
HP_REDUC	-0.54897	0.06601	-9.22016	3.23E-16	1.30E-15	25.59047	down
TANG_SEN	-0.54894	0.014382	-21.0112	8.32E-46	2.58E-44	93.66058	down
WP_FERRC	-0.54881	-0.02598	-15.9232	1.27E-33	1.62E-32	65.59432	down
GOBP_REG	-0.5488	0.008603	-11.2859	1.39E-21	8.14E-21	37.89874	down
GOMF_BET	-0.54879	0.081733	-9.72332	1.65E-17	7.30E-17	28.54762	down
MODULE_:	-0.54873	-0.05933	-17.5341	1.26E-37	2.09E-36	74.81438	down
GSE25085_	0.548692	-0.06219	19.81048	4.69E-43	1.15E-41	87.32341	up
SMID_BRE/	0.548677	0.061986	15.0204	2.50E-31	2.79E-30	60.30945	up
GOBP_POS	0.548582	-0.0535	10.82324	2.27E-20	1.23E-19	35.11225	up
GOBP_GAS	-0.54856	0.091807	-16.3732	9.39E-35	1.29E-33	68.19825	down
GOBP_POS	-0.54835	0.081458	-13.7722	4.18E-28	3.82E-27	52.88966	down
MODULE_!	-0.54825	-0.03273	-32.8275	6.17E-69	1.82E-66	146.8826	down
GOCC_BO:	0.548091	-0.059	7.031852	7.46E-11	2.01E-10	13.35399	up
WP_TRANS	-0.54805	-0.09377	-12.3379	2.38E-24	1.70E-23	44.25466	down
GOBP_POS	-0.54788	-0.0156	-15.1551	1.13E-31	1.28E-30	61.10297	down
HP_APLAS	0.547744	0.110646	10.9167	1.29E-20	7.09E-20	35.67436	up
GOBP_ACY	-0.54771	0.00138	-9.83432	8.54E-18	3.86E-17	29.20461	down

REACTOM	-0.54771	0.00138	-9.83432	8.54E-18	3.86E-17	29.20461	down
GOBP_NEC	-0.54769	-0.01834	-20.4865	1.30E-44	3.63E-43	90.91412	down
HP_ACCES	0.547616	-0.0149	16.9094	4.34E-36	6.51E-35	71.27278	up
GOCC_MIT	0.547576	-0.03767	16.3801	9.02E-35	1.24E-33	68.23792	up
REACTOM	0.547533	0.011303	12.74178	2.07E-25	1.58E-24	46.69357	up
SOBOLEV_	-0.54745	0.150038	-10.3116	4.92E-19	2.42E-18	32.04528	down
GOMF_BET	-0.54745	-0.09538	-9.10922	6.20E-16	2.44E-15	24.94368	down
GOBP_NEC	-0.54743	-0.10419	-8.73285	5.55E-15	2.04E-14	22.76598	down
GOBP_POS	0.54738	0.046942	9.87319	6.78E-18	3.08E-17	29.43501	up
GOBP_DNA	0.547251	-0.02489	21.62084	3.58E-47	1.24E-45	96.8085	up
GOBP_NO	-0.54719	0.205919	-11.8989	3.39E-23	2.24E-22	41.60134	down
KAUFFMAI	0.547178	-0.05307	18.44051	8.04E-40	1.56E-38	79.87124	up
REACTOM	-0.54704	-0.20632	-10.6141	8.00E-20	4.17E-19	33.85616	down
GOBP_NEU	-0.54703	0.091417	-8.22359	1.03E-13	3.45E-13	19.86616	down
ZHU_CMV_	-0.547	0.011553	-11.806	5.95E-23	3.86E-22	41.04021	down
SENGUPTA	0.546962	0.060303	20.02925	1.46E-43	3.75E-42	88.49168	up
GOBP_ON	-0.54692	0.138647	-10.6071	8.34E-20	4.34E-19	33.81405	down
REACTOM	0.54691	0.012949	13.74566	4.90E-28	4.46E-27	52.73089	up
GOBP_HIS	0.546868	0.079714	10.08654	1.90E-18	8.97E-18	30.70276	up
HP_CIGAR	0.546745	0.039884	7.95998	4.58E-13	1.46E-12	18.3896	up
GOBP_NEC	-0.54669	0.314998	-8.03598	2.99E-13	9.65E-13	18.81342	down
REACTOM	0.546693	-0.06016	13.12369	2.06E-26	1.70E-25	48.99547	up
REACTOM	0.546468	-0.11218	9.973519	3.72E-18	1.73E-17	30.03056	up
HP_CUTAN	0.546439	-0.00807	18.16207	3.76E-39	6.91E-38	78.3283	up
GOBP_TYP	-0.54634	0.04963	-9.90436	5.63E-18	2.57E-17	29.61991	down
GOBP_ALP	-0.54614	-0.07093	-15.8493	1.95E-33	2.46E-32	65.16468	down
HP_HYPER	-0.54608	-0.02346	-17.8735	1.88E-38	3.29E-37	76.71908	down
GOMF_INC	0.545695	-0.10623	10.56349	1.08E-19	5.59E-19	33.55278	up
GOBP_REG	-0.54553	0.00884	-12.6856	2.90E-25	2.20E-24	46.3544	down
HP_SEVER	-0.54548	-0.11838	-10.0374	2.54E-18	1.19E-17	30.41041	down
GOBP_FLA	-0.54546	-0.0043	-8.1638	1.45E-13	4.79E-13	19.52964	down
GOBP_ANI	-0.54542	-0.11277	-11.7871	6.67E-23	4.31E-22	40.92559	down
MATZUK_(	-0.54542	0.112532	-14.8871	5.49E-31	6.01E-30	59.52259	down
GOMF_LYS	0.545331	0.047231	15.00732	2.70E-31	3.00E-30	60.23236	up
RUAN_RES	-0.54528	-0.05287	-14.3577	1.27E-29	1.27E-28	56.3845	down
GOBP_REG	-0.5452	-0.05937	-13.6594	8.23E-28	7.37E-27	52.21384	down
REACTOM	-0.54518	-0.04891	-13.6825	7.16E-28	6.45E-27	52.35275	down
CHEN_LVA	-0.54504	0.055901	-18.7152	1.77E-40	3.58E-39	81.38415	down
GNF2_SMC	0.544987	-0.05112	12.22506	4.70E-24	3.30E-23	43.57289	up
RB_P107_D	0.544981	-0.03221	17.46201	1.89E-37	3.10E-36	74.40791	up
GOBP_NEC	-0.54487	0.006906	-14.8718	6.01E-31	6.56E-30	59.43255	down
GOBP_NEC	-0.54477	0.18946	-10.2925	5.52E-19	2.71E-18	31.93124	down
WP_FOLAT	-0.54473	-0.09927	-13.5611	1.48E-27	1.31E-26	51.62456	down
DESCARTE	-0.54459	-0.00254	-18.1892	3.23E-39	5.98E-38	78.47893	down
REACTOM	0.544473	-0.06393	11.70718	1.08E-22	6.88E-22	40.44287	up
GOBP_CON	-0.5443	0.158898	-8.07495	2.40E-13	7.80E-13	19.03135	down
HP_NUMEI	0.544279	0.102246	11.10747	4.08E-21	2.32E-20	36.82318	up
GOBP_REG	0.544206	0.016006	10.78468	2.86E-20	1.53E-19	34.88049	up
GOMF_BUI	0.543953	-0.04069	12.58278	5.40E-25	4.01E-24	45.73388	up
GOBP_LEY	-0.54386	0.084477	-15.3901	2.85E-32	3.35E-31	62.48291	down
DESCARTE	-0.54361	0.137595	-8.22098	1.05E-13	3.50E-13	19.85144	down
HP_PREMA	-0.54341	-0.06194	-10.3403	4.15E-19	2.05E-18	32.21636	down
GOBP_RES	-0.54338	0.125467	-13.2405	1.02E-26	8.57E-26	49.69829	down
HP_MORN	0.543277	-0.05615	11.9784	2.09E-23	1.40E-22	42.08206	up
REACTOM	0.543248	0.006888	17.73373	4.11E-38	7.03E-37	75.93646	up
SEF1_C	0.543229	-0.00254	11.89378	3.50E-23	2.30E-22	41.57056	up
MODULE_4	-0.54317	0.148636	-10.4982	1.61E-19	8.18E-19	33.16174	down
GOBP_CEL	-0.54296	0.096005	-9.59352	3.57E-17	1.54E-16	27.78133	down

BIOCARTA	-0.5429	-0.13595	-9.93449	4.70E-18	2.16E-17	29.79874	down
GOMF_CA	-0.54289	-0.08325	-10.2343	7.83E-19	3.80E-18	31.58363	down
GOBP_REG	0.54284	-0.1162	9.653738	2.50E-17	1.09E-16	28.13653	up
HOFT_PBM	-0.54271	0.102248	-10.7055	4.61E-20	2.44E-19	34.40496	down
GOBP_OD	-0.54271	0.012793	-10.2929	5.51E-19	2.71E-18	31.93332	down
LI_PBMC_N	-0.5426	0.105957	-11.2426	1.80E-21	1.05E-20	37.63775	down
GOBP_HO	-0.54247	-0.04417	-28.3143	6.91E-61	8.89E-59	128.3809	down
HP_VERY_L	-0.5424	-0.15508	-12.0206	1.62E-23	1.10E-22	42.33709	down
GOBP_ADE	0.542227	0.043335	10.34803	3.96E-19	1.96E-18	32.26289	up
REACTOM	0.542185	-0.04702	10.98073	8.77E-21	4.87E-20	36.05975	up
HP_DYSGE	0.541868	0.024757	9.785204	1.14E-17	5.11E-17	28.91369	up
GOBP_CEL	-0.54187	-0.08712	-11.9736	2.16E-23	1.44E-22	42.05294	down
MODULE_2	-0.54173	0.00347	-19.2736	8.44E-42	1.89E-40	84.43093	down
GOMF_GLI	-0.5417	0.071895	-9.69236	1.99E-17	8.73E-17	28.3646	down
GOCC_EXT	-0.54152	0.059363	-11.3254	1.09E-21	6.47E-21	38.1371	down
GOCC_MIT	-0.54142	-0.13793	-14.592	3.15E-30	3.29E-29	57.77648	down
HP_HYPOF	0.541248	0.004191	20.04239	1.36E-43	3.50E-42	88.56169	up
HP_HYPOF	0.541187	-0.01008	10.32688	4.49E-19	2.22E-18	32.13645	up
GOBP_LEU	-0.54106	0.143195	-12.8123	1.35E-25	1.05E-24	47.11913	down
WP_EXTRA	-0.54078	-0.0128	-16.2532	1.88E-34	2.51E-33	67.50555	down
WP_ONE_(	-0.54054	-0.08171	-20.1159	9.20E-44	2.40E-42	88.95264	down
GOBP_MIT	0.540482	-0.07408	16.02503	7.02E-34	9.13E-33	66.18518	up
GOCC_OXI	-0.54044	-0.14845	-13.0758	2.75E-26	2.25E-25	48.70727	down
WP_MACR	-0.54035	0.148603	-8.06231	2.57E-13	8.36E-13	18.96059	down
HP_ABNOI	0.540236	0.048328	17.57985	9.74E-38	1.63E-36	75.07184	up
GOBP_RES	-0.54023	0.025469	-22.5446	3.33E-49	1.42E-47	101.4887	down
REACTOM	-0.54015	-0.04938	-22.3405	9.27E-49	3.77E-47	100.4637	down
HP_ACUTE	-0.53996	-0.04653	-19.7827	5.44E-43	1.33E-41	87.1745	down
MOOTHA_	-0.53995	-0.02276	-13.8839	2.14E-28	2.00E-27	53.55826	down
REACTOM	0.539825	0.013324	13.01882	3.88E-26	3.13E-25	48.36393	up
GOCC_SEX	0.539801	0.033075	15.87246	1.70E-33	2.16E-32	65.29916	up
GOMF_LIG	-0.53978	-0.02723	-20.5596	8.82E-45	2.49E-43	91.29888	down
PLASARI_T	-0.53971	0.15223	-11.3243	1.10E-21	6.51E-21	38.13074	down
GOCC_ATF	-0.53966	-0.19579	-8.50325	2.09E-14	7.37E-14	21.45141	down
GOBP_END	-0.53949	0.114371	-9.58254	3.81E-17	1.64E-16	27.71656	down
HP_ABNOI	0.539431	0.009762	24.98499	2.37E-54	1.66E-52	113.3428	up
GOBP_NEC	-0.53933	0.078778	-13.8155	3.23E-28	2.97E-27	53.14916	down
BIOCARTA	0.53919	0.003925	15.43217	2.22E-32	2.64E-31	62.72931	up
LEE_METAS	0.539183	-0.07004	10.50669	1.53E-19	7.79E-19	33.21239	up
HP_NAEVU	0.53917	-0.15235	12.58543	5.31E-25	3.95E-24	45.74987	up
GSE36476_	0.538804	-0.09519	13.80743	3.39E-28	3.12E-27	53.10077	up
GOBP_RES	-0.53863	0.061198	-8.88086	2.35E-15	8.90E-15	23.61921	down
WAESCH_A	0.538599	-0.0912	12.76992	1.74E-25	1.34E-24	46.86332	up
HP_PANCF	-0.53847	-0.03269	-23.1005	2.10E-50	1.01E-48	104.2534	down
GOBP_PRC	-0.5383	0.056714	-12.9052	7.70E-26	6.08E-25	47.67944	down
GOBP_CEL	-0.53826	0.039851	-16.7147	1.32E-35	1.91E-34	70.15976	down
REACTOM	0.538239	0.038207	20.30165	3.44E-44	9.29E-43	89.93791	up
REACTOM	0.538222	-0.0049	17.999	9.32E-39	1.67E-37	77.42032	up
HP_STEAT	-0.5382	-0.02306	-21.4634	8.03E-47	2.72E-45	96.00009	down
SCHAEFFEI	-0.53811	0.058927	-12.835	1.18E-25	9.17E-25	47.2557	down
GOCC_U2_	0.538105	-0.10817	9.707779	1.81E-17	7.99E-17	28.45572	up
GOBP_RES	0.538059	0.00877	12.80257	1.43E-25	1.11E-24	47.06029	up
GOMF_HIS	0.538054	-0.03446	17.1812	9.26E-37	1.45E-35	72.81916	up
GOBP_POS	0.537993	0.006614	15.74734	3.53E-33	4.40E-32	64.57079	up
HP_INCREA	-0.53784	-0.14571	-13.4111	3.66E-27	3.16E-26	50.72373	down
HP_ABNOI	0.537789	0.042797	16.59883	2.57E-35	3.62E-34	69.49579	up
GOBP_DOI	0.537747	-0.03358	19.69458	8.72E-43	2.10E-41	86.70208	up
GOBP_DRU	-0.53757	-0.09074	-11.1672	2.84E-21	1.64E-20	37.18331	down

GOMF_LYA	-0.53757	-0.07649	-23.6868	1.18E-51	6.50E-50	107.1277	down
GNF2_PA2	0.537518	-0.1149	10.6878	5.13E-20	2.71E-19	34.29859	up
GOBP_NEG	-0.53739	0.044503	-15.174	1.01E-31	1.15E-30	61.2137	down
GNF2_PEC	-0.53735	0.099703	-9.23639	2.94E-16	1.19E-15	25.68523	down
MORF_FEN	0.537296	-0.11712	10.88146	1.60E-20	8.70E-20	35.4624	up
HP_KETOA	-0.53719	0.006568	-20.2655	4.16E-44	1.11E-42	89.74648	down
GOBP_TRIG	-0.53717	0.16597	-9.5781	3.91E-17	1.68E-16	27.69041	down
GOBP_NEG	-0.53712	0.153307	-7.08502	5.62E-11	1.53E-10	13.6347	down
HP_VOMIT	-0.53705	-0.07172	-25.3297	4.70E-55	3.54E-53	114.9592	down
ITO_PTTG1	0.537003	-0.03019	10.41892	2.59E-19	1.30E-18	32.68684	up
HP_EPISOD	-0.537	-0.05079	-20.5922	7.43E-45	2.12E-43	91.47018	down
GOBP_POS	0.536993	0.023381	10.02014	2.82E-18	1.32E-17	30.3077	up
HP_ABNOI	0.536866	0.048284	9.63758	2.75E-17	1.20E-16	28.04116	up
chr1p12	-0.53677	0.107045	-10.0682	2.12E-18	9.98E-18	30.59383	down
GOBP_CEL	-0.53677	-0.07274	-18.0513	6.96E-39	1.26E-37	77.71163	down
HP_ARTER	-0.53658	0.061387	-14.4301	8.24E-30	8.36E-29	56.81492	down
WU_SILEN	-0.53654	0.08211	-15.7902	2.75E-33	3.45E-32	64.82048	down
REACTOM	0.536462	-0.04664	18.05729	6.73E-39	1.22E-37	77.74526	up
GOBP_EST	0.536461	0.038764	18.31426	1.62E-39	3.06E-38	79.17283	up
HP_IRIS_AT	-0.53628	0.122977	-10.8155	2.38E-20	1.28E-19	35.06569	down
chr18q22	-0.53627	0.133923	-13.7807	3.98E-28	3.64E-27	52.9409	down
REACTOM	-0.53592	-0.19133	-10.9191	1.27E-20	6.99E-20	35.68877	down
GOBP_NEG	0.535894	-0.00039	20.05334	1.28E-43	3.31E-42	88.61998	up
GOBP_MIT	0.535888	-0.07462	16.54776	3.44E-35	4.82E-34	69.20251	up
GOMF_NFA	-0.53566	0.091726	-9.28636	2.19E-16	8.94E-16	25.97735	down
GOBP_REG	0.535538	0.036939	9.872773	6.79E-18	3.09E-17	29.43254	up
WP_ATM_S	0.535496	0.00333	17.17036	9.85E-37	1.53E-35	72.75764	up
GOCC_RES	-0.5354	-0.22029	-8.91389	1.94E-15	7.38E-15	23.81016	down
GOBP_REG	0.535259	-0.01683	18.27352	2.03E-39	3.80E-38	78.94703	up
GOBP_NEG	-0.53522	-0.09043	-9.93201	4.77E-18	2.19E-17	29.78404	down
GOBP_ACE	-0.53522	-0.03718	-11.8473	4.63E-23	3.03E-22	41.28966	down
ZHENG_CO	-0.53509	0.071731	-14.6828	1.84E-30	1.95E-29	58.31424	down
GOCC_PRO	0.535074	-0.13209	12.05011	1.36E-23	9.20E-23	42.5155	up
QUEREC_N	-0.53492	0.00877	-20.3287	2.98E-44	8.14E-43	90.08101	down
GOCC_NU	0.534913	-0.23548	8.185084	1.28E-13	4.27E-13	19.64934	up
REACTOM	-0.53453	0.068678	-10.0815	1.95E-18	9.23E-18	30.67291	down
HP_SYSTEN	-0.53448	-0.00494	-14.108	5.62E-29	5.41E-28	54.89672	down
HP_RETICU	0.534479	-0.13014	10.78574	2.84E-20	1.53E-19	34.88683	up
HOEK_PBN	-0.53445	0.140629	-11.5094	3.58E-22	2.20E-21	39.24819	down
HP_CENTR	0.534434	-0.01017	10.53765	1.27E-19	6.50E-19	33.39786	up
HP_HYPER	0.534353	-0.04235	12.84868	1.08E-25	8.47E-25	47.33838	up
GOCC_INC	0.534298	-0.1177	14.01704	9.67E-29	9.20E-28	54.35402	up
GOBP_RES	-0.53424	-0.19779	-11.1207	3.76E-21	2.15E-20	36.90292	down
HP_TRACH	0.534144	0.023856	21.9786	5.77E-48	2.18E-46	98.63377	up
GOCC_PRO	0.534016	-0.14648	12.21744	4.92E-24	3.45E-23	43.52685	up
HP_SIDERO	0.533973	-0.05193	12.51697	8.04E-25	5.90E-24	45.33648	up
GOBP_NEG	-0.53392	0.1286	-10.6001	8.70E-20	4.52E-19	33.77234	down
GOBP_REG	-0.53386	-0.04528	-17.1903	8.79E-37	1.38E-35	72.87065	down
GOBP_NEG	-0.5338	-0.04065	-11.3871	7.51E-22	4.51E-21	38.50991	down
HP_CHOLE	-0.53368	-0.03158	-13.0403	3.41E-26	2.76E-25	48.49361	down
GOBP_NEG	0.533669	-0.10255	10.10045	1.74E-18	8.27E-18	30.78559	up
GOBP_SUC	-0.53364	-0.1291	-11.7667	7.55E-23	4.85E-22	40.80255	down
GOMF_SUC	-0.53364	-0.1291	-11.7667	7.55E-23	4.85E-22	40.80255	down
KEGG_ARA	-0.53359	-0.00802	-23.1712	1.48E-50	7.23E-49	104.6022	down
GOBP_POS	-0.53359	0.148915	-10.6029	8.55E-20	4.45E-19	33.7892	down
HP_FLAT_(	0.533516	0.040134	11.69656	1.15E-22	7.32E-22	40.3787	up
LAKE_ADU	-0.53348	0.155337	-8.96475	1.44E-15	5.53E-15	24.10464	down
MODULE_8	-0.53345	-0.02562	-31.6184	7.28E-67	1.67E-64	142.1227	down

GOBP_REG	0.53324	-0.0167	19.55803	1.82E-42	4.26E-41	85.96782	up
REACTOM	0.533119	0.014978	15.48015	1.68E-32	2.01E-31	63.01025	up
GOMF_AD	-0.53306	-0.1967	-9.71537	1.73E-17	7.65E-17	28.50056	down
GOBP_POS	-0.53306	-0.08651	-12.4146	1.49E-24	1.08E-23	44.71844	down
GOMF_LOI	-0.53293	-0.08282	-14.1011	5.85E-29	5.63E-28	54.85605	down
GOBP_REG	-0.53286	0.11721	-14.0161	9.72E-29	9.24E-28	54.34855	down
GOMF_2_A	-0.53278	-0.05945	-8.51651	1.93E-14	6.84E-14	21.52703	down
HP_PERIPH	-0.53275	-0.0382	-22.6349	2.12E-49	9.13E-48	101.9404	down
GOBP_MEI	0.532749	-0.01623	21.70675	2.31E-47	8.15E-46	97.24828	up
GOCC_HFE	-0.53266	-0.01249	-11.2088	2.21E-21	1.28E-20	37.43383	down
WP_RIBOF	-0.5326	-0.12933	-13.5155	1.95E-27	1.71E-26	51.35067	down
REACTOM	-0.53232	-0.10178	-16.2098	2.41E-34	3.21E-33	67.25526	down
HP_ABNOI	-0.53225	-0.01684	-13.7386	5.12E-28	4.65E-27	52.68879	down
GNF2_ANF	0.532188	-0.02597	10.2627	6.60E-19	3.22E-18	31.75307	up
GOBP_PAF	-0.53206	0.153324	-13.648	8.81E-28	7.88E-27	52.146	down
GOBP_OXI	-0.53191	-0.04387	-15.1746	1.01E-31	1.15E-30	61.21758	down
GOBP_REG	-0.53183	-0.0022	-13.7663	4.33E-28	3.96E-27	52.8543	down
CTACTAG_	-0.53178	0.121269	-13.1636	1.62E-26	1.34E-25	49.23548	down
HP_LIMITE	-0.53172	0.043058	-11.1033	4.18E-21	2.38E-20	36.798	down
GOCC_CRI	0.531688	0.025831	8.697348	6.82E-15	2.50E-14	22.562	up
GOBP_POS	-0.5316	-0.15821	-9.95847	4.07E-18	1.88E-17	29.94115	down
DAUER_ST	-0.53154	-0.02356	-11.3916	7.31E-22	4.40E-21	38.53697	down
HP_DENTA	0.531467	0.012286	9.964755	3.92E-18	1.82E-17	29.97849	up
REACTOM	-0.53141	-0.05528	-10.6741	5.57E-20	2.93E-19	34.21652	down
MOOTHA_	-0.5314	-0.12593	-9.663	2.36E-17	1.03E-16	28.19119	down
GOBP_POS	-0.53133	-0.06586	-9.8004	1.05E-17	4.68E-17	29.00369	down
GOBP_POS	-0.53131	-0.07152	-11.4824	4.22E-22	2.58E-21	39.08488	down
REACTOM	0.531146	-0.06796	10.11848	1.57E-18	7.45E-18	30.893	up
GOBP_REG	-0.53101	0.044622	-9.19119	3.83E-16	1.54E-15	25.42136	down
WEST_ADF	0.530732	-0.07569	15.80592	2.51E-33	3.15E-32	64.91202	up
GOBP_REG	-0.53064	0.088779	-26.2978	5.40E-57	4.87E-55	119.4239	down
REACTOM	-0.53049	0.102131	-10.9897	8.30E-21	4.62E-20	36.11405	down
HP_FLAREI	-0.53048	0.040757	-9.10879	6.21E-16	2.45E-15	24.94119	down
HP_ABNOI	0.530471	0.069249	12.61287	4.50E-25	3.36E-24	45.91555	up
WP_MITOC	-0.53041	-0.21514	-10.1089	1.66E-18	7.88E-18	30.83603	down
BIOCARTA	-0.53031	-0.07994	-12.8088	1.38E-25	1.07E-24	47.09759	down
GOBP_REG	0.5301	0.023798	13.33306	5.84E-27	4.98E-26	50.25507	up
GOMF_GLI	-0.53009	-0.07723	-14.6853	1.81E-30	1.92E-29	58.32948	down
BRACHAT_	-0.52996	-0.01806	-16.4521	5.96E-35	8.25E-34	68.65262	down
GOBP_NEC	-0.52993	0.112275	-10.427	2.46E-19	1.24E-18	32.73506	down
GOBP_POS	0.52985	0.005674	9.942643	4.48E-18	2.06E-17	29.84716	up
GOBP_POS	0.529815	-0.00724	20.33469	2.89E-44	7.90E-43	90.11267	up
WIEMANN	-0.52957	-0.11846	-11.4211	6.12E-22	3.69E-21	38.7149	down
GOBP_ENE	-0.52949	-0.12423	-16.717	1.30E-35	1.89E-34	70.1731	down
KEGG_STA	-0.52948	-0.03633	-15.1096	1.48E-31	1.67E-30	60.83485	down
REACTOM	0.529461	-0.0169	13.25377	9.42E-27	7.92E-26	49.77828	up
GOBP_CEL	-0.52942	-0.16056	-12.9571	5.63E-26	4.49E-25	47.99178	down
GNF2_BUB	0.529315	-0.07772	10.2125	8.92E-19	4.31E-18	31.45346	up
HP_FLUSH	-0.52926	-0.05626	-15.873	1.70E-33	2.16E-32	65.3025	down
GOCC_HET	0.529249	0.000965	21.8788	9.59E-48	3.51E-46	98.12625	up
GOBP_REG	0.529197	0.068105	11.61309	1.91E-22	1.19E-21	39.87439	up
GRAHAM_	-0.52887	0.14245	-10.1741	1.12E-18	5.39E-18	31.22454	down
REACTOM	-0.52872	-0.04114	-12.7404	2.08E-25	1.59E-24	46.68548	down
GNF2_APE	0.528685	-0.03577	10.79259	2.73E-20	1.47E-19	34.92804	up
REACTOM	-0.52859	0.120965	-12.0013	1.82E-23	1.23E-22	42.22071	down
HOEK_PBM	-0.52859	0.042838	-10.384	3.19E-19	1.59E-18	32.478	down
GOBP_PRE	-0.52835	0.124953	-9.87982	6.51E-18	2.97E-17	29.47436	down
GOBP_SPII	0.528333	0.030262	20.78648	2.68E-45	7.98E-44	92.48866	up

WUNDER_	-0.52826	0.039023	-11.0598	5.44E-21	3.07E-20	36.53576	down
GOBP_NEC	-0.52806	-0.02751	-10.162	1.21E-18	5.79E-18	31.15239	down
GOBP_FAT	-0.52802	-0.00427	-24.7532	7.08E-54	4.76E-52	112.2482	down
HP_SEVERI	0.527983	-0.09907	10.06783	2.12E-18	9.99E-18	30.59139	up
SCHERER_I	-0.52781	-0.03646	-10.889	1.53E-20	8.32E-20	35.5077	down
SCHERER_I	-0.52781	-0.03646	-10.889	1.53E-20	8.32E-20	35.5077	down
HP_ELEVAT	-0.5278	-0.14716	-9.23539	2.96E-16	1.19E-15	25.67942	down
REACTOM	-0.52767	-0.15906	-12.3446	2.28E-24	1.63E-23	44.29499	down
GOBP_HIST	0.527655	0.065435	10.88131	1.60E-20	8.71E-20	35.46147	up
HP_ABNOI	-0.52761	0.013376	-10.5848	9.54E-20	4.94E-19	33.68081	down
GOCC_ME	0.527544	0.086296	14.11394	5.42E-29	5.23E-28	54.93242	up
GOMF_MC	-0.52751	-0.05315	-21.8286	1.24E-47	4.49E-46	97.87042	down
HP_PROXII	-0.52748	0.021291	-9.56145	4.32E-17	1.85E-16	27.59234	down
GOCC_ARI	0.527479	-0.01581	9.644027	2.65E-17	1.15E-16	28.07921	up
GOBP_PTE	-0.52725	-0.08781	-15.9418	1.14E-33	1.46E-32	65.7021	down
GOBP_HIST	0.527056	0.051552	15.21489	7.96E-32	9.14E-31	61.45434	up
GOBP_MIT	0.526683	-0.02415	22.17336	2.15E-48	8.49E-47	99.62067	up
HP_FAT_M	-0.52663	-0.00667	-23.4217	4.32E-51	2.23E-49	105.8331	down
GOBP_CEL	-0.52662	-0.0346	-23.7915	7.12E-52	4.00E-50	107.6366	down
HECKER_IF	-0.52636	0.020325	-11.8021	6.09E-23	3.95E-22	41.01621	down
TESAR_ALI	0.52632	-0.09808	10.98173	8.71E-21	4.85E-20	36.06579	up
GOBP_DEC	-0.52631	-0.09988	-11.1169	3.85E-21	2.20E-20	36.87985	down
HP_RHIZO	0.5263	0.002687	12.57463	5.67E-25	4.21E-24	45.68465	up
GOBP_HIST	0.526253	-0.00478	13.57257	1.39E-27	1.23E-26	51.69338	up
GOBP_ISO	-0.5259	-0.05425	-19.9393	2.36E-43	5.94E-42	88.01197	down
REACTOM	0.525888	-0.06926	15.57141	9.85E-33	1.19E-31	63.54403	up
MORF_RFC	0.525868	-0.06081	13.06177	2.99E-26	2.44E-25	48.62262	up
GOBP_POS	-0.52586	0.132166	-9.76522	1.29E-17	5.73E-17	28.79544	down
REACTOM	0.525727	0.021812	16.85219	6.02E-36	8.92E-35	70.94627	up
TRAVAGLII	-0.52571	0.07105	-11.51	3.57E-22	2.19E-21	39.25186	down
BIOCARTA	-0.52556	0.033281	-12.1114	9.36E-24	6.42E-23	42.88605	down
GOBP_RES	-0.52548	-0.03854	-8.48013	2.38E-14	8.38E-14	21.31964	down
GOMF_ACT	-0.52546	-0.03342	-12.438	1.30E-24	9.42E-24	44.85968	down
GOBP_NEC	-0.52545	0.005425	-17.997	9.42E-39	1.68E-37	77.40895	down
GOBP_CHI	0.525443	0.057318	15.59221	8.72E-33	1.06E-31	63.66559	up
GOBP_CHI	0.525442	0.015423	20.90901	1.42E-45	4.31E-44	93.12854	up
SARRIO_EI	-0.52538	0.023027	-22.8537	7.12E-50	3.28E-48	103.0306	down
GOBP_MIT	0.525344	-0.11477	11.8652	4.16E-23	2.72E-22	41.3978	up
GOBP_CEL	-0.52528	0.113667	-12.4866	9.66E-25	7.07E-24	45.15306	down
GOBP_SHC	-0.52519	0.008481	-11.7962	6.31E-23	4.08E-22	40.9811	down
GOBP_POS	-0.52514	0.010456	-13.6023	1.16E-27	1.03E-26	51.87186	down
RIZ_ERYTH	0.525082	-0.02095	19.77411	5.69E-43	1.39E-41	87.12864	up
REACTOM	0.524853	-0.10343	11.65038	1.53E-22	9.59E-22	40.0997	up
CHIARETTI	0.524835	0.041347	13.72549	5.53E-28	5.01E-27	52.61011	up
GOMF_PR(	-0.52469	0.08539	-8.67235	7.88E-15	2.87E-14	22.41852	down
GOBP_LIPI	-0.52456	-0.0842	-21.6211	3.57E-47	1.24E-45	96.80995	down
MIR8074	-0.52454	0.100375	-13.3754	4.53E-27	3.89E-26	50.50943	down
GOCC_R2T	0.524293	-0.10834	7.493117	6.13E-12	1.80E-11	15.82227	up
GSE13547_	0.524285	0.009792	14.16588	3.98E-29	3.86E-28	55.2422	up
GOBP_CEN	0.524221	0.025654	20.06993	1.17E-43	3.04E-42	88.70824	up
DOANE_BI	-0.52417	-0.02129	-22.1936	1.94E-48	7.70E-47	99.72271	down
HP_ABNOI	0.524107	0.030097	19.58148	1.60E-42	3.77E-41	86.09407	up
MENON_F	0.524105	0.068175	17.11468	1.35E-36	2.08E-35	72.4415	up
GOMF_CO	-0.5241	-0.0312	-11.0616	5.38E-21	3.04E-20	36.54661	down
GOBP_RES	-0.52409	0.013678	-11.5938	2.15E-22	1.34E-21	39.75806	down
WP_GASTI	0.523985	-0.01094	18.32807	1.50E-39	2.85E-38	79.24933	up
GOBP_TRII	0.523798	-0.04986	10.65082	6.41E-20	3.36E-19	34.07657	up
KEGG_NOI	0.52369	-0.05598	12.13553	8.09E-24	5.58E-23	43.03176	up

AIZARANI_	-0.52358	0.069645	-12.0919	1.05E-23	7.21E-23	42.76791	down
MEBARKI_I	-0.52353	-0.00956	-17.0319	2.16E-36	3.31E-35	71.97096	down
GOBP_REG	0.523467	-0.06126	9.612135	3.20E-17	1.38E-16	27.89106	up
DAZARD_U	-0.5234	0.08116	-11.0113	7.29E-21	4.07E-20	36.2441	down
RUAN_RES	-0.52337	-0.09199	-16.9471	3.50E-36	5.29E-35	71.4876	down
GOBP_COI	0.523221	-0.12193	9.937567	4.62E-18	2.13E-17	29.81702	up
GOMF_ME	-0.52321	-0.0254	-11.0957	4.38E-21	2.49E-20	36.75231	down
MISHRA_C	-0.52321	-0.02561	-16.845	6.27E-36	9.28E-35	70.90504	down
HP_POLYN	-0.52318	-0.0344	-20.4018	2.02E-44	5.59E-43	90.46706	down
GOCC_TUI	0.523172	0.098675	8.527326	1.82E-14	6.45E-14	21.5887	up
FAELT_B_C	0.523115	-0.08153	15.56731	1.01E-32	1.22E-31	63.5201	up
MYLLYKAN	0.523088	-0.08224	8.443089	2.95E-14	1.03E-13	21.10883	up
REACTOM	0.523038	-0.04302	11.37982	7.85E-22	4.71E-21	38.46578	up
MODULE_4	-0.52298	0.10165	-10.291	5.57E-19	2.74E-18	31.92195	down
HP_CUTAN	0.522977	0.014963	9.172668	4.27E-16	1.71E-15	25.31334	up
REACTOM	0.522943	-0.00026	12.57629	5.62E-25	4.17E-24	45.69467	up
GOBP_IRO	-0.52256	-0.03127	-13.2818	7.96E-27	6.72E-26	49.94662	down
GOBP_AM	-0.52249	-0.04601	-12.6399	3.82E-25	2.87E-24	46.07841	down
GOBP_POS	0.522303	-0.03114	16.96034	3.25E-36	4.91E-35	71.56322	up
GOMF_CA	-0.52216	-0.08084	-20.7394	3.43E-45	1.01E-43	92.2421	down
GOBP_RES	-0.52211	-0.03675	-19.1247	1.89E-41	4.13E-40	83.62245	down
GOBP_AM	-0.52209	-0.18606	-9.56145	4.32E-17	1.85E-16	27.59233	down
BIOCARTA	0.522015	-0.07105	9.258093	2.59E-16	1.05E-15	25.81207	up
GOBP_DE_	-0.52199	-0.12538	-10.2511	7.08E-19	3.45E-18	31.68358	down
DACOSTA_	-0.52191	0.093534	-13.6148	1.08E-27	9.58E-27	51.94664	down
REACTOM	0.521887	-0.08925	8.071252	2.45E-13	7.96E-13	19.01065	up
HP_ABNOI	0.521827	-0.08165	9.893869	5.99E-18	2.73E-17	29.55766	up
HP_ABNOI	-0.52181	-0.00935	-19.3426	5.81E-42	1.32E-40	84.8048	down
HP_RENAL	-0.52173	-0.0716	-18.5807	3.71E-40	7.32E-39	80.64474	down
MIR6506_3	-0.52173	0.114744	-8.41787	3.41E-14	1.18E-13	20.96547	down
HP_ABNOI	-0.5217	0.008285	-18.2066	2.94E-39	5.46E-38	78.57556	down
GOBP_STE	-0.52153	-0.07433	-17.3061	4.56E-37	7.27E-36	73.52699	down
REACTOM	-0.52129	0.128775	-6.68933	4.56E-10	1.16E-09	11.57113	down
GOBP_PRC	-0.52127	-0.12699	-15.2784	5.48E-32	6.35E-31	61.82754	down
GOBP_NEC	-0.52127	0.012167	-13.6815	7.20E-28	6.48E-27	52.34664	down
REACTOM	-0.52112	-0.18833	-8.82348	3.28E-15	1.23E-14	23.28794	down
GOBP_MEI	0.520942	-0.00948	18.80751	1.07E-40	2.20E-39	81.89048	up
HP_ORCHI	-0.52083	0.080949	-9.71258	1.76E-17	7.77E-17	28.4841	down
GOBP_HIS	0.520824	0.011246	22.76714	1.10E-49	4.93E-48	102.6	up
QIU_PBMC	-0.52082	0.139103	-13.9826	1.19E-28	1.12E-27	54.1483	down
GOBP_SIAI	-0.5206	0.034368	-15.3596	3.40E-32	3.99E-31	62.30379	down
PUJANA_B	0.520559	0.00622	18.0817	5.88E-39	1.07E-37	77.88119	up
WENG_PO	-0.52056	-0.1658	-10.2523	7.03E-19	3.42E-18	31.69111	down
FARDIN_H	-0.52045	-0.07382	-7.97628	4.18E-13	1.34E-12	18.48035	down
GOBP_CAT	-0.52036	0.045157	-24.6351	1.24E-53	8.18E-52	111.6876	down
REACTOM	0.520277	-0.09118	13.87242	2.30E-28	2.14E-27	53.48963	up
GOCC_H4_	0.520192	0.002854	17.09565	1.50E-36	2.32E-35	72.33331	up
GRUETZM/	-0.52019	-0.03926	-25.6202	1.22E-55	9.82E-54	116.3103	down
BMI1_DN_I	-0.51995	0.031521	-29.6438	2.38E-63	3.98E-61	134.0446	down
HP_ABNOI	0.519819	0.005123	15.91794	1.31E-33	1.67E-32	65.56351	up
GOCC_DN	0.519788	0.094932	13.45692	2.77E-27	2.42E-26	50.99923	up
GOMF_MC	-0.51977	0.029519	-12.6553	3.48E-25	2.63E-24	46.17174	down
HP_URETH	0.51974	-0.04138	19.06808	2.58E-41	5.52E-40	83.31405	up
GOCC_RES	-0.51973	-0.21025	-10.3435	4.07E-19	2.01E-18	32.23603	down
GOCC_KIN	0.519702	0.025086	23.31497	7.29E-51	3.65E-49	105.3097	up
GOBP_REG	-0.51962	-0.08873	-12.4262	1.39E-24	1.01E-23	44.78794	down
REACTOM	0.519592	-0.09235	7.376002	1.16E-11	3.33E-11	15.18874	up
GOBP_POS	-0.51947	0.085052	-12.1882	5.88E-24	4.09E-23	43.35037	down

GNF2_TDG	0.519405	0.047281	9.397586	1.14E-16	4.74E-16	26.62897	up
GOBP_PRC	0.51917	0.07462	12.74564	2.02E-25	1.55E-24	46.71682	up
GOBP_COI	-0.51909	0.118953	-9.08807	7.01E-16	2.75E-15	24.82062	down
GOMF_CO	-0.51909	0.118953	-9.08807	7.01E-16	2.75E-15	24.82062	down
GOBP_DEF	-0.51906	0.077441	-19.453	3.20E-42	7.40E-41	85.40134	down
GOBP_REG	0.519021	-0.02403	10.10312	1.72E-18	8.14E-18	30.80148	up
MOOTHA_	-0.51898	0.036992	-9.40335	1.10E-16	4.59E-16	26.66281	down
GOBP_DN/	0.51896	-0.02384	16.13941	3.62E-34	4.77E-33	66.84789	up
GOMF_HY	-0.51891	0.188548	-8.82636	3.23E-15	1.21E-14	23.30455	down
MORI_EMU	0.518829	-0.04008	17.98207	1.02E-38	1.82E-37	77.32585	up
GOBP_TET	-0.51878	-0.06859	-12.9824	4.83E-26	3.88E-25	48.14458	down
HP_PERITC	0.518681	0.006557	11.39959	6.97E-22	4.19E-21	38.58507	up
MYLLYKAN	-0.51862	0.154348	-7.3924	1.06E-11	3.06E-11	15.27718	down
HP_OPTIC_	0.518608	0.031889	15.67796	5.29E-33	6.53E-32	64.16623	up
FARMER_B	-0.51851	-0.06669	-13.1583	1.67E-26	1.39E-25	49.20359	down
HP_ABNOI	-0.51845	-0.13911	-13.9155	1.77E-28	1.66E-27	53.74696	down
HP_DEEP_I	0.518447	0.01065	28.7754	9.47E-62	1.32E-59	130.3665	up
REACTOM	0.518412	-0.0117	17.92735	1.39E-38	2.46E-37	77.02038	up
HP_BIFID_I	-0.51837	0.10769	-8.81508	3.45E-15	1.29E-14	23.23946	down
GOBP_REG	-0.51832	0.146948	-9.19036	3.85E-16	1.54E-15	25.41653	down
GOCC_RN/	0.518309	-0.01248	12.10306	9.84E-24	6.75E-23	42.83551	up
HP_SCHW/	0.518302	-0.10226	10.15991	1.22E-18	5.86E-18	31.13986	up
GOMF_MA	-0.51829	-0.04511	-9.99026	3.37E-18	1.57E-17	30.13002	down
HP_ABNOI	-0.51826	0.091028	-10.2714	6.27E-19	3.07E-18	31.80483	down
GOBP_SEC	-0.51823	-0.05244	-21.7213	2.14E-47	7.60E-46	97.32279	down
GOBP_MO	-0.51821	-0.02806	-11.7449	8.61E-23	5.52E-22	40.67059	down
HP_ABNOI	-0.51813	-0.03844	-17.6329	7.23E-38	1.22E-36	75.37039	down
MORF_CDI	0.518022	-0.06074	13.52783	1.81E-27	1.60E-26	51.42493	up
GOBP_NEC	-0.51793	0.052881	-6.67086	5.02E-10	1.27E-09	11.47628	down
ERWIN_CC	-0.51782	-0.00331	-9.47186	7.34E-17	3.10E-16	27.06515	down
GOMF_FO	-0.51773	-0.06441	-11.1168	3.85E-21	2.20E-20	36.87935	down
KAUFFMAI	0.517685	-0.06955	17.01931	2.32E-36	3.54E-35	71.89914	up
GOBP_RIB(	-0.51761	0.026502	-24.7679	6.60E-54	4.45E-52	112.3178	down
MIR381_5I	0.517562	0.037801	12.80935	1.37E-25	1.07E-24	47.10115	up
REACTOM	0.517348	-0.04731	18.4038	9.85E-40	1.90E-38	79.66835	up
GSE21546_	0.517332	-0.07377	16.43997	6.39E-35	8.84E-34	68.58271	up
GOBP_CEL	0.517131	0.091759	10.26307	6.59E-19	3.22E-18	31.75528	up
TIAN_TNF_	-0.51711	0.103373	-11.7999	6.17E-23	4.00E-22	41.00335	down
REACTOM	-0.51703	-0.0749	-13.377	4.49E-27	3.85E-26	50.51889	down
ELLWOOD	-0.51679	-0.03737	-21.3743	1.27E-46	4.20E-45	95.54144	down
GOBP_CEL	-0.51673	0.045044	-11.8183	5.52E-23	3.59E-22	41.11446	down
GOBP_REG	-0.51659	-0.02666	-15.2432	6.74E-32	7.77E-31	61.62084	down
GOBP_INT	-0.51652	-0.01519	-18.9355	5.31E-41	1.12E-39	82.59066	down
GOMF_LIP	-0.51652	-0.01519	-18.9355	5.31E-41	1.12E-39	82.59066	down
WOTTON_	-0.51651	0.045279	-15.1231	1.37E-31	1.54E-30	60.91424	down
GOMF_DE/	0.516476	0.044677	10.6405	6.82E-20	3.57E-19	34.01464	up
MANNO_N	0.516437	0.021403	24.48284	2.56E-53	1.65E-51	110.963	up
ZNF354C_	0.516337	0.041762	6.534586	1.02E-09	2.52E-09	10.78095	up
HAHTOLA_	-0.51629	0.085407	-13.9011	1.93E-28	1.81E-27	53.66094	down
GOBP_POS	-0.51629	0.004378	-14.8487	6.89E-31	7.49E-30	59.29584	down
GOBP_RES	-0.51611	0.052472	-9.84862	7.84E-18	3.55E-17	29.28934	down
GOMF_FRU	-0.5161	-0.04746	-10.9057	1.38E-20	7.56E-20	35.60815	down
GOBP_CEL	-0.51597	0.228089	-9.04104	9.24E-16	3.59E-15	24.54727	down
KEGG_LYSI	-0.51591	-0.08387	-14.0622	7.38E-29	7.06E-28	54.62384	down
GOBP_POS	-0.51585	0.009896	-24.7194	8.31E-54	5.56E-52	112.0879	down
KEGG_PAR	-0.51567	-0.16236	-11.6947	1.17E-22	7.40E-22	40.3672	down
HAY_BONI	-0.51566	0.073439	-11.879	3.82E-23	2.51E-22	41.48107	down
HP_MITOC	-0.51561	-0.16235	-11.6222	1.81E-22	1.13E-21	39.92927	down

WONG_EN	-0.51557	0.046531	-9.14215	5.11E-16	2.03E-15	25.13548	down
GCM_PPP1	0.515565	-0.07243	10.58452	9.56E-20	4.95E-19	33.67884	up
GINESTIER	0.515558	-0.08168	17.51025	1.44E-37	2.38E-36	74.67991	up
GOBP_COI	-0.51555	0.071486	-9.713	1.76E-17	7.75E-17	28.48656	down
REACTOM	0.51545	0.046356	13.0148	3.97E-26	3.21E-25	48.33971	up
GOBP_HIS	0.515309	-0.00583	11.22494	2.00E-21	1.17E-20	37.53131	up
BIOCARTA	0.515287	0.05585	12.93378	6.48E-26	5.14E-25	47.85146	up
GOBP_REG	-0.51525	0.045338	-11.1217	3.74E-21	2.14E-20	36.90917	down
GOBP_NEC	0.515203	0.070664	9.841455	8.19E-18	3.70E-17	29.24687	up
SGCGSSAA	0.51517	0.005133	20.93382	1.24E-45	3.80E-44	93.25792	up
REACTOM	-0.51507	-0.08413	-14.0655	7.24E-29	6.93E-28	54.64324	down
chr4q35	-0.51505	0.052039	-11.9265	2.87E-23	1.90E-22	41.76839	down
HALLMARI	0.514892	0.038999	24.12749	1.40E-52	8.31E-51	109.2605	up
GOBP_PRC	0.514885	0.033625	10.0736	2.05E-18	9.67E-18	30.62574	up
GOBP_ATF	-0.51488	-0.20789	-9.97785	3.63E-18	1.68E-17	30.05632	down
CADWELL_	-0.51483	-0.01192	-26.3235	4.81E-57	4.37E-55	119.5409	down
GOBP_REG	0.514721	-0.06253	12.22186	4.79E-24	3.36E-23	43.55355	up
GOBP_PRC	0.514666	-0.1014	9.038846	9.35E-16	3.64E-15	24.53452	up
GOBP_POS	-0.51444	0.160423	-8.93361	1.73E-15	6.61E-15	23.92432	down
GOMF_CX(	-0.51442	0.163258	-10.5115	1.48E-19	7.57E-19	33.24134	down
GOBP_SIGI	0.514223	0.003282	20.74132	3.40E-45	1.00E-43	92.25232	up
REACTOM	-0.5142	-0.22309	-9.78432	1.15E-17	5.14E-17	28.90845	down
GOBP_POS	0.514178	-0.08414	14.34472	1.37E-29	1.37E-28	56.30738	up
WP_HISTO	0.514115	-0.00133	14.74376	1.28E-30	1.37E-29	58.67534	up
NELSON_R	-0.5141	0.000231	-20.5632	8.65E-45	2.45E-43	91.31781	down
GOBP_POS	-0.51403	-0.04955	-20.599	7.17E-45	2.06E-43	91.50583	down
GOBP_NEL	-0.51395	-0.00782	-10.1402	1.38E-18	6.57E-18	31.0225	down
REACTOM	0.513925	-0.14475	9.916128	5.25E-18	2.40E-17	29.68975	up
HP_SPONT	-0.51373	-0.04497	-20.0302	1.45E-43	3.73E-42	88.49699	down
GOBP_RES	-0.5137	0.128125	-16.931	3.84E-36	5.77E-35	71.39575	down
HP_ANAPH	0.513655	0.190552	8.876149	2.42E-15	9.13E-15	23.59198	up
GOBP_RES	-0.51364	0.018738	-9.18914	3.88E-16	1.55E-15	25.40942	down
WP_PHOT(	-0.51354	-0.05921	-14.5134	5.02E-30	5.15E-29	57.30983	down
GOBP_CH(	-0.51338	-0.07673	-21.0049	8.60E-46	2.65E-44	93.62811	down
GOMF_PH(	-0.51336	-0.05779	-7.2477	2.34E-11	6.55E-11	14.49994	down
GOMF_OLI	-0.51334	-0.08882	-12.3133	2.76E-24	1.96E-23	44.10617	down
GOMF_GLI	-0.51333	-0.15214	-8.57016	1.42E-14	5.08E-14	21.83331	down
KEGG_SYS	-0.51323	0.046924	-10.7309	3.96E-20	2.10E-19	34.5574	down
ANDERSEN	0.513148	-0.03927	9.851986	7.69E-18	3.48E-17	29.30929	up
LOPEZ_ME	0.513123	0.010022	11.77618	7.13E-23	4.59E-22	40.85985	up
GOCC_CUI	0.513083	-0.13954	9.623502	2.99E-17	1.30E-16	27.9581	up
GOBP_POS	-0.51293	0.111077	-19.7953	5.08E-43	1.25E-41	87.24236	down
GOBP_NEC	-0.51271	0.153988	-11.3403	9.97E-22	5.93E-21	38.22749	down
REACTOM	-0.51262	-0.03648	-12.5693	5.86E-25	4.34E-24	45.65273	down
SOTIRIOU_	-0.51261	0.038094	-15.1495	1.17E-31	1.33E-30	61.06956	down
MODULE_	-0.51249	0.057249	-12.9576	5.61E-26	4.48E-25	47.99512	down
GOMF_TR/	-0.51248	0.159385	-11.0737	5.00E-21	2.83E-20	36.61962	down
YAO_HOX/	-0.51225	0.018511	-20.5954	7.31E-45	2.09E-43	91.48665	down
HOEK_T_C	0.51224	-0.05857	9.74347	1.47E-17	6.50E-17	28.66674	up
GOBP_REG	0.512206	-0.04376	11.55564	2.71E-22	1.68E-21	39.52733	up
GOBP_DIA	-0.51216	0.064177	-10.8488	1.94E-20	1.05E-19	35.26612	down
GOCC_MI(	-0.51211	-0.12409	-14.8581	6.52E-31	7.10E-30	59.35139	down
GOBP_LUN	-0.51203	0.178164	-8.61709	1.08E-14	3.91E-14	22.10179	down
HP_CONJL	-0.51201	-0.02149	-12.2335	4.47E-24	3.14E-23	43.62385	down
GOCC_NU	0.51182	-0.16213	9.954824	4.16E-18	1.92E-17	29.9195	up
GOBP_SKE	-0.51181	0.142792	-9.9503	4.28E-18	1.97E-17	29.89262	down
GOCC_PO:	-0.51174	-0.0408	-8.72086	5.95E-15	2.19E-14	22.69708	down
GOBP_TRA	0.511721	0.053343	12.62267	4.24E-25	3.17E-24	45.9747	up

REACTOM	0.511648	-0.0866	14.22694	2.76E-29	2.71E-28	55.60609	up
FAN_OVAI	-0.51146	-0.00237	-18.7002	1.92E-40	3.86E-39	81.30205	down
GOBP_GAL	-0.51141	-0.02388	-10.738	3.79E-20	2.02E-19	34.59988	down
HP_RENAL	0.511253	0.022362	13.59622	1.20E-27	1.07E-26	51.83525	up
GOMF_SIA	-0.51118	0.040344	-15.2357	7.04E-32	8.11E-31	61.57655	down
GOBP_CEL	-0.51106	0.036892	-18.1641	3.72E-39	6.84E-38	78.33975	down
GOBP_POS	0.511038	-0.06763	10.62561	7.46E-20	3.90E-19	33.9253	up
WP_THIAN	-0.51102	-0.04755	-9.88933	6.15E-18	2.80E-17	29.53077	down
GOBP_REG	0.510858	0.094058	10.06816	2.12E-18	9.98E-18	30.59336	up
GOMF_CA1	0.510854	-0.03891	19.28799	7.81E-42	1.76E-40	84.50885	up
GOBP_MIT	-0.51067	-0.08324	-6.42925	1.75E-09	4.24E-09	10.24883	down
MARTIN_N	-0.51065	0.079283	-16.8091	7.70E-36	1.13E-34	70.70022	down
GSE21546_	0.510584	-0.06207	15.5285	1.27E-32	1.52E-31	63.29318	up
GOBP_INT	-0.51058	-0.00036	-15.9769	9.29E-34	1.20E-32	65.90569	down
BOCHKIS_I	-0.51053	-0.06859	-25.1342	1.17E-54	8.42E-53	114.0442	down
GOBP_RES	0.510475	-0.03011	9.99219	3.33E-18	1.55E-17	30.14152	up
GOBP_REG	-0.51045	0.045698	-9.39867	1.13E-16	4.71E-16	26.63534	down
REACTOM	0.510414	-0.09592	12.78913	1.55E-25	1.20E-24	46.97923	up
MODULE_2	-0.51025	-0.0954	-11.0503	5.76E-21	3.25E-20	36.47896	down
HP_MEGAI	-0.51022	-0.04951	-10.7263	4.07E-20	2.16E-19	34.52969	down
GOBP_REG	-0.51015	0.116224	-9.763	1.31E-17	5.81E-17	28.78229	down
HP_RECUR	-0.51015	0.113295	-8.00326	3.59E-13	1.16E-12	18.63079	down
GOBP_MEI	-0.51012	-0.03514	-10.8973	1.45E-20	7.94E-20	35.55757	down
GOMF_LIP	-0.51011	0.117075	-8.4847	2.32E-14	8.17E-14	21.34565	down
GOBP_POS	-0.51011	0.169862	-10.3404	4.14E-19	2.05E-18	32.21713	down
GOBP_POS	-0.51011	0.173552	-8.83793	3.02E-15	1.13E-14	23.37132	down
SPIRA_SM(	-0.51005	0.095215	-14.474	6.35E-30	6.47E-29	57.07565	down
GOBP_DN/	-0.50981	-0.11161	-10.3416	4.12E-19	2.03E-18	32.22418	down
REACTOM	-0.50969	-0.10339	-8.27463	7.72E-14	2.61E-13	20.15405	down
GOBP_TEL	0.509659	-0.04476	16.98263	2.86E-36	4.34E-35	71.69022	up
GOBP_POS	0.509525	0.041971	19.06835	2.57E-41	5.52E-40	83.31551	up
MOOTHA_	-0.5095	-0.01886	-18.8496	8.49E-41	1.76E-39	82.12084	down
GOMF_PO	-0.50944	0.031532	-16.7367	1.16E-35	1.69E-34	70.28616	down
ZFP36L1_T	-0.50939	-0.1143	-12.2401	4.29E-24	3.02E-23	43.66388	down
GSE17301_	0.509262	-0.06812	22.4461	5.45E-49	2.25E-47	100.9946	up
GOBP_ECT	0.509118	-0.01954	9.135872	5.30E-16	2.10E-15	25.09889	up
CAFFAREL_	-0.50909	-0.09885	-10.5681	1.05E-19	5.45E-19	33.58029	down
AIZARANI_	-0.50901	0.110707	-10.7726	3.08E-20	1.65E-19	34.80765	down
GOBP_ME1	-0.50899	-0.08242	-12.4077	1.56E-24	1.13E-23	44.67675	down
HP_HYPOI	0.508947	-0.09201	12.52975	7.44E-25	5.47E-24	45.41366	up
GOBP_REG	0.508923	-0.06534	13.9037	1.90E-28	1.78E-27	53.67668	up
HP_CEREBI	-0.5088	-0.12192	-9.04227	9.17E-16	3.57E-15	24.55443	down
GSE18893_	0.508717	-0.12496	12.10244	9.88E-24	6.77E-23	42.8318	up
GOMF_CA	-0.50846	-0.07078	-16.2657	1.75E-34	2.35E-33	67.57814	down
GOCC_PEI	0.508436	0.025909	7.512027	5.53E-12	1.62E-11	15.92497	up
FERRARI_R	-0.5084	0.086472	-12.0285	1.55E-23	1.04E-22	42.38503	down
REACTOM	-0.50837	-0.06474	-12.3855	1.78E-24	1.28E-23	44.54241	down
HP_BICORI	0.508356	0.005198	14.29708	1.82E-29	1.80E-28	56.02386	up
WAKABAY.	-0.50823	-0.08716	-19.7765	5.62E-43	1.37E-41	87.14164	down
SCHUHMA	-0.50818	0.076646	-9.20824	3.47E-16	1.39E-15	25.5209	down
BERTUCCI_	-0.50812	-0.00904	-12.9076	7.59E-26	5.99E-25	47.69336	down
REACTOM	-0.50807	0.024862	-12.9653	5.36E-26	4.29E-25	48.04135	down
GOBP_NE(	-0.50806	-0.00373	-22.6722	1.76E-49	7.68E-48	102.1264	down
HP_HYPOI	-0.508	-0.03878	-13.0087	4.12E-26	3.32E-25	48.30308	down
GSE25146_	0.507989	0.004689	22.7109	1.45E-49	6.39E-48	102.3197	up
GOMF_CA	-0.50717	0.076869	-12.0204	1.62E-23	1.10E-22	42.33612	down
GOBP_POS	-0.50711	0.064072	-13.6958	6.61E-28	5.96E-27	52.43195	down
GOBP_REG	-0.50698	0.107853	-15.9525	1.07E-33	1.38E-32	65.76445	down

GOBP_DOI	-0.50695	0.033604	-11.7994	6.19E-23	4.01E-22	41.00038	down
HP_ABSEN	0.506932	-0.00332	10.92034	1.26E-20	6.94E-20	35.69626	up
HP_CHROI	0.506849	-0.04333	14.4213	8.69E-30	8.80E-29	56.76274	up
DODD_NA	0.506825	0.003891	19.35622	5.40E-42	1.23E-40	84.87836	up
KEGG_PAN	-0.50662	-0.0331	-12.1663	6.71E-24	4.66E-23	43.21768	down
GOBP_PYR	0.5066	-0.15125	9.461884	7.78E-17	3.28E-16	27.00651	up
XU_HGF_T/	0.506581	0.012175	14.2174	2.92E-29	2.86E-28	55.54926	up
GOBP_NEC	-0.50642	-0.0471	-10.8811	1.60E-20	8.72E-20	35.4603	down
HP_APLAS	0.506409	-0.01667	13.51139	2.00E-27	1.76E-26	51.32628	up
MIR5705	-0.50635	0.055456	-9.5711	4.08E-17	1.75E-16	27.64916	down
GOBP_SPE	0.506334	0.030596	14.86705	6.18E-31	6.74E-30	59.40443	up
GOBP_POS	0.506282	-0.09971	8.676274	7.70E-15	2.81E-14	22.44103	up
WP_RAS_A	-0.50624	0.026867	-20.7538	3.19E-45	9.40E-44	92.31739	down
INAMURA_	0.506184	0.012438	9.5429	4.82E-17	2.06E-16	27.48307	up
GOMF_LO'	-0.50613	0.01559	-11.965	2.27E-23	1.51E-22	42.00123	down
GOBP_SYN	0.506088	-0.11658	14.81888	8.22E-31	8.90E-30	59.11973	up
GOBP_RES	-0.50608	0.001311	-19.1292	1.85E-41	4.03E-40	83.64664	down
MAGRANG	-0.50601	0.077767	-18.1424	4.19E-39	7.69E-38	78.21877	down
BIOCARTA	-0.50567	0.135796	-11.7899	6.56E-23	4.24E-22	40.94262	down
GOBP_SM(	-0.50562	0.253159	-8.45746	2.71E-14	9.51E-14	21.19056	down
REACTOM	0.505439	-0.09196	11.96973	2.21E-23	1.47E-22	42.02965	up
GOCC_SPII	0.505438	-0.02405	19.67403	9.74E-43	2.34E-41	86.59169	up
GOBP_CHI	0.505437	0.000215	22.15276	2.39E-48	9.39E-47	99.51648	up
MODULE_:	-0.5052	-0.027	-26.3633	4.01E-57	3.67E-55	119.7217	down
GOMF_NU	-0.50494	-0.01486	-10.5763	1.00E-19	5.19E-19	33.62976	down
GOBP_PRC	-0.50486	-0.11551	-13.4094	3.69E-27	3.19E-26	50.7138	down
HP_ABNOI	-0.50485	-0.17951	-11.2736	1.49E-21	8.75E-21	37.8246	down
GOMF_DN	0.504848	-0.09104	7.529794	5.01E-12	1.48E-11	16.02158	up
GOBP_RES	-0.50472	-0.02753	-18.5526	4.33E-40	8.52E-39	80.48984	down
GOBP_PRC	0.504698	-0.048	12.25606	3.90E-24	2.75E-23	43.76021	up
HP_APLAS	0.504688	0.111603	12.23418	4.45E-24	3.12E-23	43.628	up
WP_MAMI	-0.50467	0.071852	-10.434	2.36E-19	1.19E-18	32.77719	down
HP_INCRE/	-0.50466	-0.17657	-10.7027	4.69E-20	2.48E-19	34.3883	down
GOBP_REG	0.504646	-0.01798	19.10689	2.09E-41	4.53E-40	83.52535	up
GOMF_INS	-0.50463	0.012282	-8.67784	7.63E-15	2.79E-14	22.45002	down
GOBP_RES	-0.5046	0.111665	-10.7733	3.07E-20	1.64E-19	34.81187	down
GOBP_REG	-0.50433	0.001137	-11.0153	7.12E-21	3.98E-20	36.2679	down
LIU_VAV3_	-0.50424	0.087402	-17.3303	3.98E-37	6.37E-36	73.66364	down
GOBP_NEC	-0.50422	-0.05434	-11.995	1.89E-23	1.27E-22	42.18229	down
BUSSLINGI	-0.50419	-0.01683	-21.5571	4.96E-47	1.70E-45	96.48161	down
GSE22886_	0.50403	-0.0677	13.30182	7.05E-27	5.98E-26	50.06723	up
GOBP_GAS	-0.50401	0.091954	-10.6898	5.07E-20	2.68E-19	34.31081	down
GOUYER_T	-0.50397	0.042114	-11.272	1.51E-21	8.83E-21	37.8152	down
HP_DIGEN	-0.50397	0.135937	-12.205	5.31E-24	3.71E-23	43.45188	down
DESCARTE	-0.50384	0.1456	-11.7841	6.79E-23	4.38E-22	40.90756	down
GOBP_NEC	-0.50376	0.069107	-14.0926	6.15E-29	5.92E-28	54.80528	down
KORKOLA_	-0.50373	0.080151	-11.1845	2.56E-21	1.48E-20	37.28744	down
PUJANA_C	0.503589	-0.03489	14.10504	5.72E-29	5.50E-28	54.87932	up
GOBP_FAT	-0.50344	-0.05577	-22.6251	2.22E-49	9.58E-48	101.8912	down
CAO_BLO(	-0.50339	0.226976	-8.57801	1.36E-14	4.86E-14	21.87821	down
GOBP_CEL	-0.5033	-0.09645	-20.1246	8.78E-44	2.30E-42	88.9988	down
GOMF_ATI	-0.50324	0.043987	-9.59917	3.45E-17	1.49E-16	27.81462	down
THAKAR_P	-0.50312	0.130258	-9.22252	3.19E-16	1.29E-15	25.60423	down
YAO_TEMI	-0.50303	-0.01127	-20.7871	2.68E-45	7.96E-44	92.49211	down
GOBP_POS	-0.503	0.226992	-8.3049	6.50E-14	2.21E-13	20.32514	down
GOBP_REG	-0.50298	0.100225	-10.1364	1.41E-18	6.71E-18	30.99974	down
GOBP_MA	-0.5029	0.08749	-8.30416	6.52E-14	2.22E-13	20.32096	down
GOBP_TET	-0.50286	-0.05617	-13.8631	2.43E-28	2.25E-27	53.43382	down

REACTOM	-0.50283	0.081518	-9.62651	2.94E-17	1.27E-16	27.97587	down
GOBP_SEM	0.5026	0.033809	10.89438	1.48E-20	8.07E-20	35.54007	up
HP_PROGF	-0.50248	-0.08006	-9.98585	3.46E-18	1.61E-17	30.10384	down
GOBP_ADH	-0.50241	0.035452	-13.477	2.46E-27	2.15E-26	51.11989	down
GOMF_OP	-0.50228	0.022015	-11.1416	3.32E-21	1.90E-20	37.02878	down
GOCC_CAI	-0.50225	0.087945	-8.40236	3.72E-14	1.29E-13	20.87735	down
DESCARTE	-0.50219	0.12702	-12.5186	7.96E-25	5.84E-24	45.34616	down
REACTOM	-0.50214	-0.05186	-12.5537	6.44E-25	4.75E-24	45.55834	down
GOBP_LIPI	-0.50214	-0.03545	-26.0577	1.62E-56	1.39E-54	118.3264	down
GOBP_REG	-0.50201	-0.17808	-9.98043	3.57E-18	1.66E-17	30.07164	down
ABRAHAM	-0.50193	0.093348	-15.2695	5.78E-32	6.68E-31	61.77496	down
GOMF_CA	-0.50191	0.109017	-9.26087	2.54E-16	1.03E-15	25.82832	down
GOCC_CHI	0.501873	-0.08177	8.227792	1.01E-13	3.38E-13	19.88982	up
BONOME_	-0.50185	0.040377	-12.8422	1.13E-25	8.80E-25	47.2992	down
GOMF_PR(	-0.50169	0.095348	-11.5521	2.77E-22	1.71E-21	39.5061	down
TOMLINS_	0.501676	-0.04079	14.68232	1.84E-30	1.95E-29	58.31154	up
GOBP_ABS	0.501608	-0.18377	9.008078	1.12E-15	4.33E-15	24.35591	up
LU_TUMOI	-0.50156	0.126152	-11.3054	1.23E-21	7.26E-21	38.01673	down
HP_MEDUI	-0.50135	-0.03136	-11.3366	1.02E-21	6.06E-21	38.20481	down
GOBP_PLU	0.501275	-0.1011	8.365493	4.60E-14	1.58E-13	20.66821	up
BIOCARTA	0.501256	0.048796	16.15479	3.31E-34	4.37E-33	66.93685	up
GOBP_POS	0.501123	-0.13238	8.394818	3.89E-14	1.34E-13	20.83456	up
HP_FASTIN	-0.50098	-0.15751	-9.77913	1.19E-17	5.29E-17	28.87773	down
REACTOM	-0.50076	0.022151	-12.5353	7.19E-25	5.30E-24	45.44733	down
HP_JUNCT	0.500672	-0.07937	8.080485	2.32E-13	7.57E-13	19.06233	up
HP_NORM	0.500654	-0.02041	10.44596	2.20E-19	1.11E-18	32.8487	up
GOMF_DN	0.50061	-0.0644	16.23068	2.14E-34	2.85E-33	67.37567	up
GOBP_ISO	0.500579	0.098473	10.39028	3.07E-19	1.53E-18	32.51552	up
HP_HEMAI	-0.5005	0.121888	-12.3092	2.83E-24	2.01E-23	44.08124	down
WEST_ADF	-0.50046	0.009564	-22.5059	4.04E-49	1.71E-47	101.2947	down
GOBP_CEL	-0.50034	0.105176	-10.8483	1.95E-20	1.06E-19	35.26302	down
MIR6886_5	0.500319	0.00428	10.69308	4.97E-20	2.63E-19	34.33029	up
GOMF_STE	-0.50027	0.003145	-31.4134	1.66E-66	3.66E-64	141.3019	down
GOBP_MRI	0.500212	-0.03015	12.34917	2.22E-24	1.59E-23	44.32286	up
HP_TOXEN	-0.50018	-0.05028	-15.5497	1.12E-32	1.35E-31	63.41695	down
HP_SMOO	-0.50016	0.132655	-8.5767	1.37E-14	4.90E-14	21.87073	down
HP_ABNOI	0.500131	0.030242	10.41659	2.62E-19	1.32E-18	32.67293	up
